# Callose in leptoid cell walls of the moss *Polytrichum* and the evolution of callose synthase across bryophytes

**DOI:** 10.3389/fpls.2024.1357324

**Published:** 2024-02-07

**Authors:** Karen Renzaglia, Emily Duran, Laxmi Sagwan-Barkdoll, Jason Henry

**Affiliations:** ^1^ Southern Illinois University Carbondale, Department of Plant Biology, Carbondale, IL, United States; ^2^ Southeast Missouri University, Department of Biology, Cape Girardeau, MO, United States

**Keywords:** callose, callose synthase, cell walls, food-conducting cells, leptoid, plasmodesmata, *Polytrichum*, sieve plates

## Abstract

**Introduction:**

Leptoids, the food-conducting cells of polytrichaceous mosses, share key structural features with sieve elements in tracheophytes, including an elongated shape with oblique end walls containing modified plasmodesmata or pores. In tracheophytes, callose is instrumental in developing the pores in sieve elements that enable efficient photoassimilate transport. Aside from a few studies using aniline blue fluorescence that yielded confusing results, little is known about callose in moss leptoids.

**Methods:**

Callose location and abundance during the development of leptoid cell walls was investigated in the moss *Polytrichum* commune using aniline blue fluorescence and quantitative immunogold labeling (label density) in the transmission electron microscope. To evaluate changes during abiotic stress, callose abundance in leptoids of hydrated plants was compared to plants dried for 14 days under field conditions. A bioinformatic study to assess the evolution of callose within and across bryophytes was conducted using callose synthase (CalS) genes from 46 bryophytes (24 mosses, 15 liverworts, and 7 hornworts) and one representative each of five tracheophyte groups.

**Results:**

Callose abundance increases around plasmodesmata from meristematic cells to end walls in mature leptoids. Controlled drying resulted in a significant increase in label density around plasmodesmata and pores over counts in hydrated plants. Phylogenetic analysis of the CalS protein family recovered main clades (A, B, and C). Different from tracheophytes, where the greatest diversity of homologs is found in clade A, the majority of gene duplication in bryophytes is in clade B.

**Discussion:**

This work identifies callose as a crucial cell wall polymer around plasmodesmata from their inception to functioning in leptoids, and during water stress similar to sieve elements of tracheophytes. Among bryophytes, mosses exhibit the greatest number of multiple duplication events, while only two duplications are revealed in hornwort and none in liverworts. The absence in bryophytes of the CalS 7 gene that is essential for sieve pore development in angiosperms, reveals that a different gene is responsible for synthesizing the callose associated with leptoids in mosses.

## 1 Introduction

Studies of moss conducting-cells have targeted members of the Polytrichaceae because of their complex anatomy that resembles a protostele of tracheophytes (Pressel et al., 2006; Brodribb et al., 2020). In the stem of *Polytrichum*, a prominent central cylinder of water-conducting cells, the hydrome, is surrounded by the leptome, which contains the leptoids intermixed with parenchyma cells (Hébant, 1976; Ligrone and Duckett, 1994). Leptoids have been referred to as moss sieve elements because of their striking structural and developmental similarities to sieve cells of non-angiosperm tracheophytes (Stevenson, 1974; Scheirer, 1978). Among the most salient features shared across these specialized cells are the oblique overlapping end walls with numerous enlarged plasmodesmata (pores) that facilitate efficient transport of photosynthates from cell to cell (Hébant, 1974; Ligrone and Duckett, 1994). In sieve elements of tracheophytes, callose is an abundant cell wall polymer that is important to the expansion and development of plasmodesmata in end walls, including in the highly developed sieve plates of angiosperms (Pressel et al., 2006; Kalmbach and Helariutta, 2019). In contrast, the occurrence of callose in moss leptoids has been reported and postulated, but is highly understudied with no definitive micrographic support for precise location and developmental changes in cell walls (Stevenson, 1974; Scheirer, 1978). Although the intracellular organization in leptoids has been shown to be modified during water stress (Ligrone and Duckett, 1994; Pressel et al., 2006), nothing is known about cell wall modifications during leptoid development and during abiotic stress. The present study fills this void by presenting unequivocal data on the location and changes in callose abundance in leptoid cell walls during development and during water stress in the moss *Polytrichum commune*.

Because cell wall structure and composition are closely linked to many biological processes in plants (Amsbury et al., 2016), a deeper understanding of cell walls in physiologically significant cells such as those involved in translocation of photosynthate is essential to better understand structure-function relationships in mosses. Callose is a versatile cell wall polymer that plays a role in many essential plant functions involved in development and defense, including cell plate formation (Verma and Hong, 2001; Thiele et al., 2009), sieve pore development and regulation (Chen and Kim, 2009), transport through plasmodesmata (Iglesias and Meins, 2000; Wang et al., 2022; Li et al., 2023), pollen development and growth (McCormick, 1993; Chen and Kim, 2009), and biotic and abiotic stress responses (Bacic et al., 2009). This cell wall polymer is synthesized in specific tissues during plant growth and development. Callose is produced in the membrane by callose synthase using a substrate of UDP glucose (Chen and Kim, 2009). Structurally callose is a linear polymer of repeating 1-3-β-D-(with few 1-4 and 1-6) glucan residues taking on a helical conformation.

Studies on bryophytes have included callose in more comprehensive works on cell wall composition (Wallace et al., 2011; Berry et al., 2016; Henry and Renzaglia, 2021; Pfeifer et al., 2022), while only a few studies have focused specifically on callose (Scherp et al., 2002; Scherp et al., 2004; Schuette et al., 2009; Renzaglia et al., 2015; Renzaglia et al., 2020a; Muller et al., 2022). These studies identify callose as important in fundamental processes such as cell plate formation, spore development and plasmodesmata function. The present study uses aniline blue fluorescence and immunogold labeling in the transmission electron microscope (TEM) to address the following questions related to callose deposition in the developing leptoid cell walls of *P. commune*. What is the location and abundance of callose in meristematic cell walls and developing plasmodesmata? How does callose labeling density change during the development of leptoids? Does the abundance of callose labeling in leptoids change during development following water stress under field conditions? Our controlled study provides quantitative evidence that callose predominates and increases in association with plasmodesmata and leptoid cell wall during growth and development from the apical meristem. We further demonstrate that callose abundance is enhanced in plasmodesmata as they expand in leptoid cell walls following water stress, but there is no difference in pore size between hydrated and dehydrated plants.

To assess the evolution of the genes involved in callose synthesis, a key metabolic process that was important for plant diversification on land, we present an analysis of callose synthase (CalS) protein sequences in a wide range of bryophytes. Our phylogenetic analysis reveals independent evolutionary paths of the CalS gene family within each of the three groups of bryophytes and from those in tracheophytes. We recover three main clades of evolution in this gene family as has been shown in recent studies (Záveská Drábková and Honys, 2017; Ušák et al., 2023). Whereas the greatest diversity of CalS proteins in seed plants is seen in clade A, the majority of gene duplication in bryophytes has taken place in clade B. Moreover, multiple duplication events are seen in mosses, while only two duplications are revealed in hornwort and none in liverworts. The absence in bryophytes of CalS7, the gene associated with callose in sieve plates of angiosperms, identifies an independent derivation of the callose synthase gene associated with the highly specialized end walls of leptoids from those in food conducting cells of other land plants.

## 2 Materials and methods

### 2.1 Field experiments

Field experiments were conducted using shaded populations of *Polytrichum commune* on wooded bluff tops in the Shawnee National Forest of Jackson County, Illinois. Voucher specimens are deposited in the SIUC Plant Biology Herbarium.

To ensure adequate rain events to maintain hydration in control plants, experiments were conducted based on 10-day weather predictions during 2021 and 2022. For drying experiments, populations of *P. commune* greater than one square foot in size were covered by a three-by-three feet canopy to shelter from rain for 14 days. Hydrated plants were maintained under the same conditions minus the canopy and served as the control. Aggregates of plants, not individual plants, in each experimental plot were weighed before and after drying to avoid disruption of growth due to handling and to determine changes in weight due to water loss.

### 2.2 TEM preparation

TEM preparation was undertaking immediately after collection in the field and follows that outlined in Renzaglia et al. (2017). Ten stems were fixed for both hydrated and dried treatments during three independent fixations. For dehydrated plants, only visibly dried stems were sampled (**Supplementary Figure S1**). Stems were cut into two 1 cm portions, one at the apex and a second just below the apex. For orientation, the base of both stem segments was marked by an oblique cut. Samples were immediately fixed in 2.5% v/v glutaraldehyde in 0.5M Sorenson’s buffer (pH 7.2) for 1 h and overnight at 4°C to ensure maximum preservation of the tissue. Following 2-3 rinses in buffer for 15 min each, plants were post-fixed in 2% buffered osmium tetroxide and rinsed in autoclaved, distilled water. Specimens were dehydrated in a progressively higher ethanol to water concentrations and rinsed twice in 100% ethanol. Plants were infiltrated with LR White resin through progressive placement in higher concentrations of resin diluted with ethanol. Following 2 changes of 100% resin, plant segments were placed in gel capsules and heated in an oven at 60°C for 48 h. The samples were sectioned on an ultramicrotome, and thin sections (90-100 nm) were collected on 200 mesh nickel grids for immuno-labeling, or thick sections (1 μm) were collected on glass slides for aniline blue staining. Grids were not post-stained in order to readily visualize the gold particles. Because osmication was reduced and no post-staining was conducted, membranes and organelle visibility is decreased compared with standard TEM protocols. Grids were imaged on a Hitachi H7650 microscope and digital images were collected. TEM images were uploaded into ImageJ for measurements.

### 2.3 Immunogold labeling and histochemical staining

Specimens were processed as follows. Thin sections (90-100 nm) were collected on 200 mesh Ni grids, with 3 treatments and 2 controls processed for each block. Grids were blocked in BSA/PBS overnight in a humid chamber, and then maintained overnight at 4°C in a 1:20 ratio of anti-callose (monoclonal primary antibody to (1-3)-β-glucan purchased from Biosupplies Australia, Catalog No. 400-2) to BSA/PBS. Following 4 washes in BSA/PBS, grids were placed in the gold-conjugated anti-mouse secondary antibody for 1 h at room temperature and washed 4 times in PBS. Grids were rinsed in autoclaved distilled water, dried, imaged on the TEM and digital micrographs were collected.

### 2.4 Label density assessment

TEM micrographs were opened in the PhotoScapeX (Mooii Tech) editing application. Images were cropped to 800 x 800 pixels, and 4 counting frames 100 x 100 pixels in size were randomly placed onto the cell wall in the image, and labels within the frames were counted. This process was repeated for 21 images (84 counting frames) for both freshly collected and dried specimens. The number of labels in each counting frame was input into Microsoft Excel. Three zones were analyzed: the apex at the zone of active division, just below the zone of active division, and the mature region. Once all counts were completed, a two-tailed t-test of unequal variance was run to assess significant differences in label density between the freshly collected and dried groups in all three zones.

### 2.5 Aniline blue staining

Staining for callose was carried out on thick sections (1 μm) of material embedded in LR White resin. Samples were placed in 1% aniline blue (a fluorescent dye that localizes callose, Smith and McCully, 1978) in 0.067 M Na_2_HPO_4_ buffer (pH 8.5) in a dark, humid chamber at 4°C for 3 nights, followed by 3 rinses in buffer. Controls were placed in buffer without stain. All stained material was viewed with a Leica DM500B compound microscope (excitation filter ultraviolet fluorescence between 360-400 nm). Images were collected digitally using a Q-Imaging Retiga 2000R digital camera.​ Control sections lack fluorescence entirely at the apex or show general cell wall autofluorescence in mature stem regions and therefore are not included in the illustrations.

### 2.6 Phylogenetic reconstruction

Twelve callose synthase (CalS) protein sequences from *Arabidopsis thaliana* were downloaded from the TAIR database (https://www.arabidopsis.org/). CalS3, CalS5, CalS7, CalS8, CalS9, CalS10, CalS11, and CalS12 sequences from *A. thaliana* were used to construct CalS phylogenetic tree on bryophytes. CalS are homologous among mosses, liverwort, and hornwort and were identified using the blast search at the OneKP- The 1,000-plant project database (https://db.cngb.org/onekp/; Leebens-Mack et al., 2019; Carpenter et al., 2019). Only sequences with a low E value, a sequence similarity of 50%, and ≥1700 bp long were downloaded from the OneKP database (Leebens-Mack et al., 2019). Additional CalS homologous sequences were downloaded from well- assembled and annotated genomes of 46 bryophytes (24 mosses, 15 liverworts, and 7 hornworts), and five tracheophytes (*Selaginella bryopteris*, *Ceratopteris richardii*, *Gnetum montanum*, *Ginkgo biloba* and *Arabidopsis thaliana*) from NCBI (https://www.ncbi.nlm.nih.gov/) and Phytozome v13 databases (https://phytozome-next.jgi.doe.gov/). Duplicate sequences from all searches were deleted.

Amino acid sequences were aligned using MUSCLE method in MEGA11 (Tamura et al., 2021). The aligned CS sequences were used to construct a maximum likelihood tree with MEGA11 based on the Le et al. (2008) (LG) model. Initial tree(s) for the heuristic search were obtained automatically by applying Neighbor-Join and BioNJ algorithms to a matrix of pairwise distances estimated using the LG model and then selecting the topology with superior log likelihood value. A discrete Gamma distribution was used to model evolutionary rate differences among sites [5 categories (+G, parameter = 1.0909)]. The rate variation model allowed for some sites to be evolutionarily invariable ([+I], 2.74% sites). To estimate the reliability of each node, a bootstrap method was utilized with 100 replications. The final tree was annotated by FigTree (http://tree.bio.ed.ac.uk/software/figtree/) to highlight clades and nodes. All CS sequences used to construct the phylogenetic tree can be found in **Supplementary Files S2**, **S3**.

## 3 Results

Following 14 days in the field, dried plants demonstrated visible signs of dehydration compared with the robust hydrated plants (**Supplementary Figure S1**). Dried plants weighed 39% (range 27%-45%) less on average than they did when hydrated prior to drying. No weight change (range from 3% less than to 4% more than original weight) was observed in hydrated plants exposed to rain under field conditions.

**Figure 1:**
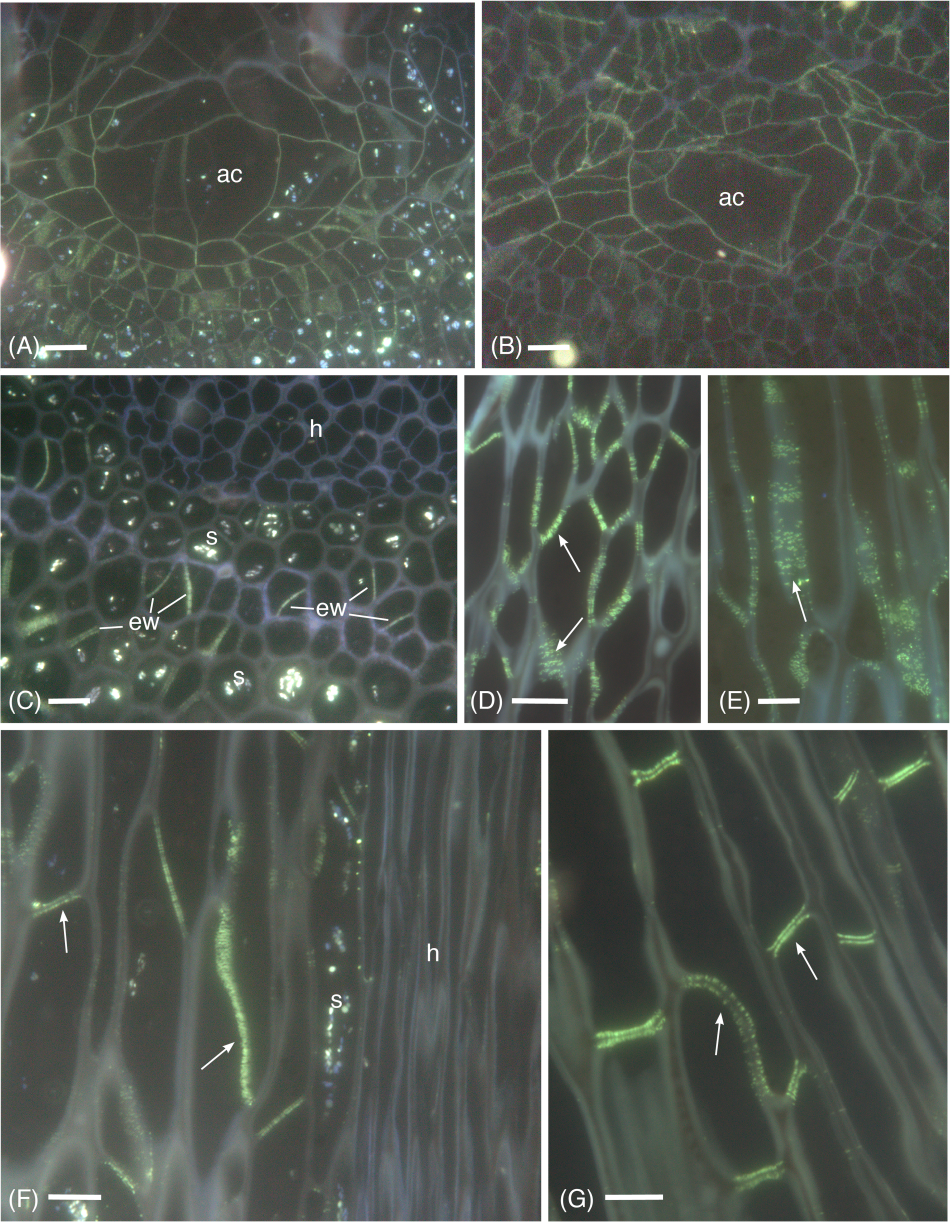
Aniline blue fluorescence of callose in apical sections of hydrated **(A, C, F)** and 14-day dried **(B, D, E, G)** gametophyte shoots of *P. commune*. **(A, B)** Nearly longitudinal section of the apical meristem showing diffuse fluorescence of the apical cell (ac) and derivatives. **(A)** Hydrated plant **(B)** Dried plant. **(C)** Cross section of hydrated plant showing developing leptoids with callose in end walls (ew) and central hydrome (h) lacking fluorescence. Associated parenchyma cells contain starch (s). **(D)** Callose (arrow) is abundant around plasmodesmata of unexpanded, differentiating leptoids in longitudinal section of dried plants. **(E)** Aggregates of plasmodesmata surrounded by callose (arrow) are prominent in elongating leptoids of dried plants. **(F)** In longitudinal section of a nearly mature hydrated stem, callose is evident in the end walls (arrows) and is less prominent in the region between cell walls of adjacent leptoids. Starch (s) is abundant in associated parenchyma. **(G)** In nearly mature leptoids of dried plants, callose (arrows) is abundant in end walls of adjacent cells and is absent from the zone between cells [Bars: 10 μm].

**Figure 2:**
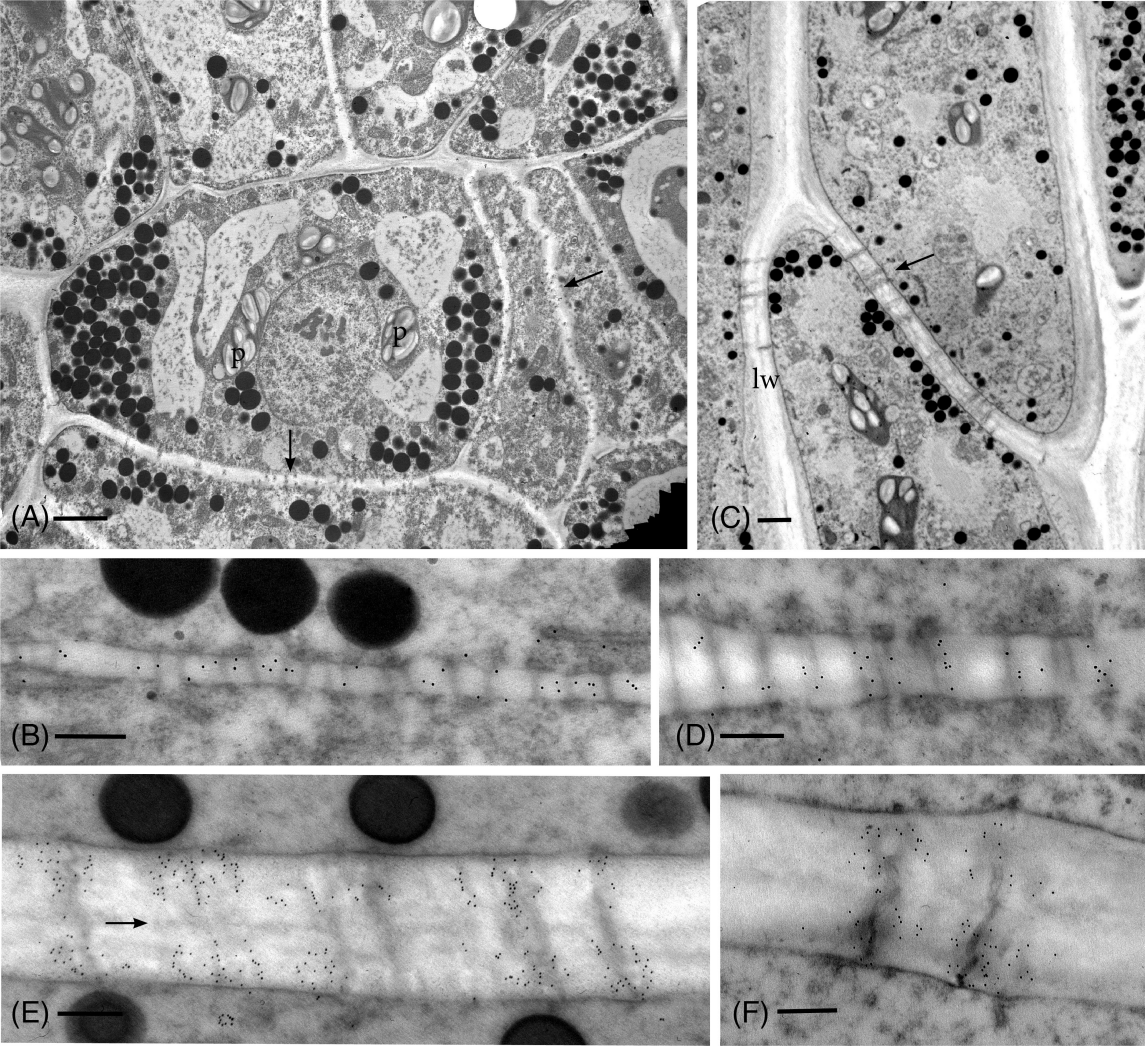
TEM micrographs showing callose labeling in apical region of hydrated plants. **(A)** Plasmodesmata are scattered and abundant in newly-formed thin primary cell walls (arrows). Cells contain dense cytoplasm with abundant oil droplets and starch-filled plastids (p). **(B)** Newly-formed cell wall with abundant plasmodesmata and sparce, scattered callose labels. **(C)** Differentiating leptoids showing PDs in oblique end walls (arrow) and lateral walls (lw). **(D)** Callose labeling is associated with elongating plasmodesmata in developing end walls of leptoids. **(E)** Callose localizes at the neck region of PDs in maturing leptoids and is sparce to absent in the zone between cells (arrow). **(F)** Plasmodesmata and callose labeling in the lateral wall are similar to those in end walls. [Bars: **(A)** = 2.0 μm, **(C)** =1.0 μm, **(B, D–F)** = 0.25 μm].

**Figure 3:**
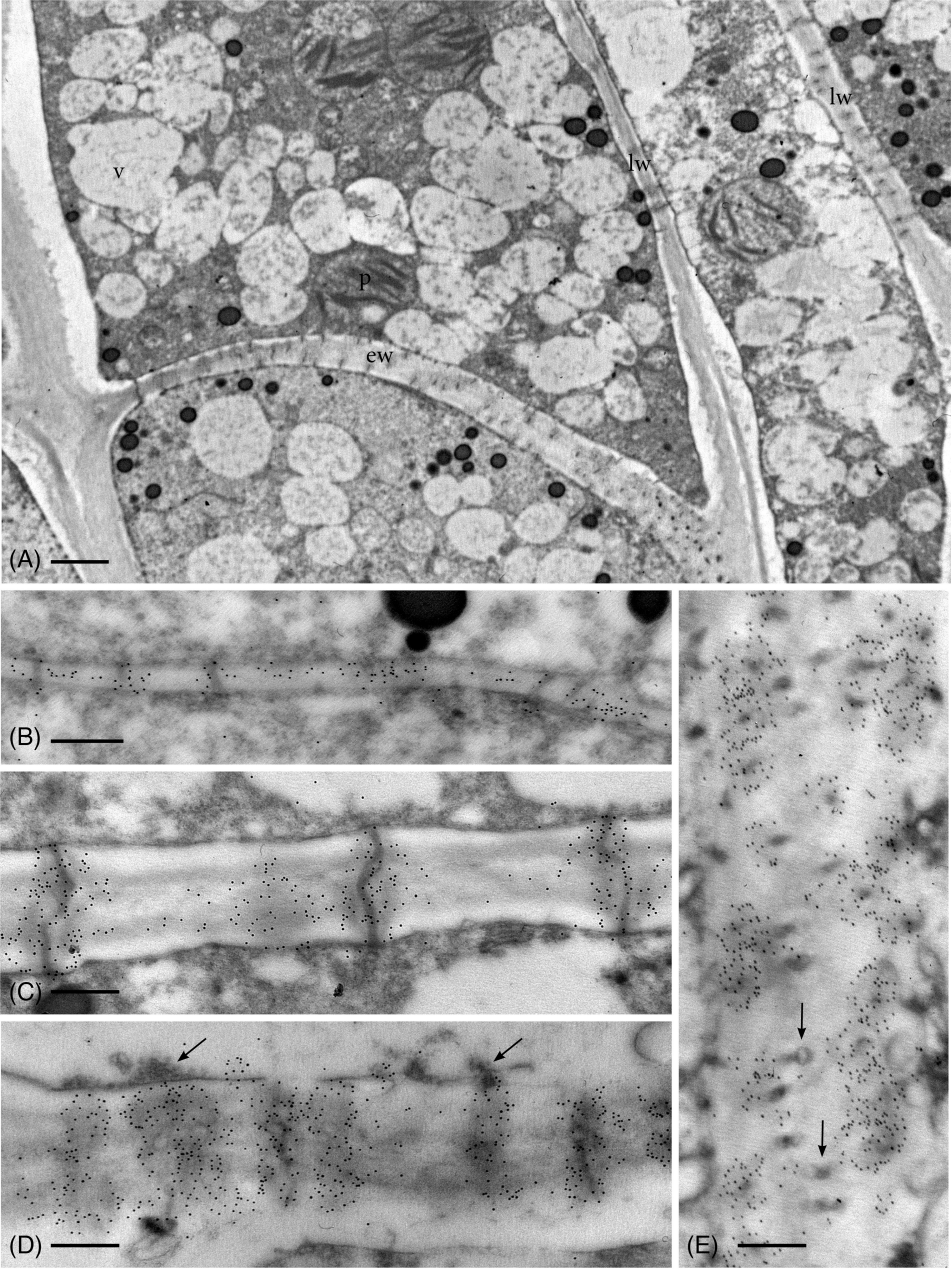
TEM micrographs showing callose labeling in apical region of leptoids in 14-day dried plants. **(A)** Oblique end walls (ew) and lateral walls (lw) of developing leptoids contain abundant plasmodesmata. **(B)** Callose labels are aggregated around plasmodesmata and are abundant throughout newly-formed thin primary cell walls. **(C)** Elongating PDs in developing leptoid end walls are surrounded by dense callose labels. **(D)** Enlarged PDs are surrounded by abundant callose and topped with dense granular plugs (arrows). **(E)** Paradermal section of a leptoid end wall showing callose encircling PDs in cross section, except at the middle lamellar region between adjacent cells (arrows). [Bar: **(A)** = 1.0 μm; **(B–E)** = 0.25 μm.

**Figure 4:**
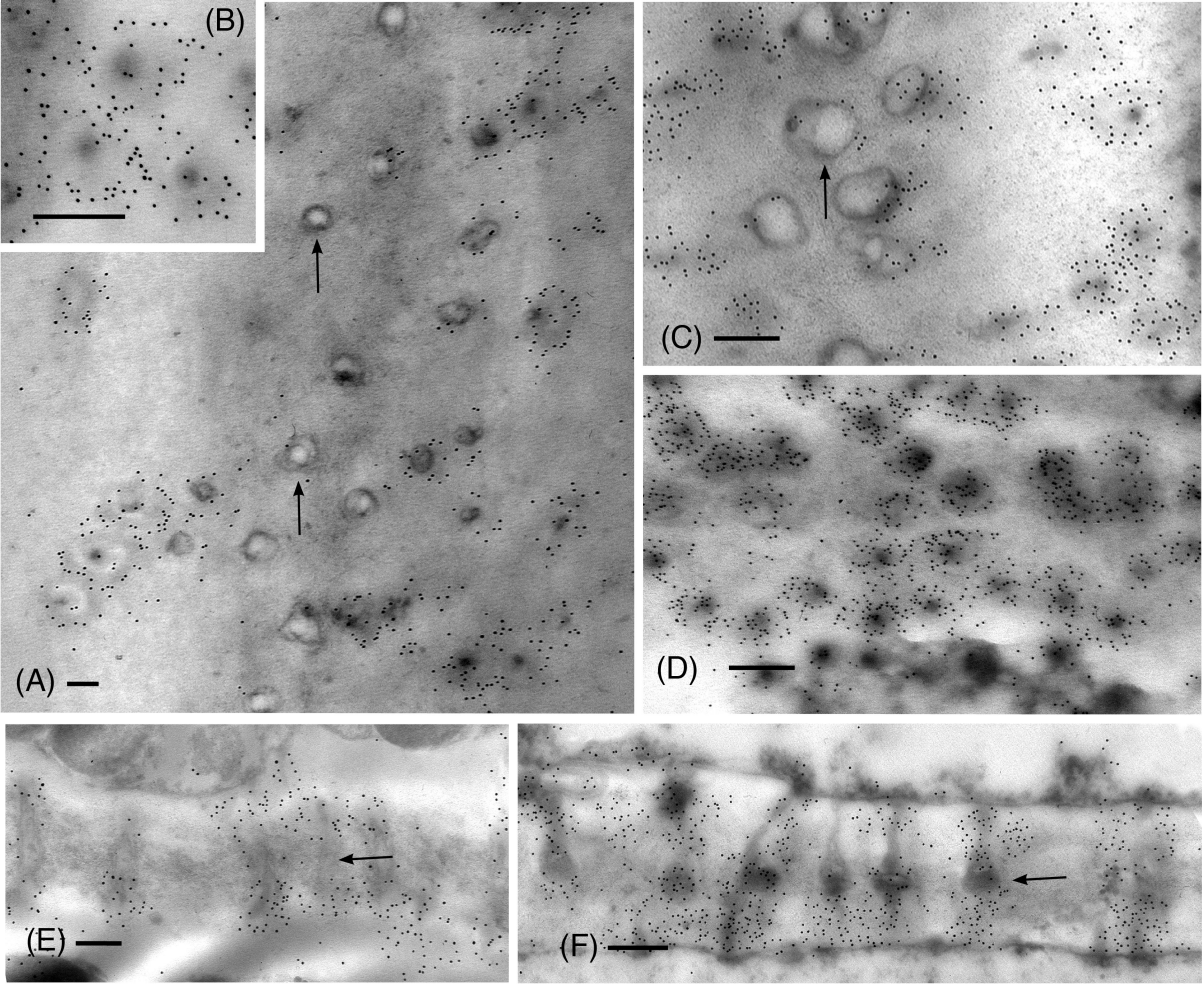
TEM micrographs showing labeling for callose in mature leptoid end walls of hydrated **(A, B, E)** and 14-day dried **(C, D, F)** plants. **(A)** Cross section of PDs in hydrated plants showing rings of callose in neck region and enlarged pocket with little to no callose (arrows) in the zone between cells. **(B)** Enlargement showing cross section of neck region of PDs in hydrated plants. **(C)** In dried plants, little to no labeling is found in the central pocket (arrow) and abundant callose surrounds the neck regions of PDs to the right. **(D)** Label density for callose around PDs in cross section of this dried plant is visibly greater than that in hydrated plants [cf., (**A, B**)]. **(E)** Longitudinal sections of PDs surrounded by callose in hydrated plants. **(F)** Longitudinal sections of PDs surrounded by abundant callose in dried plants. The central zone (middle lamellar region) between adjacent leptoids (arrow) forms a pocket with little to no callose. [Bar: = 0.25 μm].

Micrographic data demonstrates the widespread occurrence of callose in the apical region, and differentiating and mature leptoids in both hydrated and dried plants of *P. commune* (**Figures 1**−**4**). Aniline blue staining reveals the location of callose throughout development (**Figure 1**). In contrast to leptoids, no fluorescence is visible in hydroids, indicating the absence of callose in water conducting cells (**Figures 1C, F**). Aniline blue fluorescence is diffuse and similar between hydrated and 14-day dried plants at the apical region (**Figures 1A, B**). As cells elongate and differentiate, extensive regions of plasmodesmata and associated callose are visible in cell wall and end walls of leptoids in both hydrated (**Figure 1C**) and dried plants (**Figures 1D, E**). Mature leptoids are elongated with oblique to transverse end walls (**Figures 1F, G**) that are particularly prominent in dried plants (**Figure 1G**). Transverse appearing end walls are likely due to the plane of section of oblique walls. The middle lamella region between end walls of adjacent cells visibly lacks callose in aniline blue images (**Figures 1F, G**).

Anti-callose labeling in the TEM reveals details of callose localization and abundance during development and following drying in *P. commune* (**Figures 2**–**4**). In hydrated plants, plasmodesmata are scattered throughout newly formed primary walls and callose is loosely distributed across the walls (**Figures 2A, B**), corresponding to the aniline blue fluorescence (**Figure 1A**). Primary wall thickening in developing leptoid end walls corresponds to increase in length of plasmodesmata (**Figures 2C–E**), and a sparce association of callose along plasmodesmata (**Figure 2D**), followed by an aggregation of callose along plasmodesmata in leptoid end walls (**Figures 2C, E**). Callose localizes at the neck region of plasmodesmata in maturing leptoids and is sparce to absent in the zone between cells (**Figure 2E**). Similar plasmodesmata with callose labeling are visible in lateral walls (**Figure 2F**).

As in hydrated plants, plasmodesmata in leptoids of dried plants are abundant across the entire end wall and in aggregates along lateral walls (**Figure 3A**). Callose labeling appears more abundant throughout development in leptoid cell walls of dried plants (**Figures 3B–E**) than hydrated plants (**Figure 2**). In meristematic cells, callose is associate with plasmodesmata and is scattered along newly formed primary walls (**Figure 3B**). With differentiation of leptoids and cell wall thickening, callose abundantly labels enlarged plasmodesmata (**Figures 3C–E**). Less labeling is found in the zone between cells and is visible in cross sections of plasmodesmata (**Figure 3E**). Dense granular plugs cap maturing plasmodesmata (**Figures 3D, E**).

From the apical region to mature leptoids, the diameter of plasmodesmata is similar between hydrated and dehydrated plants. Diameters range from 0.03 to 0.06 μm in newly formed cells and expand up to 0.1 μm in diameter in mature cells in both treatments. Mature leptoids have abundant elongated and enlarge plasmodesmata in end walls and scattered aggregates of similar plasmodesmata in lateral walls (**Figure 4**). Callose labeling encircles plasmodesmata in food conducting cells of both hydrated (**Figures 4A, B**) and dried plants (**Figure 4C**). A middle expanded pocket, which measures up to 0.2 μm in diameter, characterizes leptoid plasmodesmata and this region lacks callose compared with an abundance on either side (**Figures 4A, C–E**), similar to those in aniline blue fluorescence (**Figures 1F, G**). The greater abundance of callose in leptoids of dried plants (**Figure 4E**) is evident compared with hydrated plants (**Figure 4D**). Label density analysis for anti-callose shows a significant difference between hydrated and dehydrated plants in all developmental regions (**Table 1**). Occlusion of plasmodesmata with a dense granular plug, presumably p-protein, in leptoids is widespread in dried plant (**Figure 4E**) and less so in hydrated plants (**Figure 4D**).

**Table 1:** Density of anti-callose immuno-gold labels in leptoid cell walls across three developmental stages (meristem, subapical and mature) in stems of hydrated versus stems that were dehydrated in the field for 14 days.

Sample Stem Region	Hydrated		Dehydrated		P-value
	Total counts	Average	Total counts	Average	
Meristem	260	3.09	388	4.61	0.00624
Subapical	375	4.46	490	5.83	0.00167
Mature	320	3.80	511	6.08	0.00035

Total label counts, average number of labels per counting frame, and significant t-test P-values (P<0.05) are noted for each.

### 3.1 Bioinformatics and callose synthase evolution

**Figure 5:**
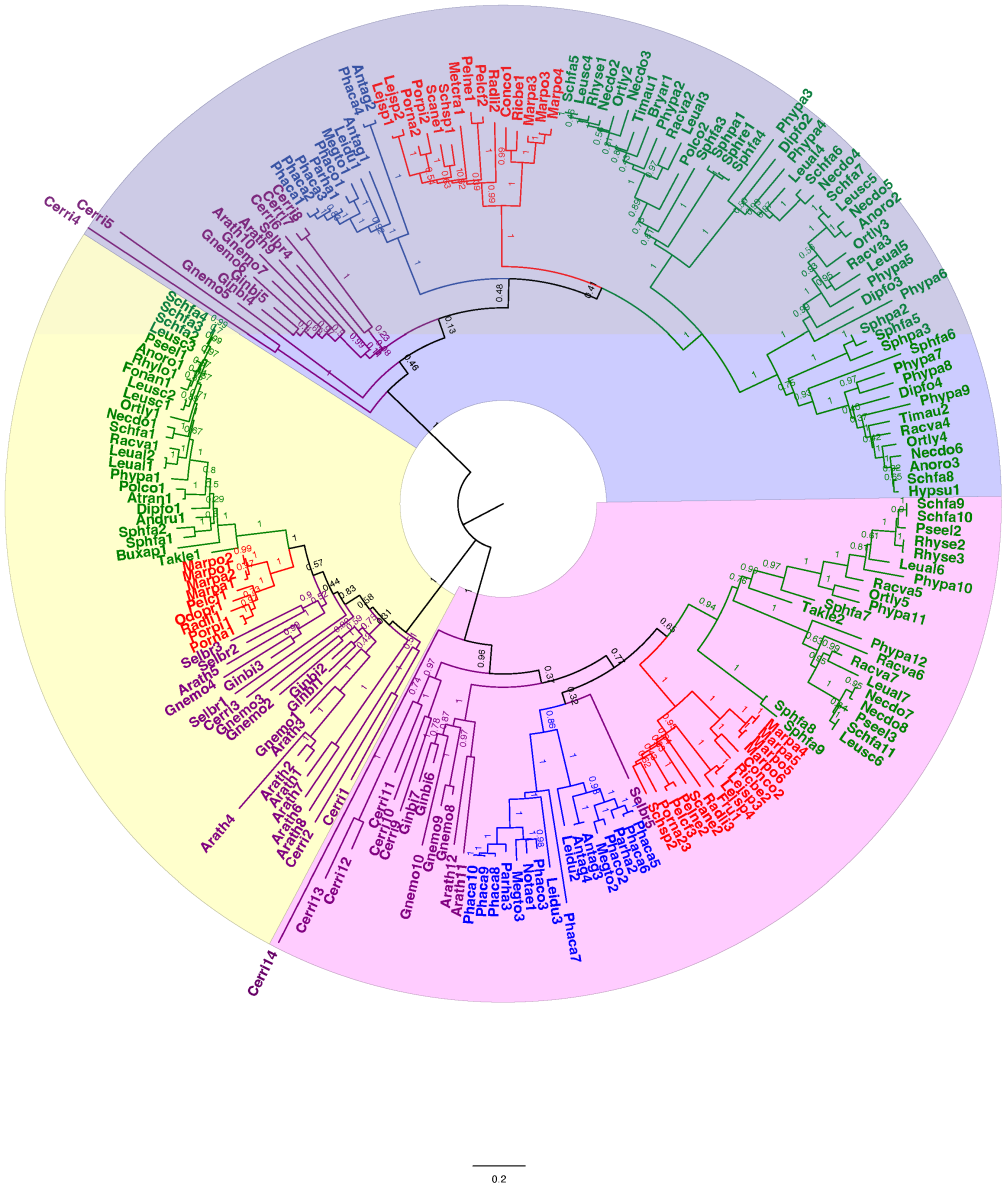
Unrooted phylogenetic tree of amino acid sequences of callose synthase proteins. The evolutionary history was inferred using maximum likelihood. Bootstrap support values are shown on the branches above. The ML log likelihood -175442.05. The analysis included 209 amino acid sequences and 1415 positions in the final dataset. Taxa are colored coded with green for mosses, blue for hornworts, red for liverworts, and purple for tracheophytes. Scale bar represents 0.2 expected substitutions per site. The amino acid sequences along with the accession number, and species and length of the CalS protein of the species can be found in **Supplementary Files S2**, **S3**.

**Figure 6:**
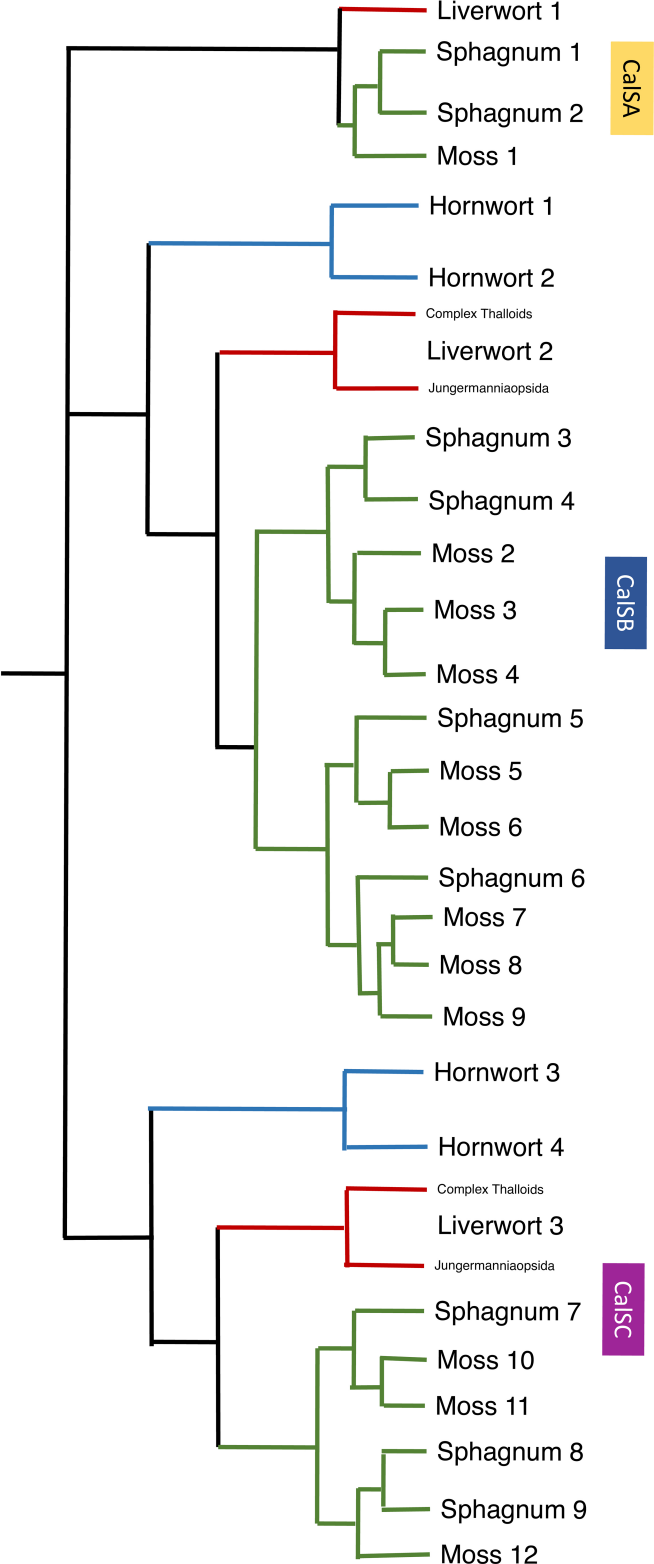
A summary of callose synthase (CalS) evolution in bryophytes based on **Figure 5** identifies three clades (CalSA, CalSB, and CalSC) with multiple duplication events in true mosses, *Sphagnum* and two in hornworts. The CalS A clade has been lost in hornworts but retained in liverwort and mosses with one event of duplication in Sphagnum but none in true mosses or liverworts. Branches are colored green for mosses, blue for hornworts, and red for liverwort.

A total of 209 protein sequences were used to generate a callose synthase (CalS) tree. Of the 209 sequences, 159 were bryophyte sequences downloaded from 1000 Kp database, including 27 hornwort, 39 liverwort, and 93 moss CalS-like proteins. The CalS protein phylogeny displays three main clades, CalSA (yellow), CalSB (lavender) and CalSC (pink) (**Figure 5**). A summary tree is presented in **Figure 6**. In *A. thaliana*, CalSA contains CalS1-CalS8 from, CalSB contains CalS9-CalS10, and CalSC contains CalS11-CalS12. The CalSA group is absent in hornworts while CalSB and CalSC are found in mosses, liverworts and tracheophytes. Liverworts have a single CalS paralog in all three clades with a clear divergence of amino acid sequences between the complex thalloid and jungermanniopsid (leafy and simple thalloid) groups in clades B and C (**Figure 6**). Hornworts have two paralogs in clades B and C, with *Phaeoceros carolinianus* containing three paralogs of CalSC. In comparison, mosses have multiple paralogs in groups CalSB and CalSC. Our study identifies two candidate CalS partial sequences in *Polytrichum commune*, with one copy in CalSA and another in CalSB (**Figure 5**). The CalSB clade has multiple paralogs in true mosses (eight in *P. patens)*. In the CalSC clade, true mosses have three paralogs. Our phylogenetic tree shows a divergence in CalS proteins from *Sphagnum* versus other mosses, which is evident in all three CalS clades. Unlike *P. patens*, the genome of *S. fallax* has only nine copies of CalS proteins, two in CalSA, four in CalSB and three in CalSC.

## 4 Discussion

Highly differentiated food-conducting cells in land plants include moss leptoids, pteridophyte and gymnosperm sieve cells, and sieve tube elements of angiosperms. These cell types share structural and developmental similarities that include a modified cytoplasm, differentially thickened cell walls and oblique overlapping end walls with modified primary plasmodesmata that form pores of variable size (Kalmbach and Helariutta, 2019). Such cellular commonalities may be viewed as a prerequisite for an efficient symplastic transport of photoassimilates. The abundance of callose in the pores of *Polytrichum* leptoids documented in this study, provides support for the importance of this polymer in the development and functioning of food-conducting cells across diverse plant groups. Further, the increase in callose following water stress demonstrates its role in stress responses as has been shown in other plant groups (McNairn, 1972; Nedukha, 2015; Cui and Lee, 2016).

Across land plants, there are notable differences in the structure of food-conducting cells. Unique and defining features of leptoids and food-conducting parenchyma of mosses are that they are nucleated (in some degeneration of nuclei is evident), exhibit cytoplasmic polarity, and contain a system of axial microtubules associated with the nuclear envelope and traversed by organelles (Ligrone and Duckett, 1994; Ligrone et al., 2000; Pressel et al., 2006). The pores in cell walls are expanded plasmodesmata, with membranous inclusions, and are the same size in the lateral and oblique end walls as is mirrored in the pores of pteridophytes and gymnosperms (Evert and Alfieri, 1965; Kalmbach and Helariutta, 2019). Sieve tube elements of angiosperms undergo more pronounced cellular differentiation, including enucleation, selective autolysis, and the formation of sieve plates with highly expanded pores, which greatly sets them apart from leptoids and sieve cells (Kalmbach and Helariutta, 2019). The essential and specialized association with nucleated companion cells is not found in other plants. Specialized cell wall ingrowths (containing callose) are found in companion cells but have not been reported in other parenchyma cells associated with food-conducting cells (Dahiya and Brewin, 2000). Albuminous cells in gymnosperm phloem share some similarities with companion cells however they differ in ontogeny and not all sieve cells have associated albuminous cells (Esau and Thorsch, 1985). In pteridophytes, parenchyma cells are associated with sieve cells but the nature of the association is unclear (Evert et al., 1989). Similarly, the relationship of leptoids with parenchyma containing abundant starch as shown in this study in the leptome of *Polytrichum* has yet to be examined.

The highly evolved end wall sieve plates in angiosperm sieve tube elements contain enlarged pores of variable sizes, from 0.9 to over 14 μm in diameters (Liesche et al., 2015; Kalmbach and Helariutta, 2019). Pore development in sieve plates requires precisely-timed and spatially-controlled cell wall remodeling that involves callose. Sieve plate differentiation starts during cytokinesis with the synchronized production of plasmodesmata along adjacent end walls. Callose deposition first occurs around the edges of the plasmodesmata and through progressive degradation of cellulose and replacement with callose, an extensive collar of callose forms (Kalmbach and Helariutta, 2019). This so-called callose plug serves as a scaffolding that maintains the cell wall integrity until the pore is fully formed, a process that involves breakdown of the desmotubule and the traversing endoplasmic reticulum that characterize plasmodesmata. Callose is rapidly degraded beginning at the middle lamella until an enlarged pore forms with residual callose remaining only around the pore opening (Esau and Thorsch, 1985).

It is fair to posit that the deposition and degradation of callose, which occurs in angiosperms, was essential for the evolution of the greatly enlarged sieve pores. However, not all pores in food-conducting cells contain callose. Kalmbach and Helariutta (2019) report that, similar to *Arabidopsis* Cals7 mutants, conifers, cycads, some ferns and some mosses lack callose in their sieve cell end walls and only produce callose upon wounding (Neuberger and Evert, 1975; Perry and Evert, 1975; Scheirer, 1978). Among the plants that have been demonstrated to contain callose associated with end walls, pores diameters are highly variable with a trend toward increased diameters during plant diversification. Notably, some ferns, gymnosperms and polytrichaceous mosses align more closely in pore size with each other than any of these groups do with the larger pore sieve plates found in many angiosperms. Pore diameters in leptoids of *Polytrichum commune* and *P. formosum* (Pressel et al., 2006) reach diameters between 0.1-0.2 μm and contain persistent callose. In sieve cells of the leptosporangiate ferns *Phlebodium* and *Platycerium* (Evert et al., 1989) callose is present in pores that range from 0.12 to 0.38 μm. According to (Liesche et al., 2015), pore sizes in gymnosperm sieve cells range from 0.335 μm (*Picea abies*) to 0.61 μm (*Gnetum gnemon*).

The minimal expansion undergone by the pores in *Polytrichum* involves callose deposition with no degradation, sharply differentiating them from angiosperm sieve pores. Callose abundance increases from newly formed plasmodesmata in the shoot apex to fully functioning leptoids, although it is less abundant in the zone between cells. Mature leptoids in this moss are expanded near the middle lamella producing median cavities of varying sizes as demonstrated herein and in previous studies (Stevenson, 1974; Scheirer, 1978; Scheirer, 1990; Ligrone and Duckett, 1994).

Unlike phloem that is ubiquitous in tracheophytes, food-conducting cells, including leptoids, are not found in all mosses, suggesting this type of cellular differentiation is not critical to the translocation of photoassimilate. Takakiales and Andreaeales, the sister groups to the remaining mosses, do not possess food-conducting cells, suggesting the acquisition of such tissue occurred during moss diversification. Indeed, only true mosses exhibit histologically distinct food-conducting cells with significant elaboration in leptoids in the Polytrichales. Moreover, if present, food-conducting cells in true mosses are variously located in both generations, in the sporophyte only, or in the gametophyte only (Hébant, 1976). This sporadic occurrence is reminiscent of stomata in mosses where their scattered occurrence suggests they are not critical to moss survival (Renzaglia et al., 2020b). Food-conducting cells are absent in hornworts and elusive in liverworts (Ligrone et al., 2012), highlighting the diverse transport mechanisms among mosses and across bryophytes. The lesser stature and less intense demand for translocation of photosynthate in mosses are undoubtedly related to the apparent simplicity of architecture and the minimal elaboration of pores in end wall of moss leptoids compared with tracheophytes.

We have shown that dehydration in *P. commune* enhances callose production in developing and mature leptoids, suggesting this polymer plays a protective role against desiccation similar to sieve elements (Rinne and Schoot, 1998; Rinne et al., 2011). Due to its high water-holding capacity, callose can add to the plasticity in cell walls during the stress associated with cycles of desiccation and rehydration (Herburger and Holzinger, 2015). This follows the current understanding that desiccation tolerance in bryophytes is by means of ‘constitutive’ protective substances (Proctor et al., 2007). Other abiotic stresses, including mechanical stress and toxic metal exposure, have been correlated with increased callose deposition in the moss cell wall (Scherp et al., 2001; Tang, 2007; Krzesłowska et al., 2010; Krzesłowska, 2011). For example, callose thickens cell walls and protects the protoplasm of moss protonemata in response to environmental toxins such as lead (Krzesłowska and Woźny, 2000). Across plants, the level of callose deposition in plasmodesmata is highly regulated and varies in response to diverse stresses (Wu et al., 2018). Callose deposition in the neck region of plasmodesmata controls permeability by restricting molecules from moving through cells (Radford et al., 1998). The role in bryophyte stress responses is likely similar but requires further experimentation.

Although our understanding of the extent of occurrence and role of callose across bryophyte taxa and structures is incomplete, it is clear that this cell wall polymer is fundamental to the development and differentiation of bryophyte cells and tissues as in other land plants (**Table 2**). Callose plays an essential role in cytokinesis and the development of shoots, and has been localized in protonemata, rhizoids, and paraphyses (Abel et al., 1989; Scherp et al., 2001; Scherp et al., 2002; Scherp et al., 2004; Berry et al., 2016; Muller et al., 2022). Callose is also involved in sporogenesis (Schuette et al., 2009; Renzaglia et al., 2015; Renzaglia et al., 2020a) and spermatogenesis (Gorska-Brylass, 1969; Renzaglia and Garbary, 2001).

**Table 2:** Occurrence of callose in bryophytes.

	Moss	Liverwort	Hornwort	References
**Rhizoids**	+	+	?	Cao et al., 2014; Dehors et al., 2019
**Cell Plate**	+	+	+	Scherp et al., 2001; Scherp et al., 2002; Scherp et al., 2004; Ušák et al., 2023
**Plasmodesmata**	+	+	+	Muller et al., 2022
**Food conducting cells**	+	?	NA	Present study
**Protonemata/sporeling**	+	?	?	Krzesłowska and Woźny, 2000; Dehors et al., 2019
**Spermatogenesis**	–	+	+	Gorska-Brylass, 1969; Lopez-Swalls, 2016
**Water conducting cells**	–	+	NA	Ligrone et al., 2002
**Cortical parenchyma**	+	+	+	Ligrone et al., 2002
**Sporogenesis/spores**	+(aperture)	+	+ (intine)	Brown and Lemmon, 1987; Schuette et al., 2009; Renzaglia et al., 2015; Renzaglia et al., 2020a
**Placenta**	–	–	+ (limited cells)	Henry et al., 2020; Henry and Renzaglia, 2021; Henry et al., 2021
**Elaters**	NA	–	–	Kremer et al., 2004
**Guard cells**	–	NA	–	Merced and Renzaglia, 2016

+, detected; -, not detected; ?, not examined; NA, structure absent.

The evolution of callose synthases (CalS) is well-characterized in tracheophytes but is lesser known in bryophytes (Schuette et al., 2009; Záveská Drábková and Honys, 2017; Li et al., 2023). In *Arabidopsis thaliana* there are 12 members of the CalS gene clade that catalyze the synthesis of callose at different stages of development, in different tissues and in response to different environmental stimuli. Our phylogenetic analysis of protein sequences targets diverse representation across bryophytes and reveals three clades of callose synthase-like genes (CalSA, B and C) across land plants as previously reported (Li et al., 2023). This callose synthase phylogeny largely tracks the phylogenetic signal across bryophytes (Bechteler et al., 2023) with high bootstrap support for moss, liverwort and hornwort clades (**Figure 5**). There are 12 members of the CalS gene clade in the true moss *Physcomitrium patens*, three in liverworts with no duplications and four in hornworts including two duplications (**Figure 6**). In *Arabidopsis*, extensive diversification with numerous duplications occurred in the CalSA clade, which has CalS1-8 genes known to have diverse physiological functions (Záveská Drábková and Honys, 2017). In contrast, there are only four bryophyte genes in this clade: one true moss, two *Sphagnum*, one liverwort and no hornwort. The lack of CalSA in hornworts, suggests a loss early in land plant diversification and the role of other genes than CalS1-8 in fundamental cellular processes that utilize callose and are universal in plants such as in cell plate formation and plasmodesmata function. Because true mosses have only a single CalSA gene, the wide range of functions of callose synthase genes identified for angiosperms in clade A is also unlikely in mosses. CalS3 or CalS5 are the likely candidate ancestral genes found in mosses and liverworts and lost in hornworts. CalS5 was speculated to be involved in sporogenesis in *P. patens*, but this has not been assessed experimentally (Schuette et al., 2009). Because CalS7 is essential to sieve plate formation in angiosperms (Barratt et al., 2011) and does not occur in bryophytes, a different CalS gene must be involved in the development of the highly modified end walls in leptoids of *Polytrichum* that we have demonstrated to contain an abundance of callose. This finding is consistent with the lack of food conducting tissue in *Takakia* and Andreaeales, sister taxa to other mosses, and the independent origin of leptoids among mosses in the Polytrichales.

Gene duplication in mosses occurred in both the CalSB and C clades, with the vast majority of diversification occurring in CalSB (**Figure 6**). With eight duplication events in *P. patens* CalSB genes, it is likely that these genes are responsible for many important physiological roles of callose in mosses. For example, in angiosperms, CalS10 in clade B is known to function in cell plate formation during mitosis and around the plasmodesmata (Hong et al., 2001; Chen and Kim, 2009; Saatian et al., 2018). Similar functions of this gene or those derived from it in the CalSB clade are likely to be carried out in mosses and perhaps other bryophytes. The CalS protein sequences in *Sphagnum*, a sister taxon to true mosses, reflect a divergent evolution within mosses. Unlike true mosses, the genome of *S. fallax* has nine homologs, not 12, and there are three total duplications not eight, with one in each of the three clades (**Figure 6**). *Sphagnum* possesses a vast array of morphological differences from other mosses, including conducting parenchyma cells that are much less specialized than leptoids of polytrichaceous mosses (Ligrone et al., 2000). The occurrence and location of callose has yet to be reported in the Sphagnales and whether this polymer is associated with food-conducting cells in these mosses remains to be examined.

An interesting finding in this analysis is that there are no apparent duplications of callose synthase genes in liverworts but there is a primary dichotomy in the gene clade that separates those in the complex thalloid from the remaining liverworts in CalSB and C (**Figures 5**, **6**). To date, the involvement of callose in liverworts is best known from sporogenesis (Renzaglia et al., 2015) and spermatogenesis (Renzaglia and Garbary, 2001), where this polymer is highly abundant and critical to spore wall development and separation of male gametes during differentiation. The only study that focuses on callose in hornworts is that on spore wall development and the unique occurrence of this cell wall polymer in the spore intine (Renzaglia et al., 2020a). Clearly, our knowledge of the location and function of callose across bryophytes is in its infancy, and further morphological studies that examine callose across bryophytes are warranted. With the availability of more well-characterized genome sequences (Carpenter et al., 2019) and better transformation protocols for the development of targeted mutants (Waller et al., 2023), both the roles and evolution of this critical cell wall polymer in bryophytes will be clarified.

## Data availability statement

The original contributions presented in the study are included in the article/**Supplementary Material**. Further inquiries can be directed to the corresponding author.

## Author contributions

KR: Conceptualization, Formal analysis, Funding acquisition, Investigation, Methodology, Project administration, Resources, Supervision, Validation, Visualization, Writing – original draft, Writing – review & editing. ED: Data curation, Formal analysis, Investigation, Methodology, Validation, Writing – review & editing. LS: Conceptualization, Data curation, Formal analysis, Funding acquisition, Investigation, Methodology, Validation, Visualization, Writing – review & editing. JH: Formal analysis, Investigation, Methodology, Supervision, Validation, Visualization, Writing – original draft, Writing – review & editing.
