# Supplementary material for: Callose in leptoid cell walls of the moss Polytrichum and the evolution of callose synthase across bryophytes
Source: Front Plant Sci. 2024 Feb 7;15:1357324. doi: 10.3389/fpls.2024.1357324 (PMC10879339; doi:10.3389/fpls.2024.1357324)
Supplement: Supplementary file 1 [file DataSheet_1.pdf]

>CMEQ 2006259 Orthotrichum lyellii 1566

SAHSFLDKIVQPIHEILAAEAKICAGGRAPHSAWRNYDDFNEFFWAPSCFELSWPWRLDAGFFRKPKKIIYTEADRFEPV
APEVSPVVGQTREKKVGKTHFVEHRTGFHIYHSFHRLWIFLVCMLQGLGIFAFCDRKLNVHSVKLIMSVGPTFVLMKLIQ
SVMDVTLMIGAYRSTRTQNISRMLIRFLWFAILSTVIVLLYVKTIEEENSGSGSDIWFRAFYLVLGIYGGLQFFFALLLRVPW
FRLQAEKCSNFYVVMFIKWVHQERYYVGRNMFERTRDYFQYTLFWFVVGTCKFAFSYFLQIQPMVGPTRTIIGIRNVTYS
WKDLISGSNHNALTLVALWAPVVMVYFLDTQVWYTVVSSLVGGFDGAKMHLGEIRSLDMFRSRFSSLPGAFVKNLVPS
KSGGHSQTDVDVHLSAVKPGNPKVDASRFSPLWNDVINSLREEDLINNREREWLLMPDNITRSGALGEHSLVQWPLFLL
ANKVYVGIDIVHDNRQSFQDEIWDRMKRDPYLEYAVMEAFISLQSVLIDLLNEHGRTWVIDIHTDIDQAIEVGQLFYKFN
FGNIGNVLSMIAKLTEVLVSEPKKADDDSGRQEEELKLHESAARALVDLYEVVMRDFLADAELREKYDSDTLLQASKLDG
RLFSDLTWPAGPAKVQVKRLHYILAIKDSALNVPVNLEARRRLQFFSNSLFMSMPQPPPVRKMFSFSVFTPYYSQDVMY
SKAKLEDTNVDGITILYYLQTIVPDEWKNFLERMFPGVEYNQLGHYTEADVTDTLQLRLWASYRGQTLARTVRGMMYY
KRALILQAQQEGASMAEDEEEGHDLEGNELAIVNASTPRTPRGSLVRSARAQAELKFCYVVSAQIYGKQKNSFLQADKD
RAADILYLMHMNDSLRIAYIHEAKQIVDGKPFTEYYSKLVKADPSGKDQEIYSIKLPGEVILGEGKSENQNHAIVFTRGEAL
QTIDMNQEHYLEETLKVRNLLEEFDSKNLGLRRPTILGVREHVFTGSVSSLAWFMSLQERSFVTLGQRVLANPLKVRMH
YGHPDVFDRIFHITRGGISKPSKQINLSENIFAGFNSTLRQGNITHHEYIQCGKGRDVGLNQIAAFEGRVASANGEQSLSR
DIYRLGQLFDFFRMCSFFFTSVGYYFTTMLTVLTVYVFLYGKVYLALSGVDEALKASNLLENTALQAALDTQFLLQIGFFTAV
PMIVNFILEQGVLRAAISFLTMQLQLSSVFFTFSLGTRSHYFGRTILHGGAKYKATGRGFVVEHIPFAENYRAYARSHFVKG
MEIIFLLIVYLVYGAHDRTTASYILLTFSSWFLALSWLYAPYIFNPSGFEWQKTVVDFDDWTNWLFHKGGIGDEGKKSWE
VWWDEEQAHIQTPRGRFWEILLSTRFFIFQYGVVYALNASGNDKSFWVYGYSWVVLVAVFLLFKIFTFSQKASANFQLIV
RLLQGVIFLAVVAGVSVAVVLTRLTIGDVFASALAIIPTGWGLLSIAIAIRPVVQWFGLWKSVRGVARLYDAAMGMILFIPI
AFLSWFPFVSTFQTRIVFNQAFSRGLEISVLLAGNNPNATI------------------------------------------------------------------------
----------------------------------------------------------------------------------------------------------------------------------------------
----------------------------------------------------------------------------------------------------------------------------------------------
------------------------------------------------------------------------------------------------

>CMEQ 2007829 Orthotrichum lyellii 1758

VFNILPVDDPSIDHAGMSFPEVRAAITSLKGMRNLPRLSGVAWSPDMDMLDWLGHLFGFQTDNVRNQREHLVLLLAN
GMMQLYHAGPTYETLEASIVRKTRKKVTSNYVSWCRFIGCKNHLKYLEANPEHFDEGRELIYICLNLLIWGEAANLRFMP
ECLCFIYHHMLADLNRLLDRYEVDFMIPDSEENGFLNRVVVPLYGILKAEADSNNGGTAPHSSWRNYDDLNEYFWSSRC
FKQLRWPLNMNSSFFVEPKREKGYLNRGRKPQSDKVGKTGFVEQRSFWYIFRSFDKLWIGYLLILQASVVILWHNGGPP
WIELQKPDPLARFLSIFISWALLRFLQGLLDVGSQYSLVTRDTVLVGVRMILKLLVAATWAVLFIIFYRKMWWQRNIDQY
WTDIAYSKMHEFLYIVAAFLAPEVLAIVLFIVPWVRNYVETSSWRIFHLLTWWFQTRGYVGRGLREGTLDNIKYTLFWAC
VLASKLSFSYWLQIRPLIAPTKQILRATNVVYEWHEFFPDGNRAAVVALWAPVLMIYFMDTQIWYSIWSSIIGALVGLLQH
LGEIRNVEQLRLRFEIFPSAFQFSLMPMDDAVRPNVWAGAKDLFKRLGLRYGWSSVYDKMGWADIKGGRFAYVWNTII
HTFREEDLISDREVELLEIPKGEWRLSVFQWPSTLLANQILLALYSVRYHRGDDASVWNIICKNEYRKCAVIECYESMKYLL
RRVLSDDSEEYVIFIAIFEEIDAAILQKRFTSTFLLPELMEIHARVVDLVSLLLTRPNQKQLQKVVKALQNLYERLVYDFPRDS
AVTENIKARTSLHKNNEMTQLFMDAVELPATDEEPFFKTLKRLHTTLSTKDALLDVPKGLEAKRRISFFSNSLFMTMPRAP
QVERMLAFSVLTPYYNEEVIFSKQQLKVENEDGVTILFYLQRIFPEDWHNFLERMKKRDLSEAQLWEKDDAIELRLWASY
RGQTLARTVRGMMYYERALEVQTFLDSASEEDLHGIPTALERGISTNSRGSFRSIGSMGSIGSAHDVAEFNRVHRREQEL
ANAAMKFTYVVTCQMYGAQKLAKDARAADILRLMKTYPALRVAYADERTITTGDTDYKQYYSVLIKYDPILDKEVEIYRIQ
LPGPLKLGEGKPENQNHALIFTRGDAVQTIDMNQEMYFEEAIKMRNLLQEFTVRHGSRKPTILGVREHVFTGSVSSLAW
FMSAQETVFVTLSQRVLANPLKIRMHYGHPDVFDRLWFLTRGGISKASRTINISEDIFAGFNCTLRGGNVTHHEYIQAGK

GRDVGLNQIAMFEAKVASGNGEQILSRDVYRLGHHLDFFRMLSFYYTTVGFFVSNMMVVLTVYTFLWGRAYLALSGIER
SLTTGALTNSALTATLNQQLVVQLGLLTALPMLVEDALEHGFTTALWNMITMQLQLASIFFTFSMGTRCHYFGRTLLHGG
AKYRGTGRGFVVKHEKFAENYRLYSRSHFVKGIELLLLLFCYLAYGATNSTGTYVLVSISSWFLALTWIMAPFVFNPSGFDW
LLTVEDFSDFMQWLWFKGDVFVRVEQSWEIWWEEEQAHLRTTGLWGKLLELILDLRFFIFQYGIVYHLSITGNNTNVFIY
LASWSYMLFAAILHFVLSHANERYAASQHGLYRLIQVLVIAVIVAVVVVLWVETNFKFIDIIASFLAFLPTGWGIIQISLVLRR
PFLENSPLWGTIVAVARLYDLGMGLIVMAPVAILSWLPGFQAMQTRILYNEAFSRGLQISRLLAGKKNRNID--------------
-----------------------------------------------------------------------------------------------------------------------------------
----------------------------------------------------------------------------------------------

>CMEQ 2011556 Orthotrichum lyellii 1996

MAADGRLHTGATYLPRHVSRTYAAGGLTEVFDSEVVPSSLASIAPILRVANEIEASSPRVAYLCRYHAFEKAHRIDSTSSGR
GVRQFKTALLQRLERDSEPTLALRHRRSDAREIQSYYQNYYNDYVKALDGAEHSDRAQLARAYQTAAVLFEVLKAVNRDK
AEEPPPEIIAAAADVEQKKEIYVPYNVLPLDAAGASQAIMQLDEVRAAVEALRNVRGLPWQTVKESHSRAGDLDCLDW
LQDMFGFQKDNVANQREHLILMLANVHIRLAPRPEPMHKLDDRALTAVMNKLFKNYKSWCKFLGRKNKIWLPQIHQE
VRQRRILYMGLYLLVWGEAANLRFMPECLCYIYHHMASELHGMLAGNVSMVTGDNMKPVYGGEDESFLRLVITPIYNV
ISGETFKNRNGTAPHAAWRNYDDLNEYFWRVDCFRLGWPMRPDADFFVPAQSSTTNDVHGRNYRLSSKSFFVEIRTF
WHVFRSFDRLWAFYILGLQAMIVLAWNVGPNLRNAFSGAVIKQVLSIFITASILRLIQAFLDIVFGYHAFRSIKLLGVLRLVL
KLITSAAWVVVLTICYVRTWVNPHGLIGEIQKWLGRSLESSYLYVSAVLVYLIPNLIGAFFFLFPMIRRWIESSNWTVVRFLL
WWSQPRLYIGRGMHESQVSLFGYTFFWVLLIASKFAFSYFFQIEPLVAPTKAIMEQTSVAYTWHEFFPNARNNPGALISL
WAPIIMVYFMDSQIWYAVFSTIFGGISGSFYRLGEIRTLGMLRSRFSSLPGAFNESLVPDDRKRARKGFSFSREFEKIAPSK
DRLKAVRFSQLWNEVITSFRQEDLISDKERDLMLVPYSSDTHLKLVQWPPFLLASKVPIALQMAKQAAETGRTADLSRKL
KIDEYMKCAVTECYESLKRVLKILIVGEVETRVIDGLFDEVDVNLEKETLLDNFKLKDLPVLSVKFIELLELLEKYNAGHEAID
SARDLAVLKLQDMYEVITRDMMSDSMRDSWDSSLGALAGGQGRKSELFSSKGDEPAKVVFPQSHSDAWIEQINRLHL
LLTERESAMDVPENLEARRRIAFFTNSLFMSMPQAPKVRNMLSFSVLTPYYKEDVVYSKENLMKENEDGISVLFYLQKIYP
DEWSNFLQRIGLGTSEDPEAQIFGISELEDKLREWASFRGQTLSRTVRGMMYYRRALELQAFLDIATEEELVDGYKVLTDA
TPDQKKSQRSTWSQLQAIADMKFTYVAACQMYGEQKRQGHHSATEILKLMLKNVSLRVAYIDEVEERQNEKPSKVYYS
VLVKAINGLDQEIYRIKLPGTVRLGEGKPENQNHAVIFTRGEGLQTIDMNQDNYLEEAFKMRNLLQEFHEPHGVRPPTIL
GVREHIFTGSVSSLAWFMSNQETSFVTIGQRVLASPLKVRFHYGHPDVFDRLFHITRGGMSKASQVINLSEDIFAGFNSIL
RRGNVTHHEYIQVGKGRDVGLNQISLFEAKIACGNGEQALSRDVYRLGHRFDFFRMLSCYFTTVGYYFSTMVVVLTVYV
FLYGRIYLALSGVDDSLVHSANNKALNAALASQILVQLGLLMALPMVMEIGLERGFRTALSDFLTMQLQLASVFFTFSLGT
KTHYFGRTILHGGAKYRATGRGFVVRHERFADNYRLYSRSHFTKAIELFLLLIVYTLYVSKSAKGAVTYILITFSMWFLVASW
LFAPFLFNPSGFEWQKIVEDWVDWNKWMSNRGGIGVEGNKSWESWWEEEQEHLNYSGYFGRFVESILSIRFLLYQYGI
VYHLNIARSSNDLSINVRPASLLSSLPLKLLTFISGVEVNNFEQLCKLFVSEFIDYYYTTHVVYFYHLNIARSSNDLSINVYGLS
WLVIVAVLTILKIVSMGREKFSADFQLMFRLLKASLFIGSISVIAILHVKNFTVGDLFASILAFIPTGWALLQISQACKPVVMK
LGFWNSVKALARGYEYLMGLLLFAPVAVLSWFPFVSEFQTRLLFNQAFSRGLQISRILGGRKKL---------

>CMEQ 2012235 Orthotrichum lyellii 1973

EEERTLHESQGNEDGDTPFVPQILHQQKKNIDSILQTARNVEGQYPQVARILYEYSYSLSQSVDPRSESRGVLQFKTALLDF
IKRKREEKSDRSEDVYIIIKFYKDLKRQLDQLENEDWLRQQPQYNQISPEEWAEVKRNIYVTVEILNELVDFLIKENPEMKR
HVEFDSDLKEEIEKTAKKLDDYKPYNILPFEAPGVVNPFQRSPEVVAAINTIELNSVDDHVFDSDFKRPHLRNLDIFDYLQY
TFGFQAHNVLNQREHLVLLLANSQSRLGSLRNNHPGSLDDKPKLDEHAIFNVRTKLLENYERWCDFLRQKKNTDRRLQ

DSSLMPQPRLLFSALYLLIWGEASNVRFLPECICYIFHHMAFEITDPQSEVFNHPFRKRSVILNGFKSFLDAIIKPVHEVLAA
EAKVCNHGKTPHSRWRNYDDFNEYFWAPFCFELSWPWRLDAGFFVKPKQDSNKKRNKANFRLTHAEEPLLQGQDQR
SQPGYRREKKSGKSHFVEHRSGLHLYHSFHRLWIFLVCMLQGLAVFAFCDGKLNATSIKYILSVGPTFVIMKFIQSVLDVIL
MVGAYRSTRARTLSRIWLRLLWFGSLSAIIVELYVKTIQEQESGSGQSTWFRLYCILLIFYGGSHFFVALLMNMPWLRRQA
EKCSNFGPVPFLNWIHQERYYVGRGLYESTSDYFTYILFWIIVLACKFSFSYFLQIRTMVGPTRTIVDLTDLNYRWRDVFSKS
NHNALTLVSLWAPVVMIYFLDLQVWYTVISALVGGLDGARMGLGQIRSLDMLRTRFTYLPEAFRRHRRDGEASSESDVV
QAPKVDDAIQFAPIWNDVITSLREEDLINNKEKEWLVMPGNSLTLLTASSQHLTLVQWPLFLLANKVFIACDMVEVHKQ
APQKELWERIERDPYMSYALQEAYYTLRIILELLLANDQGYRWVKNVYESMKESMRGGRLKDEFNLKRDQLVKVLDKAA
KLMNVLINEAKKLKDDEVEDHIENTREAYTAAVQKGLLDFYDVVMRDFIADSQTRIDADRGVGRDSERLFTNLAFPTEES
NSLVRRLNQILTIKESALNVPVNLEACRRLEFFSNSLFMKMPKAPPVRNMFSFSVFTPYYSEDVIYSPQQLAKENDDGISM
MYYLRTIVPDEWENFLERMYLKKEDREAKKALLKTVFPKEYRFKPNEKIRVPDDLNENIKLQLRLWASYRGQTLARTVRG
MMYYKRALVLQAQLEGASLSDDLEQGSQYSTSSVSQSSDILDARAQAELKFLYVVSAQIYGEQQQGAKGAEGRQKAADI
SYLMKTNDSLRISYIHKGKVKVEEGKEVTKYYSKLMKADPSGNDQEIYSIELPGEVILGEGKPENQNHAIVFTRGEALQTID
MNQEHYLEETFKMRNLLEEFDDAKSYGFRKPKILGVREHVFTGSVSSLAWFMSLQERSFVTLGQRVLAKPLKVRMHYG
HPDVFDRIFHITRGGISKASRQINLSEDIFAGFNSTLRQGNITHHEYIQCGKGRDVGLNQIAAFEGKVASGNGEQTLSRDIY
RLGQLFDFFRMLSFFFTTVGYYFTTMLTVLTVYVFLYGKVYLALSGVDADLKSKGLSANVALQSAVDTQFLLQIGVFTAVP
MIMNFVLEEGIWKAIISFFTMQLQLSSVFFTFSLGTRTHYFGRTILHGGAKYASTGRGFVVAHIPFAENYRMYSRSHFVKA
LEIMLLLIVYLAYGASERTTFTYILLTFSSWFLAISWLWAPFIFNPSGFEWQKTVMDFEDWTNWLFHKGGIGDEGKKSWE
VWWLDEQSHIQTLRGQFWEIILSLRFFLIQYGVVYALNVVGHDRNFRVYGFSWVVLAGVVLTFKVFSVNQKSSANFQLIL
RLFQMIVFLGIIGGAAVAVALTSLTIGDVFASALSLIPTGWGLLSIAIALRPVMKKLRLWKSVRAIARLYEAFMGTIVFIPIALL
SWFPFISTFQTRLVFNQAFSRGLEISSLLAGNNPNKDM-------------------------------

>CMEQ 2014675 Orthotrichum lyellii 1949

LMRSSYNRDGKVRGSSSGRGGQRAPQSLAQQADIDAVLLAADEIGQDNIQVARILAEHAYRLTQQLDPRSEGRGVLQF
KTGLKSIIKQKQQARQGDVVDRSQDIRIIQLYYKHFREYHDIDQLEEDARNRGPVLSTDLMPESQDQSFDKLRRVYEISK
MLNDVVDALLKEAEPGDASRLQDTDEKRVLEEDAKKLKGFKAYNILPLKTPLDTPTVLNPFDFFPEVVGATYVLGYTKDLP
RFPSDYEVPEDRVLDIFDFLHYAFGFQKDNVANQREHIVLLLASAQSRLGTLDQVRDSDANQTSLNPDKAISNVHDRILE
NYVRWCHFLRREPQNKRAFTQQRRLILTALYLLVWGEAANLRFMPECLCFIFHNLADECIDLLGRTFVERSKTVKANADG
SIEFSFLEQIITPVYDVVAAEAKSSQGGKVPHSNWRNYDDFNEYFWQPNCFVELGWPWREDAGFFKPPKMKGAKKVP
APITGANLPETAGRRRKHKVGKVHFVEHRTGFHIYHSFHRLWIFFICMLQGLTIWAFCSKNGNLNLHKIMSVGPTFVIMK
FIQSVFDIVFMWGAFKATRLQTVARMLLRFLWFACLSAAVLFLYIKTLEEDARNDGSGSWFRIFYILISSYAGANVIFVFLLR
IPFLQRQAAKCSNVYIFQFFKWLHQERYYVGRSMYERTRNYIKYSMFWIVILACKFAFTMHFQIMPLVDPTRLIIEFNNIK
YKWPDFVSDSNHNALTIVSLWAPVVMIYFLDTQVWYVVISALLGGIEGARDKLGEIRTLEMLRKRFPNYPAAYVKHMQP
PINRLLSPSPSHHGSSRLTKPKKLDAIRFQPIWNHVIKTLREEDLINNREKVLLKMPPNLMFHSNGAPNNLIHWPLFLLAN
KVHIAVELAVEHKTNQAGLWEKVSRDDYMAYAVQETFETLEPMLVSVLNPAGAQWVNEIFGRLKYSLGIADLRDNFNL
NRLRDVLEKLRDLTEHLGNEDVPERRVKATTAFFQLFDVVMRDFLSFRTRGEIDEAPGFREAVQSGLLFTDLNWPNKAG
QKQAKRLNNLLTVQKIKDQEGKTKTLNTETIPHNLEARRRLQFFTNSLFMHMPEAPPIRKMFSFCVFTPYYEEDVMYDM
EKLYQENEDGITILFYLQKIYPDEWQNFLERIGLIENIVTREIENRKSEKHEWVKLELRLWASYRGQTLARTVRGMMYYKA
ALIIQGMQEGASGGDLEEGIPPSLVEAQGSINRSAEAQAELKFTYVVTCQIYGEQKRKGKVQAADILYLMQKHESLRIAYI
DVVESSRKGTKPSYYSKLCKVDRSDPSLRDQEVYSIKLPGDIKLGEGKPENQNHAIVFTRGDCIQTIDMNQDNFMEEAFK
MRNLLEEFSKTHHGLHKPTILGVREHVFTGSVSSLAWFMSMQESSFVTLGQRVLARPLKVRMHYGHPDVFDRVFHISR
GGISKASRVINLSEDIFAGFNTTLRLGNVTHHEYIQVGKGRDVGLNQIALFEAKVASGNGEQTLSRDVYRLGQLLDFPRM
LSFFYTSVGFYVTTMMTVLTLYVFLYGKAYLALSGVDASLKRSSEILQNPSLESALNTQFLFQIGIFTAVPMIVNLILEQGILKA

IISFCTMQLQLASVFFTFSLGTRTHYFGRTILHGGAKYRSTGRGFVVTHIHFAENYRLYSRSHFTKALEIIMLLIIYLAYGAQN
RTSVTFILLTFSSWFLALSWLFAPYIFNPSGFEWQKTVEDFDDWTNWMFYKGGVGVKIENSWEAWWFDEQDHIRSLR
GRFWEVILSLRFFLFQYGVVYSLKVTKGSNSILAYVYSWFVLLGFLIIFKVFTVSQKASANFQLAVRLFQGLLFISLIAGLVVSI
VLSPLSVGDVFALALALIPTGWGLLSIAIAFRPLMERIGVWKSIREIARVYDACMGMIIFIPIAFLSWFPFISTFQTRLVFNQ
AFSRGLEISLILSGKQPNR------------------------------------------------------

>RCBT 2000508 Sphagnum palustre 1759

ETVDHHLKKQMENDAKKIREFKPFNILPLETPGVVNAFQMFPEVTAATGALEWSTPPFPEFPDGYEQPERALDVFDFLQ
YVFGFQEDNVANQREHLILLLANAQSRLSSVSLTSQHSKLDGAVVTHVHSKIVENYERWCLFLRKKSQVKSGTVEHKVCI
MALYLLIWGEAANIRFLPECLCYVFHHMADEMYDLLGKPRVERSEKIYIEGSQYSFLEKIICPIHEILAAESDVPAHGRAAH
SAWRNYDDFNESCFELSWPWRLEAGFFLKPKKDVDTDDDNVPEQSFTAGRQRERKLGKTHFVEHRTFLHLYHSFHRLW
IFLVCMLQGLTIFAFCNQKLDTHSIKYILSVGSTFIVMRFVQCVLDVILMFGAYRSTRGRTVARMLIRFLWFALLSAVIVFIYV
KVLIEESKTPAKDTWFTLYYLILGVYGGIQLFLSLLFHIPFLRSQADRCSDVSFLQFFKWVKEERYYVGRGMYEKTSDYVKYT
MFWLLIGLCKFAFSYFLQIKPMVKPTRIIIGLHNIQYRWHDLISKSNHNALTLLSLWAPVVMIYFLDTQVWYTVMSALVG
ALEGARMGLGEIRSLDTLRQRFTTFPEAFVKHMQPTKTSELPRSISMGAEAVKEKENAIRFAPLWNEIIVCLREEDLISNRE
KLLLMMPDNKITNSRTHPQQSLVQWPLFLLANKVYVAKDILSETKYATAQDELWERMKNDLYLAYAVQEAYESLQVVLSA
LLNEDGHHWVEDVFREIDLAIEKGEFGIDIDQNFRKFDLKKLGTVLDKTAKLTAILVKEAEKKDEKEREQEISEMHSAARRA
LVDLYEVVMHDFITDQNLRATCGNAAHLAAKQKGQLFSGLTWPSDKEKMLVSRLHYILSIKDSALSVPSNLEARRRLQYFT
NSLFMKIPESPSVRKMFAFSVFTPYYEEDVMYSLVQLNKKNIDGITTLFYLQKIFPDDWTNFKERMLPLVKEDDLYMKTE
DDIKDTRELRLWASYRGQTLARTVRGMMYYKRALILQAAEEGAFKTDLLDEEQGLASPMTSQGASRRSAQAQAELKFT
YVVSAQIYGSQRSSSKKEDQEKAADISFLMQKNDSLRIAYIHVVKKGKDGKQSEYYSKLVKADPSGKDQEIYSIKLPGKFLL
GEGKPENQNHAIIFTRGDALQTIDMNQENYMEEAFKMRNLLEEFDSDKHGRRRPTILGVREHVFTGSVSSLAWFMSM
QESSFVTLGQRVLARPLKVRMHYGHPDVFDRIFHISRGGISKASRVINLSEDIFAGFNSTLRQGNVTHHEYIQVGKGRDV
GLNQIALFEAKVASGNGEQSLSRDVYRLGQLFDFFRMMSFFYTSVGFYVTTMMTVLTLYVFLYGKAYLALSGVDASLQA
DNNIIQNAALQSALNTQFLVQIGIFTAVPMIMNLVLEQGILKAIISFCTMQLQLASVFFTFSLGTRTHYFGRTILHGGAKYR
STGRGFVVTHIKFAENYRMYSRSHFTKAMEIIMLLIVYLAYGAEDRKAVNFILLTFSSWFLALSWLFAPYIFNPSGFEWQKT
VEDFEDWTKWLFYKGGVGVKIENSWEAWWFDEQTHIRTNTSRFWETILSLRFFIFQYGIVYHLHVDEHSTHLNVYGFS
WLVLIAIVVIFQLFTFTHKSPKVQRLVRLFQTLLFMSIVTGIIVAVVLTPLTIGDVFAVGLALIPTGWGLLSVAIACRPAVKGLR
LWESVQEIARAYDACMGMLVFIPIAFLSWFPFVSTFQTRLVFNQAFSRGLEISLILAGNRPNSSV----------------------------
--------------------------------------------------------------------------------------------------------------------------------------
-----------------------------------------------------------------------------

>RCBT 2000510 Sphagnum palustre 1758

ETVDHHLKKQMENDAKKIREFKPFNILPLETPGVVNAFQMFPEVTAATGALEWSTPPFPEFPDGYEQPERALDVFDFLQ
YVFGFQEDNVANQREHLILLLANAQSRLSSVSLTSQHSKLDGAVVTHVHSKIVENYERWCLFLRKKSQVKSGTVEHKVCI
MALYLLIWGEAANIRFLPECLCYVFHHMADEMYDLLGKPRVERSEKIYIEGSQYSFLEKIICPIHEILAAESDVPAHGRAAH
SAWRNYDDFNESCFELSWPWRLEAGFFLKPKKDVDTDDDNVPEQSFTAGRQRERKLGKTHFVEHRTFLHLYHSFHRLW
IFLVCMLQGLTIFAFCNQKLDTHSIKYILSVGSTFIVMRFVQCVLDVILMFGAYRSTRGRTVARMLIRFLWFALLSAVIVFIYV
KVLIEESKTPAKDTWFTLYYLILGVYGGIQLFLSLLFHIPFLRSQADRCSDVSFLQFFKWVKEERYYVGRGMYEKTSDYVKYT
MFWLLIGLCKFAFSYFLQIKPMVKPTRIIIGLHNIQYRWHDLISKSNHNALTLLSLWAPVVMIYFLDTQVWYTVMSALVG
ALEGARMGLGEIRSLDTLRQRFTTFPEAFVKHMQPTKTSELPRSISMGAEAVKEKENAIRFAPLWNEIIVCLREEDLISNRE

KLLLMMPDNKITNSRTHPQQSLVQWPLFLLANKVYVAKDILSETKYATAQDELWERMKNDLYLAYAVQEAYESLQVVLSA
LLNEDGHHWVEDVFREIDLAIEKGEFGIDIDQNFRKFDLKKLGTVLDKTAKLTAILVKEAEKKDEKEREQEISEMHSAARRA
LVDLYEVVMHDFITDQNLRATCGNAAHLAAKQKGQLFSGLTWPSDKEKMLVSRLHYILSIKDSALSVPSNLEARRRLQYFT
NSLFMKIPESPSVRKMFAFSVFTPYYEEDVMYSLVQLNKKNIDGITTLFYLQKIFPDDWTNFKERMLPLVKEDDLYMKTE
DDIKDTRELRLWASYRGQTLARTVRGMMYYKRALILQAAEEGAFKTDLLDEEQGLASPMTSQGASRRSAQAQAELKFT
YVVSAQIYGSQRSSSKKEDQEKAADISFLMQKNDSLRIAYIHVVKKGKDGKQSEYYSKLVKADPSGKDQEIYSIKLPGKFLL
GEGKPENQNHAIIFTRGDALQTIDMNQENYMEEAFKMRNLLEEFDSDKHGRRRPTILGVREHVFTGSVSSLAWFMSLQ
ESSFVTIGQRVLARPLKVRMHYGHPDVFDRLFHISRGGISKPSRGINLSEDIFAGFNSTLRQGNITHHEYIQVGKGRDVGL
NQIAAFEAKVASGNGEQTLSRDVYRLGQLFDLFRMLSFFFTSVGFYVTTMMTVLTVYAFLYGKVYLALSGVEASLETAGNI
LSNAALQTALNTQFLFQIGVFTAVPMIMNFLLEQGVFTAVISFCTMQLQLSSVFFTFSLGTRTHYFGRTILHGGAKYRSTG
RGFVVEHIPFAENYRLYSRSHFVKAMEIIMLLIVYRAYGAQSRTSVSYILLTFSSWFLAISWLYAPYIFNPSGFEWQKTVKDF
EDWTNWLFYKGGIGDKGLKSWEVWWNEEQAHIQTTRGRIWECILSFRFFIIQYGVVYALHVTGRDKNFNVYGFSWVV
LVGVLFKGFTLSKKASANFQLLVRLFQGVLFLVCIGGLSFALAYTALTVGDMFAAGLALIPTGWGLLSIAIAVRPVIERVGLW
KSVRGIARLYDACMGMVIFIPIALLSWFPFMSTFQTRLVFNQAFSRGLEISVLLAGDQPNASF------------------------------
--------------------------------------------------------------------------------------------------------------------------------------
-----------------------------------------------------------------------------

>RCBT 2000511 Sphagnum palustre 1769

VIESLLSDADPVEVSKLDSASGKEILEEDAKKIRKRRAYNILPMEEDGVSNPFSYFPEVTGATKALLYTEGSPRFPYDYVMPE
NRNLDVFDFLHYVVQDMFGFQKDNVANQREHLILLLASSQSRKGVLRHGQRTEGSAVNEAAVDDVYERVLGSYMRW
CDFLLKEPKAKKAKDATWQEKIYLIALYLLIWGEAANLRFMPECLCYIFHHMAHDMFELLRKEEVEWSSQTAKPSEDGSR
KLCFLEQVITPVYQIVAAEAHNNGNGVASHSAWRNYDDFNEFFWQADCFDHLSWPWKEDAAFFMKPKKRSYDDNDK
ILQHSEPAALRSQTRGHRVGKINFVEHRTGFHLYHSFYRLWIFFICMLQGLTIWAFCNQNFHVRTIKKILSIAPTFAILTFFQS
VLDVLLMWGAYRSTRHHIVRMLIRLVWFGAVSGGVIFLYVKTLLEDRQGTGSTIWFRIFYLVLGSYAILHLLIGMLSHIPWL
RIRTAEWSKYRVIRFIKAVHQERYFVGRGMYERLWDYFRYVLFWVFVLFCKFSFSYHFQLLPMVVPTRLIVELNNINYVW
HDFVSKNNHNALTLLALWAPVVMIYFLDVQVWYTVTSALLGGLEGAKDRLGEIRDLSMLRKRFIDYPQALVQRLQPMN
SSRVSRQISLAEGPVTDGKAIRSKQDAINAIKFAPIWNEVIKSLREEDLINNKEKELLIMPEQDVMQQNSWRIHWPLFLVA
NKVHVAVELAAGNKKNLELLWEKVNRVEYMANAVEEAFETLQPVLENLLNADGAQWVRSLFGDIKEAIGSQAFVANF
NLTNLREMLKMTRKITKQLWRNKTAERVSKVEAALQRLQAVVMNGFLPQDIREGFERWDRFERPLFTNLNWPDRNG
QKDAKRLFNLLTVQKINASKTLDTETIPRNLEARRRLQFFTNSLFMHMPEAPTIRKMFSFCVFTPYYAEDVMYDLKKLCEE
NKDGISILFYLQKIYPDEWQNFLERIGLTGRTVDTKVDEKNEEVILQLRLWASYRGQTLARTVRGMMYYKRALELQAAQE
GASTTDVEEGLQLSGGLLERSAKYQAELKFTYVVTCQIFGQQKKEGAVQAADILYLMHKYDSLRIAYIDVVETTKDKMVT
KSYYSKLVKADPYGQDQEIYSIKLPGEVKLGEGKPENQNHAIIFTRGDAIQTIDMNQDNYLEEALKVRNLLAEFDREDISLR
PPTILGVREHVFTGSVSSLAWFMSMQESSFVTLGQRVLARPLKVRMHYGHPDVFDRIFHISRGGISKASRVINLSEDIFAG
FNSTLRQGNVTHHEYIQVGKGRDVGLNQIALFEAKVASGNGEQSLSRDVYRLGQLFDFFRMMSFFYTSVGFYVTTMMT
VLTLYVFLYGKAYLALSGVDASLQADNNIIQNAALQSALNTQFLVQIGIFTAVPMIMNLVLEQGILKAIISFCTMQLQLASVF
FTFSLGTRTHYFGRTILHGGAKYRSTGRGFVVTHIKFAENYRMYSRSHFTKAMEIIMLLIVYLAYGAEDRKAVNFILLTFSS
WFLALSWLFAPYIFNPSGFEWQKTVEDFEDWTKWLFYKGGVGVKIENSWEAWWFDEQTHIRTNTSRFWETILSLRFFI
FQYGIVYHLHVDEHSTHLNVYGFSWLVLIAIVVIFQLFTFTHKSPKVQRLVRLFQTLLFMSIVTGIIVAVVLTPLTIGDVFAVG
LALIPTGWGLLSVAIACRPAVKGLRLWESVQEIARAYDACMGMLVFIPIAFLSWFPFVSTFQTRLVFNQAFSRGLEISLILAG
NRPN-----------------------------------------------------------------------------------------------------------------------------------
----------------------------------------------------------------------------------------------------------

>RDOO 2006785 Racomitrium varium 1759

IYNILPVDYPEVDHAGMLFPEVKAALAALQNVRNLPVPTDLRTWTLESDMLDWLGNFFGFQRDNVRNQREHLVLLLA
NGLMHLFPEPMSFNTLEANVVTMIRKKVTGNYAQWCRFTGCRNNMKLLKKRRGQQEQDEDQKVEMGRELMYICLFL
LIWGEAANLRFMPECLCYIYHHMLVDLNAVLDSSEDDIEVPGEMPSFLNHIVVPIYEVVKAEADSNNGGTAPHSSWRNY
DDMNEYFWSSRCFDQLDWPIRRDCSYLKEPKIEKGYLHRGRKVQHQKVGKTGFVEQRSFWYIFRSFDRIWVAHILVLQA
SVMTLWHNGGPPWIELQKPDPLARFLSIFITWSFLRVLQGLLDIGSQYSLVSRETLLTGVRMILKPLVATTWAILFIIYYRRM
WWQRNVDGYWTVYANNRLHEYLYICAAFIVPEVLALVLFILPWVRNFVENSSWRIFHVLTWWFQSRLFVARGLREGV
MDNLKYSLFWLSVFASKCAFSYWLQIRPLIAPTKQILRANNVTYRWHEFFPNGSRAAIVALWAPVLLIYFMDVQIWYSIW
SSGVGAFVGLLQHLGEIRNVDQLRLRFKIFPTAFDFSLMPPKDTGHLTLWENAKDLIKRFRLRYSTIYDKVEWGQLEAGRF
AHVWNKIVKTFREEDLISDREVELLEVLEVGGSWRVSVFQWPSILLANQILQVVKKEVQYFKGNDKQLWVIISKNEYRM
CAVIESYESIKHILLRRLLRNDSVEYNIIQSCFEEIDASIRNQRFTSSFLLRELLNIHTRVVHLIEVLLNKPTASQTQEVVDALQS
LYECLVEDFPRDSGIKDSIKAHLRGLNKETELFMDAVTLPNKDDEPFFKHLLRLHTTLSTTDPLLNVPKGLEARRRISFFSNS
LFMTMPRAPQVDRMLAFSVLTPYYSEDVIFSTKALKEENEDGITILFYLQRIFPEDWANFLERMRKQDLLEHRLWEKDDA
IELRLWASYRGQTLARTVRGMMYYEKALEVQAFLDTASEIDMLGIKELLERGLERGGSTNSRGSFGTEHVSIQMNRDHK
VQQNLAAASMKFTYVVTCQIYGQQKKNNDYQAADILRLMRTYPGLRIAYVDEVRTGKDVNYYSVLVKYDSGLRNEVEIY
RIQLPGPLKLGEGKPENQNHALIFTRGDAVQTIDMNQEMYFEEAIKMRNLLQEFTVRHGTRKPTILGVREHVFTGSVSSL
AWFMSAQETVFVTLSQRVLANPLKIRMHYGHPDVFDRLWFLTRGGISKASRTINISEDIFAGFNCTLRGGNVTHHEYIQA
GKGRDVGLNQIAMFEAKVASGNGEQILSRDVYRLGHHLDFFRMLSFYYTTVGFFVSNMMVVLTVYTFLWGRVYLALSG
IETSLTGGSAALTNAALTATLNQQLVVQLGLLTALPMLVEDALENGFTTALWDMITMQLQLASIFFTFSMGTRCHYFGRTL
LHGGAKYRATGRGFVVKHEKFAENYRLYSRSHFVKGIELLLLLLCYLAYGVVSSSAAYILVNISSWFLALTWILAPFIFNPSGF
DWLKTVEDFGDFMQWLWFRGDVFVKVEQSWEIWWDEEQAHLRTTGLWGKLLEIILDLRFFVFQYGIVYHLGITGGSR
SIFVYLASWSYMAFAAILHLVLSNANERFAANNHGLYRAIQALTIAVIAAIVVVLWIETDFKVLDIVASFLAFLPTGWGIIQIC
QVLRRPFLENSPVWGTIVAVARLYDLAMGLIVMAPVAILSWLPGFQAMQTRILYNEAFSRGLQIQRLLAGKKNRNID-----
----------------------------------------------------------------------------------------------------------------------------------
-------------------------------------------------------------------------------------------------

>RDOO 2006786 Racomitrium varium 1772

MGDLVYNIVPVDDIAAATDHPAVQFPEVRAAVRALRSIGDLRKPTHTQWRGNMDILDWLGCWFGFQSSNVKNQREH
LVLLLANAQMRATPESTDKLDGRVMRKIRRKVTKNYESWCTFVGKHKELRLSSGKRAGDERGERLELLYTSLYLLIWGEA
ANLRFMPECLCFIFHNMAQELNKMLERDSDRGFKPTTCETNGFLKLVVSPLFEVVKAEAQVNKDGNAAHSSWRNYDDI
NEYFWSDRCFTHLRWPMEQGSNFLVKPEDKSKHKVGKTGFVEKRSFFNIFRSFDRLWIGYILVLQACIVTLWSGQQRAP
WVELQNKDSLARLLTIFITWSALRLFLALLDVVMQYSLVSWETWTTGLRMILKVLVAGIWVAIFSIFYRIMWNKRHQDH
AWSAAANILFNRYLYTMGAFVLPEGLALALFIIPFVRNVLETSSFKLFHLMTWWFQTRSYVARGLREGIVDNFKYTLFWIL
VLASKFLFSYFLQIKPLIRPTKEILTLTNIEYRWHQIFSKGNRAAVLALWAPVILIYFMDTQIWYTIWSALVGALVGLMDHLG
EIRNVHQLKLRFQMFPRAVQFNLIPEAWGANIKQQYSWWVNVKNFFQRIRLRYGVPSQEAKEENMEAKRFSHIWNEIL
KTFREEDLISNRELELLEIPAPVWNISVFQWPSSLLANEVYTALGLVKDKHLDDKAVWKKIAKSEYRRCAIVESYESIKHILVY
KILRQNSADHILVKSLFEDHIDSAINSRKFALAFSLGKLPEVHQCLLTVVKKILAQKTDEVIEALQALWHCVVNEFARSEERG
FIKQNFVEKHINTSTVFKDSVVLPEKRDKAFYKQIKRLQTTLETKDTLLSVPKGLEARRRISFFANSLFMTMPRAPQVEKM
CAFSVLTPYYEEEVIYSLKDLNTTNEDGITTLFYLQKVFPDDWKHFKERLRRSDGLDETDKEFIDRMSGLDIAEGGIQKKN
DTKNYSKKGDEDGARLQDEPGLELCLWASYRGQTLARTVRGMMYYERALECQAFLDAATEKDLNEALGFKEMLVRASS
SVSEGSSRRQENIIPDETRKRQVLATAAMKFTYVVAAQVYGKQKKNKANQAKGILYLLETYKGLRIAYVDEVEAPTGKQYF

SVLVKYDRDAKMEMEIFRVQLPGPLKQGEGKPENQNHALIFTRGDAVQTIDMNQEMYFEEAFKMRNLLEEFTRFHGIR
KPTILGVREHVFTGSVSSLAWFMSAQETVFVTLNQRVYANPLKIRMHYGHPDVFDRLWFIGRGGISKASRTINISEDIFAG
FNCTLRGGTVTHHEYIQAGKGRDVGLNQIAMFEAKVASGNGEQMLSRDVYRLGHHLDFFRMFSFYYTTVGFFVSNM
MIVLTVFVFLWGRVYLALSGIERSLTTGSSALANAALTATLNQQLVVQLGLLTALPMLVEDALEHGFTTALWDMITMQLQL
ASLFFTFEMGTRSHYFGRTLLHGGAKYRATGRTFVVKHEKFAEMYRLYSRSHFTKGIELLMLLFCYLAYGVVSSSATYIVVM
ISSWFLAFTWIMAPFIFNPSGFDWLKTVVDFDDFMQWLWFKGDVFVKVEQSWEVWWEEEQSHFRTTGLWGKLLEIL
LDLRFFIFQYGIVYHLQITGNNTSIFVYLLSWTYMLAAILLHLVISNASDRYAADRHGLYRLIQTLTIAVIVAIMIVLGTKTNFT
FLDILASFLAFLPTGWGILQICLVLRRPFLENSRVWGTITAVARLYDLGMGLIIMFPVAVLSWLPGFQAMQTRILYNEAFSR
GLQISRLLA----------------------------------------------------------------------------------------------------------------------
-----------------------------------------------------------------------------------------------------------

>RDOO 2006787 Racomitrium varium 1780

MGDLVYNIVPVDDIAAATDHPAVQFPEVRAAVRALRSIGDLRKPTHTQWRGNMDILDWLGCWFGFQSSNVKNQREH
LVLLLANAQMRATPESTDKLDGRVMRKIRRKVTKNYESWCTFVGKHKELRLSSGKRAGDERGERLELLYTSLYLLIWGEA
ANLRFMPECLCFIFHNMAQELNKMLERDSDRGFKPTTCETNGFLKLVVSPLFEVVKAEAQVNKDGNAAHSSWRNYDDI
NEYFWSDRCFTHLRWPMEQGSNFLVKPEDKSKHKVGKTGFVEKRSFFNIFRSFDRLWIGYILVLQACIVTLWSGQQRAP
WVELQNKDSLARLLTIFITWSALRLFLALLDVVMQYSLVSWETWTTGLRMILKVLVAGIWVAIFSIFYRIMWNKRHQDH
AWSAAANILFNRYLYTMGAFVLPEGLALALFIIPFVRNVLETSSFKLFHLMTWWFQTRSYVARGLREGIVDNFKYTLFWIL
VLASKFLFSYFLQIKPLIRPTKEILTLTNIEYRWHQIFSKGNRAAVLALWAPVILIYFMDTQIWYTIWSALVGALVGLMDHLG
EIRNVHQLKLRFQMFPRAVQFNLIPEAWGANIKQQYSWWVNVKNFFQRIRLRYGVPSQEAKEENMEAKRFSHIWNEIL
KTFREEDLISNRELELLEIPAPVWNISVFQWPSSLLANEVYTALGLVKDKHLDDKAVWKKIAKSEYRRCAIVESYESIKHILVY
KILRQNSADHILVKSLFEDHIDSAINSRKFALAFSLGKLPEVHQCLLTVVKKILAQKTDEVIEALQALWHCVVNEFARSEERG
FIKQNFVEKHINTSTVFKDSVVLPEKRDKAFYKQIKRLQTTLETKDTLLSVPKGLEARRRISFFANSLFMTMPRAPQVEKM
CAFSVLTPYYEEEVIYSLKDLNTTNEDGITTLFYLQKVFPDDWKHFKERLRRSDGLDETDKEFIDRMSGLDIAEGGIQKKN
DTKNYSKKGDEDGARLQDEPGLELCLWASYRGQTLARTVRGMMYYERALECQAFLDAATEKDLNEALGFKEMLVRASS
SVSEGSSRRQENIIPDETRKRQVLATAAMKFTYVVAAQVYGKQKKNKANQAKGILYLLETYKGLRIAYVDEVEAPTGKQYF
SVLVKYDRDAKMEMEIFRVQLPGPLKQGEGKPENQNHALIFTRGDAVQTIDMNQEMYFEEALKMRNLLEEFDKYHGV
RKPTILGVREHVFTGSVSSLAWFMSAQETSFVTLGQRVLANPLKIRMHYGHPDVFNRLWFMSRGGISKASRTINISEDIF
AGFNCTQRGGTVTHHEYIQAGKGRDVGLNQIAMFEAKVASGNGEQVLSRDVYRLGHRLDFFRMLSFYYTTVGFFINNLI
VVLTVFAFLWGRVYLAVSGVEGSLTSSKVLSNTALLASLNQQLIVQLGIMTALPMIVENALEHGFTKALWEFFTMQMQL
ASVFFTFSMGTRAHYFGRTLLHGGAAYRATGRGFVVKHERFAVIYRLYSRSHFVKAIELIGLLIIYRVYGASRSSNTYLFISLTS
WFMSLTWIVGPFIFNPSGFDWLKTLEDFEDFMTWLKYKGGFIVGSEQSWERWWLDEQKHLEYTGLWGKLADIILNLR
FFFFQYGIVYQLNIAATSQSIFVYLISWSYVFVAGLIHVIIASAGSRFATKRHGLYRAIQAGLIALVVLVIVLLKIFTAFNLRDLLT
SLLAFVPTGWGILQIIAVLRTRRLEKSFVWPVVVNVARLYELGMGLIVLAPVAILSWLPGFQAMQTRVLFNEGFSRGLQIS
QLLVTVQKAKKSE----------------------------------------------------------------------------------------------------------------
-----------------------------------------------------------------------------------------------------------

>RDOO 2016948 Racomitrium varium 1987

QRISKRVLNKWELLVYRAKMAAERRAIDEPQGPWAGSASNTTVPQTLLQEANIDAILQTGDELAKENAHVARILTEYAYT
LAQSLDPNSEGRGVLQFKTGLLSVIKQKRSKKEGEKIDRSHDVRILQEFYKHYREKNHLEQLENEDRIRRTSDLYEEDSTTT
EQRAETQRKIYLTARILNEVVDALTKDSQTEELNPELKRIMEEDAKKVKGFKAYNILPLDTPGVANVFANFPEVVGATRAL

WYSSESAQDLPDFPDDAGVADLDRHQDIFDVLQYAFGFQADNAANQREHLILLLSNSQSRLRIMIDTETKLDDAAVSNV
HDKMMANYERWCKFLRRDSMAKRAFTVQLRLFLTALYLLIWGEAANLRFLPECLCYIFHHMADELYDLLDRPVLERSRTF
LPDSPHSFLDKIVKPIHEILVAEAKICAGGRAAHSAWRNYDDFNEFFWAPSCFELSWPWRPDAGFFRKPKKRIYTEADRY
EPTAVEQESLPMLGETREKKVGKTHFVEHRTGFHIYHSFHRLWIFLVCMLQGLGIFAFCDRRLTVRNVKLIMSVGPTFVL
MKLIQSVMDVTLMIGAYRSTRARNISRMLIRFLWFTILSVIIVLLYVKTIEEEKSGSGTDKWFKAFYWVLGIYGGLQLFFAVL
LRVPWFRMQADKCSNFYVVMIIKWVHQERYYVGRNMYERTRDYFKYTLFWFVVGTCKFAFSYFLQIQPMVGPTRTIV
GIRNVKYRWKDLISQSNHNALTLVSLWAPVVMIYFLDTQVWYTVVSALVGGFNGARMHLGEIRSLDMLRSRFSSLPGAF
VKTLVPSRSGGHSQMDVNVPHSAVKPGNPRVDAIRFAPLWNEVIHSLREEDLINNREREWLLMPNNTISSGALGQQTL
VQWPLFLLANKVYYVGIDIVKDNRQSFQDELWDRIRRDPYLEYAVQEAFATLQTVLIDLLNEHGRAWVLEFYHDVHQALE
VSQVLYKFNFEELGNVVTKIANLTEILVSKPKKAPDDSGRQDEELKMQENAARALVDLYEVVMRDFLADMELRGKYEDD
VVLQASKLDGRLFSDLTWPTGPAKEQARRLNYILAIKDSALNVPVNLEARRRLQFFSNSLFMSMPQPPPVRKMFSFSVFT
PYYSEDVMYSKAQLEDRNVDGITILYYLQTIVPDEWKNFLERMFPGADYNQLGHYSEADIPEADILQLRLWASYRGQTLA
RTVRGMMYYKKALVLQAQQEGASMAEDEEGGQDLEGHDLEGNELAIVNVRGSPRTPRTPRTPRGSLLRTARAQAELK
FCYVVTAQIYGKQKNSTLQADKDRAADILYLMQTNDSLRIAYVHEAKQMIDGKPYTEYSSKLVKADPSGKDQEIFSIKLPG
EVILGESKPENQNHAIVFTRGEALQTIDMNQEHYLEETLKMRNLLEEFDSKSLGLRAPTILGVREHVFTGSVSSLAWFMSL
QERSFVTLGQRVLANPLKVRMHYGHPDVFDRIFHITRGGISKASNQINLSQDIFAGFNSTLRQGNITHHEYIQCGKGRDV
GLNQIAAFEGKVASGNGEQTLSRDIYRLGQLFDFFRMCSFFFTSVGYYFTTMLTVLTVYVFLYGKVYLALSGVDEALKASN
LLDNTALQAALDTQFLLQIGVFTAVPMIVNFILEQGVLRAVISFFTMQLQLSSVFFTFSLGTRTHYFGRTILHGGAKYKSTG
GGFVVEHIPFAENYRTYARSHFVKGMEIILLLIVYLVYGAHDRTAASYILLTFSSWFLALSWLYAPYIFNPSGFEWQKTVIDFE
DWTNWLFHKGGIGDEGKKSWEVWWDEEQAHIQTPRGRFWEILLSTRFFIFQYGVVYALNAAGGNKSFWVYGYSWV
VIVGVFILFKIFTFSQKASANFQLIVRLFQGIVFLAVVAGVSVAVVLTNLTIGDVFASALAIIPTGWGLLSIAIAIRPIVKWCGL
WKSVRGIARLYDAAMGMIVFIPIALLSWFPFVSTFQTRLVFNQAFSRGLEISVLLAGNNPNAAI------------------

>RDOO 2019568 Racomitrium varium 1999

MERPPRPPHRISRRVLGNWEKLVARAVRDSVLASSSNRDGRGRGSNSGQRGQMAPQSLAQQANIDAVLQAVDEIEQE
NIQVARILAEHAYWLTQQLDPRSEGRGVLQFKTRLKSIIKQKQQQAKHGEVDRSQDIRVIQEYYKRFRERNNIDALEAEA
QMRRQGWSTDLVPDSQDQSFEKLRRIYEISSILNDVVDALLKEAEPGDASRLQDTDEKRVMEEDAAKVKGFRAYNILPLE
TPETPAVLNPFEYFPEVVGATKILRYTLDLPRFPSDAELPTDRVLDIFDFLYYAFGFQKDNVANQREHIILLLASAQSRLGTLD
QSREGDTDKLADKAIGDVHERILQNYVRWCHFLRREPQSKRAFTQQRRLCLTALYLLVWGEASNLRFMPECLCYIFHNLA
DECFDLLDRTYVERSRTVKPNADNSIEYSFLEQIITPVYDVVAAEAKFSQGGKIPHSHWRNYDDFNEYFWQPSCFLELRW
PWNLDAGFFKPPKVKGDKKKAFNIAEPNPGNETDPLVLPPGRSKPKVKRKVGKVHFVEHRSGFHLYHSFHRLWIFLVCM
LQGLTIWAFCSKNGNLNLHVRTIKRIMSVGPTFVVMKLIQSLFDVVFMWGAFRSTRLQTVARMLLRLIWFGCLSAAILFL
YIKTLQEDARDDGSGSWFRIYYILVSSYAGANVVFVFLLRIPWLQRQAAKCSNVYIFQFIKWLHQERYYVGRSMYERTRN
YAKYSIFWIFILACKFAFAMHFQIMPLVVPTRLIIGFDNIKYKWPDFVSSSNHNALTILSLWAPVIMIYFLDTQVWYTVVSAL
LGGIEGARDKLGEIRTLDMLRKRFPNYPGAFVKHMQPPVNRFSSDTVLQPGESTHNKAIRDKRDAIKFQPIWNKVVKSL
REEDLINNREKLLLKMPPNPTLHPNGAPDNLVHWPLFLLANKVHIAVELAAQHKSTDRDGLWKKVMHDEYMACAVEE
TFETLEPLLLSVLNPAGRSWVHSVFGDIRRYMLGYGEDVGDSFTVQFRLSALKDVLDKLRDLTEQLGNEDVPERRTKTTLA
FQKLYNVVVRDFLPEVSRDHLAGWQFHQDSLRNGTLFSDLYWPDKAKQKQAIRLNNLLTVQKIKDQEGKTKTLNTETIP
HNLEARRRLQFFTNSLFMHMPQAPPIRKMFSFSVFTPYYTEDVMYDMDKLYAENEDGITILFYLQKIYPDEWYNFLERIG
LIESIVMRVVENPKSEGDVKVKLELRLWASYRGQTLARTVRGMMYYKQALILQGKQEGASGGDLEEGIPPSLVEAQGSIA
RSAVAQAELKFTYVVTCQIYGEQKRQGKVQAADILYLMQTYESLRVAYIDVVEGSAKGKKPSYYSKLCKVNRSDPTRRDQ
EVYSIKLPGDVRLGEGKPENQNHAIIFTRGDCIQTIDMNQDNFMEEALKMRNLLEEFNQTSHGLNRPTILGVREHVFTG
SVSSLAWFMSMQESSFVTLGQRVLARPLKVRMHYGHPDVFDRIFHITRGGISKASRGINLSEDIFAGFNTTLRVGNVTH

HEYIQVGKGRDVGLNQIALFEAKVASGNGEQTLSRDVYRLGQLLDFPRMLSFFHTSVGFYVTTMMTVLTLYVFLYGKAYL
ALSGVDASLKKSSDILQNAALKAALNTQFLFQIGIFTAVPMIVNLILEQGILKAIISFCTMQLQLASVFFTFSLGTRTHYFGRT
ILHGGAKYRSTGRGFVVTHIKYAENYRLYSRSHFTKALEVIMLLIIYLAYGAQNRTSVTYVLLTFSSWFLALSWLFAPYIFNPS
GFEWQKTVEDFEDWTNWLFYKGGVGVKTDNSWEAWWIDEQDHIRTLRGRFWEFVLSIRFFLFQYGVVYSLSVTRGSS
SISVYAYSWLVLLGLVIIFKVFTVSQKASASFQLGVRLFQGLLFTSLVAGLIVAIVLSPLTVGDVFAVALAIIPTVWGLLSIAIAFR
PLMERMHVWKSVREIARVYDACMGMLIFIPIAFLSWFPFVSTFQTRLVFNQAFSRGLEISLILSGNRPNR------

>RDOO 2020191 Racomitrium varium 2005

MAPRMPHLVRKRVVAKWKNLVRLAIEAEEERTLHESHGDVYGDTSFVPQILHQQKKNIDEILQTARDVEQTYPQVARILY
EYSYALSQTLDPRSESRGVLQFKTGLLAVIKQKRGEKTDRSDDVYIIEGFYKDLKRNLDQLEDEDWLQQQPQYSQISPED
WTELKRKIYVTCQILNEVVDFLIKENPEAKRHVEFDSNLKEDLEKTAQKLDDYKPYNILPFEAPGVVNRFRNSPEVVAAIN
AIEWDLRNDHEFDADFKRPKLRNLDIFDFLQFAFGFQADNVLNQREHLVLLLANSQSRLGPLRNTVPDLLPKLDETAIFN
VNKKLLDNYERWCDFLRKDPKTNRRLDSSSAMPQSRLFFSALYLLIWGEAANVRFLPECICYIFHHMADEMVDLIEKPFH
QKSMILKECDSFLDLVIKPVHEIVAAEAKVCNHGKSPHSRWRNYDDFNEYFWAPFCFELGWPWRLEAGFFVKPKQDSS
KKSIKTNSRNSQDQVPLLQEQNQPSVAPGRRRERKAGKSHFVEHRSGLHLYHSFHRLWIFLVCMLQGLAVFSFCDGKLN
TTSIKYILSVGPTFVTMKFLQSVLDVVLMIGAYRSTRARTLSRIWLRLFWFGSLSAIIVLLFVKTIQEQESGSGSNTWFNLYV
VLLIIYGGSHLFVALLLNMPWLRRQTEKCSHFGPVPFLNWVHQERFYVGRGLYERSGDYFSYILFWVIILACKFSFSYFLQIR
TMVGPTRIIIDLTDINYQWRDIFSKSNHNAMTLVSLWAPVVMIYFLDLQVWYTVISALVGGLDGARMGLGEIRSLDMLR
TRFTYLPEAFTKRMQPHQTHEPQTMRESLNIRKGKVDAKHFAPIWNEVIISLREEDLINNKEKEWLVMPGNTLTSLTTSS
QHLSLVQWPLFLLANKVFVACDMADVHKQATQEELWERIGRDSYMMFAVQEAFYTLRIILDHLLVHDQGGRWVAKVY
ESMEQAMIIGQLRYKFHLKKDQLVKVLDKAAKLTNVLINEAKKSDDFLKENAEINKKMRDDYTAAVGKNLLDFYDVVMQ
DFIADSETRTDVKGDRDLHAAIQNGKLFAELSFPTDESTSLVRRLNNILTIKESALNVPVNLEARRRLEFFSNSLFMKMPDA
PPVRTMFSFSVFTPYYSEDVIYSPQQLAKENDDGISMMYYLRTIVPDEWENFLERIYPNKKEDREARKALLKTIFPKEFRFK
PNEEQKNPEDLNEEVKLKLRLWASYRGQTLARTVRGMMYYKRALILQAQEEGASFSDDLEQGSQHSTSSASQDLGILDA
RAQAELKFLYVVSAQIYGEQNQGGKGAEGRQKAGDISYLMKMNDSLRISYIHKGKVKVGDKEVTEYYSKLMKADPSGN
DQEIYSIKLPGEVILGEGKPENQNHAIVFTRGEALQTIDMNQEHYLEETFKMRNLLEEFDSRKYGLPDRRPTVLGVREHVF
TGSVSSLAWFMSLQERSFVTLGQRVLAKPLKVRMHYGHPDVFDRIFHITRGGISKASKQINLSEDIFAGFNSTLRQGLITH
HEYIQCGKGRDVGLNQIAAFEGKVASGNGEQTLSRDIYRLGQLFDFFRMLSFFFTTVGYYFTTMLTVLTVYVFLYGKVYLA
LSGVDAALKEKGLSANVALQSALDTQFLLQIGVFTAVPMIMNFVLEEGILRAIISFFTMQLQLSSVFFTFSLGTRTHYFGRTI
LHGGAKYASTGRGFVVAHIPFAENYRMYSRSHFVKALEIMLLLIVYLAYGAPERTAFTYILLTFSSWFLAISWLWAPYIFNPS
GFEWQKTVTDFEDWTNWLFHKGGIGEGGTKSWEVWWLDEQSHIQTPRGRFWEIVLSLRFFFIQYGVVYALNVVGHD
KNFRIYGFSWCVLAGIVLIFKVFSVNQKSSANFQLVLRLFQMTVFLGIIAGVIVAVAITNLTIGDVFASALSLIPTGWGLLSIAI
ALRPVMKRLGLWKSIRAIARLYEAFMGAIVFIPIALLSWFPFVSTFQTRLVFNQAFSRGLEISTLLAGNNPNKDM

>RDOO 2020438 Racomitrium varium 1934

MASGGGTESGAALPRRGLARTSTIGGVTESFDSEVVPSSLASIAPILRVANEIEASSPRVAYLCRYHAFEKAHRIDPTSSGR
GVRQFKTALLQRLERDSEPTLALRHRRSDAREIQSYYQNYYNDYVKALDAAENTDRAQLAKAYQTAAVLFEVLKAVNRDK
AEEPPPEIIAAAADVEQKKEIYVPYNILPLDAAGASQAIMQLDEVRAAVEALRNVRGLPWQTLKEPQPRAGDLDCLDWL
QDMFGFQKGNVANQREHLILMLANVHIRLLPRPEPMHKLDDRALNAVMNKLFKNYKSWCKFLGRKNNLWLPQIHQE
IRQRKILYMGLYLLIWGEAANLRFMPECLCYIYHHMASELHGMLAGNVSMVTGDNMKPAYGGEKESFLNLVVTPIYEVIS
RETVKSRNGTAPHSAWRNYDDLNEYFWKVHCFKLGWPMRTDADFFVPARSSSTTTKSINGKVYQSTSKSFFVEIRTFW

HLFRSFDRLWAFYILGLQAMIVLAWNVGTRLQDAFTGTVLKQVLSIFITASILRFLQAILDLVLSYHAFRSLQLSGVVRLVLKL
VTSAAWVIILSICYVRTWVNPQGLIGEIQKWLGKSWESSYLYIAAVVVYLIPNVIGACFFLFPMIRRWIESSNWTVVRVLL
WWSQPRLYIGRGMHESQFALFGYTFFWVLLIASKFAFSYYIQIEPLVAPTRAIMQQTSVTYTWHEFFPNARNNPGALISL
WAPVVMVYFMDSQIWYAVFSTIFGGISGSFRRLGEIRTLGMLRSRFSSLPGAFNESLIPDDGKRARKRFSFSRDFEKVAPS
KDRAKAARFSQLWNEVITSFRQEDLISDKERDLMLVPYSSDPDLKLVQWPPFLLASKVPIALQMAKQAAETGRAVDLLRK
IKYDEYMKCAVTECYESLKRVLKVLIVGEVEIRVIEGLLNQVDQNVDKETLLDNFKLGELPVLSEKFIELLELLEKNHAGQKA
IDSARILVVLKLQDMYEVVTRDMMSDSMRDSWDSSHGALAGGQGRKSELFSSKGDEPAKVLFPPLRTEAWIEQIKRLHL
LLTERESAMDVPENLEARRRIAFFTNSLFMNMPRAPRVRNMLSFSVLTPYYKEDVVYSKENLMKENEDGISVLFYLQKIY
PDEWNNFLQRIGLGTSEDPEGQIFSSTDLMDQLREWASFRGQTLSRTVRGMMYYRRALELQAFLDMATEDELVDGYK
VLTDATPEQKKSQRSTWSQLQAIADMKFTYVAACQMYGEQKRQGHHSATEILKLMLNNPSLRVAYIDEAEEQQNEKPS
KVYYSVLVKAVNGLDQEIYRIRLPGTVRLGEGKPENQNHAVIFTRGEGLQAIDMNQDNYLEEAFKMRNLLQEFHEPHGV
RPPTILGVREHIFTGSVSSLAWFMSNQETSFVTIGQRVLASPLKVRFHYGHPDVFDRLFHITRGGMSKASRVINLSEDIFA
GFNSILRRGNVTHHEYIQVGKGRDVGLNQISLFEAKIACGNGEQALSRDIYRLGHRFDFFRMLSAYFTTVGYYFSTMIVVL
TVYVFLYGRIYLALSGVDDSLVHSANNKALTAALASQALVQLGLLMALPMVMEIGLERGFRTALSDFLTMQLQLASVFFTF
SLGTKTHYFGRTILHGGAKYRATGRGFVVRHERFADNYRLYSRSHFTKGIELFLLLMVYSLYVSKSAKGAVTYILITFSMWFL
VASWLFAPFLFNPSGFEWQKIVEDWDDWNKWMSNRGGIGVEGSKSWESWWDEEQEHLKYTGFIGRFVESVLSFRFF
LYQYGIVYHLNIAQSSNDLSITVYGLSWLVIVAVLTILKIVSMGRDKFSADFQLMFRLLKATLFIGSVSVIAILHVKNFTVGDL
FASILAFIPTGWALLQIAQACKPVVVRLGFWNSVKALARGYEYMMGLFLFTPVAVLSWFPFVSEFQTRLLFNQAFSRGLQ
ISRILAGRKKL----------------------------------------------------------------------

>SKQD 2006862 Takakia lepidozioides 1568

LAGNVSLVTGENMTPAYGGEDEIFLKKVIIPIYNVIAKENARNKNGTAPHSTWRNYDDLNEYFWRIDCFQLGWPMREEA
DFFVPPAIVGRKLPFMKQKLAKKALEPTGKAYFVEIRTFWHLFRSFDRMWAFYILGLQALIVLAWNVGANLSAAFNWTV
FKQVLSIFITASVLRLAQAMLDIVMSYHAYHSLKFFGVLRLLLKLLTSAAWVVILSVCYVHTWNNPTGLIKDLQKWFGNSG
KSSYVYLAAVVVYLVPNFIGGLFFIFPMLRRWIESSHWKIVRFLLWWSQPRLYIGRGMHESQFALFGYTFFWFLLLSTKFV
FSYYVQINPLVGPTKRIMHQTAVTYAWHEFFPHAKSNAGALISLWTPVILVYFMDSQIWYSVFSTLFGGISGSFRRLGEIRT
LEMLRSRFSSLPGAFNENLVPADRKKARRGFSFRRHFDKVQPQKNRLKAARFAPLWNEVIMSFRMEDLISDRERDLMLV
PYSLDPDLDMVQWPPFLLASKVPIALQMAKEADELGRAADLFRKIRHDEYMKSAVTECYSSFKRVLRVLIVGDVEKRVIE
GVLNEVDVNINKGTLLTNFKMSALPVLSAKFLELLELLEADHGDKEGVESARDLAVLKLQDMYEVVTRDLMNESQRDIW
ESSHGSLTRGHEGELFSAVLFPPPRTEAWIEQIKRLHLLMTEREAAMDVPQNLEARRRIAFFTNSLFMDMPRAPRVRNM
LSFSVLTPYYKEDVVYSKDNLNKENEDGVSVLFYLQKIYPDEWDNFLERLGLTTSPDPEAEIFGNEELEDKLREWASFRGQ
TLSRTVRGMMYYRRALELQAFLDMATEDELADGYKVITNTPAEQKKSQRSTWSQLQAIADMKFTYVAACQNYGEQKR
QSHHNATEILKLMLNNSSLRVAYIDEVEERLKDKTDKVYYSVLVKAVNGLDQEIYRIKLPGLVKLGEGKPENQNHAIIFTRG
EALQAIDMNQDNYLEEAFKMRNLLEEFHEPHGVRPPSILGVREHIFTGSVSSLAWFMSNQEMSFVTIGQRVLASPLKVR
FHYGHPDVFDRVFHITRGGISKASRGINLSEDIFAGFNSILRRGNVTHHEYIQVGKGRDVGLNQISLFEAKIANGNGEQAL
SRDIYRLGHRFDFFRMLSCYFTTVGFYSSTLIVVLTVYLFLYGRIYLALSGVDKSLLYLGDNKALTAALASQSLVQLGLLMALP
MVMEIGLERGFRTALSDFFIMQLQLASVFFTFSLGTKTHYFGRTVLHGGAKYRATGRGFVVRHERFAENYRLYSRSHFTK
AIEILMLLIVYSVYASSHKGAITYVLITFSMWLLVATWLFAPFLFNPSGFEWQKIVEDWDDWNKWINNRGGIGVDGNKS
WESWWDEEQEHLKYTGRRGCLIEILLSFRFFLYQYGIVYHLHIVEVSKTHSLSVYGLSWLVIVAVLTVLKIVSMGRRRFSAD
FQLMFRLLKAMLFIGFVSVVAVLFVKNFTIGDLFASILAFIPTGWALLQIAQACKPLVARIGFWDSVKALARAYEFMMGLV
LFTPVAVLAWFPFVSEFQTRLLFNQAFSRGLQISRILAGRRKV-----------------------------------------------------------------
----------------------------------------------------------------------------------------------------------------------------------------

----------------------------------------------------------------------------------------------------------------------------------------------
-----------------------------------------------------------------------------------------------

>SKQD 2009602 Takakia lepidozioides 1762

MAEAVFNIIPVDNFLSDHAALRFPEVRAAIAALQCVGDLAKPPDVRWTSNMDILDWLGGFFGFQRDNVRNQREHLVL
LLANAQMRLQPAPLSIDKLEASVVRKIRKKVTQNYVSWCRFVRKPHHLRLLDGKQEVLDERRELLYTSLYLLIWGEAANLR
FMPECLCFIFHHMTFELNRMLERFIDELSAQSVRPTYCEPNGFLLRVVLPLYTIVKAEAESNNNGTAPHSWWRNYDDLN
EYFWSNRCFTQLQWPLQLDSNFLVEPWKEKVDRHTKQKVGKTGFVEQRSFWNVFRSFDRLWIGYILVLQACIVTLWHG
SGPPWVELQKRDSLARFLTIFITWAALRVLQALLDFGMQYSLISRETISIGVRMVLKPLVATGWVIIFTVFYRSMWAQRHR
DRSWSTSANNLLIRFLEAAAVFVLPEVLALLLFILPWVRNFVENSSWRVLNLLTWWFQSRTFVGRGLREGVVDNIKYTVF
WLLVLASKFAFSYFLQIKPLIKPTKEILQIRNITYKWHEFFAHGNRAAVVALWAPVILIYFMDTQIWYSIWSALVGALVGLFE
HLGEIRNVQQLKLRFQIFPSASQFILMPEEVSVTQKMWGGWRAKIKDIFKRFKLRYGWSSPYQKVDSKQIEAGRFSHIW
NKIVQIFREEDLISDRELELLEIPTASWNVSVFQWPSVLLANEILIALGHVTFHHGDDKAVWRKISSSEYRRCAVIESYESIK
HVIKRIVRDDTEEHQIFLAIFEEIDTSILQGRFTETFALYELPQIHKSILALLTVLLKRPTPKDLQKVVDALQNLYDVVLRDFPR
DAGVQEDIRARAAYPHNNREVLLFTEAVELPDTEDEPFFKQLQRLHTTLSTKEALLNVPRGLEARRRISFFSNSLFMTMPR
APQVDKMLAFSVLTPYYSEEVIYTKQQLREENEDGVSTLFYLQKIFPDDWKNFLERMGKEGLKEKDLWETDDAFQLRM
WASYRGQTLARTVRGMMYYERALELQAFLDTASEVEVLGYRELLDRASSMTSEGSFGRGSFGRLPQNISPSNSDSADSD
KKQQELATAAMKFTYVVTCQIYGSQKQKGDNRARDILHLMKTYKSLRIAYIDEVPAGRDSKQYYSVLVKYDPIVQAEVEIY
RIMLPGPMKLGEGKPENQNHAIIFTRGDALQTIDMNQEHYFEEAIKMRNLLQEFNQSRGVRKPTILGVREHVFTGSVSS
LAWFMSAQETSFVTLGQRVLANPLKVRMHYGHPDVFDRLWFLSRGGISKASRTINISEDIFAGFNCTLRGGTVTHHEYI
QAGKGRDVGLNQISLFEAKVASGNGEQVLSRDVYRLGHRLDFFRMLSFYYSTVGFFINNMMIVLTVYSFLYGRVYLALSG
VEASITSASNALNNSALTAILNQQLVVQLGLLTALPMVVENALEHGFTRAIWEFCTMQLQLASVFFTFSMGTRTHFFGRT
LLHGGAKYRATGRGFVVRHERFAENYRLYSRSHFVKAVELVILLTLYSAYGVATSTATYILVTITSWFLALSWLMAPFIFNPSG
FDWLKTVDDYDDFMNWLWYKGGVFVKAEQSWEVWWGEEQSHLRTTGLWGKLLEIVLDLRFFFFQYGIVYQLGIASK
NKSILVYLISWSYVFVAGLFHLVLSTANERYAAKRHGLYRLIQALLISLIVVILALLLVLTNLKIVDLLTSLLAFMPTGWAIIQICL
VLKTPFLENTRAWGTVVAVARLYELGMALVVMLPVALLSWLPGFQAMQTRILFNEAFSRGLQMTRIVAGKRS------------
----------------------------------------------------------------------------------------------------------------------------------------------
---------------------------------------------------------------------------------------------

>UHLI 2006658 Sphagnum recurvatum 1706

GATKALLYTEGSPRFPYDYVMPENRNLDVFDFLHYVFCFQKDNVANQREHLILLLASSQSRKGVLRDGHRTEGSAVNEA
AVDDVYERVLGNYMRWCDFLLKEPKAKKAKDATQQEKIYLIALHLLIWGEAANLRFMPECLCYIFHHMAHDMFELFRKE
EVEWSSQTAKPSEDGSRELCFLEQVITPIYQIVAAEAHNNGNGVASHSAWRNYDDFNEFFWQADCFDHLSWPWKEDA
AFFMKPKKRSYDDNDKTVQHAESAALLSQTRGRRVGKINFVEHRTGFHLFHSFYRLWIFFICMLQGLTIWAFCNQNFHL
RTIKKILSIGPTFAFLTFFQSVLDVLLMWGAYRSTRHHIVMRMLIRLVWFGALSGGVIFLYVKTLLEDRQGTGSTIWFRIFYL
VLGSYAILHLLIGMLSHIPWLRIRTAEWSKYRVIRFIKAVHQERYFVGRGMYERLWDYFRYVLFWVFVLFCKFSFSYHFQLL
PMVVPTRLIVELNNINYVWHDFVSKNNHNALTLLALWAPVVMIYFLDVQVWYTVTSALLGGLEGAKDRLGEIRDLTML
RKRFIDYPQALVQRLQPTNSSRASRQISLAEGAATDGKVIRSKQDAINAIKFAPIWNEVIKSLREEDLINNKEKELLIMPEQ
DVMQQNSWRIHWPLFLVANKVHVAVELAAGNKKNLELLWEKVNRVEYMANAVEEAFETLQPVLENLLNADGAQWV
RSLFGDIKEAIGSQAFVANFNLTNLREILKMTRKITKQLWRNKTAERVSKVEAALQRLQAVVMNGFLPQDIREGFERWN
RSEKPLFTNLNWPDRNGQKDAKRLFNLLTVQKINASKTLDTETIPRNLEAQRRLQFFTNSLFMHMPEAPTIRKMFSFCVF

TPYYAEDVMYDLKKLCEENKDGISILFYLQKIYPDEWQNFLERIGLTGRTVDTKVDEKNEEVILQLRLWASYRGQTLARTV
RGMMYYKRALELQAAQEGASTADVEEGLQLSGGLLERSAKYQAELKFTYVVTCQIFGEQKKQGAVQAADILYLMHKYDS
LRIAYIDVVETMKDKKVTKSYYSKLVKADPYGQDQEIYSIKLPGEVKLGEGKPENQNHAIIFTRGDAIQTIDMNQDNYLEE
ALKVRNLLAEFDREDISLRPPTILGVREHVFTGSVSSLAWFMSMQESSFVTLGQRVLARPLKVRMHYGHPDVFDRIFHIS
RGGISKASRVINLSEDIFAGFNSTLRQGNVTHHEYIQVGKGRDVGLNQIALFEAKVASGNGEQSLSRDVYRLGQLFDFFR
MLSFFYTSVGFYVTTMMTVLTLYVFLYGKAYLALSGVDASLQADNNIIQNAALQSALNTQFLVQIGIFTAVPMIMNLILEQ
GILRAIISFCTMQLQLASVFFTFSLGTRTHYFGRTILHGGAKYRSTGRGFVVTHIKFAENYRMYSRSHFTKAMEIIMLLIVYL
AYGAEDRKAVNFILLTFSSWFLALSWLFAPYIFNPSGFEWQKTVEDFEDWTKWLYYKGGVGVKIENSWEAWWFDEQTH
IRTNTSRFWEIILSLRFFIFQYGIVYHLHVDEHSTRLNVYGFSWLVLVVIVVIFKLFTFTRKSPKVQRLLRLFQTLLFMSIVTGII
VAVVLTPLTIGDVFAVGLALIPTGWGLLSVAIACRPAVKGLRLWESVQEIARAYDACMGMLVFIPIAFLSWFPFVSTFQTRL
VFNQAFSRGLEISLILAGNRPN----------------------------------------------------------------------------------------------------
--------------------------------------------------------------------------------------------------------------------------------------------
----------------------------------------------------------

>ZQRI 2012342 Timmia austriaca 1985

MDRPPRPAQRISRRVLGNWEKLVDKAVRTELHRAQSYNRDGRGRGNSGQRGERAPQSLAQQADIDAVLQAADEIGH
DNIQVARILAEHAFRLTQQLDPRSEGRGVLQFVTGLKSIIKQKQQARQGDVVDRSQDIRIIQVYYKHYREETGIDKLEEEAR
NRRPGFSTDLDPESQDQSFENLRKVYERSRILNDVVDALLKEAEPEDASRLQNTDEKRVMEEDAKKLKGYKAYNILPLKTS
MDPPGVLNPFDYFPEVVAATQVLQYTLDLPRFPSDFDLPEDRVLDIFDFLHYAFGFQKDNVANQREHIILLLASAQSRIGT
VDQSREGDGNKLADKVISDVHERILQNYVRWCHFLRREPQSKRAFTPPRRLCLTALYLLVWGEAANLRFMPECLCYIFHN
LADECFDLLERTYVERSKTVKPNADGTTEFSFLEQIITPVYDVVAAEAKSSQNGKVPHSHWRNYDDFNEYFWQPSCFLEL
SWPWRTDAGFFKTPQVKGAKKAPPQELNPQYEANPLVPPRRRSKHKVGKVHFVEHRSGFHIYHSFHRLWIFLVCMLQG
LTIWAFCSKNGNLNLHVRTIKRIMSVGPTYVIMKFIQSVFDVVFMWGAFKSTRLQTVARMLLRFLWFTCLSAAILFLYVKT
LEEDARNDGSGSWFRIYYILVSSYAGANVLFVFLLRIPFLQRQAAKCSNVYLFQFIKWLNQERYYVGRSMYERTRNYMKY
SIFWIFILACKFAFTMHFQIMPLVVPTRLIIGFDNIKYKWPDFVSDSNHNALTIASIWAPVVMIYFLDTQVWYTVVSALLG
GIEGARDKLGEIRTLDMLRKRFTNYPGAYVKHMQPPGNRFSSASPMQPDEPKKMMAMQNKRDAIRFQHIWNHVIKS
LREEDLINNREKVLLKMPPNLMFHSNGAPNNLIHWPLFLLANKVHIAVELAVDHKTDQSGLWEKVRGDEYMAYAVEET
FQSLEPLLTSVLNTEGGRWVGTIFGELRASIHSNSLREQFKLNRLRDVLEKLRDLTEHLGNDDVPERRVKATTAFYQLYEVV
MRDFFPADSRDRSDQWEYLQETLHNGLVFSDLNWPYKAGIKQAKRLNNLLTVQKIKDQEGKTKTLNTETIPHNLEARRR
LQFFTNSLFMHMPPAPPIRKMFSFCVFTPYYEEDVMYDMEKLSQENEDGITILFYLQKIYPDEWQNFLERIGLIENIVFREI
ENRKSKMHEELKIQLRLWASYRGQTLARTVRGMMYYKLALIIQGIQEGASGADLEEGIPPSLVEAHGSMDRSAINQAEL
KFTYVVTCQIYGEQKRKGKVQAADILYLMAKHDSLRIAFIDVVESSRKDKKPSYYSKLCKADRSDPTVYRDQEVYSIKLPGE
VKLGEGKPENQNHAIIFTRGDCIQTIDMNQDNFMEEAFKMRNLLEEFNQTHHGLHRPTILGVREHVFTGSVSSLAWFM
SMQESSFVTLGQRVLARPLKVRMHYGHPDVFDRVFHITRGGISKASRVINLSEDIFAGFNTTLRLGNVTHHEYIQVGKGR
DVGLNQIALFEAKVASGNGEQTLSRDLYRLGQLLDFPRMLSFFFTSVGFYVTTMMTVLTLYVFLYGKAYLALSGVDASLKR
NSEILQNPALEAALNTQFLFQIGIFTAVPMIVNLILEQGILKAIISFFTMQLQLASVFFTFSLGTRTHYFGRTILHGGAKYRST
GRGFVVTHIQFAENYRLYSRSHFTKALEIIMLLIVYLAYGAQNRSSVTFILLTFSSWFLAISWLFAPYIFNPSGFEWQKTVED
FDDWTNWLFYKGGVGVKTENSWEAWWFDEQDHIRSPRGRFWEIVLSLRFFLFQYGVVYSLSVTRGSDSILVYAYSWFV
LLGLVIIFKVFTVSQKASASFQLGVRLFQGLLFVCLIAGLVVAIVLSPLTIGDVFAVALALIPTGWGLLSIAIAVRPLLERMHV
WKSVREIARVYDACMGMIIFIPIAFLSWFPFISTFQTRLVFNQAFSRGLEISLILSGNR-------------------

>gZQRI 2068262 Timmia austriaca 1667

VLVDSEIKLDEGAIAAVHINMMSNYERWCKFLRRESMAKRAYSVHLRLFLTALYLLIWGEAANLRFLPECLCYIFHHMADE
LYDLLDKPVVERSRTFIPDSPHSFLDKIIKPIYEILAAEAKICAGGRAPHSAWRNYDDFNEFFWAPSCFELSWPWRPDAGF
FRKPKKIIYTEADRFEPMAPEQESPPMVGEPREKKVGKTHFVEHRTGFHIYHSFHRLWIFLVCMLQGLGIFAFCDRRLTVR
NVKLIMSVGPTFVIMKLIQSVMDVTLMIGAYSSTRLRNVSRMLIRFIWFTILSVVIVLLYVKTIEEEKSGSGTDTWFRAFYW
VLGIYGGLQLFLALLLRVPWFRIQAEKCSNFYVVQLIKWVHQERYYVGRNMYERTRDYFQYTLFWFVVGTCKFAFSYFLQ
IQPMVGPTRTIIGIQNVNYRWKDLISSSNHNALTLVALWAPVIMIYFLDTQVWYTVVSALVGGFDGARMHLGEIRSLDM
LRSRFSSLPGAFVKNLVPSRSGGHSQMDMNNPLSAVKPGNPKVDAIRFAPLWNEVIHSLREEDLINNRENEWLLMPDN
TISSGALGQQTLVQWPLFLLANKVYVAIDIAHENKQAYQDELWDRIKRDPYLEYAVEEAFVSLQSVLLDLLNEHGRAWVI
GIYQDIYDALGVSQVLNKFNFMELNSVLTRIAKLTEVLVLEPKKVDEIERHEEEVKMQESADRALQDLYEVVMRDFLLDSE
LRTKYEGDSLLQASKLDGRLFSDLTWPAGAAKLQVRRLNYILAIKDSALNVPVNLEARRRLQFFSNSLFMSMPQPPPVRK
MFSFSVFTPYFGEDVMYSTAQLEDKNVDGITILYYLQTIVPDEWRNFLERMIPGVEYNQLVHYTEADVTNIRELRLWASY
RGQTLARTVRGMMYYKKALVLQAQQEGASMAEDEEEGHDLEGNELAIVNVGTPRTPRGSLVRSARAQAELKFCYVVT
AQFYGKQKNSLVQADKDRAADILYLMHMNDSLRVAYIHEAKKMTDGKSITEYYSKLVKADPSGKDQEIFSIKLPGEVTLG
EDKPENQNHAIVFTRGEALQTIDMNQEHYLEETLKMRNLLEEFDSKTLGLRRPTILGVREHVFTGSVSSLAWFMSLQERS
FVTLGQRVLANPLKVRMHYGHPDVFDRIFHITRGGISKPSKQINLSEDIFAGFNSTLRQGNITHHEYIQCGKGRDVGLNQI
AAFEGKVASGNGEQSLSRDIYRLGQLFDFFRMCSFFFTSVGYYFTTMLTVLTIYVFLYGKVYLALSGVDEALKASNLLENTA
LQAALDTQFLLQIGVFTAVPMIVNFILEQGILRAVISFFTMQLQLCSVFFTFSLGTRSHYFGRTILHGGAKNKVTGRGFVVE
HIPFAENYRTYARSHFVKGMEIIMLLIVYLVYGAHDRTTASYILLTFSSWFLALSWLYAPYIFNPSGFEWQKTVIDFDDWTN
WLFHKGGIGDEGKKSWEVWWDEEQAHIQTPRGRFWEILLSTRFFIFQYGVVYALKVAGNDKTFWVYGYSWVVIVGVF
LLFKIFTFSQKASANFQLIVRLFQGVVFLAVVAGVSVAVVLTELTVGDVFASALAIIPTGWGLLSIAVAIRPIVKSCGLWKSVR
GIARLYDAAMGMILFIPIALLSWFPFVTTFQTRLVFNQAFSRGLEISVLLAGNNPNAAI-------------------------------------
------------------------------------------------------------------------------------------------------------------------------------
------------------------------------------------------------------------------------------------------------------------------------
----------------------

>AWOI 2009411 Diphyscium foliosum 1956

RSESYAVPQSLAAQTDIEAVMNVAEEISNENVEVARILYEYAYNLTQQMDPTNQGRGVLQFKTGLKTILMQKRSKHHIDR
SQDFKILTEYYGMYKETEDIPTLDEEEQKYRDGRSPDEDPEHQAWRSEKRKRFYEISSVLNKAANFLMAADPELAAKVRD
PSKKLEMDADAKKIEEFKPYNILPLESTGVTNPFQNFPEVSAATEALYTSEWRPPFPRFPADYSKAVGRDVLDLFDFLHYAF
GFQTDNVANQREHLIMLLANAESRLGVLAEESVLAHNAKLAEVAIDTVHTRILENYERWCQFLTKEPQSKRAMNPQKRL
CLTALYLLIWGEAANVRFLPECLCYIFHHMAEECFHLLDRDYVERSKLVKECEDGTIDYSFLEHIITPVYNIVAAEAKNSQNG
KAPHGSWRNYDDFNEYFWQPSCFEELGWPWREGAGFFRKKGEGKASSSRGDARPLLGSDTTRERREHRVGKIHFVEH
RSSLHLYHSFHRLWIFLVIMLQGLTIWAFCSKNGNLNLHVRTIKRIMSVGPTFAVMKLLKSILDVAFMWGAFRDTSKKIVT
RVLLRLGWFTGLSGGLLYLYVKTLQEDSRNPPVTPWFRLYYIVLGSYAGAQVLFAFILRLPWIRKQADKCSNVYIFQFIKW
MQEERYYVGRGMYERTSDYVKYSLFWIFVLACKFAFTMHFQIMPMVTPTRLIIGFDNITYKWYSFISKGNKNVFTIVSLW
APVVLIYILDVQVWYTVVSALLGGLEGARDKLGEVRSLGMLRKRFIDYPQAFVERLQPQRSSTVRAAADRSVDEIKAIKDK
EDATRFLPIWNAVINSLREEDLLSNRERDMLEMPSNSKMYPNGAQATLIRWPLFLLANKVYFAAGLAAENIHDGQDEL
WEKVTRDEYMGYAVQESFQTLEQLLLSVVSASAQRWVKDIFKVVKVNIENSAFVGLCNLKKLPDVLEIIRDMTEHLSQD
VENSGSRRKAITALNKLSKLVMKQTDGLLTSEASDRFSQWANYEKAVEEERLFNDLRWPDEAWRDQANRLQNLLKVHK
YKDDADGKQKTYNTKTVPYNLEARRRLEFFTNSLFMNMPIARPVSKMFSFCVFTPYYSEDVMYDLKKKGANKNVDKSK
KDEIKELDRENEDGITILFYLQKVYPDEWKNFLERLTKTEGELMRQVKEQDNGDLRLELRLWASYRGQTLARTVRGIMYY
KKALELQAEQERGSVPDLERGGSSSSVRAQRGSMLRTPQAQAELKFVYLVSCQIYGDQKKTGKPQALDILHLMKQNESL
RIAYVDEVTVETGAQNKTTTYYSKLVKVDKMDKGKDQLIYSVKLPGPFKVGEGKPENQNHAIIFSRGDAVQTIDMNQDN

YLEEALKVRNLLEEFDQPHHGLHRPSILGVREHVFTGSVSSLAWFMSMQESSFVTLGQRVLARPLKVRMHYGHPDIFDR
VFHLTRGGVSKASHGINLSEDIFAGFNTTLRQGNVTHHEYIQVGKGRDVGLNQIATFEAKVASGNGEQALSRDLYRLGQL
LDFPRMLSFFFTSVGFYVTTMMTVLTLYAFLYGKAYLALSGVDASLKKNNDILQNSALQSVLTSQFLFQIGLFTAVPMIVNL
VLEKGLLKAFMDFCTMQLQLASVFFTFSLGTRTHYFGRIILHGGAKYRPTGRGFVVRHIGFAENYRLFSRSHFTKAFEIIML
LIVYLAYGAQNRTSVTYILLTFSSWFLALSWLYAPYIFNPSGFEWQKAVEDFDDWTNWILYKGGVGVKSDSSWEAWWAE
EQEHLRTAAGKFWEFVLSLRFFFFQFGIVYSLNVTQGSTSILVYVYSWILLIVFGLIFKVFTISQKASTKKAKYHLCLRLFQALL
FLALIAGAIVAIIKSPLTLGDCFAVGLALVPTGWGVISIAIIFPSLVKSIGLWKSVREIARLYDATMGMIIFIPIAFLSWFPFFTTF
QTRLIFNQAFSRGLEIYKIVSGERKKT--------------------------------------------------------------------------------------

>AWOI 2012069 Diphyscium foliosum 1971

MASAGGSESASTPVQRRLSRTYTTGAPTELFDSEVVPSSLASIAPILRVANEIESARPRVAYLCRYHAFEKAHWIDPTSSGR
GVRQFKTALLQRLEKDSEPTLALRHRRSDAREIQSYYQQYYNDYVKALDGAEHSDRAQLAKAYQTAAVLFEVLKAVNRDK
TEEPPPEIIAAAADVEQKKEIYVPYNILPLDAAGASQAIMQLDEIKAGLESLRNVRGLPWPSVQETHPRSGDVDCLDWLQ
DMFGFQKDNVANQREHLILILANVHIRLLPRPEPMHKLDDRALNAVMNKLFKNYKSWCKFLGRKHNLWLPQIHQEIRQ
RKILYMGLYLLVWGEAANLRFMPECLCYIYHNMASELHGMLAGNVSMVTGDNMKPAYGGGDESFLSLVVKPIYDVISKE
TLKNKNGTAPHSAWRNYDDLNEYFWKIDCFRLGWPMRSEADFFDPSPICGPQLLLMRNVLKGRVFKSTSKAFFVEIRTF
WHLFRSYDRMWAFYILGLQASIVLAWNVDGHLHNAFNGTVIKQVLSIFITASILRLVQAFLDIIFSYYAFQSLKFLGVFRLFL
KLLTSAAWVIVLTVCYVHTWNNPQGLIKDIQSWLGKSWQSSYLYVAAVMVYLTPNFIGAFFFLFPMLRRWIESSNWRIV
RFLLWWSQPRLYIGRGMHESQCALFGYTFFWMLLIASKFTFSYFIQIEPLVVPTKAIMQQHSVTYTWHEFFPHASNNPG
ALISLWAPVVMVYFMDSQIWYAVYSTIFGGILGSFRRLGEIRTLGMLRSRFSSLPGAFNENLVPADQKEPRRGFSFRRNFE
KVSPQKNRLKAARFSQLWNEVITSFRQEDLISDKERDLMLVPYSSDPHLNVVQWPPFLLASKVPIALQMARQAAETGRV
ADLLRKIRNDEYMKSAVLECYESFKRVLKVLIVGEVETRVIEGLLNEVEVNISKETLVENFKLRELPVLSAKFIELLELLEMNH
TGQDAVESARDLAVLKLQDMYEVVTRDMMSETMRDIWESSHGAIARGQGRNSELFSSKGDEPAVLFPPPRREAWIEQI
KRLHLLLTERESAMDVPENLEARRRIAFFTNSLFMDMPQAPRVRNMLSFSVLTPYYKEDVVYSKEDLTTENEDGISVLFYL
QKIYPDEWSNFLQRVGLDTTTDPEGEIFGNDDLENKLREWASFRGQTLSRTVRGMMYYRRALELQAFLDMATEDELVD
GYKIITEATPEQKKSQRSTWSQLQAIADMKFTYIAACQNYGEQKRQSHHNATEILKLMLKNRSLRVAYIDEVEERQNEKSE
KVFYSVLVKAVDGLDQEIYRIKLPGAVRLGEGKPENQNHALIFTRGEGLQAIDMNQDNYLEEAFKMRNLLQEFHEPHGV
RPPTILGVREHIFTGSVSSLAWFMSNQETSFVTIGQRVLASPLKVRFHYGHPDVFDRLFHITRGGMSKASRVINLSEDIFA
GFNSILRRGNVTHHEYIQVGKGRDVGLNQISLFEAKIANGNGEQVLSRDIYRLGHRFDFFRMLSCYFTTVGYYFSTMIVVL
TVYVFLYGRIYLALSGVDNSLVHSANNKALTAALASQSLVQLGLLMALPMVMEIGLERGFRTAMSDFLTMQLQLASVFFT
FSLGTKTHYFGRTVLHGGAKYRATGRGFVVRHERFAENYRLYSRSHFTKATELLLLLVVYTIYVSQSASGAVTYILITFSMWF
LVATWLFAPFLFNPSGFEWQKIVEDWDDWSKWINNRGGIGVEGNKSWESWWDEEQEHLKYSGVRGRCIEIILSLRFFL
YQYGIVYHLNIVESSNGLSISGCNSNNSFIFLFLTPRRWITFTNCHVGFCCEYCQVYGLSWLVILAVLTVLKIVSMGRDKFSA
DFQLMFRLLKALLFIGSISVIAVLHVKQFTVSDLFASILAFIPTGWALLQIAQACKPIVIKAGFWESVKSLARGYESMMGLLL
FTPIAVLSWFPFVSEFQTRLLFNQAFSRGLQISRILAGRKKL-------------------------------------------------------------------------
----

>AWOI 2014174 Diphyscium foliosum 1983

REKKLIRRRVLAQWERIVGKAIEAAEERTLLQWQDQSGEYGDTSSVPQILQQQKKNIDDILQTAREVEQKYPQVARILLEY
AYALSQNLDPRSESRGVLQFKTGLLSVIKQKRGEFLREQKLGDKVDRSEDVYIIQEFYKDLKRNLDQLEDEDWLRQQSQYS
HITPETWTELKRKVYVTAQILNEVVDFLTRDNPDYRRDQEFDPNLKVDLEKTAKKVDDFKPYNILPFEAPGVVNAFQNSP
EVMAAIRALELNLPDVYAFADDFKMPTMRNLDIFDFLQYSFGFQADNVSNQREHLILLLANSQSRLGDLVKRDPGSIPKL
DDGAIFRVHTTLLDNYERWCKFLRKDSMTSRRSESRVHSTQLRLILSALYLLIWGEAANVRFLPECLCYIFHHMAHETYDL
LMEPFSHKSVIIVPDSSHSFLETIIKPIHGVVAAEAKVCGHGKAPHSRWRNYDDFNEFFWAPSCFELGWPWRSSAGFFV
KPKQVVDKKADKYESRKPPEEVPLLPGQRREKKAGKSHFVEHRSGFHLYHSFHRLWIFLVCMLQGLAILAFCDGKLRGGS
IKYIFSVGPTFVSMKFIQSVLDVLLLIGAYRSTRARTISRIWIRFFWFASLSAAITLLYIKTIQEQDSGSGLNSSFRLYCFVLGIYG
GLQLFIALLLRLPWFRVQADKCSNFGLVQFLKWVHQERYYVGRGLYERTGDYFKYILFWLIIGACKFSFSYFLQIKPMVSPT
RIIIDLTDINYRWRDLVSKSNHNALTLVSLWAPVVMIYFLDLQVWYTVVSALVGGLDGARMGLGEIRSLHMFRDRFSSLP
TAFVKRLRASQLGMRESYSDQDPKQKVDARNFAPVWNDVIKSLREEDLINNREKEWLLMPDNILGPHSLVQWPLFLLA
NKVYYVACEIVNEHKNTTQMELWERLGRDPYMMFAVQEAYYTLKIILENLLNEHGRLWVTAIYKQMDEALLYNQLVLKFH
LDKRQLTSLLEKAAKLTDALMVEAKKVDDTEKQKEETKKAQMNVVRVLLDFHEVVMRDFIADPAIRLEIEQNRANLAAK
QDGGLFNEVTWPTGDSKLQVRRLHQILTIKDSALKVPVNLEARRRLEFFSNSLFMSMPESPPVRKMFSFSVFTPYYAEDV
IYSPAKLASENKDGISMMYYLRTIVPDEWDNFLERMFPTQENRGELKEFLKTIFPREYKTQEKPRDPQELNSFVLMQLRL
WASYRGQTLARTVRGMMYYKRALVLQAQQEGAPPSEDLEQGLQHSRVSTVQESGALNARAQAELKFLYVVSAQIYGE
QKEGAKGVEGKERATDISYLMQKNDSLRIAYIHKGKVKRGENEVTEYYSKLMKADPSGHDQEIYSIKLPGEVILGEGKPEN
QNHAIVFTRGEALQTIDMNQEHYLEETFKMRNLLEEFDSKNYGLRNPTILGVREHVFTGSVSSLAWFMSLQERSFVTLG
QRVLAKPLKVRMHYGHPDVFDRLFHITRGGISKASKQINLSEDIFAGFNSTLRQGNVTHHEYIQCGKGRDVGLNQIAAFE
GKVASGNGEQTLSRDIYRLGQLFDFFRMLSFFFTTVGYYFTTMLTVLTVYAFLYGKVYLALSGVDAALKAKGLSNNVALQS
ALDTQFLLQIGVFTAVPMIMNFILEEGVLRAIISFCTMQLQLSSVFFTFSLGTRTHYFGRTILHGGAKYMSTGRGFVVEHIP
FAENYRLYSRSHFVKALEIIMLLIIYLAYGAPERTTVTYILLTFSSWFLAISWLFAPYIFNPSGFEWEKIVTDFEDWTNWLFHK
GGIGDEGKKSWEVWWLEEQEHIQTPRGRFWEILLSLRFFIIQYGVFYALNVVGHDKNFRVYGFSWCALAGIVLIFKVFSF
NQKASANFQLIVRLFQGVVFLAIIGGSVAVALTSLTIGDVFASALSLIPTGWGLLSIAIALRPVMKRIGLWKSIRAIARLYEAF
MGALVFIPIALLSWFPFVSTFQTRLVFNQAFSRGLEISQLLAGTNPN----------------------------------------------------------
--

>AWOI 2073991 Diphyscium foliosum 1978

MATIPRQPQRISKRVLNKWERLVYKARMVAERRATAEEEAQGPGAGAAANTTVPQSLLQQTNIDAILQTADDLQQENP
KVARILCEYAYSLSQNLDPNSDGRGVLQFKTGLLSIIKQKRSKKEGEKIDRSQDIHIIQEFYKDYREKNHLDQLEEEDRMRR
QSDSYDEDSTTTEQRAELQRKVYQTARILNEVIDALKKENPDEDLDPELKRIMEKDAEKVRGFKAYNILPLETPGVANVFQ
NFPEVVGATRALEYNTSGLPEFPEDFEMPKERHLDVFDFLEYAFGFQVDNAANQREHLILLLSNSQSRMGVLVDTESKLD
EGAIAYVHEKMMKNYGRWCEFLRRESMAKRAYSMQLRVFLTALFLLIWGEAANLRFLPECLCYIFHHMADEMYDLLDR
PQVERSRTFLPDSPHSFLDRIIKPIHTILAAEAKICADGRAPYSAWRNYDDFNEFFWAPSCFELSWPWRPDAGFFRKPKK
VYYTEADRSEPVKPEEDPILNLGEKRERKVGKTHFVEHRTGFHIYHSFHRLWIFLVCMLQALSIFAFCDRKLTVRNIKLIMS
VGPTFVLMKFLQSVLDVTLLIGAYRSTRARNVSRMLLRFIWFTILSAIIVLLYVKTIEEANSGSGSDTYFKAFYWLLGIYGGL
QLFFALLLRVPWFRIQADKCESLYVVMLIKWVHQERYYVGRNMYERTRDYFMYTLFWFIVGTCKFAFSYFLQIQPMVGP
TRTIIGLRNINYRWKDLISGSNHNALTLVSLWAPVVLIYFLDTQVWYTVVSALVGGLEGARMRLGEIRSLDMLRTRFSTLP
EAFVKHLVPPRSGGNPLLSTSVPSGALKPENPKVDAIRFAPLWNEIITSLREEDLINNREKEWLLMPDNKATSSALSQHTL
VQWPLFLLANKVYVGIDIVNENRQSPQDELWDRVKRDPYLEYAVQEAFATLQTVLMELLNENGRAWVTGIYEDINAAVI
DNVFLSSFNLLELSNLLNRTAKLTEILASVPKKTDESARQEEETKMQESAVRALVDLYEVVMRDFVADPDLRVKYESDTTL
QASKQDGRLFNELTWPTGQSKTQVNRLNYILTIKDSALNVPVNLEARRRLQFFTNSLFMSMPEPPPVRKMFSFSVFTSYY
NEEVIYTQTQLEVKNVDGISMLYYLQKIFPDEWKNFLERMFPGMDYNQLAHYTETDVTNTQELRLWASYRGQTLARTV

RGMMYYQKALVLQAQQEGASIAEDEELGTDQEGNELAMVTVSTPRTPRGSLVRSAKAQAELKFCYVVSAQNYGKQKN
STEQADKDTAADILYLMQMNDSLRIAYIHEAKKIIDGKPHTDYYSKLIKADPSGKDQEIYSIKLPGEVILGEGKPENQNHAII
FTRGEALQTIDMNQEHYLEETLKMRNLLQEFDSKSSGLRRPTILGIREHVFTGSVSSLAWFMSLQERSFVTLGQRVLARPL
KVRMHYGHPDVFDRIFHITRGGISKASKQINLSEDIFAGFNSTLRQGNITHHEYIQCGKGRDVGLNQIASFEGKVASGNG
EQTLGRDVYRLGQLFDFFRMCSFFFTSVGFYFTTMLTVLTVYVFLYGKVYLALSGVDEALKSNSLLDNSALQSALDTQFLLQ
IGIFTAVPMIMNFILEQGVTRAIISFCTMQLQLSSVFFTFSLGTRTHFFGRTILHGGAKYKDPGRGFVVEHIPFAENYRLYSR
SHFVKGMEIIVLLIVYLVYGSHDRTAASFILLTFSSWFLALSWLYAPYIFNPSGFEWQKTVKDFDDWTNWLFHKGGIGDEG
KKSWEVWWVEEQAHIQTTRGRFWEVLLSTRFFIFQYGVCYALNAAGGNKSFAVYGYSWAVLVGFFLLFKIFTFSQKASA
NFQLIVRLFQGIVFLAVVAGVSVAVVLTRLTVGDVFASVLAFIPTGWGLLSIAIAIRPIMKWVGLWKSIRGIARLYDAAMG
MILFIPIALLSWFPFVSTFQTRLVFNQAFSRGLEISVLLAGDNPNAAL-------------------------------------------------------------
-------

>DHWX 2000693 Fontinalis antipyretica 1731

LDEVRAAVEALRNVRGLPWLTDKEPHSRAGDLDCLDWLQDMFGFQKDNVANQREHLILMLANVHNRLLPRPEPMHK
LDDRALNAVMNKLFKNYRSWCKFLGRKNNLWLPQIHQEVRQRKILYMGLYLLVWGEAANLRFMPECLCYIYHHMASEL
HGMLAGNVSMVTGDNMKPAYGGEDESFLTLIVTPIYKVISRETLRNRNGTAPHSAWRNYDDLNEYFWKGDCFRMGW
PMRPDAYFFVPAQILSIRSHWPTGKDDQSSSKSFFVEIRSFWHLFRSFDRLWAFYILGLQAMIVLAWNVGPNLQNAFTG
AVIKQILSIFITASILRLIQAFLDIVFGYHAFRSIKLLGVLRLVLKLLTSAAWVIVLTISYARTWVDPQGLIGEIQKWLGKSLESSY
LYVAVVVVYLIPNFVGAFFFLFPMIRRWIESSNWTMVRVLLWWSQPRLYIGRGMHESQMALLGYSFFWVLLIVSKFAFS
YFIQIEPLVGPTKAIMQQNSVTYTWHEFFPNARNNPGALISLWAPVIMVYFMDSQIWYAVFSTIFGGISGSFRRLGEIRTL
GMLRSRFSSLPGAFNESLVPDDGKRARRVFSFSRDFEKIAPSKDRLKAARFSQLWNEVITSFRQEDLISDKERDLMLVPYS
SDPHLKLVQWPPFLLASKVPVALQMAKQAAETGRAADLLRKIKTDEYMKCAVTECYESLKRVLKRYIVGEVEIRVIDGLFD
EVDVNVEKETLLDNFKLGELPVLSAKFIELLELLERNHAGQEAIDSARDLAVLKLQDMYEVVTRDMMSDSMRDSWDSSL
GALAGGQGRKSELFSAKGDEPAKVVFPLSRNEAWTEQIKRLHLLLTERESGMDVPENLEARRRIAFFTNSLFMSMPHAP
RVRNMLSFSVLTPYFKEDVVYSKENLMKENEDGISVLFYLQKIYPDEWNNFLQRIGLESSEDPEAQIFGCNGLEDKLREW
ASFRGQTLSRTVRGMMYYRRALELQAFLDMATEDELVDGYKVLTDATPEQKKSQRSTWSQLQAIADMKFTYVAACQM
YGEQKRRGHHSATEILKLMLNNLSLRVAYIDEVEERPNEKPSKVYYSVLVKAINGLDQEIYRIKLPGIVRLGEGKPENQNHA
VIFTRGEGLQTIDMNQDNYLEEAFKMRNLLQEFHEPHGVRPPTILGVREHIFTGSVSSLAWFMSNQETSFVTIGQRVLAS
PLKVRFHYGHPDVFDRLFHITRGGMSKASRVINLSEDIFAGFNSILRRGNVTHHEYIQVGKGRDVGLNQISLFEAKIACGN
GEQALSRDIYRLGHRFDFFRMLSCYFTTVGYYLSTMIVVLTVYVFLYGRIYLALSGVDDSLVYSANNKALTAALASQALVQL
GLLMALPMVMEIGLERGFRTALSDFLTMQLQLASVFFTFSLGTKTHYFGRTILHGGAKYRATGRGFVVRHERFADNYRLY
SRSHFTKAIELFLLLIVYSLYVSRSAKGAVTYILITFSMWFLVASWLFAPFLFNPSGFEWQKIVEDWDDWNKWMSNRGGI
GVEGSKSWESWWDEEQEHLNYTGFFGRCVESLLSIRFFLYQYGIVYHLNITRSSKDLSISVYGLSWLVIVVVLTVLKIVSMG
RDKFSADFQLLFRLLKASLFIGSVSVIAILHVKSFTVGDLFACILAFIPTGWALLQIFQACKPVVIRLGFWKSVKALARGYEYL
MGLLLFTPIAILSWFPFVSEFQTRLLFNQAFSRGLQISRILAGRKKL---------------------------------------------------------------
------------------------------------------------------------------------------------------------------------------------------------------------
----------------------------------------------------------------------------------------------------------------------

>HRWG 2005492 Buxbaumia aphylla 1909

GASELFDSEVVPSSLASIAPVLRVANEIESSRPRVAYLCRCHAFEKAHRIDPTSSGRGVRQFKTALLQRLEKDDAQTIGLRH
RRTDAREIQSYYQQYYNDYVKALDGAEHADRAQLAKAYQTASVLFEVLKTANRDKTEDPPPEIIAAAADVEQKKEIYVSY

NVLPLDSAGASQAIMQLDEIRAALESLRNVRGLPWTFVEESRPRSGDVDCFDWLQYMFGFQKDNVANQREHLILILAN
VHIRLLPRPEPMHKLDDRALNTVMTKLFKNYKSWCKFLGRKHNLWLPQIHQEIRQRKILYMGLYLLVWGEAANLRFMP
ECLCYIFHHMASELHGMLAGNVSMVTGDNMKPAYGGEEESFLRKVIQPIYNVISKETVINKNGTAPHSAWRNYDDLNE
YFWKTGCFRLGWMRPESDFFELSPSCDQNFMRNAVKGSSITPKRKQFFVEIRTFWHVFRSFDRMWAFYILGLQALIVLA
WNANGNFQNILSGSVFKKLLSIFITASLLRFIQAILDITLSYHAMYSLKMLGIIRLILKLLISAAWVIVLSVCYVHTWTNPQGL
IKDIQTLLGKKWESSYLYIAAVLLYLVPNLVGAFFFVFPMLRRWIESSNWRIVRFLLWWSQPRLYIGRGMHESQLALFGYSF
FWVLLLGSKFCFSYFIQIAPLVDPTRAIMMQHSVTYTWHEFFPHAKNNFGALISLWSPVIMVYFMDSQIWYSVYSTIFGG
VLGSFRRLGEIRTLGMLRSRFSSLPGAFNENLLPADRKKTAHRFSFKQNSDMASSTNTKLKSAGFSQLWNEVITSFRHEDL
ISDKERNLMLVPYSSDPHLNLVQWPPFLLASKVPIAIQMAKQAAESGRSADLLRKIKNDEYLKSAVKECYESFKRVLKVLVI
GKIEISVIEGLLDAVEFSIGKETLLDSFNLRELPVLSSKFIDLLELLVKDHSGQEALESAGDLAVLKLQDMYEVVTRDMMSEA
TRASWELSHGAISGGQGRNSELFSSKGDQPAIVFPPPRKEAWIEQIKRLHLLMTERESGMDVPENLEARRRIAFFTNSLF
MDMPRAPRVRNMLSFSVLTPYYKEDVVYSKFDLMTENEDGISVLYYLQKIYPDEWSNFLQRVGLEGTADPEAEIFGNED
LEDSLREWASFRGQTLSRTVRGMMYYRKALELQAFLDMATEEELVAGYKIIADATPEQRKSQRSTWSQLQAIADMKFTY
VAACQNYGEQKRQSHHNASEILKLMLKYPGLRVAFVDEVEERQNEITNKVFYSVLVKAVDGLDQEIYRIKLPGPVRLGEG
KPENQNHAIVFTRGEGLQAIDMNQDNYLEEAFKMRNLLQEFHEPHGVRPPTILGVREHVFTGSVSSLAWFMSNQETSF
VTIGQRVLATPLKVRFHYGHPDVFDRLFHITRGGMSKASRVINLSEDIFAGFNSILRRGNVTHHEYIQVGKGRDVGLNQIS
LFEAKIANGNGEQVLSRDIYRLGHRFDYFRMLSCYFTTVGYYVSSLIVVLTVYIFLYGRIYLALSGVDNSLVHSANNKALTAA
LASQSLVQLGLLMALPMVMEIGLERGFRTALSDFLTMQLQLASVFFTFSLGTKTHYFGRTILHGGAKYRATGRGFVVRHE
RFAENYRLYSRSHFTKGAELLLLLIVYSIYVSKSASGAVTYTLITFSMWFLVATWLFAPFLFNPSGFEWQKIVEDWDDWNK
WLSNRGGIGVEGNKSWESWWDEEQEHLTHTGLGGRLIEVLLSSRFFLYQYGIIYHLNIVETSNDLSIGVYGLSWLVIVAVL
TVLKIVSMGRDKFSADFQLMFRLLKALVFIGSISVLAVLHIKQFTAGDLFASILAFLPTGWALLQIAQACRPIVIQLGFWESV
KSLARGYEFLLGLLLFTPIAVLSWFPFISEFQTRLLFNQAFSRGLQISRILAGRKKV---------------------------------------------
-----------------------------------------------------------------------------------------

>IGUH 2003398 Schwetschkeopsis fabronia 1961

MSSGGRLEFGAADLPRRLSRMYTAGGLTEVFDSEVVPSSLASIAPILRVANEIEGSSARVAYLCRYHAFEKAHRIDPTSSGR
GVRQFKTALLQRLERDSEPTLALRHRRSDAREIQSYYQNYYNDYVKALDGAEHSDRAQLARAYQTAYVLFEVLKAVNRDK
AEEPPPEIIAAAADVEQKKEIYVPYNILPLDAAGASQAIMQLDEVRAAVEALRNVRGLPWLTEKEPNSRAGDLDCLEWL
QDMFGFQKDNVANQREHLILMLANVHIRLLPRPEPMHKLDDRALNAVMNKLFKNYKSWCKFLGRKNNLWLPQIHQE
VRQRKILYMGLYLLVWGEAANLRFMPECLCYIYHHMASELHGMLAGNVSMVTGDNMRPAYGGEDESFLTFIVTPIYEVI
SRETLKNRNGTAPHSAWRNYDDLNEYFWKGDCFRIGWPMRPDADFFFPARTSSSTNDIDGKDYQLSSKSFFVELRSFW
HLFRSFDRLWAFYILGLQAMIVLAWNVGPNLQNALTGAVLKQLLSIFITASILRLIQGKVSELFTCVFSCCQFFELLAELFTLL
FDVAFLDIVFCYHAFRSIKLLGVLRLVLKLFTSAAWVIVLTISYARTWVNPQGLIGEIQKWLGKSLESSYLYVVAVVVYLIPNFI
GALFFLFPMIRRWIESSNWTVVRVLLWWSQPRLYIGRGMHESQIALLGYTFFWVLLIASKFAFSYFIQIEPLVAPTKAIMQ
QSSVIYTWHEFFPNARNNPGALISLWAPVIMVYFMDSQIWYAVFSTIFGGISGSFRRLGEIRTLGMLRSRFSSLPGAFNES
LVPDDGKRARKGFSFSRDFEKISPSKDRSKAARFSQLWNEVITSFRQEDLISDKERDLMLVPYSSDPHLKLVQWPPFLLAS
KVPIALQMAKQAAETGRAADLLRKIKIDEYMKCAVTECYESLKRVLKRFIVGEVEIRVIDGLFDEVDVNVEKETLLDNFKLG
ELPVLSAKFIELLELLEKNHAGQEAIDSARDLAVLKLQDMYEVVTRDMMSDSMRDSWDSSLGALAGGQGRKSELFSAK
GDEPAKVVFPLSRSEAWIEQIKRLHLLLTERESAMDVPENLEARRRIAFFTNSLFMNMPHAPRVRNMLSFSVLTPYFKED
VVYSKENLMKENEDGISVLFYLQKIYPDEWSNFLQRIGLESSEDPEAQIFGCNDFEDKLREWASFRGQTLSRTVRGMMY
YRRALELQAFLDMATEDELVDGYKVLTGATPEQKKSQRSTWSQLQAIADMKFTYVAACQMYGEQKRQGHHSATEILKL
MLNNLSLRVAYIDEVEERQNESKVYYSVLVKAINGLDQEIYRIKLPGVVRLGEGKPENQNHAVIFTRGEGLQTIDMNQDN
YLEEAFKMRNLLQEFHEPHGVRPPTILGVREHIFTGSVSSLAWFMSNQETSFVTIGQRVLAIPLKVRFHYGHPDVFDRLF

HITRGGMSKASRVINLSEDIFAGFNSILRRGNVTHHEYIQVGKGRDVGLNQISLFEAKIACGNGEQALSRDVYRLGHRFDF
FRMLSCYFTTVGYYLSTMIVVLTVYVFLYGRIYLALSGVDDSLVHSANNKALTAALASQALVQLGLLMALPMVMEIGLERG
FRTALSDFLTMQLQLASVFFTFSLGTKTHYFGRTVLHGGAKYRATGRGFVVRHERFADNYRLYSRSHFTKAIELFLLLIVYTL
YVSRSAKGAATYILITFSMWFLVASWLFAPFLFNPSGFEWQKIVEDWDDWNKWMSNRGGIGVEGSKSWESWWDEE
QEHLNFTGIFGRFVESLLSIRFFLYQYGIVYHLNIARSSKDLSISVYGLSWLVIVAVLTVLKIVSMGRDKFSADFQLLFRLLKAL
LFIGSVSVIAILHVKSFTVGDLFACILAFIPTGWALLQILQACKPVVVRLGFWKSVKALARGYEYLMGLLLFTPIAILSWFPFV
SEFQTRLLFNQAFSRGLQISRILAGRKTL--------------------------------------------------------------------------------

>IGUH 2003399 Schwetschkeopsis fabronia 1932

MASGGKPESGVTLLSRRIPRTYTAGGLTEVFDSEVVPSSLASIAPILRVANEIEASSPRVAYLCRYHAFEKAHRIDSTSSGRG
VRQFKTALLQRLERDSEPTLALRHRRSDAREIQSYYQNYYNDYVKSLDGAEHSDRAQLARAYQTAAVLFDVLKAVNRDKA
EEPPPEIIAAAADVEQKKEIYVPYNVLPLDAAGSSQAIMQLDEVRAAVEALRNVRGLPWQTVKESHPRAGDLDCLDWL
QDMFGFQKDNVANQREHLTLMLANVHIRLLPRPDPMHKLDDRALNAVLNKLFKNYKSWCKFLGRKNNLWLPQIHQE
VRQRKILYMGLYLLVWGESANLRFMPECLCYIYHHMACELHGMLAGNVSMVTGDNMKPAYGGEDESFLTLVVTPIYKVI
SKESLKNRNGTAPHSAWRNYDDLNEYFWKVDCFRMGWPMRPDADFFVPDQTFLNTTVVTNEKVYQSTSKSFFVEIRT
FWHLFRSFDRLWAFYILGLQAMIVLAWNVGRNLEDAFDGTVVKQVLSIFITASILRLIQAFLDIVFGYHAFRSIRLFGEFRLF
LKLLTSAAWVILLTICYVRTWVNPQGLIAEIQKWFGESLETSYLYIAAVLLYLIPNFIGACFFLFPMIRRWIESSKWTVVLLW
WSQPRLYIGRGMHESQLALLGYTIFWILLISCKFSFSYFFQIEPLVTPTRAIMKQTSLSYTWHEFFPNARNNPGALISLWAPI
IMVYFMDCQIWYAIFSTIFGGISGSFRRLGEIRTLGMLRSRFSSLPGAFNESLVPDDGKRARKAFSFSRQFEKVTPSKDRSK
NARFSQLWNEVITSFRQEDLISDKEKDLMLVPYSSDPHLKLVQWPPFLLASKVPIALQMAKQAAETGRVADFLRKIKNDE
YMKCAVTECYESLKRVLMRFIVGDIETRLIDGLFDEVDVNVEKDTLLDNFKLGELPVLSAKFIELLELLEMNHTDKEAVDN
ARDLAVLKLQDMYEVVTRDMMSDKMRDSWDSSFGALVGGQGWKSELFSSKGDEPAKILFPTPRSEAWIEQIKRLHLLL
TERESGMDVPENLEARRRIAFFTNSLFMNMPQAPRVRNMLSFSVLTPYYKEDVVYSKENLMKENEDGISVLFYLQKIYP
DEWSNFLQRIGLESVEDPEAQIFVNTDLEDKLREWASFRGQTLARTVRGMMYYRRALELQTFLDMATENELVDGYKVLT
DASIEQKKSQRSTWSQLQAIADMKFTYVAACQMYGEQKRQGHHHATEILRLMLAYPSLRVAYIDEVEEPEGSKSSKVYYS
VLVKAVNGLDQEIYRIKLPGTVSLGEGKPENQNHAVIFTRGEGLQAIDMNQDNYLEEALKMRNLLQEFHEPHGVRSPTIL
GVREHVFTGSVSSLAWFMSNQETSFVTIGQRVLASPLKVRFHYGHPDVFDRLFHITRGGMSKASRVINLSEDIFAGFNSIL
RRGNVTHHEYIQVGKGRDVGLNQISLFEAKIACGNGEQALSRDIYRLGHRFDFFRMLSCYFTTVGYYFSTMIVVLTVYVFL
YGRIYLALSGVDDSLVHSANNKPLTAALASQALVQLGLLMALPMVMEIGLERGFRTALSDFLTMQLQLASVFFTFSLGTKT
HYFGRTILHGGAKYRATGRGFVVRHERFADNYRLYSRSHFTKAIELFLLLIVYTLYVSESSRGAVTYILITFSMWFLVASWLFA
PFLFNPSGFEWQKIVEDWDDWNKWMSNRGGIGVEGSKSWESWWDEEHEHLNYTGFIGRVVESILSLRFFLYQYGIVY
HLNIARSSNDLSISVYGLSWLFILAVLTILKIVSMGRDKFSADFQLMFRLLKASLFIGSVSVIAILHVKNLTVGDLFASILAFIPT
GWALLQIAQASKPVVIRLGFWSSVKSLARGYEYSMGLLLFTPIAVLSWFPFVSEFQTRLLFNQAFSRGLQISRILAGRKKL--
-------------------------------------------------------------------------------------------------------------

>IGUH 2003401 Schwetschkeopsis fabronia 1964

MSSGGRLEFGAADLPRRLSRMYTAGGLTEVFDSEVVPSSLASIAPILRVANEIEGSSARVAYLCRYHAFEKAHRIDPTSSGR
GVRQFKTALLQRLERDSEPTLALRHRRSDAREIQSYYQNYYNDYVKALDGAEHSDRAQLARAYQTAYVLFEVLKAVNRDK
AEEPPPEIIAAAADVEQKKEIYVPYNILPLDAAGASQAIMQLDEVRAAVEALRNVRGLPWLTEKEPNSRAGDLDCLEWL
QDMFGFQKDNVANQREHLILMLANVHIRLLPRPEPMHKLDDRALNAVMNKLFKNYKSWCKFLGRKNNLWLPQIHQE
VRQRKILYMGLYLLVWGEAANLRFMPECLCYIYHHMASELHGMLAGNVSMVTGDNMRPAYGGEDESFLTFIVTPIYEVI

SRETLKNRNGTAPHSAWRNYDDLNEYFWKGDCFRIGWPMRPDADFFFPARTSSSTNDIDGKDYQLSSKSFFVELRSFW
HLFRSFDRLWAFYILGLQAMIVLAWNVGPNLQNALTGAVLKQLLSIFITASILRLIQGKVSELFTCVFSCCQFFELLAELFTLL
FDVAFLDIVFCYHAFRSIKLLGVLRLVLKLFTSAAWVIVLTISYARTWVNPQGLIGEIQKWLGKSLESSYLYVVAVVVYLIPNFI
GALFFLFPMIRRWIESSNWTVVRVLLWWSQPRLYIGRGMHESQIALLGYTFFWVLLIASKFAFSYFIQIEPLVAPTKAIMQ
QSSVIYTWHEFFPNARNNPGALISLWAPVIMVYFMDSQIWYAVFSTIFGGISGSFRRLGEIRTLGMLRSRFSSLPGAFNES
LVPDDGKRARKGFSFSRDFEKISPSKDRSKAARFSQLWNEVITSFRQEDLISDKERDLMLVPYSSDPHLKLVQWPPFLLAS
KVPIALQMAKQAAETGRAADLLRKIKIDEYMKCAVTECYESLKRVLKRFIVGEVEIRVIDGLFDEVDVNVEKETLLDNFKLG
ELPVLSAKFIELLELLEKNHAGQEAIDSARDLAVLKLQDMYEVVTRDMMSDSMRDSWDSSLGALAGGQGRKSELFSAK
GDEPAKVVFPLSRSEAWIEQIKRLHLLLTERESAMDVPENLEARRRIAFFTNSLFMNMPHAPRVRNMLSFSVLTPYFKED
VVYSKENLMKENEDGISVLFYLQKIYPDEWSNFLQRIGLESSEDPEAQIFGCNDFEDKLREWASFRGQTLSRTVRGMMY
YRRALELQAFLDMATEDELVDGYKVLTGATPEQKKSQRSTWSQLQAIADMKFTYVAACQMYGEQKRQGHHSATEILKL
MLNNLSLRVAYIDEVEERQNESKVYYSVLVKAINGLDQEIYRIKLPGVVRLGEGKPENQNHAVIFTRGEGLQTIDMNQDN
YLEEAFKMRNLLQEFHEPHGVRPPTILGVREHIFTGSVSSLAWFMSNQETSFVTIGQRVLAIPLKVRFHYGHPDVFDRLF
HITRGGMSKASRVINLSEDIFAGFNSILRRGNVTHHEYIQVGKGRDVGLNQISLFEAKIACGNGEQALSRDVYRLGHRFDF
FRMLSCYFTTVGYYLSTMFAQIVVLTVYVFLYGRIYLALSGVDDSLVHSANNKALTAALASQALVQLGLLMALPMVMEIGL
ERGFRTALSDFLTMQLQLASVFFTFSLGTKTHYFGRTVLHGGAKYRATGRGFVVRHERFADNYRLYSRSHFTKAIELFLLLIV
YTLYVSRSAKGAATYILITFSMWFLVASWLFAPFLFNPSGFEWQKIVEDWDDWNKWMSNRGGIGVEGSKSWESWWD
EEQEHLNFTGIFGRFVESLLSIRFFLYQYGIVYHLNIARSSKDLSISVYGLSWLVIVAVLTVLKIVSMGRDKFSADFQLLFRLLK
ALLFIGSVSVIAILHVKSFTVGDLFACILAFIPTGWALLQILQACKPVVVRLGFWKSVKALARGYEYLMGLLLFTPIAILSWFP
FVSEFQTRLLFNQAFSRGLQISRILAGRKTL---------------------------------------------------------------------------------

>IGUH 2003402 Schwetschkeopsis fabronia 1997

LEFGAADLPRRLSRMYTAGGLTEVFDSEVVPSSLASIAPILRVANEIEGSSARVAYLCRYHAFEKAHRIDPTSSGRGVRQFK
TALLQRLERDSEPTLALRHRRSDAREIQSYYQNYYNDYVKALDGAEHSDRAQLARAYQTAYVLFEVLKAVNRDKAEEPPP
EIIAAAADVEQKKEIYVPYNILPLDAAGASQAIMQLDEVRAAVEALRNVRGLPWLTEKEPNSRAGDLDCLEWLQDMFGF
QKDNVANQREHLILMLANVHIRLLPRPEPMHKLDDRALNAVMNKLFKNYKSWCKFLGRKNNLWLPQIHQEVRQRKIL
YMGLYLLVWGEAANLRFMPECLCYIYHHMASELHGMLAGNVSMVTGDNMRPAYGGEDESFLTFIVTPIYEVISRETLKN
RNGTAPHSAWRNYDDLNEYFWKGDCFRIGWPMRPDADFFFPARTSSSTNDIDGKDYQLSSKSFFVELRSFWHLFRSFD
RLWAFYILGLQAMIVLAWNVGPNLQNALTGAVLKQLLSIFITASILRLIQGKLYFRVSRGLNSYHRHIQISLRYAAFLDIVFCY
HAFRSIKLLGVLRLVLKLFTSAAWVIVLTISYARTWVNPQGLIGEIQKWLGKSLESSYLYVVAVVVYLIPNFIGALFFLFPMIR
RWIESSNWTVVRVLLWWSQPRLYIGRGMHESQIALLGYTFFWVLLIASKFAFSYFIQIEPLVAPTKAIMQQSSVIYTWHEF
FPNARNNPGALISLWAPVIMVYFMDSQIWYAVFSTIFGGISGSFRRLGEIRTLGMLRSRFSSLPGAFNESLVPDDGKRARK
GFSFSRDFEKISPSKDRSKAARFSQLWNEVITSFRQEDLISDKERDLMLVPYSSDPHLKLVQWPPFLLASKVPIALQMAKQ
AAETGRAADLLRKIKIDEYMKCAVTECYESLKRVLKRFIVGEVEIRVIDGLFDEVDVNVEKETLLDNFKLGELPVLSAKFIELL
ELLVGSLHPISRCGFPPKCLIFYELRGVHMLCYGTLAVCRKRITLVKRPLIVRKNHAGQEAIDSARDLAVLKLQDMYEVVTR
DMMSDSMRDSWDSSLGALAGGQGRKSELFSAKGDEPAKVVFPLSRSEAWIEQIKRLHLLLTERESAMDVPENLEARRRI
AFFTNSLFMNMPHAPRVRNMLSFSVLTPYFKEDVVYSKENLMKENEDGISVLFYLQKIYPDEWSNFLQRIGLESSEDPEA
QIFGCNDFEDKLREWASFRGQTLSRTVRGMMYYRRALELQAFLDMATEDELVDGYKVLTGATPEQKKSQRSTWSQLQ
AIADMKFTYVAACQMYGEQKRQGHHSATEILKLMLNNLSLRVAYIDEVEERQNESKVYYSVLVKAINGLDQEIYRIKLPGV
VRLGEGKPENQNHAVIFTRGEGLQTIDMNQDNYLEEAFKMRNLLQEFHEPHGVRPPTILGVREHIFTGSVSSLAWFMS
NQETSFVTIGQRVLAIPLKVRFHYGHPDVFDRLFHITRGGMSKASRVINLSEDIFAGFNSILRRGNVTHHEYIQVGKGRDV
GLNQISLFEAKIACGNGEQALSRDVYRLGHRFDFFRMLSCYFTTVGYYLSTMIVVLTVYVFLYGRIYLALSGVDDSLVHSAN
NKALTAALASQALVQLGLLMALPMVMEIGLERGFRTALSDFLTMQLQLASVFFTFSLGTKTHYFGRTVLHGGAKYRATGR

GFVVRHERFADNYRLYSRSHFTKAIELFLLLIVYTLYVSRSAKGAATYILITFSMWFLVASWLFAPFLFNPSGFEWQKIVEDW
DDWNKWMSNRGGIGVEGSKSWESWWDEEQEHLNFTGIFGRFVESLLSIRFFLYQYGIVYHLNIARSSKDLSISVYGLSW
LVIVAVLTVLKIVSMGRDKFSADFQLLFRLLKALLFIGSVSVIAILHVKSFTVGDLFACILAFIPTGWALLQILQACKPVVVRLG
FWKSVKALARGYEYLMGLLLFTPIAILSWFPFVSEFQTRLLFNQAFSRGLQISRILAGRK---------------------------------------
---------

>IGUH 2013096 Schwetschkeopsis fabronia 1779

MGDLVYNILPVEDNAAARDHPAVKFPEVQAAVRALRSIGDLRRPPQWRTGISVDVLDWLGCWFGFQDSNVKNQREH
LVLLLANAQMRGIPDSADRLDGKVLRKIRRKVTKNYESWCTYVGKDNVLRLPFGKQVGAERQELLYTSLFLLIWGEAANL
RFMPECLCFIFHHMAQELNKMLERDAFENFKPTTCAHNGFLRLVVSPLYEVVGAEAAVNGTGNAAHSSWRNYDDINEY
FWSERCFTHLKWPMDGSSTFLIKPQDVRNGAKHKVGKTGFVEQRTFFNIFRSFDRLWIGYILILQACIVTLWSGQQRAP
WVELRNKDSLARLLTIFITWSALRLILALLDLVMMHSLISWETWRTGLRMILKVLVASIWVLIFSIFYSSMWHRRHKDHA
WSSAANSLFIRYLYTMGAFCLPEGLALALFIIPFVRNFLEKSRFRVFHLLTWWFQSRTYVARGLREGIVDNFKYTLFWALVL
ASKFLFSYFLQLKPLIRPTKEILEITTIDYKWHQIFGKGNRVAVLALWAPVILIYFMDTQIWYTIWSALVGALVGLLDHLGEIR
NVHQLKLRFQMFPGAVQFNLIPESWGAGIREQYSWWVNVKNFFMRIRLRYGAPSQEAKEESMEARRFSHIWNEILKT
FREEDLISNRELELLEIPTPVWNISVFQWPSTLLANEVQTALDFLKSKDGGKGMDDKAVWKKTIKSEYGRCAVVESYESM
KHILVYRILRLNSPDQIMVKGLFEDHIDRAINQRKFSDAFTLSKLPEVHKCVLTLVKNILTHKADEVVKALQTLWHYVVNEF
PKAEERTLIKENFARQRLSTATVFKDSVVLPGVGDKNFYKQLKRLQTTLETKDTLLSVPKGLEARRRISFFANSLFMTMPRA
PQVEKMFAFSVLTPYYEEEVIYSLKDLNKRNEDGIDTLFYLRTIFADDWKHFKERFSGVEETDKQFIHRMSGQHDAAEGG
SQMKHDSKAKKKSGKEKTIEDDGLELCLWASYRGQTLARTVRGMMYYERALECQAFLDAATEKDLNEALGFKEMIARA
SSNVSEGSSRRQEERISDDTRERQVLATAAMKFTYVVAAQLYGKQKKKEENQAKGIAYLLKTYRGLRIAYVDEVETPAGNQ
YFSVLVKYDRTANMEVEIFRVELPGKLKLGEGKPENQNHALIFTRGDAVQTIDMNQEMYFEEALKMRNLLQEFDKHHG
VRKPTILGVREHVFTGSISSLAWFMSAQETSFVTLGQRVLANPLKIRLHYGHPDVFNRLWFMSRGGISKASRTINISEDIFA
GFNCTQRGGTVTHHEYIQAGKGRDVGLNQIALFEAKVASGNGEQVLSRDVYRLGHRLDFFRMLSFYYTTVGFFINNLM
VVLTVFAFLWGRFYLAVSGVESSLSNSKVLSNTALLASLNQQLIVQLGILTALPMIVENALEHGFTKALWEFFTMQLQLAS
VFFTFSMGTRAHYFGRTLLHGGASYRATGRGFVVKHERFAVIYRLYSRSHFVKAIELIALLVVYLIYGGHRSSSTYLLISLTSW
FMALTWLLGPFIFNPSGFDWLKTLEDFEDFIGWLKYKGGVIVDSEQSWERWWMDEQKHLKTTGLWGKLMDIILNLRF
FFFQYGIVYQLKIAATSQSIFVYIVSWSYVFVAGVIHMIIASAGQRYSTKKHGLYRAIQAILITLVIGAIVVLKIFTHFSLKDLLTS
LLAFVPTGWGILQILTVLRTDWLEKTDIWPIVVNVGRLYEFGMGLIVLAPVAILSWLPGFQAMQTRVLFNEAFSRGLHIA
QLLETVQKNPKSE----------------------------------------------------------------------------------------------------------------------
---------------------------------------------------------------------------------------------------------------------------------------------
-----------

>IGUH 2016263 Schwetschkeopsis fabronia 1841

MGDLVYNILPVDYPQEFYHAGMLFPEVKAAMTALLNIPVPPHEGIWNSNFDMLDWLGRFFGFQADNVRNQREHVVL
LLANGLMHLFPEPSPEPTGFNIPLEANVVKRIRKKLTGNYTRWCRFIGCKDNMHLLKRRRLRRGRRERPEQEEEEEEQM
GRELMYTCLFLLIWGEAANLRFMPECLCFIYHHMLVDLNIVLNDTFEDDIEVQRTPSYREPNGFLKHVVVPIYQVVKAEA
DSNKGGSAPHSSWRNYDDMNEYFWSSRCFDQLKWPFEQDSSYLREPIQERGYLNRGRKVQHHKVGKTGFVEQRSFW
YIFRSFDRIWVPHILFLQASVAILWHNGGPPWIELQKPDPLARFLSIFITWSLLRVLQALLDIGSQYSLVSRETLLTGVRMLL
KLLIAATWTILFIVYYRRMWWQRNIDQYWTVYANNRLHEYLYIAAAFIVPEVLALLLFILPWIRNFVENSNWRIFHILTW
WFQSRLFVARGLREGLFDNLKYTLFWLSVFASKCSFSYWLQIRPLIAPTKQILRANNITYRWHEFFPDGSRAAIVALWAPV
LLIYFMDIQIWYSIWSSGVGAFVGLLQHLGEIRNVHQLRLRFKIFPTAFDFSLMPKDTGHLNLWEHAKDLIGRFRLRYGW
SAMHEKVEWGNIEGGRFAHVWNKIVHTFREEDLISDRELELLEVPVGAWRLSVFQWPSALLANQILLVLINDVRYFKGD

DKMLWGIVCKNEYRMCAVIECYESIKYILLYRILKSDSVEHKIMLIVFEEIDKSIKNQRFTSTFLLRELLSIHGRVVHLVEVLLT
MPTRRQIQKVVDALQSLYESAVEDFPRDSALKDVIREQLRGRNKGTELFMDAVILPDTEDNEVFFKHLRRLHTTLSTRDPL
FNVPKGLEARRRISFFSNSLFMTMPRAPQVDRMLAFSVLTPYYSEEVIFSTKALKEENEDGISILFYLQKVFPEDWTNFLER
MKKQRLLEDHLWEIDDAIELRLWASYRGQTLARTVRGMMYYERALEVQAFLDSAKEEDFIGIKALLERTSSTNSRGSMG
SEHEWMEANKKNKMQQNLAAASMKFTYVVTCQIYGQQKRQNDYQAADILRLMKTYRGLRIAYVDERDGEEGKRYYS
VLVKYDHTLKKEVEIYRIQLPGPLKLGEGKPENQNHALIFTRGDAVQAIDMNQLLFYTRNCCLPEPSYTFTCIDTSIIRLHLC
DNILMEVDACYNMNTRGEAKGLEDEVCVNNNVSRCLQDMYFEEAFKMRNLLEEFNRKHGIRKPAILGVREHVFTGSVS
SLAWFMSAQETVFVTLNQRVYANPLKVRMHYGHPDVFDRLWFLGRGGISKASRTINISEDIFAGFNCTLRGGTVTHHEY
IQAGKGRDVGLNQIAMFEAKVASGNGEQMLSRDVYRLGHHLDFFRMFSVYYTTVGFFVSNMLIVLTVFAFLWGRVYLA
LSGIERSLTTGSGALANAALTATLNQQLVVQLGLLTALPMLVEDALEHGFTTALWNMITMQLQLASLFFTFEMGTRSHYF
GRTLLHGGAKYRATGRTFVVKHEKFAEIYRLYSRSHFTKGIELLMLLFTYLAYGVISWSATYVLVMISSWFLAFTWIMAPFIF
NPSGFDWLKTVEDFDDFMQWLWFKGDVFLKGEQSWEVWWEDEQSHLKTTGLWGKLLEIILDLRFFIFQYGIVYHLQIT
GNNTSIFVYLLSWTYMLAAILLHLVISNASDHYAADNHRLYRLIQTLTIAFIIAIGVLLWTRTNFTFLDILASFLAFLPTGWGII
QICLVLRRPFLENSKVWGTITAVARLYDLGMGLIILTPVAVLSWLPGFQAMQNRILYNEAFSRGLQISRLLTGKKNVHAD---
------------------------------------------------------------------------------------------------------------------------------------------
--------------------------------------------------------------

>IGUH 2016264 Schwetschkeopsis fabronia 1692

EEEQMGRELMYTCLFLLIWGEAANLRFMPECLCFIYHHMLVDLNIVLNDTFEDDIEVQRTPSYREPNGFLKHVVVPIYQV
VKAEADSNKGGSAPHSSWRNYDDMNEYFWSSRCFDQLKWPFEQDSSYLREPIQERGYLNRGRKVQHHKVGKTGFVE
QRSFWYIFRSFDRIWVPHILFLQASVAILWHNGGPPWIELQKPDPLARFLSIFITWSLLRVLQALLDIGSQYSLVSRETLLTG
VRMLLKLLIAATWTILFIVYYRRMWWQRNIDQYWTVYANNRLHEYLYIAAAFIVPEVLALLLFILPWIRNFVENSNWRIF
HILTWWFQSRLFVARGLREGLFDNLKYTLFWLSVFASKCSFSYWLQIRPLIAPTKQILRANNITYRWHEFFPDGSRAAIVAL
WAPVLLIYFMDIQIWYSIWSSGVGAFVGLLQHLGEIRNVHQLRLRFKIFPTAFDFSLMPKDTGHLNLWEHAKDLIGRFRL
RYGWSAMHEKVEWGNIEGGRFAHVWNKIVHTFREEDLISDRELELLEVPVGAWRLSVFQWPSALLANQILLVLINDVR
YFKGDDKMLWGIVCKNEYRMCAVIECYESIKYILLYRILKSDSVEHKIMLIVFEEIDKSIKNQRFTSTFLLRELLSIHGRVVHLV
EVLLTMPTRRQIQKVVDALQSLYESAVEDFPRDSALKDVIREQLRGRNKGTELFMDAVILPDTEDNEVFFKHLRRLHTTLS
TRDPLFNVPKGLEARRRISFFSNSLFMTMPRAPQVDRMLAFSVLTPYYSEEVIFSTKALKEENEDGISILFYLQKVFPEDWT
NFLERMKKQRLLEDHLWEIDDAIELRLWASYRGQTLARTVRGMMYYERALEVQAFLDSAKEEDFIGIKALLERTSSTNSR
GSMGSEHEWMEANKKNKMQQNLAAASMKFTYVVTCQIYGQQKRQNDYQAADILRLMKTYRGLRIAYVDERDGEEG
KRYYSVLVKYDHTLKKEVEIYRIQLPGPLKLGEGKPENQNHALIFTRGDAVQAIDMNQLLFYTRNCCLPEPSYTFTCIDTSII
RLHLCDNILMEVDACYNMNTRGEAKGLLEDEVCVNNNVSRCLQDMYFEEAFKMRNLLEEFNRKHGIRKPAILGVREHV
FTGSVSSLAWFMSAQETVFVTLNQRVYANPLKVRMHYGHPDVFDRLWFLGRGGISKASRTINISEDIFAGFNCTLRGGT
VTHHEYIQAGKGRDVGLNQIAMFEAKVASGNGEQMLSRDVYRLGHHLDFFRMFSVYYTTVGFFVSNMLIVLTVFAFLW
GRVYLALSGIERSLTTGSGALANAALTATLNQQLVVQLGLLTALPMLVEDALEHGFTTALWNMITMQLQLASLFFTFEMG
TRSHYFGRTLLHGGAKYRATGRTFVVKHEKFAEIYRLYSRSHFTKGIELLMLLFTYLAYGVISWSATYVLVMISSWFLAFTWI
MAPFIFNPSGFDWLKTVEDFDDFMQWLWFKGDVFLKGEQSWEVWWEDEQSHLKTTGLWGKLLEIILDLRFFIFQYGI
VYHLQITGNNTSIFVYLLSWTYMLAAILLHLVISNASDHYAADNHRLYRLIQTLTIAFIIAIGVLLWTRTNFTFLDILASFLAFL
PTGWGIIQICLVLRRPFLENSKVWGTITAVARLYDLGMGLIILTPVAVLSWLPGFQAMQNRILYNEAFSRGLQISRLLTGKK
NVHAD----------------------------------------------------------------------------------------------------------------------------
------------------------------------------------------------------------------------------------------------------------------------------
---------------------------------------------------------------------------------

>IGUH 2019275 Schwetschkeopsis fabronia 1994

KVPHLVHKRTIAKWERLVGRAIEAKEGRPLHGSHWNEYTNTPFVPQILHQQKKNIDNILQTARSVQDRHPQVARILYEYS
YALSQALDPRSESRGVLQFKTVLLSVIQQKRGEKTDRSEDVYIIEEFYKHLKRKLDELEDEDWLRQQPQYNQISPEAWAEL
KRNIYMTVEILNEVVDFLIKENPEMKRHVEFDSDLKEEIEKTAKKVDDYKPYNILPFEAPGLVNPFQNSPEVVAAINSIELN
ANALDDHEFHIDFKRPNMRNLDVFDFLQFTFGFQADNVLNQREHLVLLLANSQSRLGSLRNSNSGSSQFKPKLDERAIF
NVHTKLLENYERWCDFLRKDKMSNRRFQESSLMPQSRLVFSALYLLIWGEASNVRFLPECICYIFHQMADEISDPGSGIL
NHPFREKSMILKEFDAFLDAVIKPVHEIVAAEAKFCKDRKLPHSKWRNYDDINEYFWTPVCFELSWPWRLSSGFFVKPK
QDSNKKRNKKFRNTRDEIPLLQEQNQRSEPGHRREKKAGKSHFVEHRSGLHLYHSFHRLWIFLVCMLQGLAVFAFCDGK
FNATSIKYILSVGLTFVIMKFIQSVLDVILIVGAYRSSRARTLSRIWLRLFWFASLSAIIVFLFLKTIQEEESRSGQSTWFRVYCV
LLILYGGSHLFVALLMNMPWLRKQAEKYSNFGPVPFLNWVHQERYYVGRGLYENTGDYLSYILFWIVVLACKFSFSYFLQI
KTMVGPTRTIVDLTDLNYRWRDVVSKSNHNALTLVSLWAPVVMIYFLDLQVWYTVISALVGGLDGARMGLGEIRSLDM
LRNRFTFLPAAFTKRLQAHQHKVLRESVYTNEKVEAIQFAPIWNDVIASLREEDLINNKEMEWLVMPGNTLTNLTGNSQ
DLTLVQWPLFLLANKVFVACDLAEVHKQASQLELWKIIGRDPFMMFALQEAFVTLRIILEHLLEEDYLGKRWVNFVYEN
MREAMERDQLKHKYNLKKDQLKKMLDKAAKLTTVLINEAKKLKNDYTKEMREAYTAAVRKELLDFYDVVMRDFIIDSET
RDDAFLQAGKQSEGLFTELTLPTDESNSLVRRLNQILTIKESALNVPVNLEARRRLEFFSNSLFMKMPNSPTVRKMFSFSVF
TPYYEEDVIYSPSKLAEENNDGISMMYYLRTIVPDEWANFLERIYPRKEDREARKALLKTIFPKEYEFKGNEKPRKPDDLNE
TVKLQLRLWASYRGQTLARTVRGMMYYKRALMLQAKLEGESLSDDVEQGSQYSTMSTSSHLDILDAGAQAELKFLYVV
SAQIYGEQQQGGKGDDGRQKAADISYLMRTNDSLRISYIHKGKVKVGAEEVTKYYSKLMKADPSGNDQEIYSIELPGEVI
LGEGKPENQNHAIVFTRGEALQTIDMNQEHYLEETFKMRNLLEEFDDTKNYGLRKPTILGVREHVFTGSVSSLAWFMSL
QERSFVTLGQRVLAKPLKVRMHYGHPDVFDRIFHITRGGISKASKQINLSEDIFAGFNSNLRQGNVTHHEYIQCGKGRDV
GLNQIAAFEGKVASGNGEQTLSRDIYRLGQLFDFFRMLSFFFTTVGYYFTTMLTVLTVYVFLYGKVYLALSGVDADLKNKG
LSTNVALQSALDTQFLLQIGVFTAVPMIMNFLLEEGILKAIISFCTMQLQLSSVFFTFSLGTRTHYFGRTILHGGAKYASTGR
GFVVAHIPFAENYRMYSRSHFVKALEIMLLLIVYLAYGAPERTTFTYILLTFSSWFLAISWLWAPFIFNPSGFEWQKTVSDF
EDWTNWLFHKGGIGEKGVKSWEVWWEEEQEHICTIRGRFWEIILSLRFFLVQYGVVYALNIVGHDRNFRVYGFSWCVL
AGVVLTFKVFSVNQKTFSNFQLILRLFQMIVFLALIGGVVVAVAITSLTIGDVFASALSLIPTGWGLLSIAIASKPAVKKIGLW
KSVRAIARLYEAFMGAIVFIPIALLSWFPFVSTFQTRLVFNQAFSRGLEISTLLSGNNPNKDI----------------------------------
---------------

>IGUH 2021779 Schwetschkeopsis fabronia 1982

MERPPRPPHRMSKRVLVKWEKLVEKALVTSSYNRDGRGRGSNSRQRGERAPQSLAQQADIDAVLQAADEIGHENLQV
GRILTEHAYRLTQQLDPRSEGRGVLQFKTGLKSIMKQKQQAKQGEAVDRSQDIRIIQVYYKHYRERHHIDQLEEEARNRR
PVLSTDLVPESQDQSFEKLRRVYEISTILNDVVDALLKEAEPGDASRLQDTDEKRVMEEDAKKLKGFKAYNILPLKTPLDAP
SVLNPFDFFPEVVGATQVLVYTRDLPRFPSDYEVPEDRVVDIFDFLHYAFGFQKDNVANQREHIILLLSSAQSRLGTLDQG
RDSDGNKTSLNPDKAVSNVHERILENYVRWCHFLRREPQNKRAFTQQRRLILAALYLLVWGEAANLRFMPECLCFIFHN
LADECIDLLGRTYVERSKTVKPNEDGSIEFSFLDQIITPVYDAVAAEAKSSQGGKVPHSHWRNYDDFNEYFWQPNCFVEL
GWPWRTDAGFFKPPKMKDAMKVPLPQTEANLPEAAGRRRKHKVGKVHFVEHRSGFHIYHSFHRLWIFFICMLQGLTV
WAFCSKNGNLNLHVRTIKRIMSVGPTFVIMKFIQSVFDIVFMWGAFKATRLQTVARMLLRFLWFTCLSAAVLFLYIKTLEE
DARNDGSGSWFRIYYILISSYAGANAVFVFLLRIPFLQRQGAKCSNVYLFQFVKWLYQERYYVGRSMYERTRNYIKYSIFWI
VILACKFAFTMHFQIMPLVDPTRLIIGFNDIKYKWPDFVSDSNHNALTIVSLWAPVVMIYFLDTQVWYTVISAFLGGIEGA
RDKLGEIRTLEMLRKRFPNYPAAYVKHMQPPINRLSSASPSHHSASRVTKAKPKKLDAIRFQPIWNRVIKSLREEDLINNR
EKVLLKMPPNLMFHSNGAPNDLIHWPLFLLANKVHIAVELAVEHKTRHDGLWEKVRRDEYMAYAVQETFETLEPLLVSV

LNPMGASWVNDIFEKLRYSLGSADLRDSFNLNKLRDVLEKLRDLTEHLGNEDVDERRSKATRFFFQLYDVVMRDFLSSRT
REEFEEMPGFREAVQNGLLFTELNWPNKAGQKQAKRLNNLLTVQKIKDQEGKTKTLNTETIPHNLEARRRLQFFTNSLF
MHMPEAPPIRKMFSFCVFTPYYEEDVMYDMEKLNQENEDGITILFYLQKIYPDEWQNFLERIGLIEAIVIREIQNPKSGR
HEKVKLDLRLWASYRGQTLARTVRGMMYYKAALIIQGIQEGASGGDLEEGIPPSLVEAQGSINRSAEAQAELKFTYVVTC
QIYGEQKRKGKVQAADILYLMQKHESLRIAYIDVVESSKKGRKPSYYSKLCKVDRSDPSQKDQEVYSIKLPGDIKLGEGKPE
NQNHAIIFTRGDCIQTIDMNQDNFMEEAFKMRNLLEEFSQTHHGLHKPTILGVREHVFTGSVSSLAWFMSMQESSFVT
LGQRVLARPLKVRMHYGHPDVFDRVFHITRGGISKASRVINLSEDIFAGFNTTLRLGNVTHHEYIQVGKGRDVGLNQIAL
FEAKVASGNGEQTLSRDVYRLGQLLDFPRMLSFFFTSIGFYVTTMMTVLTLYVFLYGKAYLALSGVDAALKRNSKILQNPSL
EAALNTQFLFQIGIFTAVPMIVNLILEQGILKAIISFCTMQLQLASVFFTFSLGTRTHYFGRTILHGGAKYRSTGRGFVVTHIH
FAENYRSYSRSHFTKALEIIMLLIIYLAYGAQNRTSVTFILLTFSSWFLALSWLFAPYIFNPSGFEWQKTVEDFDDWTNWM
FYKGGVGVKIENSWEAWWFDEQDHIRSPRGRFWETVLSLRFFLFQYGVVYSLSVTRGSSSILVYAYSWFVLLGLVIIFKVFT
VSQKASANFQLAVRLFQGLLFVCLIAGVVVAIVLSPLTVGDVFALALALIPTGWGLLSIAIAFRPLMERMRFWKSVREIARI
YDACMGMIIFIPIAFLSWFPFVSTFQTRFVFNQAFSRGLEISLILSGNRPNR----------------------------------------------------
----------

>IGUH 2021788 Schwetschkeopsis fabronia 1979

TKRSSERSKSYVVPQSLAAQTDIDDVMEAAEAIQKEDTEVARILFEYAYNLASQMDPRNEGRGVLQFKSALKAVFLQKRT
KHHIDRSQDVRLLTEYHTMLKMRDDIVNLDEEEQAARegrvlenenpeyqawraerlrkfyaianilnnavnflapaep
EVGSRVRDPAKKALDADAKKIEQFKAYNILPLESAGVNNAFQSFPEVVAATRALYTNEWSQFPRFRANYSNEVGRDVLDI
FDFLHYSFCFQKDNVSNQREHLILLLANAESREGTLSEGTALAHNAKLDVKSIEKVHDRILANYVRWCNFLNKKPQTKLA
MNPQKQLCLTALYLLIWGEAANVRFLPECLCYIFHNMAGECFTLLEKDVVERSTVTIKVPETATPIDNEDKLYEYAFLKQIIT
PIYDVVAAEAKNSEHGKAPHGSWRNYDDFNEYFWQSSCFDLKWPWRLEAGFFTGPKKKGKKATSPGGDSVRLLGPDP
RRERRVGKINFVEHRSSFHLYHSFHRLWILLACMFQILAIWAFCSKNGNLNLHVRTIKKILSVGPTFAVMKFSKSILDVAFM
WGAIRNTHKRAVTRTLIRLAWLACLSGGIIYLYVKTLEEDARNSASTPWFRLYNLVLGSYAGAQVLFSFILRLPFLRKQVDR
CSNVRMCQFITWMQQERYYVGRGMYERTSDYVKYSLFWIVVLACKFAFTMHFQIMPMVEPTRLIIGFNNITYKWHSF
VSKGNNNVFTIVSLWAPVVMIYVLDVQVWYTVASALLGGLEGARDKLGEIRSLEMLRKRFLDYPEAFVKHMDSSRSSST
PTRQEFPRQGRFSEDSKAIQNKKDARRFLPIWNAVINSLREEDLLNNRERAMLEMPPNDNTYPNGEKDSVMCWPLFLL
ANKLYIAVNLGAENKNNKYTQEELWEKVTADQYMEFAIEESFLTLEQLLLSVLRSNDKALRWIRDIFDRVRESVRESSFVA
NYNLEKLPKAVDIIVDLTQQLGEDEAENPACRKKAMEALNKLAKLVMTDLLGKGDSDRLAYFQEAIQQEKLFNGLHWPD
EEWQKRAIRLHSLLKVHKFRDEVDGKQKTYNTESIPKNLEARRRLQFFTNSLFMHMPDAKPVSKMFSFCVFTPYYSEDV
MYDLKKKDAKKNHEKSKKDEIKELDRKNEDGITILFYLQKIYPDEFKNFLERLQLTAVEFKRQVENPTNTTNADNKPDTKL
ELRLWASYRGQTLARTVRGMMYYKKALELQAELERISDPDLERGGPSSSVRSQRNLSLRSPQAQAELKFVYLVSCQIYGE
QKKQGKAQAADILYLMEKNESLRVAYVDEVNVETGPQAKTTSYYSKLVKVDKTDKTKDQLIYSVKLPGPFKLGEGKPENQ
NHAIIFSRGDAVQTIDMNQDNYLEEAFKVRNLLEEFDQVHGRNRPTILGVREHVFTGSVSSLAMFMSMQESSFVTLGQ
RVLARPLKVRMHYGHPDIFDRIFHFTTGGVSKASSGINLSEDIFAGFNTTLRQGNVTHHEYIQVGKGRDVGLNQIATFEA
KVASGNGEQALARDLYRLGQLLDFPRMLSFFFTSVGFYVTTMMTVLTLYAFLYGKAYLALSGVDASLKANNDILQNSALQ
SVLTSQFLFQIGLFTAVPMIVNLVLELGLIQAILRFCTMQLQLASVFFTFSLGTRTHYFGRIILHGGAKYRSTGRGFVVRHINF
AENYRLFSRSHFTKAFEIILLLVIYLAYGAQNRTSATYILLTFSSWFLSLSWLYAPYIFNPSGFEWQKTVEDFDDWTNWILYK
GGVGVKSDSSWEAWWLEEQDHLRTVGGKFWEFVFSLRFFFFQYGVSYHLNAFEGSTSIMVYVYSWLTLTVFVAIFKVFT
ISQKASTRKAKLHLVVRLFQAALFILLIAGAIVAIIVSPLSLTDCFAVALAIVPTGWGLISIAIIFRHQVESIGLWHSVREIARLYD
ACMGMFIFVPIAVLSWFPFFSTFQTRLVFNQAFSRGLEISLILAGNRANTS----------------------------------------------------
-------------

>IGUH 2021864 Schwetschkeopsis fabronia 1971

MATPPRQPQRISKRVLYKWEQLVYRAKMAAERKAAEEPQGPGAGAASNTTVPQTLVQQAEIESILQTADELAKKSPDVA
RILCEYAYTLVQGLDPNSEGRGVLQFKTGLLSVIKQKRSKKEGETIDRSHDVQILQEFYQRYREQHHLDQLEYEDNKRRDSY
DEDSTTTEQRAEVKRKMYLIARILNEVVDALTKDGQTEEFDPDKKRTMEEDAKKVKGFKAYNILPLETPGVANVFQSFPE
VGGATKALWYNESELPKFPEDSRTGELDRHLDLFDFLEYAFGFQADNVANQREHLILLLSNSQSRLRVMLDTEDKLDDG
AILTVHLKMMSNYDRWCKFLRRDSMANRAYSMQLRLFLTALYLLIWGEAANLRFLPECLCYIFHHMADELYDLLDRDAV
ERSRTFLPDSPHSFLDKIVKPIYEILAAEAKICAGGRAPHSAWRNYDDFNEFFWAPSCFQLSWPWRLDAGFFRKPRKIIYT
EADRFEEPAASEESPAMVGETREKKVGKTHFVEHRTGFHIYHSFHRLFIFLVCMLQGLGIFAFCDRRLTVHTIKLILSVGPTF
VLMKLIQSVMDVTLMIGAYRSTRAHNISRMLIRFLWFTLLSAVIVLLYVKTIEEENSGSGSDTWFKAFYLVLGIYGGLQFFF
ALLLRVPWFRLQAEKCSNFYVVMFIRWVHQERYYVGRNMFERTRDYFMYTFFWFVVGTCKFAFSYFLQIQPMVGPTR
TIIGIRNVNYRWKDLISESNHNALTLVALWAPVIMIYFLDTQVWYTVISSLIGGFDGARMHLGEIRSLDMLRSRFSSLPGAF
VKNLVPSRGGGVSQADVNVPLSAVKPGNPKVDAIRFSPLWNEVIDSLREEDLINNREREWLLMPDNTTSSGTLGQQSLV
QWPLFLLANKVYVGIDIVHANRQSFQDEIWDRMKRDPYLEFAVKEAFISLQSVLMELLNEHGRSWVIGIYTDIEQAIEVG
QLLHKFNFGEIDSVLGRIAKLAEVLVSEPKNVDDESVRQEEELKMHENAARALVDLYETVMRDFLADSELREKYEMDTLL
QTSKQDGRLFSDLTWPTGPAKEQVGRLHYILAIKDSALNVPVNLEARRRLQFFSNSLFMSMPQPRPVRNMFSFSVFTPY
YSEDVMYSKAQLEDKNVDGITILYYLQTIVPDEWKNFLERMIPGVEYNQLGHYTEDHVTDTLQLRLWASYRGQTLARTV
RGMMYYKKALVLQAQQEGASVAEDEEEGHNIEGNELAIVNVSTPRTPSGSLVRNARAQAELKFCYVVSAQIYGKQKNSL
LQADKDRAADILYLMHMNDSLRIAYIHEAKQMIDGKPFTEYYSKLIKADPSGKDQEVYSIKLPGEVTLGEGKPENQNHAII
FTRGEALQTIDMNQEHYLEETLKMRNLLEEFDSKSLGLRRPTILGVREHVFTGSVSSLAWFMSLQERSFVTLGQRVLAKP
LKVRMHYGHPDVFDRIFHITRGGISKPSKQINLSEDIFAGFNSTLRQGNITHHEYIQCGDVGLNQIAAFEGKVASGNGEQ
TLSRDIYRLGQLFDFFRMCSFFFTSVGYYFTTMLTVLTVYVFLYGKVYLALSGVDEALKASDLLENTALQSALDTQFLLQIGV
FTAVPMIVNFILEQGVLRAVISFFTMQLQLSSVFFTFSLGTRTHYFGRTILHGGAKYKATGRGFVVEHIPFAENYRAYARSH
FVKGMEIIMLLIVYLVYGAHDRTAASYILLTFSSWFLALSWLYAPYIFNPSGFEWQKTVIDFDDWTNWLFHKGGIGDEGKK
SWEVWWDEEQAHIQTPRGRFWEILLSTRFFIFQYGVVYALNAAGNDKSFWVYGYSWVVIVGVFLLFKIFTFSQKASANF
QLIVRLLQGVVFLAVVAGVSVAVVLTRLTIGDVFASALAIIPTGWGLLSIAIAIRPVVQWFGIWKSVRGIARLYDAAMGMIL
FIPIALLSWFPFVSTFQTRLVFNQAFSRGLEISVLLAGNNPAA-----------------------------------------------------------------
------

>JADL 2005118 Rhynchostegium serrulatum 1776

MGDLVYNILPVDYPQEFHHAGMLFPEVKAAIAALQNVPVLPHQGIWDSDSDMLDWLGRFFGFQADNVRNQREHLVL
LLANGLMHLFPEPSPGPPGFNIPLEANVVKRIRKKVTGNYTRWCRFIGCKDNMHLLKRSRLRRGRRERPEQEDFEEEQM
GRELMYTCLFLLIWGEAANLRFMPECLCFIYHHMLVELNIVLNNTFEDDIEVQRGTPSYREQNGFLNRVIKPIYEIVKAEA
DSNKGGSAPHSSWRNYDDMNEYFWSSRCFDQLRWPFESDCSYCFKPTRERGYLNRGRKVQHRKVGKTGFVEQRSFW
YIFRSFDRIWVPHILFLQASALILWHNGGPPWIELQKPDPLARFLSIFITWSGLRVLQALLDIGSQYSLVSRETLLTGVRMLL
KLLVAATWTILFIIYYRRMWWQRNIDQYWTVYANNRLHEYLYIAAAFIVPEVLALLLFILPLIRNFVENSNWRVFHILTWW
FQSRLFVARGLREGLLDNLKYTLFWLSVFASKFSFSYWLQIRPLIAPTKQILRARNVTYRWHEFFPDGSRAAIVALWAPVLL
IYFMDIQIWYSIWSSGVGAFVGLLQHLGEIRNVHQLRLRFKIFPTAFDFSLMPKDTGHLNLWEHAKDLVGRFRLRYGWS
AIHEKIEWRNIEGGRFAHVWNKIVHTFREEDLISDRELELLEVPVGAWRLSVFQWPSALLANQILQVLINDVHYFKGDDK
LLWSKIIKNEYRRCAVIECYESIKHILLCRILKTDSVEHKIMLIVFEEIDRSIKNQRFTSTFLLRELLSIHGRVVHLVEVLLDMPT
RRQIQKVVDALQSLYESAVEDFPRDSALRDVIREQLRERNIETELFMDAVILPDIEDNDAFFKHLRRLHTTLSTRDPLFNVP
KGLEARRRISFFSNSLFMTMPRAPQVDRMLAFSVLTPYYSEEVIFSTKALKEENEDGISIWFYLQKVFPEDWTNFLQRME

KQGLSEDHLWEKDDAIELRLWASYRGQTLARTVRGMMYYERALEVQAFLDSAKEEDFIGIKALLERASSSNSIGSTGGS
MGSEHEWMEANKKNKMQQNLAAASMKFTYVVTCQIYGQQKRQKDYQAADILRLMKTYRGLRIAYVDERNDEEGGKI
YYSVLVKYDHTLEKEVEIYRIQLPGPLKLGEGKPENQNHALIFTRGDAVQTIDMNQDMYFEEAFKMRNLLEEFNRKHGIR
KPAILGVREHVFTGSVSSLAWFMSAQETVFVTLNQRVYANPLKVRMHYGHPDVFDRLWFLGRGGISKASRTINISEDIFA
GFNCTLRGGTVTHHEYIQAGKGRDVGLNQIAMFEAKVASGNGEQMLSRDVYRLGHHLDFFRMFSFYYTTVGFFVSNM
LIVLTVFAFLWGRVYLALSGIERSLTTGSDALANGALTATLNQQLVVQLGLLTALPMLVEDALEHGFTTALWNMITMQLQL
ASLFFTFEMGTRSHYFGRTLLHGGAKYRATGRTFVVKHEKFAEIYRLYSRSHFTKGIELLMLLFTYLAYGVITSSATYVLVMIS
SWFLAFTWIMAPFIFNPSGFDWLKTVEDFDDFIQWLWFKGDVFLKAEQSWEVWWEDEQSHLRTTGLWGKFLEIILDL
RFFIFQYGIVYHLQIAGNNTSIFVYLLSWTYMLAAILLHLIISNASDHYAADNHRLYRLIQTLTIAVIVAVAVVLWTQTKFTFL
DILASFLAFLPTGWGIIQICLVLRRPFLENSKVWGTITAVARLYDLGMGLIILTPVAVLSWLPGFQAMQTRILYNEAFSRGLQ
ISRLLTGKKN--------------------------------------------------------------------------------------------------------------------------------
------------------------------------------------------------------------------------------------------------------------------------------------
-------

>JADL 2005119 Rhynchostegium serrulatum 1836

MGDLVYNILPVDYPQEFHHAGMLFPEVKAAIAALQNVPVLPHQGIWDSDSDMLDWLGRFFGFQADNVRNQREHLVL
LLANGLMHLFPEPSPGPPGFNIPLEANVVKRIRKKVTGNYTRWCRFIGCKDNMHLLKRSRLRRGRRERPEQEDFEEEQM
GRELMYTCLFLLIWGEAANLRFMPECLCFIYHHMLVELNIVLNNTFEDDIEVQRGTPSYREQNGFLNRVIKPIYEIVKAEA
DSNKGGSAPHSSWRNYDDMNEYFWSSRCFDQLRWPFESDCSYCFKVSVMIRTSTVRHNKDRGLYNCVYTWSTPIDSR
AHKMVCRSSRCFDQLRWPFESDCSYCFKPTRERGYLNRGRKVQHRKVGKTGFVEQRSFWYIFRSFDRIWVPHILFLQAS
ALILWHNGGPPWIELQKPDPLARFLSIFITWSGLRVLQALLDIGSQYSLVSRETLLTGVRMLLKLLVAATWTILFIIYYRRMW
WQRNIDQYWTVYANNRLHEYLYIAAAFIVPEVLALLLFILPLIRNFVENSNWRVFHILTWWFQSRLFVARGLREGLLDNL
KYTLFWLSVFASKFSFSYWLQIRPLIAPTKQILRARNVTYRWHEFFPDGSRAAIVALWAPVLLIYFMDIQIWYSIWSSGVG
AFVGLLQHLGEIRNVHQLRLRFKIFPTAFDFSLMPKDTGHLNLWEHAKDLVGRFRLRYGWSAIHEKIEWRNIEGGRFAHV
WNKIVHTFREEDLISDRELELLEVPVGAWRLSVFQWPSALLANQILQVLINDVHYFKGDDKLLWSKIIKNEYRRCAVIECY
ESIKHILLCRILKTDSVEHKIMLIVFEEIDRSIKNQRFTSTFLLRELLSIHGRVVHLVEVLLDMPTRRQIQKVVDALQSLYESAV
EDFPRDSALRDVIREQLRERNIETELFMDAVILPDIEDNDAFFKHLRRLHTTLSTRDPLFNVPKGLEARRRISFFSNSLFMT
MPRAPQVDRMLAFSVLTPYYSEEVIFSTKALKEENEDGISIWFYLQKVFPEDWTNFLQRMEKQGLSEDHLWEKDDAIEL
RLWASYRGQTLARTVRGMMYYERALEVQAFLDSAKEEDFIGIKALLERASSSNSIGSTGGSMGSEHEWMEANKKNKM
QQNLAAASMKFTYVVTCQIYGQQKRQKDYQAADILRLMKTYRGLRIAYVDERNDEEGGKIYYSVLVKYDHTLEKEVEIYR
IQLPGPLKLGEGKPENQNHALIFTRGDAVQTIDMNQDMYFEEAFKMRNLLEEFNRKHGIRKPAILGVREHVFTGSVSSLA
WFMSAQETVFVTLNQRVYANPLKVRMHYGHPDVFDRLWFLGRGGISKASRTINISEDIFAGFNCTLRGGTVTHHEYIQA
GKGRDVGLNQIAMFEAKVASGNGEQMLSRDVYRLGHHLDFFRMFSFYYTTVGFFVSNMLIVLTVFAFLWGRVYLALSGI
ERSLTTGSDALANGALTATLNQQLVVQLGLLTALPMLVEDALEHGFTTALWNMITMQLQLASLFFTFEMGTRSHYFGRTL
LHGGAKYRATGRTFVVKHEKFAEIYRLYSRSHFTKGIELLMLLFTYLAYGVITSSATYVLVMISSWFLAFTWIMAPFIFNPSG
FDWLKTVEDFDDFIQWLWFKGDVFLKAEQSWEVWWEDEQSHLRTTGLWGKFLEIILDLRFFIFQYGIVYHLQIAGNNTS
IFVYLLSWTYMLAAILLHLIISNASDHYAADNHRLYRLIQTLTIAVIVAVAVVLWTQTKFTFLDILASFLAFLPTGWGIIQICLVL
RRPFLENSKVWGTITAVARLYDLGMGLIILTPVAVLSWLPGFQAMQTRILYNEAFSRGLQISRLLTGKKN---------------------
------------------------------------------------------------------------------------------------------------------------------------------------
----------------------------------------------------

>JADL 2005634 Rhynchostegium serrulatum 1982

MDRPPRPPHRMSKRVLVKWEKLVEKALVTSSYNREARGRGSNSRQRGERAPQSLAQQANIDAVLQAADEIGQDNLQV
GRILTEHAYRLTQQLDPRSEGRGVLQFKTGLKSIIKQKQQARQGEVVDRSQDIRIIQIYYKHYRERHRIDQLEEEARNRRP
MLSTDLVPESQDQSFEKLRRVYEISTILNEVVDALLKEAEPEDASRLQDTDEKRVLEEDAKKLKGFKAYNILPMKTPLGAPS
VLNPFDFFPEVVGATQVLIYTRDLPRFPSDYEVPEDRVVDIFDFLHYAFGFQKDNVANQREHIILLLSSAQSRLGTLDQGR
DSDGNKTSLNPDKAVSNVHERILENYVRWCHFLRREPQNKRAFTQQRRLILAALYLLVWGEAANLRFMPECLCYIFHNL
ADECIDLLGRTYVERSKTVKANEDGSLEFSFLDQIITPVYDAVAAEAESSQGGKVPHSHWRNYDDFNEYFWQPNCFVEL
EWPWRTDAGFFKLPKMKDPMKVPLPQTEANLPEAAGRRRKLKVGKVHFVEHRSGFHIYHSFHRLWIFFICMLQGLTV
WAFCSKNGNLNLHVRTIKRIMSVGPTFVILKFIQSVFDVVFMWGAFKATRPQTVARMLLRFLWFTCLSAAVLFLYIKTLEE
DARNDGSGSWFRIYYILISSYAGANVVFVFLLRIPFLQRQGAKCSNVYLFQFVKWLYQERYYVGRSMYERTRNYIKYSVF
WIVILACKFAFTMHFQIMPLVDPTRLIIGFNDIKYKWPDFVSDSNHNALTIVSLWAPVVMIYFLDTQVWYTVISAFLGGIE
GARDKLGEIRTLEMLRKRFPNYPAAYVKHMQPPINRLSSASPSHHSAPRVNKTKPRKPDAIRFQPIWNHVIKSLREEDLIN
NREKVLLKMPPNLMFHSNGAPNDLIHWPLFLLANKVHIAVELAVEHKTHQDGLWEKVRRDEYMAYAVQETFETLEPLL
VSVLNSDGARWVNEIFEKLRYSLGTADLRDNFNLNKLRDVLEKLRDLTEHLGNEDVDERRGKATRCFFQLYDVVMRDFLS
FKTREEFLETPGFQEAVQNGLLFTELMWPNKAGQKQAKRLNNLLTVQKIKDQEGKTKTLNTETIPHNLEARRRLQFFTN
SLFMHMPEAPPIRKMFSFCVFTPYYEEDVMYDMEKLSQENEDGITILFYLQKIYPDEWQNFLERIGLIEKTVIKEINNRKS
ERHEKVKLDLRLWASYRGQTLARTVRGMMYYKAALIIQGMQEGASGGDLEEGIPPSLVEAQGSINRSAEAQAELKFTYV
VTCQIYGEQKRKGKVQAADILYLMQKHESLRIAYIDVVESSRKGRKPSYYSKLCKVDRSDPSQKDQEVYSIKLPGSIKLGEG
KPENQNHAIIFTRGDCIQTIDMNQDNFMEEAFKMRNLLEEFSQTHHGLHKPTILGVREHVFTGSVSSLAWFMSMQESS
FVTLGQRVLARPLKVRMHYGHPDVFDRVFHITRGGISKASRVINLSEDIFAGFNTTLRLGNVTHHEYIQVGKGRDVGLNQ
IALFEAKVASGNGEQTLSRDVYRLGQLLDFPRMLSFFFTSIGFYVTTMMTVLTLYVFLYGKAYLALSGVDAALKRNSKILQN
PSLEAALNTQFLFQIGIFTAVPMIVTLILEQGILKAIISFCTMQLQLASVFFTFSLGTRTHYFGRTILHGGAKYRPTGRGFVVT
HIHFAENYRSYSRSHFTKALEIIMLLIIYLAYGAQNRTSVTFILLTFSSWFLALSWLFAPYIFNPSGFEWQKTVEDFDDWTN
WMFYKGGVGVKIENSWEAWWFDEQDHIRSPRGRFWETFLSLRFFLFQYGVVYSLSVTRGSSSILVYAYSWFVLLGLVIIF
KIFTVSQKASANFQLAVRLFQGLLFVCLIAGVVVAIVLSPLTVGDVFALALALIPTGWGLLSIAIAFRPLMERMRFWKSVRE
IARVYDACMGMIIFIPIAFLSWFPFVSTFQTRLVFNQAFSRGLEISLILSGNRPNR---------------------------------------------
----------------

>JMXW 2005869 Bryum argenteum 1898

LAQQTDIDAVLQAADEIGHDNLQVARILDEHALKLTQQLDPRSEGRGVLQFKTALKSIIKQKQQARQGDVVDRSQDIRIL
QIYYKHYREMHKIDQLEEEARNPRLLLSTDQRREQDSDSQDMSVDKLRKVYEISRILNDVVDTLLKDAEPGDASRLQDTD
EKRVMEEDARKLKGFKPYNILPLKSPIDTPSVLNAFDYFPEVEGSTQLLVYTQDLPRVLDLFDFLHYAFGFQKDNVANQRE
HITLLLASAQSRLGPLDGGRDGDGSKLNLDKAISNVHERILENYVRWCHFLRREPQNKRAFTQQRRLTLTALYLLVWGEA
ANLRFMPECLCYIFHHLADECIDLLGRTFVDRSKIVKANPDGSMEFSFLEQVIQPVYDVVAAEARFGQGGKVPHSDWRN
YDDFNEYFWQPNCFVELGWPWRTDAGFFKNPKVKLPRRNWLQAPPDDQVPNPEIRGRRRKHKVGKVHFVEHRTGF
HIYHSFHRLWIFLVCMLQGLTIWAFCSKNGNLNLHVRTIKKIMSVGPTFVVMKFIQSVFDIVFMWGAFKATRMQTVAR
MLLRFLWFTCLSGAVLFLYIKTLEEDARNDGSGAWFRIYWILIASYAGANLIFVFLLRIFQFFKWLHQERYYVGRSMYERTR
NYFKYSLFWIVVLICKFAFTMHFQIIPLVDPTRLIISFNNIVYKWPDFVSDSNHNALTIVSLWAPVIMIYFLDTQIWYTVISAV
LGGIEGARDKLGEIRTLEMLRKRFPKYPAAYVKRMQPPLNRFTSVYAADHDTSRSHRDLPKKQDAIRFQPIWNSVIKSLRE
EDLINNREKVLLKMPPNSMFHSNGKPNDLIYWPLFLLANKVHIAVELAVEHKSRLEELWEKVHRDEYMAYAVREAFETLE
PMLLSVLNPAGQNWARQIFEVLNRSLEDSTFHDYFSLNKLRDVLEKLRDLTEHLGYEDVSERRKKATTSFFQLYDVVMRD
FITGQFRAEFFDYWREFQEDLSQGVLFTDLAWPNKAGQKQAKRLNNLLTVQKIKDQEGKTKTLNTETIPHNLEARRRLQ
FFTNSLFMHMPQAPPRKMFSFCVFTPYYEEDVMYDMEKLSQENEDGITILFYLQKIYPDEWQNFLERIGLIENIVFREIEN
RNEKVKLDLRLWASYRGQTLARTVRGMMYYKAALIIQGMQEESSSGDLEEGDSSSPDARLEAQAQAELKFTYVVTCQIY

GEQKRKGKVQAADILYLMQMHDSLRIAYVDVVESSRKGSKPSYYSKLCKFDKADASLKDQEVYSIKLPGDIKLGEGKPEN
QNHAIIFTRGDCIQTIDMNQDNFLEEAFKMRNLLQEFSLTHHGLHKPTILGVREHVFTGSVSSLAWFMSMQESSFVTLG
QRVLARPLKVRMHYGHPDVFDRVFHITRGGISKASRVINLSEDIFAGFNTTLRLGNVTHHEYIEVGKGRDVGLNQIALFEA
KVASGNGEQTLSRDVYRLGQLLDFPRMLSFFYTSIGFYVTTMMTVLTLYVFLYGKAYLALSGVDASLKRNSEILQNPALESA
LNTQFLFQIGIFTAVPMIVNLILEEGILKAIISFCTMQLQLASVFFTFSLGTRSHYFGRTILHGGAKYRSTGRGFVVTHIHFAE
NYRSYSRSHFTKALEIIMLLIIYLAYGAQNRTSVTFILLTFSSWFLALSWLFAPYIFNPSGFEWQKTVEDFDDWTNWMFYK
GGVGVKVENSWEAWWFDEQDHIRSPRGRMWEFILSLRFFLFQYGVVYSLSVTRGSNSILVYAYSWLVIIGLVIIFKVFTVS
QKASANFQLAVRLFQGQLFVYLIAGLVVSIVLSPLTVGDVFALALALVPTGWGLLSIAIAFRPLMERMHVWKSVREIARVY
DACMGMIIFIPIAFLSWFPFVSTFQTRLVFNQAFSRGLEISLILGNRPNR-------------------------------------------------------
------------------------------------------------------------------------------------------

>LNSF 2070349 Hypnum subimponens 1634

RRDSMANRAYSMQLRLFLTALYLLIWGEAANLRFLPECLCYIFHHMADELYDLLDRDTVERSRTFLPDSPHSFLDKIVKPIY
EILAAEAKICAGGKAPHSAWRNYDDFNEFFWAPSCFELSWPWRLEAGFFRKPKKIIYTEADRFEEPAASEESPAVMGETR
EKKVGKTHFVEHRTGFHIYHSFHRLFIFLVCMLQGLGIFAFCDRRLTIHNIKLIMSVGPTFVLMKLIQSVMDVTLMIGAYRS
TRAHNISRMLIRFVWFTLLSAAIVLLYVKTIEEENSGSGSDTWFKAFYLVLGIYGGLQFFFALLLRVPWFRLQAEKCSNFYIV
MLIKWVHQERYYVGRNMFERTRDYFMYTFFWFVVGTCKFAFSYFLQIQPMVGPTRTIIGIRNVNYRWKDLISESNHNA
LTLVALWAPVIMIYFLDTQVWYTVISSIIGGFDGARMHLGEIRSLDMLRSRFSSLPGAFVKNLVPSRGGGVSQPDVNVPLS
AVKPGNPVDAIRFSPLWNEVIESLREEDLINNREREWLLMPDNTTSSGALGQHSLVQWPLFLLANKVYVAIDIVHDNRQ
SFQNEIWDRMKRDPYLEFAVMEAFISLQSVLMDLLNEHGRTWVIGIYADIEQAIEVGQLLHKFNFTEIDSVLGRVAKLAE
VLVAEPKKVDDDSMRQEEELKMHESAARALVDLYEAVMRDFLADSELREKYESDTLLQNSKQDGRLFSDLTWPTGPAKE
QVGRLHYILAIKDSALNVPVNLEARRRLQFFSNSLFMSMPQPRPVRNMFSFSVFTPYYSEDVMYSKAQLEDTNVDGITIL
YYLQTIVPDEWKNFLERMFPGVDYNQLGHYTETDVTDTLQLRLWASYRGQTLARTVRGMMYYKKALVLQAQQEGASV
AEDEEEGHGIEGNELAIVSVSTPRTPSGSLVRNARAQAELKFCYVVSAQIYGKQKNSMLQADKDRAADILYLMHMNDSL
RIAYIHEAKQMVEGKSFTEYYSKLIKADPSGKDQEVYSIKLPGEVTLGEGKPENQNHAIIFTRGEALQTIDMNQEHYLEETL
KMRNLLEEFDSKSLGLRRPTILGVREHVFTGSVSSLAWFMSLQERSFVTLGQRVLAKPLKVRMHYGHPDVFDRIFHITRG
GISKPSKQINLSEDIFAGFNSTLRQGNITHHEYIQCGKGRDVGLNQIAAFEGKVASGNGEQTLSRDIYRLGQLFDFFRMCS
FFFTSVGYYFTTMLTVLTVYVFLYGKVYLALSGVDEALKASDLLENTALQAALDTQFLLQIGVFTAVPMIVNFILEQGVLRAV
ISFFTMQLQLSSVFFTFSLGTRTHYFGRTILHGGAKYKATGRGFVVEHIPFAENYRAYARSHFVKGMEIIMLLIVYLVYGAH
DRTAASYILLTFSSWFLALSWLYAPYIFNPSGFEWQKTVIDFDDWTNWLFHKGGIGDEGKKSWEVWWDEEQAHIQTPR
GRFWEILLSTRFFIFQYGVVYALNAAGNDKSFWVYGYSWVVIVGVFLLFKIFTFSQKASANFQLIVRLLQGVVFLAVVAGV
SVAVVLTRLTIGDVFASALAIIPTGWGLLSIAIAIRPVVQWFGIWKSVRGIARLYDAAMGMILFIPIALLSWFPFVSTFQTRL
VFNQAFSRGLEISVLLAGNNPNAA-----------------------------------------------------------------------------------------------
-------------------------------------------------------------------------------------------------------------------------------------------
-------------------------------------------------------------------------------------------------------------------------------------------
--------------------------------------

>QKQO 2005131 Pseudotaxiphyllum elegans 1758

HAGMLFPEVKAAMAALQNIPVLPHQGIWDNNADMLDWLGRFFGFQADNVRNQREHLVLLLANGLMHSFPEPSPGP
TSFNIPLEANVVKTIRKKLTENYTRWCRFIGCKDNMHLLKRRRLRRGRRERPEPEEGEENMGRELMYTCLFLLIWGEAGN
LRFMPECLCFIYHHMLVELNIVLNNTFEDDIEVQRGTPSYREQNGFLKHVIVPIYQVVKAEADSNKGGSAPHSSWRNYD

DMNEYFWSSRCFDQLRWPFEPDSSYLREPIRERGYLNRGRNVQHHKVGKTGFVEQRSFWYIFRSFDRIWVPHILFLQAS
VVILWHNGGPPWIELQKPDPLARFLSIFITWSLLRVLQSLLDIGSQYSLVSRETLLPGVRMLLKLLVAATWTILFIIYYRRMW
WQRNIDQYWTVYANNRLHEYLYIAAAFIVPEVLALLLFILPWIRNFVENSNWRVFHILTWWFQSRLFVARGLREGLLDNL
KYTLFWLSVFASKCSFSYWLQIRPLIAPTKQILQAKNVTYRWHEFFPDGSRAAIVALWAPVLLIYFMDIQIWYSIWSSGVG
AFVGLLQHLGEIRNVHQLRLRFKIFPSAFDFSLMPKDTGHLNLWEHAKDLVSRFRLRYGWSAIHEKVEWRRGRFAHVW
NKIVHTFREEDLISDRELELLEVPVGAWRLSVFQWPSALLANQILQVLINDVHYFKGDDKMLWGIISKNDYRRCAVIECYE
SIKYVLLCRILKTDSVEHKIMLIVFEEIDKSIKNQRFTSTFRPGELLSIHGRVVHLVEVLLTMPTRFRDRTGPFMTVVDALQSL
YESAVEDFPRDPALKDVIREQLRERNKETELFMDAVILPDTEDNEAFFKHLRRLHTTLSTRDPLFNVPKGLEARRRISFFSN
SLFMTMPRAPQVDRMLAFSVLTPYYSEEVIFSTKALKEENEDGISILFYLQKVFPEDWTNFLERMRKQGLLEHHLWEKDD
AIELRLWASYRGQTLARTVRGMMYYERALEVQAFLDSAKEEDFIGIKALLERASSTNSRGSMGSEHEWIEANKKNKMQ
QNLAAASIKFTYVVTCQIYGQQKRQKDYQAADILRLMKNYQGLRIAYVDERNDEEGKNYYSVLVKYDHTLEKEVEIYRIQL
PGPLKLGEGKPENQNHALIFTRGDAVQTIDMNQDMYFEEAFKMRNLLEEFNRFHGIRKPAILGVREHVFTGSVSSLAWF
MSSQETVFVTLNQRVYVNPLKIRMHYGHPDVFDRLWFLGRGGISKASRTINISEDIFAGFNCTLRGGTVTHHEYIQAGKG
RDVGLNQIAMFEAKVASGNGEQMLSRDVYRLGHHLDFFRMFSFYYTTVGFFVSNMLIVLTVFAFLWGRVYLALSGIERS
LTTGSGALANAALTATLNQQLVVQLGLLTALPMLVEDALEHGFTTALWNFITMQLQLASLFFTFEMGTRSHYFGRTLLHG
GAKYRATGRTFVVKHEKFAEIYRLYSRSHFTKGIELLMLLFTYLAYGVITTSATYVVVMISSWFLAFTWIMAPFIFNPSGFD
WLKTVEDFDDFIQWLWFKGDVFLKGEQSWEVWWEDEQSHLKTTGLWGKLLEIILDLRFFIFQYGIVYHLQITGNNTSIF
VYLLSWTYMLAAILLHLIISNASDRYAADNHLLYRLIQTLTIAVIVAIGVVLYIKTNFTFLDILASFLAFLPTGWGIIQICLVLRRP
FLENSKVWGTITAVARLYDLGMGLIILTPVAVLSWLPGFQAMQTRILYNEAFSRGLQISRLLTGKKNIHAD-------------------
-------------------------------------------------------------------------------------------------------------------------------
-------------------------------------------------------------------------------------------------------------------------

>QKQO 2008279 Pseudotaxiphyllum elegans 1986

MASGGRLEFGATDLPRRLSRTNTAGGLTEVFDSEVVPSSLASIAPILRVANEIEASSARVAYLCRYHAFEKAHRIDPTSSGR
GVRQFKTALLQRLERDSEPTLALRHRRSDAREIQSYYQNYYNDYVKALDGAEHSDRAQLARAYQTAAVLFEVLKAVNRDK
AEEPPPEIIAAAADVEQKKEIYVPYNILPLDAAGASQAIMQLDEVRAAVEALRNVRGLPSLTEKEPHSRAGDLDCLDWLQ
DMFGFQKDNVANQREHLILMLANVHIRLLPRPEPMHKLDDRALNAVMNKLFKNYKSWCKFLGRKNNLWLPQIHQEV
RQRKILYMGLYLLVWGEAANLRFMPECLCYIYHHMASELHGMLAGNVSMVTGDNMKPAYGGEDESFLTFIVTPIHKVIS
RESLKNRNGTAPHSAWRNYDDLNEYFWKGDCFRMGWPMRPDADFFVPAQTSSSTNDVHGKDYHSSSKSFFVEIRSF
WHLFRSFDRLWAFYILGLQAMIVLAWNVGPNLQNAFTGAVIKQILSIFITASILRLIQAFLDIVFGYHAFRSIKLSGVLRLVLK
LFTSAAWVIVLTISYARTWVNPQGLIGEIQKWLGKSLESSYLYVAAVMVYLIPNFIGAFFFLFPMIRRWIESSNWTMVRVL
LWWSQPRLYIGRGMHESQIALLGYTFFWVLLIASKFAFSYFIQIEPLVAPTKAIMQQSSVVYTWHEFFPNARNNPGALVS
LWAPVIMVYFMDSQIWYAVFSTIFGGISGSFRRLGEIRTLGMLRSRFSSLPGAFNESLVPDDGKRARKGFSFSRDFEKIAPS
KDRLKAARFSQLWNEVITSFRQEDLISDKERDLMLVPYSSDPHLKLVQWPPFLLASKVPIALQMAKQAAETGRTADLLRKI
KIDEYMKCAVTECYESLKRVLKRFIVGEVEMRVIDGLFDEVDVNVEKETLLDNFKLGELPVLSAKFIELLELLEKNHAGQEAI
DSARDHAVLKLQDMYEVVTRDMMSDSMRDSWDSSLGALAGGQGRKSELFSAKGDEPAKVVFPLSRNEAWIEQIKRL
HLLLTERESAMDVPENLEARRRIAFFTNSLFMNMPHAPRVRNMLSFSVLTPYFKEDVVYSKENLMKENEDGISVLFYLQK
IYPDEWSNFLQRVGLETSEDPETQIFGCNDLEDKLREWASFRGQTLSRTVRGMMYYRRALELQAFLDMTTFPLFNHLNL
SLSFQLGDQEKVCVPQRCGCLTSLGHLMGHYFLYSWVFKSDDYVLAELVDGYKVLTDATPEQKKSQRSTWSQLQAIAD
MKFTYVAACQMYGEQKRRGHHSATEILKLMLNNLSLRVAYIDEVEERWNEKPSKVYYSVLVKAINGLDQEIYRIKLPGIVR
LGEGKPENQNHAVIFTRGEGLQTIDMNQDNYLEEAFKMRNLLQEFHEPHGVRPPTILGVREHIFTGSVSSLAWFMSNQ
ETSFVTIGQRVLASPLKVRFHYGHPDVFDRLFHITRGGMSKASRVINLSEDIFAGFNSILRRGNVTHHEYIQVGKGRDVGL
NQISLFEAKIACGNGEQALSRDIYRLGHRFDFFRMLSCYFTTVGYYFSTMIVVLTVYVFLYGRIYLALSGVDDSLVHSANNK

ALTAALASQALVQLGLLMALPMVMEIGLERGFRTALSDFLTMQLQLASVFFTFSLGTKTHYFGRTILHGGAKYRATGRGF
VVRHERFADNYRLYSRSHFTKAIELFLLLIVYTLYVSRSAKGAVTYILITFSMWFLVASWLFAPFLFNPSGFEWQKIVEDWD
DWNKWMSNRGGIGVEGSKSWESWWDEEQEHLNYTGVFGRFVESLLSIRFLLYQYGIVYHLNIARSSKDLSISVYGLSWL
VIVAVLTVLKIVSMGRDKFSADFQLLFRLLKASLFIGSVSVIAILHVKSFTVGDLFACILAFIPTGWALLQISQASKPVVIRLGF
WKSVKALARGYEYLMGLLLFTPIAVLSWFPFVSEFQTRLLFNQAFSRGLQISRILAGRKKL-----------------------------------
---------------------

>QKQO 2010537 Pseudotaxiphyllum elegans 1779

MGDLVYNIVPVDDNAAARDHPAAKFAEVKAAVNALRSIGDLRRPPQWRNGISVDVLDWLGCWFGFQDSNVKNQRE
HLVLLLANAQMRATPESPNKLDDKVLRKIRRKVTKNYESWCTFVGKENALRLPFGKQAGAERQELLYTSLFLLIWGEAAN
LRFMPECLCFIFHHMAQELNKMLEKDAFEITKPTTCEHNGFLRLVVTPLYKVVEAEAALNGTGNAAHSSWRNYDDINEY
FWSERCFTHLKWPMDGSSNFLVKPEDVRNGAKHKVGKTGFVEQRTFFNIFRSFDRLWIGYILILQACIVTLWSGQQRAP
WVELRNKDSQARLLTIFITWSALRLILALLDLVMMHSLISWETWRTGLRMVLKVLVASIWVLVFSIFYSSMWHRRHKDH
AWSSAANSLFVRYLYTMAAFCLPEGLALALFIIPFVRNFLEKSRFRVFHLLTWWFQSRIYVARGLREGIVDNFKYTLFWTLV
LASKFLFSYFLQLKPLIRPTKEILEITTIDYKWHQIFGKGNRVAVLALWAPVILIYFMDTQIWYTIWSALVGALVGLLDHLGEI
RNVHQLKLRFQMFPSAVQFNLIPESWGAGIRERYSWWVNVKNFFMRIRLRYGAPSQEAKEESMEARRFSHIWNEILKT
FREEDLISNRELELLEIPTPVWNISVFQWPSTLVANEVQTALDFLKSKDGGKYMEDKAVWKKIVKSEYGRCAVVESYESIK
HILVYRILRLNSPDQIMVKSLFEDHVDKAINQRKFSDAFTLSKLPEVHKCVLTLVKNILTHKTDEVVKALQTLWHYVVNEFA
RDEERTLIKENFEGQHLSTATVFKDSVVLPGDGDENFYKQLKRLQTTLETKDTLLSVPKGLEARRRISFFANSLFMTMPRA
PQVEKMFAFSVLTPYYEEEVIYSLKDLNKTNEDGIDTLFYLRTVFPDDWKNFKERFSAVEETDKQFINHMSGQDDAAEGG
SQMKHDSKAKKKSGKEKTIEDDGLELCLWASYRGQTLARTVRGMMYYERALECQAFLDAATEKDLNEALGFKEMIARA
SSNVSEGSSRRQEERISDDTRERQVLATAAMKFTYVVAAQLYGKQKNIAENQAKGIAYLLKTYKGLRIAYVDEVETPTGNQ
YFSVLVKYDQAANMEMEIFRVQLPGKLKLGEGKPENQNHALIFTRGDAVQTIDMNQEMYFEEALKMRNLLQEFDKRH
GVRKPTILGVREHVFTGSVSSLAWFMSAQETSFVTLGQRVLANPLKIRMHYGHPDVFNRLWFMSRGGISKASRTINISE
DIFAGFNCTQRGGTVTHHEYIQAGKGRDVGLNQIALFEAKVASGNGEQVLSRDVYRLGHRLDFFRMLSFYYTTVGFFIN
NLIVVLTVFAFLWGRFYLAVSGIESSLSNSKVLSNTALLASLNQQLIVQLGILTALPMIVENALEHGFTKALWEFFTMQLQL
ASVFFTFSMGTRAHYFGRTLLHGGASYRATGRGFVVKHERFAVIYRLYSRSHFVKAIEVMALLIVYLIYGASRSSSTYLLISLT
SWFMALTWLLGPFIFNPSGFDWLKTLEDFEDFTGWLKYKGGVIVDSEQSWERWWMDEQKHLKTTGLWGKLMDIILN
LRFFFFQYGIVYQLKIAATSQSIFVYIVSWSYVFVAGVIHMIIASAGQRYSTKKHGLYRAIQAILITLVIGAIVLLKVFTHFSLKD
LLTSLLAFVPTGWGILQILTVLRTDWLEKTYIWPIVVNVGRLYEFGIGLIVLAPVAILSWLPGFQAMQTRVLFNEAFSRGLHI
SQLLVTVQKAKKSE--------------------------------------------------------------------------------------------------------
---------------------------------------------------------------------------------------------------------------------------------------
------------

>TMAJ 2010647 Neckera douglasii 2044

RVPHLVRKRTIEKWERLVGGAIEREEERTLHGSHWNEYTDTPFVPQILHQQKKNIDNILQTARSVQDRYPQVARILYEYSY
ALSQALDPRSESRGVLQFKTGLLSVIQQKRGEKTDRSEDVYIIEGFYKDLKRKLDDLEDEDWLRQQPQYNKLSPERWAEL
KRNLYMTVEILNEVVDFLIKENPEMKRDVEFDSELKEEIEKTAKKVDDYKPYNILPFEAPGLVNPFQNSPEIGAAINSIALD
DRELNALDDREFDRDFKRPNMRDLDVFDFLQFIFGFQADNVLNQREHLVLLLANSQSRLGSLRNSNSGSSQFKPKLDEH
AILKVHTKLLENYERWCDFLGKKKMSNRRLQGSFMPQSRIVFSALYLLIWGEASNVRFLPECICYIFHQMAGEISDPESVIL
SHPFNERSIILKDFDSFLDAVIKPVHEIIAAEAKFCKDRKDRKGRKERMLPHSKWRNYDDINEYFWTPVCFELSWPWRLS

SGFFVKPKQDSNKKINKIKFRNTRDEIPLLQEQNQRSEPGHRREKKAGKSHFVEHRSGLHLYHSFHRLWIFLVCMLQGLA
VFAFCDGKFNAASIKYILSVGPTFVIMKFIQSVLDVILIIGAYRSSRARILSRIWLRLFWFASLSAIIVLLFVKTIQEEESRSGQS
TFFRLYCVLLILYGGSHLFVALLMNMPWLRSHAEKWSNFGPVPFLNWVHQEQYYVGRGLYESTGDYFSYILFWIVVLACK
FSFSYFLQIKTMVGPTRTIVNLTNLNYRWRDVVSKSNHNALTLVSLWAPVVMIYFLDLQVWYTVISALVGGLDGARMGL
GEIRSLDMFRNRFTYLPAAFTKRLQATVLRESENTNEKVEAMQFAPIWNDVIASLREEDLINNKEMEWLVMPGSTLTNLT
ANSQDLTLVQWPLFLLANKVFVACDLAEVHKQAPQLELWKIIGRDPYMRFALQEAFDTLRIILEHLLENDDLGRRWVNF
VYGNMREAIELKQLKDKYNLKKDQLEKVLVKAAKLTTVLINEAKKLKDEHTKEMREAYTAAVRNELLDFYDVVMRDFIKD
SETRDDAVRQAGKQSEGLFTELTLPTDESNSLVRRLNQILTIKESALNVPVNLEACRRLEFFSNSLFMKMPKSPTVRKMFS
FSVFTPYYAEDVIYSPSKLAEENKDGISMMYYLRTIVPDEWANFLERIFPKKEDRELRKALLKTIFPKEYEFKGNEKPRKPDD
LKESVKLQLRLWASYRGQTLARTVRGMMYYKRALILQAKLEGASLSDDVEQGSQYSTTSASPDLGVLDARAQAELKFLYV
VSAQIYGEQQQGGKGDDGRQKAADISYLMRTNDSLRIAYIHKGKVKQGEEEVTKYYSKLMKADPSGKDQEIYSIELPGEV
ILGEGKPENQNHAIVFTRGEALQTIDMNQEHYLEETFKMRNLLEEFDDDTKNYGHRKPTILGVREHVFTGSVSSLAWFM
SLQERSFVTLGQRVLANPLKVRMHYGHPDVFDRIFHITRGGISKASHQINLSEDIFAGFNSTLRQGNVTHHEYIQCGKGR
DVGLNQIAAFEGKVASGNGEQTLSRDIYRLGQLFDFFRMLSFFFTTVGYYFTTMLTVLTVYVFLYGKVYLALSGVDADLKS
RGLSANIALQSALDTQFLLQIGVFTAVPMIMNFLLEEGILKAIISFCTMQLQLSSVFFTFSLGTRTHYFGRTILHGGAKYAST
GRGFVVEHISFAENYRMYSRSHFVKALEIMLLLIVYLAYGAPERTTFTYILLTFSSWFLAISWLWAPFIFNPSGFEWQKKSYI
CIFTLYLFRILLSITYIVLLFGLQFLHPGLCPTSKTGVSDFEDWTNWLFHKGGIDEKGGNSWEKWWEEEQEHICTTRGQF
WEIVLSLRFFLVQYGVVYALNVVGHDRNFRVYGFSWCVLAGVVLTFKVFSINQKSFANFQLILRLFQMIAFLALIGGVVVA
VAMTSLTIGDVFASALSLIPTGWGLLSIAIALRPAVKKIKLWKSVRAIARLYEAFMGAIVFIPIALLSWFPFVSTFQTRLVFNQ
AFSRGLEISTLLSGNNPNKDM

>TMAJ 2013573 Neckera douglasii 1972

MVSGGRPEAGATPASRRLPRTYTAGGLTEVFDSEVVPSSLASIAPILRVANEIEASSPRVAYLCRYHAFEKAHRIDSTSSGRG
VRQFKTALLQRLERDSEPTLALRHRRSDAREIQSYYQNYYNDYVKSLDGAEHSDRAQLARAYQTAAVLFEVLKAVNRDKA
EEPPPEIIAAAADVEQKKEIYVPYNVLPLDAAGSSQAIMQLDEVKAAVEALRNVRGLPWQTLKESHPRAGDLDCLDWLQ
DMFGFQKDNVANQREHLTLMLANVHIRLLPRPDPMHKLDDRALNAVSNKLFKNYKSWCKFLGRKNNLWLPQIHQEV
RQRKILYMGLYLLVWGESANLRFMPECLCYIYHHMASELHGMLAGNVSMVTGDNMKPAYGGEDESFLTLVVTPIYKVIS
KETLKNRNGTAPHSAWRNYDDLNEHFWKVDCFRMGWPMRPDADFFVPDQTFLNTTEVTNGKVFQSTSKSFFVEIRTF
WHLFRSFDRLWTFYILGLQAMIVLAWNVGRNLEDAFNGSVVKQVLSIFITASILRLIQAFLDIVFGYHAFRSIKLFGEFRLVL
KLLTSAAWVIILTICYVRTWVNPQGLIAEIQKWFGESLESSYLYIAAVLLYLIPNFIGACFFLFPMIRRWIESSRWTVVLLWW
STYDQPQPRLYIGRGMHESQLALLGYTVFWVLLISCKFSFSYFIQIEPLVAPTRAIMQQTSLSYTWHEFFPNARNNPGALIS
LWAPIIMVYFMDCQIWYAVFSTIFGGISGSFRRLGEIRTLGMLRSRFSSLPGAFNESLIPDDGKRARKAFSFSRQFEKVATS
KDRSKTARFSQLWNEVITSFRQEDLISDKEKDLMLVPYSSDPQLKLVQWPPFLLASKVPIALQMAKQAAETGRAADLLRKI
KNDEYMKCAVTECYESLKRVLMRFIVGDIETRLIDGLFDEVDVNVEKDTLLDNFKLGELPVLSAKFIELLELLEMNHADKE
AVDNARDLAVLKLQDMYEVVTRDMMSDSMRDIWDSSFGGLVGGQGRKSELFSSKGDEPAKVIFPTPRSQAWIEQIKRL
HLLLTERESGMDVPENLEARRRIAFFTNSLFMNMPRAPRVRNMLSFSVLTPYYKEDVVYSKENLMKENEDGISVLFYLQK
IYPDEWSNFLQRIGLETSEDPEAQIFGTTDLEDKLREWASFRGQTLARTVRGMMYYRRALELQTFLDMATENELVDGYK
VLTDASIEQKKSQRSTWSQLQAIADMKFTYVAACQMYGEQKRQGHHHATEILKLMLAYPSLRVAYIDEVEERQNEKSSK
VYYSVLVKAVNGLDQEIYRIKLPGTVSLGEGKPENQNHAVIFTRGEGLQAIDMNQDNYLEEAFKMRNLLQEFHEPHGVR
SPTILGVREHVFTGSVSSLAWFMSNQETSFVTIGQRVLASPVCLLASPLKVRFHYGHPDVFDRLFHITRGGMSKASRVINL
SEDIFAGFNSILRRGNVTHHEYIQVGKGRDVGLNQISLFEAKIACGNGEQALSRDIYRLGHRFDFFRMLSCYFTTVGYYFST
MIVVLTVYVFLYGRIYLALSGVDDSLVHSANNKPLTAALASQALVQLGLLMALPMVMEIGLERGFRAALSDFLIMQLQLAS
VFFTFSLGTKTHYFGRTILHGGAKYRATGRGFVVRHERFADNYRLYSRSHFTKAIELFLLLVVYSLYVPESSKGAVTYILITFS

MWFLVASWLFAPFLFNPSGFEWQKIVEDWDDWNKWMSNRGGIGVEGSKSWESWWDEEHEHLNYTGFIGRVVESIL
SIRFFLYQYGIVYHLNIAQSSNDLSISSMYSTGIQLPRILCIKDLIFHDSFPSFQVYGLSWLFILAVLTVLKIVSMGRDKFSADF
QLMFRLLKASLFIGSISVIAILHVKNLTVGDLFASTLAFIPTGWALLQIAQAFKPVIIRLGFWSSVKSLARGYEYSMGLLLFTPI
AVLSWFPFVSEFQTRLLFNQAFSRGLQISRILAGRKKL------------------------------------------------------------------------

>TMAJ 2017341 Neckera douglasii 1976

MATPPRQPQRISKRVLYKWEQLVYRAKMDAERKAAEEPQGLGAGAASNATVPQTLIQQAEIESILQTADELAKKSPDVA
RILCEYAYTLVQNLDPNSEGRGVLQFKTGLLSVIKQKRSKKEGEKIDRSHDVQILQEFYQRYREQNHLEQLEYEDNRRRHS
DSYDEDSTTTEQRAEVQRKMYLIAKILNEVVDALTKDGQTEEFDSDLKRTMEEDAKKVKGFKAYNILPLETPGVANVFQS
FPEVGGAAKVLWYNESELPKFPKDAVTGDLDRHLDLFDFLEYAFGFQADNVANQREHLILLLSNSQSRLSVMLDTEDKLD
DGAILDVHLKMMSNYDRWCKFLRRDSMANRAYSMQLRLFLTALYLLIWGEAANLRFLPECLCYIFHHMADELYDLLDKD
TVERSRTFLPDSSHSFLDKIVKPIYEILAAEAKICANERAPHSAWRNYDDFNEFFWAPSCFELSWPWRLEAGFFRKPRKIIY
TEADRFEDPAASEESPAMAGEKREKKVGKTHFVEHRTGFHIYHSFHRLFIFLVCMLQGLGIFAFCDRRLTIHTMKLILSVGP
TFVLMKLIQSVMDVTLMIGAYRSTRANNISRMLIRFLWFTLLSAVIVLLYMKTIEEENSGSGSDTWFKAFYLVLGIYGGLQF
FFALLLRVPWFRLQAKKCSNFYVVSFLKWVHQERYYVGRDMFERTRDYFMYTLFWFVVGTCKFAFSYFLQIEPLVGPTRT
IIGIRNVNYRWKDLISNSNHNALTLVALWAPVIMIYFLDAQVWYTLISSLVGGFDGARMHLGEIRSLDMLRSRFSSLPGAF
VKNLVPSRGGGVSQADVNVPLSAVKPGNPKVDAIRFSPLWNEVIESLREEDLINNREREWLLMPDNTTSSGTLGQHSLV
QWPLFLLANKVYVGIDIVHDNKQSFQDEIWDRMKRDPYLEFAVMEAFISLQSVLMDLLNEHGRTWVIGIYADIEQAIEV
GQLLHKFNFTEIDSVLGRIATLAEVLVAEPKKVDDDSARQEEELKMHESADRALVDLYEAVMRDFLADSELREKYESDTLL
QTSKQDGRLFSDLTWPTGPAKEQVRRLHYILAIKDSALNVPVNLEARRRLQFFSNSLFMSMPQPRPVRNMFSFSVFTPY
YSEDVMYSKAQLEDTNVDGITILYYLQTIVPDEWKNFLERMLPGVEYNQLGHYSEADVTEADTLQLRLWASYRGQTLAR
TVRGMMYYKKALVLQAQQEGASVAEDEEEGHDIEGNELAIVNVSTPRTPSGSLVRNARAQAELKFCYVVSAQIYGKQK
NSLLQADKDRAADILYLMHMNDSLRVAYIHEAKQMIDGKPFTEYYSRLIKADPSGKEQEVYSIKLPGEVTLGEGKPENQN
HAIVFTRGEALQTIDMNQEHYLEETLKMRNLLEEFDSKSLGLNRPTILGVREHVFTGSVSSLAWFMSLQERSFVTLGQRV
LAKPLKVRMHYGPDVFDRIFHITRGGISKPSKQINLSEDIFAGFNSTLRQGNITHHEYIQCGKGRDVGLNQIAAFEGKVAS
GNGEQTLSRDIYRLGQLFDFFRMLSFFFTSVGYYFTTMLTVLTIYVFLYGKVYLALSGVDEALKASDLLDNTALQAALYTQFL
LQIGIFTTVPMIVNFILEQGVLRAVISFFTMQLQLSSVFFTFSLGTRTHYFGRTILHGSAKYKATGRGFVVEHIPFAENYRAY
ARSHFVKGMEIIMLLIVYLVYGAHDRTAASYILLTFSSWFLALSWLYAPYIFNPSGFEWQKTVIDFDDWTNWLFHKGGIG
DEGKKSWEVWWDEEQAHIKTPRGKFWEILLSTRFFIFQYGVVYALNAAGNNKSFWVYGYSWVVIVGVFLLFKIFTFSQK
ASANFQLIVRLLQGVVFLAVVAGLSVAVVLTRLTIGDVFASALAIIPTGWGLLSIAIALRPLVQWLGIWKSVRGIARLYDAA
MGMILFIPIALFSWFPFVSTFQTRLVFNQAFSRGLEISVLLAGNNPN------------------------------------------------------------
---------

>TMAJ 2020682 Neckera douglasii 1455

MGDLVYNIIPVDDNAAATGHPALEFPEVKAAVRALRSIGDLRRPPQWRTGISVDVLDWLGCWFGFQDSNVKNQREHL
VLLLANAQMRATPESADKLDGKVLRKIRRKVTKNYESWCTFVGKENVLRLPFGKQAGAERQELLYTSLFLLIWGEAANLR
FMPECLCFIFHHMAQELNKMLERDAFENFKPTTCAHNGFLRLVVSPLYKVVAAEAAVNSKGNAAHSSWRNYDDINEYF
WSERCFTHLKWPMDGGSNFLVKPEDVSKGAKHKVGKTGFVEQRSFFNIFRSFDRLWIGYILILQACIVTLWSGQQRAP
WVELRNKDSLARLLTIFITWSALRLILALFDLVMMHSLISWETWRTGLRMILKVLVASIWVLIFSIFYSSMWNRRHKDHA
WSSAANSLFVKYLYTMGAFCLPEGLALALFIIPFVRNFLEKSRFRVFHLLTWWFQSRTYVARGLREGIVDNFKYTLFWTLV
LASKFLFSYFLQLKPLIRPTKEILEITTIDYKWHQIFGKGNRVAVLALWAPVILIYFMDTQIWYTIWSALVGALVGLLDHLGEI

RNVHQLKLRFQMFPGAIQFNLIPETWGAGIREPYSWWVNVKNFFMRIRLRYGAPSQEAKEESMEARRFSHIWNEILKT
FREEDLISNRELELLEIPTPVWNISVFQWPSTLLANEVQTALDLLKKEDGGEGMQDKAVWKKIVKSEYGRCAVVESYESIK
HILVYRILRLNSPDQIMVKSLFEDHIDRAVNQRKFSDAFTLSNLPEVHEYVLTLVKNILKLKTDEVVKALQTLWHYVVNEFS
KVEERALIKENFVRQRLSTATVFKDSVVLPGKGDINFYKQLKRLQTTLETKDTLLKVPKGLEARRRISFFANSLFMTMPRAP
QVDKMFAFSVLTPYYKEEVIYSLKELNKTNEDGIDTLFYLRTVFDDDWKHFKERFSGVEETDEQFIDRMSGQGDAAEGG
SQMKHDSKAKKKLGNEKTIEEDGLELCLWASYRGQTLARTVRGMMYYERALECQAFLDAATEKDLHEALGFKEMIARA
SSNVSEERISDDTRERQVLATAAMKFTYVVAAQIYGDQKKKKENQAKGIAYLLKTYKGLRIAYVDEVETPTGNQYFSVLVK
HDRAANMEVEIFRVELPGNMKLGEGKPENQNHALIFTRGDAVQTIDMNQEMYFEEALKMRNLLQEFDKRHGVRKPTI
LGVREHVFTGSVSSLAWFMSAQETSFVTLGQRVLANPLKIRMHYGHPDVFNRLWFMSRGGISKASATINISEDIFAGFN
CTQRGGTVTHHEYIQAGKGRDVGLNQIALFEAKVASGNGEQVLSRDVYRLGHRLDFFRMLSFYYTTVGFFINNLMVVLT
VFAFLWGRFYLAVSGIESSLSNSKVLSNTALLASLNQQLIVQLGILTALPMIVENALEHGFTKALWEFFTMQLQLASVFFTF
SMGTRAHYFGRTLLHGGAS-----------------------------------------------------------------------------------------------------------------
------------------------------------------------------------------------------------------------------------------------------------------------------
------------------------------------------------------------------------------------------------------------------------------------------------------
------------------------------------------------------------------------------------------------------------------------------------------------------
-----------------------------------------------------------------------------

>TMAJ 2020683 Neckera douglasii 1772

MGDLVYNIIPVDDNAAATGHPALEFPEVKAAVRALRSIGDLRRPPQWRTGISVDVLDWLGCWFGFQDSNVKNQREHL
VLLLANAQMRATPESADKLDGKVLRKIRRKVTKNYESWCTFVGKENVLRLPFGKQAGAERQELLYTSLFLLIWGEAANLR
FMPECLCFIFHHMAQELNKMLERDAFENFKPTTCAHNGFLRLVVSPLYKVVAAEAAVNSKGNAAHSSWRNYDDINEYF
WSERCFTHLKWPMDGGSNFLVKPEDVSKGAKHKVGKTGFVEQRSFFNIFRSFDRLWIGYILILQACIVTLWSGQQRAP
WVELRNKDSLARLLTIFITWSALRLILALFDLVMMHSLISWETWRTGLRMILKVLVASIWVLIFSIFYSSMWNRRHKDHA
WSSAANSLFVKYLYTMGAFCLPEGLALALFIIPFVRNFLEKSRFRVFHLLTWWFQSRTYVARGLREGIVDNFKYTLFWTLV
LASKFLFSYFLQLKPLIRPTKEILEITTIDYKWHQIFGKGNRVAVLALWAPVILIYFMDTQIWYTIWSALVGALVGLLDHLGEI
RNVHQLKLRFQMFPGAIQFNLIPETWGAGIREPYSWWVNVKNFFMRIRLRYGAPSQEAKEESMEARRFSHIWNEILKT
FREEDLISNRELELLEIPTPVWNISVFQWPSTLLANEVQTALDLLKKEDGGEGMQDKAVWKKIVKSEYGRCAVVESYESIK
HILVYRILRLNSPDQIMVKSLFEDHIDRAVNQRKFSDAFTLSNLPEVHEYVLTLVKNILKLKTDEVVKALQTLWHYVVNEFS
KVEERALIKENFVRQRLSTATVFKDSVVLPGKGDINFYKQLKRLQTTLETKDTLLKVPKGLEARRRISFFANSLFMTMPRAP
QVDKMFAFSVLTPYYKEEVIYSLKELNKTNEDGIDTLFYLRTVFDDDWKHFKERFSGVEETDEQFIDRMSGQGDAAEGG
SQMKHDSKAKKKLGNEKTIEEDGLELCLWASYRGQTLARTVRGMMYYERALECQAFLDAATEKDLHEALGFKEMIARA
SSNVSEERISDDTRERQVLATAAMKFTYVVAAQIYGDQKKKKENQAKGIAYLLKTYKGLRIAYVDEVETPTGNQYFSVLVK
HDRAANMEVEIFRVELPGNMKLGEGKPENQNHALIFTRGDAVQTIDMNQEMYFEEALKMRNLLQEFDKRHGVRKPTI
LGVREHVFTGSVSSLAWFMSAQETSFVTLGQRVLANPLKIRMHYGHPDVFNRLWFMSRGGISKASATINISEDIFAGFN
CTQRGGTVTHHEYIQAGKGRDVGLNQIALFEAKVASGNGEQVLSRDVYRLGHRLDFFRMLSFYYTTVGFFINNLMVVLT
VFAFLWGRFYLAVSGIESSLSNSKVLSNTALLASLNQQLIVQLGILTALPMIVENALEHGFTKALWEFFTMQLQLASVFFTF
SMGTRAHYFGRTLLHGGASYKATGRGFVVKHERFAVIYRLYSRSHFVKAIELIALLIVYLIYGAHRSSSTYLLISLTSWFMALT
WLLGPFIFNPSGFDWLKTLEDFEDFIGWLKYKGGVIVDAEQSWERWWMDEQMHLKTTGLWGKLMDIILNLRFFFFQY
GIVYQLKIAATSQSIFVYIVSWSYMFVAGVIHMIIASAGQRYSTKKHGLYRAIQAILITLVIGAIVLLKVFTHFSLKDLLTSLLAF
VPTGWGILQILTVLRTDWLEKSAIWPIVVNVGRLYEFGMGLIVMAPVAILSWLPGFQAMQTRVLFNEAFSRGLHIRQLLV
TVQKAKKSE-------------------------------------------------------------------------------------------------------------------------------------
------------------------------------------------------------------------------------------------------------------------------------------------------
------------

>TMAJ 2025002 Neckera douglasii 2004

TKRSSERSKSYVVPQSLAAQTDIDAVMEAADAIKEENTEVARILYEYAYNLASQMDPGNEGRGVLQFKSALKAVLLQKRT
KHHIDRSQDVRLLTEYHIMLKMRDKIVDLDEEEQAAREGRVLGNETPEYQAWRAKRLRKFYAVANILNLAANFLAPAEPE
VGSRVRDPAKKALDADARKIEQFKAYNILPLESAGVNNSFQSFPEVVAATRALYTNEWSQFPQFRANYSKEVGRDVLDIF
DFLHYTFCFQKDNVSNQREHLILLLANAASREGTLSEGTALAHNTKLDVKSIEKVHDRILANYVRWCNFLNKKPQTKLAM
NPQKQLCLTALYLLIWGEAANVRFLPECLCYIFHNMAGECFTLLEKDFVERSTVTIKIPESATPVDNEDKLYEYAFLKQIITPIY
DVVAAEAKNSADGKAPHGSWRNYDDFNEYFWQSSCFDLKWPWRLEAGFFTKPKKKEKKATSSGGDSLGPDPRRERR
VGKINFVEHRSSFHLYHSFHRLWILLACMFQILAIWAFCSKNGNLNLHVRTIKKILSVGPTFAVMKFSKSILDVAFMWGAI
RNTHKRIVTRTLIRLAWLACLSGGIIYLYVKTLEEDARISASTPWFRLYNLVLGSYAGAQVLFTFILRLPFLRKQVDRCSNVR
MCQFIKWMQQERYYVGRGMYERTSDYVKYSLFWIVVLACKFAFTMHFQIMPMVEPTRLIIDFNNITYKWHSFISKGNN
NIFTIVSLWAPVVMIYVLDVQVWYTVASALLGGLVGARDKLGEIRSLEMLRKRFLDYPAAFVEHMDPSSSSSTPTRQEFP
RQERSTEDSKAIQNKKKAIQNKKDAIQNKKDARRFLPIWNAVINSLREEDLLNNRERAMLEMPPNGDTYPNGETDSVM
CWPLFLLANKLHIAVDLGAENKEKENNYSQEELWGKVIADEYMEFAIKESFLTLEQLLLSVLRSNDKALRWIRDIFDSVRES
VKLTSFVSNYRLQKLPEAVKIIVDLTQQLGEDEAESPACRKKAMEALNKLAKLVMIDLLKKEDSDRLAYFQEAIQQEKLFNG
LHWPDVEWRKRAIRLHSILKVHKFKDEVDGKQKTYNTESIPKNLEARRRLQFFTNSLFMHMPDAKPVSKMFSFCVFTPY
YEEDVLYDLKNKDGKKNDGEKSKKDEIKELDRKNEDGITILFYLQKIYPDEFKNFLERLQLTGDEFKRQLENPTNATNADKK
PDTRLELRLWASYRGQTLARTVRGMMYYKKALELQAEQERISDPDLERGGPSSSVRLQRGLSLRSPQAQVELKFVYLISC
QKYGDQKRQEQKDREEKKKGKSQAADILYLMQMNESLRVAYVDQVIVDTGPQAKTTSYYSKLVKVDKTDKTKDQLIYSV
KLPGPFKIGEGKPENQNHAIIFSRGDAVQTIDMNQDNYLEEAFKMRNLLEEFGEIHGRNRPTILGVREHVFTGSVSSLAW
FMSMQESSFVTLGQRVLARPLKVRMHYGHPDVFDRIFHFTTGGVSKASSGINLSEDIFAGFNTTLRKGNVTHHEYIQVG
KGRDVGLNQIATFEAKVASGNGEQALARDLYRLGQLLDFPRMLSFFFTSVGFYVTTMMTVLTLYAFLYGKAYLALSGVDA
SLKANNDILQNSALQSVLTSQFLFQIGLFTAVPMIVNLVLEQGLIKAILSFCTMQLQLASVFFTFSLGTRTHYFGRIILHGGA
KYRSTGRGFVVRHINFAENYRLFSRSHFTKAFEIILLLVIYLAYGAQDRTSVTYILLTFSSWFLSFSWLYAPYIFNPSGFEWQKT
VEDFDDWTNWILYKGGVGVKSESSWEAWWLEEQAHLRTVGGKFWEFVFSLRFFFFQYGVSYHLNVFEGSTSIMVYVY
SWLTLTVFVAIFKVFTISQKASTRKAKLHLVVRLFQAALFIFLIAGAIVAIFVSPLSLTDCFAVALAIVPTGWGLISIAIIFRDQVE
SIGLWHSVREIARLYDACMGMFIFVPIAVLSWFPFFSTFQTRLVFNQAFSRGLEIHLILAGNRANTST------------------------
----------------

>TMAJ 2025003 Neckera douglasii 1991

ISRRVLRNWEQLVRAAVTADLETGGGKSDWSDSEQGDGEEDDGLERMPRRTTTKRSSERSKSYVVPQSLAAQTDIDAV
MEAADAIKEENTEVARILYEYAYNLASQMDPGNEGRGVLQFKSALKAVLLQKRTKHHIDRSQDVRLLTEYHIMLKMRDKI
VDLDEEEQAAREGRVLGNETPEYQAWRAKRLRKFYAVANILNLAANFLAPAEPEVGSRVRDPAKKALDADARKIEQFKAY
NILPLESAGVNNSFQSFPEVVAATRALYTNEWSQFPQFRANYSKEVGRDVLDIFDFLHYTFCFQKDNVSNQREHLILLLA
NAASREGTLSEGTALAHNTKLDVKSIEKVHDRILANYVRWCNFLNKKPQTKLAMNPQKQLCLTALYLLIWGEAANVRFLP
ECLCYIFHNMAGECFTLLEKDFVERSTVTIKIPESATPVDNEDKLYEYAFLKQIITPIYDVVAAEAKNSADGKAPHGSWRNY
DDFNEYFWQSSCFDLKWPWRLEAGFFTKPKKKEKKATSSGGDSLGPDPRRERRVGKINFVEHRSSFHLYHSFHRLWILLA
CMFQILAIWAFCSKNGNLNLHVRTIKKILSVGPTFAVMKFSKSILDVAFMWGAIRNTHKRIVTRTLIRLAWLACLSGGIIYL
YVKTLEEDARISASTPWFRLYNLVLGSYAGAQVLFTFILRLPFLRKQVDRCSNVRMCQFIKWMQQERYYVGRGMYERTS
DYVKYSLFWIVVLACKFAFTMHFQIMPLVDPTRLIIGFNDIKYKWPDFVSDSNHNALTIVSLWAPVVMIYFLDTQVWYTV
ISAFLGGIEGARDKLGEIRTLEMLRKRFPNYPAAYVKHMQPPINRLSSASPSDHSASRAKPKKLDAIRFQPIWNHVIKSLRE
EDLINNREKVLLKMPPNLMFHSNGASNDLIHWPLFLLANKVHIAVELAVEHKTHQDGLWEKVRRDEYMAYAVQETFET

LEPLLVSVLNPAGARWVNEIFGRLRYSLGSADLRDDFNLNKLRDVLEKLRDLTEHLGNEDVDERRGKATRCFFQLYDVVM
HDFLSAKTREEFEETPGFQEAVQNGLLFTELNWPNKAGQKQAKRLNNLLTVQKIKDQEGKTKTLNTETIPHNLEARRRL
QFFTNSLFMHMPEAPPIRKMFSFCVFTPYYEEDVMYDMEKLSQENEDGITILFYLQKIYPDEWQNFLERIGLIENIVFREI
ENRKSERHEKVKLDLRLWASYRGQTLARTVRGMMYYKAALIIQGMQEGASGGDLEEGIPPSLVEAQGSINRSAEAQAEL
KFTYVVTCQIYGEQKRKGKVQAADILYLMQKHESLRIAYIDVVESSKKGRKPSYYSKLCKVDRSDPSQKDQEVYSIKLPGSIK
LGEGKPENQNHAIVFTRGDCIQTIDMNQDNFMEEAFKMRNLLEEFSQTHHGLHKPTILGVREHVFTGSVSSLAWFMS
MQESSFVTLGQRVLARPLKVRMHYGHPDVFDRVFHITRGGISKASRVINLSEDIFAGFNTTLRLGNVTHHEYIQVGKGRD
VGLNQIALFEAKVASGNGEQTLSRDVYRLGQLLDFPRMLSFFFTSIGFYVTTMMTVLTLYAFLYGKAYLALSGVDAALKRN
SEILQNPSLEAVLNTQFLFQIGIFTAVPMIVNLILEQGILKAIISFCTMQLQLASVFFTFSLGTRTHYFGRTILHGGAKYRSTG
RGFVVTHIHFAENYRSYSRSHFTKALEIIMLLIIYLAYGAQNRTSVTFVLLTFSSWFLALSWLFAPYIFNPSGFEWQKTVEDF
DDWTNWMFYKGGVGVKIENSWEAWWFDEQDHIRSLRGRFWEIFLSLRFFLFQYGVVYSLRVTKGSSSILVYAYSWFVL
LGLVIIFKVFTVSQKASANFQLAVRLFQGLLFVCLIAGVVVAIVLSPLTVGDVFALALALIPTGWGLLSIAIALRPLMERMRF
WKSVREIARVYDACMGMIIFIPIAFLSWFPFVSTFQTRLVFNQAFSRGLEISLILSGKGPNR---------------------------------
-----------------

>TMAJ 2025004 Neckera douglasii 1978

MDRPPRPPHRMSKRVLVKWEKLVEKALVTSSYNRDGRGRGSNSRQRGERAPQSLAQQANIDAVLQAADEIGHDNLQV
GRILTEHAYRLTQQLDPRSEGRGVLQFKTGLKSIIKQKQQARQGEVVDRSQDIRIIQIYYKQYREKHHIDQLEEEARNRRPV
LSTELPESQDQSFEKLRKVYEISTILNDVVDALLKEAEPEDAFRLQDTAEKRVLEEDAKKLKGFKAYNILPLKTPLGAPSVLN
PFDFFPEVVGATQVLIYTRDLPRFPSDYEVPEDRVVDIFDFLHYAFGFQKDNVANQREHIILLLSSAQSRLGTLDQGRDSD
GNKTLLNPDKAVSNVHERILENYVRWCHFLRREPQNKRAFTQQRRLILAALYLLVWGEAANLRFMPECLCYIFHNLADE
CIDLLGRTYVERSKIVKANEDGSIEFSFLDQIITPVYDAVAAEAESSQGGKVPHSNWRNYDDFNEYFWQPNCFVELGWP
WRTDAGFFKPPKMKDAMKVPLPQIEANLPEAAGRRRKHKVGKVHFVEHRSGFHIYHSFHRLWIFFICMLQGLTVWAFC
SKNGNLNLHVRTIKRIMSVGPTFVIMKFIQSVFDIVFMWGAFKATRLQTVARMLLRFLWFTCLSAAVLFLYIKTLEEDARN
DGSGSWFRIYYILISSFAGANAVFVFLLRIPFLQRQGAKCSNVYLFQFVKWLYQERYYVGRSMYERTRNYIKYSIFWIVILAC
KFAFTMHFQIMPLVDPTRLIIGFNDIKYKWPDFVSDSNHNALTIVSLWAPVVMIYFLDTQVWYTVISAFLGGIEGARDKL
GEIRTLEMLRKRFPNYPAAYVKHMQPPINRLSSASPSDHSASRAKPKKLDAIRFQPIWNHVIKSLREEDLINNREKVLLKM
PPNLMFHSNGASNDLIHWPLFLLANKVHIAVELAVEHKTHQDGLWEKVRRDEYMAYAVQETFETLEPLLVSVLNPAGAR
WVNEIFGRLRYSLGSADLRDDFNLNKLRDVLEKLRDLTEHLGNEDVDERRGKATRCFFQLYDVVMHDFLSAKTREEFEET
PGFQEAVQNGLLFTELNWPNKAGQKQAKRLNNLLTVQKIKDQEGKTKTLNTETIPHNLEARRRLQFFTNSLFMHMPEA
PPIRKMFSFCVFTPYYEEDVMYDMEKLSQENEDGITILFYLQKIYPDEWQNFLERIGLIENIVFREIENRKSERHEKVKLDLR
LWASYRGQTLARTVRGMMYYKAALIIQGMQEGASGGDLEEGIPPSLVEAQGSINRSAEAQAELKFTYVVTCQIYGEQKR
KGKVQAADILYLMQKHESLRIAYIDVVESSKKGRKPSYYSKLCKVDRSDPSQKDQEVYSIKLPGSIKLGEGKPENQNHAIVF
TRGDCIQTIDMNQDNFMEEAFKMRNLLEEFSQTHHGLHKPTILGVREHVFTGSVSSLAWFMSMQESSFVTLGQRVLA
RPLKVRMHYGHPDVFDRVFHITRGGISKASRVINLSEDIFAGFNTTLRLGNVTHHEYIQVGKGRDVGLNQIALFEAKVAS
GNGEQTLSRDVYRLGQLLDFPRMLSFFFTSIGFYVTTMMTVLTLYAFLYGKAYLALSGVDAALKRNSEILQNPSLEAVLNT
QFLFQIGIFTAVPMIVNLILEQGILKAIISFCTMQLQLASVFFTFSLGTRTHYFGRTILHGGAKYRSTGRGFVVTHIHFAENYR
SYSRSHFTKALEIIMLLIIYLAYGAQNRTSVTFVLLTFSSWFLALSWLFAPYIFNPSGFEWQKTVEDFDDWTNWMFYKGG
VGVKIENSWEAWWFDEQDHIRSLRGRFWEIFLSLRFFLFQYGVVYSLRVTKGSSSILVYAYSWFVLLGLVIIFKVFTVSQKA
SANFQLAVRLFQGLLFVCLIAGVVVAIVLSPLTVGDVFALALALIPTGWGLLSIAIALRPLMERMRFWKSVREIARVYDAC
MGMIIFIPIAFLSWFPFVSTFQTRLVFNQAFSRGLEISLILSGKGPNR-------------------------------------------------------------
------

>VBMM 2004118 Anomodon rostratus 2022

MASGGRLECGAVDLPRRLSRTYTAGGLTEVFDSEVVPSSLASIAPILRVANEIEASSARVAYLCRYHAFEKAHLFDPTSSGR
GVRQFKTALLQRLERDSEPTLALRHRRSDAREIQSYYQNYYNDYVKALDGAEHSDRAQLARAYQTAAVLFEVLKAVNRDK
AEEPPPEIIAAAADVEQKKEIYVPYNVLPLDAAGASQAIMQLDEVRAAVEALRNVRGLPWLTEKESHSRAGDLDCLDWL
QDMFGFQKDNVANQREHLILMLANVHIRLLPRPEPMHKLDDRALNAVMNKLFKNYKSWCKFLGRKNNLWLPQIHQE
VRQRKILYMGLYLLVWGEAANLRFMPECLCYIYHHMASELHGMLAGNVSMVTGDNMKPAYGGEDESFLTFVVTPIYKV
ISKETLKNRNGTAPHSAWRNYDDLNEYFWKGDCFRMGWPMRPDADFFVPAQTSSSTNDIHGKDYQSSSKSFFVEIRSF
WHLFRSFDRLWAFYILGLQAMIVLAWNVGPNLQNAFTGAVIKQILSIFITASILRLIPFFALFKASCISFRRGLNFYHRHILISL
SESLRIIYIGLFVLSIFQTYNNAVHSVLLYAAFLDIVFCYHAFRSIKLLGVLRLVLKLFTSAAWVIVLTISYARTWVNPQGLIGEI
QKWLGKSLESSYLYVVAVVVYLIPNFIGAFFFLFPMIRRWIESSNWTVVRVLLWWSQPRLYIGRGMHESQIALFGYTFFW
VLLIASKFAFSYFIQIEPLVAPTKAIMQQSSVIYTWHEFFPYYATLAIVSISYDLDRMLMSYEHELTALKLNVARNNPGALISL
WAPVIMVYFMDSQIWYAVFSTIFGGISGSFRRLGEIRTLGMLRCRFSSLPGAFNESLVPDDGKRARKGFSFSRDFEKISPS
KDRLKAARFSQLWNEVITSFRQEDLISDKERDLMLVPYSSDPHLKLVQWPPFLLASKVPIALQMAKQAAETGRAADLLRK
IKIDEYMKCAVTECYESLKRVLKRFIIGEVEIRVIDGLFDEVDVNVEKETLLDNFKLGELPALSAKFIELLELLEKNHAGQEAID
SARDLAVLKLQDMYEVVTRDMMSDSMRDSWDSSLGALAGGQGRKSELFSAKGDEPAKVVFPLSRNEAWIEQIKRLHL
LLTERESAMDVPENLEARRRIAFFTNSLFMNMPHAPRVRNMLSFSVLTPYFKEDVVYSKENLMKENEDGISVLFYLQKIY
PDEWSNFLQRIGLESSEDPEAQIFDCSDLEDKLREWASFRGQTLSRTVRGMMYYRRALELQAFLDMATEDELVDGYKVL
TDATPEQKKSQRSTWSQLQAIADMKFTYVAACQMYGEQKRRGHHSATEILKLMLNNLSLRVAYIDEAEERQDEKPRKVY
YSVLVKAINGLDQEIYRIKLPGIVRLGEGKPENQNHAVIFTRGEGLQTIDMNQDNYLEEAFKMRNLLQEFHEPHGVRPPT
ILGVREHIFTGSVSSLAWFMSNQETSFVTIGQRVLASPLKVRFHYGHPDVFDRLFHITRGGMSKASRVINLSEDIFAGFNSI
LRRGNVTHHEYIQVGKGRDVGLNQISLFEAKIACGNGEQALSRDIYRLGHRFDFFRMLSCYFTTVGYYLSTMIVVLTVYVF
LYGRIYLALSGVDDSLVHSANNKALTAALASQALVQLGLLMALPMVMEIGLERGFRTAFSDFLTMQLQLASVFFTFSLGTK
THYFGRTILHGGAKYRATGRGFVVRHERFADNYRLYSRSHFTKAIELFLLLIVYTLYVSTSAKGAVTYILITFSMWFLVASWL
FAPFLFNPSGFEWQKIVEDWDDWNKWMSNRGGIGVEGSKSWESWWDEEQEHLNYTGAFGRFVEVLLSIRFFLYQYGI
VYHLNIARSSKDLSISVYGLSWLVIVAVLTVLKIVSMGRDKFSADFQLLFRLLKASLFIGSVSVIAILHVKSFTVGDLFACILAFI
PTGWALLQIFQACKPVVTRLGFWKSVKALARGYEYLMGLLLFTPIAILSWFPFVSEFQTRLLFNQAFSRGLQISRILAGRKK
L----------------------

>VBMM 2004436 Anomodon rostratus 1593

KHPFHERSIILKDFDSFLDAVIKPVHEIIAAEAKFCKDRKDRKLPHSKWRNYDDINEYFWTPICFELSWPWRLSSGFFVKPK
QDSNKKRNKLKFRNTRDEIPLLQEQNQRSEPGHRRERKAGKSHFVEHRSGLHLYHSFHRLWIFLVCMLQGLAVFAFCDG
KFNAASIKYILSVGPTFVIMKFIQSILDVTLIIGAYRSSRARTLSRIWLRLFCFASLSAIIVLLFVKTIQEEESRSGRSAWFRLYCV
LLILYGGSHLFVAVLMNMPWLRRQAEKCSNFGPVPFLNWVHQERYYVGRGLYESTGDYFSYILFWIVVLACKFSFSYFLQI
KTMVGPTRTIVNLTDLNYRWRDVISKSNHNALTLVSLWAPVVMIYFLDLQVWYTVISALVGGLDGARMGLGEIRSLDML
RDRFTFLPAAFTKRLQAHQHKVLRESVNTNEKVEAIQFAPIWNDVIASLREEDLINNKEMEWLVMPGNTLTNLTGNSQD
LTLVQWPLFLLANKVFVACDLAEVHKQAPQLELWKIIGRDPYMRFALQEAFDTTRIILEHLLENDYLGRRWVNFVYGNM
REAIEREQLKHKYNLKKDQLEKVLVKAAKLTTVLINEAKKLKDDDTKEMREEYTAAVRKELLDFYDVVMRDFIKDSETRDD
AVLQAGKQSEGLFTELTLPTDESNSLVRRLNQILTIKESALNVPVNLEACRRLEFFSNSLFMKMPNAPTVRTMFSFSVFTPY
YAEDVIYSPSKLAEENKDGISMMYYLRTIVPDEWANFLERIFPKKEDREARKALLKTIFPKEYELKGIEKPRKPDDLNETIKL
QLRLWASYRGQTLARTVRGMMYYKRALILQAKLEGASLSDDVEQGSQYSTTSASPQGVLDAGAQAELKFLYVVSAQIYG
EQQQGGKGDDGRQKAADISYLMRTNDSLRIAYIHKGKVKQGSEEVTKYYSKLMKADPSGNDQEIYSIELPGEVILGEGKP

ENQNHAIVFTRGEALQTIDMNQEHYLEETFKMRNLLEEFDDTKNYGLRKPTILGVREHVFTGSVSSLAWFMSLQERSFV
TLGQRVLAKPLKVRMHYGHPDVFDRIFHITRGGISKASHQINLSEDIFAGFNSTLRQGNVTHHEYIQCGKGRDVGLNQIA
AFEGKVASGNGEQTLSRDIYRLGQLFDFFRMLSFFFTTVGYYFTTMLTVLTVYVFLYGKVYLALSGVDADLKSKGLSANVA
LQSALDTQFLLQIGVFTAVPMIMNFLLEEGILKAIISFCTMQLQLSSVFFTFSLGTRTHYFGRTILHGGAKYASTGRGFVVEH
IRFAENYRMYSRSHFVKALEIMLLLIVYLAYGAPERTTFTYILLTFSSWFLAVSWLWAPFIFNPSGFEWQKTVSDFEDWTN
WLFHKGGIDEKGGNSWEKWWEEEQEHICTIRGQFWEIVLSLRFFLVQYGVVYALNVVGHDRNFRVYGFSWCVLGGVV
LKVFSVNQKSSANFQLILRLFQMIVFLALIGGVVVAVAITSLTIGDVFASALSLIPTGWGLLSIAIALKPAVKKVRMWKSVRA
IARLYEAFMGAIVFIPIALLSWFPFVSTFQTRLVFNQAFSRGLEISTLLSGNNPNKDM--------------------------------------------
---------------------------------------------------------------------------------------------------------------------------------
---------------------------------------------------------------------------------------------------------------------------------
----------------------------------------------------------------------------------------------------------------------------

>VBMM 2008307 Anomodon rostratus 1979

MATPPRQPQRISKRVLYKWEQLVYRKKMAAERKAAEEPQGLGAGAASNTTVPQTLVQQAEIESILQTADELAKKSPAVA
RILCEYAYTLVQGLDPNSEGRGVLQFKTGLLSVIKQKRSKKEGEKIDRSHDVQILQEFYQRYREQNHLEQLEYEDNRRRHS
DSYDEDSATTEQRAEVQRKMYLIARILNEVVDALTKDGQTKEFDSDLKRTMEEDAKKVKGFKAYNILPLETPGVANVFQS
FPEVGGAAKVLWYNESELPKFPEDSVPGDLDRHLDLFDFLEYAFGFQADNVANQREHLILLLSNSQSRLRVMLDTEDKL
DDGAILTVHLKMMSNYDRWCKFLRRDSMANRAYSMQLRLFLTALYLLIWGEAANLRFLPECLCYIFHHMADELYDLLDR
DTVERSRIFLPESSHSFLDKIVKPIYEILAAEAKIGAGGRAPHSDWRNYDDFNEFFWAPSCFELSWPWRLDAGFFIKPRKKI
YTEADRYEEPAASEESPAMVGEKREKKVGKTHFVEHRTGFHIYHSFHRLFIFLVCMLQGLGIFAFCDRKLTVHTIKLIMSVG
PTFVLMKLIQSVMDVTLMIGAYRSTRAHNISRMLIRFLWFTLLSAVVVLLYVKTIEEENSGSGSDTWFKTFYLVVGIYGGL
QLFFALLLRVPWFRLQAEKCSNFYVIMFIKWVHQERYYVGRNMFERTRDYFMYTFFWFVVGTCKFAFSYFLQIQPMVG
PTRTIIGIRNVKYRWKDLISESNHNALTLVALWAPVIMIYFLDTQVWYTVISSLVGGFDGARMHLGEISRSLDMLRSRFSSL
PGAFVKNLVPSRGGGVSPADENVPLSAVKPGNPKVDAIRFSPLWNEVINSLREEDLINNRERDWLLMPDNTTSSGTLGQ
HSLVQWPLFLLANKVYVGIDIVHDNRQSFQNEIWDRMKRDPYLEFAVMEAFISLQSVLMDLLNEHGRTWVIGIYADIEQ
AIEVGQLLHKFNFAEIDNVLGRIAKLAEVLVAEPKKVDDDSVRQEEELKMHESAARALVDLYEVVMRDFLADSELREKYEL
DTLLQTSKQDGRLFSDLTWPTGPAKEQVGRLHYILAIKDSALNVPVNLEARRRLQFFSNSLFMSMPQPRPVRNMFSFSV
FTPYYSEDVMYSKAQLEDTNVDGITILYYLQTIVPDEWKNFLERMLPDVEYNQLGNYNEEHVTDTLQLRLWASYRGQTL
ARTVRGMMYYKRALVLQAQQEGASVAEDEEEGHDIEGNELAIVNVSTPRTPSGSLVRNARAQAELKFCYVVSAQIYGKQ
KNSLLQADKDRAADILYLMHMNDSLRIAYIHEAKQMIEGKSFTEYYSKLIKADPSGKDQEVYSIKLPGEVTLGEIKPENQN
HAIVFTRGEALQTIDMNQEHYLEETLKMRNLLEEFDSKSLGLRPPTILGVREHVFTGSVSSLAWFMSLQERSFVTLGQRVL
VKPLKVRMHYGHPDVFDRIFHITRGGISKPSKQINLSEDIFAGFNSTLRQGNITHHEYIQCGKGRDVGLNQIAAFEGKVAS
GNGEQTLSRDIYRLGQLFDFFRMCSFFFTSVGYYFTTMLTVLTVYVFLYGKVYLALSGVDEALKASDLLENTALQSALDTQF
LLQIGVFTAVPMIVNFILEQGVLRAVISFFTMQLQLSSVFFTFSLGTRTHYFGRTILHGGAKNKATGRGFVVEHIPFAENYL
AYARSHFVKGMEIIMLLIVYLVYGAHDRKAASYILLTFSSWFLALSWLYAPYIFNPSGFEWQKTVIDFDDWTNWLFHKGGI
GDEGKKSWEVWWDEEQAHIQTPRGRFWEILLSTRFFIFQYGVVYALNAAGNDKSFWVYGYSWVVIVGVFLLFKIFTFS
QKASANFQLIVRLLQGVVFLAVVAGVSVAVVLTRLTIGDVFASALAIIPTGWGLLSIAIAIRPLVQWLGIWKSVRGIARLYDA
AMGMILFIPIALLSWFPFVSTFQTRLVFNQAFSRGLEISVLLAGNNPNAAM---------------------------------------------------
-------------

>VMXJ 2005189 Leucobryum albidum 1956

MASGGGSESGGGLPRRPPRTYTAGAVTESFDSEVVPSSLAAIAPILRVANEIESSSPRVAYLCRYHAFEKAHRIDPTSQGR
GVRQFKTALLQRLERDSEPTLALRHRRSDAREIQSYYQNYYNDYVKALDGAEHSDRAQLAKAYQTAAVLFEVLKAVNRDK
AEEPPPELICELMNMGHITSAEFLITWDEIDLFGSLGFSVADVMFEVVTWKVGKVIGGIYTNPRVQRGLALFADHCSSNG
RGAKEGNICSSQCSLDATGASHAIMQLDEKDNVANQREHLILMLANVHIRLLPRPEPMHKLDDRALNAVMNKLFKNYK
SWCKFLGRKNYHWLPQIHQEIRQRKILYMGLYLLIWGEAANLRFMPECLCYIYHHMASELHGMLAGNVSMVTGDNM
KPAYGGEDESFLKLVVTPIYEVIARETLKSRNGTAPHSAWRNYDDLNEYFWKVDCFEMGWPMRTDADFFVPAQASSNT
TKTINGKVYQSTSKSFFVELRSFWHLFRSFDRLWAFYILALQAMIVLAWNVGTHLQDAFNGTVIKQVLSIFITASILRLLQAI
LDLIFGYHAFRSIKLSGVIRLILKLITSGAWVIVLTICYVRTWDNPHGLIGTIQKWLGKSWENSYLYVAAVVVYLIPNFIGACF
FLFPMIRRWIESSNWTLIRILLWWSQPRLYIGRGMHESQFALFGYTFFWIILIASKLAFSYYIQIEPLVAPTRVIMRQTSVTY
TWHEFFPHARNNPGALISLWAPVVMVYFMDSQIWYAVFSTIFGGISGSFRRLGEIRTLGMLRSRFSSLPGAFNESLIPDD
GKRARKAFAFSRDFEKVAPSKDRSKAARFSQLWNEVITSFRQEDLISDKERDLMLVPYSSDPDLNLVQWPPFLLASKVPIA
LQMARQAAETGRTADLLRKIKNDEYMKCAVTECYESLKRVLKVLIVGEVEIRVIEGLLNQVDQNVDKETLLDNFKLGELPV
LSAKFIELLELLEKNHAGQEAVDSARDLVVLKLQDMYEVVTRDMMSDSMRDSWDSSHGALAGGQGRRSELFSSKGDE
PAKVSFPPPRSEAWIEQIKRLHLLLTERESAMEVPENLEARRRIAFFTNSLFMNMPRAPRVRNMLSFSVLTPYYKEDVVYS
KENLMKENEDGISVLFYLQKIYPDEWNNFLERIGLGTSEDPESQIFRSTDLMDRLREWASFRGQTLSRTVRGMMYYRRA
LELQTFLDMATEDELVDGYKILTDATPEQKKSQRSTWSQLQAIADMKFTYVAACQMYGEQKRQGHHSATEILKLMLNN
PSLRVAYIDEREERQNEKPSKVYYSVLVKAINGLDQEIYRIKLPGPVRLGEGKPENQNHAVIFTRGEGLQTIDMNQDNYLE
EAFKMRNLLQEFHEPHGVRPPTILGVREHIFTGSVSSLAWFMSNQETSFVTIGQRVLASPLKVRFHYGHPDVFDRLFHIT
RGGMSKASHVINLSEDIFAGFNSILRRGNVTHHEYIQVGKGRDVGLNQISLFEAKIACGNGEQVLSRDIYRLGHRFDFFR
MLSAYFTTVGYYFSTMIVVLTVYVFLYGRIYLALSGVDGSLVHSANNKALTAALASQALVQLGLLMALPMVMEIGLERGFR
TALSDFLTMQLQLASVFFTFSLGTKTHYFGRTILHGGAKYRATGRGFVVRHERFADNYRLYSRSHFTKGIELFLLLMVYSLYV
SKSAKGAVTYILITFSMWFLVASWLFAPFLFNPSGFEWQKIVEDWDDWNKWMSNRGGIGVEGSKSWESWWDEEQD
HLNYSGFIGRFVESVLAIRFFLYQYGIVYHLNIARSSHDLSITVYGLSWLVIVAVLTILKIVSMGRDKFSADFQLMFRLLKASLF
IGSVSVIAILHVKNFTVGDLFASILAFLPTGWALLQIAQACKPVVVSLGFWNSIKSLAKGYEYMMGLFLFTPVAVLSWFPF
VSEFQTRLLFNQAFSRGLQISRILAGRKKL--------------------------------------------------------------------------------

>VMXJ 2005192 Leucobryum albidum 1724

QIIAAATDVEQKKEIYVPHNVLPLDATGASHAIMQLDEKDNVANQREHLILMLANVHIRLLPRPEPMHKLDDRALNAV
MNKLFKNYKSWCKFLGRKNYHWLPQIHQEIRQRKILYMGLYLLIWGEAANLRFMPECLCYIYHHMASELHGMLAGNVS
MVTGDNMKPAYGGEDESFLKLVVTPIYEVIARETLKSRNGTAPHSAWRNYDDLNEYFWKVDCFEMGWPMRTDADFF
VPAQASSNTTKTINGKVYQSTSKSFFVELRSFWHLFRSFDRLWAFYILALQAMIVLAWNVGTHLQDAFNGTVIKQVLSIFI
TASILRLLQAILDLIFGYHAFRSIKLSGVIRLILKLITSGAWVIVLTICYVRTWDNPHGLIGTIQKWLGKSWENSYLYVAAVVVY
LIPNFIGACFFLFPMIRRWIESSNWTLIRILLWWSQPRLYIGRGMHESQFALFGYTFFWIILIASKLAFSYYIQIEPLVAPTRVI
MRQTSVTYTWHEFFPHARNNPGALISLWAPVVMVYFMDSQIWYAVFSTIFGGISGSFRRLGEIRTLGMLRSRFSSLPGA
FNESLIPDDGKRARKAFAFSRDFEKVAPSKDRSKAARFSQLWNEVITSFRQEDLISDKERDLMLVPYSSDPDLNLVQWPP
FLLASKVPIALQMARQAAETGRTADLLRKIKNDEYMKCAVTECYESLKRVLKVLIVGEVEIRVIEGLLNQVDQNVDKETLL
DNFKLGELPVLSAKFIELLELLEKNHAGQEAVDSARDLVVLKLQDMYEVVTRDMMSDSMRDSWDSSHGALAGGQGRR
SELFSSKGDEPAKVSFPPPRSEAWIEQIKRLHLLLTERESAMEVPENLEARRRIAFFTNSLFMNMPRAPRVRNMLSFSVLT
PYYKEDVVYSKENLMKENEDGISVLFYLQKIYPDEWNNFLERIGLGTSEDPESQIFRSTDLMDRLREWASFRGQTLSRTV
RGMMYYRRALELQTFLDMATEDELVDGYKILTDATPEQKKSQRSTWSQLQAIADMKFTYVAACQMYGEQKRQGHHSA
TEILKLMLNNPSLRVAYIDEREERQNEKPSKVYYSVLVKAINGLDQEIYRIKLPGPVRLGEGKPENQNHAVIFTRGEGLQTI
DMNQDNYLEEAFKMRNLLQEFHEPHGVRPPTILGVREHIFTGSVSSLAWFMSNQETSFVTIGQRVLASPLKVRFHYGH
PDVFDRLFHITRGGMSKASHVINLSEDIFAGFNSILRRGNVTHHEYIQVGKGRDVGLNQISLFEAKIACGNGEQVLSRDIY

RLGHRFDFFRMLSAYFTTVGYYFSTMIVVLTVYVFLYGRIYLALSGVDGSLVHSANNKALTAALASQALVQLGLLMALPMV
MEIGLERGFRTALSDFLTMQLQLASVFFTFSLGTKTHYFGRTILHGGAKYRATGRGFVVRHERFADNYRLYSRSHFTKGIEL
FLLLMVYSLYVSKSAKGAVTYILITFSMWFLVASWLFAPFLFNPSGFEWQKIVEDWDDWNKWMSNRGGIGVEGSKSW
ESWWDEEQDHLNYSGFIGRFVESVLAIRFFLYQYGIVYHLNIARSSHDLSITVYGLSWLVIVAVLTILKIVSMGRDKFSADFQ
LMFRLLKASLFIGSVSVIAILHVKNFTVGDLFASILAFLPTGWALLQIAQACKPVVVSLGFWNSIKSLAKGYEYMMGLFLFT
PVAVLSWFPFVSEFQTRLLFNQAFSRGLQISRILAGRKKL-------------------------------------------------------------------
-------------------------------------------------------------------------------------------------------------------------------
-----------------------------------------------------------------------------------------------------

>VMXJ 2011519 Leucobryum albidum 1725

SNQREHLILLLANAESRVGTLSEETALAHNAKLDVKAIEKVYERILANYVRWCDFLKQKPQTKLASNPQKQLCLTALYLLIW
GEAANVRFLPECLCYIFHHMAGECFTLLDKNHVERSTVTIKLTEDESPDFAFLEQIITPVYNIVAAEAKNSEHGKAPHGSW
RNYDDFNEYFWQPSCFDLKWPWRLEAGFFTQPRKKGDKTAHSSRGEATPLLRSDSMRNSRRERRVGKIHFVEHRSSLH
LYHSFHRLWVFLVCMLQVLTVWAFCSENGKLNLHVRTIKRMMSVGPTFAVMKLLKSILDVAFMWGAIRNTRKQIVYRM
LIRLVWLLCLSGGITYLYVRTLLEDARNPLSTTWFRLYYIVLCSYAGAEVLFTFILRLPFLRKLVDRCSNVRMCQFITWMQQE
RYYVGRDMYERTSDYVRYSLFWIFVLACKFAFTMHFQIMPMVEPTRLIIGFRNITYNWHDFISKGNDNALTLVSLWAPV
VLIYVLDVQVWYTVTSALLGGLEGARDKLGEIRSLDMLRKRFLDCPEAFVKNLEPPRSSVTPARQDFGRQDQSIDERRAI
QNKADARRFLPIWNAVINSLREEDLLNNRERGMLEMPSNSDTYPNGRRDTIMCWPLFLLANKLHIAVGLAADNKHGQ
TDLWLKVTADEYMKFAIQESFQTLEQLLMTVFRSNDRALRWIQDIFGDVRESVKVVAFVGIYNLQKLPQVVEIIRDLTQQ
LGEDEDENPVCRKKATTALNMLAKVVMNDLLEGKSDRLLQWGHYQEGIRNKWLFDDMHWPNEEWQKRANRLHNIL
KVHTFKDEADGKPKTYNTESIPKNLEARRRLEFFTNSLFMNMPKAIPVSKMFSFCVFTPYYSEDVMYDLKKKGGKKGEKS
KKDEIKELDRENEDGITILFYLQKIYPDEFKNFLERLKLTEREFVRQVWWDPRSNLSEETKRRNEETKLELRLWASYRGQTL
ARTVRGMMYYKKALELQAEQERISVPDLERGVPSSSSRRQRGLSMRSPQAQAELKFVYLVSCQIYGDQKKTGKPQAADI
LYLMEKNESLRVAYVDEVTVATGAQEKTPTYYSKLVKVDQMDKGKDQLIYSIKLPGQFRLGEGKPENQNHAIIFSRGDAV
QTIDMNQDNYLEEAFKMRNLLEEFDKVHGRHRPSILGVREHVFTGSVSSLAWFMSMQESSFVTLGQRVLARPLKVRM
HYGHPDVFDRIFHFTRGGVSKASAGINLSEDIFAGFNTTLRQGNVTHHEYIQCGKGRDVGLNQIATFEAKVASGNGEQA
LARDLYRLGQLLDFPRMLSFFFTSVGFYVTTLMTVLTLYVFLYGKAYLALSGVDASLKANNDILQNSALQSVLTSQFLFQIGI
FTAVPMIVNLVLEQGLFKAIMSFCTMQLQLASVFFTFSLGTRTHYFGRIILHGGAKYRSTGRGFVVRHINFAENYRLFSRSH
FTKAFEIILLLVIYLAYGAQDRTSVTYLLLTFSSWFLALSWLYAPYIFNPSGFEWQKTVEDFEDWSNWVLYKGGVGVKAEQS
WEAWWLEEQDHLRTASGKFWEVVLSLRFFFFQYGVSYHLHVFQGSTSIMVYVYSWITLLVFGFIFKVFTISRKASAKTAK
RHLVVRLFQAALFMGLIAGVIVAIVVSPLSFTDCFAVALALVPTGWGLISIAIVFRRQMEFIGVWHSVREIARLYDACMGM
IIYIPIAFLSWFPFFSTFQTRLVFNQAFSRGLEISKILAGNRANTST------------------------------------------------------------
-------------------------------------------------------------------------------------------------------------------------------
-------------------------------------------------------------------------------------------------------------

>VMXJ 2012569 Leucobryum albidum 1985

RPPRNPQRISRRVLGNWEKLVGRAVRTELLGRASYSRDGWGRGSNSGQTGMAPQSLAQQVDIDAVLQAADEIGQEN
VQVARILTEHAYRLTQQLDPRSEGRGVLQFKTGLKSIIKQRKLARHGEVFDRSQDIRVIQDYYKRYRERNNIDVLEAEAQM
RRQGWSNELTTDKSQDRSFEHLRRIYEISKILNDVVDALLEEAEPEDASRLQDTDEKRVLEEDAAKVKRFRAFNILPLEAPE
TSAILNPYEHFSEVVGATMILRYSPEMPRFPSDFVVPADRVLDIFDFLHYAFGFQKDNVANQREHLILLLASAQSRLGTLD
QSDEDKLADKAVNDVHDRVLQNYVRWCHFLRREPQSKRAFTQQRRLCLVALYLLVWGEASNLRFMPECLCYIFHSLAD

ECFDLLERTFVERSRIVKTATDGSTEYSFLEQIITPVYNVVAAEAKSSQGGKIPYSHWRNYDDFNEYFWQPSCFLHLRGNS
PDSFWNLEAGFFKPPKEKGAKKKVVILGNETEPLLRSKPKIKRRVGKVLFVEHRSGFHLYHSFHRLWIFLVCMLQGLTIWA
FCSKNGNLNLHVRTIKRIMSVGPTYCVMKLIQSLFDVVFMWGAFRSTRFQTVARMLLRLIWFACLSAAVLFLYVKTLKED
SRNDGSGSWFRIYYIVVSSYAGANVLFGFLLRIPWLQRQAAKCSNVYLFQFIKWLHQERYYVGRSMYERTRNYAKYSIFW
VFVLACKFAFAVHFQIMPLVVPTRLIIGFNNIKYKWPDFISDSNHNALTILAIWAPVVMIYFLDLQVWYTVASALLGGIEGA
RDKLGEIRTLEMLRKRFPNYPGVFVKHMQPHISGPNSDPSMQPGEFTHNKAIKEKRDAIRFQPIWNRVIKSLREEDLINN
REKLLLKMPPNLMFHPNGAPNNLVHWPLFLLANKVHIAVELAAQHKTKNQESLWDKIRLDEYMACAVQETFDTLETLL
VYVLNPDGRNWVYTIYQDIRLSLGESTFPTKFKLSALKDVLEKLRDLTEQLGNEDAQERRDKATTVLDKLFSIVMRDFIDD
EIRQRYDGWTFLQENIRSGRLFSNLNWPDKTRQKQAIRLNNLVTVQKIKDQEGKTKNLNTETVPHNLEARRRLQFFTNS
LFMHMPKAPPIRKMFSFCVFTPYYEEDVMYDMAKLNAENEDGITILFYLQKIYPDEWYNFLERIGLIENIVFREVDNPKSE
RHAEVKLQLRLWASYRGQTLARTVRGMMYYKEALIIQGKQEGASGVDLEEGIPPSLVEAQGSLARSAVAQAELKFTYVV
TCQIYGEQKRQGKVQAADILYLMQTYDSLRIAYIDRVEGGGRGTKPSFYSKLCKVDRSDPTRKDQEVYSIKLPGEVKLGEG
KPENQNHAIIFTRGDCIQTIDMNQDNFMEEAFKMRNLLDEFNQTHHGLHRPTILGVREHVFTGSVSSLAWFMSMQES
SFVTLGQRVLARPLKVRMHYGHPDVFDRIFHITRGGISKASRGINLSEDIFAGFNTTLRLGNVTHHEYIQVGKGRDVGLN
QIALFEAKVASGNGEQTLSRDVYRLGQLLDFPRMLSFFYTSVGFYVTTMMTVLTLYVFLYGKAYLALSGVDASLKRDSDIL
QNRSLETALNTQFLFQIGIFTAVPMIVNLILEQGILKAIISFLTMQLQLASVFFTFSLGTRTHYFGRTILHGGAKYRSTGRGFV
VTHIKYAENYRLYSRSHFTKALEVIMLLIVYLAYGAQNRTSVTYILLTFSSWFLALSWLFAPYIFNPSGFEWQKTVEDFEDW
TKWLFYKGGVGVKTENSWEAWWLEEQEHIRSLRGRIWEFVLSIRFFLFQYGVVYSLHVTRGSSSILVYVYSWFVLLGLVIIF
KVFSVSQKASANFSLAVRLFQGLLFTCLLAGLIVAVVLSPLTVGDVFAVALALIPTGWGLLSIAIAFRPLMERLRVWKSVREI
ARIYDACMGMIIFAPIAFLSWFPFISTFQTRLVFNQAFSRGLEIALILSGNRPNR----------------------------------------------
------------

>VMXJ 2014275 Leucobryum albidum 1772

VAGRAYNILPVDFPGDVDHAGMLFPEVKAALAALQKIWKLPVPSDQRLWTLDSDMLDWLGGFFGFQADNVRNQREH
LVLLLANRLMHMFSTPMSFNTLEANVVRTIRKKVTGNYSKWCSFIGCKNNMKLLKRRRGRQEQNEEQEEEMGRELMYI
CLFLLIWGEAANLRFMPECLCYIYHHMLADLNAVLNNFDNDIEVQGEMPSYAGPNGFLNNIVFPIYQVVKAEANSNNG
GAAPHSSWRNYDDMNEYFWSSRCFDQLRWPIRPDCSYLQEPKRERGYLNRGRKEQHHKVGKTGFVEQRSFWYIYRSF
DRIWVAHILFLQASVMTLWHNGGLPWIELQKPDPLARFLSIFITWSLLRVLQGLLDIGSQYSLVSRETLLTGVRMILKLLVA
ATWAILFIIFYRRMWWQRNIDGYWTSYANDRLHVYLYICAAFIVPEVLALVLFILPWVRNFIENSNWRIFNILTWWFQSR
LFVARGLREGVLDNLKYTLFWLSVLASKCAFSYWLQIRPLIAPTKQILRAKNITYQWHEFFSNGYRAAIVALWAPVLLIYFM
DVQIWYSIWSSGVGAFVGLLQHLGEIRNVHQLRLRFKIFPSAFDFSLMPMKDAGHLTLWENAKDLVKRIRLRYGWSAIH
EKVEWGQLEGGRFAHVWNIIVKTFREEDLISDREVELLEVPEVGCSWRLSVFQWPSALLANQILHTYNDVKYFKGNDK
MLWGIISKNEYRRCAVIECYESMKHILLGRLLRNDSEEYQIIKSVFEEIDASILNQRFTSTFLLRELLNIHERVVHLIEVLLGRP
TTSQIQKVVDALQSLYECLVEDFPRDTGIFKYNVRVHLNGRNKETELFMEAVTLPSKDDEPFFKNLLRLHTTLSTREPLLNV
PKGLEARRRISFFSNSLFMTMPRAPEVDHMLAFSVLTPYYSEEVIFSTKALKEENEDGITILFYLQKIFPEDWANFLERMKK
QDLLEHHLWEKDDAIEIRLWASYRGQTLARTVRGMMYYARALEVQAFLDTASEIDVLGIKELLERGSSTNSGGSFGSEPE
MIQLNRETQLNREHKVQQNLAAASMKFTYVVTCQIYGQQKKNKDYQATDILRLMKTYPGLRIAYVDERMEKGVKLYYS
VLAKYDPDLRDEVEIYRIQLPGPLKLGEGKPENQNHALIFTRGDAVQTIDMNQEMYFEEAFKMRNLLQEFTRFYGIRKPT
ILGVREHVFTGSVSSLAWFMSAQETVFVTLNQRVYANPLKIRMHYGHPDVFDRLWFLGRGGISKASRSINISEDIFAGFN
CTLRGGTVTHHEYIQAGKGRDVGLNQIAMFEAKVASGNGEQMLSRDVYRLGHHLDFFRMFSFYYTTVGFFVSNMLIVL
TVFVFLWGRVYLALSGIERSLTTGSSALANAALTATLNQQLVVQLGLLTALPMLVEDALENGFTSALWNMITMQLQLAFLF
FTFEMGTRSHYFGRTLLHGGAKYRATGRTFVVKHEKFAEIYRLYSRSHFTKGIELLMLLLCYLAYGVISSSATYIVVMISSWFL
AFTWIMAPFIFNPSGFDWLKTVEDFDDFIQWLWFKGDVFVKVEQSWEVWWEEEQWHFRTTGLWGKLLEILLDLRFFL

FQYGIVYHLHISVNNTSIFVYLLSWTYMLAAILLHLVISNASEHYAANRHGLYRLIQLLTIAVLVAIAIVLGTKTNFTFLDIFASF
LAFLPTGWGIIQICLVLRRPFLDNSKVWGTITAVARLYDLGMGIIIMTPVAVLSWLPGFQAMQTRILYNEAFSRGLQISRLL
TGKKNLH----------------------------------------------------------------------------------------------------------------------------
----------------------------------------------------------------------------------------------------------------------------------------
--------

>VMXJ 2015443 Leucobryum albidum 2005

MAPKVPHLVRKKVVARWERLVAKAIEAEEERTLKESEGVVSGDTSFVPQILHQQKKNIDTILQTARGVEQTYPQVARILYE
YSFALSQELDPRSESRGVLQFKTGLLSVIKQKRGEKTDRSDDVYIIEDFYKVLKSNLDKLDDEDWLRQQPQYSKQTPEEWA
ELKRKIYVTCRILNEVVDFLIKENPEAKRHVEIDSNLKEDLEKTAQKLDNYKPYNILPFEAPGVVNRFQNSPEVVAAINAIE
WDLRNDHEFDVNFKRPKERKLDIFDFLQYAFGFQADNVLNQREHLVLLLANSQSRLGPLRDTGPNLSPKLDETAIFNVHK
KLLGNYERWCDFLRKDPKINRRLEGRLAQMPQPRLFFSALFLLIWGEASNVRFLPECICYIFHHMADELADDTRELIDNPF
RHKSIILRDYDSYLDSVIKPVHEVVAAEFKVWNHGKSPHSRWRNYDDFNEYFWAPFCFELGWPWRLDAGFFVKPKQDT
KKSTKSKSRTTQDQTPLLQEQQQAVASGRRGNRKAGKSNFVEHRSFLHLYHSFHRLWIFLVCMLQGLAVLAFCDGKFNT
TSIKYILSVGPTFVVMKFLQSGLDVLLIIGAYRSTRARTLSRIWLRLLWFGSLSAIIVLLFVKTIQEQDSGSGSNSWFNFYVTL
LIIYAGSHLFVALLLNMPLLRRQAEKCSHFGPIPFLNWVHQERYYVGRGLYERMSDYLKYILFWLIILACKFSFSYFLQIKTM
VVPTRIIVDLTDINYRWRDIISKSNHNALTLVSLWAPVVMIYFLDLQVWYTVISALVGGLDGARIGLGEIRSLYLLRTRFTYLP
EAFSKHMQSNQLPDQTLRESVNLRKEKIFAKHFAPIWNEVITSLREEDLINNKEKEWLLMPSNTLTSLTTPSEYLSLVQWP
LFLLANKVFVACDMVEVHKQATQGELLERIGPDSYMMFAVQEAFYSLQIILEHLLVNDQGVRWVTNVYKLMEEAKNIG
QLRFQFHLRKTQLERVLDKAAKLTNVLIDEAKKIDDKLKENAEENGMKKEYTDAVRKHLLDFYDVVMQDFIADSETRIDA
IGDRDLQAAIGNGQLFSELSLPTDESRSLVRRLNYILTIKESALNVPVNLEARRRLEFFSNSLFMKMPNAPPVRKMFSFSVF
TPYYSEDVIYSPQQLAKENDDGISMMYYLRTIVPDEWENFLERVYPRKEDREARKLLLKTVFPKEYKLKSHEEQRKPDDL
NEDVKLKLRLWASYRGQTLARTVRGMMYYKRALVLQAQQEGAALSEDLEQGSQYSTSSASLDSCILDARAQAEMKFLY
VVSAQIYGEQNQGLKGAEGRQKAADISYLMKTNDSLRISYIHKGKVKVGDKEVTEYYSKLMKADPSGNDQEIYSIKLPGE
VILGEGKPENQNHAIVFTRGEALQTIDMNQEHYLEETFKMRNLLEEFDSKQDGLPDRRPTILGVREHVFTGSVSSLAWF
MSLQERSFVTLGQRVLAKPLKVRMHYGHPDVFDRIFHITRGGISKASKQINLSEDIFAGFNSTLRQGLGTHHEYIQCGKGR
DVGLNQIAAFEGKVASGNGEQTLSRDIYRLGQLFDFFRMMSFFFTTVGYYFTTMLTVLTVYVFLYGKVYLALSGVDASLN
AKGISSNTALQSALNTQFLLQIGVFTAVPMIMNFVLEEGLWRAIISFFTMQFQLSSVFFTFSLGTRTHYFGRTILHGGAKYA
STGRGFVVAHIPFAENYRMYSRSHFVKALEIMILLIVYLAYGAPERTSLTYILLTYSSWFLAISWLWGPYMFNPSGFEWQKT
VADFDDWSNWLFHKGGIGEEGKKSWEVWWQEEQSHIQTPRGRFWEIVLSLRFFIIQYGVVYALNVTGHDKNFRVYGF
SWCVLAGIIIAFKIFSVNQKSFSNFQLVLRLFQLTVFLGLIAGVIVAVVLTPLTIGDVFASALSLIPTGWGLLSIAIALRPVMKW
LRLWKSIRAIARLYEAFMGAIVFIPIALLSWFPFISTFQTRLVFNQAFSRGLEISTLLSGGNPNKDV----------------------------
----------

>VMXJ 2132845 Leucobryum albidum 1788

MGELVYNIVPVDDLAAAKDHPAVLFPEVRGAVFALRSVGDLRRPPNAQWRNGMDILDWLGFWFGFQDSNVKNQRE
HLVLLLANTQMRAIPEATDKLDGRVVRKIRRKVTKNYESWCKFVGKKSALSFLPSGKTVADDSNERQELLFTSLYLLIWGE
AANLRFMPECLCFIFHNMAQELNKLRERDTARDFKPTTCVSETNGFLKLVVSPLFEVVKAEAQVNSAGNAAHSSWRNY
DDINEYFWTDRCFTHLKWPMDQSSNFLVKPQQVKTGSKHKVGKTGFVEQRTFFNIFRSFDRLWIGYILVLQACIVTLWS
GEQRAPWVELQNKDSQARLLTIFITWSALRLFLALLDLVMQHSLVSWETWLTGLRMILKILVASIWVAIFSIFYRSMWDK
RHQDHAWSPAANKLFNRYLYTMGVFVIPEGLALALFIIPFLRNGLEKSNFKLFHVMTWWFQTRAYVARGLREGLVDNFK

YTLFWILVLASKFLFSYFLQLKPLIRPTKEILALTDIQYKWHQIFKKGNRAAVLALWAPVILIYFMDTQIWYTIWSALVGALV
GLWEHLGEIRDVRQLKLRFQVFPRAVQFNLIPESWNDNTRQRNSWWVNVKNFFQRFKLRYGTPSSHEAKDEALEFRRF
SHIWNEILKTFREEDLISNRELELLEIPTPLWNISVFQWPSTLLANEVYTALDLVKDREEMEDKDLWKKICKIEYRRCAIVES
FESIKHILLHKILRLNSTDHTLVKTLFEDHIDLAISNGKFTGAFNLKKLPEVHKCLLTLVKKILAKKVDEVVEALQALWHCVITE
FASASEIVILKQNVVEKRINTSTIFKDSVVLPEDKKFYKQIKRLETSLETKDTLLSVPKGLEARRRISFFANSLFMTMPRAPQV
EKMCAFSVLTPYYEEEVIYSLKDLNTPNEDGITTLYYLQRVFPDDWKHFKERFKREDGSKETDQQFIDRMSGLDDVAGG
GTQKKNDAKDDTKLGDEDGLELCLWASYRGQTLARTVRGMMYYERALECQAFLDAARERDIVEAQGYREMLERASSS
VSEGSSRRQEEVIPDETRRQTRPGNGETRKEQVLAVAAMKFTYVVAAQVYGKQKKNKANQAKGIAYLLETYKGLRIAYV
DEEEKHAGKQFFSVLVKYDRDIKKEVEIFRVQLPGPLKQGEGKPENQNHALIFTRGDAVQTIDMNQEMYFEEALKMRNL
LEEFDKRYGVRKPTILGVREHVFTGSVSSLAWFMSAQETSFVTLGQRVLANPLKIRMHYGHPDVFNRLWFMSRGGISKA
SRTINISEDIFAGFNCTQRGGTVTHHEYIQAGKGRDVGLNQIAMFEAKVASGNGEQVLSRDVYRLGHRLDFFRMLSFYY
TTVGFFINNLVVVLTVFAFLWGRVYLAVSGVESSLTHSKALSNTALLASLNQQLIVQLGLLTAVPMIVENALEHGFTKALWE
FFTMQLQLASVFFTFSMGTRAHYFGRTLLHGGAAYRATGRGFVVKHERFAVIYRLYRTSHFVKAIELIALLIIYRVYGASRSS
TTYIFISLTSWFLAISWLVGPFIFNPSGFDWLKTLEDFEDFTTWLKYKGGFIVGSEQSWERWWMDEQKHLEFTGWWGK
LADILLNLRFFFFQYGIVYQLNIAATSQSIFVYLVSWSYMFVAGLIHVIIATAGNRYATKQHGLYRAIQAAIITMVILVIVLLKV
FTDFSLKDLLTSLLAFVPTGWGILQIIAVLRTRWLEKSILWPVVVNVARLYEFGIGLIVLAPVAILSWLPGFQAMQTRVLFNE
GFSRGLQISQILVQKAKKSE--------------------------------------------------------------------------------------------------
-----------------------------------------------------------------------------------------------------------------------------------------
------------

>WOGB 2015637 Andreaea rupestris 1628

WCKFLGRKHNLWLPQIHQEIRQRKILYMGLYLLVWGEAANLRFMPECLCYIYHHMASELHGMLAGNVSMVTGDNMK
PAYGGGNESFLTLVIKPIYDVISQETLKNRNGTAPHSAWRNYDDLNEYFWKVDCFRLGWPMRPDAQGPKLAVMRRKVF
KPTGKVFFVEIRTFWHLFRSFDRMWAFYILGLQASIVLAWNVGPRLQDAFNGTVIKQVLSIFITASILRLIQAVLDIVMSYH
AYHSLKFLGAFRLVLKLLTSAAWVIVLTVCYVHTWNNPQGLIKDIQNWLGKGWQSSYLYVAAVVVYLVPNLIGGLFFMFP
MMRRWIESSNWRIVRFLLWWSQPRLYIGRGMHESQFALLGYTFFWVLLLASKFAFSYYIQIEPLVDPTRRIMQQSSVTY
TWHEFFPHAKNNPGALISLWAPVIMVYFMDSQIWYAVYSTIFGGISGAFRRLGEIRTLGMLRSRFSSLPGAFNENLVPAD
QKKSRRKFSFRKDFEKVSPQKNRLKAARFSQLWNEVITSFREEDLISDKERDLMLVPYSSDPHLNLVQWPPFLLASKVPIA
LQMARQAAETGRAADLLRKIKNDEYMKSAVQECYESFKRVLKDVIIGEVETRVIEGLLTEVDVNIQKETLLENFKLRELPILS
AKFIELLELLEKNHVGPEAVEAARDLAVLKLQDMYEVVTRDMMTDAMRDVWESSHGALARGQGRISELFSAKGDYPAV
LFPPPRKDASWLEQIKRLHLLLTERESAMDVPENLEARRRIAFFTNSLFMDMPRAPRVHNMLSFSVLTPYYKEDVVYSKE
NLMTDNEDGISVLFYLQKIYPDEWNNFLQRLGLESSSDPEDDIFRNEELEDKLREWASFRGQTLSRTVRGMMYYRRALE
LQAFLDMATEDELVEGYKVILESTAEQKKSQRSTWSRLQAIADMKFTYVAACQNYGEQKRQSHHNATEILKLMLNNPSL
RVAYIDEVEERQKEKTEKVYYSVLVKAVDGLDQEIYRIKLPGPVKLGEGKPENQNHAIIFTRGEGLQAIDMNQDNYLEEAF
KMRNLLEEFHEPHGVRPPTILGVREHIFTGSVSSLAWFMSNQETSFVTIGQRVLASPLKVRFHYGHPDVFDRLFHITRGG
MSKASRVINLSEDIFAGFNSILRRGNVTHHEYIQVGKGRDVGLNQISLFEAKIANGNGEQVLSRDIYRLGHRFDFFRMLSC
YFTTVGYYFSTMIVVLTVYVFLYGRIYLALSGVDNSLVHSANNKALTAALASQSLVQLGLLMALPMVMEIGLERGFRTAMS
DFLTMQLQLASVFFTFSLGTKTHYFGRTVLHGGAKYRATGRGFVVRHERFAENYRLYSRSHFTKAIELLLLLTVYTIYVSRSA
SGAVTYTLITVSMWFLVATWLFAPFLFNPSGFEWQKIVEDWDDWNKWINNRGGIGVEGNKSWESWWDEEQEHLKY
TGLLGRFIEAILSIRFFLYQYGIVYHLNIVEASHNRSITVYGLSWLVIVAVLTVLKIVSMGRDKFSADFQLMFRMLKAMLFIGS
ISVLAVLHVKQFTVGDLFASILAFIPTGWALLQISQACKPIVVKIGFWESVKSLARGYEFMMGLLLFIPVAVLSWFPFVSEF
QTRLLFNQAFSRGLQISRILAGRKKL--------------------------------------------------------------------------------------------
-----------------------------------------------------------------------------------------------------------------------------------------

----------------------------------------------------------------------------------------------------------------------------------------------------
---------------------------------------------

>WSPM 2000747 Rhytidiadelphus loreus 1886

PTSSGRGVRQFKTALLQRLERDSEPTLALRHRRSDAREIQSYYQNYYNDYVKALDGAEHSDRAQLARAYQTAAVLFEVLK
AVNRDKAEEPPPEIIAAAADVEQKKEIYVPYNILPLDAAGASQAIMQLDEVRAAVEALRNVRGLPWLTEKEPHSRAGDL
DCLDWLQDMFGFQKDNVANQREHLILMLANVHIRLLPRPEPMHKLDDRALNAVMNKLFKNYKSWCKFLGRKNNLW
LPQIHQEVRQRKILYMGLYLLIWGEAANLRFMPECLCYIYHHMASELHGMLAGNVSMVTGDNMKPAYGGEDESFLTFV
VTPIYKVISRETLKNRNGTAPHSAWRNYDDLNEYFWKEDCFRMGWPMRPDADFFVPAQTSSSTNDIHAKDYQSSSKSF
FVEIRSFWHLFRSFDRLWAFYILGLQAMIVLAWNVGPNLQNAFTGAVIKQILSIFITASILRLIQAFLDIVFCYHAFRSIKLLG
VLRLVLKLFTSAAWVVVLTISYARTWVNPQGLIGEIQKWLGKSLENSYLYVAAVVVYLIPNFIGAFFFLFPMIRRWIESSNW
TVVRVLLWWSQPRLYIGRGMHESQIALLGYTFFWVLLIASKFSFSYFIQIEPLVSPTKAIMQQSSVIYTWHEFFPNARNNP
GALISLWAPVIMVYFMDSQIWYAVFSTIFGGYKFMYCTFGLLTCSVISINQLLPRRFLCDISGSFRRLGEIRTLGMLRSRFSS
LPGAFNESLVPDDGKQARKGFSFSRDFEKISPLKDRLKAARFSQLWNEVITSFRQEDLISDKERDLMLVPYSSDPHLKLVQ
WPPFLLASKVPIALQMAKQAAETGRAADLLRKIKADEYMKCAVTECYESLKRVLKRFIVGEVEIRVIDGLFDEVDVNVEKG
TLLDNFKLGELPVLSAKFIELLELLEKNHADQEAIDSARDLAVLKLQDMYEVVTRDMMSDSMRDSWDSSLGALAGGQG
RKSELFSAKGDEPAKVVFPLSRNEAWIEQIKRLHLLLTERESAMDVPENLEARRRIAFFTNSLFMNMPHAPRVRNMLSFS
VLTPYFKEDVVYSKENLMKENEDGISVLFYLQKIYPDEWSNFLQRIGLESSEDPEAQIFGSSDLEDHLREWASFRGQTLSR
TVRGMMYYRRALELQAFLDMATEDELVDGYKVLTEATPEQKKSQRSTWSQLQAIADMKFTYVAACQMYGEQKRRGH
HSATEILKLMLNNLSLRVAYIDEVDGRPKEKSMKVYYSVLVKAINGLDQEIYRIKLPGIVRLGEGKPENQNHAVIFTRGEGL
QTIDMNQDNYLEEAFKMRNLLQEFHEPHGVRPPTILGVREHIFTGSVSSLAWFMSNQETSFVTIGQRVLASPLKVRFHY
GHPDVFDRLFHITRGGMSKASRVINLSEDIFAGFNSILRRGNVTHHEYIQVGKGRDVGLNQISLFEAKIACGNGEQALSR
DVYRLGHRFDFFRMLSCYFTTVGYYLSTMIVVLTVYVFLYGRIYLALSGVDDSLVHSANNKALTAALASQALVQLGLLMAL
PMVMEIGLERGFRTALSDFLTMQLQLASVFFTFSLGTKTHYFGRTILHGGAKYRATGRGFVVRHERFADNYRLYSRSHFTK
AIELFLLLIVYTLYVSRSAKGAVTYVLITFSMWFLVASWLFAPFLFNPSGFEWQKIVEDWDDWNKWMSNRGGIGVEGSK
SWESWWDEEQEHLNYTGVFGRFVESLLSIRFFLYQYGIVYHLNIARSSKDLSISVYGLSWLVIVAVLTVLKIVSMGRDKFSA
DFQLLFRLLKASLFIGSVSVIAILHVKNFTVGDLFACILAFIPTGWALLQILEACKPVVIRLGFWKSVKALARGYEYLMGLLLF
TPIAILSWFPFVSEFQTRLLFNQAFSRGLQISRILAGRKKL---------------------------------------------------------------------
--------------------------------------------------------------------------------------

>ZACW 2006709 Leucodon sciuroides 1932

MASGGKPESGVTLLSRRIPRTYTAGGLTEVFDSEVVPSSLASIAPILRVANEIEASSPRVAYLCRYHAFEKAHRIDSTSSGRG
VRQFKTALLQRLERDSEPTLALRHRRSDAREIQSYYQNYYNDYVKSLDGAEHSDRAQLARAYQTAAVLFDVLKAVNRDKA
EEPPPEIIAAAADVEQKKEIYVPYNVLPLDAAGSSQAIMQLDEVRAAVEALRNVRGLPWQTVKESHPRAGDLDCLDWL
QDMFGFQKDNVANQREHLTLMLANVHIRLLPRPDPMHKLDDRALNAVLNKLFKNYKSWCKFLGRKNNLWLPQIHQE
VRQRKILYMGLYLLVWGESANLRFMPECLCYVYHHMACELHGMLAGNVSMVTGDNMKPAYGGEDESFLTLVVTPIYKV
ISKESLKNRNGTAPHSAWRNYDDLNEYFWKVDCFCMGWPMRPDADFFVPDQTFLNTTVVTNEKVYQSTSKSFFVEIR
TFWHLFRSFDRLWAFYILGLQAMIVLAWNVGPNLQNALTGAVMKQILSIFITASILRLIQAFLDIVFCYHAFRSIKLLGVLRL
VLKLFTSAAWVIVLTISYARTWVNPQGLIGEIQKWLGKSLESSYLYVAAVVVYLIPNFIGAFFFLFPMIRRWIESSNWTVVR
VLLWWSQPRLYIGRGMHESQIALLGYTFFWVLLIASKFAFSYFIQIEPLVAPTKAIMQQRSVIYTWHEFFPNARNNPGALI
SLWAPVIMVYFMDSQIWYAVFSTIFGGISGSFRRLGEIRTLGMLRSRFSSLPGAFNESLVPDDGKRARKGFSFSRDFEKISP

SKDRSKAARFSQLWNEVITSFRQEDLISDKERDLMLVPYSSDPHLKLVQWPPFLLASKVPIALQMAKQAAETGRAADLLR
KIKIDEYMKCAVTECYESLKRVLKRFIVGEVEIRVIDGLFDEVDVNVEKETLLDNFKLGELPVLSAKFIELLELLEKNHAGQEA
IDSARDLAVLKLQDMYEVVTRDMMSDSMRDSWDSSLGALAGGQGRKSELFSAKGDEPAKVVFPLSRSEAWIEQIKRLH
LLLTERESAMDVPENLEARRRIAFFTNSLFMNMPHAPRVRNMLSFSVLTPYFKEDVVYSKENLMKENEDGISVLFYLQKI
YPDEWSNFLQRIGLESSEDPEAQIFGCNDFEDKLREWASFRGQTLSRTVRGMMYYRRALELQAFLDMATEDELVDGYK
VLTDATPEQKKSQRSTWSQLQAIADMKFTYVAACQMYGEQKRQGHHSATEILKLMLNNLSLRVAYIDEVEERQNESKVY
YSVLVKAINGLDQEIYRIKLPGVVRLGEGKPENQNHAVIFTRGEGLQAIDMNQDNYLEEALKMRNLLQEFHEPHGVRSP
TILGVREHVFTGSVSSLAWFMSNQETSFVTIGQRVLASPLKVRFHYGHPDVFDRLFHITRGGMSKASRVINLSEDIFAGFN
SILRRGNVTHHEYIQVGKGRDVGLNQISLFEAKIACGNGEQALSRDIYRLGHRFDFFRMLSCYFTTVGYYFSTMIVVLTVY
VFLYGRIYLALSGVDDSLVHSANNKPLTAALASQALVQLGLLMALPMVMEIGLERGFRTALSDFLTMQLQLASVFFTFSLG
TKTHYFGRTILHGGAKYRATGRGFVVRHERFADNYRLYSRSHFTKAIELFLLLIVYTLYVSESSRGAVTYILITFSMWFLVAS
WLFAPFLFNPSGFEWQKIVEDWDDWNKWMSNRGGIGVEGSKSWESWWDEEHEHLNYTGFIGRVVESILSLRFFLYQ
YGIVYHLNIARSSNDLSISVYGLSWLFILAVLTILKIVSMGRDKFSADFQLMFRLLKASLFIGSVSVIAILHVKNLTVGDLFASIL
AFIPTGWALLQIAQASKPVVIRLGFWSSVKSLARGYEYSMGLLLFTPIAVLSWFPFVSEFQTRLLFNQAFSRGLQISRILAG
RKKL-------------------------------------------------------------------------------------------------------------

>ZACW 2006711 Leucodon sciuroides 1931

MASGGKPESGVTLLSRRIPRTYTAGGLTEVFDSEVVPSSLASIAPILRVANEIEASSPRVAYLCRYHAFEKAHRIDSTSSGRG
VRQFKTALLQRLERDSEPTLALRHRRSDAREIQSYYQNYYNDYVKSLDGAEHSDRAQLARAYQTAAVLFDVLKAVNRDKA
EEPPPEIIAAAADVEQKKEIYVPYNVLPLDAAGSSQAIMQLDEVRAAVEALRNVRGLPWLTEKEPISRAGDLDCLEWLQD
MFGFQKDNVANQREHLILMLANVHIRLLPRPEPMHKLDDRALNAVMNKLFKNYKSWCKFLGRKNNLWLPQIHQEVR
QRKILYMGLYLLVWGEAANLRFMPECLCYIYHHMASELHGMLAGNVSMVTGDNMRPAYGGEDESFLTFIVTPIYEVISR
ETLKNRNGTAPHSAWRNYDDLNEYFWKGDCFRIGWPMRPDADFFFPARTSSSTNDIDGKDYQLSSKSFFVELRSFWHL
FRSFDRLWAFYILGLQAMIVLAWNVGPNLQNALTGAVMKQILSIFITASILRLIQAFLDIVFCYHAFRSIKLLGVLRLVLKLFT
SAAWVIVLTISYARTWVNPQGLIGEIQKWLGKSLESSYLYVAAVVVYLIPNFIGAFFFLFPMIRRWIESSNWTVVRVLLWW
SQPRLYIGRGMHESQIALLGYTFFWVLLIASKFAFSYFIQIEPLVAPTKAIMQQRSVIYTWHEFFPNARNNPGALISLWAPV
IMVYFMDSQIWYAVFSTIFGGISGSFRRLGEIRTLGMLRSRFSSLPGAFNESLVPDDGKRARKGFSFSRDFEKISPSKDRSK
AARFSQLWNEVITSFRQEDLISDKERDLMLVPYSSDPHLKLVQWPPFLLASKVPIALQMAKQAAETGRAADLLRKIKIDEY
MKCAVTECYESLKRVLKRFIVGEVEIRVIDGLFDEVDVNVEKETLLDNFKLGELPVLSAKFIELLELLEKNHAGQEAIDSARD
LAVLKLQDMYEVVTRDMMSDSMRDSWDSSLGALAGGQGRKSELFSAKGDEPAKVVFPLSRSEAWIEQIKRLHLLLTER
ESAMDVPENLEARRRIAFFTNSLFMNMPHAPRVRNMLSFSVLTPYFKEDVVYSKENLMKENEDGISVLFYLQKIYPDEW
SNFLQRIGLESSEDPEAQIFGCNDFEDKLREWASFRGQTLSRTVRGMMYYRRALELQAFLDMATEDELVDGYKVLTDAT
PEQKKSQRSTWSQLQAIADMKFTYVAACQMYGEQKRQGHHSATEILKLMLNNLSLRVAYIDEVEERQNESKVYYSVLVK
AINGLDQEIYRIKLPGVVRLGEGKPENQNHAVIFTRGEGLQTIDMNQDNYLEEAFKMRNLLQEFHEPHGVRPPTILGVR
EHIFTGSVSSLAWFMSNQETSFVTIGQRVLAIPLKVRFHYGHPDVFDRLFHITRGGMSKASRVINLSEDIFAGFNSILRRG
NVTHHEYIQVGKGRDVGLNQISLFEAKIACGNGEQALSRDIYRLGHRFDFFRMLSCYFTTVGYYLSTMIVVLTVYVFLYGRI
YLALSGVDDSLVHSANNKALTAALASQALVQLGLLMALPMVMEIGLERGFRTALSDFLTMQLQLASVFFTFSLGTKTHYF
GRTILHGGAKYRATGRGFVVRHERFADNYRLYSRSHFTKAIELFLLLIVYTLYVSRSAKGAATYILITFSMWFLVASWLFAPFL
FNPSGFEWQKIVEDWDDWNKWMSNRGGIGVEGSKSWESWWDEEQEHLNFTGIFGRFVESLLSIRFFLYQYGIVYHLN
IARSSKDLSISVYGLSWLVIVAVLTVLKIVSMGRDKFSADFQLLFRLLKALLFIGSVSVIAILHVKSFTVGDLFACILAFIPTGWA
LLQILQACKPVVVRLGFWKSVKALARGYEYLMGLLLFTPIAILSWFPFVSEFQTRLLFNQAFSRGLQISRILAGRKTL--------
------------------------------------------------------------------------------------------------------

>ZACW 2006713 Leucodon sciuroides 1931

MSSGGRLESGAADLPRRLSRMYTAGGLTEVFDSEVVPSSLASIAPILRVANEIEGSSARVAYLCRYHAFEKAHRIDPTSSGR
GVRQFKTALLQRLERDSEPTLALRHRRSDSREIQSYYQNYYNDYVKALDGAEHSDRAQLARAYQTAYVLFEVLKAVNRDK
AEEPPPEIIAAAADVEQKKEIYVPYNILPLDAAGASQAIMQLDEVRAAVEALRNVRGLPWLTEKEPISRAGDLDCLEWLQ
DMFGFQKDNVANQREHLILMLANVHIRLLPRPEPMHKLDDRALNAVMNKLFKNYKSWCKFLGRKNNLWLPQIHQEV
RQRKILYMGLYLLVWGEAANLRFMPECLCYIYHHMASELHGMLAGNVSMVTGDNMRPAYGGEDESFLTFIVTPIYEVIS
RETLKNRNGTAPHSAWRNYDDLNEYFWKGDCFRIGWPMRPDADFFFPARTSSSTNDIDGKDYQLSSKSFFVELRSFWH
LFRSFDRLWAFYILGLQAMIVLAWNVGPNLQNALTGAVMKQILSIFITASILRLIQAFLDIVFCYHAFRSIKLLGVLRLVLKLF
TSAAWVIVLTISYARTWVNPQGLIGEIQKWLGKSLESSYLYVAAVVVYLIPNFIGAFFFLFPMIRRWIESSNWTVVRVLLW
WSQPRLYIGRGMHESQIALLGYTFFWVLLIASKFAFSYFIQIEPLVAPTKAIMQQRSVIYTWHEFFPNARNNPGALISLWA
PVIMVYFMDSQIWYAVFSTIFGGISGSFRRLGEIRTLGMLRSRFSSLPGAFNESLVPDDGKRARKGFSFSRDFEKISPSKDR
SKAARFSQLWNEVITSFRQEDLISDKERDLMLVPYSSDPHLKLVQWPPFLLASKVPIALQMAKQAAETGRAADLLRKIKID
EYMKCAVTECYESLKRVLKRFIVGEVEIRVIDGLFDEVDVNVEKETLLDNFKLGELPVLSAKFIELLELLEKNHAGQEAIDSA
RDLAVLKLQDMYEVVTRDMMSDSMRDSWDSSLGALAGGQGRKSELFSAKGDEPAKVVFPLSRSEAWIEQIKRLHLLLT
ERESAMDVPENLEARRRIAFFTNSLFMNMPHAPRVRNMLSFSVLTPYFKEDVVYSKENLMKENEDGISVLFYLQKIYPDE
WSNFLQRIGLESSEDPEAQIFGCNDFEDKLREWASFRGQTLSRTVRGMMYYRRALELQAFLDMATEDELVDGYKVLTD
ATPEQKKSQRSTWSQLQAIADMKFTYVAACQMYGEQKRQGHHSATEILKLMLNNLSLRVAYIDEVEERQNESKVYYSVL
VKAINGLDQEIYRIKLPGVVRLGEGKPENQNHAVIFTRGEGLQAIDMNQDNYLEEALKMRNLLQEFHEPHGVRSPTILG
VREHVFTGSVSSLAWFMSNQETSFVTIGQRVLASPLKVRFHYGHPDVFDRLFHITRGGMSKASRVINLSEDIFAGFNSILR
RGNVTHHEYIQVGKGRDVGLNQISLFEAKIACGNGEQALSRDIYRLGHRFDFFRMLSCYFTTVGYYFSTMIVVLTVYVFLY
GRIYLALSGVDDSLVHSANNKPLTAALASQALVQLGLLMALPMVMEIGLERGFRTALSDFLTMQLQLASVFFTFSLGTKT
HYFGRTILHGGAKYRATGRGFVVRHERFADNYRLYSRSHFTKAIELFLLLIVYTLYVSESSRGAVTYILITFSMWFLVASWLFA
PFLFNPSGFEWQKIVEDWDDWNKWMSNRGGIGVEGSKSWESWWDEEHEHLNYTGFIGRVVESILSLRFFLYQYGIVY
HLNIARSSNDLSISVYGLSWLFILAVLTILKIVSMGRDKFSADFQLMFRLLKASLFIGSVSVIAILHVKNLTVGDLFASILAFIPT
GWALLQIAQASKPVVIRLGFWSSVKSLARGYEYSMGLLLFTPIAVLSWFPFVSEFQTRLLFNQAFSRGLQISRILAGRKKL----------------------------------------------------------------------------------------------------------------

>ZACW 2012855 Leucodon sciuroides 1580

KEFDAFLDAVIKPVHEIVAAEAKFCKDRKLPHSKWRNYDDINEYFWTPVCFELSWPWRLSSGFFVKPKQDSNKKRNKKF
RNTRDEIPLLQEQNQRSEPGHRREKKAGKSHFVEHRSGLHLYHSFHRLWIFLVCMLQGLAVFAFCDGKFNATSIKYILSVG
LTFVIMKFIQSVLDVILIIGAYRSSRARTLSRIWLRLFWFASLSAIIVFLFLKTIQEEESRSGQSTWFRIYCVLLILYGGSHLFVAL
LMNMPWLRKQAEKCSNFGPVPFLNWVHQERYYVGRGLYENTGDYFSYILFWIVVLACKFSFSYFLQIKTMVGPTRTIVD
LTDLNYRWRDVVSKSNHNALTLVSLWAPVVMIYFLDLQVWYTVISALVGGLDGARMGLGEIRSLDMLRNRFTFLPAAFT
KRLQAHQHKVLRESVYTNEKVEAIQFAPIWNDVIASLREEDLINNKEMEWLVMPGNTLTNLTANSQDLTLVQWPLFLLA
NKVFVACDLAEVHKQASQLDLWKIIGRDPYMMFALQEAFVTLRIILEHLLEEDYLGKRWVNFVYGNMREAMERDQLKH
KYNLKDQLKKMLDKAAKLTTVLINEAKKLKDDYTKEMREAYTAAVRKELLDFYDVVMRDFIIDSETRDDAILQAGKQSEG
LFTELTLPTDESNSLVRRLNQILTIKESALNVPVNLEARRRLEFFSNSLFMKIPNSPTVRKMFSFSVFTPYYEEDVIYSPSKLAE
ENNDGISMMYYLRTIVPDEWANFLERIYPRKEDREARKALLKTIFPKEYEFKGNEKPRKPDDLNEAVKLQLRLWASYRGQ
TLARTVRGMMYYKRALILQAKLEGESLSDDVEQGSQYSTTSTSSDLDILDAGAQAELKFLYVVSAQIYGEQQQGVKGDD
GRQKAADISYLMRTNDSLRISYIHKGKVKVGAEEVTKYYSKLMKADPSGNDQEIYSIELPGEVILGEGKPENQNHAIVFTR
GEALQTIDMNQEHYLEETFKMRNLLEEFDDTKNYGLRKPTILGVREHVFTGSVSSLAWFMSLQERSFVTLGQRVLAKPLK

VRMHYGHPDVFDRIFHITRGGISKASKQINLSEDIFAGFNSNLRQGNVTHHEYIQCGKGRDVGLNQIAAFEGKVASGNG
EQTLSRDIYRLGQLFDFFRMLSFFFTTVGYYFTTMLTVLTVYVFLYGKVYLALSGVDADLKNKGLSTNVALQSALDTQFLLQ
IGVFTAVPMIMNFLLEEGILKAIISFCTMQLQLSSVFFTFSLGTRTHYFGRTILHGGAKYASTGRGFVVAHIPFAENYRMYS
RSHFVKALEIMLLLIVYLAYGAPERTTFTYILLTFSSWFLAISWLWAPFIFNPSGFEWQKTVSDFEDWTNWLFHKGGIGEK
GVKSWEVWWEEEQEHICTIRGRFWEIVLSLRFFLVQYGVVYALNIVGHDRNFRVYGFSWCVLAGVVLTFKVFSVNQKTF
SNFQLILRLFQMIVFLALIGGVVVAVAITSLTIGDVFASALSLIPTGWGLLSIAIASKPAVKKIGLWKSVRAIARLYEAFMGAIV
FIPIALLSWFPFVSTFQTRLVFNQAFSRGLEISTLLSGNNPNKDM-------------------------------------------------------------
----------------------------------------------------------------------------------------------------------------------------------------------
----------------------------------------------------------------------------------------------------------------------------------------------
------------------------------------------------------------------------------------------------------------------------------

>ZACW 2012977 Leucodon sciuroides 1779

MGDLVYNILPVEDNAAARDHPAVKFPEVQAAVRALRSIGDLRRPPQWRTGISVDVLDWLGCWFGFQDSNVKNQREH
LVLLLANAQMRGIPDSADKLDGKVLRKIRRKVTKNYESWCTYVGKDNMLRLPFGKQVGAERQELLYTSLFLLIWGEAAN
LRFMPECLCFIFHHMAQELNKMLERDAFENFKPTTCAHNGFLRLVVSPLYEVVGAEAAVNGTGNAAHSSWRNYDDINE
YFWSERCFTHLKWPMDGSSTFLIKPQDVGNGAKHKVGKTGFVEQRTFFNIFRSFDRLWIGYILILQACIVTLWSGQQRA
PWVELRNKDSLARLLTIFITWSALRLILALLDLVMMHSLISWETWRTGLRMILKVLVASIWVLIFSIFYSSMWHRRHKDHA
WSSAANSLFIRYLYTMGAFCLPEGLALALFIIPFVRNFLEKSRFRVFHLLTWWFQSRTYVARGLREGIVDNFKYTLFWALVL
ASKFLFSYFLQLKPLIRPTKEILEITTIDYKWHQIFGKGNRVAVLALWAPVILIYFMDTQIWYTIWSALVGALVGLLDHLGEIR
NVHQLKLRFQMFPGAVQFNLIPESWGAGIREQYSWWVNVKNFFMRIRLRYGAPSQEAKEESMEARRFSHIWNEILKT
FREEDLISNRELELLEIPTPVWNISVFQWPSTLLANEVQTALDFLKSKDGGKGMDDKAVWKKTIKSEYGRCAVVESYESM
KHILVYRILRLNSPDQIMVKGLFEDHIDRAINQRKFSDAFTLSKLPEVHKCVLTLVKNILTHKAEEVVKALQTLWHYVVNEF
PKAEERTLIKENFARQRLSTATVFKDSVVLPGVGDKNFYKQLKRLQTTLETKDTLLSVPKGLEARRRISFFANSLFMTMPRA
PQVEKMFAFSVLTPYYEEEVIYSLKDLNKRNEDGIDTLFYLRTIFADDWKHFKERFSGVEETDKQFIHRMSGQHDAAEGG
SQMKHDSKAKKKSGKEKTIEDDGLELCLWASYRGQTLARTVRGMMYYERALECQAFLDAATEKDLNEALGFKEMIARA
SSNVSEGSSRRQEERISDDTRERQVLATAAMKFTYVVAAQLYGKQKKKEENQAKGIAYLLKTYRGLRIAYVDEVETPAGNQ
YFSVLVKYDRTANMEVEIFRVELPGKLKLGEGKPENQNHALIFTRGDAVQTIDMNQEMYFEEALKMRNLLQEFDKHHG
VRKPTILGVREHVFTGSISSLAWFMSAQETSFVTLGQRVLANPLKIRLHYGHPDVFNRLWFMSRGGISKASRTINISEDIFA
GFNCTQRGGTVTHHEYIQAGKGRDVGLNQIALFEAKVASGNGEQVLSRDVYRLGHRLDFFRMLSFYYTTVGFFINNLM
VVLTVFAFLWGRFYLAVSGVESSLSNSKVLSNTALLASLNQQLIVQLGILTALPMIVENALEHGFTKALWEFFTMQLQLAS
VFFTFSMGTRAHYFGRTLLHGGASYRATGRGFVVKHERFAVIYRLYSRSHFVKAIELIALLVVYLIYGGHRSSSTYLLISLTSW
FMALTWLLGPFIFNPSGFDWLKTLEDFEDFIGWLKYKGGVIVDSEQSWERWWMDEQKHLKTTGLWGKLMDIILNLRF
FFFQYGIVYQLKIAATSQSIFVYIVSWSYVFVAGVIHMIIASAGQRYSTKKHGLYRAIQAILITLVIGAIVVLKIFTHFSLKDLLTS
LLAFVPTGWGILQILTVLRTDWLEKTDIWPIVVNVGRLYEFGMGLIVLAPVAILSWLPGFQAMQTRVLFNEAFSRGLHIA
QLLETVQKNPKSE---------------------------------------------------------------------------------------------------
----------------------------------------------------------------------------------------------------------------------------------------------
-----------

>ZACW 2017067 Leucodon sciuroides 1982

MERPPRPPHRMSKRVLVKWEKLVEKALVTSSYNRDGRGRGSNSRQRGERAPQSLAQQADIDAVLQAADEIGHENLQV
GRILTEHAYRLTQQLDPRSEGRGVLQFKTGLKSIIKQKQQAKQGEAVDRSQDIRIIQVYYKHYRERHHIDQLEEEARNRRP

VLSTDLVPESQDQSFEKLRRVYEISTILNDVVDALLKEAEPGDASRLQDTDEKRVMEEDAKKLKGFKAYNILPLKTPLDAPS
VLNPFDFFPEVVGATQVLVYTRDLPRFPSDYEVPEDRVVDIFDFLHYAFGFQKDNVANQREHIILLLSSAQSRLGTLDQGR
DSDGNKTSLNPDKAVSNVHERILENYVRWCHFLRREPQNKRAFTQQRRLILAALYLLVWGEAANLRFMPECLCFIFHNL
ADECIDLLGRTYVERSKTVKPNEDGSIEFSFLDQIITPVYDAVAAEAKSSQGGKVPHSHWRNYDDFNEYFWQPNCFVEL
GWPWRTDAGFFKPPKMKDAMKVPLPQTEANLPEAAGRRRKHKVGKVHFVEHRSGFHIYHSFHRLWIFFICMLQGLTV
WAFCSKNGNLNLHVRTIKRIMSVGPTFVIMKFIQSVFDIVFMWGAFKATRLQTVARMLLRFLWFTCLSAAVLFLYIKTLEE
DARNDGSGSWFRIYYILISSYAGANAVFVFLLRIPFLQRQGAKCSNVYLFQFVKWLYQERYYVGRSMYERTRNYIKYSIFWI
VILACKFAFTMHFQIMPLVDPTRLIIGFNDIKYKWPDFVSDSNHNALTIVSLWAPVVMIYFLDTQVWYTVISAFLGGIEGA
RDKLGEIRTLEMLRKRFPNYPAAYVKHMQPPINRLSSASPSHHSASRVTKVKPKKPDAIRFQPIWNRVIKSLREEDLINNR
EKVLLKMPPNLMFHSNGAPNDLIHWPLFLLANKVHIAVELAVEHKTRHDGLWEKVQRDEYMAYAVQETFETLEPLLVSV
LNPMGASWVNDIFEKLRYSLGSADLRDSFNLNKLRDVLEKLRDLTEHLGNEDVDERRSKATRFFFQLYDVVMRDFLSSRT
REEFEEMPGFREAVQNGLLFTELNWPNKAGQKQAKRLNNLLTVQKIKDQEGKTKTLNTETIPHNLEARRRLQFFTNSLF
MHMPEAPPIRKMFSFCVFTPYYEEDVMYDMEKLNQENEDGITILFYLQKIYPDEWQNFLERIGLIEAIVIREIENPKSGRH
EKVKLDLRLWASYRGQTLARTVRGMMYYKAALIIQGIQEGASGGDLEEGIPPSLVEAQGSINRSAEAQAELKFTYVVTCQ
IYGEQKRKGKVQAADILYLMQKHESLRIAYIDVVESSKKGRKPSYYSKLCKVDRSDPSQKDQEVYSIKLPGDIKLGEGKPEN
QNHAIIFTRGDCIQTIDMNQDNFMEEAFKMRNLLEEFSQTHHGLHKPTILGVREHVFTGSVSSLAWFMSMQESSFVTL
GQRVLARPLKVRMHYGHPDVFDRVFHITRGGISKASRVINLSEDIFAGFNTTLRLGNVTHHEYIQVGKGRDVGLNQIALF
EAKVASGNGEQTLSRDVYRLGQLLDFPRMLSFFFTSIGFYVTTMMTVLTLYVFLYGKAYLALSGVDAALKRNSKILQNPSL
EAALNTQFLFQIGIFTAVPMIVNLILEQGILKAIISFCTMQLQLASVFFTFSLGTRTHYFGRTILHGGAKYRSTGRGFVVTHIH
FAENYRSYSRSHFTKALEIIMLLIIYLAYGAQNRTSVTFILLTFSSWFLALSWLFAPYIFNPSGFEWQKTVEDFDDWTNWM
FYKGGVGVKIENSWEAWWFDEQDHIRSPRGRFWETVLSLRFFLFQYGVVYSLSVTRGSSSILVYAYSWFVLLGLVIIFKVFT
VSQKASANFQLAVRLFQGLLFVCLIAGVVVAIVLSPLTVGDVFALALALIPTGWGLLSIAIAFRPLMERMRFWKSVREIARI
YDACMGMIIFIPIAFLSWFPFVSTFQTRFVFNQAFSRGLEISLILSGNRPNR--------------------------------------------------
----------

>ZTHV 2009163 Atrichum angustatum 1934

MASSAVPEGTNTPVLRRLSRTYTTGALTELFDSEVVPSSLASIAPILRVANEIEAQRPRVAYLCRYHAFEKAHRLDPTSSGR
GVRQFKTALLQRLEKDSEPTLAARHRRSDAREIQSYYQQYYNDYVKALEQPEHSDRAQLAKAYQTAAVLFEVLKAVNRDK
AEEPPPEIIAAAADVEQKKEIYVPYNVLPLPLDAAGASQAIMQLDEVRAGVESLRNVRGLPWQANKEGQHRSGDLDCL
DWLQDMFGFQKDNVANQREHLILMLANVHIRCLPRPEPMHKLDDRALNEVMNKLFKNYKYWCKFLGRKHNLWLPQI
HQEVRQRRILYMGLYLLVWGEAANLRFMPECLCYIFHHMASELHGMLAGNVSMVTGDNMKPAYGGEDESFLSLVVTPI
YEVIAKETLKNRGGTAPHSAWRNYDDLNEYFWKVDCFRLGWPMRPEADFFTPSPSPGKTMMANAMEGNVLNTSSKT
FFVEIRTFWHIFRSFDRMWAFYILGLQASIVLAWNVGRDLTKAFNGTVIKQVLSIFITASILRLTQAVLDIVMSYQAFRSLKL
LGVLRLILKLFTSAAWVIVLTVCYVHTWNNPQGLIKAIQNLLGNTWQSSYLYVAAVVVYLIPNIIGGFFFLFPMLRRSIESSN
WKVVRFLLWWSQPRLYIGRGMHESQLALFGYTFFWVLLIVSKFAFSYYIQIEPLVAPTRAIMDQHSVTYTWHEFFPHAS
NNPGALISLWAPVIMVYFMDSQIWYAVYSTIFGGISGSFRRLGEIRTLGMLRSRFSALPGAFNENLVPQKNKKRRRGFSF
RRAFEKVTPQESRQKASRFSQLWNEVITSFRQEDLISDKERDLMLVPYSSDPHLNLVQWPPFLLASKVPIALQMARQAA
ETGRAADLMRKIKTDEYMKSAVMECYESFKRVLNVLIVGEVEARVIKGIFEQVESNVEKDTLLDNFKLKELPVLSVKFIELL
ELLESDHSGQEAEKARDLVVLKLQDMYEVVTRDMMSEGMRDTWELGNGGEAREAGISELFSGKGDEPAVQFPPPRTE
AWIEQIKRLHLLLTERESAMDVPENLEARRRIAFFTNSLFMDMPQAPRVRNMLSFSVLTPYYREDVVYSKANLMTENED
GISVLFYLQKIYPDEWSNFLERVGLGPLTDESEIWNNEDLEDKLREWASFRGQTLSRTVRGMMYYRRALELQAFLDMAS
EDELVDGYKIITEASAETKKSQRSTWSQLQAIADMKFTYVAACQNYGEQKRQSHHNAAEILKLMLNNPSLRVAYIDEVEE
RRKERTVKVYYSVLVKAVDGLDQEIYRIKLPGPVRLGEGKPENQNHAIIFTRGEGLQAIDMNQDNYLEEAFKMRNLLQEF

HEPHGVRPPTILGVREHIFTGSVSSLAWFMSNQETSFVTIGQRVLASPLKVRFHYGHPDVFDRLFHITRGGMSKASRVIN
LSEDIFAGFNSILRRGNVTHHEYIQVGKGRDVGLNQISLFEAKIANGNGEQSLSRDIYRLGHRFDFFRMLSCYFTTVGYYFS
TMIVVLTVYVFLYGRMYLALSGVDNSLVHLANNKALTAALASQSLVQLGLLMALPMVMEIGLERGFRTAMSDFLTMQLQ
LASVFFTFSLGTKTHYFGRTILHGGAKYRPTGRGFVVRHERFAENYRLYSRSHFTKGIELLLLLIVYTIYVSRSASGAVTYILITF
SMWFLVASWLFAPFLFNPSGFEWQKIVEDWDDWNKWISNRGGIGVEGSKSWESWWDEEQEHLSFTGFRGRLIECIL
AMRFFLYQYGIIYHLNIVQTSNNLSISVYGLSWLVILAVIAVLKIVSMGRDKFSADFQLMFRLLKACLFVGSISVIAVLHVRQ
FTVGDLFASILAFIPTGWALLQIAQACKPVVLKIGFWDSVKSFARGYEFMMGLVLFTPVAVFSWFPFVSEFQTRLLFNQAF
SRGLQISRILAGRKKL-----------------------------------------------------------------------------------------------------------

>BNCU 2011463 Radula lindenbergia 1764

GDVYNIIPVQDTLSEHAALRFPEVRAAISALQAIGGLQRADGWMQGMDMLDWLGLTFGFQEDNVRNQREHLVLLLA
NAQMRQQPAPDPIDKLVFDVVRNLRKKITKNYLSWCHFLHVKHNLEKLEGRHHEMDERKELMYSSLYLLVWGEAANLR
FMPECLCFIFHNMAGELNRILDLSIDEGGQRIHAASEGRQGFLLKVITPIYNVAKAESDASGKGAAPHSAWRNYDDMNE
YFWSKRCLTQLRWPLQMSCNYLVTPAKGKGKVTRQKVGKTGFVEQRSFWNIFRSFDRLWVGLILMLQALIIVAFRGKGR
RTGESDKLPWKVLSERDSQAEVLTIFITWAGLRVVQALLDFGMQYTLISLGTLKVGLRMTLKLLVACVWVVLFSVYYSRM
WHQRNRDDRWTDAALQRLYVYLGFALAFIAPEVLALILFIIPWVRNAVETSNWRIFAPFTWWFQSHIFVGRGLREGFFD
NLKYTLFWLVVLVAKVAFSYFLQVLPLVKPTRTTLNIRDGSIDYKWHQFFKDANRTALVCMWSPVILIYFMDLQIWYSLFS
ALVGALVGLMSHLGEIRHAAQMKARFLVLPSAVQFNLTPEEGVLKHRPFSNWWGGLKDVVRRIKLRYGVYRLKEQEGA
KRMEIQRFRQMWNDIIAIFREEDLISDGELELLVMPTAGWNISVIQWPAILLSNEVRVALRYCDPEHHRWYSSDRWLWR
KISSNEYRRCAVLETYESLKHVLDRIVLEASDDSHIFPVIFKEIEDSIHQGRFMKTFRLKALPDIHTRVSKLIGLLKKRPQSSDL
PKVVEAMQGLYEVLVRDFPKDQNTKSKFNELVSRSLKFVDAVELPDPEGDDQFFHQLDRLYTTLCTGEAYLNVPRSLEAK
RRLAFFSNSVFMTMPRAPPVEKMLSFSVLTPYYAEDVMYNKKQLDEQNEDGVRTLFYLQKIFPDDWKYFQERMKGLGI
FTEDEMWEVDDGLELRLWASYRGQTLARTVRGMMYYDRALQKLAYLDTASEYEITGFKDLVSRTTSGMELSVRGSRHS
SLGGYEKSFREPEIFLGHKDELNKATASMKFTYVVSCQIYGTQKAQKAQQASDILYLMKQYEPLRIAYVDERGGDREQRQ
RYSVLIKYDRELNREVEIYRVQLPGPLLLGEGKPENQNHALIFTRGEAIQTIDMNQENYFEEALKMRNLLQEFEKKSGIRKP
TILGVREHVFTGAVSSLASFMSAQETSFVTLGQRVLANPLKVRMHYGHPDVFDRLWFMTRGGLSKASKVINISEDIYAGF
NCTLRGGTVTHHEYIQVGKGRDVGLNQISMFEAKVASGNGEQILSRDLYRLGHRLDFFRMLSVYYTTVGFFVNNMLVVL
TVYAFLWGRVYLAVSGIEESITDGANSNTALSAALNQQFVVQMGILTALPMIVENSLERGFLTAIWEFLTMQLQLASVFFT
FSMGTRTHFFGRTILHGGAAYKATGRGFVVRHEKFTTNYQLYARSHFVKGLEIILLLIVYQAYGSSKDTTVYILTTFSCWFLGI
TWILAPFLFNPSGFDLLKAVDDFDEFMTWIWYKGGVFVKDTESWAKWWDVQQEHLQNTGFWGKCLEIVLDLRFFFF
QYGIVYRMKIAAGSTSILVYLLSWTYIFAACVVHLVLVLAGDRYGAKQHIRYRSIQTLILVLLALAIVVLFAFTDFSFWDIFVS
MLAFIPTGWGMLQICQVLRKPFLENTPLWSLITTVARLYELLMGVIVMAPVVLLSWFPGFQAMQTRILFNEAFSRGLQI
NKILVGKNP-----------------------------------------------------------------------------------------------------------------------
-------------------------------------------------------------------------------------------

>BNCU 2018895 Radula lindenbergia 1941

RALGNWEGLVQTAVLHGGRLGEARQGQGVAEKRRHQRDDELGRVVPPSLGQGGSSEININDILQAAEEIQPDDPQVA
RILCEYAYNMAQNLDPESEGRGVLQFKTGLLSVIRQKQSKKEGERIDRSNDIQRIQRFYDFYRRKHQIDDLERMERDGAS
LHEDPVEHERRMQKMRKVYRTSRILNIVLNALTRDSTQEEMDSWISKELKDQMETDANKIAEFKPYNILPLETPVTAETI
MTFEEVTAGVASLEHTRDLPDFPEDYRRPEHRKIDIFDVLEYIFGFQEGNVNNQREHLVLSLANRQSMLGLPTESLKLDD
DAINVCFKRLLDNYVKWCEYLRMAPITERAETGQRKLMLMALYLLIWGEAANLRFLPECLCYIFHNMADELFSKDLLSGH

DLQRSKTYRDSNEKDGKQYSFLEQVISPIYQTVLAEAKSNKGGKASHATWRNYDDFNEFFWSPKCFVLGWPWRLDAGF
LVKPRKKGVAPDDGKVASTVTGQKKEKRVSKMTFVEHRSFLHMYHSFHRLWIFFTVMLQAMMVVAFCHERLNTDTFK
KMLSVGPTYLIMKSVQSVFDLLFIYGAYASTRGWSFSRIFARLLFYCALATIITLLYVKMIQENTRGSASTWYNLYLIVLGSYA
GAQLLATVVLRVPVFRRLTDRCANWTIIRFVFWIHQDRYFVGRGLYERNRDYLGYSLFWVFVLACKFAFSYHFQIKSLVEP
TQTIVKLTGGQLRYKWHDVISENNHNALTVASIWAPVIMIYYLDTQVWYTVMSAIVGGIVGARERLGEIRSLGMLRKRFS
SFPEETTRNLVPSRGQTQQMTRSQSTVGVQAGWSKNKIEAVKFAPLWNEIIKSLREEDYINNREKELLLMPANQGHARF
VQWPLFLLANKVYMAVEMAQDNKSTNQIQLWERIEKDEYMAFAVHEAYNSLEVIMKALLVNDEAKTWVRSVFGDVE
GGIQESMLVGHFHLRQVDLVLTKIRVLLALLIHPEDDISKRKAVVAMQGLYETVMHDFLSVELREKFESWTVLTQAKNSG
RLFANLKWPETQEQKDAVRRLHNLLTIKESSANIPRNLEARRRLQFFTNSLFMNMPVAPSVRKMLSFSVFTPYYAEDVM
YSKEQVRSENEDGVSILFYLQKIFPDEWKNFLERVGFTHKVLMDQLEVPDLDILELRLWASFRGQTLARTVRGMMYYKK
ALVLASLLEGSGGLDLEEGLHDSHGEGDSNFGYPKARAEAEMKFTYVVSCQKYGAQKQQKDMRAADIAYLMRTYESLRI
AYIDTVETLKDERLATVYYSKLLKADIDGNDQEIYSVRLPGDFILGEGKPENQNHAIIFTRGDALQTIDMNQDNSFEEAFK
MRNLLEEFKAQNCERRPSILGIRENVFTGSVSSLAWFMSQQEGSFVTLGQRVLARPLKVRMHYGHPDVFDRIFHITRGG
ISKASKTINISEDIFAGFNSTLRQGNVTHHEYIQVGKGRDVGLNQIAIFEAKVSGGNGEQILSRDVFRLGQLFDFFRMLSFF
YTSVGYYITTLMTTLVVYFFLYGKCYLALSGLDEQLREVADISGNAALSSALNTQFLLQIGVFTAIPMIVNFMLELGVLKAVIS
FVTMQLQLCSVFFTFSLGTKTHYFGRTVLHGGAKYLATGRGFVVQHIPFGENYRLYSRSHFTKALEIVTLLIVYLAYGSDNS
GITYVLLSFSSWFLAISWLFAPYIFNPSGFEWQKTVEDFDDWTNWLFYKGGVSVKGAESYESWLEEEQAHIKTWRGRIF
EILLSVRFFLFQYGVVYALNVSGRTTSLAVYGYSWIVFAALVVLYKVFTITNQASINFQLFLRLFQGVLFLGCIAAVVVCIVLTS
LTVGDCFAILLAFIPTGWGLLCVALAIKPFLEQLHVWGFFLSAFRMYDAIMGGIIFLPIALLSWFPFVSTFQTRLVFNQAFSR
GLEISLILAGNRPNAST------------------------------------

>BNCU 2087280 Radula lindenbergia 1917

SRTYTAANFSDVFDSEVVPSSLSSIAAILRVANEIESQRPRVAYLCRFYAFEKAHKLDPTSSGRGVRQFKTALLQRLERDNIP
TLSARHRRSDAREMQYFYQQYYDTYVKALDGAEHSDRAQLAKAYQTAGILFEVLTAVNKSEQSEPPPEIIAASKIVEEKQEI
LQPYNILPLDAAGASQAIMQLPEIKAAVESLRNIRGLPWPPGWEQGRHKSGELDLLDWLQSMFGFQKASVANQREHLI
LLLANVHIRLIPKPEPMNKLDDRALNQVMDKLFKNYKSWCKFLGRKNSLWLPTIPQEIQQRKILYMGLYLLIWGEAANLR
FMPECLCYIYHNMAYELFGMLAGNVSLATGENIKPAYGGDEESFLRKVITPIYEILEKESKSNCNGTAPHSAWRNYDDLNE
FFWSVDCFRLGWPMRLDSDFFVPPAQYKSKVPKLDIKKTGGATPSYLGKSNFVEIRSFAHLFRSYDRMWTFYILGLQALIV
IAWSIEPNGSTRDLFEGDNVKTILSIFITAAILRCIQGVLDIALSVHAFRSLKLLGFTRLILKLCLSGAWVIVLSVCYVHTWSNP
TGLVRSVQQALGSSWKNPSLYITAVVIYLVPNILGAILFVFPMLRRWIENSNWKIVRFFMWWSQPRLYVGRGMHESQW
TLFKYSVFWILLLGSKFVFSYYIQIRPLVRPTKTIMNARDIRFRWHEIFPHVKNNLGAVLSIWAPVVLVYFMDTQIWYAFYS
TIFGGISGAFRRLGEIRTLGMLRSRFRSLPGAFNANLVPAEKVSRRGFSLARGYKEVQPGKDRMEASKFAQLWNEVIMSF
REEDLISNKERDLMLVPYSSGNLTLVQWPPFLLASKIPIALQMALEHRGNDADLWRKIKVDDYRRCAVEECFESFKHVLG
VILVGELERRVVIDGILAEIEKNIAEGTLLANFKMSALPILHSKFVELTEYLIKGEESSRKYVVKRLQDMFEVVTRDMMDETR
REYLESTLGPLSISGKTSKDVKDHQLFAAAEPKPALLYPPPRTDAWIEQIKRMHRLLTVKETAVDVPTNLEARRRIAFFTNSL
FMDMPRAPRVRNMYSFSVLTPYYHEEVLFSKQQLNEENEDGVSVLFYLQKIYPDEWDNFLERMKLQDERQFWNNDEY
VDELRHWASYRGQTLSKTVRGMMYYRRALELQAFLDMASDDELVEGYKVVASAPADTKKSQRSMWAQLQAIADMKF
TYVATCQSYGAQKRAGDVRATDILNLMLNNPSLRVAYIDECEDEEKDKDQKVYYSVLVKAANGLDEEIYRIRLPGPVRLGE
GKPENQNHAMIFTRGEGLQTIDMNQDNYLEEAFKMRNLLEEFHEPHGVRPPTILGVREHIFTGSVSSLAWFMSNQETS
FVTIGQRVLASPLKVRFHYGHPDVFDRLFHITRGGVSKASRIINLSEDIFAGFNSTLRRGNITHHEYIQVGKGRDVGLNQIS
LFEAKVACGNGEQTLSRDMYRLGHRFDFFRMLSCYFTTVGYYFSTMIVVLTVYIFLYGRIYLALSGIERSLVSSADINNDSAL
QAALASQSLVQLGLLMALPMVMEIGLERGFRTALSDFVIMQLQLASVFFTFSVGTKTHYYGRTILHGGAKYRATGRGFV
VRHEKFAENYRLYSRSHFVKGIELLMLLIIYSVYGGSSVSRAGIPYLLITFSMWFLVATWLFAPFLFNPSGFEWQKIVEDWD

DWSKWINNRGGIGVQADKSWESWWSEEHEHLHTTGLRGRLIEILLSLRFLIYQYGLIYTLDITRGSNGLGVYGFSWVVVL
AVLTVLKVVSMGRKRFSADFQLMFRLLKAVLFVGFLSILIVLFIFAHLSVSDIFASLLAFLPTGWALLMIAQAAKPIVRPTGM
WDSVKALARAYDFIMGLVIFTPVAVLAWFPFVSEFQTRLLFNQAFSRGLQISRILAGRKK------------------------------------
-----------------------

>CHJJ 2004303 Lejeuneaceae sp 1917

RARTNWELLVRNALRSPELIGRSHPGRTEELGGVVPPSLGQESDINVILQAAEEIQPDDPQVARILCEYAYSMAHNLDPQ
SEGRGVLQFKTGLMSVIRQKQSKREGEKIDRSRDIQRIQEFYEFYRKKHQIDELERRQRENPIHEDPSETQRRNQKLKKVY
QTSRVLNDVLNALTRDATPEERDKLIQKELKDRMETDALKTLDYKAYNILPLETPGVADAIMGFEEVKAAAASLEHTSDLP
KFPDDYRRPEQRPVDIFDLLEYIFGFQEGNVNNQREHLVLLLSNSQSLLGQPTDTSMLDEMAISRCFDRLLDNYMKWGD
YLRLVSVTSRAETRSRKLLLMAMFLLIWGEAANLRFLPECLCYIFHQMVDELLGILGGRDVQRSKTYRPSEHDGKQYSFLD
QVVAPIYLVVLAEAKNNNGGKASHAAWRNYDDFNEFFWSPKCFVLQWPWRLDAGYLMKPRKKGVVADEGEPAELGR
IREKRVGKMTFVEHRTYLHVYHSFHRLWIFFTVMLQALMVVAFCNEKLNIRTFKKMLSVGPTYLTMKSVQSLFDLLFIYG
AYASTQGWAFSRIFTRLIFYVGACVGITFLYAKMMQEDGQGSSSAWYHLFLVVLGSYAGAQLLVTAILRIPLFRRQLDKCS
NWSIIRFVYWVHQDRYFVGRGLYERTRDYFAYTTFWVFVLACKFAFSYHFQIRSLVSPTKTIVNLPSNRLQYKWHDFFSAK
NHNALTVGAIWAPVVMIYYLDIQVWYTVLSALVGGLSGAKARLGEIRTLSMFRKRFSTFPGEMSKNLAPTRTLPQMNRS
QSTASQTGSTKPKSDAANFSPLWNEIIISLREEDYINNWEKELLVMPGNNDDGKLVQWPLFLLANKVYIAVEMAQDNKS
TNQYQLWERIEKDEYMAYAVEEAYRTLEVVLRALVNDEAKTWVRSVFGDVENGILEGMLVGHFHLQNISLLLTEVRKLSS
LLIRVEDENGKRDAVKAMQDLYETVMHDFLSVELREKFESWALLAQAKNDGRLFAILNWPTSREEKDRVRRLNNLLTIKE
SAANIPTNDEAKRRLQFFTNSLFMHMPVSPPVRKMLSFSVFTPYYEEDVLYSKEEVKRKNEDGVSTLYYLQTIYPDEWKN
FLQRVGLTNEEIKRQLENEKLDMLDLRLWVSFRGQTLARTVRGMMYYKKALVLASLLEGSGTDDIEEGLQSISGGAEMN
PGFRKARALAELKFTYVVTCQIYGVHKRRKDPRARDIQYLMKIHDSLRIAYVDLVESLKDEKNTTTYFSKLIKADVDGNDQ
EIYSIKLPGEFRLGEGKPENQNHAIIFTRGDAVQTIDMNQDNYFEEAFKMRNLLEEFKAENCLRKPTILGIRENVFTGSVSS
LAWFMSQQEGSFVTLGQRVLARPLKVRMHYGHPDVFDRIFHITRGGISKASKTINISEDIFAGFNSTLRQGNVTHHEYIQ
VGKGRDVGLNQIAIFEGKVAGGNGEQLLSRDVFRLGQLFDFFRMLSFFYTSVGYYITTLMTTLVVYLFLYGKCYLALSGLD
RQLQIVADISGNAALNSALNTQFLLQIGVFTAVPMIMNFILEKGVLKAVISFITMQLQLASVFFTFSLGTKTHYFGRTVLHG
GAKYSATGRGFVVKHISFAENYRLYSRSHFVKALEIAMLLITYLAYGEDNSGVAYILLSFTSWFLAISWLFAPYIFNPSGFEW
QKTVEDFDDWTNWLFYKGGISVKGAESWESWLEEEQAHIRTLRGRLWEIVLSARFFFFQYGVVYSLHISGKSTSLAVYGY
SWIVFVGIIVLSKVFTFTNQASLNFQLFLRLFQGLLFLGSLAGLGVLIAFTSLSVGDCFAIFLAFIPTGWGLLSIALATKPVLEP
LHIWGFVQSGFRLYDALMGAIIFMPIAFLSWFPFVSTFQTRLVFNQAFSRGLEISKILAGDRPNVS----------------------------
---------------------------------

>CHJJ 2004304 Lejeuneaceae sp 1510

RARTNWELLVRNALRSPELIGRSHPGRTEELGGVVPPSLGQESDINVILQAAEEIQPDDPQVARILCEYAYSMAHNLDPQ
SEGRGVLQFKTGLMSVIRQKQSKREGEKIDRSRDIQRIQEFYEFYRKKHQIDELERRQRENPIHEDPSETQRRNQKLKKVY
QTSRVLNDVLNALTRDATPEERDKLIQKELKDRMETDALKTLDYKAYNILPLETPGVADAIMGFEEVKAAAASLEHTSDLP
KFPDDYRRPEQRPVDIFDLLEYIFGFQEGNVNNQREHLVLLLSNSQSLLGQPTDTSMLDEMAISRCFDRLLDNYMKWGD
YLRLVSVTSRAETRSRKLLLMAMFLLIWGEAANLRFLPECLCYIFHQMVDELLGILGGRDVQRSKTYRPSEHDGKQYSFLD
QVVAPIYLVVLAEAKNNNGGKASHAAWRNYDDFNEFFWSPKCFVLQWPWRLDAGYLMKPRKKGVVADEGEPAELGR
IREKRVGKMTFVEHRTYLHVYHSFHRLWIFFTVMLQALMVVAFCNEKLNIRTFKKMLSVGPTYLTMKSVQSLFDLLFIYG
AYASTQGWAFSRIFTRLIFYVGACVGITFLYAKMMQEDGQGSSSAWYHLFLVVLGSYAGAQLLVTAILRIPLFRRQLDKCS

NWSIIRFVYWVHQDRYFVGRGLYERTRDYFAYTTFWVFVLACKFAFSYHFQIRSLVSPTKTIVNLPSNRLQYKWHDFFSAK
NHNALTVGAIWAPVVMIYYLDIQVWYTVLSALVGGLSGAKARLGEIRTLSMFRKRFSTFPGEMSKNLAPTRTLPQMNRS
QSTASQTGSTKPKSDAANFSPLWNEIIISLREEDYINNWEKELLVMPGNNDDGKLVQWPLFLLANKVYIAVEMAQDNKS
TNQYQLWERIEKDEYMAYAVEEAYRTLEVVLRALVNDEAKTWVRSVFGDVENGILEGMLVGHFHLQNISLLLTEVRKLSS
LLIRVEDENGKRDAVKAMQDLYETVMHDFLSVELREKFESWALLAQAKNDGRLFAILNWPTSREEKDRVRRLNNLLTIKE
SAANIPTNDEAKRRLQFFTNSLFMHMPVSPPVRKMLSFSVFTPYYEEDVLYSKEEVKRKNEDGVSTLYYLQTIYPDEWKN
FLQRVGLTNEEIKRQLENEKLDMLDLRLWVSFRGQTLARTVRGMMYYKKALVLASLLEGSGTDDIEEGLQSISGGAEMN
PGFRKARALAELKFTYVVTCQIYGVHKRRKDPRARDIQYLMKIHDSLRIAYVDLVESLKDEKNTTTYFSKLIKADVDGNDQ
EIYSIKLPGEFRLGEGKPENQNHAIIFTRGDAVQTIDMNQDNYFEEAFKMRNLLEEFKAENCLRKPTILGIRENVFTGSVSS
LAWFMSQQEGSFVTLGQRVLARPLKVRMHYGHPDVFDRIFHITRGGISKASKTINISEDIFAGFNSTLRQGNVTHHEYIQ
VGKGRDVGLNQIAIFEGKVAGGNGEQLLSRDVFRLGQLFDFFRMLSFFYTSVGYYITTLVSLLNLVPLLY---------------------
-----------------------------------------------------------------------------------------------------------------------------------
-----------------------------------------------------------------------------------------------------------------------------------
-----------------------------------------------------------------------------------------------------------------------------------
--------------------------------

>CHJJ 2005114 Lejeuneaceae sp 1409

YNIIPVHDTLSDHPALHFPEVRAAIAALQVVGDLRIPFEGWVPGMDMMDWLGLTFGFQEGNVKNQREHLVLLLANSQ
MRLQPPPDPIDKLVLDVVRRLRQKVTKNYLSWCHFLHLTHNLEKLEGRRHESDERKELMYTALYFLIWGEAANLRFMPE
CLCFIFHNMSGELNKILKLSIDDAGQRIQPASYGVNGFLRFVIFPIYEVVQAEAVAGGGGAPHSAWRNYDDMNEYFWSK
RCMTRLGWPLAKTCNYLVKPAKGKGKVSRQKVGKTGFVEQRSFWNVFRSFDRLWVGLILLLQVLITVAFEAKGRRTGQS
DRMPWTVLSRRDSQAHVLAIFITWSALRVLQALLDFGMQYSLISAGRVGIRMTLKLLVASVWVVLFSVYYARMWAQRH
RDRGWTSGALERLYVYLGFCLVFITPEVLALLLFIIPYVRNAVETSNWRIFAPFTWWFQSKIFVGRGLREGFFDNLKYTLF
WIAVLAAKIAFSYFLQILPLVKPTRATLNIRDGTIDYKWHEFFKNANRMALLCMWAPVILIYFMDLQIWYSLFSALVGALV
GLLGHLGEIRDSTQIRSRFRFFPMAVIFNLMPEEKLLKQRPFYNTWGRLKNAIRRIKLRFGFQGRKKDAAARSMEVAKFK
QIWNEIIGIFREEDIISNEERELLEMPTTGWNISVTQWPLFLLSNEVRIALRFCDPEHHHYWYHSDKWLWRKVSSNEYRR
CAVIECYESLKHLLTRLIRPSSDDAQIFPLIFREIDNAIHQGLFMKTFKMKELPKLHESLITLTGILMKRPLLNDLQLVVDALQ
NLYEIAVRDFPRDLNVKIKFKELASRSLLFVEAVELPNPEGDDTFFYQLKRLHNSLSTRTAYLNVPKSLEARRRISFFSNSIFM
TMPRAPHVERMLAFSVLTPYYKEDVLYDKKQLYTQNEDGVSTLFYLQKIFPDDWRHFQERMAGRGIHTDEETMWEHE
DGLEIRLWASYRGQTLVRTVRGMMYYGRALQKLAFLDLASEHDIDGYKELLSRTASSMEITTTTGSRHSSFGYGRSARSFR
APEMYDGHQKEQDTALAAMKFTYVVACQVYGEQKRQNDAKAADILYLMETYEALRVAYFDQTGGDREPKRYYSVLVKF
DPILQQEVEIYRVELPGPLILGEGKPENQNHALIFTRGDAIQTIDMNQENYFEEALKMRNLLQEFDKSRGIRKPTILGVRE
HVFTGAVSSLASFMSAQETSFVTLGQRVLANPLKVRMHYGHPDVFDRLWFLSRGGMSKASKVINISEDIYAGFNCTLRG
GTVTHHEYIQVGKGRDVGLNQISLFEAKVASGNGEQMLSRDLYRLGHRLDFFRMLSVYYTTTGFFINNMIVVLTVYAFL
WGRCYLAVSGIEESISGSAKSNTALSAALNQQFLVQMGILTALPMIVENSLE------------------------------------------------
-----------------------------------------------------------------------------------------------------------------------------------
-----------------------------------------------------------------------------------------------------------------------------------
-----------------------------------------------------------------------------------------------------------------------------------
-----------------------------------------------------------------------------------------------------

>CHJJ 2005115 Lejeuneaceae sp 1763

YNIIPVHDTLSDHPALHFPEVRAAIAALQVVGDLRIPFEGWVPGMDMMDWLGLTFGFQEGNVKNQREHLVLLLANSQ
MRLQPPPDPIDKLVLDVVRRLRQKVTKNYLSWCHFLHLTHNLEKLEGRRHESDERKELMYTALYFLIWGEAANLRFMPE
CLCFIFHNMSGELNKILKLSIDDAGQRIQPASYGVNGFLRFVIFPIYEVVQAEAVAGGGGAPHSAWRNYDDMNEYFWSK
RCMTRLGWPLAKTCNYLVKPAKGKGKVSRQKVGKTGFVEQRSFWNVFRSFDRLWVGLILLLQVLITVAFEAKGRRTGQS
DRMPWTVLSRRDSQAHVLAIFITWSALRVLQALLDFGMQYSLISAGRVGIRMTLKLLVASVWVVLFSVYYARMWAQRH
RDRGWTSGALERLYVYLGFCLVFITPEVLALLLFIIPYVRNAVETSNWRIFAPFTWWFQSKIFVGRGLREGFFDNLKYTLF
WIAVLAAKIAFSYFLQILPLVKPTRATLNIRDGTIDYKWHEFFKNANRMALLCMWAPVILIYFMDLQIWYSLFSALVGALV
GLLGHLGEIRDSTQIRSRFRFFPMAVIFNLMPEEKLLKQRPFYNTWGRLKNAIRRIKLRFGFQGRKKDAAARSMEVAKFK
QIWNEIIGIFREEDIISNEERELLEMPTTGWNISVTQWPLFLLSNEVRIALRFCDPEHHHYWYHSDKWLWRKVSSNEYRR
CAVIECYESLKHLLTRLIRPSSDDAQIFPLIFREIDNAIHQGLFMKTFKMKELPKLHESLITLTGILMKRPLLNDLQLVVDALQ
NLYEIAVRDFPRDLNVKIKFKELASRSLLFVEAVELPNPEGDDTFFYQLKRLHNSLSTRTAYLNVPKSLEARRRISFFSNSIFM
TMPRAPHVERMLAFSVLTPYYKEDVLYDKKQLYTQNEDGVSTLFYLQKIFPDDWRHFQERMAGRGIHTDEETMWEHE
DGLEIRLWASYRGQTLVRTVRGMMYYGRALQKLAFLDLASEHDIDGYKELLSRTASSMEITTTTGSRHSSFGYGRSARSFR
APEMYDGHQKEQDTALAAMKFTYVVACQVYGEQKRQNDAKAADILYLMETYEALRVAYFDQTGGDREPKRYYSVLVKF
DPILQQEVEIYRVELPGPLILGEGKPENQNHALIFTRGDAIQTIDMNQENYFEEALKMRNLLQEFDKSRGIRKPTILGVRE
HVFTGAVSSLASFMSAQETSFVTLGQRVLANPLKVRMHYGHPDVFDRLWFLSRGGMSKASKVINISEDIYAGFNCTLRG
GTVTHHEYIQVGKGRDVGLNQISLFEAKVASGNGEQMLSRDLYRLGHRLDFFRMLSVYYTTTGFFINNMIVVLTVYAFL
WGRCYLAVSGIEESITGSAKSNTALSAALNQQFLVQMGILTALPMIVENSLERGFLRAIWEFLTMQLQLASVFFTFSMGTR
AHYFGRTILHGGAAYKATGRGFVVRHEGFTANYQLYSRSHFVKGIEIILLLIVYQTWGSSKNTVVFVLTTFSCWFLGITWIM
GPFLFNPSGFDWLKSVNDFGEFMGWIWYKDDFLFVKATDSWKKWWDDEQEHFRHTGFWGKVMEIILDLRFFFFQYG
IVYRLQIANGSTSILVYLLSWTYIFAFGVIHLVLAFSGERYGAKEHLKYRSIQALVIMLLLLAVVILFVFTSFSIWDIFTSLLAFLP
TGWGLILICQVLRRPFLENTPVWPMVVAVARLYDLVMGIFVMAPVVVLSWLPGFQAMQTRILFNEAFSRGLQFSQIVA
GR-------------------------------------------------------------------------------------------------------------------------------
-------------------------------------------------------------------------------

>HMHL 2009140 Marchantia paleacea 1914

ERAWNNWERLIQNALRSEELRFSVAGGDDLGGVVPTSLGQQSSNINLILQVAEEIQPDNPHVARILCEYAYTMAQQLDP
QSEGRGVLQFKTGLMSVIRQKQSKKEGERIDRSHDIIQIQRFYEEYRKRHRIDELEKLQQQRGRRLSEDPDEAQRRAQTM
KKVYQISKVLNDVLNSLTENSTEEERDKLISKETKARMESAAAKTAEFKAFNILPLETPGVSDTILLIEEVRAAALTLSYSDELP
RLPESAFKAGLQRDVDIFDLLEYIFGFQIGNVNNQREHLILLLSNSQSAFGPPSRGSEVDEAAIKRCYDRLLDNYTKWCQY
LRITAVTESATGDPRKMVLLTAMYLLIWGEAANLRFLPECLCYIFHHMVKELYGLLGNTDAQRSMNYVEGSDCPFLDKIIT
PLYSVIAAEAENTQEGKASHAAWRNYDDFNEYFWSPRCLVWPWRPDAGFMMKPNTKKLKGAANALQGRKKEKKVG
KMTFVEHRTGFHVYHSFHRLWIFFTVMLQGLMIFAFCDEKFTPNTFKKMLSVGPTFVIMKLIQSLLDIFFIYGAYASTQG
WTFSRIFARLAWFGGLSGSITYLFVKMIQEENNGTGSTWYHLYLIVLGSYAAAQVVVTLLMRILFSRRKADKCGDWAIIRF
VMWVHQDRYFVGRGLYERTRDYVGYSAFWIFILACKFAFSYYFQVRALVAPTKDIVVQRFEYKWHDFVSRNNHNALTIG
ALWAPVILIYYLDTQVWYTVVSAIVGGLDGASARLGEIRTLSMLRKRFSSFPEAFATNLVSSRLGRGEKNPGSSPAQVNWS
MQKVHAFKFAPMWNEIINCLREEDYISKKERDLLVMPKNQGSGALVQWPLFLLANKVYLAVEMAQDSKLLNQTQLWE
KVCKDEYMAYAIEEAYKVLEEVLKNLVREDVSQIWVKGVFNDVGAGIQESALVGHFYLKKVDSVLARVTALTTLLIHEETEK
LKSQVILAMQNLYESVMNDFFTTELRERIEEFRALKDARIDGKLFLNLTWPTTDREKENVKRLHYLLTIKESAANIPRNLEA
RRRMEFFTNSLFMDMPKPPSVQRMLSFSVFTPYYSEDVLYSKGKLTEENEDGISILFYLQKIFPDEWKNFLERQGLTNAL
MERQMEDNNNDCLELRLWASYRGQTLARTVRGMMYYKKALILQSLLEGTSDVEEGVLGSTLEESSGYRMARAIAETKF
TYVVTCQIYGQQKEKRAQQAADILYLMNKYDSLRIAYVDIVDKMQEDKESKEYKSIKEYYSKLIKADADGKDQEIYSIKLPG
QFKLGEGKPENQNHAIVFTRGDACQTIDMNQDNYFEEAFKMRNLLEEFNSTDSLRRPTILGIREHVFTGSVSSLAWFMS

QQETSFVTLGQRVLARALKVRMHYGHPDVFDRIFHITRGGISKASRTINISEDIYAGFNSTLRQGNITHHEYIQVGKGRDV
GLNQIAMFEAKVSSGNGEQILSRDVFRLGQLFDFFRMFSFFYTSVGYYITTLMTTLVVYVFLYGKVYLALSGMDKQLQTFA
DINSNKALESALNTQFLLQIGVFTAVPMIMNFILEQGVLKAVISFLTMQLQLCSVFFTFSLGTKTHFFGRTVLHGGAKYRAT
GRGFVVQHISFADNYRLYSRSHFNKALEISMLLITYLSYGEDNRGVAYVLLSFSSWFMALSWLFAPYIFNPAGFEWQKTVE
DFEDWTNWLFYKGGIGVKDNESWESWWEAEQEHIRTLRGRFWEIVLSLRFFFFQYGVVYSLQVSGSSTSLSIYGYSWLA
FLLLIVLFQIFTFTNKSSVKFQLFLRLFQGGLFLCLIAAVAVTIAFTSLSVGDCFATLLVFIPTGWGVLSICQAIRPILEPLGAWN
AVRSGFRLYDAMMGAVIFTPIAILSWFPFVSTFQTRLVFNQAFSRGLEISLILAGNRPNAST----------------------------------
-----------------------------

>HMHL 2011621 Marchantia paleacea 1779

YNIIPVHNTLDSHPALRFPEVRAAIAALQVVGDLRKPSNMSWTPNLDMLDWLCFTFGFQESNARNQREHLVLLLANAQ
MRMQPPPDPIDKLQLEIVREIRKKITKNYIRWCSFLRIKHNLEKIEDHGIRRRHESNEQKELLYVSLYLLIWGEAANLRFMP
ECLCFIFHHMCGELNRILDRSIDDSGQYMQPASGGIQNGFLQKVIRPIYAVVKAEAQECRAGKNPHSAWRNYDDMNEY
FWSKRCLTQLRWPLEMSCNYLVKPEDKSRHKVGKTGFVEQRSFWNIYRSFDRLWIGLILMLQILVIVAFRAKPEIANPDLA
PSAQGTTTGPAKAPWTILAQRDSQAHVLSIFITWSALRVLQAFLDFGMQYSLISAGTVMVGLRMTLKLIMGAVWTVLFS
IYYSRMWAQRNADGLWSAKALQGLYLYLGFCAVFVTPEALALLLFILPYIRNFVETRDWRIFSMLTWWFQSHIFVGRGLR
ESIFDNIKYTLFWLCVLTAKFSFSYFLQILPLVSPTRATLDVTGIEYRWHEFFKNANRTATLAMWAPVVLIYFMDLQIWYSV
FSALVGALVGLLAHLGEIRNAAQLKARFHIFPVAVQFNLMPEDIFVNSKPYSWAAKCNEWWVKVKNLARRVKLRYGVVK
GHQTEGKTMETGRFRHVWNEIILIFREEDLISDRELELLTMPSSRWNISVTQWPSVLISNEVLIALGLCKDWYYTDHGLW
KKIASNEYRRCAVIESYESIRHVLKRILREDSNEYQMFQEIFDEITRAIKESQFVQRFNLKALSNVHTRLVQLINVLMKRPLL
NDLQKVVDALQNLYEDLLRDFPKDQELGRNWREMSATSSLLFVDAVDLPDVADNTFFDQLRRVQTTLSTREALLDVPR
NLEAKRRIAFFSNSLFMTMPRAPQVDKMLAFSVLTPYYSEDVMYHKKQLITENEDGVSILFYLQKIFKDDWENFQERMA
RQGIRTEIDMWKLDDGLELRLWASYRGQTLARTVRGMMYYHRALQKLAFLDVASEHDILEGYRELMERTSGGVDSFN
GELREGSVHSASSHTDGKEGMVYSDYQREMDLATTAMKYTYVVACQIYGIQKAKGQQQAKDILYLMKTWEALRVAYV
DEKPGQGDKDPKRYASVLIKYDQVRQEEVEIYRVELPGDFKLGEGKPENQNHALIFTRGDAVQTIDMNQENYFEEALKM
RNLLQEFKQLHGRRRPAILGVREHVFTGAVSSLAWFMSAQETSFVTLGQRVLAKPLKVRMHYGHPDVFDRLWFITRGG
LSKASRVINISEDIFAGFNATLRGATVTHHEYIQVGKGRDVGLNQIALFEAKVASGNGEQMLSRDVYRLGHRVDFFRMLS
VYYTTVGFFVNNMIVVLTVYAYLWGRVYLALSGIEVSITRDSSANGALTAALNQQFVVQMGVFTALPMIVENSLEKGFLL
AIWEFLTMQLQLASVFFTFSMGTRTHYFGRTILHGGAKYRATGRGFVVKHEKFSDNYRQYARSHFVKGLEIIVLLIVYQMY
GSIRNTTTYILTTFSCWFLGITWILAPFLFNPSGFDWLKSVDDFDDFMTWIWYKGGVFVKGEDSWQKWWDEEQEHFQ
STGFWGKCLEIILSLRFFFFQYGIVYRLKVSSGSTSIVVYLISWTYLFAAGLIHLILSHAAEKYGAKRHRKYRGIQAFIISFLVLA
VVLLFVFTDFSIWDIFTSILAFVPTGWGVLSICLVLRRPFLENTPVWPMVTGVARLYEMGMGIGVMTPVVVLSWLPGLQ
AMQTRILFSEAFSRGLSIQRLLAGRK-------------------------------------------------------------------------------------------
---------------------------------------------------------------------------------------------------

>HMHL 2055394 Marchantia paleacea 1909

TANNSDVFDSEIVPSSLSAVAPILRVANEIESHRPRVAYLCRFYAFEKAHKLDPTSSGRGVRQFKTSLLQRLERDNKPTLRAR
QKRSDARELQSFYQQYYENYVKALDGAEHSDRAQLTKAYQTAGILFEVLTQVSKSEMAEAPPEIIAAGKIVEEKQEIYLPYN
ILPLDAAGASQAIMQLPEIKAGVEALRNIRGLPWPSQMEQARHKSGELDLLDWLQCMFGFQKDNVRNQREHLILMLA
NVHIRLIPKAEPMSKLEDRALNEVMDKLFKNYKSWCKFLGRKNSLWLPTIQQEIHQRKILYMGLYLLIWGEAANLRFMPE
CLCYIYHNMAYELYGMLAGNVSVVTGENIKPAYGGEDESFLKKVITPIYDIIFKETKNNGHGTAPHSAWRNYDDLNEFFW

SVDCFRLGWPMRLDADFFVPPSQSSLSSKLSIGKGGKVASTRRLGKTNFVEIRSFWHLFRSYDRMWTFYILGLQAMIVM
AWNIEGSGTFKSTFEGNNFKRILSIFITAAILRVIQGLLDIAMSMKAYRSIKFMGMLRLFLKLLVSIAWVIVLSVCFVHTWE
NPTGLIKNVQNVLGSSWKNPSLYITAVAIYLLPNALAAVLFVFPLMRRWIENSNWRLVRLLLWWSQPRLYIGRGMHESQ
WTLFKYTMFWILLLASKFVFSYFIQIKPLVKPTKTIMNDNSIQFTWHELFPNVKKNVGAIIAIWAPVILVYFMDTQIWYSV
YSTVFGGVSGAFRRLGEIRTLGMLRSRFRSLPGAFNANLVPADKVARRGFSLARGYKEVQPGKDRKEAAKFAQLWNEVIT
SFREEDLISNKEMDLMLVPYSSVNLTLVQWPPFLLASKIPVALQMAVEHRGRDMDLWRKIRADDYMRCAVEECFESFKH
VLGTILVGEVERRLVIDGILEEIDKDISEGSLLSNFKMSALPVLHTKFVQLTEFLIKGEADKRDSVVLLLQDMFEVVTRDMM
NETAREYLESTHGPLSISGKTSKDVKDHQLFAATDPKPAVLFPPPATDAWIEQIKRLHRLLTVKESAINVPTNLEARRRIAFF
TNSLFMDMPRAPRVRNMLSFSVLTPYYQEQVVYSKKQLNEENEDGISVLFYLQKIYPDEFDNFLERINVTSEHEIWENEE
YENELRHWASYRGQTLARTVRGMMYYRRALELQAFLDMASDDELVDGYKVVASAPAEAKKSQRSMWAQLQAIADM
KFTYVATCQIYGAQKRAADVRATDILNLMLNNPSLRVAYIDEVEEREKDKNQKVYYSVLVKASNGLDQEIYRIKLPGIVKLG
EGKPENQNHAMIFTRGEALQTIDMNQDNYLEEAFKMRNLLEEFHEPHGVRPPTILGVREHIFTGSVSSLAWFMSNQET
SFVTIGQRVLASPLKVRFHYGHPDVFDRLFHITRGGMSKASRVINLSEDIFAGFNSTLRRGNVTHHEYIQVGKGRDVGLN
QISLFEAKVACGNGEQTLSRDMYRLGHRFDFFRMMSCYFTTIGFYASTVIVVLTVYVFLYGRIYLALSGIEKSLVNSADVNN
DPALQAALASQSLVQLGLLMALPMVMEIGLERGFRTALSDFIIMQLQLASVFFTFSLGTKTHYYGRTILHGGAKYRATGRG
FVVRHEKFAENYRLYSRSHFVKGIELMMLLIIYSVYGTSAKGGIPYLLITFSMWFLVTTWLFAPFLFNPSGFEWQKIVEDW
DDWSKWINNRGGIGVLATKSWESWWEEEQEHLKYTGLRGRVLEVLLSIRFFIYQYGMVYTLSIARGSTSLSVYGLSWLVII
AVLAVLKIVSMGRRRFSADFQLMFRLLKALLFIGFVTIVIVLFLFAKLSVGDLFASLLAFLPTGWGLLMIAQAARPVVVRSG
MGDSVKALARAYEFIMGLCIFTPVAMLAWFPFVSEFQTRLLFNQAFSRGLQISRILAGRR-----------------------------------
--------------------------------

>IHWO 2011602 Marchantia paleacea 1909

TANNSDVFDSEIVPSSLSAVAPILRVANEIESHRPRVAYLCRFYAFEKAHKLDPTSSGRGVRQFKTSLLQRLERDNKPTLRAR
QKRSDARELQSFYQQYYENYVKALDGAEHSDRAQLTKAYQTAGILFEVLTQVSKSEMAEAPPEIIAAGKIVEEKQEIYLPYN
ILPLDAAGASQAIMQLPEIKAGVEALRNIRGLPWPSQMEQARHKSGELDLLDWLQCMFGFQKDNVRNQREHLILMLA
NVHIRLIPKAEPMSKLEDRALNEVMDKLFKNYKSWCKFLGRKNSLWLPTIQQEIHQRKILYMGLYLLIWGEAANLRFMPE
CLCYIYHNMAYELYGMLAGNVSVVTGENIKPAYGGEDESFLKKVITPIYDIIFKETKNNGHGTAPHSAWRNYDDLNEFFW
SVDCFRLGWPMRLDADFFVPPSQSSLSSKLSIGKGGKVASTRRLGKTNFVEIRSFWHLFRSYDRMWTFYILGLQAMIVM
AWNIEGSGTFKSTFEGNNFKRILSIFITAAILRVIQGLLDIAMSMKAYRSIKFMGMLRLFLKLLVSIAWVIVLSVCFVHTWE
NPTGLIKNVQNVLGSSWKNPSLYITAVAIYLLPNALAAVLFVFPLMRRWIENSNWRLVRLLLWWSQPRLYIGRGMHESQ
WTLFKYTMFWILLLASKFVFSYFIQIKPLVKPTKTIMNDNSIQFTWHELFPNVKKNVGAIIAIWAPVILVYFMDTQIWYSV
YSTVFGGVSGAFRRLGEIRTLGMLRSRFRSLPGAFNANLVPADKVARRGFSLARGYKEVQPGKDRKEAAKFAQLWNEVIT
SFREEDLISNKEMDLMLVPYSSVNLTLVQWPPFLLASKIPVALQMAVEHRGRDMDLWRKIRADDYMRCAVEECFESFKH
VLGTILVGEVERRLVIDGILEEIDKDISEGSLLSNFKMSALPVLHTKFVQLTEFLIKGEADKRDSVVLLLQDMFEVVTRDMM
NETAREYLESTHGPLSISGKTSKDVKDHQLFAATDPKPAVLFPPPATDAWIEQIKRLHRLLTVKESAINVPTNLEARRRIAFF
TNSLFMDMPRAPRVRNMLSFSVLTPYYQEQVVYSKKQLNEENEDGISVLFYLQKIYPDEFDNFLERINVTSEHEIWENEE
YENELRHWASYRGQTLARTVRGMMYYRRALELQAFLDMASDDELVDGYKVVASAPAEAKKSQRSMWAQLQAIADM
KFTYVATCQIYGAQKRAADVRATDILNLMLNNPSLRVAYIDEVEEREKDKNQKVYYSVLVKASNGLDQEIYRIKLPGIVKLG
EGKPENQNHAMIFTRGEALQTIDMNQDNYLEEAFKMRNLLEEFHEPHGVRPPTILGVREHIFTGSVSSLAWFMSNQET
SFVTIGQRVLASPLKVRFHYGHPDVFDRLFHITRGGMSKASRVINLSEDIFAGFNSTLRRGNVTHHEYIQVGKGRDVGLN
QISLFEAKVACGNGEQTLSRDMYRLGHRFDFFRMMSCYFTTIGFYASTVIVVLTVYVFLYGRIYLALSGIEKSLVNSADVNN
DPALQAALASQSLVQLGLLMALPMVMEIGLERGFRTALSDFIIMQLQLASVFFTFSLGTKTHYYGRTILHGGAKYRATGRG
FVVRHEKFAENYRLYSRSHFVKGIELMMLLIIYSVYGTSAKGGIPYLLITFSMWFLVTTWLFAPFLFNPSGFEWQKIVEDW

DDWSKWINNRGGIGVLATKSWESWWEEEQEHLKYTGLRGRVLEVLLSIRFFIYQYGMVYTLSIARGSTSLSVYGLSWLVII
AVLAVLKIVSMGRRRFSADFQLMFRLLKALLFIGFVTIVIVLFLFAKLSVGDLFASLLAFLPTGWGLLMIAQAARPVVVRSG
MGDSVKALARAYEFIMGLCIFTPVAMLAWFPFVSEFQTRLLFNQAFSRGLQISRILAGRR------------------------------------
--------------------------------

>IHWO 2068567 Marchantia paleacea 1779

YNIIPVHNTLDSHPALRFPEVRAAIAALQVVGDLRKPSNMSWTPNLDMLDWLCFTFGFQESNARNQREHLVLLLANAQ
MRMQPPPDPIDKLQLEIVREIRKKITKNYIRWCSFLRIKHNLEKIEDHGIRRRHESNEQKELLYVSLYLLIWGEAANLRFMP
ECLCFIFHHMCGELNRILDRSIDDSGQYMQPASGGIQNGFLQKVIRPIYAVVKAEAQECRAGKNPHSAWRNYDDMNEY
FWSKRCLTQLRWPLEMSCNYLVKPEDKSRHKVGKTGFVEQRSFWNIYRSFDRLWIGLILMLQILVIVAFRAKPEIANPDLA
PSAQETTTGPAKAPWTILAQRDSQAHVLSIFITWSALRVLQAFLDFGMQYSLISAGTVMVGLRMTLKLIMGAVWTVLFS
IYYSRMWAQRNADGLWSAKALQGLYLYLGFCAVFVTPEALALLLFILPYIRNFVETRDWRIFSMLTWWFQSHIFVGRGLR
ESIFDNIKYTLFWLCVLTAKFSFSYFLQILPLVSPTRATLDVTGIEYRWHEFFKNANRTATLAMWAPVVLIYFMDLQIWYSV
FSALVGALVGLLAHLGEIRNAAQLKARFHIFPVAVQFNLMPEDIFVNSKPYSWAAKCNEWWVKVKNLARRVKLRYGVVK
GHQTEGKTMETGRFRHVWNEIILIFREEDLISDRELELLTMPSSRWNISVTQWPSVLISNEVLIALGLCKDWYYTDHGLW
KKIASNEYRRCAVIESYESIRHVLKRILREDSNEYQMFQEIFDEITRAIKESQFVQRFNLKALSNVHTRLVQLINVLMKRPLL
NDLQKVVDALQNLYEDLLRDFPKDQELGRNWREMSATSSLLFVDAVDLPDVADNTFFDQLRRVQTTLSTREALLDVPR
NLEAKRRIAFFSNSLFMTMPRAPQVDKMLAFSVLTPYYSEDVMYHKKQLITENEDGVSILFYLQKIFKDDWENFQERMA
RQGIRTEIDMWKLDDGLELRLWASYRGQTLARTVRGMMYYHRALQKLAFLDVASEHDILEGYRELMERTSGGVDSFN
GELREGSVHSASSHTDGKEGMVYSDYQREMDLATTAMKYTYVVACQIYGIQKAKGQQQAKDILYLMKTWEALRVAYV
DEKPGQGDKDPKRYASVLIKYDQVRQEEVEIYRVELPGDFKLGEGKPENQNHALIFTRGDAVQTIDMNQENYFEEALKM
RNLLQEFKQLHGRRRPAILGVREHVFTGAVSSLAWFMSAQETSFVTLGQRVLAKPLKVRMHYGHPDVFDRLWFITRGG
LSKASRVINISEDIFAGFNATLRGATVTHHEYIQVGKGRDVGLNQIALFEAKVASGNGEQMLSRDVYRLGHRVDFFRMLS
VYYTTVGFFVNNMIVVLTVYAYLWGRVYLALSGIEVSITRDSSANGALTAALNQQFVVQMGVFTALPMIVENSLEKGFLL
AIWEFLTMQLQLASVFFTFSMGTRTHYFGRTILHGGAKYRATGRGFVVKHEKFSDNYRQYARSHFVKGLEIIVLLIVYQMY
GSIRNTTTYILTTFSCWFLGITWILAPFLFNPSGFDWLKSVDDFDDFMTWIWYKGGVFVKGEDSWQKWWDEEQEHFQ
STGFWGKCLEIILSLRFFFFQYGIVYRLKVSSGSTSIVVYLISWTYLFAAGLIHLILSHAAEKYGAKRHRKYRGIQAFIISFLVLA
VVLLFVFTDFSIWDIFTSILAFVPTGWGVLSICLVLRRPFLENTPVWPMVTGVARLYEMGMGIGVMTPVVVLSWLPGLQ
AMQTRILFSEAFSRGLSIQRLLAGRK------------------------------------------------------------------------------------------------
----------------------------------------------------------------------------------------------------

>ILBQ 2007227 Conocephalum conicum 1778

YNIIPIHNTLHPHPALRYPEVRAAIAALKVVGDLRIPPNWRSNYDMLNWLGITFGFQESNVQNQREHLVLLLANAQMRL
QPQPDPVDQLQLNIVRGIRRKLTKNYIKWCSFLRIRHNLEKVEDHGIMRKHESNEQKELLYASLYLLIWGEAANLRFMPEC
LCFIFHNISGELNRILDRSIDDSGQYAQPASQEGFLLKVIKPIYAVVRAEANESGNGKKPHSAWRNYDDMNEYFWSHRCL
TQLRWPLEMSCNYLVKPEDKSRHKVGKTGFVEQRSFWNIYRSFDRVWIGLILMLQILIIVAFRAKREIPNTDLAPSGQTTV
TGPARAPWTVLAERDTQGHILSIFITWSALRVLQAFLDLGMQYSLISAGTLLVGFRMTLKLILASVWTVLFSVYYSRMWA
QRNAVGSWSNDALQRLYLYLGFCGVFVIPEVLALLLFILPYIRNFVETRDWRIFSLLTWWFQSNIFVGRGLRESIFDNFKYS
LFWLCVLTAKFSFSYFLQILPLVKPTRATLEITGINYRWHEVFHNADRTATLAMWAPVVLIYFMDLQIWYSVFSALVGALV
GLLAHLGEIRDAAQLKARFHIFPLAFQFNLMPEETFMKQRPSWRGKAHEWWAATKNLARRIRLRYGVVKSQQAEGKT
METGRFRHVWNEIIIIFREEDIISDRELELLTMPSSAWNISVTQWPSVLISNEVLIALGMCQAWHYTDRGFWKKLSSNEY

RRCAVIESYESTKHVLRRILREDHNEYRIFSEVFHEIERAIQDNQFVHRFELKNLTKIHGRLVELIAILLKRPLLNDLKKVVDAL
QNLYEDLHREFPKDQEIGRRWRDLGATSSLLFVDAVVLPDAEGDDAFFAQLKRLHTTLSTREALLNVPRNLEAKRRISFFS
NSLFMTMPRAPQVDKMLAFSVLTPYYAESVMYNRKQLEDENEDGVSTLFYLRKIFKEDWEHFKERMGHRAIPTKDDDP
FITNMWKVDEGLELRLWASYRGQTLVRTVRGMMYYHRALEKLAFLDVASELEIDGYKELMVRTSDGVDGFNGESMEG
SLRSVSSLTGSKEGGADIEYHKDMDLATAAMKFTYVVACQIYGAQKKKGEQQAKDVLYLMKTWDALRVAYVDEQQPGP
TEKEVKYSSVLIKWDPVRQEEVEIYRVELPGPVKLGEGKPENQNHALIFTRGDSVQTIDMNQENYFEEALKMRNLLQEFK
RYHGKRRPAILGVREHVFTGSVSSLAWFMSAQETSFVTLGQRVLAKPLKVRMHYGHPDVFDRLWFLTRGGLSKASRVIN
ISEDIFAGFNATLRGATVTHHEYIQVGKGRDVGLNQISMFEAKVASGNGEQMLSRDIYRLGHRVDFFRMLSVYYSTVGFF
VNNMIVVLTVYAYLWGRVYLALSGIEVSISQDSSANGALTAALNQQFVVQMGFFTALPMIVENSLEKGFLRAIWEFLTM
QLQLASVFFTFSMGTRTHYFGRTILHGGAKYMATGRGFVVQHERFTANYRLYSRSHFVKGLEIIILLIVYQMYGAVKDTTT
YILTTFSCWFLGITWILAPFMFNPSGFDWLKSVDDFDDFISWIWYKGGVFVKPEDCWRKWWEEEQEHLQSTGFWGKC
LEIILSLRFFFFQYGIVYRLKIASGNTSIIVYLISWTYLFAAGLIQLIISHASNKYGAKRHRKYRGIQAFILSFLVLTVVLLFLFTDFS
IWDILTSILAFLPTGWGVLSICLVLRRPFLENTPVWPMVTAVARLYDMGMGIVIMTPVVVLSWVPGMQAMQTRILFSEA
FSRGLNIQKILTNR-------------------------------------------------------------------------------------------------------------
----------------------------------------------------------------------------------

>ILBQ 2007264 Conocephalum conicum 1863

IQRDNPHVARILCEYAYSMAQQLDPQSEGRGVLQFKTGLMSVIRQKQSKKEGERIDRSHDILQIQKFYEEYRRFHRIDELE
KLQQQRGRVHEDASEVESRTQKMKKVYQISKVLNDVLNSLTENTTEEEKEKLISKETKDKMESAAAKTAEFKPFNILPLET
PGVPDTISMIEEVRAAALTLAYSEELPRLPDSAIKAGLQRDLDIFDLLEYIFGFQIGNVNNQREHLILLLSNTQSAFGPPDRP
NQVDEGAIRRCYDRLLDNYIKWCQYLRLEAMTDRATGEPRLMVLLTATYLLIWGEAANLRFLPECLCYIFHHMVKELYILL
GETDAQRSILNPGEGSETAFLDRVVTPLYRVIAAEAGNSQDGKASHAAWRNYDDFNEYFWSRRCLDCLSWPWREDAG
FMMKPKKKDGKVVINLERGKREKKDGKMTFVEHRTGLHVYHSFHRLWIFFTVMLQALMIFAFSDEKLNARTFKKMLSV
GPTFVIMKFIQSFLDIVFIYGAYASTQGWTFSRIFARLGWFGGLSGAITWLYVKMIEEENSGTGSTTWFHLFMIVLGSYLA
SQLVITLFMRIPYFRRQADKCGDWAIIRFIMWIHQDRYFVGRGLYERTRDYVGYTGFWIIVLAAKFAFSYYFMIKALVEPTK
IIVETPLEYRWHDFVSQRNNNALTIGALWAPVILIYYLDTQVWYTVASAIVGGLDGASSRLGEIRTLSMLRKRFSSFPDAFA
KNLVSAKSGRGERNPGASPSQVNWSLQKLNAFKFAPMWNEIINCLREEDYISKRDRDLLLMPRNQGSGVIVQWPLFLL
ANKVYLAVDMAQDSKSMNQQQLWERVSKDEYMAYSVEEAYKLLEEVLLSLVKHDKAIIWVKGVFNDVNAGIQESALVG
HFYLKKVDSVLSKVIALTTYLVHEETEKLKPSAVRAMQDLYEAVMNDFFTTELRERIEEFKALKEAKLDGKLFVNLEWPNT
EREKENVKRLHYLLTIKESAANIPRNLEARRRLEFFTNSLFMDMPSPPSVQRSLSFSVFTPYYAEDVLYSKKKLKEENEDGISI
LFYLQKIFPDEWKNFLERKGITGKEMDRQLENDNLDLLDLRLWASYRGQTLARTVRGMMYYKKALILQSLLEGNPDVEE
GGFLGATWEESPGYRMARAIAELKFTYVVTCQIYGQQKQNKEQSAADILYLMNEYKSLRVAYVDIVDKIQEDKDGRSVNI
KEYYSKLIKADPDGKDQEIYSVKLPGNFKLGEGKPENQNHAIIFTRGDACQTIDMNQDNYFEEAFKMRNLLEEFNSMNS
LRRPTILGIREHVFTGSVSSLAWFMSQQETSFVTLGQRVLARPLKVRMHYGHPDVFDRVFHITRGGISKSSKTINISEDIFA
GFNSTLRQGNITHHEYIQVGKGRDVGLNQIAMFEAKVSSGNGEQILSRDVFRLGQLFDFFRMMSFFYTSVGYYITTLMT
TLVVYAFLYGKVYLALSGMDEQLQTVADVSGNRALETALNTQFLLQIGIFTAVPMIMNFILEQGVLKAIISFCTMQLQLCSV
FFTFSLGTKTHYFGRTVLHGGAKYRATGRGFVVQHISFGDNYRLYSRSHFIKALEISMLLITYLAYGQDNRGVAYVLLSFSS
WFMALSWLFAPYIFNPAGFEWQKTVEDFEDWTNWLFYKGGIGLKDTESWEAWWEGEQSHIKTLRGRIWEIILSLRFFF
FQYGVVYSLKVSGDSTSLTIYGYSWIALIGLILLFQVFTFTTKSSVKFQLFLRLFQGFLFLSLVGAIVVTIVFTSLSVGDCFATLL
VFIPTGWGILSICQAIRPILEPLHMWDAVRTGFRLYDAMMGAVIFTPVAILSWFPFVSTFQTRLVFNQAFSRGLEISLILAG
NRPNAST----------------------------------------------------------------------------------------------------------

>IRBN 2168378 Scapania nemorosa 1923

ELLVREALRSDGLGARAGRTAARTGRPDELGGVVPPSLGQASNINVILQAAEEIQPDNPQVARILCEYAYSMAHTLDPQS
EGRGVLQFKTGLMSVIRQKQSKREGERIDRTHDIQLIQEFYEIYRAKNQIDELERLQKDANYEEDPGALERRTQTMKKVY
QTSRVLNDVLNALTRDATPEEREKLIQKEWKDRMESDALKTLEFKAYNILPLETPGVADAIMLFEEVKAAAATLEHTSDLP
QFPDDYRRPDQRSLDIFDLLEYIFGFQDGNVNNQRENLVLLLSNAQSMLGLPTDPFKLDDSAINRCFERLLDNYIKWCEY
LRIPSMTTRSDTSQKKLFLMAMYLLIWGEAANLRFLPECLCYIFHQMVDELYGILQFNGDVQRSKTWRPYEGKQYSYLEL
VVAPIYQTVLAEAKNNNGGKASHAAWRNYDDFNEFFWSPRCFVLEWPWRQDAGFLMKPRKKGVAPVEDGKVEPLT
QGRKKEKRVGKMTFVEHRTGIHVFHSFHRLWIFFTVMLQAMMVVAFCDEKLNSRTFKKALSIGPTYLIMKTVQSLFDLL
FIYGAYASTQGWTFSRILVRLLFYGGLGGGVTFLFTKMIQEDSAGPGTTWYHIYLIVLGSYTGAQLFITALFRIPLFRRQADR
CSNWSIIRFIFWLHEDRYFVGRGLYERTRDYFAYTAFWVFILACKFAFSYHFQIKSLVSPTKVIINLPSNQLVYKWHDFVSRR
HHNALTVASLWAPVIMIYYLDTQVWYTVTSALVGGLSGARARLGEIRTLSMLRKRFSTFPQEMARILVPSRVQSLPTMNR
SQSVATGQAGWSKGKVDAFKFAPLWNEIINTLRQEDYISNKERELLVMPSNIDKSTNMVQWPLFLLANKVYMAVEMA
QDNKSINQIQLWERIEKDEYMAYAVAEAYNALEAVLKSLVNDEAKTWVRSVFGDVEGAIQESMLVGHFHLRQVNLVLLR
VQALSGILIHPEDENRRRAAVKAMQDLYETVMRDFLSVELKEKFEGWTLLAQARKDGRLFANLNWPTSQDERDAVKRL
NLLLTIKDSAANIPQNLEARRRLQYFTNSLFMNMPVAPPVRKMLSFSVFTPYYEEDVMYSKQQLVAENEDGITILFYLQKI
FPDEWRNFLERVGYIEKTLQNQLNNESIDPIELRLWASFRGQTLARTVRGMMYYKKALILANLLEAPGTADIEEGLQSFA
GFGATNPGFRKARALAELKFTYVVTCQIYGQQKQKKDLRAADISYLMKTYESLRIAYIDVVEALKDEKNTTTYYSKLIKADA
DGNDQEIYSIKLPGEFRLGEGKPENQNHAIIFTRGDAVQTIDMNQDNYFEEAFKMRNLLEEFKSTDCLRRPTILGIRENVF
TGSVSSLAWFMSQQETSFVTLGQRVLAHPLKVRMHYGHPDVFDRIFHITRGGISKASRTINISEDIFAGFNSTLRQGNITH
HEYIQVGKGRDVGLNQIALFEAKVSSGNGEQILSRDVFRLGQLFDFFRMLSFFYTSVGYYITTLMTALVVYLFLYGKCYLAL
SGLDRQLQTVADISGNAALNSALNTQFLLQIGVFTAVPMIVNFILELGVLKAVISFITMQLQLASVFFTFSLGTKTHYFGRT
VLHGGAKYRATGRGFVVRHISFGENYRLYSRSHFTKALEIAMLLIVYLAYGADNSGITYVLLSFTSWFLAISWLFAPYIFNPS
GFEWQKTVEDFDDWTNWLFYKGGISVKGAESWESWWEEEQEHIRSIRGRLLEIILSLRFFFFQYGVVYSLHVSGGTTSLA
VYGYSWVAFIGLIVLFKVFTFTSHSSVNFQLFLRLFQGVLFLGIVAAIVVCIIFTTLTVGDCFAILLAFIPTGWGLLSIALALRPIL
EPLHLYGIVRSGFRLYDAMMGAVIFAPIAILSWFPFVSTFQTRLVFNQAFSRGLEISLILAGNRPNVNT----------------------
------------------------------

>IRBN 2168395 Scapania nemorosa 1776

YNIIPVHDTLNEHSALQFPEVRAAIAGLQVIAGLPIPPEGWVHGMDILDWLGLIFGFQADNVRNQREHLVLLLANAQMR
LQPPPEPIDQLVYDVVRKLRQKVTKNYLSWCHFLRTKHHIEKLEGRHHEVNEQKELLYTSLYFLIWGEAANLRFMPECLCF
IFHNMAEELNRLVDLSVDDTGQRMHPVSQGRDGFLLNIITPIYDVVKAEADASHGGTFPHSAWRNYDDMNEYFWSKR
CLTQLRWPLEMSCNYLVRPAKEKGKVSKQKVGKTGFVEQRSFWNIFRSFDRLWVGLILLLQLMIIVAFEGKAKRTGQKDK
LPWTVLSRRDAQATALSIFITWAGLRVLQAFLDFGMQYSLVSLGTLRAGLRMTLKFVVACVWVVLFSVYYSRMWDERD
RAGGWTSSALNRLYWYLGFAAVFITPEVLALVLFIIPFVRNAVETADWRIFSVIMWWFQSHIFVGRGLREGIIDNIKYTLF
WIAVLAAKIFFSYFLQVLPLVKPTRSMLAIPNGSIDYKWHEFFKSDNRVAVVATWAPVMLIYFMDIQIWYSLFSALVGALV
GLLEHLGEIRDSTQMKARFRFFPSAVAFNLMPEEALLKQNRPFFNWWGRMKDVVRRVKLRYGVHKTKKEGAKNTEVG
KFKHLWNEIIGIFREEDIVGDDEIELLEMPSTGWNISVTQWPSILLSNELLIALGYCDPQHHWYHSDHWLWRKIASNEYR
RCAVIESYESVKHVLSRIILESSGDRYIFPIIFKEIDDAIHQERFMKTFKLKVLPDVHARLMKLISILLKRPLSKDVPKVVAALQ
DLCEVLIRDFPKDKNTKLKFRELASKSGSLLFIDAVELPDAEEDGRFFYQLKRLATTLSTKVAFNNVPRNLEARRRMSFFSN
SLFMTMPRAPQVDKMLAFSVLTPYYNEDVLYNKKQLSVENEDGVTTLYYLQQIFPDDWEFFEERMLRRGVITEDMIRED
KLQAMWKVDDGLELRLWASYRGQTLARTVRGMMYYQRALTKLAFLDVASEVDIDGYKELLSRTTSGLDLEVANGLDRQ
SSSMAYGRGNGKSFHELEMYDGHNADQDRATASMKFTYVVACQIYGAQKKKGEQQATDILHLMKTYDCLRVAYVDEV

GGEREGKQYYSVLIKYDQVLQQEVEIYRVRLPGELKLGEGKPENQNHALIFTRGDAVQTIDMNQENYFEEALKVRNLLQE
FDRHYGIRKPTILGVREHVFTGAVSSLASFMSAQESSFVTLGQRVLANPLKVRMHYGHPDVFDRLWFMTRGGLSKASRV
INISEDIYAGFNCTLRGGTVTHHEYIQVGKGRDVGLNQISMFEAKVASGNGEQMLSRDIYRLGHRLDFFRMLSVYYTTVG
FFVNNMLIVLTVYAYLWGRVYLAVSGIEDSISSGSKSNSALSAALNQQFLVQIGILTALPMIVENSLERGFLRAIWEFLTMQ
LQLASVFFTFSMGTRAHFFGRTILHGGAKYRATGRGFVVRHEKFSTNYRLYARSHFVKGIEIILLLIVYQAYGSSKNTVVYAL
TTFSCWFLGITWILAPFLFNPSGFDWLKSVDDFDDFMTWIWYKGSVFASAQESWHKWWDEEQEHLNSTGFWGKVM
EIILDLRFFFFQYGIVYRLKIANGSTSIIVYLISWTYIFAAGVIHVVLGVADDRYGAKKHLKYRAVQALVIVLLTLAVVLLFTLTN
FAIWDILTSLLAYLPTGYGLLLICQVLRKPFLENTPVWSMVVAVARLYEIGMGIFVMAPVVILSWLPGFQAMQTRILFNEA
FSRGLNVHKILTGKR------------------------------------------------------------------------------------------------------------
-------------------------------------------------------------------------------------

>JHFI 2114429 Pellia neesiana 1770

PTEVYNIIPVHDTLADHSALRYPEVRAAIAGLQIVGDLRKPQDGWESGMDLLDWLGLTFGFQEDNVRNQREHLVLLLAN
AQMRLQPPPDPIDQLVLDVVRKLRKKITKNYLGWCHFLHIKHNLEKLEGRHHEVNERKELLYTSLYFLIWGEAANLRFMP
ECLCFMFHYMAGELNRILDMSIDETGGLVQPASQGRNGFLRKVVTPIYDVVRAEAEASGGGAVPHSAWRNYDDMNEY
FWSTRCISQLRWPLDKACNYLVKPAKGKGKVSRQKVGKTGFVEQRSFWNIYRSFDRVWIGLILLLQLLIIVAFRGKSKVSG
QSDKLPWTVLSERDSQAHALSIFITWAALRVLQAFLDFGMQYSLISMGTLMVGLRMTLKLIMSSVWVVLFSVYYSRM
WGQRNQDGGWSSAALNHLYVYLGLAGVFISPEVLALLLFIIPWVRNGVETSDWRIFSLMTWWFQSHIFVGRGLREGTF
DNIKYTLFWIVVLAAKIAFSYFLQVLPLVKPTRSTLKISHGSIDYKWHEFFRNANRTATLCMWAPVILIYIMDLQIWYSVFSA
LVGALVGLLAHLGEIRDSQQMKARFQFFPSAVQFNLMPEEGLLKQKLFGSWWGRVKDVVRRVKLRYGVHKVKERDGG
KSIEVGRFKLIWNEIIGIFREEDLISDREQVLLEMPSAGWNITVTQWPSVLLSNEILIALGLCDPDHHWYASDRWLWRKIS
SNEYRRCAVIETYESTKHILMRLIKEDSVDRHIFQGIFREIDDSVRQDRFLKTFKLKALRDIHARLLKLIGILAKRPVEDDIQK
VIDALQGLYEVLVRDFPREQSTRLKFKEATSNPGTLLFVDAVELPDREDHAFFIQLARLEITLSSRVAFLDVPKSLEARRRLSF
FSNSIFMTMPRAPQVEKMLAFSVLTPYYSETVMYNKKDLDTKNEDGVSTLYYLQRIFPDDWDYFVERMARNSVDTEEK
MWKVDDGLELRLWASYRGQTLVRTVRGMMYYHRALQKLSFLDIASEVDIEGYKELLSRTTSGVDVSNGGSGHSSMVSY
TGGSGKRHNEPEMYDGHQQEQDKATAAMKFTYVVACQIYGSQKAKKEQQAADILYLMKTYEALRIAYVDERGDDRQE
KQYFSVLIKYDHELEQEVEIYRVQLPGPLKLGEGKPENQNHALIFTRGDAVQTIDMNQENYFEEALKVRNLLQEFDRKYGI
RKPTILGVREHVFTGAVSSLAWFMSAQETSFVTLGQRVLANPLKVRMHYGHPDVFDRLWFLTRGGMSKASRVINISEDI
YAGFNCTLRGGTVTHHEYIQVGKGRDVGLNQISMFEAKVASGNGEQILSRDIYRLGHRLDFFRMLSVYYSTVGFFVNNM
IVVLTVYAYLWGRVYLAVSGVEESITGSSNSNTALSAALNQQFLVQMGIFTALPMIVESSLERGFLRAIWEFLTMQLQLASV
FFTFSMGTRTHFFGRTVLHGGAKYRATGRGFVVKHENFSTNYRLYARSHFIKGLEIILLLIVYQAYGSSKNTLVYILTTFSCWF
LGITWILAPFLFNPSGFDWLKSVEDFDDFMTWIWYKGGVFVKSEQSWEKWWDEEQEHFLSTGFWGKILEIILDLRFFFF
QYGIVYRLKIANGSTSIIVYLISWTYILAAGVVNLVLGFAGDRYGAKQHLKYRSIQALIIMILMLAIVMLFVFTKLSIWDIFTS
MLAFIPTGWGVLMICQVLRRPFLENTPVWPMVVAVGRLYEMAMGIVVMTPVVILSWLPGFQAMQTRILFNEAFSRGL
QISQIIEGKKP----------------------------------------------------------------------------------------------------------------
-------------------------------------------------------------------------------------

>JHFI 2114436 Pellia neesiana 1918

ANWERLARSALSAHALKGGSRVGETDELGSLVPPSLGQQSNIDIILQAAEEIQPDNAQVARIVCEYAYTMAQDLDPQSA
GRGVLQFKTGLLSVIKQKKSKKEGERIDRSQDIELIQQFYEYYRSKHNIDDLERIHGESSSFEDNPHEYERTTQIMKKVYQT
SRVLNDVLNALTRDATPAEKDQVQQELKMRMETDALKTLDFKAYNILPLETPGVTDLIMSFGEVKAAAASLEYTTDLPEL

PDDLIKPEHRPVDIFDLLEYIFGFQEGNVNNQREHLVLLLSNSQSLLAPPTDSFGLRLDDSAISRCHDRLLDNYIKWCEYLR
MESMTPRNEKGQQKVIFMAMYLLIWGEAANLRFLPECLCYIFHQMVKELRAILDDHVVQHSKTYRTYEGKQYSYLDQV
VTPIYDTILAESKNNKDGKGSHAAWRNYDDFNEYFWSTQCFDLNWPWRLDAGFLTKPKKAKGSSDDETVPLIEPGRKK
EKRVGKMTFVEHRTYLHVYHSFHRLWIFFTIMLQGMMVVAFSNGKLNTRTFKKMLSVGPTYMLMKFIQSLFDLLFIYGA
YASTQGWTFSRMFTRLLYYGGLGGGVTFLYVKMINEASKGTASTWYNLFLIVLGSYIASQVIVTSLLRIPLFRRQAEKRTN
WSIMRFIFWIHQDRYFVGRGMYERTRDYLGYAAFWIFVLACKFAFSYHFQIRSLVSPTKIITKLGRNRLHYKWHDFFSSGH
HNALTVASLWAPVIMIYYLDTQVWYTVVSALVGGLVGARARLGEIRTLSMLRKRFSTFPGEMTRVLVPSRIQTRQIIRTPS
VIDGQAGWEKKKLDAFKFAPLWNEIISTLREEDFISTKEKESLLMPFNEGPSQVVQWPLFLLANKIYLAVEMAQDNKSIN
QEGLWARIEKDEFMKYAIQEAYGSLETVLKALVNDEAETWVRSVFSDLKGGMDEGMLAGHFHLKNVGVVLGRIINLTAIL
MHDDGPKWNRDAVIAMQNLHETVMQDFLTIELREKFESWTLFSRAKNEGRLFSKLNWPKSKEEKEAVRRLNLLLTTKD
SAANVPRNLEARRRLEFFTNSLFMDMPVAPPVRKMLSFSVFTPYYKEDVMYSTDQLVEENEDGISTLFYLQKIFPDEWW
NFLERVGLTGNEMQRQLKNDKFDPIELRLWASFRGQTLARTVRGMMYYKKALVLASLLEGSDTLDVEEGLPSAGSTTEN
NPGFRRARALAELKFTYVVACQLYGEQKQMKDAKATDIAYLMRKYESLRVSYVDIVEGQKDDKKVKTYYSKLIKADIDGK
DQEIYSIKLPGPFKIGEGKPENQNHSIVFTRGDAVQTIDMNQDNYFEEAFKMRNLLEEFKATNNLRRPTILGIREHVFTGS
VSSLAWFMSQQETSFVTLGQRVLAQPLKVRMHYGHPDVFDRIFHITRGGISKASKTINISEDIYAGFNCTLRQGNITHHE
YIQVGKGRDVGLNQIALFEAKVSSGNGEQVLSRDVFRLGQLFDFFRMLSFFYTSVGYYITTLMTSLVIYLFLYGKCYLALSGL
DQQLRVVADVSGNTALSSALNTQFLLQIGVFTAVPMIMNFILEQGVLKAFVSFITMQLQLCSVFFTFSLGTKTHYFGQCVL
HGGAKYRETGRGFVVHHIRFAENYRLFSRSHFIKAFEIAMLLIVYLSYGQDNSGITYILLTFSSWFLALSWLFAPYIFNPSGF
EWQKTVEDFDDWTNWLFYKGGIGVKGEESWEAWWDGEVGHIRTTRSRIWEVILSLRFFFFQYGVAYSLHVSGSTTNIV
IYGYTWIAFVGMIILFKIFTFTNQSSLNYQLFLRLFQGVFFVSLVAGLAIAIAVTTLTVGDCFAMFLAFIPTGWGLLSISIALRP
ILVSIGLWDVVQSGFRLYDAMMGSLIFTPIAILSWFPFVSTFQTRLVFNQAFSRGLEISLILAGNRANTS----------------------
-----------------------------------

>JPYU 2005872 Marchantia polymorpha 1693

GFQKDNVRNQREHLILMLANVHIRLIPKAEPMSKLEDRALNEVMDKLFKNYKSWCKFLGRKNSLWLPTIQQEIHQRKIL
YMGLYLLIWGEAANLRFMPECLCYIYHNMAYELYGMLAGNVSVVTGENIKPAYGGEDESFLKKVITPIYDIIFKEAKNNGN
GTAAHSAWRNYDDLNEFFWSVDCFRLGWPMRLDADFFVAPSQTSLSSKLSIGKGGKVERGKGSLIQTSKTNFVEIRSFW
HLFRSYDRMWTFYILGLQAMIVMAWNIEGSGTFKSTFEGNNFKRIHLLSIFITAAILRVIQGLLDIAMSMKAYRSIKFMG
MLRLFLKLLVSIAWVIVLSVCFVHTWENPTGLIKNVQNVLGSSWKNPSLYITAVAIYLLPNALAAVLFVFPLMRRWIENSN
WRIVRLLLWWSQPRLYIGRGMHESQWTLFKYTMFWILLLASKFVFSYFIQIKPLVKPTKTIMNDSSIQFTWHELFPNVKK
NIGAIIAIWAPVILVYFMDTQIWYSVYSTVFGGVSGAFRRLGEIRTLGMLRSRFRSLPGAFNANLVPADKVARRGFSLARG
YKEVQPGKDRKEAAKFAQLWNEVITSFREEDLISNKEMDLMLVPYSSVNLTLVQWPPFLLASKIPVALQMAVEHRGRDM
DLWRKIRADDYMRCAVEECFESFKHVLGTILVGEVERRLVIDGILEEIDKDISEGSLLSNFKMSALPVLHSKFVQLTEFLIKGE
ADKRDSVVLLLQDMFEVVTRDMMNETAREYLESTHGPLSISGKTSKDVKDHQLFAATDPKPAVLFPPPATDAWIEQIKRL
HRLLTVKESAINVPTNLEARRRIAFFTNSLFMDMPRAPRVRNMLSFSVLTPYYQEEVVYSKKQLNEENEDGISVLFYLQKIY
PDEFDNFLERINVTSEHEIWDNEEYENELRHWASYRGQTLSRTVRGMMYYRRALELQAFLDMASDDELVDGYKVVASA
PAEAKKSQRSMWAQLQAIADMKFTYVATCQIYGAQKRAADVRATDILNLMLNNPSLRVAYIDEVEGREKDKNQKVYYS
VLVKASNGLDQEIYRIKLPGMVKLGEGKPENQNHAMIFTRGEALQTIDMNQDNYLEEAFKMRNLLEEFHEPHGVRPPTI
LGVREHIFTGSVSSLAWFMSNQETSFVTIGQRVLASPLKVRFHYGHPDVFDRLFHITRGGMSKASRVINLSEDIFAGFNST
LRRGNVTHHEYIQVGKGRDVGLNQISLFEAKVACGNGEQTLSRDMYRLGHRFDFFRMMSCYFTTIGFYASTVIVVLTVY
VFLYGRIYLALSGIEKSLVNSADVNNDPALQAALASQSLVQLGLLMALPMVMEIGLERGFRTALSDFIIMQLQLASVFFTFS
LGTKTHYYGRTILHGGAKYRATGRGFVVRHEKFAENYRLYSRSHFVKGIELMMLLIIYSVYGTSAKGGVPYLLITFSMWFLV
TTWLFAPFLFNPSGFEWQKIVEDWDDWSKWINNRGGIGVLATKSWESWWEEEQEHLKHTGLMGRVLEVLLSIRFFLY

QYGMVYTLSIAGGSTSLSVYGLSWLVIIAVLAVLKIVSMGRRRFSADFQLMFRLLKALLFIGFVTIVIVLFLFAQLSVGDLFAS
LLAFLPTGWGLLMIAQAARPLVVRSGMGDSVKALARAYEFIMGLCIFTPVAMLAWFPFVSEFQTRLLFNQAFSRGLQISR
ILAGRRK------------------------------------------------------------------------------------------------------------------------------
-------------------------------------------------------------------------------------------------------------------------------------------
-------------------

>JPYU 2009036 Marchantia polymorpha 1701

RKKITKNYIRWCSFLRIKHNLEKIEDHGIRRRHESNEQKELLYVSLYLLIWGEAANLRFMPECLCFIFHHMCGELNRILDRSI
DDSGQYMQPASGGIENGFLQKVIRPIYAVVKAEAAECRAGKNPHSAWRNYDDMNEYFWSKRCLTQLRWPLEMSCNY
LVKPEDKSRHKVGKTGFVEQRSFWNIYRSFDRLWIGLILMLQILIIVAFRAKAELPQDTLAPSTQETQSGAAKAPWTILAQ
RDSQAHVLSIFITWSALRVLQAFLDFGMQGMQYSLISAGTVMVGLRMTLKLIMGAVWTVLFSIYYSRMWAQRNADGL
WSDKALQGLYLYLGFCAVFITPEALALLLFILPYIRNFVETRDWRIFSMLTWWFQSHIFVGRGLRESIFDNIKYTLFWLCVLT
AKFSFSYFLQVLPLVAPTRATLDVTGIEYRWHEFFKNANRTATLAMWAPVVLIYFMDLQIWYSVFSALVGALVGLLAHLGE
IRNAAQLKARFHIFPVAVQFNLMPEDIFVNSKPYSWRAKCNDWWVKVKNLARRVKLRYGVVKGHQAEGKTMETGRFR
HVWNEIILIFREEDLISDRELELLSMPSSRWNITVTQWPSVLISNEVLIALGLCKDWYYTDHGLWKKIASNEYRRCAVVESY
ESIRHVVKRILREDSGEYQMFQEIFEEITKAIKEKQFVQRFSLKALPNIHSRLVQLINVLMKRPLLNDLQKVVDALQNLYEDL
LRDFPKDQELARSWREMSSTSSLLFVDAVDLPDVTENTFFDQLRRVQTTLSTREALLDVPRNLEAKRRISFFSNSLFMTM
PRAPQVEKMLAFSVLTPYYSEDVMYHKKQLITENEDGVSILFYLQKIFKDDWENFQERMARQGIRTEFDMWELDDGLE
LRLWASYRGQTLARTVRGMMYYHRALEKLAFLDHASEHDILEGYRELMERTSGGVDPFNGETREGSFNGETREGSFNG
ETREGSLHSASSFTEGKEGRNYNAYKREEDLATAAMKYTYVVACQIYGIQKAKGQQQAKDILYLMKTWEALRVAYVDEK
PGLGDKDPKRYASVLIKYDQVRQEEVEIYRVELPGDFKLGEGKPENQNHALIFTRGDAVQTIDMNQENYFEEALKMRNL
LQEFKQFHGRRRPAILGVREHVFTGAVSSLAWFMSAQETSFVTLGQRVLAKPLKVRMHYGHPDVFDRLWFITRGGLSK
ASRVINISEDIFAGFNATLRGATVTHHEYIQVGKGRDVGLNQIALFEAKVASGNGEQMLSRDVYRLGHRVDFFRMLSVYY
TTVGFFVNNMIVVLTVYAYLWGRVYLALSGIEVSITRDASANGALTAALNQQFVVQMGVFTALPMIVENSLEKGFLLAIW
EFLTMQLQLASVFFTFSMGTRTHYFGRTILHGGAKYRATGRGFVVRHEKFSDNYRLYARSHFVKGLEIIILLIVYQMYGSIR
NTTTYILTTFSCWFLGITWILAPFLFNPSGFDWLKSVDDFDDFMTWIWYKGGVFVKGEDSWQKWWDEEQEHFQSTGF
WGKCLEIILSLRFFFFQYGIVYRLKISSGSTSIVVYLISWTYLFAAGLIHLILSRAAEKYGAKRHRKYRGIQAFIICFLVLAVVLLF
VFTDFSIWDIFTSILAFVPTGWGVLSICLVLRRPFLENTPVWPMVTGVARLYEMGMGICVMTPVVVLSWLPGLQAMQT
RILFSEAFSRGLSIQRLLTGRK-----------------------------------------------------------------------------------------------------
-------------------------------------------------------------------------------------------------------------------------------------------
---------------------------------

>JPYU 2009972 Marchantia polymorpha 1595

KWCQYLRITAVTESAAGDSHKMVLLTAMYLLIWGEAANLRFLPECLCYIFHHMVKELYGLLGNTNAQRSMNYVEGSDCP
FLDKIVTPLYSVIAAEAANTQDGKASHAAWRNYDDFNEYFWSPRCLESLSWPWRPDAGFMMKPNTKKMKGVVNAVP
GRKKEKKVGKMTFVEHRTGFHVYHSFHRLWIFFTVMLQGMMIFAFCDEKFTPNTFKKMLSVGPTFVIMKLIQSLLDIFFI
YGAYASTQGWTFSRIFARLAWFGGLSGSITWLFVKMIQEENTGTGSTWYHLYLIVLGSYTAAQVVVTLLMRIPFFRRQAD
KCGDWAIIRFIMWVHQDRYFVGRGLYESYVGYSAFWIFVLACKFSFSYYFQVRALVSPTKTIVLQRFEYKWHDFVSRNNH
NALTIGALWAPVILIYYLDTQVWYTVVSAIVGGLDGARARLGEIRTLSMLRKRFSSFPEAFATNLVSSRLGRGEKNPGSSPA
QVNWSMQKVQAVKFAPMWNEIINCLREEDYIGKKERELLVMPKNQGSGTLVQWPLFLLANKVYLAVEMAQDAKLLN
QTQLWEKICKDEYMAYAIEEAYKLFEEVLKSLVREDVSQIWVKGVFNDVGAGIQESALVGHFYLKKVDSVLTRVTDLTHLL

EREETDKLKSQVVLAMQNLYESVMNDFFTAELRERIEEFRALKDARIENKLFINLTWPTTDREKENVKRLHLLLTIKESAAN
IPRNLEARRRMEFFANSLFMDMPKPPSVQRMLSFSVFTPYYAEDVLYSKDKLTEENEDGISILFYLQKIFPDEWKNFLERQ
GLTNALMERQMEDKKNECQELRLWASYRGQTLARTVRGMMYYKKALILQSLLEGTSDVEEGGVLGSTLEESPGYRMAS
AIAETKFTYVVTCQIYGQQKEKRAQQATDILYLMNKYDSLRIAYVDIVDKMQEDKETKEYKSIKEYYSKLIKADADGKDQEI
YSIKLPGQFKLGEGKPENQNQAIVFTRGDACQTIDMNQDNYFEEAFKMRNLLEEFNSTDSLRRPTILGIREHVFTGSVSSL
AWFMSQQETSFVTLGQRVLARPLKVRMHYGHPDVFDRIFHITRGGISKASRTINISEDIYAGFNSTLRQGNITHHEYIQV
GKGRDVGLNQIAMFEAKVSSGNGEQILSRDVFRLGQLFDFFRMFSFFYTSVGYYITTLMTTLVVYAFLYGKVYLALSGMD
RQLQDFADINNNKALESALNTQFLLQIGVFTAIPMIMNFILEQGVLKAVISFLTMQLQLCSVFFTFSLGTKTHFFGRTVLHG
GAKYRATGRGFVVQHISFADNYRLYSRSHFNKALEISMLLITYLSYGEDNRGVSYILLSFSSWFMALSWLFAPYIFNPAGFE
WQKTVEDFDDWTNWLFYKGGIGVKDNESWESWWESEQEHIRTLRGRFWEIVLSLRFFFFQYGVVYSLQVSGTSTSLSI
YGYSWLAFFVLIVLFQIFTFTNKSSVKFQLFLRLFQGGLFLALIASVAVTIAFTSLSVGDCFATLLVFIPTGWGVLSICQALRPIL
EPLGAWNAVRSGFRLYDAMMGAVIFTPIAILSWFPFVSTFQTRLVFNQAFSRGLEISLILAGNRPNAST--------------------
--------------------------------------------------------------------------------------------------------------------------------------------
--------------------------------------------------------------------------------------------------------------------------------------------
---------------------------------------------------------------------------------------

>KRUQ 2016796 Porella navicularis 1773

PDDVYNIIPVHHTLSEHAAMQFGEVRAAIAALDVVGDLRPPERWMPHMDMLDWLGLTFGFQDDNVKNQREHLVLLL
ANAQMRLQPPPDPIDKLVLDVVRKLRKKITKNYLSWCHFLHIGHNLEKLEGRHHEVNEQKELMYTSLYFLIWGEAANLR
FMPECLCFIFHNMAGELKRILALSVDDNGQLIRAASEGRGGFLMKVVTPIYNVVKAEADANGGGTVPHSAWRNYDDM
NEYFWSKRCLTQLRWPLEMSCNYLVQPAKGKGKVSRQKVGKTGFVEQRSFWNVYRSFDRVWIGLILLLQLLITVAFRGK
ARTTGASDKLPWTVLSERDSQAHALTIFITWAALRVLQAFLDFGMQYSLISLGTLKVGARMTLKLIVACVWVVLFAVYYS
RMWAQRNSDGAWTTAALQRLYVYLGFALAFICPEVLALVLFITPWVRNAVETSNWRIFAPLTWWFQSHVFVGRGLREG
FFDNLKYSLFWILVLLAKISFSYFLQILPLVKPTRSTLRIPDGEINYKWHEFFKNANRTALLCMWAPVILIYIMDLQIWYSLFS
AVVGALIGLLAHLGEIRNATQMQARFRMFPSAVYFNLMPEEGLLRHRPFYNWWGRVKDVVRRLKLRYGVLKVKEREG
KNAEIGKFKHIWNEIIGIFREEDLICDEELELLVMPTTGWNVSVTQWPSILLSNEVRIALRLCDPEHRHWYHNDRWLWRK
IASNEYRRCAVIESYESLKHVLKRIIRNTSDDHHIFPVIFKEIDDSIHQGRFMKTFKLKELPEVHERVVKLIAILMKRPGLDDI
QKVIDALQGLYEVLIRDFPKDQNTKNKVMELASTSLKFVDAVELPDAEDAVQFFNQLTRLHTTINTRLAFLNVPKNLEAR
RRISFFSNSIFMTMPRAPQVDKMLAFSVLTPYYREDVMYNKKQLDTENEDGVKTLFYLQKIFPDDWRFFKQRMARKYEL
FIQDDERFIDHLWEEDDGLELRLWASYRGQTLVRTVRGMMYYHRALKKLAFLDGASENDIEGYRELLSRTSSGLELALTG
SRHSSFGYIGSGESFHEPDMYDGHREEQDRATAAMKFTYVVACQIYGAQKAKREQQAADILYLMKTFEPLRVAYFDVTG
GERDVKKYYSVLIKYDPVLEQEVEIYRVQLPGPLILGEGKPENQNHALIFTRGDAVQTIDMNQENYFEEALKMRNLLQEF
DKYHGIRKPTILGVREHVFTGAVSSLASFMSAQETSFVTLGQRVLANPLKVRMHYGHPDVFDRLWFLTRGGMSKASKVI
NISEDIYAGFNCTLRGGTVTHHEYIQVGKGRDVGLNQISMFEAKVASGNGEQMLSRDIYRLGHRLDFFRMLSVYYTTVG
FFVNNMIVVLTVYAYLWGRIYLAVSGIEESITGSSNSNTALSAALNQQFLVQMGILTALPMIVENSLEKGFLSAIWEFLTMQ
LQLASVFFTFSMGTRAHFFGRTILHGGAAYKATGRGFVVKHEDFTTNYRLYARSHFVKGLEIILLLIIYQAYGSSKNTVVYILT
TFSCWFLGITWILAPFLFNPSGFDWLKSVEDFDDFMTWIWYKGGVFVKDTESWQKWWDDEQQHFQSTGFWGKCLEI
VLDLRFFFFQYGIVYRLKIANGSTSIIVYLISWSYIFAAGLIHLVLGLAGDRYGAKQHIKYRSIQTLVLVLLALVVVMLFVFTEFS
IWDIFTSMLAFIPTGWGLLSICLVLRKPFLENTPVWPMVVAVARLYEMAMGILVMAPVVFLSWLPGFQAMQTRILFNEA
FSRGLEISKIISGKKP---------------------------------------------------------------------------------------------------------------------
---------------------------------------------------------------------------------------------

>KRUQ 2017364 Porella navicularis 1927

RAVGNWEKLVGSALATGELRGSSRPERDDDLGGGTVPPSLGQNDGQESNINDILQAAEEIQPHDAQVARILCEYAYNM
AQNLDPQSEGRGVLQFKTGLLSVIRQKQSKREGERIDRSHDIQHIQRFYELYRTTNRIEELERRQKETGVLHEDPVEHERR
TQRLKKVYQTSRVLNIVLNALTRDSTPEERDNWIQKELKDTMEMDAKKTADFKAYNILPLETPGVADAIMSFEEVKAAAA
SLAHTDELPQFPGDYRKPVSRDVDIFDLLEYIFGFQEGNVNNQREHLILLLSNSQSLLSVPTEGLMLDESAITRCFERLLDN
YIKWCNYLRIDPMTDRAVTGQRKLILMGMYLLIWGEAANLRFLPECLCYIFHQMVTELYGILEGRDVQRSKTYKTYEKDG
KQYSYLDQVVTPIYQTVLAEAKNNKGGKASHAAWRNYDDFNEFFWSSRCFVLQWPWRSDAGFFMKPRKKGVAIEGG
KGAGLVPGQRREKRVGKMTFVEHRTYFHIFHSFHRLWIFFTVMLQAMMVVAFCDEKLNTRTFKKMLSVGPTYLIMKTV
QSLFDLTFIYGAYGSTQGWTFSRIFTRLLFYGGLAAGILFLYIKMIQEDSQGAATTWYNLFLIVLGSYAGAQLLATAVLRIPFF
RRQTDKCSNWGIIRFIYWVHQDRYFVGRGLYERSRDYLGYTAFWVFVLACKFAFSYHFQIRSLVSPTKTIVNLSSNALQYK
WHDFFSAKNHNALTVASIWAPVIMIYYLDTQVWYTVTSALVGGLSGARARLGEIRALSMLRKRFSTFPEEMARNLFPSR
VQTQQLNRVQSSLRGQAGWPKAKVDAFKFSPIWNAIIISLREEDYINNREKELLLMPSNHGASNMVQWPLFLLANKVY
MAVEMAQDNKSTNQIGLWERIEKDEYMAYAVKEAYNTLETVLKALVNNEAKTWVRSVFGDVEGGIQESMLVGHFHLR
QVDQVLSRIQVLTALLIHDEDKDGKKKAVIAMQDLYETVLHDFLSADLREKFQGWTLLSQAKNEGRLFANLNWPQTIEE
KEAVKRLHNLLTIKESSANIPRNLEARRRLQFFTNSLFMHMPVAPPVRQMLSFSVFTPYYEEDVMYSKEQVKVENEDGISI
LFYLQKIFPDEWKNFQERIGLTNAVIDAQLKNISLDMLELRLWASFRGQTLARTVRGMMYYKKALVLASLLEEPTADIEEG
LQSFAGTGDSNPGFRRARALAELKFTYVLTCQIYGVQKQKRDVRAADISYLMKKYESLRIAYIDMVESMKDEKSSMQYYS
KLIKADVDGNDQEIYSIKLPGEFRLGEGKPENQNHAIVFTRGDAVQTIDMNQDMSFEEAFKMRNLLEEFKAENCLRRPTI
LGIRENVFTGSVSSLAWFMSQQETSFVTLGQRVLAHPLKIRMHYGHPDVFDRIFHITRGGISKASKTINISEDIYAGFNSTL
RQGNVTHHEYIQVGKGRDVGLNQIAIFEAKVSGGNGEQILSRDVFRLGQLLDFFRMLSFFYTSVGYYITTLMTSLVIYLFLY
GKCYLALSGLDRQLQIVADISGNAALSSALNTQFLLQIGVFTAVPMIVNFILEQGFLKAVTSFITMQLQLCSVFFTFSLGTKT
HYFGRTVLHGGAKYSATGRGFVVRHIPFADNYRLYSRSHFTKALEIAMLLIVYLAYGEDNSGLTYILLSFSSWFLALSWLFAP
YIFNPSGFEWQKTVEDFDDWTNWLFYKGGISVKGAESWESWLEEEQVHIKTWRGRIWEIVLSLRFFFFQYGVVYSLSVS
GSSTSLAVYGYSWIVFVGIVILFKIFTFTSQASVNFQLFLRLFQGVLMLGIITAVVVVIVKTSLTVGDCFALLLAFIPTGWGLLS
IAVALKKYLELMHIWGFLLSSFRLYDAMMGAVIFTPIAILSWLPFVSTFQTRLVFNQAFSRGLEISLILAGNRPNAST---------
----------------------------------------

>KRUQ 2019279 Porella navicularis 1919

RGLSRTYTTANFSDVFDSEVVPSSLSSIAAILRVANEIESQRPRVAYLCRFYAFEKAHKLDPTSSGRGVRQFKTALLQRLERD
NIPTLSARHRRSDAREMQSFYQQYYDTYVKALDGAEHSDRAQLAKAYQTAGILFEVLTAVNKSEQAEPPPEIIAASKIVEEK
QEILQPFNILPLDAAGASQAIMQLPEIKASVESLRNIRGLPWPPGWEQGRHKSGELDLLDWLQSMFGFQKANVANQRE
HLILLLANVHIRLIPKPEPMLKLDDRALNQVMDKLFKNYKSWCKFLGRKNSLWLPTIPQEIQQRKVLYMGLYLLIWGEAT
NLRFMPECLCYIYHNMAYELFGMLAGNVSLATGENIKPAYGGDEESFLRKVITPIYEIVEKEARSNCNGTAPHSAWRNYD
DLNEFFWSVDCFLLGWPMRLDSDFFVPPAIYNRKVPNSDVKKTGGPAPKYLGKSNFVEIRSFGHLFRSYDRLWTFYILGL
QALIVIAWSIEPNGSLRSMFEGDNFKKILSIFITAAILRCVQGVLDFALSIHAYRSMKFLGLLRLVLKLCVSAAWVIVLAVCYA
HTWSNPTGLVRSIQRLLGSSWKSPSLYITAVVIYLVPNILGAILFVFPMLRRWIENSNWRIVRFLLWWSQPRLYVGRGMH
ESQWTLFKYSVFWILLLGSKFVFSYYIQIKPLVRPTKTIMNARDIQFTWHELFPNVRKNIGAVISIWAPVVLVYFMDTQIW
YSVYSTLFGGISGAFRRLGEIRTLGMLRSRFRSLPGAFNANLVPAEKVAKRGFSLARGYKEVQPGKDRKEASKFAQLWNE
VIMSFREEDLISNKERDLMLVPYSSSVNLTLVQWPPFLLASKIPIALQMAVEHRGNDADLWRKIRADDYRRCAVEECFESF
KNVLGIILVGELEKRVVIDGILAEIEKDIAEGNLLANFKMSALPVLHSKFVELTEYLIKGEESSRKFVVKRLQDMFEVVTRDM
MNETAREYLESTLGPLSISGKTSKDVKDHQLFAATEPKPALLFPPPPTDAWLEQIRRLHRLLTVKETAVNVPNNLEARRRIA
FFTNSLFMDMPRAPRVRNMYSFSVLTPYYHEEVLFSKGQLNEENEDGVSVLFYLQKIYPDEWENFLERMKLQDERQFW

NNDEYVDELRHWASYRGQTLSKTVRGMMYYRRALELQAFLDMASDDELVEGYKVVASAPADTKKSQRSMWAQLQAI
ADMKFTYVATCQSYGAQKRAGDVRATDILNLMLNNPSLRVAYIDECEDEEKDKDQKVYYSVLVKAANGLDEEIYRIKLPG
PVRLGEGKPENQNHAMIFTRGEGLQTIDMNQDNYLEEAFKMRNLLEEFHEPHGVRAPTILGVREHIFTGSVSSLAWFM
SNQETTFVTIGQRVLASPLKVRFHYGHPDVFDRLFHITRGGVSKASRIINLSEDIFAGFNSTLRRGNITHHEYIQVGKGRDV
GLNQISLFEAKVACGNGEQTLSRDMYRLGHRFDFFRMMSCYFTTVGYYFSTMTVVLTVYVFLYGRIYLALSGIEKSLVNSA
DVKNDTALQAALASQSLVQLGLLMALPMVMEIGLERGFRTALSDFIIMQLQLASVFFTFSVGTKTHYYGRTLLHGGAKYR
ATGRGFVVRHEKFAENYRLYSRSHFVKAIELLVLLIIYSVYGTSVKANVPYLLITFSMWFLVATWLFAPFLFNPSGFEWQKIV
EDWDDWSKWINNRGGIGVQADKSWESWWSEEQEHLENTGLRGRLIEMLLSVRFLLYQYGLIYTLNISKGSKGLGVYGL
SWLVIIAVLAVLKIVSMGRKRFSADFQLMFRLLKALLFMGFVAVLVVLFVFAHLAVSDIFASLLAFLPTGWALLMIAQAARP
IVRPTGMWDSVKALARAYDFIMGLVIFTPVAVLAWFPFVSEFQTRLLFNQAFSRGLQISRILAGRKK-----------------------
---------------------------------

>LGOW 2021351 Schistochila sp 1773

PDDIYNIIPVHDTLSEHSALRFPEVRAAIAALKVVGDLRVPPQGWMPGMDMLDWLGLTFGFQVDNVKNQREHLVLLL
ANAQMRLQPPPDPIDKLVFDVVRKLRRKITKNYLSWCHFLHIKHRIEKLEGRHHEVSEQKELMYTSLYFLIWGEAANLRF
MPECLCFLFHNMAGELNRILDSSVDDTGQHMQPFSQGRNGFLINVVTPIYNVVKAEADASGGGSFPHSAWRNYDDM
NEYFWSKRCLTQLSWPLDKSCNYLVPPAKGKGKVSRQKVGKTGFVEQRSFWNIYRSFDRVWIGLILLLQVLIIIAFKAKAK
RTGESDKAPWSVLSERDSQAHALTIFITWAGLRVLLAFLDFGMQYSLISRGTLRVGARMTLKLLVASTWVVLFSVYYSRM
WAKRNNDGAWTHAALERLYVYLGLAAVYILPEVLALVLFILPWIRNAVETTDWRIFSILMWWFQSHIFVGRGLREGLFD
NIKYTLFWILVLAAKIAFSYFLQILPLVKPTRTTLDIREGSIHYRWHEFFKGANHVALLCMWAPVVLIYIMDLQIWYSFFSAL
VGAFVGLLSHLGEIRNSAQMKARFRFFPTAVQFNLMPEEGLLKQQRFYNWWGRVKDVVRRVKLRYGVQRINEKEGGK
AVEVGRFRHVWNEIIGIFREEDLLSDEEVELLEMPSAGWNISVTQWPSILLSNEVLIALGFCDPEHHWYHSDHWLWRKI
ASNEYRRCAVIESYESLKHVLSRIIRVASDDSHIFQVIFKEIDDAIHQERFMKTFKLKVLPEVHARLMKLITILMKRPLLNDIQ
KVIDALQDLFEVLVRDFPKDKSTKTNFRELASKSGSLLFVDAVELPDAEEDGRFFYQLKRLGTTMSTKVAFLNVPKNLEAR
RRISFFSNSLFMTMPRAPQVDKMLAFSVLTPYYSEDVMYNKKQLTTENEDGFTTLFYLQKIFPDDWEFFQERMARRGIS
DDAQMWKVDDGLEIRLWASYRGQTLVRTVRGMMYYHRALQKLAFLDVASEVDIDGYKELLSRTSSGLELATTNGVSRH
SSLGRGNSGKSFHELEMYDGHHAEQDKATASMKFTYVVACQIYGAQKKKAEQQARDILHLMKTFEALRVAYVDETGAD
RDEKQYYSVLIKYDPVLEQEVEIYRVQLPGPLKLGEGKPENQNHALIFTRGDAVQTIDMNQENYFEEALKVRNLLQEFDR
SHGIRKPTILGVREHVFTGAVSSLASFMSAQETSFVTLGQRVLANPLKVRMHYGHPDVFDRLWFLTRGGLSKASRVINISE
DIFAGFNCTLRGGSVSHHEYIQVGKGRDVGLNQISMFEAKVASGNGEQMLSRDIYRLGHRLDFFRMLSVYYTTVGFFVN
NMIVVLTVYAFLWGRAYLSLSGIEDSISDSTNSNTALSAALNQQFLVQMGILTALPMIVENSLEKGFLVAIWEFLKMQLQL
ASVFFTFSMGTRAHFFGRTILHGGAKYRATGRGFVVRHESFSANYRLYARSHFVKGLEIILLLIVYQAYGSSKSSVVYVLTTV
SCWFLGITWILAPFVFNPSGFDWLKSVDDFDEFMTWIWYKGGVFVQATQSWAKWWDEEQEHFLSTGFWGKCLEIILD
LRFFFFQYGIVYRLKIANGSTSIIVYLISWIYIFAAGVINLVLGFADDRYGAKQHIKYRSIQALVIVLLVLAIVMLFVFTNFVIWD
IFTSFLAFIPTGWGLLLICQVLRKPFLENTPVWSMVVAVARLYEMGMGIVVMAPVVFLSWLPGFQAMQTRILFNEAFSR
GLHFYQIVAGRKSTS------------------------------------------------------------------------------------------------------------
--------------------------------------------------------------------------------------------

>LGOW 2105553 Schistochila sp 1915

IDKWEHLVRQALRSEELRRSRIGRSDDLGGGVVPPSLGQASNINIILQAAEEIQPDDPQVARILCEYAYSMAHTLDPQSEG
RGVLQFKTGLMSVIRQKQSKREGERIDRTHDIQLIQEFYEFYRSKHQIDELERKQKEDPNFEEGPGALEQRTRRMKKVYQ

TSRVLNDVLNALTRDATPEEREKLIQKELKDRMETDALKTLDFKAYNILPLATPGVADAIMVFEEVKAAAASMEHTSDLP
QFPEDYRRPEQRAVDIFDLLEYIFGFQNGNVNNQREHLVLLLSNSQSMLGLPTDSLKLDETAVNHCFDRLLDNYIKWCDY
LRIVSMTSRATTIQRKLLMMAMYSLIWGEAANLRFLPECLCYIFHQMTEELYSILGVPDIQRSKTYAASAEKDGKQYSFLD
QIVTPIYQTLLAESKNNNGGKASHAAWRNYDDFNEFFWSPRCFVLQWPWRPDAGFLMKPKKKGVVPEEGKGAPLEP
GRKKEKRVGKMTFVEHRTALHVYHSFHRLWIFFTVMLQALMVVAFCHEKLDTRTFKKLLSVGPTYLILKTVQSLFDLLFIY
GAYASTQGWTFSRILTRLLFYGGLGGGVTFLYVKVSQEESAGNASTWYHLYLIVLGSYVGAQLLVTAVLRIPFSRRQADKCS
NWGIIRFVFWLHQDRYFVGRGLYERNRDYFGYSAFWVVVLACKFAFSYHFQIKSLVSPTQIIIDLSSNELQYRWHDFISKG
HHNALTVASIWAPVIMIYYLDPQVWYTVTSALVGGLSGARARLGEIRTLGMLRKRFSTFPDEMARILVPSRIQTQQLNRS
QSSAVGQAGWSKAKVDAFKFSPLWNEVINCLREEDYINNREKELLLMPSNTGKSRLVQWPLFLLANKVYMAVEMAQD
NKSINQIQLWERIEKDEYMAYAVAEAYNALESVLKSLVNDEAKTWVRSVFGDVEGGIQESMLVGHFHLRQVNLVLMRV
QALSGILVLSEDENRYRSAVKAMQDLYETVMKDFLSVELREKFESWTLLAQAKNDGRLFANLNWPTSPEQKEAVKRLNL
LLTIKESSANIPQNLEARRRLQYFTNSLFMHMPTAPPVRKMLSFSVFTPYYEEDVMYSKEQLVSKNEDGISILFYLQKIFPD
EWRNFLERVGLVEAEMHRQLRNEQLDLIDLRLWASFRGQTLSRTVRGMMYYKKAIILANLLEAPATSDIEAGLQSFGGA
GETNPGFRKARALAELKFTYVVTCQIYGMQKQKKDNRAADISYLMRKYESLRIAYVDVVESLREEKTTTTYFSKLIKADAD
GNDQEIYSIKLPGEFRLGEGKPENQNHAIIFTRGDAVQTIDMNQDNYFEEAFKMRNLLEEFKDLNCLRRPTILGIREHVFT
GSVSSLGLFMSQQETSFVTLGQRVLAHPLKVRMHYGHPDVFDRIFHITRGGISKASKTINISEDIYAGFNSTLRQGNITHH
EYIQVGKGRDVGLNQIALFEAKVSSGNGEQILSRDVFRLGQLFDFFRMLSFFYTSVGYYITTLMTTLVVYLFLYGKCYLALS
GLDRQLQLVADTEGNAALSSALNTQFLLQIGVFTAVPMIMNFILEQGVLKAAVSFATMQFQLCSVFFTFSLGTKTHYFGR
TILHGGAKYRATGRGFVVRHISFGENYRLYSRSHFTKALEIAMLLIVYLAYGEDNHGITYVLLSFTSWFLALSWLFAPYIFNP
SGFEWQKTVEDFEDWTNWLFYKGGISVKGAESWESWWEEEQEHIRSLRGKLWEIILSLRFFFFQYGVVYSLHVSGSSTS
LAVYGYSWLAFIGLIVLFRVFTFTSQSSVNFQLFLRLFQGVLFLALVAVVVVCIVVTTLTVGDCFALLLAFIPTGWGLLSIALA
LRKVLEPLHMWGIVRSGFRLYDTMMGAVIFIPIAFLSWFPFVSTFQTRLVFNQAFSRGLEISLILAG-------------------------
-----------------------------------

>NRWZ 2101168 Metzgeria crassipilis 1923

KRSVGNWERLVKGALSSAELRTRRRRRGDDLGGGVVPPSLGQQSDIDIILRAAEEIQPDDPQVARILCEYAYSMAHNLDP
QSEGRGVLQFKTGLMSVIRQKQSKRDGDKIDRSQDIQRVQEFYEYYRRKNQIDELEQRHRQGTSSYEEDPGELELRTQKL
KKVYQISGVLNDVLDALTRDYSQEEKGRILQKDLKDRMETDALKNTDFKSYNILPLEAPGVADPIMSFDEVQAATVSLEHT
RDMPRFPDDYHRPEQRAVDIFDLLEFTFGFQEGNVSNQREHLVLLLANSQSMLGLPTVRGLLDENAINRCYDRLLDNYV
KWCEYLRIEPVTSKANTGQRRLTLMAMYLLIWGEAANLRFLPECLCYIFHQMVLELNGLMDGRDAQRARTYSLESEKD
GKQYSFLEQVVSPIYQTVLAESKNNGGGKASHSAWRNYDDFNEYFWSLACFGLQWPWRLEAGYLVKPKKKGVVPDDG
KLTLAPAEAVRKREKRIGKLTFVEHRIGFHAYHSFHRLWIFLTVMLQAMMVVAFSDGELNNRSFKKLLSVGPTYLIMKFVQ
ALFDLLFIYGAYASTQGWTFSRIFTRLLYYGGLGAGITFLYVKMMREGSATAWYHLFLIVLGSYAGAQILVTILLRVPFFRRQ
ADKCSNWGIMRFIFWVHQDRYFVGRGLYERTRDYTAYTLFWIVVLACKFAFSYHFQIASLVSPTQVIANLNRSELVYRWH
DFASAGHHNALTIASIWAPVIMIYYLDIQVWYTVTSALVGGLLGATGRLGEIRTLSMLRKRFSTFPQENARILVPSRLQNQL
FKSQSIAREKSLWSKAKTDAIKFAPMWNEIIKYLREEDYINNKEKELLLMPSNTGSSQIVEYPLFLLANKVYMAVEMAQD
NKSTNQTQLWDRIVKDEYMSYAVQEAYDILYNVLKSLVKDEARTWVLTVFGDVKDGIQESMLVGHYHLKKVDIVLQRVT
VLTGILICDGDEKHRAMAVKAMQDLYEAVMHDFLSVELREKFESWQQLTLAKVDGRLFANLNWPSSDREKAAVRRLNL
LLTIKESSANIPKNLEARRRLQYFTNSLFMHMPAAPSVRKMLSFSVFTPYYAEDVMYSEEKLTTRNEDGITTLRYLQTISPD
EWWNFKERVGLTENELKRQLDNESIDVIELRLWASYRGQTLARTVRGMMYYKKALVLASLLEGSGMEDVEEGFHSIEGA
VESNAGFRKARALAELKFTYVVTCQIYGEQKLKKDVRAADISYLMRKYESLRVAYIDVVESLKGEKTLSTYYSKLVKADTDG
NDQEIYSIKLPGEFRIGEGKPENQNHAIIFTRGDAVQTIDMNQDNYFEEAFKMRNLLEEFKATNCLRRPTILGIREHVFTG
SVSSLAWFMSQQETSFVTLGQRVLARPLKVRMHYGHPDVFDRIFHITRGGISKASKTINISEDIFAGFNSTLRQGNVTHH

EYIQVGKGRDVGLNQIALFEAKVSSGNGEQLLSRDVFRLGQLFDFFRMLSFFYTSVGYYITTLMTTLVVYLFLYGKCYLALS
GLDHQLQEVADITGNAALNSALNTQFLLQIGVFTAVPMIVNFVLEQGVLKACVSFITMQLQLASVFFTFSLGTKTHYFGR
TVLHGGAKYRATGRGFVVEHISFAENYRLYSRSHFTKAFEIAILLIVYLAYGEDNSGVTYVLLSFSSWFLALSWLFAPYLFNP
SGFEWQKTVEDFDDWTNWLFYRGGIGVKGNESWESWWDEELEHIRSYRGKFWEVVLSLRFFFFQYGVVYSLHVSGSS
TNLAIYGYSWLVLIGLILLFKVFTFTNQSSVNFQLLLRMFQGLLFLGVIAAIATVIATTSLTVGDCFAMLLAFIPTTWGLLSVC
LALRKQLEWLRLWGIVRSGFRLFDAMMGALIFAPVAILSWFPFVSTFQSRILFNQAFSRGLEIGRILQGNRPNAN----------
-------------------------------------------

>PIUF 2003440 Pellia cf. Epiphylla 1917

RGLSRTYTTANMTDVFDSEVVPSSLSSIAAILRVANEIESQRPRVAYLCRFYAFEKAHKLDPTSSGRGVRQFKTALLQRLER
DNGPTLAARHRRSDAREMQSFYQQYYDTYVKALDGADSDRAQLAKAYQTAGILFEVLTAVNKSEMAEPPPEIIAAGKIVE
EKQEILQPYNILPLDTAGASQAIMQLPEIKASVESLRNIRGLPWPSGLEQGRHKSGELDILDWLQSMFGFQKANVANQR
EHLILLLANVHIRLIPKPEPMNKLDERALNQVMEKLFKNYKSWCKFLGRKHSLWLPTIQQEIQQRKILFMGLYLLIWGEAA
NLRFMPECLCYIYHNMASELYGMLAGNVSLVTGENIKPAYGGEDESFLRNVITPIYEILEKEAKSNGNGTAPHSSWRNYD
DLNEFFWSVDCFRLGWPMRLDSDFFVPPANPNHKVSSTDVKKPGISTPKPVGKSNFAEIRTFWHLFRSYDRMWTFYIL
GLQAMIVIAWSVVPNGSIWDTFKGDNFKKVLSIFITAAILRCLQGVLDLALSVHAYRSMKFMGVLRLFLKLVVSGAWVIV
LAVCYVHTWSNPTGLIRDIQKLLGNSWKSPSLYITAVVIYLVPNLLGAVLFVFPMLRRWIENSNWRVVRLLLWWSQPRLYI
GRGMHESQWTLFKYSLFWVLLLVSKFVFSYYIQIKPLVRPTKTIMNARDIKFTWHEIFPHVHDNIGAVISIWAPVILVYFM
DTQIWYSVYSTLFGGISGAFRRLGEIRTLGMLRSRFRSLPGAFNANLVPQETVQRRGFSLARGFKEVQPGKDRKEASKFA
QLWNEVIMSFREEDLISNKERDLMLVPYSSVNLTLVQWPPFLLASKIPIALQMAVEFRGSDADLWRKIKADDYRRCAVEE
CFASFKHVMGTIIVGEVERKLVIDGILAEIGKDISEGTLLTNFKMSALPVLHSIFVELTEYLIKGEEASRDAVIKLLQDMFEVV
TRDMMNETAREYLESTHGPLSISGKTSKDVKDHQLFAATEPKPALLFPPPRTDAWMEQIRRLHRLLTVRESAINVPNNLE
ARRRIAFFTNSLFMDMPRAPRVRNMYSFSVLTPYYHEEVLFSKKQLNEENEDGVSVLFYLQKIYPDEWENFLERMGLED
ERQFWNNDEYVDELRHWASYRGQTLTRTVRGMMYYRRALELQAFLDMASDDELVDGYKIVASAPADMKKSQRSMW
AQLQAIADMKFTYVATCQSYGAQKRAGDVRATDILNLMLNNPSLRVAYIDEREGEEKDKNHKVYYSVLVKAANGLDEEIY
RIKLPGRVRLGEGKPENQNHAMVFTRGEGLQTIDMNQDNYLEEAFKMRNLLEEFHEPHGVRPPTILGVREHIFTGSVSS
LAWFMSNQETSFVTIGQRVLASPLKVRFHYGHPDVFDRLFHITRGGVSKASRVINLSEDIFAGFNSTLRRGNITHHEYIQV
GKGRDVGLNQISLFEAKVACGNGEQTLSRDMYRLGHRFDFFRMLSCYFTTIGYYFSTMIVVLTVYVFLYGRIYLALSGIEKS
LVNSADINNDTALQAALASQSLVQLGLLMALPMVMEIGLERGFRTALSDFIIMQLQLASVFFTFSLGTKTHYYGRTILHGG
AKYRATGRGFVVRHEKFAENYRLYSRSHFVKGIELMMLLIIYSVYGTSARGGIPYLLITFSMWFLVATWLFAPFLFNPSGFE
WQKIVEDWDDWSKWINNRGGIGVKGDKSWESWWSEEQEHLEHTGLRGRVIEVLLSIRFFIYQYGLVYTLNISKANNGL
GVYGLSWLVIIAVLFVLKIVSMGRRRFSADFQLMFRLLKALLFLGFVSVLIVLFLFAKLSISDIFASLLAFLPTGWALLMIAQA
AAPAVRGTGMWDSVKALARAYDFIMGLVIFTPVAVLAWFPFVSEFQTRLLFNQAFSRGLQISRILAGRKK------------------
-----------------------------------------

>PIUF 2011212 Pellia cf. Epiphylla 1770

PTEVYNIIPVHDTLADHSALRYPEVRAAIAGLQIVGDLRKPQEGWESGMDLLDWLGLTFGFQEDNVRNQREHLVLLLAN
AQMRLQPPPDPIDQLVLDVVRKLRKKITKNYLGWCHFLHIKHNLEKLEGRHHEANERKELLYTSLYFLIWGEAANLRFMP
ECLCFMFHYMAGELNRILDMSIDETGGLMQPASQGRNGFLRKVVTPIYEVVRAEAEASGGGAVPHSAWRNYDDMNE
YFWSTRCISQLRWPLDKACNYLVKPAKGKGKVSRQKVGKTGFVEQRSFWNIYRSFDRVWIGLILLLQLLIIVAFRGKSKVS
GQSDKLPWTVLSERDSQAHALSIFITWAALRVLQAFLDFGMQYSLISMGTLMVGLRMTLKLIMSSVWVVLFSVYYSRM

WAQRNQDGGWSSAALNHLYVYLGLAGVFISPEVLALLLFIIPWVRNAVETSDWRIFSLMTWWFQSHIFVGRGLREGTF
DNIKYTLFWIVVLAAKIAFSYFLQVLPLVKPTRSTLHISHGSIHYKWHEFFRNANRTATLCMWAPVVLIYIMDLQIWYSVFS
ALVGALVGLLAHLGEIRDSQQMKARFQFFPSAVQFNLMPEEGLLKQKLFGSWWSRVKDVVRRVKLRYGVHKVKERDG
GKSIEVGRFKLIWNEIIGIFREEDLISDREQVLLEMPSAGWNITVTQWPSVLLSNEILIALGLCDPDHHWYASDRWLWRKI
SSNEYRRCAVIESYESAKHILTRLIKEDSVDRHIFQGIFREIDDSVRQDRFLKTFKLKALRDIHARLLKLIGILVKRPVEDDIQK
VIDALQGLYEVLVRDFPREQSTRLKFKEATSNPGSLLFVDAVELPDQEDHAFFLQLARLEITLSSRVAFLDVPKSLEARRRLS
FFSNSIFMTMPRAPQVEKMLAFSVLTPYYSETVMYNKKDLDTKNEDGVSTLYYLQRIFPDDWDYFVERMARNGVHTEE
KMWKVDDGLELRLWASYRGQTLVRTVRGMMYYHRALQKLSFLDIASEVDIEGYKELLSRTTSGVDVSNGGSGHSSMV
GYTGGSGKRHNEPEMYDGHQQEQDKATAAMKFTYVVACQIYGSQKAKKEQQAADILYLMKTYEALRIAYVDERGDDR
QEKQYSSVLIKYDQELEQEVEIYRVQLPGPLKLGEGKPENQNHALIFTRGEAVQTIDMNQENYFEEALKVRNLLQEFDRK
YGIRKPTILGVREHVFTGAVSSLAWFMSAQETSFVTLGQRVLANPLKVRMHYGHPDVFDRLWFLTRGGMSKASRVINIS
EDIYAGFNCTLRGGTVTHHEYIQVGKGRDVGLNQISMFEAKVASGNGEQILSRDIYRLGHRLDFFRMLSVYYSTVGFFIN
NMLVVLTVYAFLWGRVYLSVSGVEESITGSSNSNTALSAALNQQFLVQMGIFTALPMIVESSLERGFLRAIWEFLTMQLQL
ASVFFTFSMGTRTHFFGRTVLHGGAKYRATGRGFVVKHENFSTNYRLYARSHFIKGLEIILLLIVYQAYGSSKNTVVYILTTFS
CWFLGITWILAPFLFNPSGFDWLKSVEDFDDFMTWIWYKGGVFVKSEQSWEKWWDEEQEHFLSTGFWGKILEIILDLR
FFFFQYGIVYRLKIANGSTSIIVYLISWTYILTAGVVNLVLGFAGDRYGAKQHLKYRSIQALVIMILMLAIVMLFVFTNLSIWD
IFTSMLAFIPTGWGVLMICQVLRRPFLENTPVWPMVVAVGRLYEMAMGIAVMAPVVFLSWLPGFQAMQTRILFNEAF
SRGLQISQIIEGKKP-----------------------------------------------------------------------------------------------------------------
------------------------------------------------------------------------------------------

>PIUF 2014265 Pellia cf. Epiphylla 1929

RIVANWERLARGALSAHALRGGSRVGETDELGSLVPPSLGQQSNIDIILQAAEEIQPDNAQVARIVCEYAYTMAQDLDPQ
SAGRGVLQFKTGLLSVIKQKKSKKEGERIDRSQDIELIQQFYEYYRSKHDIDDLERIRGEYSSFEDNPQEYERTTQIIKKVYQT
SRVLNDVLNALTRDATPAEKDQVQHELKIRMETDALKTLDFKAYNILPLETPGVTDLIMSFDEVKAAAASLEYTTDLPELP
DDLIKPEHRPVDIFDLLEYIFGFQEGNVNNQREHLVLLLSNSQSLLAPPTDNFGLRLDDSAINRCHDRLLDNYIKWCEYLR
MESMTPRNEKGQQKVIFMAMYLLIWGEAANLRFLPECLCYIFHHMVKELRAILDEHVVQHSKIYKTYEGKQYSYLDQVV
TPIYDTILAESKNNNEGKGSHASWRNYDDFNEYFWSTQCFDLNWPLRLDAGFLMKPKKTKGPSDDKMVPSTGSGHKK
EKRVGKMTFVEHRTGLHVYHSFHRLWIFFTVMLQGMMVVAFSNGKLNTRTFKKMLSVGPTYMLMKFVQSLFDLLFIY
GAYASTQGWTFSRMFTRLLYYGGLGGGVTFLYVKMINEANKGTASTWYNLFLIVLGSYIASQVIVTSLLRIPLFRRQAEKRT
NWSIMRFIFWVHQDRYFVGRGMYERTRDYLRYAAFWVFVLACKFAFSYHFQIKSLVSPTKIITKLGRNRLHYKWHDFFSS
GHHNALTVASLWAPVIMIYYLDTQVWYTVVSALVGGLVGARARLGEIRTLSMLRKRFSTFPAEMSRVLVPSRIQTQQIIRT
PSVISGQAGWEKKKVDAFKFAPLWNEIINTLREEDFISNKEKESLLMPFNEGPSQVVQWPLFLLANKVYLAVEMAQDNK
SINQEGLWARIEKDEFMKYAIQEAYGSLETVLKALVNDEAKTWVRSVFGDLEGGMQEGMLAGHFHLKNVSVVLGRIINL
TAVLMHDDEPKWNRDAVIAMQNLHEAVMQDFLTTDLREKFESWTLLSQAKNEGRLFSKLNWPKSKEEKEAVRRLNLLL
TTKESAANVPRNLEARRRLAFFTNSLFMDMPVAPPVRKMLSFSVFTPYYKEDVMYSTDQLVEENEDGISTLFYLQKIFPD
EWRNFSERVQLTGSEMQRQLKNEKFDPIELRLWASFRGQTLARTVRGMMYYKKALVLASLLEGSETLDVEEGLSSARSM
TENNPGFRRARALAELKFTYVVACQLYGEQRQMKDAKAADIAYLMRKYESLRVSYVDIVEGQKDDKKVKTYYSKLIKADI
DGKDQEIYSIKLPGPFKIGEGKPENQNHSIVFTRGDAVQTIDMNQDNYFEEAFKMRNLLEEFKATNNLRRPTILGIREHVF
TGSVSSLAWFMSQQETSFVTLGQRVLAQPLKVRMHYGHPDVFDRIFHITRGGISKASRTINISEDIYAGFNCTLRQGNIT
HHEYIQVGKGRDVGLNQIALFEAKVSSGNGEQVLSRDVFRLGQLFDFFRMLSFFYTSVGYYITTLMTSLVIYLFLYGKCYLA
LSGLDQQLRVVADVSGNTALSSALNTQFLLQIGVFTAVPMIMNFILEQGVLKAFVSFITMQLQLCSVFFTFSLGTKTHYFG
QCVLHGGAKYRETGRGFVVHHIGFAENYRLFSRSHFTKAFEIVMLLIVYLSYGQDNSGITYILLTFSSWFLALSWLFAPYIFN
PSGFEWQKTVEDFDDWTNWLFYKGGIGVNGKESWEAWWDGELGHIRTTRSRIWEVILSLRFFFFQYGVVYSLHVSGS

TTSLVVYGYSWIVFVGMIVLFKIFTFTNKSSLNYQLFLRLFQGAFFVSLVAGLAISIAPLAISIAVTTLTVGDCFAIFLAFIPTG
WGLLSIAVALRPILEYVGLWDVIQSGARLYDAMMGSLIFTPIAILSWFPFVSTFQTRLVFNQAFSRGLEISLILAGNRANTST
-------------------------------------------------

>TGKW 2015918 Frullania 1757

YNIIPVHDTLSDHPALQFPEVRAAIAALQVVGDLRIPFEGWAPGMDLMDWLGLTFGFQEGNVKNQREHLVLLLANSQ
MRLQPPPDPIDKLVLDVVRRLRQKVTKNYLSWCRFLHLTHRLEKLEGRRHESDERKELMYTALYFLIWGEAANLRFMPEC
LCFIFHHMAGELNKILMLSIDEAGQKIQPASYGVNGFLRYVIFPIYEVVQAEAATGGGGAPHSAWRNYDDMNETRLGW
PLAKTCNYLVKPAKGKGKVSRQKVGKTGFVEQRSYWNVFRSFDRLWVGLILLLQLLITVAFEAKGRRTGQSDRMPWTVL
SHRDSQAHALAIFITWSALRVLQALLDFGMQYSLISAGTIRVGIRMTLKVIVASVWVVLFSVYYGRMWAERHRDRGWSS
AALERLYVYLGFCLVFISPEVLALLLFIIPYVRNAVETSNWRIFSPITWWFQSKIFVGRGLREGFIDNLKYTLFWIAVLAAKIA
FSYFLQILPLVKPTRATLDIRDGTINYKWHEFFKNANRMALLCMWAPVILIYFMDLQIWYSLFSALVGALVGLLGHLGEIR
DSVQIRSRFRFFPMAVIFNLMPEDGLLKQRPFYNAWGRVKNAVKRIKLRFGVQGRKKDAAARSMEVAKFKQIWNEIIGI
FREEDIISNDERDLLEMPTTGWNISVTQWPLFLLSNEVRIALRFCDPEHNHYWYHSDRWLWRKVAHNEYRRCAVIECYE
SLKHLLTRLIRPSSDDAQIFPLIFKEIDNAIQQGLFMKTFKMKEFPKLHESLITLTGLLMKRPRLNDLQLVVDALQNLYEIVVR
DFPRDTNVKIKFKELASRSLLFVEAVELPNPEGDDAFFYQLKRLHMSLNTRVSHLDVPKSLEARRRISFFSNSIFMTMPRA
PHVERMLAFSVLTPYYKEDVLYDKKQLYTQNEDGVSTLFYLQRIFPDDWRHFQERMAGRGIHTDEETMWQHEDGLEIR
LWASYRGQTLVRTVRGMMYYGRALQKLAFLDLASEHDIDGYKELLSRTASTMEITTTSGSRHSSFGYGRSGRSFRAPELY
DGHQKEQDTALAAMKFTYVVACQVYGEQKRLNDPKAADIRYLMQTYEALRVAYFDQTGGDREPKRYYSVLVKFDQLLQ
QEVEIYRVELPGPLILGEGKPENQNHALIFTRGDAIQTIDMNQENYFEEALKMRNLLQEFDKSRGIRKPTILGVREHVFTG
AVSSLASFMSAQETSFVTLGQRVLANPLKVRMHYGHPDVFDRLWFLSRGGLSKASKVINISEDIYAGFNCTLRGGTVTH
HEYIQVGKGRDVGLNQISLFEAKVASGNGEQMLSRDVYRLGHRLDFFRMLSVYYTTTGFFINNMIVVLTVYAFLWGRCY
LAVSGIEESITGSAKSNTALSAALNQQFLVQMGILTALPMIVENSLERGFLRAIWEFFTMQLQLASVFFTFSMGTRAHYFG
RTILHGGAAYKATGRGFVVKHEGFTANYQLYSRSHFVKGIEIILLLIVYEIWGASKNTVVFVLTTFSCWFLGITWIMGPFIFN
PSGFDWLKSVNDFDEFMGWIWYKDDFLFVKATDSWKKWWDDEQEHFRHTGFWGKVMEIVLDLRFFFFQYGIVYRL
QIANGSTSILVYLLSWTYIFAAGLIHLALGLSSERYGAKQHLKYRSIQALVVMLLLLAIVILFVFTHFAIWDIFTSLLALLPTGW
GLILICQVLRRPFLENTPVWPMVVAVARLYDLMMGIFVMAPVVFLSWLPGFQAMQTRILFNEAFSRGLQFSQIVAGR---
---------------------------------------------------------------------------------------------------------------------------------------
-------------------------------------------------------------------------------

>UUHD 2011853 Porella pinnata 1788

RAVDNWEQLVRNALVSGELRGRSRPEGELRGRHRPERDDELGGAVPPSLGQESNIIDILQAAEEIQPLDAQVARILCEYAY
NMAQNLDPQSEGRGVLQFKTGLLSVIRQKQSKREGEKIDRSQDIERIQSFYEHYRTTHKIDELERRQKETGVLDDNPAEH
ERRTQRLKKVYQTSRVLNIVLNALTRDSTPEERDSWIQKELKDRMEMDANKNADFKAFNILPLETPGVADAIMLFEEVK
AAAGSLAYTTDLPQFPEDYLRPKYRPLDIFDLLEYTFGFQEGNVNNQREHLVLLLSNSQSLLGVPTQGLMLDESAINRCFE
RLLDNYVKWCDYLRIEPMTVRAKTGQRKLILMGMYLLIWGEAANLRFLPECLCYIFHQMVTELYEILVGSAVQRSKTYKP
HDKDGKQYSYLDQVVTPIYQTVLAEAKNNKGGKASHAAWRNYDDFNEFFWSSRCFVLGWPWRSDAGFFLKPRKKGT
PIEGSVKTPGQRKVKRVGKMTFVEHRTFFHIYHSFHRLWIFFTIMLQAMMVVAFCNEKLNTQTFKKMLSVGPTYLIMKT
VQSLFDLFFIYGAYGSTQGWTFSRIFTRLFFYGGLAAGILFLYIKMMQEDGRGAATTWYNLFLIVLGSYAGAQLLATAVLRI
PLFRRQLDKCGNWGIIRFIYWVHQDRYFVGRGLYERSRDYLGYTAFWVVVLACKFAFSYHFQIRSLVSPTKTIVNLPANEL
QYKWHDFISAKNHNALAVASLWAPVIMIYYLDTQVWYTVTSALIGGLSGAKARLGEIRALSMLRKRFSTFPEEMARNLLP

SRIQPQQMNRVQSSLGVQAGWPEPKVHAFKFSPVWNEIIISLREEDYINNREKELLLMPSNHGASTMVQWPLFLLANK
VYMAVEMAQDNKSLNQTGLWERIQKDEYMAYAVQEAYHTSEAVLKALVNNEAKTWVHTVFGDVDAVIQGSMLVGHF
HLKQIDLVLSRIRVLTALLIRDEDENGKKAAVKAMIDLYETVLHDFLSAELRDEFLGSRLLSEAKSQGRLFSNLNWPRTREEK
EAVKRLHNLLTIKESSANIPRNLEARRRLQFFTNSLFMHMPVAPPVQQMLSFSVFTPYYAEDVMYTKEQIREENEDGISIL
FYLQKIFPDEWKNFQERVGLINAVIEEQLRNKSLDMLELRLWASFRGQTLARTVRGMMYYKKALILASLLEEPTVDIEEGL
QSFAGTGDSNPGFRKARALAELKFTYVVTCQIYGVQKQNRDVRAADLSYLMRKYESLRIAYIDMVEEKTSTTYYSKLIKAD
VDGNDQEIYSIKLPGEFRLGEGKPENQNHAIVFTRGDAVQTIDMNQDNYFEEAFKMRNLLEEFKAQNCLRRPTILGIRE
HVFTGSVSSLAWFMSQQETSFVTLGQRVLAHPLKVRMHYGHPDVFDRIFHITRGGISKASKTINISEDIYAGFNSTLRQG
NVTHHEYIQVGKGRDVGLNQIAIFEAKVSGGNGEQILSRDVFRLGQLFDFFRMLSFFYTSVGYYITTLMTSLVIYLFLYGKC
YLALSGLDRQLQIVADISGNAALSSALNTQFLLQIGVFTAVPMIMNFILEQGFLKAVISFISMQLQFCSVFFTFSLGTKTHYF
GRTVLHGGAKYSATGRGFVVRHISFADNYRLYSRSHFTKALEIAMLLIVYLAYGEDNSGLTYILLSFSSWFLALSWLFAPYIF
NPSGFEWQKTVEDFDDWTNWLFYKGGISVKGAESWESWLEEEQVHIKSWRGRIWEIVLSLRFFFFQYGVVYSLSVSGS
STSLAVYGYSWIVLIGLIILFKIF-----------------------------------------------------------------------------------------------------
------------------------------------------------------------------------------------

>UUHD 2150804 Porella pinnata 1930

ESQSQGSRPVRRGLSRTYTTANFSDVFDSEVVPSSLSSIAAILRVANEIESQRPRVAYLCRFYAFEKAHKLDPTSSGRGVRQ
FKTALLQRLERDNIPTLSARHRRSDAREMQSFYQQYYDTYVKALDGAEHSDRAQLAKAYQTAGILFEVLTAVNKSEQAEP
PPEIIAASKIVEEKQEILQPFNILPLDAAGASQAIMQLPEIKASVESLRNIRGLPWPPGWEQGRHKSGELDLLDWLQSMF
GFQKANVANQREHLILLLANVHIRLIPKPEPMVKLDDRALNQVMDKLFKNYKSWCKFLGRKNSLWLPTIPQEIQQRKVL
YMGLYLLIWGEAANLRFMPECLCYIYHNMAYELFGMLAGNVSLATGENIKPAYGGDEESFLRKVITPIYEIVEKEARSNCN
GTAPHSAWRNYDDLNEFFWSVDCFLLGWPMRLDSDFFVPPAIYNRKVPNSDVKKTGGPAPKYLGKTNFVEIRSFGHLF
RSYDRLWTFYILGLQAMIVIAWSVEPNGSLGRIFEGDNFKKILSIFITAAILRCVQGVLDFALSIHAYRSMKFMGLLRLVLKL
CVSGAWVIVLAVCYAHTWSNPTGLVRSIQRLLGSSWKSPSLYITAVVIYLIPNILGAILFVFPMLRRWIENSNWRIVRFLLW
WSQPRLYVGRGMHESQWTLFKYSVFWILLLASKFIFSYYIQIRPLVRPTKTIMNARDIQFTWHELFPNVRKNIGAVISIWA
PVVLVYFMDTQIWYSVYSTLFGGISGAFRRLGEIRTLGMLRSRFRSLPGAFNANLVPAEKVAKRGFSLARGYKEVQPGKD
RKEASKFAQLWNEVIMSFREEDLISNKERDLMLVPYSSSVNLTLVQWPPFLLASKIPIALQMAVEHRGNDADLWRKIRAD
DYRRCAVEECFESFKNVLGIILVGELEKRVVIDGILAEIEKDIAEGNLLANFKMSALPVLHSKFVELTEYLIKGEEGSRKFVVK
RLQDMFEVVTRDMMNETAREYLESTHGPLSISGKTSKDVKDHQLFAATEPKPALLFPPPPTDAWLEQIRRLHRLLTVKET
AVNVPNNLEARRRIAFFTNSLFMDMPRAPRVRNMYSFSVLTPYYHEEVLFSKGQLNEENEDGVSVLFYLQKIYPDEWEN
FLERMKLQDERQFWNNDEYVDELRHWASYRGQTLSKTVRGMMYYRRALELQAFLDMASDDELVEGYKVVASAPADT
KKSQRSMWAQLQAIADMKFTYVATCQSYGAQKRAGDVRATDILNLMLNNPSLRVAYIDECEDEEKDKDQKVYYSVLVK
AANGLDEEIYRIKLPGPVRLGEGKPENQNHAMIFTRGEGLQTIDMNQDNYLEEAFKMRNLLEEFHEPHGVRAPTILGVR
EHIFTGSVSSLAWFMSNQETTFVTIGQRVLASPLKVRFHYGHPDVFDRLFHITRGGVSKASRGINLSEDIFAGFNSTLRRG
NITHHEYIQVGKGRDVGLNQISLFEAKVACGNGEQTLSRDMYRLGHRFDFFRMMSCYFTTVGYYLSTMTVVLTVYIFLYG
RIYLALSGIEKSLVNSADVKNDTALQAALASQSLVQLGLLMALPMVMEIGLERGFRTALSDFIIMQLQLASVFFTFSVGTKT
HYYGRTLLHGGAKYRATGRGFVVRHEKFAENYRLYSRSHFVKGIELLVLLIIYSVYGTSVKAGVPYLLITFSMWFLVATWLFA
PFLFNPSGFEWQKIVEDWDDWSKWINNRGGIGVQADKSWESWWSEEQEHLENTGLRGRLIEMLLSVRFLIYQYGLIYT
LNISKGSKGLGVYGLSWLVIIAVLAVLKIVSMGRKRFSADFQLMFRLLKALLFMGFVSVLVVLFVFAHLAVSDIFASLLAFLP
TGWALLMIAQAARPIVRPTGMWDSVKALARAYDFIMGLVIFTPVAVLAWFPFVSEFQTRLLFNQAFSRGLQISRILAGRK
K----------------------------------------------

>WJLO 2001546 Riccia berychiana 1782

YNIIPVHDTLDPHPALRYPEVRAAIAALQVVGNLRRPAEWVSNYDMLDWLGMTFGFQKSSVRNQREHLVLLLANAQM
RLQPPPDPIDKLQIEIVRQIRRKLTKNYIKWCSFLHIKHNLEKIEDHGIRRKHESNEQKELLYASLYLLIWGEAGNLRFMPECL
CFIFHNMTYELNRILDASIDDGGQHMQPASQDGFLHKVIRPIYAVAKAEADKSGNGKNPHSAWRNYDDMNEYFWSH
RCLTQLRWPLEMSCNYLVKPEDNSRHKVGKTGFVEQRSFWNIYRSFDRVWIGLILMLQILIIVAFRAKPEIRDPNSIEVPAP
LITGPARAPWTVLTERDSQAHLLSIFITWSALRVLQAFLDFGMQYSLISAGTLLVGLRMTLKFIIASVWTVLFSVYYSRMLA
QRDADGSWSDQALQRLYLYLGFCAVFVAPEVLALLLFIINFVETKDWRIFSLLTWWFQSHIFVGRGLRESVLDNIKYSLFW
LSVLAAKFSFSYFLQVLPLVKPTRATLNITGIDYRWHEFFKNANRTATLAMWAPVVLIYFMDLQIWYSVCSALVGALVGLL
AHLGEIRNAAQLKARFHVFPLAVQFNLMPEETFLNQRPHTWRRMTYEWWAATKNIGRRIWLRYGVVKGQQAEGKT
METGRFRHVWNEIILIFREEDIISDRELELLSMPSSVWNISVTQWPSVLISNEVLIALGLCEEWSYSDRAFWKRIASNEYRR
CAVIESYESIKHVLRRILREDHKELGIFEEIFGELERAIHENQFIHRFELKNMKRIHSRLVELIAVLLKRPLLNDRKKVVDALQN
LYEDLHREFPKDQELCRRWKDLGATSSLLFVDAVELPDAGDDAFFSQLKRLHTTLSTREALLNVPKSLEAKRRISFFSNSLF
MTMPRAPQVDKMLAFSVLTPYYAETVMYDRKQLEKENEDGVSTLFYLQKIFKDDWEYFKERMGRRGIRPKDSVQDDR
DDMFMSMLWKIDDGLELRLWASYRGQTLVRTVRGMMYYQRALQKLAFLDVASELDIEGYKELMVRTADGVDAFNGE
SLEGSVPSVSSHNESREGGTLYTDYQKEMDMATAGMKFTYVVACQIYGAQKKKGEQQAKDILYLMKTWNALRIAYVDE
EMGPTDKDPKTYSSVLVKWDPVREEEVEIYRVELPGPVKLGEGKPENQNHAVIFTRGDAVQTIDMNQENYFEEALKMR
NLLQEFKRYHGIRKPAILGVREHVFTGSVSSLAWFMSAQETSFVTLGQRVLAKPLKVRMHYGHPDIFDRLWFIPRGGLSK
ASRVINISEDIFAGFNATLRGATVTHHEYIQVGKGRDVGLNQISMFEAKVASGNGEQILSRDIYRLGHRVDFFRMLSVYYS
TVGFFVNNMIVVLTVYAYLWGRVYLALSGIEVSITENSSANGALTAALNQQFVVQMGIFTALPMIVENSLEKGFLPAIWEF
LTMQLQLASVFFTFSMGTRAHFFGRTVLHGGAKYRATGRGFVVQHEKFAVNYRLYARSHFIKGLEIILLLIVYQMYGSVKD
TAVYILTTFSCWFLGITWILAPFLFNPSGFDWLKSVDDFDDFMTWIWYKGGVLVKADVSWRQWWDEEQEHLQSTGF
WGKCLEIILSLRFFFFQYGIVYRLKIANHSTSIIVYLISWTYLFVAGLIHLIIAHASDKYGAKKHVKYRGIQAVVLCFLVLTIVLLF
VFTRFSIWDVFTSMLAFIPTGWGVLSICLVLRRPFLEHTPVWPMVTAVARLYEMGMGIVIMAPVVILSWVPGMQAMQ
TRILFSEAFSRGLNIQKILTGKK-------------------------------------------------------------------------------------------------------
--------------------------------------------------------------------------------------------

>WJLO 2001956 Riccia berychiana 1977

RAIDNWEKLVRNALNSEELSSRLGRRDELGRVVPTSLGQQSSSINLILQVAEEIQPDNPHVARILCEYAYSMAQQLDPQSE
GRGVLQFKTGLMSVIRQKQSKKEGERIDRTHDIVQIQRFYEEYRRFHKIDELERLQQQRGRVREDAGEVERRTQKMKKV
YQISKVLNDVLNSLTENTTEEEREKLISKETKVKMETAAAKTAEFKPFNILPLETPGVPDTIVLIEEVNAAVHTLGYSEQLPEL
PEGAVKAGLQRKSDIFDLLEYIFGFQIGNVNNQREHLILLLANSQAALGLPSRADQVDEGAIRRCYDRLLDNYMKWCQYL
RIQAVTDSATGDPRRMVLLTATYLLIWGEAANLRFCLCYIFHHIVKELEQLLLNPVAERSKLNAEGTETAFLDRVVTPLYQV
VAAEAANNEEGKASHAAWRNYDDFNEYFWSPRCLESLSWPWRQDAGFMMKPKKKEGKVIPLGKKKGEKKVGKMTF
VEHRTGFHVYHSFHRLWIFFTVMLQALMIFAFSDENLNGRTFKKMLSVGPTFVIMKFIQSFLDIFFIYGAYASTQGWTFSR
IFARLGWFGGLSGAITWLYSKMIQEDNNGAGTTWYHLFMIVLGSYLAAQLLVTVVMRIPFFRRRTERCSDWLIIRFLMW
LHQDRYFVGRGLYERTRDYAGYTAFWIVVLAGKFAFSYYFQIRALVSPTKSIIETTFDYKWHDFVSRHNHNALTIGSLWAP
VILIYYLDTQVWYIVASAFVGGLDGASSRLGEIRTLSMLRKRFASFPDAFAKNLVSSRLGRGEKNPASSPSQVNWSPKKLN
AFKFAPMWNETINCLREEDYISNKERNLLVMPRNQWPGTLVQWPLFLLANKVYLAVEMAQDSKSLNQQQLWERVSK
DEYMAYAVEEAYKLLEEVLISLSKHDAAQIWVKGVFNDVNAGIQESALVGHFYLKRVDSVLSKVQALTAILIHEETVKSKAQ
AIMAMQDLYEAVMNDFFTAELRERIEEFRALKEARMEGRLFINLEWPSLDREKENVKRLHGLLTNQESAANIPRNLEAR
RRLEFFTNSLFMDMPSPPSVLRSLSFSVFTPYYSEDVLYSKKKLKEENEDGISILFYLQKIFPDEWKNFLERQVLTGKEMER
QLENDSLEMMDLRLWASYRGQTLARTVRGMMYYKKALILQSLLEGNSDVEEGGFLGTTLEDSPGYRMARATAEMKFT

YVVTCQIYGQQKQKGEQAAADILYLMNKYESLRVAYVDIVDKVQEDRDGKSVNIKEFYSKLIKADADGKDQEIYSIKLPGN
FKLGEGKPENQNHAIVFTRGDACQTIDMNQDNYFEEAFKMRNLLEEFNSTNCLRRPTILGIREHVFTGSVSSLAWFMSQ
QETSFVTLGQRVLARPLKVRMHYGHPDVFDRVFHITRGGISKSSRTINISEDIFAGFNSTLRQGNITHHEYIQVGKGRDVG
LNQIAMFEAKVSSGNGEQILSRDVFRLGQLFDFFRMFSFFYSSVGHYITTLMTTLVVYVFLYGKVYLALSGMDQQLQSFA
DISGNKALESAINTQFLLQIGIFTAIPMIMNFILEQGVLKAFISFCTMQLQLSSVFFTFSLGTRTHYFGRTVLHGGAKYRATG
RGFVVQHISFADNYRLYSRSHFIKALEISMLLITYLAYGQDNKGVAYILLSFSSWFMALSWLFAPYIFNPAGFEWQKTVEDF
DDWTNWLFYKGGIGLKDTESWEAWWEGEQSHIRTLRGRFWEIILSLRFFFFQYGVVYSLNVSGDSTSLAIYGYSWIAFA
AIILLFQVFTFTTKSSVKFQLFLRLFQGGLFLALVAAVTVTIVFTSLTIGDCFATLLVFIPTGWGLLSVSIASVTDIVRYSLIGFLS
HLVPSFSFVNRCTVSEMETVQYLKTAPSSGTYWFVLVNDERCLVVLPAICQAIKPILERLRLWDAVRSGFRLYDAMMGAVI
FTPVAILSWFPFVSTFQTRLVFNQAFSRGLEISLILAGNRPNAST

>YBQN 2130644 Odontoschisma prostratum 1931

GSRPSRRGLSRAYTTASFSDVFDSEVVPSSLSSIASILRVANEIERERPRVAYLCRFYAFEKAHKLDPTSSGRGVRQFKTALLQ
RLERDNVPTLQARYKRSDAREMQSFYQNYYDNYVKALDGTEHSDRAQLAKAYQTAGILFEVLTAVNKSEQAEPPPEIIAA
SKIVEEKQEILQPYNILPLDAAGASQAIMQLPEIKASVESLRNIRGLPWPPGWEQGRHKSGELDLLDWLQSMFGFQKAN
VANQREHLILLLANVHIRLIPKPEQMNKLDDRALNQVMDKLFKNYKSWCKFLGRKNSLWLPTIPQEIQQRKVLYMGLYL
LIWGEAANLRFMPECLCYIYHNMAYELFGILAGNVSLVTGENVKPAYGGEEESFLRKVITPIYNIVEKEARSNCHGTAAHS
AWRNYDDLNEFFWSVDCFRLGWPMRLDSDFFVPPASYSPKVPAFKEKPVPAPDVKKTGLTPRHLGKSNFVEIRSFGHLF
RSFDRMWTFYILGLQVMIVIAWTIEPNHPLSKVFEGDNFKKILSIFITAAILRCIQGVLDIVLTAHAYRSMKFFGIVRLFLKLF
VSAAWVVVLSVCYVHTYNHPTGLVRSVQKLLGSSWKNPSLYITAVVIYLVPNILGAVLFIFPMLRRWIESSNWRIVRFLLW
WAQPRLYVGRGMHESQWTLFKYSLFWVLLLTSKFVFSYYIQIRPLVKPTQTIMRARNIQYTWHELFPHVKNNLGAVISIW
APVILVYFMDTQIWYSVFSTLFGGISGAFRRLGEIRTLGMLRSRFRSLPGAFNANLIPTDKAARRGFSLARGYKEVQPGKD
RKEASKFAQLWNEVIMSFREEDLISDKERDLMLVPYSSVNLSLVQWPPFLLASKIPIALQMAVEHRGNDADLWRKIKADD
YRRCAVEECFESLKLVLGTILVGEIERRVVIDGILAEIEKDIAEGTLLTNFKMSALPVLHSTFVELTEYLIKGEKSSRKYVVKRLQ
DMFEVVTRDMMNETAREYLESTHGPLSISGKTSKDVKDHQLFAATEPKPALIFPPPPTDAWIEQITRLHRLLTVKESAVNV
PNNLEARRRIAFFTNSLFMDMPRAPRVRNMYSFSVLTPYYHEEVLFSKTQLNEENEDGVSVLFYLQKIYPDEWENFLER
MKLQSELQIWNNDEYVDELRHWASYRGQTLTRTVRGMMYYRRALELQAFLDMASEDELVEGYKVVASAPADTKRSQR
SMWAQLQAIADMKFTYVATCQSYGAQKRAGDVRATDILNLMLNYPSLRVAYIDECEDEEKDKDQKVYYSVLVKASNGL
DEEIYRIKLPGPVRLGEGKPENQNHAMIFTRGEGLQTIDMNQDNYLEEAFKMRNLLEEFHEPHGVRPPTILGVREHIFTG
SVSSLAWFMSNQETSFVTIGQRVLASPLKVRFHYGHPDVFDRLFHITRGGVSKASRIINLSEDIFAGFNSTLRRGNITHHEY
IQVGKGRDVGLNQISLFEAKVACGNGEQTLSRDMYRLGHRFDFFRMLSCYFTTIGYYFSTQIVVLTVYVFLYGRIYLALSGI
EKSLVNSADINNDTALQAALASQSLVQLGLLMALPMVMEIGLERGFRTALSDFIVMQLQLASVFFTFSLGTKTHYYGRTIL
HGGARYRSTGRDFVVRHEKFAENYRLYSRSHFVKGIELMVLLIIYSVYGTSVKAGIPYLLITFSMWFLVATWLFAPFLFNPS
GFEWQKIVEDWDDWTKWINNRGGIGVQAEKSWESWWKQEQEHLEHTGFRGRLIEMLLSVRFFLYQYGLVYMLHISR
RNKGLGVYGLSWLVIIAVLAVLKIVSLGRKRFSADFQLMFRLLKALLFLGFLSVLVVLFVFAHLSVADIFVSLIAFLPTGWALL
MIAQAARPVVRSAGMWDSVKALARAYDFIMGLVIFTPVAVLAWFPFVSEFQTRLLFNQAFSRGLQISRILAGRKK--------
--------------------------------------

>AKXB 2007915 Phaeomegaceros coriaceus 1933

KAKKNWEQLVRKALLRVGDEPGATGWDRLFGAPSYDTTPALPSALGNTSIDAVLAASNEIQTESPQVARILCEHAYRLSQ
NLDPKSQGRGVLQFKTGLLSIIKQKRSKKDGEQIDRSNDVKLIFEFYKTYRKKLDGLEDDDKKFRQLGERGDVVLKPEDKK

RVADIKRMYETSRVLNEVLDAYLKETSPEERESLLGDQQLKRDMERDAAKIAEFKSYNIIPLETPSAGPFVLLPEIKGAHYAL
KLGGEIPALPDTYPNPNRPLDMFDLLQYIFGFQEDNVKNQREHVILLLANTQARLSLPDDRAEGFTVRLDDGSMRHLHN
KVLDNYKKWCSYLRKTSVLKKGNSTHSAPVQLLLTCLYLVIWGEAGNVRFLPECLCYIFHNMASELLLQLGKPVADRARGV
GDGDHEFLDQVVKPVYDIIAAEAKHGQGKKGSHSQWRTYDDLNEYFWSDHCFTLGWPWTRDSSFFLQPGGGKQDIA
NEAAPGRRKQLRMGKTLFVEHRTGFHIYHSFHRLWIFFIMMLQGLGIFAFSTQLQTLHRIKVILSLGPSFVLLKFVQSLFDV
ILMYGAYASTRGQNVARIFVRFIWFSASSAFVMYLYVRVMSEPDLNSSSALFNIYILVLGCYAGFQLIVSTISRISALRVQAD
KIGGWSIIRFISWVHQERYYIGRGLYEKTQDFLLYMLFWIVVLVAKFSFSYFFQIKPMVEPTRIIIRKTIENYKWHDFVSKSN
HNALTLVALWAPVVMIYFVDTQVWYTVFSALWGGLDGARLRLGEIRSLSMLRKRFNTFPEEFVSRLLPSVLKLKIDTSSAN
GQQAPQKDKVNALKFAPLWNEIIDNLRLEDYISNKERDWLLMPKNTGSLQLVQWPLFLLANKILLAVDLAAEHKGSQYE
LQEKIFKDEYMQFAVQEAYFSVQRMMMKLVNNEAELWVKGIFADIEASIKESALATSIVLKRLGGPGGLVAKLATLTGILEK
EENPVLLKNATLALQEIFEVVMKDVLSLTLREKFESWSVLEKARRTGALFSDLNWPSTEEENEEIKRLNSLLTVKDSASNIPK
NLEARRRLEFFSNSLFMDMPATPSVRQMFSFSVFTPYYAEDVIYGYAALNKENEDGITILFYLQKIFPDEWKNYLERVFRTE
AQLERELARHLEKLKENKEPTEISGTPELLQLRLWASYRGQTLARTVRGMMYYRSALILQSKLEASGVPDEEAATRPVAGA
MPASLMGQSIADMKFTYVVSCQIYGKQKQQRHQQAADIAYLLQTHEGLRVAYIDTVEKIIDGKTSSEYFSKLVKADATGK
DQEIYSIRLPGNPLLGEGKPENQNHAIIFTRGEAIQTIDMNQDNYFEEAFKMRNLLQEFDVQHGLRKPSILGVREHVFTG
SVSSLAWFMSNQETSFVTLGQRVLAKPLKVRMHYGHPDVFDRIFHITRGGISKASKVINISEDIYAGFNSTLRQGNVTHH
EYIQVGKGRDVGLNQIATFEAKVASGNGEQVLSRDIYRLGQLFDFFRMLSFFYTSIGFYVCTMMTVLTVYVFLYGKAYLAL
SGVGAALQDDVVNSTALQTALNTQFLFQIGIFTAIPMIMGFILEQGVLKAFISFITMQLQLASVFFTFSLGTRTHYFGRTILH
GGAKYRATGRGFVVQHIPFAENYRLYSRSHFVKGLEVALLLIVYGAYGTSKGTVSYVLLTFSSWFLAISWLYAPYIFNPSGFE
WQKTVEDFDKWTNWIFYKGGVGVNSNESWEAWWEEEQAHITSPRGRFWEVILSFRFFIFQYGVVYSLSATGSNTSLG
VYGISWLVLVGLLVVFKAFTFSQKASAKFQLVVRLGQGILFLLLIAGIVLSIVYTELSVGDVFASLLALLPTGWAILSICLAIRPL
VESLRLWKSVRAFARFYDALMGMVIFVPIAVLSWFPFVSTFQTRLVFNQAFSRGLEISLILAGNKPN------------------------
-----------------------

>AKXB 2011770 Phaeomegaceros coriaceus 1772

YNIIPVHDTLADHAALQFPEVRAAIVALQTVGSLARPPYTEWRDGMDLLDWLGAFFGFQADSINNQREHLALLLANGQ
MRLNPDPIDKLSNRVVSSIRKKVTKNYTGWCRFLEIPSNMSIIDSKYTSVNERLELLYTGLYLLIWGEAANLRFMPECLCFL
FHNMAGELNRILEDRIDEGTAQPARPAYCEPGGFLKRVVIPLYNIVSAESNASEHGKAPHSAWRNYDDMNEYFWNKRC
LTRLGWPLSLSSNYFVEPQAAGSTSRVGKTGFVEQRSFWNIYQSFDRLWIGLILFLQASIILAWNGDQAPWTELRNKDQ
VARVLSVFITWAVLRLLQAVLNMVMNLRLVSAETLSLGLRMVLKVVVAVAWTIAFAVLWSSLWNLRKAEGEGFDWTPE
SNKRFRNMFYAGAVFIVPETLALLLFILPWIRNFVENSQWRIFHLLTWWFQSRIYVARGLREALLDNIRYTLFWIVTIVTKF
AFSYALQIQPLIKPTKETIRLPKDAVTYRWPELFGRGTRFAIVAMWAPVILIYMMDLQIWYSVYSSLVGALVGLFQHLGEIR
NVEQLKERFQYFASALQFALMAEDQFVPQKGFGRNFWVGAKNSVKNSYTRFKLRYGFQDDSYKKDEKQIEAGRFSYIW
NEILAIFREEDLISDSELELLEVPAKSWNISVMQWPSVLLSNAILVALGLCKNFYGNDKALWRRVSRNEYRRCAVIECYESA
QHVLLKIVAENTEEYIIVKAILDELNLSLRLGTFLKKFDLNQLPNIHKKVAELTGALLGWSKKQQTRDKVVTALQNLIRDTLIR
DFPKSSGTYEEFGQGSSVYPSRSDGLLFTADDSILLPDPEDHAFYKQLKRLNTTLSTKEAILNVPQSLEARRRITFFSNSLFM
TMPRAPQVDRMLAFSVLTPYYKETVLYENWELEKRNEDGVSTIFYLQQIFPDDWKNFEERMGRQGISKIEINTTETGLFE
RRLWASYRGQTLARTVRGMMYYNRALEMLAFLDGASEVEVEQVQEMFLRTSSAGPSIRPMREINQHSSRHSSGRLQSI
RDKHRATAAMKFTYVAACQVYGQQKADDQKPVSKKEITHPARDILYLMKTYEGLRVAYVDEKPVGRDAKEYYSVLVKYD
HATQREVEIYRVQLPGPLILGEGKPENQNHAFIFTRGDAVQTIDMNQENYFEEALKMRNLLQEFTIYYGLRRPTILGVREH
VFTGAVSSLAWFMSAQESSFVTLGQRVLATPLKVRMHYGHPDVFDRLWFLTRGGISKASKVINISEDIFAGFNCTERGGN
ITHHEYIQVGKGRDVGLNQIALFEAKVASGNGEQILSRDVYRLGHRLDFFRMLSFYYTTVGFFISNMMVVLTVYSFLWGR
VYLALSGVEASIVDAKTLDNTSLTASLNQQFLVQMGLFTALPMIVENTLERGFGNAVWEFIVMQLQLSSVFFTFSMGTR

AHYFGRTILHGGAKYRATGRGFVVTHEKFAENYRLYSRSHFVKGLELIFLLIIYAAFGELSRTSSVYILITFSSWFLALTWIMA
PFIFNPSGFDWLKTVEDMEDFTQWIFFKEGGIGEGKLSWERWWDEEQAHLQSTGIIGKIAEIILDLRFLFFQYGIVYRLRIS
SGSRSIFVYLLSWIYVFAVGVFVKMITWGRDRYSAKAHAMYRLIQLFSAVFALLLIILLVQLTAFEFIDVLISMLAFIPTGWAL
IQIAQVLRPLLEKSGVWPTVVALARLYEFGMGIAVLVPVAVLSWLPGFQAMQTRILFNEAFSRGLQIKKLVTGKNPNAF---
---------------------------------------------------------------------------------------------------------------------------------
--------------------------------------------------------------

>AKXB 2068347 Phaeomegaceros coriaceus 1608

VKPFYQILQAEAASSNNGTAPHSAWRNYDDLNEYFWDPRCFTQLGWPLKLESNYFVEPTKGWNTTFYHFYVHVSLTFK
LLEFYQTNVVSHMRYLQWNATAQRVGKTGFVEQRSFWNIFRSFHRLWVILILFFQASIILSWNGGGPPWNELKNKDSLA
RVLSVFITWSGLRILQSLLDAGMESGLVTTETRLTGIRMTLKVLVATAWTITFTILYGQMWKQRHASQLGWSAAANAKLY
NFLYAAAVFILPEVLATLLFVIPWLRNFIETSNWRLFHVITWWFQSRLYVGRGLREGIVDNIRYTSFWIVILCSKFTFSYILQI
KPMIQPTKELLDFPDSAIIYRWHEFFRHGNRIAAICMWAPVVLIYFMDLQIWYSVYSSLVGALVGLFQHLGEIRNYQQLK
LRFEFFAKALQYVLIADEDYKSNSFWSSWWTKVKEGIKRFKLRYGFDTGDHYDKVNKKQIEENKFEHIWNEIITIFREEDLI
SDREVELLEVPITIQKAWNIENVIQWPLILIANEILVALGQCKDFQGGSDHTLWQKISKSEYRRCAVIEVYETIKQVILGIIAK
EKDEYKIITSLIVEIGTSLKLGNFTKKFQLSALPEIHSRIVSLVSSLMQYQRTESDSDGKRVVDAVQYLYDGLVRDFPISAWED
IRRQELSLYPLRDAENLLFVDAVELPGKEDDFYRHLKRLYTTLSTKESLLNVPQSLEARRRITFFSNSLFMTMPRAPQVEKM
LAFSVLTPYYDETVLFSKEQLRTENEDGVSTLFYLQKIYPDEWRHFAERMEKKNINVESPEIWDGEGLFELRLWASYRGQT
LARTVRGMMYYDRALQLLAFIDTAKQNDIDRVRNLLYKTSSGLASHLHASSSFLFRDGISPHGLHSGAEFYDKKIKEKHHA
TAAMKFTYVVTCQIYGIQKAKKEAVAADIETLMKRFAALRIAYVDVSPSGKEHYSVLSKYDGTTEKEVEIYRIQLPGPVKIG
EGKPENQNHAIIFTRGDAVQAIDMNQDNYFEEALKMRNLLQEFLQYYGIRKPTILGVREHVFTGSVSSLAWFMSAQESS
FVTLGQRVLATPLKVRMHYGHPDVFDRLWFLTRGGISKASRVINISEDIYAGFNCTLRGGNVTHHEYIQAGKGRDVGLN
QIALFEAKVASGNGEQVLSRDVYRLGHRLDFFRGLSFYFTTVGFFINNLIVVLTVYAFLWGRVYLALSGVEASIIGSNDDPIS
NLNSNALAASFNQQFVVQIGLFTALPMIVENSLEHGFTRAIWEFTTMQLQLASIFFTFSMGTRAHFFGRTILHGGAKYR
ATGRGFVVKHEKFAENYRLYSRSHFTKSLELVLLLVVYQVYGTVTKSTASYLLMTFSAWFLALSWLMAPFIFNPSGFDWLK
TVDDVDDFTNWIFFRGGILVKANQSWEAWWDEEYQHLHSAGLWAKLLEIILNFRFFFFQYGIVYRLKIAAGSTSIMVYLL
SWIYIVVVGVLQKFLSSAREKYAATTHRTYRGIQAAVIAGIVAVIILLLKFTHFVFMDVLVSLMAFLPTGWALIQIAQVLKQP
YLEGTPLWSTVVAVARLYEFGMGMLVLLPVACLSWLPGFQAMQTRILFNEAFSRGLQISRILVGKKP---------------------
---------------------------------------------------------------------------------------------------------------------------------
---------------------------------------------------------------------------------------------------------------------------------
--------------------------------------------------------------------

>ANON 2008762 Leiosporoceros dussii B 1935

KAKENWEKLVRKALRVSDDRGGGTGWARLFGAPSDEATPSLPAALGDTNINAVLAASNEIQKDNPHVARILCEHAYRLS
QNLDPKSQGRGVLQFKTGLLSIIKQKQTKKDGEKIDRSKDVDLIFKFYKFYKEKHRIDELEAQEEQWRQLGEQGDLVLKAK
DKERMASMRKAYETSRVLNEVLDALVKEASPEEVQRLFGDQQLKRDLERDAAKISQFKPYNIIPLETPGIADPVILLPEIKG
AHFAIKLQRGGELPWLPDSYPVPKQRPLDMFDLLHYFYGFQKDNVNNQREHVILLLANTQSRLDVPKDKADGLNIRLDD
ATISHLHDKVLENYNRWCSYLRKASVCKKGNSAQVQLLLTSLYFLIWGEAANVRFLPECICYIFHNMGHEFLQQLNRPTA
DRPARIADGNFEFLNTIIRPVYNVIAKEAKHGEKKSGFHSRWRTYDDLNEFFWSDHCFTLDWPWKLTDPFFVTPEAVDP
KASGDSKSVRPVKPPRRMGKTLFVEHRTAFHIYHSFHRLWIFFILMLQGLALFAFTSDLSFVHRLKVILSLGPSFVLFKFIQS
VLDVILMYGAYTSTRPQNIARIFVRFVWFAASMAFVTYLYVKVMLEDMVNSVTALSKIYLLVLGIYAGFQLIVSTVSHISTV

RLLADKFGSWGIVRFISWVHQERYYVGRGLYEKTEDYLVYVLFWIVLLAAKFSFSYFFQIKPMVGPSRIIVSTDILQYNWH
DFVSKSNHNALTLVALWAPVVMIYFLDTQVWYTVFSALWGGLDGARLRLGEIRSLSMLRKRFTTFPEEFVKTLMPPLART
GINYANGDGEQLNKKHKANALKFAPLWNEIIQNLRDEDYISNKERDWLLMPKNTGSLLLVQWPLFLLANKILLAVDLASE
HKGSQEELREKIFKDEYMGFAVQEAYFSVQRMFMKLVNDEAEQWVRGVFGEAEANVNRSSLLTSFVLKRLSGNGGLVS
MIAALAGLLEKEETPERVKSVTKALQDIFEVVMNDVISLELREKFETSEHLGNALQTGQLFSKISWPSAADEIDEIKRLNAL
LTVKDSSSNVPKNLEARRRLQFFSNSLFMKMPPTPPVRQMFSFSVFTPYYAEDVMFSYSALNKPNEDGITTLFYLQKIFPD
EWKNYLERVFRTEAQLERELETHLEKIIAEKRQTDTPVLLQLRLWASYRGQTLARTVRGMMYYRSALMLQSELEASVVTD
EEALSTRPVSGAMPASVMGQAVAGAKFTYVVSCQIYGEQKEKRLQQAEDIAFLLQTFEGLRVAYIDTVEKLADGKMNKE
YFSKLVKADATGKDQEIYSIKLPGQPKLGEGKPENQNHAIIFTRGDAIQTIDMNQDNYFEEAFKMRNLLQEFDVKHGLRK
PSILGVREHVFTGSVSSLAWFMSNQETSFVTLGQRVLAKPLKVRMHYGHPDVFDRIFHITRGGVSKASRVINISEDIFAGF
NTTLRQGNITHHEYIQVGKGRDVGLNQIALFEAKVSSGNGEQSLSRDVYRLGQLFDFFRMLSFFYTSVGFYVCTMMTVL
TVYVFLYGKAYLALSGVGKSLADSENNTALQTALNTQFLFQIGVFTAVPMIMGFILEQGVLKAFISFVTMQLQLASVFFTFS
LGTRTHYFGRTILHGGAKYRATGRGFVVQHISFAENYRLYSRSHFVKGLEVALLLVVYLAYGSAKGTVSYVLLTFSSWFLSLS
WIYAPYIFNPSGFEWQKTVEDFDNWTNWIFYKGGVGVKSDESWEAWWEDEQAHISSPRGRFWEIVLSLRFFIFQYGV
VYSLQATGTRTSLTVYGYSWLALVILLMIFKAFTFSQKASAKFQLLVRLGQGSLFLLLIPALVLAIIYTNLTVSDVFASLLALLPT
GWGILSICLACRPVVENLRLWKSVRAFARFYDAMMGFTIFVPIAVLSWFPFVSTFQTRLVFNQAFSRGLEISLILAGNRPN
----------------------------------------------

>ANON 2036486 Leiosporoceros dussii B 1756

FNILPLHNTLADHAAMRFPEVRAAIVALRAVGHLSRPPSHTEWRDGMDILDWLGSFFGFQADNIKNQREHLTLLLANA
QMRANPQQVDKMEGKQVSRIRRKLTKNYLEWCNFLEIIPAMRIVDGQGMIASDERLELLYTSLFLLIWGEAANLRFMPE
CLCYMYHHMCKELNNILDDRIDEKTGQSALPATCQPGGFMRLIVKPLYKIVSEEAKASEHGKAPYSAWRNYDDMNEFF
WNKSCFIKLGWPLDQSSNYLVSPQMKQGKQKVTKTGFVERRSFWSIFHSFDRLWTGLILFLQASIIVSWNGNGAPWTE
LKNKDQLARVLSIFITWSALRGLQAVLNMVMHYKLVTNENRTLGLRMVLKVAAAAAWIVVFSALWSSLWSLRKQEGVG
FDWTPKSNNRLRNVLYAAAAFLIPEALALLLFIIPWIRNFVENSNWRIFHLLTWWFQNRMYVARGLREGIFDNLRYSLFW
IVVVGTKFAFSYVFQIRPLIKPTKEILNLSSDEVHYLWHEYFGHGNRIAVLAVWAPVLFIYFMDLQIWYTVYSSLVGALVGL
FDHLGEIRNIGQIKQRFQYFASALQFALMAEDNLMQNTRSFWQGKLWTSIKNARARFQLRFGLIKNPGQIEAGRFMHI
WNEIMAIFREEDLVSDRELELLEVPPSSSWGVTVIQWPSFLLSNSILVALGQCENFHGDDKALWRKVSRNEYRRCAVVEC
FESACYLLLRIVSENTEEHTIIKLILDELKTAINLRTFTKKFQVKQISAVHGQLIKLASKILEWRDSKATKGQVVRALQDLYDTLI
RDFPQSSASYADLGQGSNLYPTRSGDLFFGSEDSVVLPERDEHAFYKQLKRVHTTLSTKEAILHVPKGLEAKRRITFFSNSL
FMTLPRAPAVDQMLAFSVLTPYYKETVLYQNKDLEEKNEDGITTIFYLQQIYPDDWANFLERMGKRGILPQKIEATDEGLF
ERRLWASYRGQTLARTVRGMMYYNRALQVLAFLDGASEKDVEQGHEFFRQASQGFGDSGDGIPMDEIRQHHSQSFT
DRVFSNHDKWVATAAMKFTYVAACQVYGDQKHKKEIAAGDIRYLMEKYDGLRVAYVDQKQVGKNAYEYYSVLVKYDQ
ATQREVEIYRVQLPGPVLVGEGKPENQNHAFIFTRGDAVQTIDMNQENYFEEALKMRNLLQEFTFNRGIRKPTILGVREH
VFTGAVSSLAWFMSAQETSFVTLGQRVLANPLKIRMHYGHPDVFDRLWFLTRGGISKASKVINISEDIFAGFNCTERRGN
VTHHEYIQVGKGRDVGIIQIYLFEAKVASGNGEQILSRDVYRLGHRLDFFRMLSFYYTTVGFFISNMLVVLTVYAFLWGRV
YLALSGVEASILKLDNSALTASINQQFLVQLGLFTALPMIVENSLEHGFGQAVWEFILMQLQLSSVFFTFSMGTRAHYFGR
TILHGGAKYRATGRGFVVTHEKFAENYRVFSRSHFVKALELVFLLVVYEVYGGLSRNSSVYVLVTFSSWFLALSWIMAPFLF
NPSGFDWLKTVEDVGNFSQWIFHKDGQYAEAKVSWERWWEEEQAHLQSTGIIGKIVEIILSLRFLFFQYGIVYRLRISDG
NRNFYVYLLSWTYVIAVGAFVKLITWAREKYSAKQHGTYRFIQLLSAIFVLTVIVLLAQLTKFSFSDVLSSLLAFLPTGWALLQ
IAQVLRHPFVEKSGAWPTVVALARLYEYGMGLAVLLPVAFLSWLPGFQAMQTRILFNEAFSRGLQIQRILTGKNP---------
--------------------------------------------------------------------------------------------------------------------------------------------
----------------------------------------------------------------------------

>ANON 2036504 Leiosporoceros dussii B 1786

PSGIYNLIPLHDNSTHPATKFPDSVKPGIKAAISALQYVSSLRQPPNVQWKDDMDLLDWLGAFFGFQKDNVRNQREHLV
LLLANKQMRLKPDHPEKLAPEVITQIRRKLTKNYTSWCKFLNQKSNIRNLGGKRLDVASLDERLELLYTSLYLLIWGEAANL
RFMPECLTFIFHHMAGELNNIIEHTIDETTKSPALPAYCEPHGFLNKVVTPLYQIIKIEAEASNDGKAPHSAWRNYDDMNE
YFWDRRCFSRLGWPLKLESNYLVGPPKTKGIIVQRVGKTGFVEQRSFWNIYRSFDRVWILLILVFQASIILAWNGGGPPW
TELKNKDSLARFLSVFITWAGLRILQSLLDAGMESSLVTPETRLIGIRMILKFLVATAWTIAFTILYGQVWKQRHAAGGWSP
AADAKLNNFLYSAAVFVLAEVLAMVLFIVPWIRSFLETSNWVICHLFTWWFQSRLYVGRGLREGIFDNLRYTLFWIVILAS
KFSFSYILQIKPMIQPTKEILAFPDSVIIYRWHEFFGHGNHIATACLWAPVVLIYVMDIQIWYAVYSALVGALVGLFQHLGEI
RNYQQLKLRFLFFAKALQYVLIADEDSTLYRSFWTVWWTKFTVGLNRFKLRYGLETDAYNALEGKRRTIEITKFEQIWNEII
TIFREEDLVSDREVELLEVPIPDNKWKIKVMQWPIILLANEILIALEQCKEFQGQDAALWQRICNSEYRKCAVIEAYEGVKH
MMRRIIIAGRYEEKRIINSLIEEIDTALESGMFIKKFQLSVLQDMYSRVVTLVSHLLDYQKTEIESDFKRVVDAVQYLYDGLV
RDFPVVTGSWEKLGEGTLYPLSDPEHLLFVDAVDLPDKEDGYFYRQIKRLHTILSTKESLLNVPSSLEARRRITFFSNSLFMT
MPRATKVENMLAFSVLTPYYNETVLFSKEQLSERNEDGVTTLFYLQKIYPDEWRHFLERMERKNLSETDVSEFREGLLHL
RLWASYRGQTLARTVRGMMYYDRALQMLAYLDTAKLPDIERVRNLLAKTSSGMASHLHGSGSFFLREGLSPHGHLSSSE
FYGHKQKQEYEATAAMKFTYVVTCQIYGLQRARKEAVAEDIQYLMKEFEALRVAYVDEQSSGPDKKYFSVLAKYDKSAEK
EVDIYRIQLPGPVKIGEGKPENQNHAIIFTRGEAVQAIDMNQDNYFEEALKMRNLLQEFTQYYGLRKPTILGVREHVFTG
SVSSLAWFMSAQESSFVTLGQRVLATPLKIRMHYGHPDVFDRLWFLTRGGISKASRVINISEDIYAGFNCTLRGGNVTHH
EYIQAGKGRDVGLNQIALFEAKVASGNGEQVLSRDVYRLGHRLDFFRMLSFYFTTVGFFVSNLVVVLTVYAFLWGRVYLA
LSGVEASIIGSNNDPISTINSNALAASFNQQFVVQIGLFTALPMIVENSLEHGFTTSIWEFITMQLQLASIFFTFSMGTRAH
FFGRTILHGGAKYRATGRGFVVKHEKFGENYRLYSRSHFVKGLELVLLLVVYQVYGTVSQSSTSYLLMTFSAWFLAITWIM
APFIFNPSGFDWLKTVDDVEDFTNWIFFRGGVLVSATQSWETWWDEEYEHLHSAGLWAKLLEILLNFRFFFFQFGIVYR
LNIAAGSTSILVYLLSWLYVLAVGVLQKFLSSAREKYAAKNHSTYRGIQAAIISGIVTVLIIFLKFTQFAFIDLLTSLMAFLPTG
WALIQIAQVLKKPYLGDSPVWSTVVAVARLYEFGMGMIVLVPVAFLSWLPGFQAMQTRILFNEAFSRGLQISRILVGKRP
HS----------------------------------------------------------------------------------------------------------------------------------------
--------------------------------------------------------

>BSNI 2012143 Anthoceros agrestis B 1932

KRAKENWDKLVRRALNASDAQSGWGRLLAPSSYVSTPALPSALGDKSIDAVLAASNEIKDDDPQVARILCEHAYRLSQKL
DPKSQGRGVLQFKTGLLSIIQQKHSKKDGKSIDRSNDVALIFEFYKNYRKNHKIDELEEQEEQWRQHGVQGDMVIKAED
KKRAASIRKAYETSKVLNEVLDSLIAEASPEEVERLFGDQQLKRDLERDAAKISEFKPYNIIPLESTGAADPVILLPEIKGAHY
AIKLQKDTEIPPLPDTFPRPKQRSLDMFDLLQYVFGFQKDNVNNQREHVLLLLANAQDRLGIPNDKVDLLAFQLDNKAIT
NLHDKVLENYMKWCSYLRKVSVCKRNNSASVQLLLTSLYLLIWGEASNVRFLPECICYIFHHMSTELLEQLNTKEAQRGR
GVTDKDTEFLETVVKPIYDVIAAEAKYSESNKSYSHSKWRTYDDLNEYFWSDHCFTLGWPWNPESSFFIKPGNATQVTD
GNGGQQKKTRMGKTLFVEHRTGFHIYHSFHRLWMFFLIFLQGLTIFAFTSELQWLHRVKIHLSCGPSFFILKFVQSVFDVV
LMYGAYTSTRGQNVARILVRFVWNSVASAFVLYLYVKVMLESNLNQGSALFNIYLLVLGVYAAFQLFVFTISRISALRMLA
DNYGSWSIIRFISWIHQERYYIGRGLYERTQDFLLYTLFWALILVAKFFFSYFFQIKPMVSPSRLIIQKRSIEYNWHDFLSQSN
HNALTLVALWAPVVMIYFVDTQVWYTVFSALWGGIHGAVLRLGEIRSLSMLRKRFDSFPVEFCSRLLPTEVQMSMDRA
WIAAGGGDTKKEELQRKKVHAAKFSPLWNEIIRNLREEDYISNRERDWLLMPSNSGSLQVVQWPLFLLANKVLLAVDM
AAEHKAAQYELEEKVWKDTYMGTAVTEAYFSLQRMLKRLINDEAAEWARVIFASIEEAIKNSALVNLLVLKKLSGPGGLVA
KIAALTGILEKEESPERVVNATTALLDIYEVVMNDVLSLDLREKLDRGQSREQIFTRLNWPSTPEECEEVRRLNLLLTAKDSA
SNIPKNLEARRRLEFFSNSLFMEMPSTPSVRQMFSFSVFTPYYAEEVIYGYTALNQPNEDGITVLFYLQKIFPDEWKNYLER

VSRTQNQLERELEWHLEKLKDKKDPVDTPELIQLRLWASYRGQTLARTVRGMMYYRNALILQTLLESSTAPDVEAQTTR
PVSGSLMTPAFLGQAIADMKFTYVVSCQIYGEQKQKRHPQAADIAYLLQVHEGLRVAYIDIVEKLSDGKIHREYFSKLVKA
DATGKDQEIYSIKLPGLPKLGEGKPENQNHAIIFTRGDAIQTIDMNQDNYFEEAFKMRNLLQEFDVQHGLRKPSILGVRE
HVFTGSVSSLAWFMSNQETSFVTLGQRILATPLRVRMHYGHPDVFDRIFHITRGGISKASRVINISEDIYAGFNSTLRRGNI
THHEYIQVGKGRDVGLNQIATFEAKVASGNGEQVLSRDIYRLGQLFDFFRMLSFFYTSVGFYICTMMTVLTVYIFLYGKAY
LALSGVGKALEEDITNNTALQTALNTQFLFQIGVFTAIPMIMGFILEQGVLRAVISFVTMQLQLSAVFFTFSLGTRTHYFGR
TILHGGAKYRATGRGFVVQHIAFAENYRLYSRSHFVKGLEVAILLIVYLAYGSSKGTVSYVLLTFSSWFLALSWLYAPYIFNPS
GFEWQKTVEDFDNWTNWIFYKGGIGVKSEESWEAWWEEEQSHITSPRGRFWEVVLSLRFFIFQYGVVYSLSATGTDTN
LNIYGYSWAVLIGLLLVFKVFTLSQKASAKFQLVVRLFQGTLFLILVTGVVVTIVETSVSVGDVFASLLALLPTGWGILSIAVA
VRPLLEGTGLWKSVRALARFYDALMGMMIFIPIAVLSWFPFVSTFQTRLVFNQAFSRGLEISLILAGNKPN-----------------
------------------------------

>BSNI 2053016 Anthoceros agrestis B 1788

FNVVPLHDCPNARHPATEFDEVKAATLALRTVGNLRQPPNVEWKDGMDLLDWLGAFFGFQKDNVRNQREHLVLLLA
NRQMRLKPEPNDKLAHEVVTQIRRKITKNYTEWCKFVNRKSNIRNLGGKRLDVASLDERLELLYTSLYLLIWGEAANLRF
MPECLAFIFHHMAGELNRIIEHNIDETTSQPALPAYCEPNGFLNNVVAPLYKIIQAESLSSNNGTAPHSAWRNYDDLNEYF
WDKRCFTQLGWPLKRQSNFLVEPPKGKFMTTQRVGKTGFVEQRSFWNIYRSFDRVWILFILVFQASILLCWNGGAAP
WIELKNKDSLARVLSIFITWGGLRILQTLLDAGMESSLVTMETRLVGIRMTLKFLLATAWTMTFAILYGQMWKQRHAAQ
GWSAAADRKLNNFLYAAAAFIFPEVLSLLLIVVPWFQNFLDTSNWKIFHLLTWWFQSRLYVGRGLREGLYDNLRYTAFW
VVILGSKFAFSYILQIKPMIEPTKELLKFPDSDIIYAWHEFFRHGNRIAVACMWAPVVLIYFMDIQIWYSVYSSLVGALVGLL
QHLGEIRNYQQLKLRFQYFAKALQYVLIADEEAMQYSWFSKTWTKVKEGAKRFSLRYGLAEDLYSKINKDTIEANKFEHI
WNEIIHIFREEDLVSDSEVALLEVPIPLVKEEKGEKGKSRNQTHKTWEISVMQWPLILIANEILIALGQAKDYRGGSDKTLW
QRICKSEYRRCAVIEVYESVKHMMRAIIIHEKEEHRIIVTLIAEIDASLKMGTFTKKFQLSELPEIHARVVTLVSNLMQFQKT
ESEDDSKKVIDAVQYLYDGLVRDFPLGTWEQLGEGTLYPLRNEDNPLFVDAVELPDKDDGFYRQVRRLHTTLSTKESLLN
VPRSLEARRRITFFSNSLFMTMPRAPTVEKMLAFSVLTPYYDETVLFSKEQLQQENEDGVSTLFYLQKIYPDEWRHFLER
MEKLDYSETQIWDKAKGLQDLRLWASYRGQTLARTVRGMMYYDRALQLQAFIDKAEQHDIDRVRNLLYKTSSGMASH
LHGSSSFLMREGVSPHGLHSSSEFYSQKAKQKHQATAAMKFTYVVTCQIYGIQKAKKEVVAEDIQYLMREFPPLRIAYVD
ETSPGSKENQYFSVLAKYDAAAGREVDIYRIQLPGPVKIGEGKPENQNHAIIFTRGDAVQAIDMNQDNYFEEALKMRNL
LQEFTVYYGLRKPTILGVREHVFTGSVSSLAWFMSAQESSFVTLGQRVLATPLKVRMHYGHPDVFDRLWFLTRGGISKAS
RVINISEDIYAGFNCTLRGGNVTHHEYIQAGKGRDVGLNQIALFEAKVASGNGEQVLSRDVYRLGHRLDFFRMLSFYYTT
VGFFINNMIVVLTVYAFLWGRVYLALSGVEASIIGSNNDPKSTINSDALAVSFNQQFVVQIGLFTALPMLVENSLEHGFTR
AVWEMITMQLQLASIFFTFSMGTRAHFFGRTILHGGAKYRATGRGFVVKHEKFAENYRLYSRSHFVKGLELVLLLVVYQV
YGTVSKSTASYLLMTFSAWFLAITWIMAPFIFNPSGFDWLKTVDDVEDFTNWIFFRGGILVKANQSWETWWEEEYQHL
HTAGLWAKLLEIILNFRFLFFQYGIVYRLKIAAGSTSIIVYLLSWIYVLVAGVLQKFLSSARDKYAAKTHRTYRSIQAAVIAGVV
AVFILMVKFTHFVFMDLIVSLMAFIPTGWALIQIAQVLKHPYLEGSPLWSTVVAVARLYEFGMGMLVLIPVAFLSWLPGF
QAMQTRILFNEAFSRGLQISRILVGKKP------------------------------------------------------------------------------------------
--------------------------------------------------------------------------------------------------

>DXOU 2038415 Nothoceros aenigmaticus 1589

RPAYCEPGGFLKKVVTPLYTIVSAESKASDHGKAPHSAWRNYDDMNEYFWNKRCLTRLGWPLSLSSNYFVQPQSAGSK
YRVGKTGFVEQRSFWNIYQSFDRLWIGLILFLQASIILAWNGDQAPWIELQNKDQIARVLSVFITWAGLRVLQAVLNMV

MNRKLVSAETLSLGVRMILKVVVAVAWTIAFAVLWSSLWNLRKKEGDGFDWTPNSNRRFRSMLYAGAVFIVPETLALLL
FILPWIRNFVENSQWRFFHLLTWWFQSRIYVARGLREGLVDNVRYTLFWIVTIVTKFAFSYALQIQPLIKPTKETIRLPKDA
VTYRWPELFGRGTRFAIVAMWAPVILIYMMDLQIWYSVYSSLVGALVGLFQHLGEIRNVEQLKERFQYFASALQFALMA
EDQFVEQKGFGRNLLVRAKNSVKNSYARFKLRYGFQDDLYKKDDRQIEAGRFSYIWNEILVIFREEDLISDSELELLEVPAK
AWNITVMQWPSVLLSNAILVALGLCKNFYGINDRALWKRVSRNDYRRCAVIECYESAQHVLLKIVAENTEEYNIVKAILDE
LNSSLGCGTFLKKFDLNQLPNIHKKVAELTSTLLGWSKKKQTRDNVVIALQNLIRDTLIRDFPKTSGTYEELGQGSVYPSRS
DGLLFTADNSILLPDPEDYAFYKQLKRLNTTLSTKEAILNVPQSLEARRRITFFSNSLFMTMPRAPQVDRMLAFSVLTPYFK
ETVLYENWELEKRNEDGVTTIFYLQQIFPDDWKNFEERMGGQGISKQEISTTERGLSERRLWASYRGQTLARTVRGMM
YYNRALEMLAFLDGASEVEVEQVQEMFLRTSAAGPSIRPMREISQHSSRHSSGRLLSIREKHRATAAMKFTYVAACQVY
GQQKADDRKPVTKRETTHPARDILYLMKTYEGLRVAYVDEKTVGRDAKEYYSVLVKYDQAMQREVEIYRVQLPGPLILGE
GKPENQNHALIFTRGDALQTIDMNQENYFEEALKMRNLLQEFTIHHGVRRPTILGVREHVFTGAVSSLAWFMSAQESSF
VTLGQRVLATPLKVRMHYGHPDVFDRLWFLSRGGISKASKVINISEDIFAGFNCTERGGNITHHEYIQVGKGRDVGLNQI
ALFEAKVASGNGEQILSRDIYRLGHRLDFFRMLSFYYTTVGFFISNMMVVLTVYSFLWGRVYLALSGVEASIVNAKTLDNA
SLTASLNQQFLVQMGLFTALPMIVENTLERGFGSAVWEFIVMQLQLSSVFFTFSMGTRAHYFGRTILHGGAKYRATGRG
FVVTHEKFAENYRLYSRSHFVKGLELILLLSVYAAFGELSRTSSVYILITFSSWFLALTWIMAPFIFNPSGFDWLKTVEDMED
FAQWIFFKEGGIGEGKLSWERWWDEEQAHLQSTGIVGKVAEIILDLRFLFFQYGIVYRLRISSGSHSIFVYLLSWIYVFVVG
VFVKMITWGRDRYSAKEHSVYRLIQLLSAVFALLFIILLVELTAFEFIDVLISMLAFLPTGWAVIQIAQVLRHPFLEKSGMWP
TVVALARLYEFGMGVAVLVPVAVLSWLPGFQAMQTRILFNEAFSRGLQIKKLVTGKSPNAF---------------------------------
-------------------------------------------------------------------------------------------------------------------------------------------
-------------------------------------------------------------------------------------------------------------------------------------------
----------------------------------------------------------------------------------

>FAJB 2008240 Paraphymatoceros hallii 1922

KARKNWEQLVRKALLRISDDSGATGWGRLFGPSYESTPALPSALGNTSIDAVLAASNEIQRESPQVARILCEHAYRLSQNL
DPKSQGRGVLQFKTGLLSIIKQKQSKKDGERIDRSNDVKLIFEFYRTYRKKLDELEAEEQKFRELGESVILKTEDKKRAATIKK
IYETSRVLNEVLDALMKEATPEDRESLLGDQQLKRDLERDAAKIADFKAYNIIPLETPTADPLLPEIKGAHFALKLGGEIPLSL
LPETRPRPNHNLRPLDMFDLLQYIFGFQEDNVSNQREHVILLLANTQARLSLPDDRAEEVRLDDGSIRHLHDKVLDNYKK
WCSYLRKDSVCKKGNSAPVQLLLTCLYLVVWGEAGNVRFLPECICYIFHNMAKELLLQLDKPVADRARGVGDGDHEFLD
QVINPIYNIIAAEAKHGQGKKGSHSQWRTYDDLNEYFWSDHCFTLSWPWSRDAPFFLKPSGVAEDSTPAVRRKQTRM
GKTLFVEHRTGFHIYHSFHRLWIFFILMLQGLGIFAFSTELQTVHRIKVILSLGPSFVVLKFLQSVFDVILMYGAYASTRGQN
VARIFVRFIWFAASSAFVSYLYVRVMSEQNLNQGSALFNIYLLVLGCYAGFQLIVSTISRISALRLQADKIGSWSIVRFISWV
HQERYYIGRGLYERTQDFLLYMLFWVVVLVAKFAFSYFFQIKPMVGPTRIIINTRIQQYNWHDFFSKSNHNALTLVALWSP
VVMIYFLDTQVWYTVFSALWGGLDGARLRLGEIRSLSMLRKRFNTFPQEFVNTLLPSVLKLKINTSTPIGQLEQKEKVNAL
KFAPLWNEIILNLRLEDYISNKEKDWLWMPKNTGSLQLVQWPLFLLANKILLAVDLAAEHKGSQFELQEKIFKDEYMGFA
VQEAYFSVQRMLMKLVNNEAELWVKGIFADVEGSIKEQALATSIVLKKLSGPSGLVAKLATLTGILEKEINSERSKMATNAL
QDIFEVVMKDVLSLNLREKFESWPVLEKARRTGHLFSDLNWPSSTEENEEIKRLNSLLTVKDSASNIPKNLEARRRLQFFS
NSLFMDMPATPSVRQMFSFSVFTPYYTEDVMYSYGALNKPNEDGITILFYLQKIFPDEWKNYLERVFRTEATLERELDYHL
QKLKDKKEDVADTYELLQLRLWASYRGQTLARTVRGMMYYRSALILQSQLEASGPPDLEAATRPVSGALPPSLVGQSIAD
MKFTYVVSCQIYGKQKQQRHQQAADIAYLLQTHEGLRVAYIDTIEKIVDGKTSSEYFSKLVKADATGKDQEIYSIRLPGNPL
LGEGKPENQNHAIIFTRGDCIQTIDMNQDNYFEEAFKMRNLLQEFDVQHGLRKPSILGVREHVFTGSVSSLAWFMSNQ
ETSFVTLGQRVLAKPLKVRMHYGHPDVFDRIFHITRGGISKASKVINISEDIYAGFNSTLRQGNVTHHEYIQVGKGRDVGL
NQIALFEAKVASGNGEQVLSRDIYRLGQLFDFFRMLSFFYTSIGFYVCTMMTVLTVYVFLYGKAYLALSGVGAELEKDVVN
STALQTALNTQFLFQIGVFTAIPMIMGFILEQGVLKAFISFVTMQLQLASVFFTFSLGTRTHYFGRTILHGGAKYRATGRGF

VVQHIPFAENYRLYSRSHFVKGLEVALLLIVYGAYGSNKGTVSYVLLTFSSWFLSISWLYAPYIFNPSGFEWQKAVEDFDKW
TNWIFYKGGVGVNSDESWEAWWEEEQAHITSPRGRFWEVVLSLRFFIFQYGVVYSLQATGTNTSLNVYGISWLALVGLL
IVFKAFTFSQKASAKFQLVVRLGQGVLFLLLIAGIVLSIVYTKLSLGDVFASLLALLPTGWGILSICLAIRPAVEKLRLWKSVRA
FARFYDALMGMVIFVPIAVLSWFPFVSTFQTRLVFNQAFSRGLEISLILAGNKPN--------------------------------------------
-------------

>FAJB 2010681 Paraphymatoceros hallii 1632

IWGEAANLRFMPECLAFIFHNMAGELNKIIEHQLDQSTSEPAKPAYCEPLGFLNNVVKPLYQILQAESASSRNGTAPHSA
WRNYDDLNEYFWDPRCFTQLGWPLKLESKYLVEPPKGRLNMTTSRVGKTGFVEQRSFWNIYRSFDRVWIILILFLQASIIL
SWNGGGPPWTELKNKDSLARVLSVFITWGGLRILQSLLDAGMASGLVTSETRLIGIRMTLKLLVATAWTITFIVLYGQMW
KQRHSSRSRFWSAAADAKLYNFMYAAAVFILPEVLATLLFAVPWFRNFIENSNWRVFHFLTWWFQSRLYVGRGLREGIT
DNVRYTSFWIVILGSKFTFSYILQIKPMIEPTKQLLAFPDAVIIYRWHEFFRHGNRIAAACMWAPVILIYFMDIQIWYAVYS
SLVGALVGLFQHLGEIRNYQQLKLRFQFFASALQYVLIADESYTGYSFWTRWWRKVKEGVKRFQLRYGDDPYSKLDRNIK
EANKFEQIWNEIITIFREEDLISDSEVELLEVPIPIQKAWKISVMQWPLILIANEILVALGQCKNFHGSDQMLWQRIIKSEYR
KCAVMEVYESIKQMLLRIISEDSDEYKIMTSLIAEIDNSLSSGTFTKKFQLSALPEIHSRIVSLVSNLMQYEHTESESDEKRVV
DAVQYLYDGLVRDFPVTTWEDLGRQELSLYPLRDAENLLFVDAVELPRKEDSIYRDFYRQLKRLHTTLSMKESLLNVPQSL
EARRRITFFSNSLFMTMPRAPQVEKMLAFSVLTPYYDETVIFSKEQLWTENEDGVFTLFYLQKIYPDEWRHFAERMEKRN
LNINDIHIWEGDDLRELRLWASYRGQTLARTVRGMMYYDRALQLLAFIDTAEQSDIDRVRNLLYKTSSGMASHLHGTTS
FLLREGISPHGLHSSAEFYDKKIKDKHQATAAMKFTYVVTCQIYGIQKAKNEAVAHDIQYLMKEFPALRVAYVDVAQSGKE
HFSVLSKYDSSLEKEVEIYRIQLPGPVKIGEGKPENQNHAIIFTRGDAVQAIDMNQDNYFEEALKMRNLLQEFTQYYGIRK
PTILGVREHVFTGSVSSLAWFMSAQESSFVTLGQRVLATPLKVRMHYGHPDVFDRLWFLTRGGISKASKVINISEDIYAGF
NCTLRGGNVTHHEYIQAGKGRDVGLNQIALFEAKVASGNGEQVLSRDVYRLGHRLDFFRMLSFYFTTVGFFINNLIVVLT
VYAFLWGRVYLALSGVEASIIGSNNDPISTLNSDSLAASFNQQFVVQIGLFTALPMIVENSLEHGFTTAIWEFITMQLQLA
SIFFTFSMGTRAHFFGRTILHGGAKYRATGRGFVVKHEKFAENYRLYSRSHFVKGIELVLLLVVYQVYGTVSKSTASYLLMTF
SSWFLAITWLMAPFIFNPSGFDWLKTVDDVEDFTNWIFFRGGILVKANQSWEAWWDEEYQHLHSAGLWAKLLEIILNF
RFFFFQYGIVYRLKVAAGSTSILVYLLSWIYVVIVGVLQKFLSSARDKYAATTHRTYRSIQAAVIAGIVTVLILLLKFTHFVFLD
VLVSLMAFLPTGWALIQIAQVLKKPYLEGSPLWSTVVAVARLYEFGMGMLVMLPVACLSWLPGFQAMQTRILFNEAFSR
GLQISRILVGTKKP------------------------------------------------------------------------------------------------------------
-----------------------------------------------------------------------------------------------------------------------------------
----------------------------------------------------------------------------------------

>FAJB 2012507 Paraphymatoceros hallii 1767

YNIVPVHDTLADHAALQFPEVRAAIMALQTVGSLARPPYTEWRDGMDLLDWLGAFFGFQADNINNQREHLALLLANG
QMRANPDPIDKLSSKVVSSIRKKVTKNYVDWCRFLEIPINMSIIDSKYTSNNERLELLYTGLYLLIWGEAANLRFMPECLCF
LFHNMAWELNRILEDRIDEGTAQPARPAYCEPGGFLKRVVTPLYKVLSAESKASEHGKAPHSAWRNYDDMNEYFWDKR
CLTRLGWPLSLSSTYFVEPQAAGSRHRVGKTGFVEQRSFWNIYQSFDRLWIGLILFFQASIILAWNGDKAPWTELQNKD
QLARILSIFITWAGLRVLQAVLNLLMQYKLVSAETLSLGVRMLLKVIVAVAWTIAFAVLWSSLWNLRLKEGEGFDWTPESN
RRFRNILYAGAVFIFPEALALLLFIIPWVRNFVENSHWKAFHLLTWWFQTRIYVARGLREGLLDNIRYTLFWVIVIVTKFAFS
YTLQIRPLIKPTKETINLPSNAVTYRWPEIFGRGTRFAIVAMWAPVVLIYMMDLQIWYSVYSSFAGALVGLFQHLGEIRNV
EQLKQRFQYFASALQFALMAEDQFVSQKRFGHSWWISIKNSYTRFKLRYGFEDDSYRKDERQIEAGRFSYIWNEIMAIFR
EEDLISDRELELLEVPAKAWNISVMQWPSVLLSNAILVALGQCKNFYGNDKALWRRVARNEYRRCAVIECYESAQHVLLR

IVAENTEEYLMIKAILDELNLSLRLGTFSKKFDLTQLPNIHKRVAALTSSLLKWRKQQNSREQVVLALQDLYDTLIRDFPRAS
GTYAELGQGSSVYPSKNSGLLFASDESVQLPDPEDLAFYKQLQRFHTTLSTKEAILTVPQSLEARRRITFFSNSLFMTMPRA
PQVDRMLAFSVLTPYYKETVVYENWELEKRNEDGVSTIFYLQQIFPDDWNNFEERMERQGILKDEIHRTDEGLYERRLW
ASYRGQTLARTVRGMMYYSRALELLAFLDGASEVEVEQMQEMFLRTSNSSAGPSVRPMMEIGQHSSRHSSGRLRSNR
DKHRATAAMKFTYVAACQVYGQQKADEKKHDTKRESTHPARDILYLMKTYEGLRVAYVDEKSVGRDAKEYYSVLVKYDH
ATQSEVEIYRVQLPGPLILGEGKPENQNHAFIFTRGDAVQTIDMNQENYFEEALKMRNLLQEFTVQYGLRKPTILGVREH
VFTGAVSSLAWFMSAQESSFVTLGQRVLATPLKVRMHYGHPDVFDRLWFLTRGGISKASKVINISEDIFAGFNCTERGGN
VTHHEYIQVGKGRDVGLNQIALFEAKVASGNGEQILSRDVYRLGHRLDFFRMLSFYYTTVGFFISNMMVVLTVYSFLWG
RCYLALSGVEASIVSAKTLDNASLTASLNQQFLVQMGLFTALPMIVENTLERGFGNAVWEFVVMQLQLSSVFFTFSMGT
RAHFFGRTVLHGGAKYRATGRGFVVTHEKFAENYRLYSRSHFVKGLELIFLLIIYGAFGELSRTSSVYILITFSSWFMALTWI
MAPFIFNPSGFDWLKTVEDMEDFAHWIFFKEGGISDAKISWERWWDEEQAHLQGTGTLGKLAEIILDLRFLFFQYGIVY
RLRISSGSRSILVYLLSWIYVLVVGAFVKVITWARVRYSAKEHAIYRLIQLFCAIFCLLLIILLVQLTDFAFIDVFISMLAFIPTGW
AVIQIAQVLRHPFLEKSGLWPTVVALARLYEFGMGVAVLVPVAVLSWLPGFQAMQTRILFNEAFSRGLHFKQIVTGRNP--
---------------------------------------------------------------------------------------------------------------------------------------
-----------------------------------------------------------------------

>RXRQ 2016252 Phaeoceros carolinianus 1937

RARANWDVLVEKKLRAGIAQARPGAGGGLLQPISLAIGNARVDTVLSVADRLRKYSQQVARICCEYAYRLAQDLDPNSQ
NRGMLQLKTGLLSIITQKKSKREGQAIDRRNDAKFIIDFYKTYRKKIDKLEALEEQWHERTNQGDGAPAAESKKRVAKIKE
GYLTFRILNEVLDALIPDRTEKKALLAADQQEQGQGRSDIESDAARASEYKSYNILPLETLNATHLFEFLPQITGAQSALYLG
NRDRGRQQDHLPELPRTVPRPNQRSEDMLDLLQYIFGFQKDNVNNQREHIILLLANTHARVSHPDGQANDLAVQRM
HDKVLENYQRWCTYLRKESACRRGNSVQVQLLLTALYLLIWGEAGNIRFLPECLCYIFHNMAFELLQQLDNTVAVRARGI
GDGDHEFLEQVIKPLYGVVAAEAKYGKGKTHTDWRNYDDLNEYFWSDHCFTLGWPMLLNQPFFTKPSEEPARRWGG
QRRRMGKTLFAEHRTSFHIYHSFHRLWIFFLIMLQILVFIAFTSKLDGWHRARLILSFGPSYVSLKLFQSLLDFWMMLGAY
KSTRGQNIARVFVRFLWFLALSSGVIFLYVMVMREADLNKTSALLNIYILVIGVYTAVEVATGIISRISAVRLQVDKFETWGI
VRFISWVHQERFYVGRGMYESTKDCAIYVFFWVVILAAKFSFSYFFQIQPLVNPTRVIYSKKITEYNWFSFFSKDHHNMW
TLGSLWGPVVLIYFLDTQVWYTVFSAIWGGIDGARMHLGEIRSLNMLSNRFRSFPEEFVKKLTPAAIRSQITSGAQSGNS
DAQRFAPIWNEVIRNLRDEDYISNRERDLLLMPKNNGSLQEVQWPLFLLANKIALATNLSGEHKGAQQELEAEISDDDY
MLSAVKEAYASVRQMLLTLVQDPVEQTWVRNVFQLIDEAKAEKALVGDLNLKKLGGPDGLVSKLGVLTKKLKKLKGISEE
KIAKKHNKITETLARIFETVMRDVLGLQLREKFISSDIMRRAREEPQLLFSDINWLSTVEKHEEIVRLHELLTTKVSASSVPK
NDEARRRLEFFSNSLFMGMPATPSLQKMFSFSVLTPYYEEDVMFSYAALNKPNEDGITILFYLQKIFPDEWNNYLERVFH
KESQKKHADLEKHFADYVKLRRKNLKDKEEKVDTHEEGPILQLRLWASFRGQTLARTVRGMMYYRSALVLLSELEDPPAR
DEEAGSRPALGAMPPSLRAQSVADMKFSYVVSCQIYGKLKKSEGVAELQKAADIGYLLRSYEGLRVAYIHSEEITVGNAPT
KLFFSKLVKADAVGMEQEIYSIKLPGDPLLGEGKPENQNHAIVFTRGDAIQTIDMNQDNYFEETVKMRNLLQEFDVHHG
LRKPSILGVREHVFTGSVSSLGWFMSNQETSFVTLGQRVLAKPLRVRMHYGHPDVFDRIFHISRGGISKASKVINISEDIYA
GFNSTLRQGNVTHHEYIQVGKGRDVGLNQIAIFEGKVAGGNGEQVLSRDIYRLGQLFDPVRMLSFFYTSVGFYICTMMT
VLTVYVFLYGKAYLALSGVETTLTGEETNNKALQTALNTQFLFQIGVFTAVPMIMGAILEQGVLKAFTGFVTMQLQAASVF
FTFSLGTRTHYFGRTILHGGAKYRSTGRGFVVQHISFAENYRLFSRSHFVKGFEIALLLIVYAAYGSSKGTVSYILLTFSSWFLA
LSWLYAPFLFNPSGFEWQKAVEDFDKWSNWMFYKGGVGVKLDESWEAWWEDEMSHITSWWGRFWEIVFSLRFLIF
QYGVVYSLNVTGKDNSLYVYGYSWAALVGLLVLWKVFTIGQRAAANAQLLVRAFQTVILVGILTSLVVLIIRTSLTLADVFAA
FFLALLPTGWLILSICVALKPVVKPLGLWESVRSLARSYDALFGMLIFVPIAVLSWFPFVSTFQTRLVFNQAFSRGLEISLILS
GKQEDES------------------------------------------

>RXRQ 2024670 Phaeoceros carolinianus sporophyte 1980

KARKNWEQLVRKALLRISDDSGGTGWGRLFGPSSYESTPALPSALGNTSIDAVLAASNEIQKESPQVARILCEHAYRLSQN
LDPKSQGRGVLQFKTGLLSIIKQKQSKKDGERIDRSNDVKLIFEFYRTYRKKLDELEAEEKKFRDLGDSVILKADDKKRAASI
KKMYETSRVLNDVLDALMKEASPEDKESLQRESDQQLKKDLERDAAKIADFKAYNIIPLETPTADFVLLPEIKGAHFALKLG
GEVPATLLPETYPRPKQDLRPLDMFDLLQYIFGFQEDNVSNQREHVILLLANTQARLLPPDDRVDGFSVRLDDGSIRHLH
DKVLDNYKKWCSYLRKDSVCRKGSSVAIQLLLTCLYLVIWGEAGNVRFLPECICYIFHHMAKELLQQLGEPVAGRSRGVG
DGDHEFLDQVISPIYSVIAAEAKHGQGKKGSHSQWRTYDDLNEYFWSDHCFTLGWPWNRDAPFFLKPSVVKQDGVD
NPPRRKQRMGKTLFVEHRTGFHIYHSFHRLWIFFILMLQGLGIFAFSTELQTGHRMKVILSLGPSFVVLKFLQCSGLVLAG
GKIPCSRLVNPKRLFSIACTGLKLLVIGKGSQSLSASTVVLNLSLQLYLDFGVWSLGQHSGQNVARIFVRFIWFATSSTFVTY
LYVRVMSQPNLNQGSSLFNIYLLVLGCYAGFQLIVSTISRISALRIQADKIGSWSIVRFISWVHQERYYIGRGLYERTLDFFFY
MLFWVVVLVAKFSFSYFFQIKPMVGPTRIIINTRIQAYNWHDFFSQSNHNALTLVALWAPVVMIYFLDTQVWYTVFSAL
WGGLDGARLRLGEIRSLSMLRKRFNTFPQEFVNTLLPSVVKSKINTSIPTGLLDQTEKVNALKFAPLWNEMILNLRLEDYIS
NKEKDWLWMPKNTGSLQLVQWPLFLLANKILLAVDLAAEHKGSQFELQDKIFKDEYMGFAVQEAYFSVQRMLKNLVK
NEAELWVKGIFADVEESITESALATSIVLKKLSGPGGLIAKLANLTGILEKEFNSERSKLATNALQDIFEVVMKDVLSLSLREK
FESWSVLERARRTGLFFSDLNWPSSPEENEEIKRLNSLLTVKDSASNIPRNLEAQRRLQFFSNSLFMDMPATPSVRQMFS
FSVFTPYYTEDVMYSYGALNKPNEDGITILFYLQKIFPDEWKNYLERVFRTEATLERELDRHLEKLKDKKEDVADTDVLLQL
RLWASYRGQTLARTVRGMMYYRSALILQSQLEGSGPPDLEAATRPVSGALPPSLIGQSIADMKFTYVVSCQIYGKQKQQ
RQQQAADIAYLLQTHEGLRVAYIDTVEKIVDGKTSSEYFSKLVKADATGKDQEIYSIRLPGNPLLGEGKPENQNHAIIFTRG
DCIQTIDMNQDNYFEEAFKMRNLLQEFDVQHGLRKPSILGVREHVFTGSVSSLAWFMSNQETSFVTLGQRVLAKPLKV
RMHYGHPDVFDRIFHITRGGISKASKVINISEDIYAGFNSTLRQGNVTHHEYIQVGKGRDVGLNQIALFEAKVASGNGEQ
VLSRDIYRLGQLFDFFRMLSFFYTSIGFYVCTMMTVLTVYVFLYGKAYLALSGVGAELEKDVVNSTALQTALNTQFLFQIGV
FTAIPMIMGFILEQGVLKAFISFVTMQLQLASVFFTFSLGTRTHYFGRTILHGGAKYRATGRGFVVQHIPFAENYRLYSRSH
FVKGLEVALLLIVYGAYGSNKGTVSYVLLTFSSWFLSISWLYAPYIFNPSGFEWQKAVEDFDKWTNWIFYKGGVGVNSDES
WEAWWEEEQAHITSPRGRFWEVVLSLRFFIFQYGVVYSLTATGTNTSLNVYGISWLALVGLLIVFKAFTFSQKASAKFQLV
VRLGQGVLFILLIAGIVLSIVYTDLSLGDVFASLLALLPTGWGILSICLAIRPVVEKLRLWKSVRAFARFYDALMGMLIFVPIA
VLSWFPFVSTFQTRLVFNQAFSRGLEISLILAGNKPN

>RXRQ 2025306 Phaeoceros carolinianus 1768

YNIVPVHDTLADHAALQFPEVRAAIVALQTVGSLARPPYLEWRDGMDLLDWLGAFFGFQADNINNQREHLALLLANG
QMRANPDPIDKLSSKVVSSIRKKVTKNYVDWCRFLEIPINMSIVDSKYTTSSNERLELLYTGLYLLIWGEAANLRFMPECLC
FLFHNMAWELNRILEDRIDEGTAQPAKPAYCVPGGFLKSVVTPLYKVLSAESKASEHGKAPHSAWRNYDDMNEYFWDK
RCLTKLGWPLSLSSNYFVEPQAKVPHRVGKTGFVEQRSFWNIYQSFDRLWIGLILFLQASIILAWNGDKAPWIELQNKDQ
LARILSIFITWAALRVLQAVLNILMQHKLVSAETLSLGVRMLLKVVVAVAWTIAFAVLWSSLWNLRLKEGIGFEWTPESNK
RFRNMLYAGAVFIFPEALALLLFIIPWVRNFVENSQWKVFHLLTWWFQTRIYVARGLREGLYDNIRYSLFWIIVIVTKFTFS
YTLQIRPLIKPTKETINLKSDEVHYRWHELFGRGTRFAIVAMWAPVIMIYMMDLQIWYSVYSSFTGALVGLFEHLGEIRNV
EQLKQRFQYFASALQFALMAEDQFVKPKGFGRSWWNSIVNSYTRFKLRYGFGDDSYGKEDRQIEAGRFSYIWNEIMAIF
REEDLISDRELELLEVPAKAWNISVMQWPSVLLSNAILVALGQCKNFYGNDKALWRRVGRNEYRRCAVIECYESAQHVLL
RIVAENTEECIMIKAILDELNLSLRLGTFSKKFDLTQLPNIHKRVAALTSVLLKWHKQQNGREQVVLALQDLYDTLIRDFPRT
SGTYAELGRGSSVYPSRSGGLLFASDESVQLPDPEDLAFYKQLKRFNTTLSTKEAILTVPHSLEARRRITFFSNSLFMTMPR
APQVDRMLAFSVLTPYYKETVLYENWELEKRNEDGVSTIYYLQQIFPEDWNNFEERMERRGISKDEIDRTEEGLFERRLW
ASYRGQTLARTVRGMMYYSRALELLAFLDSASEVEVEKVQEMFLRTSSSTAGPSVRPMREISQHSSRHSSGRLHSNRDK

HRATASMKFTYVAACQVYGQQKADEKKPGTKKESTHPARDILYLMKTYGGLRVAYVDEKPVGRDEKEYYSVLVKYDHDT
QSEVEIYRVKLPGPLILGEGKPENQNHAFIFTRGDAVQTIDMNQENYFEEALKMRNLLQEFTVKYGLRKPTILGVREHVFT
GAVSSLAWFMSAQESSFVTLGQRVLANPLKVRMHYGHPDVFDRLWFLTRGGISKASKVINISEDIFAGFNCTERGGNIT
HHEYIQVGKGRDVGLNQIALFEAKVASGNGEQILSRDVYRLGHRLDFFRMLSFYYTTVGFFISNMMVVLTVYSFLWGRC
YLALSGVEASIVSAKTLDNASLTASLNQQFLVQMGLFTALPMIVENTLERGFGNAVWEFVVMQLQLSSVFFTFSMGTRA
HFFGRTVLHGGAKYRATGRGFVVTHEKFAENYRLYSRSHFVKGLELIFLLIIYGSFGELSKTSSVYILITFSSWFLALTWIMAP
FIFNPSGFDWLKTVEDMEDFANWIFFKEGGISDAKISWERWWDEEQAHLQGTGILGKIAEVILDLRFLFFQYGIVYRLRIS
AGSRSILVYLLSWIYVFVVGAFVKVITWARVRYSAKEHSIYRLIQLFTAVFSLLLIILLVQLTDFAFIDVLLSMLAFIPTGWAVIQ
IAQVLRPWLEKSPLWQTVVALARLYEFGMGVAVLVPVAVLSWLPGFQAMQTRILFNEAFSRGLHFKQIVTGRNPNS-----
--------------------------------------------------------------------------------------------------------------------------------------------
----------------------------------------------------------------------

>TWUW 2052586 Anthoceros agrestis A 1788

FNVVPLHDCPNARHPATEFDEVKAATLALRTVGNLRQPPNVEWKDGMDLLDWLGAFFGFQKDNVRNQREHLVLLLA
NRQMRLKPEPNDKLAHEVVTQIRRKITKNYTEWCKFVNRKSNIRNLGGKRLDVASLDERLELLYTSLYLLIWGEAANLRF
MPECLAFIFHHMAGELNRIIEHNIDETTSQPALPAYCEPNGFLNNVVAPLYKIIQAESLSSNNGTAPHSAWRNYDDLNEYF
WDKRCFTQLGWPLKRQSNFLVEPPKGKFMTTQRVGKTGFVEQRSFWNIYRSFDRVWILFILVFQASILLCWNGGAAP
WIELKNKDSLARVLSIFITWGGLRILQTLLDAGMESSLVTMETRLVGIRMTLKFLLATAWTMTFAILYGQMWKQRHAAQ
GWSAAADRKLNNFLYAAAAFIFPEVLSLLLIVVPWFQNFLDTSNWKIFHLLTWWFQSRLYVGRGLREGLYDNLRYTAFW
VVILGSKFAFSYILQIKPMIEPTKELLKFPDSDIIYAWHEFFRHGNRIAVACMWAPVVLIYFMDIQIWYSVYSSLVGALVGLL
QHLGEIRNYQQLKLRFQYFAKALQYVLIADEEAMQYSWFSKTWTKVKEGAKRFSLRYGLAEDLYSKINKDTIEANKFEHI
WNEIIHIFREEDLVSDSEVALLEVPIPLVKEEKGEKGKSRNQTHKTWEISVMQWPLILIANEILIALGQAKDYRGGSDKTLW
QRICKSEYRRCAVIEVYESVKHMMRAIIIHEKEEHRIIVTLIAEIDASLKMGTFTKKFQLSELPEIHARVVTLVSNLMQFQKT
ESEDDSKKVIDAVQYLYDGLVRDFPLGTWEQLGEGTLYPLRNEDNPLFVDAVELPDKDDGFYRQVRRLHTTLSTKESLLN
VPRSLEARRRITFFSNSLFMTMPRAPTVEKMLAFSVLTPYYDETVLFSKEQLQQENEDGVSTLFYLQKIYPDEWRHFLER
MEKLDYSETQIWDKAKGLQDLRLWASYRGQTLARTVRGMMYYDRALQLQAFIDKAEQHDIDRVRNLLYKTSSGMASH
LHGSSSFLMREGVSPHGLHSSSEFYSQKAKQKHQATAAMKFTYVVTCQIYGIQKAKKEVVAEDIQYLMREFPPLRIAYVD
ETSPGSKENQYFSVLAKYDAAAGREVDIYRIQLPGPVKIGEGKPENQNHAIIFTRGDAVQAIDMNQDNYFEEALKMRNL
LQEFTVYYGLRKPTILGVREHVFTGSVSSLAWFMSAQESSFVTLGQRVLATPLKVRMHYGHPDVFDRLWFLTRGGISKAS
RVINISEDIYAGFNCTLRGGNVTHHEYIQAGKGRDVGLNQIALFEAKVASGNGEQVLSRDVYRLGHRLDFFRMLSFYYTT
VGFFINNMIVVLTVYAFLWGRVYLALSGVEASIIGSNNDPKSTINSDALAVSFNQQFVVQIGLFTALPMLVENSLEHGFTR
AVWEMITMQLQLASIFFTFSMGTRAHFFGRTILHGGAKYRATGRGFVVKHEKFAENYRLYSRSHFVKGLELVLLLVVYQV
YGTVSKSTASYLLMTFSAWFLAITWIMAPFIFNPSGFDWLKTVDDVEDFTNWIFFRGGILVKANQSWETWWEEEYQHL
HTAGLWAKLLEIILNFRFLFFQYGIVYRLKIAAGSTSIIVYLLSWIYVLVAGVLQKFLSSARDKYAAKTHRTYRSIQAAVIAGVV
AVFILMVKFTHFVFMDLIVSLMAFIPTGWALIQIAQVLKHPYLEGSPLWSTVVAVARLYEFGMGMLVLIPVAFLSWLPGF
QAMQTRILFNEAFSRGLQISRILVGKKP------------------------------------------------------------------------------------------
--------------------------------------------------------------------------------------------------

>TWUW 2052598 Anthoceros agrestis A 1937

RARANWDVLVERKLRAGIAQARPGAGGGLLQPISLAIGNARVDTVLSVADRLRKYSQQVARICCEYAYRLAQDLDPNSQ
NRGMLQLKTGLLSIITQKKSKREGQAIDRRNDAKFIIDFYKTYRKKIDKLEALEEQWHERTNQGDVAPAAESKKRVAKIKE

GYLTFRILNEVLDALIPDRTEKKALLAADQQEQGQGRSDIESDAARASEYKSYNILPLETLNATHLFEFLPQITGAQSALYLG
NGDRGRQQDHLPELPRTVPRPNQRSEDMLDLLQYIFGFQKDNVNNQREHIILLLANTHARVSHPDGQANDLAVQRM
HDKVLENYQRWCNYLRKESACRRGNSVQVQLLLTALYLLIWGEAGNIRFLPECLCYIFHNMAFELLQQLDNTVAVRARGI
GDGDHEFLDQVIKPLYSVVAAEAKYGKGKTHTDWRNYDDLNEYFWSDHCFTLGWPMLLNQPFFTKPSEEPARRWGG
QRRRMGKTLFAEHRTSFHIYHSFHRLWIFFLIMLQILVVIAFTSKLDGWHRARLILSFGPSYVSLKLFQSLLDFWMMLGAY
KSTRGQNIARVFVRFLWFLALSIGVIFLYVMVMREADLNKTSALLNIYILVIGVYTAVEVATGIISRISAVRLQVDKFETWGIV
RFISWVHQERFYVGRGMYESTKDCVIYVFFWVVILAAKFSFSYFFQIQPLVNPTRVIYSKKITEYNWFSFLSKDHHNMWT
LGSLWGPVVLIYFLDTQVWYTVFSAIWGGIDGARMHLGEIRSLNMLSNRFRSFPEEFVKKLTPAAIRSQITSGAQSGNSD
AQRFAPIWNEVIRNLRVEDYISNRERDLLLMPKNNGSLQEVQWPLFLLANKIALATNLSGEHKGAQQELEAEISDDYYML
SAVKEAYASVRQMLLTLVQDPVEQTWVRNVFQLIDEAKAEKALVGDLNLKKLGDPDGLVSKLGVLTKKLKKLKGISEEKIA
KKHNKITETLRRIFETVMRDVLGLQLREKFISSDIMRRAREEPQLLFSNINWLSTVEKHEEIVRLHELLTIKVSASSVPKNDE
ARRRLEFFSNSLFMGMPATPSLQKMFSFSVLTPYYEEDVMFSYAALNKPNEDGITILFYLQKIFPDEWNNYLERVFHKESQ
KKHADLEKHFADCVKVRRKNLKDKEEKVDTHEEGPILQLRLWASYRGQTLARTVRGMMYYRSALVLLSELEDPPARDEE
AGSRPALGAMTPSLRAQSVADMKFSYVVSCQIYGKLKKSEGVAELQKAADIGYLLRSYEGLRVAYIHSEEITVGNAPTKLFF
SKLVKADAVGMEQEIYSIKLPGDPLLGEGKPENQNHAIVFTRGDAIQTIDMNQDNYFEETVKMRNLLQEFDVHHGLRKP
SILGVREHVFTGSVSSLGWFMSNQETSFVTLGQRVLAKPLRVRMHYGHPDVFDRIFHISRGGISKASKVINISEDIYAGFN
STLRQGNVTHHEYIQVGKGRDVGLNQIAIFEGKVAGGNGEQVLSRDIYRLGQLFDPVRMLSFFYTSVGFYICTMMTVLT
VYVFLYGKAYLALSGVETTLTGEETNNKALQTALNTQFLFQIGVFTAVPMIMGAILEQGVLKAFTGFVTMQLQAASVFFTF
SLGTRTHYFGRTILHGGAKYRSTGRGFVVQHISFAENYRLFSRSHFVKGFEIALLLIVYAAYGSSKGTVSYILLTFSSWFLALS
WLYAPFLFNPSGFEWQKAVEDFDKWSNWMFYKGGVGVKLDESWEAWWEDEMSHITSWWGRFWEIVFSLRFLIFQY
GVVYSLNVTGKDNSLYVYGYSWAALVGLLVLWKVFTIGQRAAANAQLLVRAFQTVILVGILTSLVVLIIRTSLTLADVFAAFF
LALLPTGWLILSICVALKPVVKPLGLWESVRSLARSYDALFGMLIFVPIAVLSWFPFVSTFQTRLVFNQAFSRGLEISLILSGK
QEDES------------------------------------------

>UCRN 2011996 Megaceros tosanus 1776

GIVYNIIPVHDPLVDHAAMQFPEVRAAIVALQTVGSLARPPYTEWRDGMDLLDWLGAFFGFQAHNISNQREHLALLLA
NGQMRLNPDPIDKLSSKVVSSIRKKTTKNYTSWCRFLEIPSNMSIIDTKYTSVNERLELLYTGLYLLIWGEAANLRFMPECL
CFLFHNMAGELNRILEDRIDEGTAQPARPAYCEPGGFLKRVVTPLYKIVSAESKASEHGKAPHSAWRNYDDMNEYFWTK
NCLTRLGWPLSLSSNYFVEPQSAGARNRVGKTGFVEQRSFWNIYQSFDRLWVGLILFLQAAIVLAWNGHNAPWIELKN
KDQVARVLSIFITWAGLRVLQAVLYMVMNRKLATEPLSQGVRMILKIVVAVAWTIAFAVLWSSMWNLRKQEGEGFDW
TPESNKRFRNVLYAGAVFIVPEVLALLLFILPWIRNFMENSQWKVFHLLTWWFQSRIYVARGLREGLLDNIKYTLFWVVTI
LTKFAFSYALQIQPLMKPTKEIIGLKRGDVTYRWPELFGRGTRFAIVAMWAPVILIYMMDLQIWYSVYSSLVGALVGLFQH
LGEIRNVEQLKERFHYFASALQFALMAEDQFIQQTGFGRNMFIRAKNAVKNSYTRFKLRYGFQADPYNKEERQLEAGRF
SYIWNEILAIFREEDLISDSELELLEVPAKAWDITVMQWPSVLLSNAILVALGLCKNFHGNDKALWKRVSRNDYRRCAVIE
CYESAQHVLLKIVAEDSEEYIIVKAILDELNSSLRLGTFLKKFDLAQLPNIHKKVSELSGALLGLGRSNQKQTRDKVVLALQN
LIRDTLIRDFPKSLGTYEDLGQGSSVYPSRSDGLLFTAESCVLLPDREDHAFYKQLKRLNNTLCTKEAILNVPQSLEARRRITF
FSNSLFMTMPRAPQVDRMLAFSVLTPYFKETVLYENWELEKRNEDGVSTIFYLQQIFPDDWKNFEERMANQDISKLEIS
TTEKGLFERRLWASYRGQTLARTVRGMMYYNRALEMLAFLDGASEVEVEQVQEILLHTSAPGSSIRPMRELSQHPSQH
SSGRLQSIRDKHRATAAMKFTYVAACQVYGQQKADDQKPGPKKEATHPARDILHLMKTYEGLRVAYVDERTVGKDAKE
YYSVLVKYDQAVQSEVEIYRVQLPGPLILGEGKPENQNHAFIFTRGEAVQTIDMNQENYFEEALKMRNLLQEFTINHGIR
KPTILGVREHVFTGAVSSLAWFMSAQESSFVTLGQRVLATPLKVRMHYGHPDVFDRLWFLTRGGISKASKVINISEDIFAG
FNCTERRGNITHHEYIQVGKGRDVGLNQIALFEAKVASGNGEQILSRDVYRLGHRLDFFRMLSFYYTTVGFFISNMLVVLT
VYSFLWGRVYLALSGVEASIVDAKTLDNTSLSAALNQQFLVQMGLFTALPMIVENTLERGFGKAIWEFLVMQLQLSSVFF

TFSMGTRAHYFGRTVLHGGAKYRATGRGFVVTHEKFAENYRLYSRSHFVKGLELILLLVVYAAYGETSKKSSVYILITFSSWF
LALTWIMAPFIFNPSGFDWLKTVEDMEDFGQWIFFKEGAIGEGKLSWERWWDEEQAHLRSTGFVGKVAEIILDLRFLFF
QYGIVYRLRISSGSRSIFVYLLSWIYVAAVGVVVKMITWGRDRYSAKAHSVYRLIQLFGAVLALLLIVLLVQLTAFEFIDVLIS
MLAFIPTGWALLQIAQVLRPLMEKTGIWPTVVALARLYEFGMGVAVLVPVAVLSWLPGFQAMQTRILFNEAFSRGLQIK
KLVTGKNPNAY-------------------------------------------------------------------------------------------------------------
----------------------------------------------------------------------------------------

>UCRN 2012030 Megaceros tosanus 1811

FNVVPLHDCPSARHPAKKFEEVQAATQALQTVGSLRQPPNVNWKDGMDLLDWLGAFFGFQRDNVRNQREHLVLLLA
NGQMRLSPKPIDKLKLAPEVITEIRRKVTKNYTSWCKFLNKRSSIRNLGGKRLDVASLDERLELLYTSLYLLIWGEAANLRF
MPECLAFIFHHMAGELNKIIEHQLDESTSEPSKPAYCEHLGFLNKVVKPFYQIIQAEAASSRNGTAPHSAWRNYDDLNEYF
WDPRCFTQIGWPLKAESKYFVEPRKGWNSTSYHFCLHICLKFLTAILISNKVTVIRECYLQWNVTAPRVGKTGFVEQRSF
WNIFRSFDRLWVILILFFQASIILSWNGGGPPWTELKNKDSLARVLSVFITWSGLRILQSLLDSAMESGLVTTETRLAGIR
MTLKVLVATAWTITFTILYGQMWKQRHASQRGWSPAANTKLYNFLYAAAVFILPEVLATGLFALPWLRNFIETSNWTLFH
VVTWWFQSRLYVGRGLREGIADNIRYSLFWIVILGSKFTFSYILQIKPMIQPTKELLDFPDSAIIYRWHEFFRHGNRIAAVC
LWAPVVLIYFMDLQIWYSVYSSLVGALVGLFQHLGEIRNYQQLKLRFQFFAKALQYVLIADEDYKGSSFWSNWWTKAKE
GIKRFKLRYGFNTDDHYDKVNRNQIEENKFEQIWNEIITIFREEDLISDREVELLEVPITVQKAWNIEDVMQWPLILIANEI
LVALGQCKDFQGGSDNTLWQRISKSEYRRCAVIEVYETIKQVMLRIIAKEKDEYKIITSLIDEIDTSLKVGNFTKKFELSSLPEI
HSRIVSLVNSLMQYQHTESESDEKRVVDAVQYLYDGLVRDLSTWEDIGKQELSLYPTRDAENLLFVDAVDLPSKDDDFYR
HLKRLYTTLSTKESLLNVPESLEARRRITFFSNSLFMTMPRAPQVEKMLAFSVLTPYYDETVLFSKEQLRTENEDGVSTLFYL
QKIYPDEWRHFAERMEKRSINVESPGIWDGDGLLELRLWASYRGQTLARTVRGMMYYDRALQLLAFIDTAKQNDIERV
RNLLYKTSSGLASRLHGSSSFILRDGISPHGLHSGAEFYDKKIKEKHHATAAMKFTYVVTCQIYGIQKAKKEAVAVDIKLLM
ERFPALRIAYVDVSESGKEYYSVLSKHDGTLDKEVEIYRIQLPGPVKIGEGKPENQNHAIIFTRGDAVQAIDMNQDNYFEE
ALKMRNLLQEFLQYYGIRKPTILGVREHVFTGSVSSLAWFMSAQESSFVTLGQRVLATPLKVRMHYGHPDVFDRLWFLT
RGGISKASKVINISEDIYAGFNCTLRGGNVTHHEYIQAGKGRDVGLNQIALFEAKVASGNGEQVLSRDVYRLGHRLDFFR
GLSFYFTTVGFFINNLIVVLTVFAFLWGRVYLALSGVEASIIGSNNDPISELNSNALAASFNQQFVVQIGLFTALPMIVENSL
EHGFTRAIWEFTTMQLQLASIFFTFSMGTRAHFFGRTILHGGAKYRATGRGFVVKHEKFAENYRLYSRSHFTKSLELVLLLI
VYQVYGTVTKSTASYLLMTFSAWFLALSWLMAPFIFNPSGFDWLKTVDDVDDFTNWIFFRGGILVKANQSWEAWWEE
EYQHLHSAGLWAKLLEIILNFRFFFFQYGIVYRLKIAAGSTSIMVYLLSWIYIVVVGVLQKFLSSAREKYAATTHRTYRGIQAA
VIAGVVAVIILLLKFTHFVFMDVLVSLMAFLPTGWALIQIAQVLKQPYLEGTPLWSTVVAVARLYEFGMGMLVLSPVACLS
WLPGFQAMQTRILFNEAFSRGLQISRILVGRKP---------------------------------------------------------------------------------
------------------------------------------------------------------------------------------

>UCRN 2054246 Megaceros tosanus 1933

KARKNWEQLVRKALLRAGDEPGLAGWGRLFGAPSYEATPALPSALGNTSIDAVLAASNEIQAASPQVARILCEHAYRLSQ
NLDPKSQGRGVLQFKTGLLSIIKQKRSKKEGEQIDRSNDVKLIFEFYKTYRKKLDELEDEQNKKRQPGERGDVVLNKEDKA
RAANIKKMYETARVLNEVSDAYLKETSPEEREKLLGDLQLKKDMERDAAKIAGFKSYNIIPLETPSADPYVLLPEVVGAHN
ALRIGGEIPALPDTFPRPDQNQRHLDMFDLLQYMFGFQADNMKNQREHVILLLANTQARLSLPDDRAEGVTLRLDDGS
MRHLHDKVLDNYRKWCSYLRKTSVLRKGNSAPVQLLLTCLYLAIWGEAGNVRFLPECICYIFHRMATHLLQQLGRSVADR
ASGVGDGDHEFLDQVVKPVYEIIAAEAKHGQGKKGSHSQWRTYDDLNEYFWSDHCFTLGWPWAPDATFFLKPGGGK
KDIANESAVGRRKQTRMGKTLFVEHRTGFHIYHSFHRLWIFFIMMLQGLGIFAFSTDLQTLHRIKVILSLGPSFVLLKFLQSL

FDVILMYGAYASTRGQNVARIFVRFIWFSASSAFVVYLYVRVMSEADLNSSSALFKIYVLVLGCYAGFQLVVSTISRISVLRV
QADKIGGWSIIRFISWVHQERYYIGRGLYERTQDFLLYMLFWVVVLVAKFSFSYFFQIKPMVEPTRIIIGRHIANYKWHDF
VSKSNHNALTLVALWAPVIMIYIVDTQVWYTVFSALWGGLDGARLRLGEIRSLSMLRKRFNTFPDEFVSRLLPSVLKLKID
TASPLGQLAPRTDKVNALKFAPLWNEIVENLRLEDYISTKERDWLLMPKNTGSLQLVQWPLFLLANKILLAVDLAAEHKG
SQYELQEKVFKDEYMQFAVQEAYFSVQRMMMKLVNEEAELWVKGIFADIEASIKESALVTSIVLKRLSGPSGLVAKLATLT
GNLEKKENPALLTNATIALQEIFEVVMKDVLSLTLREKFESWPVLEKARRTGALFSDLNWPSSIEENEEIKRLNSLLSVKDSA
SNIPKNLEARRRLQFFSNSLFMDMPATPSVRQMFSFSVFTPYYAEDVMYSYEALNKENEDGITILFYLQKIFPDEWKNYLD
RVFRTEAQLERELGVHLEKIKESTEPAEVPASDELLQLRLWASYRGQTLARTVRGMMYYRSALILQSQLEGSSVPDEEAAI
RSSAGARQPSLMEKSIADMKFTYVVSCQIYGKQKQQRSQQAADIAYLLRTHEGLRVAYIDTVEKIIEGKTFSEYFSKLVKAD
TTAKDQEIYSIRLPGNPLLGEGKPENQNHAIIFTRGDAIQTIDMNQDNYFEEAFKMRNLLQEFDVQHGLRKPSILGVREH
VFTGSVSSLAWFMSNQETSFVTLGQRVLAKPLKVRMHYGHPDVFDRIFHITRGGVSKASKVINISEDIYAGFNSTLRQGN
VTHHEYIQVGKGRDVGLNQIATFEAKVASGNGEQVLSRDIYRLGQLFDFFRMLSFFYTSIGFYVCTMLTVLTVYVFLYGKA
YLALSGVGADLKSDVLNSKALQTALNTQFLFQIGIFTAIPMIMGFILEQGVLKAFISFITMQLQLASVFFTFSLGTRTHYFGR
TILHGGAKYRATGRGFVVQHIPFAENYRLYSRSHFVKALEVAILLIVYGAYGSSEGTVSYVLLTFSSWFLSISWLYAPYIFNPS
GFEWQKTVEDFDKWTNWLFYKGGVGVGSNESWEAWWEEEQAHITSPRGRFWEIILSLRFFIFQYGVVYSLSATGNNTS
LGVYGISWLVLVGLLVVFKAFTFSQKASAKFQLVVRLGQGVLFLLLIAGIVLSIVYTELSLGDVFASLLALLPTGWAILSICLAIR
PLVEYLRLWKSVRALARFYDALMGMVIFVPIAVLSWFPFVSTFQTRLVFNQAFSRGLEISLILAGNKPN---------------------
--------------------------

>WCZB 2120204 Phaeoceros carolinianus 1781

FNVVPLHDCPSSRHPAKKFEEVQAAIQALQTVGHMRRPPNVEWKDGMDLLDWLGAFFGFQRDNVRNQREHLVLLLA
NGQMRLSPKPIDRRKLAPEVITEIRRKVLKNYTSWCKFLNKRSNIRNLGGKRLDVASLDERLELLYTSLYLLIWGEAANLRF
MPECLAFIFHNMAGELNKIIEHQLDESTSEPAKPAYCEPLGFLNNVVKPLYQILQAESASSRNGTAPHSAWRNYDDLNEYF
WDARCFTQLGWPLKPESNYLVEPPKGRWNMNTHRVGKTGFVEQRSFWNIYRSFDRVWIILILVFQASIILSWNGGGPP
WAELKNKDSLARVLSVFITWGGLRILQSLLDAGMESDLVTAETRLIGIRMTLKVLVATAWTITFIVLYGQMWKQRHSSSSQ
FWSAAADAKLYNFMYAAAVFIFPEVLATLLFVVPWFRNFIENSNWRVFHLLTWWFQSRLYVGRGLREGIADNIRYTSFW
IVILGSKFTFSYILQIKPMIQPTKELLAFPDSVIIYRWHEFFRHGNRIAAACLWAPVILIYFMDIQIWYAVYSSLVGALVGLFQ
HLGEIRNYQQLKLRFQFFASALQYVLIADGSYTGQSFWTRWWRKMKEGVKRFKLRYMDDKYNKLKENKKEANKFEQI
WNEIITIFREEDLISDSEVELLEVPIPIQKAWTITVMQWPLILIANEILVALGQCKNFHGSDQMLWRRIIKSEYRRCAVMEV
YQSISQMLLKIIIEGSDEYIIMKSLIAEIDASLKSETFTKKFQLSALPEIHSRIVSLVSNLMQYQHTESESDEKRVVDAVQYLYD
GLVRDFPVTTWEDLRGGQDLSLYPLRDKENLLFVDAVKLPRKDESIHRDFYRQLKRLHTTLSMKESLLNVPQSLEARRRIT
FFSNSLFMTMPRAPEVEKMLAFSVLTPYYDETVIFSKEQLWTENEDGVFTLFYLQKIYPDEWRHFAERMEKRSLNINNIHI
WENDDLRELRLWASYRGQTLARTVRGMMYYDRALQLLAFIDTAEESDIDRVRHLLYKTSSGMASHLHGTTSFLLREGVS
PHGLHSSAEFYDKKIKDKHQATAAMKFTYVVTCQIYGMQKAKNEAVAHDIQYLMKEFPALRVAYVDVSPSGKEHYSVLA
KYDTSLENEVEIYRIQLPGPVKVGEGKPENQNHAIIFTRGDAVQAIDMNQDNYFEEALKMRNLLQEFTQYYGIRKPTILG
VREHVFTGSVSSLAWFMSEQESSFVTLGQRVLATPLKVRMHYGHPDVFDRLWFLTRGGISKASKVINISEDIYAGFNCTL
RGGNVTHHEYIQAGKGRDVGLNQIALFEAKVASGNGEQVLSRDVYRLGHRLDFFRMLSFYFTTVGFFINNLIVVLTVYAF
LWGRVYLALSGVEASIIGSNNDPISTINSDSLAASFNQQFVVQIGLFTALPMIVENSLEHGFTTAIWEFITMQLQLASIFFTF
SMGTRAHFFGRTILHGGAKYRATGRGFVVKHEKFAENYRLYSRSHFVKGLELVLLLIVYQVYGTVSKSTASYLLMTFSSWFL
AITWLMAPFIFNPSGFDWLKTVDDVEDFTNWIFFRGGILVKANQSWEAWWDEEYQHLHSAGLWAKLLEIILNFRFFFF
QYGIVYRLKIAAGSTSILVYLLSWIHVIVVGVLQKFLSSAREKYAATTHRTYRSIQAAVIAGVVTVIILLLKFTHFVFMDVLVSL
MAFLPTGWALIQIAQVLKKPYLEGSPLWSTVVAVARLYEFGMGMLVMIPVACLSWLPGFQAMQTRILFNEAFSRGLQIS

RILVGTKKP----------------------------------------------------------------------------------------------------------------------------------
----------------------------------------------------------------------------

>WCZB 2120211 Phaeoceros carolinianus 1768

YNIVPVHDTLADHAALQFPEVRAAIVALQTVGSLARPPYLEWRDGMDLLDWLGAFFGFQADNINNQREHLALLLANG
QMRANPDPIDKLSSKVVSSIRKKVTKNYVDWCRFLELPINMSIVDSKYTSSSNERLELLYTGLYLLIWGEAANLRFMPECL
CFLFHNMAWELNRILEDRIDEGTAQPAKPAYCVPGGFLKSVVTPLYKVLSAESKASEHGKAPHSAWRNYDDMNEYFWD
KRCLTKLGWPLSLSSNYFVEPQAKAPHRVGKTGFVEQRSFWNIYQSFDRLWIGLILFLQASIILAWNGDKAPWIELQNKD
QLARILSIFITWAALRVLQAVLNILMQHKLVSAETLSLGVRMLLKVVVAVAWTIAFAVLWSSLWNLRLKEGIGFEWTPESN
KRFRNMLYAGAVFIFPEALALLLFILPWVRNFVENSQWKVFHLLTWWFQTRIYVARGLREGLYDNIRYSLFWIIVIVTKFTF
SYTLQIRPLIKPTKETINLQSDEVHYRWHELFGRGTRFAIVAMWAPVIMIYMMDLQIWYSVYSSFTGALVGLFQHLGEIR
NVEQLKQRFQYFASALQFSLMAEDQFVKPKGFGRNWWNSIVNSYTRFKLRYGFGDDSYGKEDRQIEAGRFSYIWNEIM
AIFREEDLISDRELELLEVPAKAWNISVMQWPSVLLSNAILVALGQCKNFYGNDKALWRRVGRNEYRRCAVIECYESAQH
VLLRIVAENTEEYIMIKAILDELNLSLRLGTFSKKFDLTQLPNIHKRVTALTNVLLKWHKQQNSREQVVLALQDLYDTLIRDF
PRASGTYAELDRGSSVYPSRSGGLLFASDESVQLPDPEDLAFYKQLKRFNTTLSTKEAILTVPHSLEARRRITFFSNSLFMT
MPRAPQVDRMLAFSVLTPYYKETVLYENWELEKRNEDGVSTIYYLQQIFPEDWNNFEERMERQGISKDEIDRTEKGLFE
RRLWASYRGQTLARTVRGMMYYSRALELLAFLDGASEVEVEKMQEMFLRTSNSTAGPSVRPMMEISQHSSRHSTGRL
HSNRDKHRATASMKFTYVAACQVYGQQKADEKKPGTKKESTHPARDILYLMKTYGGLRVAYVDEKPVGRAEKEYYSVLV
KYDHDTQSEVEIYRVQLPGPLILGEGKPENQNHAFIFTRGDAVQTIDMNQENYFEEALKMRNLLQEFTVQYGLRKPTILG
VREHVFTGAVSSLAWFMSAQESSFVTLGQRVLANPLKVRMHYGHPDVFDRLWFLTRGGISKASKVINISEDIFAGFNCTE
RGGNITHHEYIQVGKGRDVGLNQIALFEAKVASGNGEQILSRDVYRLGHRLDFFRMLSFYYTTVGFFISNMMVVLTVYSF
LWGRCYLALSGVEASIVSAKTLDNASLTASLNQQFLVQMGLFTALPMIVENTLERGFGNAVWEFVVMQLQLSSVFFTFS
MGTRAHFFGRTVLHGGAKYRATGRGFVVTHEKFAENYRLYSRSHFVKGLELIFLLIIYGSFGELSKTSSVYILITFSSWFLALT
WIMAPFIFNPSGFDWLKTVEDMEDFANWIFFKEGGISDAKISWERWWDEEQAHLQGTGILGKIAEIILDLRFLFFQYGIV
YRLRISAGSRSILVYLLSWIYVFVMGAFVKVITWARVRYSAKEHSIYRLIQLFTAVFCLLLIILLVQLTDFAFIDVLLSMLAFIPT
GWAVIQIAQVLRPWLEKTPLWQTVVALARLYEFGMGVVVLVPVAVLSWLPGFQAMQTRILFNEAFSRGLHFKQIVTGR
NPNS-------------------------------------------------------------------------------------------------------------------------------------------
--------------------------------------------------------------------------------

>WCZB 2120236 Phaeoceros carolinianus 1928

KARKNWEQLVRKALLRISDDSGGTGWGRLFGPSSYESTPALPSALGNTSIDAVLAASNEIQKESPQVARILCEHAYRLSQN
LDPKSQGRGVLQFKTGLLSIIKQKQSKKDGERIDRSNDVKLIFEFYRTYRKKLDELEAEEQKFRELGDSVILKAEDKKRAASI
KKMYETSRVLNDVLDALMKEASPEDKESFQRESDQQLKKDLERDAAKIADFKAYNIIPLETPTAVPFVLLPEIEGARFALKL
GGEVPAALLPETYPRPNQNLRPFADMFDLLQYIFGFQKDNVSNQREHVILLLANTQARLSPPDDRVDGLRLDDGSIRQL
HDKVLDNYKKWCSYLRKDSVCRKGSSVAIQLLLTCLYLAVWGEAGNVRFLPECICYIFHHMAKELLHQLGEPVAGRARGV
GDGDHEFLDQVINPIYSIIAAEAKHGQGKKGSHSQWRTYDDLNEYFWSDHCFTLGWPWNRDAPFFLKPSVVKQDGVD
NPPRRKQRMGKTLFVEHRTGFHIYHSFHRLWIFFILMLQGLGIFAFSTELQTVHRIKIILSLGPSFVILKFLQSVFDVILMYGA
YASTRGQNVARIFVRFIWFATSSTFVTYLYVRVMSQPNLNQGSSLFNIYLLVLGCYAGFQLIVSTISRISALRIQADKIGSWSI
VRFISWVHQERYYIGRGLYERTLDFLLYMLFWVVVLVAKFAFSYFFQIKPMVGPTRIIINTRIQSYNWHDFFSQSNHNALT
LVALWSPVVMIYFLDTQVWYTVFSALWGGLDGARLRLGEIRSLSMLRKRFNTFPQEFVNTLLPSVVKSKINTSIPTGLLDQ
TEKVNALKFAPLWNEIILNLRLEDYISNKEKDWLWMPKNTGSLQLVQWPLFLLANKILLAVDLAAEHKGSQFELQEKIFKD

EYMGFAVLEAYFSVQRMLNNLVKNEAELWVKGIFADVEASLNDSALATSIVLKKLSGPGGLIAKLANLTGILEKEFSSERSKL
ATNALQDIFEVVMKDVLSLSLREKFESWPVLERARRTGLFFSDLNWPSGPEENEEIKRLNSLLTVKDSASNIPRNLEARRR
LQFFSNSLFMDMPATPSVRQMFSFSVFTPYYTEDVMYSYGALNKPNEDGITILFYLQKIFPDEWKNYLERVFRTEATLERE
LDRHLEKLKDKKEDVADTPELLQLRLWASYRGQTLARTVRGMMYYRSALILQSQLEGSGPPDLEAATRPVSGALPPSLIG
QSIADMKFTYVVSCQIYGKQKQQRHQQAADIAYLLQTHEGLRVAYIDTVEKIVDGKTSSEYFSKLVKADATGKDQEIYSIRL
PGNPLLGEGKPENQNHAIIFTRGDCIQTIDMNQDNYFEEAFKMRNLLQEFDVQHGLRKPSILGVREHVFTGSVSSLAWF
MSNQETSFVTLGQRVLAKPLKVRMHYGHPDVFDRIFHITRGGISKASKVINISEDIYAGFNSTLRQGNVTHHEYIQVGKG
RDVGLNQIALFEAKVASGNGEQVLSRDIYRLGQLFDFFRMLSFFYTSIGFYVCTMMTVLTVYVFLYGKAYLALSGVGAELE
KDVVNSTALQTALNTQFLFQIGVFTAIPMIMGFILEQGVLKAFISFVTMQLQLASVFFTFSLGTRTHYFGRTILHGGAKYRA
TGRGFVVQHIPFAENYRLYSRSHFVKGLEVAILLIVYGAYGSNKGTVSYVLLTFSSWFLSISWLYAPYIFNPSGFEWQKAVED
FDKWTNWIFYKGGVGVNSDESWEAWWEEEQAHITSPRGRFWEIVLSLRFFIFQYGVVYSLTATGTNTSLSVYGISWLAL
VGLLIVFKAFTFSQKASAKFQLVVRLGQGVLFILLIAGIVLSIVYTDLSLGDVFASLLALLPTGWGILSICLAIRPVVEKLRLWK
SVRAFARFYDALMGMLIFVPIAVLSWFPFVSTFQTRLVFNQAFSRGLEISLILAGNKPN---------------------------------------
-------------

>WEEQ 2014103 Phaeoceros carolinianus gametophyte 1819

EASPEDKESLQRESDQQLKKDLERDAAKIADFKAYNIIPLETPTADFVLLPEIKGAHFALKLGGEVPATLLPETYPRPKQDLR
PLDMFDLLQYIFGFQEDNVSNQREHVILLLANTQARLLPPDDRVDGFSVRLDDGSIRHLHDKVLDNYKKWCSYLRKDSV
CRKGSSVAIQLLLTCLYLVIWGEAGNVRFLPECICYIFHHMAKELLQQLGGPVAGRSRGVGDGDHEFLDQVISPIYSVIAAE
AKHGQGKKGSHSQWRTYDDLNEYFWSDHCFTLGWPWNRDAPFFLKPSVVKQDGVDNPPRRKQRMGKTLFVEHRTS
IIPLGGSKGGKTLFVEHRTRFHIYHSFHRLGIFFILMLQGLGIFAFSTELQTVHQMKVILSLGPSFAVLKFLQCSGLVLAGGK
MPCSRLVNPNRLFSIACIGSKLLVIGKGSSGIKRQYCGVESVFAAVFDVILMYGAYASTRGQNVARIFVRFIWFATSSTFVTY
LYVRVMSQPNLNQGSSLFNIYLLVLGCYAGFQLIVSTISRISALRIQADKIGSWSIVRFISWVHQERYYIGRGLYERTLDFFFY
MLFWVVVLVAKFSFSYFFQIKPMVGPTRIIINTRIQAYNWHDFFSQSNHNALTLVALWAPVVMIYFLDTQVWYTVFSAL
WGGLDGARLRLGEIRSLSMLRKRFNTFPQEFVNTLLPSVVKSKINTSIPTGLLDQTEKVNALKFAPLWNEMILNLRLEDYIS
NKEKDWLWMPKNTGSLQLVQWPLFLLANKILLAVDLAAEHKGSQFELQDKIFKDEYMGFAVQEAYFSVQRMLKNLVK
NEAELWVKGIFADVEESITESALATSIVLKKLSGPGGLIAKLANLTGILEKEFNSERSKLATNALQDIFEVVMKDVLSLSLREK
FESWSVLEKARRTGLFFSDLNWPSSPEENEEIKRLNSLLTVKDSASNIPRNLEAQRRLQFFSNSLFMDMPATPSVRQMFS
FSVFTPYYTEDVMYSYGALNKPNEDGITILFYLQKIFPDEWKNYLERVFRTEATLERELDRHLEKLKDKKEDVADTDVLLQL
RLWASYRGQTLARTVRGMMYYRSALILQSQLEGSGPPDLEAATRPVSGALPPSLIGQSIADMKFTYVVSCQIYGKQKQQ
RQQQAADIAYLLQTHEGLRVAYIDTVEKIVDGKTSSEYFSKLVKADATGKDQEIYSIRLPGNPLLGEGKPENQNHAIIFTRG
DCIQTIDMNQDNYFEEAFKMRNLLQEFDVQHGLRKPSILGVREHVFTGSVSSLAWFMSNQETSFVTLGQRVLAKPLKV
RMHYGHPDVFDRIFHITRGGISKASKVINISEDIYAGFNSTLRQGNVTHHEYIQVGKGRDVGLNQIALFEAKVASGNGEQ
VLSRDIYRLGQLFDFFRMLSFFYTSIGFYVCTMMTVLTVYVFLYGKAYLALSGVGAELEKDVVNSTALQTALNTQFLFQIGV
FTAIPMIMGFILEQGVLKAFISFVTMQLQLASVFFTFSLGTRTHYFGRTILHGGAKYRATGRGFVVQHIPFAENYRLYSRSH
FVKGLEVALLLIVYGAYGSNKGTVSYVLLTFSSWFLSISWLYAPYIFNPSGFEWQKAVEDFDKWTNWIFYKGGVGVNSDES
WEAWWEEEQAHITSPRGRFWEVVLSLRFFIFQYGVVYSLTATGTNTSLNVYGISWLALVGLLIVFKAFTFSQKASAKFQLV
VRLGQGVLFILLIAGIVLSIVYTDLSLGDVFASLLALLPTGWGILSICLAIRPVVEKLRLWKSVRAFARFYDALMGMLIFVPIA
VLSWFPFVSTFQTRLVFNQAFSRGLEISLILAGNKPN------------------------------------------------------------------------
--------------------------------------------------------------------------------

>WEEQ 2091206 Phaeoceros carolinianus gametophyte 1608

LNKIIEHQLDESTSEPAKPAYCEPLGFLNNVVKPLYQILQAESASSRNGTAPHSAWRNYDDLNEYFWDARCFTQLGWPLK
LESNYLVEPPKGRWTMNTHRVGKTGFVEQRSFWNIYRSFDRVWIILILVFQASIILSWNGGGPPWAELKNKDSLARVLS
VFITWGGLRILQSLLDAGMESDLVTAETRLIGIRMTLKVLVATAWTITFIVLYGQMWKQRHSSSSQFWSAAADAKLYNFM
YAAAVFIFPEVLATLLFVVPWFRNFIENSNWRVFHLLTWWFQSRLYVGRGLREGIADNIRYTSFWIVILGSKFTFSYILQIKP
MIQPTKELLAFPDSVIIYRWHEFFRHGNRIAAACLWAPVILIYFMDIQIWYAVYSSLVGALVGLFQHLGEIRNYQQLKLRF
QFFASALQYVLIADGSYTGQSFWTRWWRKMKEGVKRFKLRYGDDKYNKLKENKKEANKFEQIWNEIITIFREEDLISDSE
VELLEVPIPIQKAWTITVMQWPLILIANEILVALGQCKNFHGSDQMLWRRIIKSEYRRCAVMEVYQSIKQMLLKIIIETSDE
YTIMKSLIAEIDASLNSETFTKKFQLSALPEIHSRIVSLVSNLMQYQHTESESDEKRVVDAVQYLYDGLVRDFPVTTWEDLR
GGQDLSLYPLRDKENLLFVDAVKLPRKDESIHRDFYRQLKRLHTTLSMKESLLNVPQSLEARRRITFFSNSLFMTMPRAPE
VEKMLAFSVLTPYYDETVIFSKEQLWTENEDGVFTLFYLQKIYPDEWRHFAERMEKRSLNINDIHIWENDDLRELRLWAS
YRGQTLARTVRGMMYYDRALQLLAFIDTAEESDIDRVRHLLYKTSSGMASHLHGTTSFLFREGVSPHGLHSSAEFYDKKIK
DKHQATAAMKFTYVVTCQIYGIQKAKNEAVAHDIQYLMKEFPALRVAYVDVSPSGKEHYSVLAKYDTSLENEVEIYRIQLP
GPVKVGEGKPENQNHAIIFTRGDAVQAIDMNQDNYFEEALKMRNLLQEFTQYYGIRKPTILGVREHVFTGSVSSLAWF
MSAQESSFVTLGQRVLATPLKVRMHYGHPDVFDRLWFLTRGGISKASKVINISEDIYAGFNCTLRGGNVTHHEYIQAGKG
RDVGLNQIALFEAKVASGNGEQVLSRDVYRLGHRLDFFRMLSFYFTTVGFFINNLIVVLTVYAFLWGRVYLALSGVEASIIG
SNNDPISTLNSDSLAASFNQQFVVQIGLFTALPMIVENSLEHGFTTAIWEFITMQLQLASIFFTFSMGTRAHFFGRTILHG
GAKYRATGRGFVVKHEKFAENYRLYSRSHFVKGLELVLLLIVYQVYGTVSKSTASYLLMTFSSWFLAITWLMAPFIFNPSGF
DWLKTVDDVEDFTNWIFFRGGILVKANQSWEAWWDEEYQHLHSAGLWAKLLEIILNFRFFFFQYGIVYRLKIAAGSTSIL
VYLLSWIHVIVVGVLQKFLSSAREKYAATTHRTYRSIQAAVIAGVVTVIILLLKFTHFVFMDVLVSLMAFLPTGWALIQIAQV
LKKPYLEGSPLWSTVVAVARLYEFGMGMLVMIPVACLSWLPGFQAMQTRILFNEAFSRGLQISRILVGTKKP--------------
-------------------------------------------------------------------------------------------------------------------------------------------
-------------------------------------------------------------------------------------------------------------------------------------------
--------------------------------------------------------------------------------

>WEEQ 2091224 Phaeoceros carolinianus gametophyte 1609

FLFHNMAWELNRILEDRIDEGTAQPAKPAYCVPGGFLKSVVTPLYKVLSAESKASEHGKAPHSAWRNYDDMNEYFWDK
RCLTKLGWPLSLSSNYFVEPQAKAPHRVGKTGFVEQRSFWNIYQSFDRLWIGLILFLQASIILAWNRDKAPWIELQNKDQ
LARILSIFITWAALRVLQAVLNILMQHKLVSAETLSLGVRMLLKVVVAVAWTIAFAVLWSSLWNLRLKEGIGFEWTPESNK
RFRNMLYAGAVFIFPEALALLLFIIPWVRNFVENSQWKVFHLLTWWFQTRIYVARGLREGLYDNIRYSLFWIIVIVTKFTFS
YTLQIRPLIKPTKETINLKSDEVHYRWHELFGRGTRFAIVAMWAPVIMIYMMDLQIWYSVYSSFTGALVGLFEHLGEIRNV
EQLKQRFQYFASALQFALMAEDQFVKPKGFGRSWWNSIVNSYTRFKLRYGFGDDSYGKEDRQIEAGRFSYIWNEIMAIF
REEDLISDRELELLEVPAKAWNISVMQWPSVLLSNAILVALGQCKNFYGNDKALWRRVGRNEYRRCAVIECYESAQHVLL
RIVAENTEEYIMIKAILDELNLSLRLGTFSKKFDLTQLPNIHKRVAALTSVLLKWHKQQNGREQVVLALQDLYDTLIRDFPRT
SGTYAELGRGSSGYPSRSGGLLFASDESVQLPDPEDLAFYKQLKRFNTTLSTKEAILTVPHSLEARRRITFFSNSLFMTMPR
APQVDRMLAFSVLTPYYKETVLYENWELEKRNEDGVSTIYYLQQIFPEDWNNFEERMERRGISKDEIDRTEEGLFERRLW
ASYRGQTLARTVRGMMYYSRALELLAFLDGASEVEVEKVQEMFLRTSNSTAGPSVRPMREISQHSSRHSSGRLHSNRD
KHRATASMKFTYVAACQVYGQQKADEKKPGTKKESTHPARDILYLMKTYGGLRVAYVDEKPVGRDEKEYYSVLVKYDHD
TQSEVEIYRVKLPGPLILGEGKPENQNHAFIFTRGDAVQTIDMNQENYFEEALKMRNLLQEFTVKYGLRKPTILGVREHVF
TGAVSSLAWFMSAQESSFVTLGQRVLANPLKVRMHYGHPDVFDRLWFLTRGGISKASKVINISEDIFAGFNCTERGGNIT
HHEYIQVGKGRDVGLNQIALFEAKVASGNGEQILSRDVYRLGHRLDFFRMLSFYYTTVGFFISNMMVVLTVYSFLWGRC
YLALSGVEASIVSAKTLDNASLTASLNQQFLVQMGLFTALPMIVENTLERGFGNAVWEFVVMQLQLSSVFFTFSMGTRA
HFFGRTVLHGGAKYRATGRGFVVTHEKFAENYRLYSRSHFVKGLELIFLLIIYGSFGELSKTSSVYILITFSSWFLALTWIMAP
FIFNPSGFDWLKTVEDMEDFANWIFFKEGGISDAKISWERWWDEEQAHLQGTGILGKIAEVILDLRFLFFQYGIVYRLRIS

AGSRSILVYLLSWIYVFVVGAFVKVITWARVRYSAKEHSIYRLIQLFTAVFSLLLIILLVQLTDFAFIDVLLSMLAFIPTGWAVIQ
IAQVLRPWLEKSPLWQTVVALARLYEFGMGVAVLVPVAVLSWLPGFQAMQTRILFNEAFSRGLHFKQIVTGRNPNS-----
--------------------------------------------------------------------------------------------------------------------------------------
--------------------------------------------------------------------------------------------------------------------------------------
------------------------------------------------------------------------------------

>ZFRE 2118264 Phaeoceros carolinianus sporophyte 1545

GKTPHSGWRNYDDMNEFFWDKKCFATVGWPLSGSSNYFMKTGDKESRQKVGKTGFVERRTFWNIYRSFDRLWIGLIL
FLQASIILAWNAQQPRKFWLELRDKDQIARILTIFITWAGLRVLQAVLNIVTQFKLVSSANLSLGVRMILKVVVAVAWTVLF
AVLWSSLWSIRKEEIPLDWSSKSNQRFRNVLYTAAVFLSPEGLALLLFIIPWIRKYVEKSDLKLFNVLTWWFQSRIYVARGL
RESFVDGLRYGAFWLVVIAVKFAFSYYLQIEPLIKPTKETLRLPSDAITYRWDQIFGRGNHVAILVMWAPVLLIYFMDMQL
WYSVCSSYVGAFVGLFRNIGEIRSVGQLEERFTFFAEISQFALVAEDKVMKPRQKVFARSWQQLKENFTRVRLRYGFGAE
AFELEPGNVALARFSLIWNEIIANFREEDIISNRELELLEMPPSVWNITVVQWPAILINNSVLVVLGKVEKYIKKGKDGKGI
WDKIRRNKFQEAAITESYQSVRHLLLRIVAEQTHEHFLVTTILEDIDLAIRNGHFFQKFELKPLRTIQQQVVKLTMDLLKWK
EEKISQAQVVTAVQNLFDTLIRDLPREKGAYSSNSVGNVYPSKPGELLFAAESSLQLPDREDITFFKQLKRLHTTLTTKETLL
DVPQNLEAKRRITFFANSVFMHMPRAPQVDRMLGFSVLTPYYKETVLFQHSDLETKTEDGVTTMFYLKKIFPDDWRNF
VERMKAKYNLQSEEEVESTQAGLFERRLWASYRGQTLARTVRGMMYYNKALELLAFLDGASELEVKNARERGGLQQS
GPAVYSRPMREISRRQTTGASSEVIPYSEKYHAMAAMKFTYVAACQVYGDQKAKKEKAAEDIQYLMKTYDGLRVAYVD
VKKGDRGTSYYSVLVKYDPATQTEVEVYRVQLPGPLILGEGKPENQNHALIFTRGDAVQTIDMNQENYFEEALKMRNLLE
EFHIAHGRTVPTILGVREHIFTGAVSSLAKFMSAQETSFVTLGQRVLATPLKVRMHYGHPDVFDRLWFLTRGGISKASKVI
NISEDIFAGFNCTARGGNVTHHEYIQVGKGRDVGLNQISMFEGKVASGNGEQVLSRDVYRLGHHLDFFRMLSFYYTTVG
FFVSNMIVVLCVYAFLWGRVYLALSGVESSIAHLDNKALTASLNQQFLVQLGIFSALPMIVENTLEMGFGHAVWQFILM
QLQMSSIFFTFSLGTRAHYFGRTILHGGAKYRATGRGFVVTHEKFAENYRLYSRSHFVKGLELILLLVVYESFGANRGTLVYI
LLTISSWFLAITWISAPFIFNPSGFDWLKTVEDLDDFTRWVFFREVGFSDGRVSWERWWDEEQAHLQGTGLLGKAAEIV
LSLRFLFFQWGLCYRFRTAAGNKSIYVYLLSWLYVLVLAVVVGFFTTIREKYASKQHIIYRLWQLLTALFVILIFVVLATVTSFK
FVDVFISILAFVPTGWALLQIAQVLRHPLLEKSGLWPIVVAVARLYEYGMGLLVFVPVACLSWLPGFQSMQTRMLFNEAF
SRGLNIMRILTGKNPNS------------------------------------------------------------------------------------------------------------
--------------------------------------------------------------------------------------------------------------------------------------
--------------------------------------------------------------------------------------------------------------------------------------
-------------------------------------------------

>Marpo2-Marchantia polymorpha 2015

MSSSTSQSIVPVGPARNASPRDRDGGDGDGGLRERARERDREREGDRDRDRERDRDRERERERGRDRERERE

RGRDSPARSPLPETQSTPGSGIRRGLSRTFTTANNNDVFDSEVVPSSLSAVAPILRVANEIESHRPRVAYLCRFYAFE

KAHKLDPTSSGRGVRQFKTSLLQRLERDNIPTLRARQKRSDARELQSFYQQYYENYVKALDGAEHSDRAQLTKAY

QTAGILFEVLTQVSKSEMAEAPPEIIAAGKIVEEKQEIYLPYNILPLDAAGASQAIMQLPEIKAGVEALRNIRGLPWPSQ

MEQARHKSGELDLLDWLQCMFGFQKDNVRNQREHLILMLANVHIRLIPKAEPMSKLEDRALNEVMDKLFKNYKSW

CKFLGRKNSLWLPTIQQEIHQRKILYMGLYLLIWGEAANLRFMPECLCYIYHNMAYELYGMLAGNVSVVTGENIKPAY

GGEDESFLKKVITPIYDIIFKEAKNNGNGTAAHSAWRNYDDLNEFFWSVDCFRLGWPMRLDADFFVPPSQTSLSSKL

SIGKGGKVASSRRLGKTNFVEIRSFWHLFRSYDRMWTFYILGLQAMIVMAWNIEGSGTFKSTFEGNNFKRILSIFITAA

ILRVIQGLLDIAMSMKAYRSIKFMGMLRLFLKLLVSIAWVIVLSVCFVHTWENPTGLIKNVQNVLGSSWKNPSLYITAVAI

YLLPNALAAVLFVFPLMRRWIENSNWRIVRLLLWWSQPRLYIGRGMHESQWTLFKYTMFWILLLASKFVFSYFIQIKP

LVKPTKTIMNDSSIQFTWHELFPNVKKNIGAIIAIWAPVILVYFMDTQIWYSVYSTVFGGVSGAFRRLGEIRTLGMLRS

RFRSLPGAFNANLVPADKVARRGFSLARGYKEVQPGKDRKEAAKFAQLWNEVITSFREEDLISNKEMDLMLVPYSS

VNLTLVQWPPFLLASKIPVALQMAVEHRGRDMDLWRKIRADDYMRCAVEECFESFKHVLGTILVGEVERRLVIDGILE

EIDKDISEGSLLSNFKMSALPVLHSKFVQLTEFLIKGEADKRDSVVLLLQDMFEVVTRDMMNETAREYLESTHGPLSI

SGKTSKDVKDHQLFAATDPKPAVLFPPPATDAWIEQIKRLHRLLTVKESAINVPTNLEARRRIAFFTNSLFMDMPRAPR

VRNMLSFSVLTPYYQEEVVYSKKQLNEENEDGISVLFYLQKIYPDEFDNFLERINVTSEHEIWDNEEYENELRHWAS

YRGQTLSRTVRGMMYYRRALELQAFLDMASDDELVDGYKVVASAPAEAKKSQRSMWAQLQAIADMKFTYVATCQI

YGAQKRAADVRATDILNLMLNNPSLRVAYIDEVEGREKDKNQKVYYSVLVKASNGLDQEIYRIKLPGIVKLGEGKPEN

QNHAMIFTRGEALQTIDMNQDNYLEEAFKMRNLLEEFHEPHGVRPPTILGVREHIFTGSVSSLAWFMSNQETSFVTI

GQRVLASPLKVRFHYGHPDVFDRLFHITRGGMSKASRVINLSEDIFAGFNSTLRRGNVTHHEYIQVGKGRDVGLNQI

SLFEAKVACGNGEQTLSRDMYRLGHRFDFFRMMSCYFTTIGFYASTVIVVLTVYVFLYGRIYLALSGIEKSLVNSADV

NNDPALQAALASQSLVQLGLLMALPMVMEIGLERGFRTALSDFIIMQLQLASVFFTFSLGTKTHYYGRTILHGGAKYR

ATGRGFVVRHEKFAENYRLYSRSHFVKGIELMMLLIIYSVYGTSAKGGVPYLLITFSMWFLVTTWLFAPFLFNPSGFE

WQKIVEDWDDWSKWINNRGGIGVLATKSWESWWEEEQEHLKHTGLMGRVLEVLLSIRFFLYQYGMVYTLSIAGGS

TSLSVYGLSWLVIIAVLAVLKIVSMGRRRFSADFQLMFRLLKALLFIGFVTIVIVLFLFAQLSVGDLFASLLAFLPTGWGL

LMIAQAARPLVVRSGMGDSVKALARAYEFIMGLCIFTPVAMLAWFPFVSEFQTRLLFNQAFSRGLQISRILAGRRKSS

>Marpo4-Marchantia polymorpha 1930

MRRREGVPGRNPERAWNNWERLIQKALRSEELKFSVAGGDDLGGVVPTSLGQESSNINLILQVAEEIQRDNPHVAR

ILCEYAYTMAQKLDPQSEGRGVLQFKTGLMSVIRQKQSKKEGERIDRSHDILQIQRFYEDYRSRNMIDELEKLQQQR

GRRLSEDPNEAQRRAQTMKKVYQISKVLNDVLNSLTRDSTEEEKDKLISKETKATMESAAAKTAEFKPFNILPLETPG

VSDTILLIDEVRAAALTLSYYDELPRLPESAYKAGLQRDVDIFDLLEYIFGFQIGNVNNQREHLILLLSNSQSAFGPPSR

GSEVDGDAVKRCYDRLLDNYNKWCQYLRITAVTESAAGDSHKMVLLTAMYLLIWGEAANLRFLPECLCYIFHHMVKE

LYGLLGNANAQRSMNYVEGSDCPFLDKIVTPLYSVIAAEAANTQDGKASHAAWRNYDDFNEYFWSPRCLESLSWP

WRPDAGFMMKPNTKKMKGGVNAVPGRKKEKKVGKMTFVEHRTGFHVYHSFHRLWIFFTVMLQGMMIFAFCDEKF

TPNTFKKMLSVGPTFVIMKLIQSLLDIFFIYGAYASTQGWTFSRIFARLAWFGGLSGSITWLFVKMIQEENTGTGSTWY
HLYLIVLGSYTGAQVVVTLLMRIPFFRRQADKCGDWAIIRFIMWVHQDRYFVGRGLYERTRDYAGYSAFWIFVLACKF
SFSYYFQVRALVSPTKTIVLQRFEYKWHDFVSRNNHNALTIGALWAPVILIYYLDTQVWYTVVSAIVGGLDGARARLG
EIRTLSMLRKRFSSFPEAFATNLVSSRLGRGEKNPGSSPAQVNWSMQKVQAVKFAPMWNEIINCLREEDYIGKKERE
LLVMPKNQGSGTLVQWPLFLLANKVYLAVEMAQDAKLLNQTQLWEKICKDEYMAYAIEEAYKLFEEVLKSLVREDVS
QIWVKGVFNDVGAGIQESALVGHFYLKKVDSVLARVTDLTQLLEREETDKLRSQVVLAMQNLYESVMNDFFTTELRE
RIEEFRALKDARIENKLFINLTWPTTDREKENVKRLHLLLTIKESAANIPRNLEARRRMEFFANSLFMDMPKPPSVQR
MLSFSVFTPYYAEDVLYSKDKLTEENEDGISILFYLQKIFPDEWKNFLERQGLTNALMERQMEDKKNDCQELRLWAS
YRGQTLARTVRGMMYYKKALILQSLLEGTSDVEEGGVLGTTLEESPGYRMASAIAETKFTYVVTCQIYGQQKERRE
DQAKDILYLMNKYDSLRIAYVDIVDKMQEDKETKEYKSIKEYYSKLIKADADGKDQEIYSIKLPGQFKLGEGKPENQN
QAIVFTRGDACQTIDMNQDNYFEEAFKMRNLLEEFNSTDSLRRPTILGIREHVFTGSVSSLAWFMSQQETSFVTLGQ
RVLARPLKVRMHYGHPDVFDRIFHITRGGISKASRTINISEDIYAGFNSTLRQGNITHHEYIQVGKGRDVGLNQIAMFE
AKVSSGNGEQILSRDVFRLGQLFDFFRMFSFFYTSVGYYITTLMTTLVVYAFLYGKVYLALSGMDRQLQDFADINNNK
ALESALNTQFLLQIGVFTAIPMIMNFILEQGVLKAVISFLTMQLQLCSVFFTFSLGTKTHFFGRTVLHGGAKYRATGRG
FVVQHISFADNYRLYSRSHFNKALEISMLLITYLSYGEDNRGVSYILLSFSSWFMALSWLFAPYIFNPAGFEWQKTVE
DFDDWTNWLFYKGGIGVKDNESWESWWESEQEHIRTLRGRFWEIVLSLRFFFFQYGVVYSLQVSGTSTSLSIYGY
SWLAFFIMIVLFQIFTFTNKSSVKFQLFLRLFQGGLFLALIASIAVTIAFTSLSVGDCFAMLLVFIPTGWGVLSICQALRPI
LEPLGAWNAVRSGFRLYDAMMGAVIFTPIAILSWFPFVSTFQTRLVFNQAFSRGLEISLILAGNRPNAST

>Marpo6-Marchantia polymorpha 1782

MEREQYRQRQSPDAAERGGYKMGGGQEVYNIIPVHNTLDEHPALRFPEVRAAIAALQVVGDLRKPSNMSWTSNLD
MLDWLCFTFGFQESNARNQREHLILLLANAQMRMQPPPDPIDKLQLEIVREIRKKITKNYIRWCSFLRIKHNLEKIEDH
GIRRRHESNEQKELLYVSLYLLIWGEAANLRFMPECLCFIFHHMCGELNRILDRSIDDSGQYMQPASGGIENGFLQK
VIRPIYAVVKAEAAECRAGKNPHSAWRNYDDMNEYFWSKRCLTQLRWPLEMSCNYLVKPEDKSRHKVGKTGFVEQ
RSFWNIYRSFDRLWIGLILMLQILIIVAFRAKAELPQDTLAPSTQETQSGAAKAPWTILAQRDSQAHVLSIFITWSALRV
LQAFLDFGMQYSLISAGTVMVGLRMTLKLIMGAVWTVLFSIYYSRMWAQRNADGLWSDKALQGLYLYLGFCAVFITP
EALALLLFILPYIRNFVETRDWRIFSMLTWWFQSHIFVGRGLRESIFDNIKYTLFWLCVLTAKFSFSYFLQVLPLVAPTR
ATLDVTGIEYRWHEFFKNANRTATLAMWAPVVLIYFMDLQIWYSVFSALVGALVGLLAHLGEIRNAAQLKARFHIFPVA
VQFNLMPEDIFVNSKPYSWRAKCNDWWVKVKNLARRVKLRYGVVKGHQAEGKTMETGRFRHVWNEIILIFREEDLI

SDRELELLSMPSSRWNITVTQWPSVLISNEVLIALGLCKDWYYTDHGLWKKIASNEYRRCAVVESYESIRHVLKRILR
EDSGEYQMFQEIFEEITKAIKEKQFVQRFSLKALPNIHSRLVQLINVLMKRPLLNDLQKVVDALQNLYEDLLRDFPKDQ
ELGRNWREMSSTSSLLFVDAVDLPDVTENTFFDQLRRVQTTLSTREALLDVPRNLEAKRRISFFSNSLFMTMPRAP
QVEKMLAFSVLTPYYSEDVMYHKKQLITENEDGVSILFYLQKIFKDDWENFQERMARHGIRTEFDMWELDDGLELRL
WASYRGQTLARTVRGMMYYHRALEKLAFLDHASEHDILEGYRELMERTSGGVDPFNGETREGSFNGETREGSLH
SASSFTEGKEGRNYNAYKREEDLATAAMKYTYVVACQIYGIQKAKGQQQAKDILYLMKTWEALRVAYVDEKPGLGD
KDPKRYASVLIKYDQVRQEEVEIYRVELPGDFKLGEGKPENQNHALIFTRGDAVQTIDMNQENYFEEALKMRNLLQE
FKQFHGRRRPAILGVREHVFTGAVSSLAWFMSAQETSFVTLGQRVLAKPLKVRMHYGHPDVFDRLWFITRGGLSKA
SRVINISEDIFAGFNATLRGATVTHHEYIQVGKGRDVGLNQIALFEAKVASGNGEQMLSRDVYRLGHRVDFFRMLSV
YYTTVGFFVNNMIVVLTVYAYLWGRVYLALSGIEVSITRDSSANGALTAALNQQFVVQMGVFTALPMIVENSLEKGFLL
AIWEFLTMQLQLASVFFTFSMGTRTHYFGRTILHGGAKYRATGRGFVVRHEKFSDNYRLYARSHFVKGLEIIILLIVYQ
MYGSIRNTTTYILTTFSCWFLGITWILAPFLFNPSGFDWLKSVDDFDDFMTWVWYKGGVFVKGEDSWQKWWDEEQ
EHFQSTGFWGKCLEIILSLRFFFFQYGIVYRLKISSGSTSIVVYLISWTYLFAAGLIHLILSHAAEKYGAKRHRKYRGIQA
FIICFLVLAVVLLFVFTDFSIWDIFTSILAFVPTGWGVLSICLVLRRPFLENTPVWPMVTGVARLYEMGMGICVMTPVVV

>Sphfa1-Sphagnum fallax 1934
MAASGGPDGISSPRVRRPLPRTNTTGFTDTFDSEVVPSSLSSIAPILRVANEVEADRPRVAYLCRYHAFEKAHRIDPT
SSGRGVRQFKTSLLQRLEKDNEPTLAARHRRSDAREIQSYYQQYYNNYVKALDGAEHSDRAALAKAYQTAAVLFDV
LKAVNRDKTEEPPPEIIAAAADVDEKKERYVPYNVLPLDAAGASQAIMQLDEIRAGVESLRNVRGLPWPSTAESRHK
PGDVDCLDWLQDMFGFQKDNVANQREHLILMLANVHIRLLPRPEPMHKLDDRALNAVMNKLFKNYKSWCKFLGRK
HSLWLPQIHQEIRQRKVLYMGLYLLIWGEAANLRFMPECLCYIYHHMASELHGMLAGNVSMVTGDNMKPAYGGEEE
IFLRKVVTPIYNVISQETKKNRNGTAPHSVWRNYDDLNEYFWKVDCFQLGWPMREDGDFFKPPDVAGPKLAVSAKL
MERKVWNPTGKTFFVEVRTFWHIFRSFDRMWAFYILGLQACIVLAWNVGRDLPNAFKDGVVVKRVLSIFITASILRLI
QAVLDIVMGYHAYHSLKFLGVLRLFLKLLTSAAWLIVLTVCYVHTWSHPQGLIKDIQNWLGKGWENSYLYVAAVVLYL
VPNVIGGFFFLFPMLRRWIESSNWRIVRFLLWWSQPRLYIGRGMHESQFALFGYTFFWVLLLASKFAFSYFIQIEPLV
GPTKKIMQQSSVTYTWHEFFPHAKNNPGALISLWAPVVLVYFMDSQIWYAVYSTIYGGISGSFRRLGEIRTLGMLRS
RFSSLPGAFNANLVPADGTKRRHRFSFRRNFKKILPQQEKLKAARFSQLWNEVICSFREEDLISDKERDLMLVPYSS
DPHLNLVQWPPFLLASKVPIALQMARQAAETGRAADLLRKIRTDEYMKSAVIECYESFKRVLKVLIVGEVEKRVIEGLL
NEVEMNIEKETLLDNFRLKELPVLSVKFIELLELLEKTDEEGVEAARDLAVLKLQDMYEVVTRDMMTESMRDTWESS

HGALAREQEKEELFSAKGERPAVLFPPPRREAWIEQIKRLHLLLTERESAMDVPENLEARRRIAFFTNSLFMDMPRA

PRVRNMLSFSVLTPYYKEDVVYSRDDLMTENEDGISVLFYLQKIYPDEWSNFLERVNITSADPETEIFGNVEKEDQLR

VWASFRGQTLSRTVRGMMYYRRALELQAFLDMATEDELVNGYKVITKTPDEQKRSQRSTWSQLQAIADMKFTYVA

ACQNYGEQKRQSHHNATEILNLMLQNPSLRVAYIDEVEERLKDKTQKVYYSVLVKAVNNLDQEIYRIRLPGPVRLGE

GKPENQNHAIIFTRGEGLQAIDMNQDNYLEEAFKMRNLLEEFHEPHGVRPPTILGVREHIFTGSVSSLAWFMSNQET

SFVTIGQRVLASPLKVRFHYGHPDVFDRLFHITRGGMSKASRVINLSEDIFAGFNSILRRGNVTHHEYIQVGKGRDVG

LNQISLFEAKIANGNGEQSLSRDMYRLGHRFDFFRMLSAYFTTVGYYFSTMIVVLTVYVFLYGRVYLSLSGVDNSLVH

SANNKALTAALGSQALVQLGLLMSLPMVMEIGLERGFRTALTDFLTMQLQLASVFFTFSLGTKTHYFGRTILHGGAKY

RATGRGFVVRHERFAENYRLYSRSHFTKAIELLLLLIVYTIYVSKSTSGAITYTLITFSMWFLVATWLFAPFLFNPSGFE

WQKIVEDWDDWNKWINNRGGIGVEGNKSWESWWDEEQEHLKYTGIRGRIIECLLSLRFFLFQYGIVYHLHIAEVSK

NLTISVYGLSWLVIVAVLAILKIVSMGRDKFSADFQLMFRLLKAMLFIGSVSVLAVLHFKSFTVGDFFASILAFIPTGWAL

LQIAQACKPIVVRIGFWESVKSLARGYEFTMGLLLFTPIAVLSWFPFVSEFQTRLLFNQAFSRGLQISRILAGRKKL

>Sphfa2-Sphagnum fallax 1938

MAASGGAEGHSSSPVIRRGLSRTYTTGQLTETFDSEVVPSSLSSIAPILRVANEIEAERPRVAYLCRYHAFEKAHRIDP

TSSGRGVRQFKTSLLQRLEKDNEPTLESRRRRSDAREIQSYYQQYYNQYVKALDGAEHSDRAALAKAYQTAGVLF

DVLKALNRDKTEEPPPEIIAAAADVEQKKEIYVPYNVLPLDAAGASQAIMQLDEIKAAVESLRNIRGLPWPRAAESRH

KSGDADCLDWLQDMFGFQKDNVANQREHLILMLANVHIRLLPRPEPMHKLDDRALNTVMNKLFKNYKSWCKFLGR

KHSLWLPQIHQEIRQRKVLYMGLYLLIWGEAANLRFMPECLCYIYHHMASELHGLLAGNVSLVTGDNMKPAYGGEDE

IFLRKVVTPIYNVISEETKKNRNGTASHSAWRNYDDLNEYFWKVDCFQLSWPMREDGDFFKPSAIIGRKFSLPAKLA

EKKVFNPTGKTFFVEIRTYWHLFRSFDRMWAFYILGLQACIVLAWNVGRDLRHAFNGHVITKVLSIFITASILRLIQAVL

DIVLSYHAYHSLKLLGILRLFLKLLTSAAWFIILTVCYVHTWSHPSGLIKDIQGWLGKSWESSYLYVAAVVVYLVPNIIGG

LFFLFPMLRRWIESSNWRIVRSLMWWSQPRLYIGRGMHESQFALFGYTFFWVLLLASKFAFSYYVQIEPLVAPTKRI

MQQRSIRYTWHEFFPHAKDNPGALISLWAPVVMVYFMDSQIWYAIYSTIYGGISGSFRRLGEIRTLGMLRSRFSSLP

GAFNENLVPTAEGKKSRRGFSFRRDFKKVLPQTERLKAARFSQLWNEVICSFREEDLISDKERDLMLVPYSSDLQLN

LVQWPPFLLASKVPIALQMARQAAETGRTADLLRKIRTDEYMKSAVVECYESFKRVLKLLIVGEVETRVIEGLLNQVQ

TNIDEDTLLLNFGLKELPVLSVKFIELLELLEKNDTDPKEVEAARDLAVLKLQDMYEVVTRDMMTESMRDMWESSHG

ALAREQGKEELFSAKGEKPAVLFPPPREEAWIEQIKRLHLLLTERESAMDVPENLEARRRIAFFTNSLFMDMPRAPQ

VRNMLSFSVLTPYYKENVVYSKDDLMTENEDGISVLFYLQKIYPDEWNNFLQRVHVNITDPETKIFGDVVLEDKLRE

WASFRGQTLSRTVRGMMYYRRALELQAFLDMATEDELVNGYKVITDAPDEQKKSQRSTRSKLQAIADMKFTYVAAC

QNYGEQKRQSHHNAAEILNLMLNNPSLRVAYIDEVEERRKDKTEKVYYSVLVKAVNNLDQEIYRIRLPGPVKLGEGK

PENQNHAIIFTRGEGLQAIDMNQDNYLEEAFKMRNLLEEFHEPHGVRPPTILGVREHIFTGSVSSLAWFMSNQETSF

VTIGQRVLASPLKVRFHYGHPDVFDRLFHITRGGMSKASRVINLSEDIFAGFNSILRRGNVTHHEYIQVGKGRDVGLN

QISLFEAKIANGNGEQVLSRDIYRLGHRFDFFRMLSAYFTTVGYYFSSMIVVLTVYVFLYGRVYLALSGVDNSLIHSAN

NKALTAALGSQALVQLGLLMSLPMVMEIGLERGFRTALSDFLTMQLQLASVFFTFSLGTRTHYFGRTVLHGGAKYRS

TGRGFVVRHERFAENYRLYSRSHFTKAIELLLLLIVYSIYVSRSTSGAVTYILITISMWFLVATWLFAPFLFNPSGFEWQ

KIVEDWDDWNKWINNRGGIGVEGNKSWESWWDEEQEHLKYTGICGRFIECLLAFRFFLFQYGIVYHLHIVEVSKNR

SISVYGLSWLVIVAVLTILKIVSIGRDKFSADFQLMFRLLKAMLFIGSISVLAVLHVRDFTVGDFFASILAFIPTGWALLQIA

QACKPIVVRIGFWESVKSLARGYEFTMGLLLFTPIAVLSWFPFVSEFQTRLLFNQAFSRGLQISRILAGRKKL

>Sphfa3-Sphagnum fallax 1974

MERPPRSNRISKRVLGNWERLVYARLRADEGRTSFNHEDYRRTTPETSGSSQAVPQSLSDQANIEAILQAADEVDD

VQVARLLCQYAFDLVQKLDPTSEGRGVLQFKTGLQSIIKRKQAARAGQQMDRNEEIILVQDYYTRYRAEHGIEQMEE

EFRLQQEGRLSGGEPDSMEKRARMMRKVYEISRILNDVVDYLLITAEPEAASRLQLTEEKRRLEEDAKKVKEYKTFN

ILPLERPGVSNAFSYFPEVTAATRAFFYTADLPIFPEEYERDERPLDLFHFLHYCFGFQRDNVANQREHLILLLASAQT

RMGFLPDTESSKLDDATINNVHQRILENYIRWCRFVRREPMSKRAFTQQRRLFLTALYLLVWGEASNLRFMPECLCY

IFHHMADELFDLLEQRYVERSKCVKPNADNIVEFSFLEQIITPVYQVVAAEAQSSGNGIAPHGAWRNYDDFNEFFWA

PNCFDLSWPWRLESGFFMKPLKPKSSWSFRRGPQKEEKPVVVPGRRKEHKVGKIHFVEHRTAFHLYHSFHRLWIF

FLCMLQGLTIWAFCSKNKTLNLHVRTIKKIMSVVPTFVVMKLFKALLDALFMWGAYRSTRLRIVVRMLLRCVWYAGM

SAAIIFLYVKTLQEDSSNTGSQKWFTIFYIVVASYAGTHLVLSVLFRIPWLRRQADKCSNLSIIQFIKWVQQERYYVGR

GLYERMRDYIRYLLFWIFLLACKFAFSFHFQIMPMVELTRVVINVKDIHYRWHDDISSSNHNALTLVALWAAVVLIYFVD

IQVWYTVISALLGGLEGAKDKLGEIRTLAMLRKRFASFPEVFVRHMQPPRLGSQMSSQMSPADGGAPAGSKAIKEK

VDAIKFAPIWNKVIKSLREEDLINNRERDLLIMPDNRISYTNQGPNLLIHWPLFLLANKVQITVELAAGHKIGSQTELLD

EIRRDEYMGFAVQEAFQGLEATLLSLLNHNGQRWVQRVFQCVKDNVENRNFVSRFRLSKLRDVLEKTRELTEQLGH

EETPDRRTKAKAALGLLFEVVWDDFIPTDLREEMVQKEPVFQHPASLLSDLHWPNEFRQAAAGRLNDLLAVQKIKD

AEGKTKTLDTESIPHNLEARRRLEFFTNSLFMHMPETPPTRKMHSFCVLTPYYEEDVMYSLDDLSKENEDGISILFYL

QKIYPDEWQNFLERIQLIENTLRRTVDQKKSEKHEETVMDLRLWASYRGQTLARTVRGMMYYKTALILQGQQEGAS

NTDVEQGLPLSMVQTHGANRSAQAQAELKFVYVVTCQIYGEQKKRGKAQAADILYLMQQNSSLRVAYIDIVETTKDR

KTATSYYSKLVKAGPSGKDEIIYSIKLPGKVILGEGKPENQNHAIIFTRGDAIQTIDMNQDHFLEEALKVRNLLEEFDCK
HGLRRPTILGVREHVFTGSVSSLAWFMSMQESSFVTLGQRVLARPLKVRMHYGHPDVFDRVFHITRGGISKASRVI
NLSEDIFAGFNSTLRQGNITHHEYIQVGKGRDVGLNQIALFEAKVASGNGEQLLSRDVYRLGQLFDFFRMLSFFFTS
VGFYVTTMMTVLTLYAFLYGKAYLALSGVDASLTSSSNVLESTALQATLNTQFLFQIGIFSAVPMIVNLILEEGILRAVISF
CAMQLQLASVFFTFSLGTRTHYFGRTVLHGGAKYRSTGRGFVVRHIKFAENYRQYSRSHFTKALEIIILLIVYLAYGAE
DRTAVTYILLTISSWFLALSWLFAPYIFNPSGFEWQKTVEDFDDWTGWLFYKGGVGVKIDYSWEAWWFDEQTHIRS
TRGRFWEFMLSLRFFLFQYGVVYALNVTQHSTAFSVYLYSWLVLIGLVIIFKIFSFSESISANFQLTVRTFQALLFTAIIAS
LVVTVKKTPLTIGDVFSMALAFIPTGWGLLNIAIAFRPWLEKTIIWKSVREVARIYDACMGMLIFIPVAFLSWFPFVSTFQ
TRLVFNQAFSRGLEISLILAGNRPNKNM

>Sphfa4-Sphagnum fallax 1963
MEKPASAKGSNRISRRVVGLWEQLVGEALRAQEARGSLEIGSSRRRDYGSSSYTVPQFLQDQTNIESILQAARDIEP
ENIQVSQILYEYAFTLVHQLDPMSEGRDVLQLKTGLSSMISKKRMQREGQKLDRTDDIQVISDYYKKYRERLDIANLE
DEERKQQQESTSSGGRPESQEWRSERLRRAYEIANILQAVIDSLLSEADPVELAKLDLASGKVILEEDAKKIRKRRAY
NILPMEEDGVSNPFSYFPEVTGATKALLYTEGSPRFPYDYVMPENRNLDVFDFLHYVFCFQKDNVANQREHLILLLA
SSQSRKGVLHDGHRTEGSAVNEAAVDDVYERVLGNYMRWCDFLLKEPKAKKAKDATQQEKIYLIALHLLIWGEAAN
LRFMPECLCYIFHHMAHDMFELFRKEEVEWSSQTAKPSEDGSRELCFLEQVITPVYQIVAAEAHNNGNGIAKHSAW
RNYDDFNEFFWQPDCFDHLSWPWKEDAAFFMKPKKRSYDDNDKTVQHAEPAALLSQTRGRRVGKINFVEHRTGF
HLFHSFYRLWIFFICMLQGLTIWAFCNQNFHVRTIKKILSIAPTFAFLTFFQSVLDVLLMWGAYRSTRHHIVMRMLIRLV
WFGALSGGVIFLYVKTLLEDRQGTGSTIWFRIFYLVLGSYAILHLLIGMLSHIPWLRIRTAEWSKYRVIRFIKAVHQERY
FVGRGMYERLWDYFRYVLFWVFVLFCKFSFSYHFQLLPMVVPTRLIVELNNINYVWHDFVSKNNHNALTLLALWAPV
VMIYFLDVQVWYTVTSALLGGLEGAKDRLGEIRDLSMLRKRFIDYPQALVQRLQPMNSSRTSRQISLAEGAATDGKV
IRSKQDAINAIKFAPIWNEVIKSLREEDLINNKEKELLIMPEQDVMQQNSWRIHWPLFLVANKVHVAVELAAGNKKNLE
LLWEKVNRVEYMANAVEEAFETLQPVLENLLNADGAQWVRSLFGDIKEAIGSQAFVANFNLTNLREILKMTRKITKQL
WRNKTAERVSKVEAALQRLQAVVMNGFLPQDIREGFERWNRSEKPLFTNLNWPDRNGQKDAKRLFNLLTVQKINA
SKTLDTETIPRNLEAQRRLQFFTNSLFMHMPEAPTIRKMFSFCVFTPYYAEDVMYDLKKLCEENKDGISILFYLQKIYP
DEWQNFLERIGLTGRTVDTKVDEKNEEVILQLRLWASYRGQTLARTVRGMMYYKRALELQAAQEGASTADVEEGL
QLSEGLLERSAKHQAELKFTYVVTCQIFGEQKKQGAVQAADILYLMHKYDSLRIAYIDVVETMKDKKVTKSYYSKLVK
ADPYGQDQEIYSIKLPGEVKLGEGKPENQNHAIIFTRGDAIQTIDMNQDNYLEEALKVRNLLAEFDREDISLRPPTILG

VREHVFTGSVSSLAWFMSMQESSFVTLGQRVLARPLKVRMHYGHPDVFDRIFHISRGGISKASRVINLSEDIFAGFN

STLRQGNVTHHEYIQVGKGRDVGLNQIALFEAKVASGNGEQSLSRDVYRLGQLFDFFRMLSFFYTSVGFYVTTMMT

VLTLYVFLYGKAYLALSGVDASLQADNNIIQNAALQSALNTQFLVQIGIFTAVPMIMNLILEQGILRAIISFCTMQLQLASV

FFTFSLGTRTHYFGRTILHGGAKYRSTGRGFVVTHIKFAENYRMYSRSHFTKAMEIIMLLIVYLAYGAEDRKAVNFILLT

FSSWFLALSWLFAPYIFNPSGFEWQKTVEDFEDWTKWLYYKGGVGVKIENSWEAWWFDEQTHIRTNTSRFWEIILS

LRFFIFQYGIVYHLHVDEHSTRLNVYGFSWLVLVVIVVIFKLFTFTRKSPKVQRLLRLFQTLLFMSIVTGIIVAVVLTPLTI

GDVFAVGLALIPTGWGLLSVAIACRPAVKGLRLWESVQEIARAYDACMGMLVFIPIAFLSWFPFVSTFQTRLVFNQAF

SRGLEISLILAGNRPNSSV

>Sphfa5-Sphagnum fallax 1963

MERPPRVPNLISKRVLQKWEDLVRAALRAEEPKGSFVGDEYGFGYESSASSAAVPQSLSQQTNIDSVLRVADEIHPE

NPQVARILCEYAYSMSQNLDPRSEGRGVMQFKTGLKSIIEQNRAKKGGEKIDRPNDIHLLQEYYNKYREMNKIVELE

EIGSSQRYEDSDRSAARAEQKRKVYEKARILNEVVDAYRRESPEETVDHNLKKQMENDAEKIREFKPFNILPLETPG

VVNAFQMFPEVTAATGALEWSTPPFPEFPYGYERPERALDVFDFLQYAFGFQEDNVANQREHLILLLANAQSRLSS

VSLTSQHSKLDGAVVTHVHSKIVENYERWCLFLRKKSQVKSGTVEHKVCIMALYLLIWGEAANIRFLPECLCYIFHHM

ADEMSDLLEKPRVERSEKIYIEGSQHSFLEKIICPIHEILAAESDVPAHGRAAHSAWRNYDDFNEFFWAPSCFELSWP

WRLEAGFFLKPKKDVDTDDDDVPEQSFTAGRQRERKLGKTHFVEHRTFLHVYHSFHRLWIFLVCMLQGLTIFAFCN

QKLDTHSIKYILSVGSTFIVMRFVQCVLDVILMFGAYRSTRGRTVVRMLIRFFWFALLSAVIVFIYVKVLIEESKTPAKDT

WFTLYYLILGVYGGIQLFLSLLFHIPFLRSQADRCSDVSFLQFFKWVKEERYYVGRGMYEKTSDYVKYTMFWLLIGIG

KFAFSYFLEIKPMVKPTRIIIGLHNIQYRWHDLISKSNHNALTLLSLWAPVVMIYFLDTQVWYTVMSALVGALEGARMG

LGEIRSLDTLRQRFTTFPEAFVKHMQPTKTSEIPRSISMGAEAVKEKENAIRFAPLWNEIIGCLREEDLISNREKLLLM

MPDNKITTSRTHPQQSLVQWPLFLLANKVYVAKDILSETKYATAQDELWERMKNDLYLAYAVQEAYESLQVVLSSLLN

EDGHHWVEDVFREIDLAIEKGEFGIEIDQNFRKFDLKKLGTVLDKTAKLTAVLVKEAEKKDEKEREQEISEMHSAARR

ALVDLYEVVMHDFITDQNLRATCGNAAHLAAKQKGQLFSGLTWPSDKEKTLVSRLHYILSIKDSALSVPSNLEARRRL

QYFTNSLFMKIPESPSVRKMLAFSVFTPYYEEDVMYSLVQLNKKNIDGITTLFYLQKIFPDDWTNFKERMLPLVKEDD

LYKKTEDDIKDTRELRLWASYRGQTLARTVRGMMYYKRALILQAAEEGAFKTDFLDEEQGLASPMTSQGASRRSAQ

AQAELKFTYVVSAQIYGSQRSSSKKEDQEKAADISFLMQKNDSLRIAYIHVVKKGKDGKQSEYYSKLVKADPSGNDQ

EIYSIKLPGKFLLGEGKPENQNHAIIFTRGDALQTIDMNQENYMEEAFKMRNLLEEFDSDKHGRRRPTILGVREHVFT

GSVSSLAWFMSLQESSFVTIGQRVLARPLKVRMHYGHPDVFDRLFHISRGGISKPSRGINLSEDIFAGFNSTLRQGNI

THHEYIQVGKGRDVGLNQIAAFEAKVASGNGEQTLSRDVYRLGQLFDLFRMLSFFFTSVGFYVTTMMTVLTIYAFLY
GKVYLALSGVEASLETAGNILNNAALQTALQGQFLFQIGVFTAVPMFMNFLLEQGVFTAVISFCTMQLQLSSVFFTFSL
GTRTHYFGRTILHGGAKYRSTGRGFVVEHIPFAENYRLYSRSHFVKAMEIIMLLIVYRAYGAQSRTTVSYILLTFSSWF
LAISWLYAPYIFNPSGFEWQKTVKDFEDWTNWLFYKGGIGDKGLKSWEVWWNEEHAHIQTTRGRIWECILSFRFFII
QYGVVYALHVTGRDKNFNVYGFSWVVLAGVLLLFKGFTLSKKASANFQLLVRLFQGVLFLACIGGLAFALADTALTV
GDMFAAGLALIPTGWGLLSIAIAVRPVVEKVGLWKSVRGIARLYDAFMGMVIFIPIALLSWFPFMSTFQTRLVFNQAFS
RGLEISVLLAGDQPNASF

>Sphfa6-Sphagnum fallax 1976
MATPSRGGGGNRVSKQHISKRVLNRWETLVYEAMMAEKRRQSARQGGGGDDDSFGGGDTTTSSAALVPQSLQQ
QRNIDAILRTADDVQQVDPQVARILCEYAYSLSQHLDPKDEGRGVLQFKTGLLSIIKQKRSKKEGEKIDRSQDIRHIQA
FYRSYRERNRVDQVEEEVRRGLERQSSDQDTSTLETQVENLRRVYLMAKILNEVVDALHSANSDMTIDLSLKAVME
NDAKKLQEYKAYNILPLETPGVTNSFQLFPEVSGATRALEYTTNLPKFPDTYNMPKERALDVFDFLHYVFGFQNDNV
ANQREHLVLLLANAQSRLGVLVDSEQNKVDIGAINSVHSRLNENYDRWCKFLRRDSMAERAFTPQLQLFLTALYLLI
WGEAANLRFLPECLCYIFHHMADELYDLLDKPTVGKSRIIVPDSPHSFLDRVIKPIHEIVVEESTIGADGRSPHSAWRN
YDDFNEFFWAPSCFEISWPWRSNAGFFKKPNKLIYSEADRFEPAKPEQEEAVDEERKVGKTHFVEHRTGLHLYHSF
HRFWIFLVCMLQGLTIFAFCNQELHIRSLKYIMSVGPTFIVMKFLQSFLDVTLMLGAYRSTRARNVSRMLIRFFWFASL
SAAILLLYIKMLQEEARSPSSNKWFRAFYWVLGIYAGFQLFLALVLRVPLFRMQLDRCSNLYIVQFVKWVHQERYYVG
RGMYESTLDYFMYTSFWWVIGVCKFAFSYFLQIKPLVEPTRIIVNTSNIHYRWPDKISASNHNALTLMSLWAPVLMIYF
MDAQIWYMFISAIVGGLDGAGKRLGEIRTLDMIRQRFSTFPEACVKCLQPSRHGLAMTRSISIGGQTTKAKVDAIKFA
PLWNAIINCLREEDLISNREREWLQMPDNSITTGTEAQHSLVQWPLFLLANKVYVGVDIVNEYRQSTQEELWERISR
DPYLVHAVQESFEDLQPILLALLNDAGRDWVRQVYEDVQNSITNGQFVIQFKLSALGLVLKSTTVLTEILAAELKKKED
KERKEEEAKNCDAAEQALMDLYEVVMQDFMFDPDLRYKYANLLDLQAAKQDGRLFSDLKWPTEQEKADVDRLNYI
LTIKDSALNVPRNLEARRRLQYFTTSLFMTMPAPPAVRKMFSFSVFTPYYDEDVMYSIDQLTTPNVDGITTLFYLQKIY
PDEWTNFLERQLPGVDRAQLASRTENDVKDSQQLRLWASYRGQTLARTVRGMMYYKRALELQGSQEGASTMDE
EEGLPVDRPGGLVRSAKVQAELKFTYVVSCQSYGEHKNSPLYQDKVADIAYLMRNNDSLRIAYVETVKEMKDGRIAT
MTYYSKLIKADPNGGNQEIYSIKLPGRVFLGEGKAENQNLAIIFTRGDALQKIDMNQDNYLEETLKMRNLLEEFDSKK
HGLRSPNILGVREHVFTGSVSSLAWFMSMQEITFVTIGQRVLARPLKVRMHYGHSDVFDRLFHISRGGISKASKQIN
LSCDTFTGFNTTLRQGNITHHEYIQCGKGRDVGLNQIAAFQAKVASGNGEQCLSRDIYRLGQLFDFFRMLSFFFNSV

GFYVTTLITVLTVYVFLYGKVYLALSGIEDALKKNKDNILGNKALQAALDTQFLFQIGLFAAVPMVMNFILEQGVSRAVI
SFITMQLQLSSIFNMFAVGTRAHAFSRTILHGGAKYRSVGRGFVVEHIPFTENYRQYSRSHFVRALEIIFLLIVYLVYGS
QDQGAVLSVLLTYSSWFLALSWLYAPFIFNPSGFEWQKTVQDFSEWSNWLVRKGGIGDEGKMSWEIWWNEEQAHI
QTFAGRFWEVVLSLRFFIFQYGVIYTLNVARNDINARVYGYSWLVMIGIFILFKVFTISPKLSANFQLVIRLFQGLVFLGL
VAGISVAVSLTSLSVRDVFASLLALIPTGWGVLSIGIAIRPILESRALRWAGLWNSVRAIARLYDAAMGTLLFIPIASLSW
FPFMSTFQTRLVFNQAFSRGLEISVLLAGTRPNTGL

>Sphfa7-Sphagnum fallax 1759
MESPAYNILPLEGPAMIENAAMNFPEVRAAIAALRAVGELSIPPELMWNPDMDLLDWLGALFGFQRDNVRNQREHLV
LLLANAQMQLYQDGMSWDKLDENMIKRIRKKVTENYISWCRFVRKKHNLRGLDGKHQPFNGSRELLYTSLFLLIWG
EAANLRFMPECLCFIFHNMANELNKMLDEDPTVRPVYGQPNGFLRGVVAPLYDVVKAETESNNNGTAPHSSWRNY
DDLNEYFWSSRCFTQLRWPLDRHCSYLVEPRKVKFPRRRMEKKIGKTGFVEQRSFWNIFRSFDRLWIGYLLVLQAC
VVTLWHGGGPPWIELQKRDSLARFLSIFISWAILRLLQAVLDIGSQYSLVTRETFLIGVRMFLKVLVATAWVILFIIYYRIM
WKQRHLDHYWSTPANDRLITFLYVAAAFCVPEVLALVLFVVPWLRNFLENSSWRIFHVLTWWFQTRAYVGRGLREG
VADNIKYTLFWLCILASKFAFSYFLQIRPLIAPTKQILRTTNVTYKWHEFIPDGNRAALIALWAPVVLIYLMDTQIWYSIW
SSMVGAFVGLLQHLGEIRNVEQLKLRFQIFPSAFQFVLMPREDAVVRDTWWAGAKDVMKRLSLRYGWLSPYEKVE
WGQIEGGRFAHVWNQIIQIFREEDLISDRELELLEIPSEPWRRLTVFQWPSTLLANQVLLALEEVKYSRGDDAAVWKII
CKTEYRRCAVIECYETMKHVMRRIIKNDSYEFAIFSSIFEEIDASRIQGRFTSTFSLRALPEVHARILDLIDVLLKKPTSK
DLQKVVDALQNLYEELVQDFPYDSSVLENLREHPSTSQSDAEVLLFMEAVDLPASDEEAFFKQLRRLHTSLSTTDAL
LNVPKGLEARRRISFFCNSLFMTMPRAPQVEKMLAFSVLTPYYSEEVIFSKQQLKEENEDGVHIVFYLQKIFPDEWD
NFLERMNKIPLNESKLWDDDAFELRMWASYRGQTLARTVRGMMYYERALELQAFLDTAAEVELLGIRDLLERGSSM
NSMGSMGSMGSIGSIGSDNEATELNRVKKKEMDLANAAMKFTYVVTCQIYGAQKIQKDNRADDILHLMKTYKALRIA
YVDEVRTGRESKKFYSVLVKYDPVKQREVEIYRIELPGPVKLGEGKPENQNHAIIFTRGDAVQTIDMNQEHYFEETIK
MRNLLQEFRTFHGIRKPTILGVREHVFTGSVSSLAWFMSAQETVFVTLSQRVLANPLKIRMHYGHPDVFDRLWFISR
GGISKASRTINISEDIFAGFNCTLRGGTVTHHEYIQAGKGRDVGLNQIAMFEAKVASGNGEQVLSRDVYRLGHRLDF
FRMLSFYYTTVGFFISNMIVVLTVFVFLWGRVYLALSGIEASLTTGKNALANKALTAALNQQLVVQLGLLTILPMVVEIA
LEHGFTTALWDMITMQLQLASVFFTFSMGTRMHYFGRTLLHGGAKYRATGRGFVVKHEKFAENYRLYSRSHFTKGI
ELILLLVVYEAYGSATSSATYILVTITSWFLALTWIMAPFVFNPSGFDWLKTVEDFDDFMQWLWFKGDIFVKVEQSWE
VWWEEEQSHFRTTGMWGKLLEIVLDLRFFLFQYGIVYHLRIANGSKSIFVYLISWSYMLVAGGLHYVLSSANERFAA

KRHGLYRAIQAIVIVLIVAIIVVLLFVTNFRVLDLITSLLAFIPTGWGILQICLVLRRPFLDNSSIWGTVKAVARLYDLGMGM
IVMAPVAILSWLPGFQAMQTRILYNEAFSRGLQISRLLVGKKSGRIGN

>Sphfa8-Sphagnum fallax 1756
MANSVYNILPVDNPLADHAALLFPEVRASIAALRTVGELRRPNGVRWTHDMDLLDWLGAWFGFQEGNVKNQREHL
VLLLANAQMRLQPQATNRLDAKVVKKIRKKVTANYVSWCNFIRQRSNLELTEEDERLELLYTALFLLIWGESANLRFM
PECLCYMFHHMSRELNRMLNRSIDEHSAMPAKPKYSEPNDFLNKVVTPLYKAVQAEAKVNKDGKAPHSQWRNYDD
MNEYFWTNRCFKRLGWLEESSNYLVAPGSGTKHKVGKTGFVEQRSFFNIFRSFDRLWIGHILVLQAIIVTLWSGTGA
PWIELQNRDSLARFLAIFITWAALRFFQAFMDLCMQHSLVSRDTLLIGVRMVLKLLVAAGWIVIFTVFYRDMWKQRHH
DHAWTKAANKYLIQYVEAAAVFVIPEVLALLLFIIPWLRNAIENSSWRIFHILTWWFQSRIYVARGLREGLFDNFKYTLF
WVLVLASKFAFSYFLQIKPLIKPTKEILRITDIQYKWPQFFSHGNRAAVVALWAPVILIYFMDLQIWYTIWSSFVGALVGL
FEHLGEIRNVLQLRQRFQIFPSAAQFNLMPEEVALPPQFFRSWWVYVKAVFTRFKLRYGWAVGGIKEDSKQLEAGR
FAKMWNHIVDIFREEDLISDSELELLEMPSTQHISVFQWPSTLLANEVRLAVEQVQKHKGNDKSLWKKICANDYRRC
AVIESYETIKFVLQEIVRENTTEYQRFLSIYEEIDMSIRQSKFTTTFKLKELPKVHEAVLELVKDLLAWPTHNDPVKVVV
RLQNLYEVVLCDFTKDMLVETKIRDSAPYEHQANGQDSELPVELPSADNHSFFKQLKRLQTTLSTKESLFNVPKGLE
ARRRISFFSNSLFMTMPRAPSVDKMLAFSVLTPYYKEDVIYSMKALSQLNEDGVSILYYLKTIFADDWQNFKQRFPNP
DLKPDQLENDTTFIARMEALDDAIEIRMWASYRGQTLARTVRGMMYYERALEFLAFLDTASESEVLGFKELVVRSSS
LSREGSLSRHKASTSDSQPLDTQAAQNALLATAAMKFTYVVAAQEYGRQKVDKDDRAWGISWLMRTYKGLRIAYVD
KVEVGSESHCFSVLVKYDPIIQEEVEIYRVRLPGPFIGEGKPENQNHAIIFTRGDALQTIDMNQEMYFEEALKMRNLL
QEFNKYYGIRKPAILGVREHVFTGAVSSLAWFMSAQETSFVTLGQRVLANPLKVRMHYGHPDVFDRLWFLSRGGIS
KASKTINISEDIFAGFNCTERGGTVTHHEYIQAGKGRDVGLNQISMFEAKVSSGNGEQVLSRDVYRLGHHLDFFRML
SFYYTTVGFFINNMIIVLVVYAFLWGRVYLTLSGVEASLSNTSDALHNTALTAALNQQFLVQLGLLTALPMIVENALEHG
FLRALWEFFTMQLQLASVFFTFSMGTRAHYFGRTLLHGGAKYRPTGRGFVVKHESFAVNYRFYARSHFTKGIELILL
LIVYETYGSFHSSVTYVLITVTSWFLALTWILAPFIFNPSGFDWLKTVEDYDDFMSWLWYKGGIITDPEQSWEAWWL
EEQGHLRTTGFWGKVLDIVLDLRFFFFQYGIVYHLHIASNHHSIAVYLASWSYVVVAGVINSILSYAHEHYAARRHRLY
RAIQAIVLIVVVTVITVLLIETPFKLLDLLTSLLAFVPTGWGILQICLVLRPLLERYTWAWGVVVAVARLYEMFIGLIVMAP
VAILSWLPGFQAMQTRVLFNEAFSRGLQISRIFAGKKNPDSWKAQ

>Sphfa9-Sphagnum fallax 1151

MVVRLFRSWWVYVKAVFTRFKLRYGWAVGGVKEDSKQLEAGRFAKMWNHIIDIFRELGLISDSELELLEMPSTQHIS

VFQWPSTLLANEVRLAVEQVQKHKGNDKSLWRKICANDYRRCAVIESYETVKFVLQEIVRENTTECQRFLSIYEEIDM

SICQNRFTTTFKLKELPKVHEAVLELVKDLLAWPTHNDPVKVVVRLQNLYEVVLCDFTKDMVVETKIRDSAPYQHQA

NGQDLELPVELPNADNHSFFKQLKRLQTTLSTKESLFNVPKGLEARRRISFFSNSLFMTMPRAPSVDKMLAFSVLTP

YYKEDVIYSMKVLSQLNEDGISILYYLKMIFADDWQNFKQCFPNPDLKPDQSENDATFIARMEALDDAIEIRMWASYR

GQTLARTVRGMMYYERALEFLAFLDTASESEVLGFKELVIRSSSLSREGSLSRHKASTSDSQPLDTQAAQNALLATA

AMKFTYVVAAQEYGKQKIDKDDRAWGISWLMRTYKGLRIAYVDKVEVGSEIHYFSVLVKYDPVIQEEVEIYRVRLPGP

FIGEGKPENQNHAIIFTRGDALQTIDMNQEMYFEEALKMRNLLQEFNKYYGIRKPAILGVREHVFTGAVSSLAWFMSA

QETSFVTLGQRVLANPLKVRMHYGHPDVFDRLWFLSRGGISKASKTINISEDIFAGFNCTERGGTVTHHEYIQAGKG

RDVGLNQISMFEAKVSSGNGEQVLSRDVYRLGHHLDFFRMLSFYYTTVGFFINNMIIVLVVYAFLWGRVYLTLNALHN

TALTASLNQQFLVQLGLLTALPMIVENALEHGFLRALWEFFTMQLQLASVFFTFSMGTRAHYFGRTLLHGGAKYRPT

GRGFVVKHESFAVNCRFYARSHFTKGIELILLLIVYETYGSFHSSVTYVLITVTSWFLALTWILAPFIFNPSGFDWLKAV

EDYDDFMSWLWYKGGIITDPEQSWEAWWLEEQGHLRTTGFWGKVLDIVLDLRFFFFQYGIVYHLHIASNNHSIAVYL

ASWSYVVVAGVINSILSYAHEHYAARHHRLYRAIQAIVLIVVVTVITVLLMETPFKLLDLLTSLLAFVPTGWGILQICLCA

EASFGEMFIGLIVMAPVAILSWLPGFQAMQTRVLFNEAFSRGLQISQIFAGKKNPDSWKAQ

>Selbr1-Selaginella bryopteris 1933

MAANKLQRTFTTGTIPTEVDSEVVPSSLAPIAAILRVANEVEADGHPRVAYLCRFYAFEKAHEQDPTSSGRGVRQFK

TALLQRLEKDDEASKKERKERTDPREMQHYYQEYYDKYVKALEGNTDRAALARAYQTAGILFEVLSSVTKMDQGEE

IDMQKMNKMDAEVKQKKEAIKHYNILPLDEAGSSQAIMKLPEVRAAHDAIKNVRGLPPNRKNKSASDILEWLQFMFG

FQEDNVANQREHLILLLANVHISLDPEPIPLGKLDQRATDIVMKKMFKNYKTWCKFLGRNDKLELPEIQLEVQQRKILY

MALYLLIWGEAANVRFMPECLCYIFHHMASEVAGMLSGNVSYITGENIKPAYGSEPEAFLHKVITPIYNIIAKERDSKD

RPHSSWRNYDDLNEYFWSENCFRLGWPMRSDDEFFRGPEQMNTGASTPISVKTVVQYFVGGGPHPRPLKKTGF

VEVRSFWHLFRSFDRMWTFYILWLQAMIIIAWNGSGSIRAVFEGAVFKKVLSVFITAAALRFFQAVLDIIFNFKAIQSLG

YAGSIRLVLKLVVSVFWIVILSSSYVHSWEHPTGLTRTIKNWFGHSSGPSVYLVAVILYLVPNAIAAIFFLMPFISRYAEE

SDALPIKVLLWWSQPRCYVGRGMHEGTVDLLSYTFFWLVLITCKLLFSYYVEIKPLVGPTKTILDFHRVEYRWHEFFP

NARGNIGAVLALWAPVILVYFMDIQIWYSVISTIWGGVIGAFMRLGEIRTLSMLRSRFRSLPTTFNLNLIPEEKAARRKM

QILKKFEPFVPEGNDRIQEARFAQLWNKVIETLRSEDLIDTKEMELMLLPYSTDPYTTKNVVQWPPFLLASMAPMAIE

MAKDFAERGETDDKELWSKIRGNEYMRCAVEECYEFLKNILDYIVTGETERRLIKGLTDAVEEKRKDGEVVRNFRMT

ALPLLTRQFVQLLQLLEEKKDDKEKDRVVFFLQDMLEVFMHDMMIDDARMSFESSHGIAPKNTESQNQKVQRESEY
FAGKDAIKYPLPNDHAWSEQIKRVKLLLTETEQAMDVPKNLEARRRLTFFTNSLFMRIPPAPRVRKMIPFSVLTPFYD
EEVVYSSNTIKEENEDGVSILFYLQNVYPDEWKTFLERVNCETEEEVSEEDLREWASFRGQTLSRTVRGMMYYRTA
LELQAFLDMANDDDIRTGFKEVSERRKTEKGQDSFWAKLDAIVDMKFTYVVACQQFGHHKHSKIAAEASKAHDIQKL
MTKYRALRIAYILEDEPPKDKEKHTGKTQKLYYSVLAKSVESRDEEIYKIKLPGPVVIGEGKPENQNHAIIFTRGQGLQ
TIDMNQDNYLEEAYKMRNLLEEFKSKHGVRMPTLLGVREHIFTGSVSSLAWFMSNQETSFVTIGQRVLANPLKVRF
HYGHPDVFDRIFHITRGGVSKASKVINLSEDIFAGFNSTLRRGLVTHHEYIQVGKGRDVGLNQISTFEAKVANGNGEQ
TLSRDVYRLGHRFDFFRMMSFYITTVGYYFSTMIVVLTVYFFLYGRLYLALSGLERSLVNVATLNSDSALQSALASQSL
VQLGLLMALPMVMEIGLERGFRSALSDFIVMQLQLASVFFTFTLGSRVHYYGRTIFHGGAKYRGTGRGFVVKHEKF
SENYRLYSRSHFVKGFELMMLLIIYSVYGTSQQKNAVTYVLITFSMWFLVGTWLFSPFLFNPSGFEWQKVVEDWND
WNKWIYSKGRVGVPANKSWESWWDEEQGHLRSTGLIGRLLEVILAFRFVLYQYGLVYHLNIVQGNKSLSIYGLSWL
VIAVVLVILKIVSFGRKRFKANFQLVFRMLKGIIFIGFVSVIVVLFVFVHLTVGDLFASVLAFMPTGWGLLQIFQACRPIVE
DYGMWDSVQALARTYEYIMGLVLFAPIAVLAWFPFVSEFQTRLLFNQAFSRGLQISRILAGRRKRVADD

>Selbr2-Selaginella bryopteris 1930
MASSRTVDPGKQSRVYTSGVFSGEVIDSEVVPSSLTSIASILRVANDVEAERPRVAYLCRFYAFEKAHRLDPTSSGR
GVRQFKTALLQRLEKDNERTLQQRIRRSDAKEIQSYYQQYYEQYVKALDKPEQTDRAQLAKAYQTAGVLFEVLCAV
NKTEEVAPEIIALGKDVKEKKELYVPYNILPLDAVGSSQAIMQLSEIKAAVDALKNIRGLPWPAVLSDQPHKAGDVDILD
WLQKMFGFQKDNVANQREHLILVLANAHVRFAQKSELSTKLDDRALNEVMLKLFKNYKKWCKFLGRKHSLVLPEVH
QDAQQRKLLYMGLYLLIWGEAANLRFMPECLCYIYHNMALELHGMLAGNVSFVTGDYIKPAYGGDEESFLRKVVTPI
YEIIEKEARHSKNGTAPHSTWRNYDDLNEYFWSSDCFRLGWPMRADADFFVPPSRPPTQGISKKPASHRLNKTGF
VELRSFWHIFRSFDRMWTFYILCLQAMIIISWNGTGSLSQIFQPDILRQISSIFITASVLRFLQGVLDVIFSLKVYHSMRF
TSMLRLVLKLIVAATWVVILLILYVHTWDNPKGLIGSIRNWLGHRWKNPSLYIAAVIVYVSPNALGAVFFVFPAIRRWIEN
SNWRIIRFLLWWSQPRLYVGRGMHESQWTLFKYTFFWVLLISSKLAFSYFVQIRPLVRPTKDIMNTRNVTYTWHEFF
PNAKKNILAVISVWVPVILIYFMDTQIWYAVYSTLFGGISGAFRRLGEIRTLGMLRSRFQSLPGTFNEYLVPAREPRER
GFTGMFSRLPRQSQSTQGKEAAKFAQLWNEVISSFREEDLISNRDMDLMLVPYSSIGHVQWPPFLLASKIPVAIQMA
EQLEQHKEDHELWKKIEADNYMKDAVMECYDAFKRVLHALVASDSDEKRVIEAIFDKVDQLTESKEFLINFRMSALRS
LTDKFVALITNYLINPDPGSRHSVSMLLQDMYEVVTKDMIDDDLWDEMESQLFISKQTQTQAPNVDPANAPRELFGIR
SIQYPPQHNPAWVEQIKRLQLLLTVKESAMDVPTNLEARRRINFFTNSLFMGMPRAPRVRDMLSFSVLTPYYTEEIVF

TKEQLYDENEDGVSILFYLQRIFPDEWDNFLERRKCKSETEILHDEGHMLELRKWASFRGQTLSRTVRGMMYYRRA
LELQAFLDMASDPEIFEGYKVVTNSAEEAKRSQRSLWAQLQAVADMKFTYVATCQNYGAQKRSSDSRATDILNLMIQ
YPSLRVAYIDEVEEREKDKIKKVYYSVLIKAVDGLDQEIYRIKLPGPVKLGEGKPENQNHAIIFTRGEALQTIDMNQDN
YLEEAFKMRNLLQEFSRDHGVRPPTILGVREHIFTGSVSSLAWFMSNQETSFVTIGQRVLANPLKVRFHYGHPDVFD
RLFHITRGGVSKASKGINLSEDIFAGFNSTLRGGNITHHEYIQVGKGRDVGLNQISLFEGKVANGNGEQTLSRDVYRL
GHRFDFFRMMSCYFTTVGFYYSTLLVVFTVYAFLYGRLYLAVSGLEKSLMRTADLNNDVPLEAALASQSLVQLGALM
AFPMVMEIGLERGFRTAFSDFIVMQLQLAPVFFTFSLGTRTHYYGRTILHGGAKYRPTGRFFVVYHEKFAENYRLYS
RSHFTKGLELLMLLIVYNVYGSSAKGSLAYLIVTFSMWFLVATWLFAPFLFNPSGFEWQKIVEDWEDWNKWINSKGG
LGVSASKSWESWWEEEQEYLDHTGFVGRILEIILSLRFFLYQYGLVYHLHVTDGRKNISIYGLSWLVILAVLTVLKIVS
MGRKRFSGDFQLMFRLLKAMLFIGFISIIIILFVVVGLTVGDLFASILVFMPTGWALLQIGLACKPIILKVGFWDSIRALAR
TYEYVMGLALFAPVAVLAWFPFVSEFQTRLLFNQAFSRGLQISRILAGRKKKGSSSSSSTH

>Selbr3-Selaginella bryopteris 1907
MPAAPSVDSTGSSGRRLSRTYTSGMFSGEVLDSEVVPASLAPIATILRVANEIEPERPRVAYLCRFYAFEKAHRLDPT
SSGRGVRQFKTALLQRLEKDNERTIRQRVKDSDPKEIQSFYQRYYKQYVKSLDGAENTDRAKLAKAYQTAGVLFEV
LCAVNKTEEVAPEIIATGQDVKEKEEIYAAYNILPLDAAGSSQAIMQLPEIKAAVEALRNVRGLPWRTTTKEADILDWLK
EVFGFQKDNVANQREHLILLFANVHVRAQTKAESVNRLEDHAINEVMRKLFKNYKSWCKFLGRKNSLIMPQPQIEHD
VHQRKLLYMGLYLLIWGEAANLRFMPECLCYIFHNMALELHGMLAGNVSFVTGDNIKPAYGGDEESFLRKVVTPIYDI
IQKEAANNNGGTAPHSAWRNYDDLNEYFWSEDCFRLGWPMRANADFFVAPSPSNSFQETKHVSNRLNKTGFVEIR
SFWHIFRSFDRMWTFYILALQVMIILAWNNVKSPSDVFKEEMLKKLSTIFIVASALRVVQSLLDILFTFKAVQSLKLTGM
LRPILKLVISLAWVIALSTLYAHSSKSAGFLKNIGNWLGNNWKSPSLYTAAVIVYLLPNLLGAVFFVFPLLRRLIENSNW
RIVRFMLWWSQVPLYVGRGMHEDQITLFSYTMFWVFLIACKFSFSYFLQFQPLVKSTKKIMKVKDITYTWHEAFPHA
KNNYGVVICQWAPNILIYFMDTQIWYAIVATAVGGLIGAWKRLGEIRTLGMLRSRFRSLPAAFNENLIPVDKARRKPFS
FVRKYEQPGNDRKHAANFSQLWNDVILSFREEDLISNREMNLMMVPYCSDPNLSIVQWPPFLLASKIPVALQMAAQ
YRGRDTIDLWKKIKADDYRHCAVEECYEAFKAVLKTIIRSEPEKGIIDAIIREVKEHIDRNTFLIYFRLNALPSLSSKFVRL
VELLIEPDPKSRDTVILLLQDMFEVVTRDMMAEEIREGVVDPKTKTNNWNQLFFNVVYPPPASPGWVEQVNRLHLLL
TVKESAMDVPINLEARRRIAFFTNSLFMDMPRAPKVRKMLPFSVLTPYYSEDIVFTKEQLHLENEDGVSILFYLQKIYP
DEWDNFLERIECKSESDIWGNEESEMQLREWASFRGQTLSRTVRGMMYYRRALELQAFFDIATDDEILEGYKAIATS
ASEDKRSQRSVYAQLQAIADMKFTYVATCQIYGVQKRSGDPRGTDILNLMVKHPSLRVAYIDEVEEMQKDNKSKKVY

YSVLVKAVNGLDQEIYRIKLPGPAKIGEGKPENQNHAIIFTRGEALQTIDMNQDNYLEEAFKMRNLLEEFHQDYGVRP
PTILGLREHIFTGSVSSLAWFMSNQETSFVTIGQRVLACPLKVRMHYGHPDVFDRLFHITRGGISKASRVINLSEDIFA
GFNSTLRRGNVTHHEYIQVGKGRDVGLNQISLFEAKVANGNGEQALSRDIYRLGHRFDFFRMMSCYFTTVGFYVSH
LIVVLTVYVFLYGRLYLALSGMDKSLMKAAIKRKDINLQSALAQQSFVQLGLLMALPMIMEIGLERGFRTAISDFIIMQL
QLASVFFTFSLGTKVHYFGRTILHGGAKYRATGRGFVVRHERFAENYRLYSRSHFTKALEIMVLLIIYMVYGSSGKGA
VAYILITASMWFLVITWLFAPFIFNPSGFEWQKIVEDWDDWKRWIANSGGIGVAAVKSWQSWWDEEHSYLEHSGIRG
RMMEILLSLRFFLYQYGLIYHLNITSGHVNIWIYGLSWVVLFGILFVLKIVSVGRRRFSGDYQLMFRFIKAAVFMGFMVI
LLLLFFLVGFTVGDLFVILLAFLPTGWALLQISMALRPILEPTGFWGSVRALARSYEYFMGLFIFTPVAILAWFPFVSEF
QTRLLFNQAFSRGLQISRILAGRQKRPRDRE

>Selbr4-Selaginella bryopteris 1921
MAFQRALRNWEKIVNAALQREELRGTTGPPKESSGIGVTTDTVPPSLLRESNIEEILQAADEIEADNPNVSRILCEYA
YSLAQNLDPKSEGRGVLQFKTGLKSVIKQKLSRKEGERIDRSQDISVLWEYYKYYRQKHNVDELKEQGGRWRMAG
DIDAQPEDLKQRTEKSRRVFATLKVLKDVLDALTRDTPDAARISEEMRKLMESDAAKVEEFKPYNILPLEAPGVADPII
LFSEIKGSIFALEYSNLPRLPGEFPKPTHRPLDVFDLLHFIFGFQRSNVDNQREHVLLLLANNQAKLGTLHEKDTHLD
EAAVNRVLDATLANYIRWCAFLRKRSVVLRAQDRQRKVLLLALFYLIWGEAANLRFLPECLCYIFHHMADELYMILEG
QVAERSKTCIDDKESFEYAFLHNVVTPIYSILAAEAANNNGGKASHATWRNYDDFNEYFWSPKCFELEWPWRTDAA
FFLRPKPKKPSDNADMRYRKSRYGRKVLFVEHRTFLHMYHSFHRLWIFLVLMLQGLTIFAFHENFHLVTIKQLLSLGP
TYVVMKFVQCLLDVILLYGAYASTNRAVLSRIFIRFLFFGSAATLITYLYVKVLNEASRSLSDSTFLKLYALIIGVYAAFHF
FVSVIMRIPFCYRMVSGLGEGGIMPFIKWVHQERYYVGRGLYESPSDFFKYFTFWLLVGASKFCFDYFLLIRPLVKPT
KTIVGIRNIVYDWHDFVSKGNHNALTLVAIWAAVVLIYFVDTQIFYTIWSALVGGLAGARDRLGEIRSITMLRRRFESYP
QAFVDTLLSSKAKETIPTIPPSGNLGNNYDSVSKVNAAKFAPFWNELIYSLREEDYLNNKERDLLLMPANNNILRLVQ
WPLFLLSSKVYVAAGLAEEHRGNQEELREKIEREDYMDSAVEETYRSLDLVLKDLLQDEAKSWARLVFEDIDRIKDD
GHFVAHFNLRKLRDILSIVNDLTTVLMRDESAENYKTAVKALQDLYEVVMRDFLNIELREKYEAWGTLQQARSEQRLF
ARLSWPTAPEQREQVKRLHSLLSKESAVNVPRNLEARRRLQFFTNSLFMKMPAAVPIQKMLSFSVFTPYYSEDVIYS
KDQLRQENEDGISILFYLQKIFPDEWQNFLQRIGITEAELERQLNQKALDLIELRLWASYRGQTLARTVRGMMYYRRA
LMLQSFLEQSDIGDVEDGLFARNPHDYQLSRRARAQADMKFTYVVTCQIYGQQKQSLKKDEKQRATDINYLMQKN
EALRIAYIDVVETVKEGKPYKEFYSKLIKADASGKDQEIYSIKLPGDPKLGEGKPENQNHAIVFTRGDAVQTIDMNQD
NYFEEALKMRNLLQEFDSKHGMRPPSILGVREHVFTGSVSSLAWFMSNQETSFVTLGQRVLAKPLKVRMHYGHPD

VFDRVFHITRGGISKASRVINISEDIYAGFNSTLRRGNITHHEYIQVGKGRDVGLNQIALFEAKVSSGNGEQMLSRDV
YRLGQLFDFFRMSSFFYTSVGYYICTMFTLWTVYAFLYGRLYLSLSGVEESLLNTADVLDNAALKSALNAQFLFQIGV
FSAVPMLVGLILEQGVLKAVISFITMQLQLCSVFFTFSLGTRTHYFGRTILHGGAKYRATGRGFVVRHIPFAENYRLYS
RSHFVKGLEIVMLLIVYMAYGAYTGASYILLSFSSWFLAISFLYAPYLFNPSGFEWQKTVDDFDDWTNWLFYKGGVG
VKGEQSWEAWWDEEQEHIRTIRSRVLETILSLRFFIFQYGVVYKLHATGKSTSLTAYAVSWAVFGAIILIFKIFSLSQKT
ATNIQLFLRLMQGIIFMLLLGGIIAAIIASPLTIGDVFATALALIPTGWAILSIAIAWRPIFRFLGLWKSMKSLARLYDALMG
MIIFIPIAILSWFPFVSTFQSRLLFNQAFSRGLEISLILAGNRPNVST

>Selbr5-Selaginella bryopteris 1742
MNPSGEVYNIIPVHNVLEGHLALNYPEVRAALAAFQTVGDLRKPPEFRWRQGMDLLDWLQGFFGFQEGNVKNQRE
HLVLLLSNSQMRLTPPPDPIDRLDQKVLRKVRRKVLRNYTLWCSYMGRTSNVQEGMDERMELMYVSLYLLIWGEAA
NLRFMPECLAYIFHHMADELNRIVNGHIDASSARLVVPETHGTDGFLKTVVTPLYNIVKEEAEASKNGTAPHSTWRNY
DDMNEYFWSNRCFKQLGWPLHRESNYFVKPQTGFMKHKVGKTGFVEQRSFWNLFRSFDRLWVMHILYFQAACIV
AFERKAPWASLRSKDVSLHLFSIFITWAGLRILQAVLDIWMQRKLISRETMLSGVRMVLKIIAGVTWVIVFAVLYSTMW
RIRREDGFWSNKANRRFRMLVEAAIVFIAPEILALLFFIVPWARNFIENKDWKIFKILTWWFQTRLFVGRGLRESLFDNI
KYTLFWVVVLAAKFAFSYAFQIAPLVNPTRAILRAKNIEYRWHEFFGRGNRIATACLWVPVILIYLMDTQIWYAVFSSAV
GALIGLFSHIGEIRSIHQFRLRFPFFASAIQFYLMPEESLLVPRRWGSWRVAFKDVMQRLRLRYGFGTPYKKIESKQIE
ERRFAHVWNQIINIFREEDLISNRELELLEVPTAKWNIPVLQWPAFLLSNEILIALGQATEWQDTDKRLWRKICKNEFR
PAAVIECYESLRHILLEKIIVERSDEHSILTELFQEIDNTILQGKFCTTFNLNELPGIHLRVLAFVEALAKKPTQDEAQKVV
VALQNLYDIVVRDFPKHKGLQGYLRPQRNEQELLFVDAVKLPDPEDNDFFPQLRRVQTILSTREAMTRVPKNLEARR
RISFFSNSLFMTMPRAPQVDKMLSFSVLTPYYSESVLYNKEDLVKENEEGVSILFYLQKIFPDEWTNLLERMLANGIK
EADLWTTDKGLELRLWASYRGQTLARTVRGMMYYNRALQMLAFLDSASEHELKGYREMLSRASSMQEGDIDITHIQ
GDSFGSGELYKQEQYRATANMKFTYIVTCQIYGAQKAKGLQIAEDIQFLMKKYEPLRIAYVDEVGGRESKLFYSVLVK
YDPVTMTEVEIYRIQLPGPLKLGEGKPENQNHAIIFTRGDAVQTIDMNQDNYFEEALKMRNLLQEFTRYYGMRKPTIL
GVREHVFTGSVSSLAHFMSSQEMSFVTLGQRVLANPLKVRMHYGHPDVFDRLWFLPRGGISKASRVINISEDIFAGF
NCTLRGGNVTHHEYIQVGKGRDVGLNQIALFEAKVASGNGEQTLSRDVYRLGHRLDFFRMLSFYYTTVGFYVSNLL
VVLTVYAFLWGRVYLALSGVERGLLSDANSNAALTAALNQQFIVQMGIFTALPMIVENAIERGFLTAIWDFFTMQLQLA
SMFFTFSMGTKAHYFGRTLLHGGAKYRATGRGFVVRHERFAEIYRLYSRSHFVKALELILLLVVYHAYGSFSTDTVVY
ILLTFSSWFLALTWIMAPFVFNPSGFDWLKTVDDYEDFVNWIFYRGGAIVKGEQSWESWWDEEQDHLRTTGFWGK

LFEVVLALRFFFFQYGIVYQLGIANHSTSILVYLLSWIYVLVAFLLHKILSFAHDKYATKEHLTYRAIQALALFFTILVIIILVE

LTSFRFSDLIVSLIAFLPTGWGMLQIAQVLRRPYLEKTGMWPTVVAVARLYELGMGFIVLLPTAILSWLPGFQAMQTRI

LFNQAFSRGLQISRILVGKKPRSY

>Cerri1-Ceratopteris richardii 1972

MSAPLGRSPSMDSSRGFDSGPSRPLTRTFTTGNLQDIMDPDVVPSTLSVIAPILRVANEIEDKAPRVAYLCRFFAFDK

AHRLDPYSEGRKVRQFKTALLHKLEKDDPYTVKLRTASNDAREVEKLYQEVYKTYVDLVANSGNENIDRAQLARTYQ

TASVLFDVLGTVSQNDIDKDVMSKAEDFQSKGELYGAYNILPLDAGGIKQAIMLLPEVMSALAAIRNTRGLEFPRAVQ

KTPKSDVLEWLQIMFGFQKDNVANQREHLILLLANVHIRQSSKAVANSQDSKLSDKALEDIMDRLFKNYKNWCSFLG

RGHNLELPTIQQEIQQRKLLYISLFLLIWGEAANLRFMPESLCYIFHHMASELHGMVDGNVSSVTGENVKPAYGGDE

NSFLQKVVTPIYEVIAAEVSCNNKGTARHSAWRNYDDLNEYFWSVDCFRLGWPMRMDADFFRVSEGSLMSRQGK

PAAGGKRPWIGKTNFVEERSFWHVFRSFDRMWSFFILALQAMVIIAWNGKLKDTFDQQIFKRVLSVFITSAILRLIQAI

VDIILSWKAWRCMRFTTMVRYVLKIVVAAGWVIILPVSYARTYSDIAFVSNFKNLLGENGGGSSLFVTALLVYLAPDVL

AFIMFLLPPLRKHIERSNSSFIRLLLWWAQPPLFIGRGMHEDWYTLFKYTSFWVLLLACKLLFSYYVEIVPLVGPTKEIL

QAPTGNYQWHEFFPHAKNNIGVLIALWSPIVLVYFMDTQIWYSIMSTVFGGVNGVWSRLGEIRTVAMLRSRFLSLPG

AFGANLVPPSSEERNGQGGSSHKNIQDGLDKGFKPSGKVDGEVEGLEMAGLDDKGDSDSQLKNAKFAQLWNEIIE

SFREEDYIDNKERELLLIPYNLDPSLKYMQWPPFLLANKIAQALDIAKDFTENNEDEDLWKKIKTDIYMKGAIEECYEL

FTNILSGIINDKCERECKLVKDIINDIGRKILGKNFLKAFRMAALPLLYIKIVELVAILKTPETEKAERVTYLLQDILEVYTR

DVQKDNNLDIPEASHHDKDQKSGDGQSENVSVEAKSSELFARKTIIYPPPASLAGKIQRLNLLLTITESAMDIPTNLEA

RRRISFFINSLFMDMPSPPKIRNMLSFSVLTPYYNEDVLFTRKGLEEENEDGVSILYYLQKIYPDEWRSFLERQNCTE

MNIWETDITADAVRQWASYRSQTLTRTVRGMMYYRRALKFQAFFDMAKDEDILQGYKARREDASVDNNQRSLAATI

DAVADMKFTYVVSCQQYGHHKHTKDQHAKDIENLMKEYPSLRVAYIDERDNEKTKEYYSVLVKCLVSKDDESIVSEQ

EIYRIKLPGRPILGEGKPENQNHAIIFTRGEVLQAIDMNQENYLEEAFKMRNLLQQFIEGPERPTIVGIRENIFTGSVSS

LGGFMSNQERSFVTIGQRVLANPLKVRFHYGHPDLFERLFHITSGGISKASKGVNLSEDIFAGFNTTLRGGNVTHHE

YIQVGKGRDVGLNQISMFEAKIANGNGEQTISRDLYRLGHRMDFFRMLSVYYTTVGFYFSTLIVIITVYVFLYGRIYLAL

SGLEASLEHFASLSNNSPLQAALASQSFVQIGLVTVLPMFVEIALEEGLKRASTEFIVMQLQLASVFFTFSMGTKIHFY

GRTLLHGGAKYRPTGRGFVVFHAKFAENYRLYSRSHFVKGLELLILLIVYNVYGSSVHKTIPYLLITFSQWFLVGTWLF

APFLFNPSGFEWQKIVDDWDDWTKWINNRGGIGIPAEKSWESWWEDEQAHLRSSGLGGQVIEILLSARFLLYQYGL

VYKLNVSHRSKSILVYGVSWVILLFVLGLIKVVSMGRQQLSAGYQLFFRLFKGLLLVGVLVVLPVLFIFANLSIGDLFVS

ALAFLPTGWALIQISQACRSLVKKIGMWESVKSLARGYETLMGSMLIAPVAILAWFPFISEFQTRLLFNEAFSRGLHIQ
RLFAGRTEKKMN

>Cerri2-Ceratopteris richardii 1947
MESSREGIEGAGGPPKRLGRTWTIAASSDAMDPEVVPSSLAVIAPILRVANEVEESDPRVAYLCRFYAFDKAHKLDP
TSEGRGVRQFKTALLHKLEKDDVTTRDKRKATSDAREIQDYYQNYYNKYVKSLENNIGVDRGQLVRTYQTAAVLFDV
LKAVNQTDLDPQIEQAGKEVEAKKEIYVEYNILPLDPTSASEPIMMFPEVKAAVLALRNTRGLPELPDTRAKKQRGID
MDLFDWLQGMFGFQKDNVANQREHLILLLANIHVRQMPRPEPLSKMSDTALDIVMKKIFKNYKRWCKFLNRRNNLW
FPTIQQEIEQAKILYIGLYLLIWGEAANLRFMPECICYIFHHMAGQLSNVYKGTAENEKAIHAEAEYSFLENVVKPIYDVI
AAEVNSSKSRHSEWRNYDDLNEYFWSPDCFRLNWPMRETEDFFRRPMGQKTADQKHKTFEQGTTGNKRKFWVD
KKNFVEVRSFLHIFRSFDRMWSFFIMSLQALIIVAWNGSGSLSNAFSGAVFEKVLSIFVTGALLRLLQAIVDIGLTIKAW
RSMKNTDILRYILKVLVATVWVIVLPVCYAHSWRDSSGLVSTFKDWLGQSRSPSLYVTAVLIYLAPDALSALLFLFPMV
RRAVEQSNSGIVHFLLWWDQPPIYVGRGMHESPIVLLKYTLFWILLLTCKFLFSYYVEIKPLVQPTKDLMNAKKPGWY
KMFPTGDDHTGIIFAIWVPIILVYFMDTQIWYAIWLTIFGGINGALSRIGEVRTVEMLESRFRSLSGAFNANLVNSRNDE
SSHRHQRGFSLQKFYRPLDELYKQYDQAGTGSLDEESNSRQTHRDGLAPHASFEVAKFSHLWNTIIKTLRDEDYIKN
EEMELLLIPYTSDVEMNYLPWPAFLLINKIASVVDVMKELTDQTNDEELYKKIFYKDHFKFHAVKECYVLLRRVLEMIV
KSQQDCRVIGKLLEDVKDIVTQRKILKSFRMSALPLLVSKFLQLISILEKPDMTKAEQVTFLLQDILEVYENDLRRETED
ESRHIDASQSSSHHDEKIRMREQLFQIKSIEYPPRSKILKQAIKRLHLLLTTDQSSVDVPANLEARRRITFFANSLFMD
MPPPPEISRMMSFSVLTPYLGEEIVCNKSALQLDNEDGISSLYYLQQIYPDEWKHFLQRINCESKEIWDTEKTALEAC
KWASFRAQTLSRTVRGMMYYRKALAHQAFFEMAHPEDVKKGFIDDIGEGPDGRNLSNLLANIEAIVDMKFTYVVTC
QDYALHKRKGDARASDILQLMEEYPAMRVAYIDEIEKDRRKEYFSILMKAAKNGDSERVIYKIKLPGKPILGEGKAENQ
NHAIIFTRGEALQAIDMNQDYYLEEALKMRNLLQEFVVKESVGSPSILGLREHIFTGSVSSLAGFMSNQESSFVTIVQ
RVLANPLKVRFHYGHPDVFDRLFHLTRGGISKASRTINLSEDIFAGFNTILRRGNITHHEYIQVGKGRDMSLNQISLFE
AKIANGNAEQTMSRDVYRLGHRVDFFRMLSVYFTTIGFYFNSLMVVLIVYVFLYGRLYLVLSGLESRLSDIGLQENIPL
QMAMASQTIVQIGFLIALPMIMEIGLERGFTTALSEFIVMQLQLASVFFTFSMGTLVHFYGRTLLHGGAKYRPTGRTFV
VFHAKFTENYRLYSRSHFTKGLELLILLIVYDAYGDPNHKGAAYYLIMFLLWFLVGTWLYAPFLFNPSGFEWQKIVED
WDDWNKWVNNRGGIGVPEEKSWESWWQDEQSHLENSGVQGKIIEVLLSTRFLLYQYGLVYHLRLVQKSSNVAAYL
ISWIVIVLVLVVVKAFAWTKQQFSAQNQLLFRVGRGLIIILIVASIIALIVPGYFVDIFVGALAFLPSGWAVVQMAQVCRPL
VQKVGMWDSVKALARFYEYLIGLVLFVPVATLAWFPFISDFQARILFNQAFSRGLQISRLLAGKKEKKMS

>Cerri3-Ceratopteris richardii 1905

MENTGRSFPQQRRMGRSLTLTGQFSADFRDSESVPSSLSQIGNILRVANEIEERNPRVAYLCRFHAFEKAHRLDPHS

KGRNVRQFKTALIQRLEKDDAETAAARTESTDAKEMRSFYQSYYEDYVKALSRAEQKDRAQLAQAYLTAGVLFEVL

CAVNNEEGVGPEIEAASKDVDEKKEMYCIYNILPLNEGGVSSPIMNAEEIRAAVSALRYLGSLSWPPNKRASKPKHA

DILDWLQVTFGFQKDSVANQREHLILLLANIHLRLDPKPPVKSKLDDRAVDELMKSLFKNYFKWCKFIQCKPAIWNPS

VKANYSEEEIQQRKLLYMGLYLLVWGEAANLRFMPECLCYIFHNMALELEGVLAREPTSVVTGENITPAYGGEEHAF

LRKIVTPIYEVIQKETVNNKNGTAPHSVWRNYDDLNEYFWSKHCFKLNWPMREDAEFFHVRSHGQTSTKRKSKTAL

PIVRKINFVEVRSFWHLFRSFDRMWTFYILALQAMIILSWNADGNPLHVFELSVFENVLSIFITAAILRLLQVVLDVLLN

YKAYNSLNKLRLFFKLAVAIAWVIVLPICYAHSWETQNGIVKVIKDWLNQTGKIPSLYITAVIIYLLPNVLSALLFLLPFLRR

WIENSNCWIFNALLWWSQPRLYIGRGMHEGPFALFKYTIFWIVLLIGKFLFSYFIQIRPLVKVTRGIMDVNKVNYEWHE

FFPHAKYNIGAIISVWAPVILVYFMDTQIWYAIFSTVVGGVTGAFRRLGEIRTLAMLRSRFRNLPDAFNRVLVPEKICDR

KVKHAKFAQLWNAVMHSLREEDIISDRDLLLLLVPSYSLSDSTLKISSVQWPLFLLASKLPIALNMAVEFEGSKDQELW

KRISADEYRKIAVEECYELFLIILDYVITGKIERRIFTHIKKYVETKIQEKTFLTTFSIKKLPLLKSKFEDLIERLEEEVEDDT

RHGSVVLLLQDMYEVVTEDLIVHGQVKIDNIPRDYSLFAKEGAVRSSLPNTQGWKEQIIRLRNLLKLKESAMDVPANL

EARRRIAFFTNSLFMGMPRAPPVSDMLSFSVLTPYYAEDTIYSKDHINVENEDGVSILFYLQKIYPDEWKNFLERLEC

KEESEEWKHEGFWVDERELALRHWASTRGQTLFRTVKGMMYYWQALKMQAYLDIASDEEILEGSQAYVFKDHDD

KSQRSLNATSEAIVDMKFTYVVSCQNYGAQKRSGDQHAADILSLMVRYPCLRIAYIDEREVREKDKLQKEYYSVLVK

AVNGLDEEVYRIKLPGPVKLGEGKPENQNHAIIFTRGEALQAIDMNQDNYLEEAFKMRNLLQEFKEDHGVHPSTILG

VREHIFTGSVSSLAWFMSMQEKSFVTIGQRVLAEPLKTRFHYGHPDVFDRLFHITRGGISKASRTINLSEDIFAGFNS

TLRQGNITHHEYIQVGKGRDVGLNQISMFEAKVANGNGEQALSRDMYRLGHRFDFFRMLTCYFTTVGFYLSSMIVIL

TVYVFLYGRLYLALSGLEASLVAEAELKRNIPLEAALASQSFVQLGLLMALPMVMEIGLEGGFWRAVSDFIVMQLQLS

SVFFTFSLGTKTHYFGRTILHGGAKYRATGRGFVVRHEKFAEIYRLYSRSHFTKGLELMVLLVIYKLYGQSSNGLVSYI

LVTFSMWFLVASWLFGPFLFNPSGFDWQKIVEDWEDWNQWFKSRGGIGVPSVKSWESWWNDEQEHLKYTGLSG

RFWEVVLSLRFFLYQYGLIYHLRIVKGHNQIEIYGFSWVVIAALLLLLKLVSMGRQRFGADFQLLFRLLKAGLFLVLLAI

LAAVLIVLNFSFNDLITCLLAFLPTGWALLQIAQALKPLVVVSGFWDSVKALAQGYEYIMGLLLFAPVAVLAWFPFVSEF

QSRLLFNQAFSRGLQISRILAGGRKG

>Cerri4-Ceratopteris richardii 1751

MAVRRLGTYEVGSSSNNPRTSAYNILPLNADAEICKLPEVKAARNELTRFPVKEGDDVFDLLKEKFLFQEDNIKNQRE

HLILLLSNARSADYDKDPASVKGKDQDLPVATVRSLFEKIMDNYWKWCSHVRIRSPDCRREEEDKQCMLKHVALYL

CIWGEAANVRFLPECICFLFHNLAKLLEHQGPLYPEGQTFLTAIVTPIYEKIKEEADKGKTTNQKHSKWKNYDDFNEL

FWSNLLFKLPQDNPIQPSNQLHLPEDDPIQHFQHNPGQPSKIKYFKDHFEDAYKNTESKKISYVERRTFFHLYHSFV

RVWILLIVLLEILVACAFSGNSLVLLLKLLICIGPTYFVMMFLKSVLDVVMMIGISVQVLSRCHVKASIIFQLLFYGACSFG

TIFLYRKMILEMPPNEILLWMLVPLGAYVVIFILRHAVRRQVERMERYKFLACLNWLYRDYFYVGGRLSEKGFYYWRY

ALFWLLLLGCKASFSYFFQIKPLAEASKSIYPHNEIVIYQWHDLISKRNHNALALMSLWAPVILIYLLDIQIWFTVFSAVV

GGILGSKDRLAEIHSLDMLRARFDTFPAELAVRLLASKKQTSAPKGIIISQAKTFSKFWNQIIKCLRNEDYIKDYEEELLI

MPEGFNNYITWPLFLLAGKVSLAIKLATENPEELFERLKNDKFMSLAIREAYASASYYYDTLIDGHKDYEIKKLGIERKL

SEIDKSSPDKEYKHIARIYRKVKKLVWLTDMDNQKKVNIRAVADALQSIYVDVSKFVKNEKYVQRADVRSTTMPNEIQ

VEDINLPANENKRTRRQKPLEPKFIRRLHWLLASTEAHSSVPKNEEAQRRLQFFTNSLFMRMPQAPSIRAMKAFSVL

TPYYREPVIYSVKDLYLDNEDGISILFYLQKVFPDEWENLLERLKITEKMLTDILIKASNISTGKDAFSKSDAEKVRETR

LWASYRGQTLARTVRGMMYYREALRFQACMEEREIEKNEINPEARAEALTDLKFSYVVSCQRYGEQKKHTEREER

EKAEDIQELMRTHDGLRIAFVHSEGKCYYSKLIKMSDTGEQEIYKIQLPGNPILGEGKPENQNHSIIFTRGEALQAIDM

NQDNYFEEALKMRNLLEQFKPPDKGPQATVLGVREHIFTGSVSSLAWFMSKQEASFVTLGQRVLARPLRVRMHYG

HPDVFDRLFHLTRGGMSKASKAINVSEDVFAGFNSTMRNGNITHHEYIQVGKGRDVGLNQISMFEAKVSSGNAEQL

LSRDVFRLGNSFDIFRLLSFYVTSVGYYACTMMTSLTIYVFLYGKAYLALSGLWRIFDDLALRNHDKPLQTALGTQFLF

QIGFFTAIPMLVMFMIEEGFIKAVVNIVVMQLQLSSVFFTFSLGTRSHYFSRTVLQGGAKYQDTGRNFVVQHIKFAQN

YRLYSRSHFVNAFEIMMLLIVIRVYGPQKSPQSYILFSFFCWFLAISWLFAPFIFNPSGFEWQKTVEDYNNWVKWLLG

RDNFDPNAKDCWENWWMEQQASNASIMSILLRTALSLRFLFPQFGIIYHLEKVKNDTSILIYGISWLIFIGIIMLFLTFSY

RSNYPGSMRPIRGVLYMILFVSFVMVEVLGIISINDIFATIFALIPTGWGLLCIAMIWSEYIQQSPIKPIWSIVKATAWYYD

AAMGMLIFIPIAVLSWFQFVSIFQTRLLFNQAFSRGLEISRILSARKQQDDEGSTAKIK

>Cerri5-Ceratopteris richardii 1683

MESKEVKAYNDWMLVLQHIWQHSKQKEKTSPFRPRKVKVNIRREPLNNILPPKDKRRVMKIEVERAMKMLELQSNK

ANNLPSENPPNIFDMLKFVFQFQNDNVENQKEHLILLLANSLSKLPPNYKENEKEDQIKKEVSNKILKNYENWCSYIG

KQRVYKDDEKLYYTALYLCIWGEAANVRLLPECICYLFHTLSEQLEKEIKKPALSDIGAESCQNSGFLDKYIVPIYEALE

KQAVRHGKAHSKWGNYDDFNEFFWSKSCFKLLENGHHENLKLSLLFSNAKRKVSFIEHRTFLHLYHSFARLWILLTL

MLQALTIIAFTMNFNRNTLKYLLSLGPTYFAMKFIESLCDIRMMMGFYVLLQHHDAARIVTLLCFYGGCTGGIAYLYWY

RYFSLWKTCLETTTTNFYLMLWVLGAYAVIKVILSLFQRLSSTNTNRLAVFMKWIHRGNLYVGDGLYESFQDYIKYSLF
WILLLAFKFSFSFYFQILPLAKASKAIYSFAEKDAYQWQDIISERHHNALALLFLWAPVVLIYLLDIEIWYIVLSSIIGGFSG
YCDGLGKILSLDMLRCHFSLFHKKFVQKLIYAKQSDSAKKQTYETCKFPEFWNKIIEHMREEDYLTNREKEFLTMPAV
GSSAEWPLFLLAGKVSLLLKDGNAAWVNIKRDPFMFSAVREVCYSILTLAECIEPNHMLFKGISEEVEIKLNDPKTFSF
EDLKSILQKVKGILRDLDNTFSANQFGHNLQDLIKLMCGDTHKKSIPLFEVEREDLLTVLCENDFFRRLKHLLNSKESD
AFIPRNREAQRRLQFFANSVFMKMPQTPSVEDMKSFSVLTPYDREAVIYSLENLQQENEDGITILFYLQKIFPDEWDN
LLERLDTTEKEITALLKEDLENGNATEEDSNKKALNVRLWASYRGQTLARTVRGMMYYRMALELQAFTEATDVNEK
QEIAVHKKQEIAEQLADFKFTYVLSCQNYWEQKESPYMEEQAKARDIQYLLKTYSHKGLRVACIEENIQGSKKKLYYS
NLIKMSEGEEKVVYSIKLPGNPMIGEGKPENQNHAIIFTRGDALQTIDMNQDNYFEEALKMRNLLEEFNGNHGLNDV
TILGVREHIFTGSVSSLASFMSNQEASFVTLGKRFLARPLKVRMHYGHPDIFDRLFHLTRGGVSKASKTININEDVFA
GFNSTLRKGNITHHEYIQVGKGRDVGLNQISLSEAKISGGNGEQLLSRDVYRLGQWFDFFRMLSFYVTSLGYYACTL
MTVVVVYIFLYGKAYLALSGLGKALSSHADIDDSRALQTALDAQFLIQIGILTAVPILAFFILEQGLVQAIIHFIVMQLQLCS
VFFTFSLGTRAHYFGRTVLQGGAKYMHTGRDFMVKHINFTENYRLYSRSHFARAFEIIMLLLVIKAYGYHNSTTSYSL
FSLFVWFLAISWLFAPFIFNPSGFEWQKTVEDIDDCRKWLFYERDIGIGEKNSWKKWWDKEQEHDTSIAESIVKTLLS
LRFFLVQYGLVYHLHIIQTPKLEHAYPLVYAYSWLVFLGVILIFLAFSYGPKISIHYQMLLRLIQGTLFIVLTIILGLCLAFKIF
SLSDLCVSALAFIPTGWGLLVIAKAWKPLMKKLHLWKGVRSIAWYYDMAMCMLILLPITILSWFPFVSTFQTRLLFNQA
FSRGLEISLILSGNRPDKSEKALAYKDN

>Cerri6-Ceratopteris richardii 1862
MSDRSRRKKEERILDNWRRVVSSVIAQPSRKSDFPDSVPASLGQASNINTILQVANEVQHEAPNVSRILCEYAYSLS
QSLDPNSGGRGVLQFKTGLMSVIKQKIAKKDGIFDRRQDIATVGKFYNDFREKHHLEEVQADANGHSQKSQDVRLR
TRTYTVLRVLKDCVYYLAQEYSIDPDTILSPKLKKAMESDAKNSEDFQPYNILPLEAPGVSEVALEFPEVKSARMALH
VPALHFSDESRAPPRQKRDIFDALEYAFGFQHDNVRNQREHLVLLLANHQSQLDYEPSEQVVHGDAVQTLCEKILG
NYENWCYYIRKEPVHSRRDSSEGKVAFMALYLLIWGEAANVRFLPEALCYIFHNMAHEFTSMLESKLIEQSVSCKSE
NGQSFLDQIITPFYVALKMEAAKSNGGKSGHSKWRNYDDFNEFFWSYSCFKELGWPLNHKSPFFGEVKGSVRHRV
GKVSFVEHRTFFHLYHSFVRTWLLLVLMFQALTIIAFTEGYRLQRLKILLSTGLTYFVMKLIESVLDVILMFGAYSSSRIN
AVNGILAHFVFYGACCGGFIFLYLKMMLEGNGSNTFFRIYILALGVYVAVQLVIRFFLHIPSFRKQAGKAANNRCIEVFK
WLYQENHFVGRGLVESPKDFLRYSLFWVVVLGCKFTFSYFLQIKPLVPATQAITSFQEEITYTWHDFVSKNNHNVMA
VFFLWAPVVVIYLLDISVWYTVLSAIWGGLLGARDRLGEIRSPEMLRGRFESFPVGFVNNLVYDDSNIGRSETGEVSK

GDSARIFAPFWNEIISCLREEDYISNRERDLLRMPSNAVGLSLVQWPLFLLVSKVFLAMDLAMEKKESSEEIWEKLSK

DQYMASAVREVFESVCLLLSDLLEGEGKKWFQGVFTDIERSIKDKSFFALFQINNIQDVVKKITALTGLLVKGNSMDG

VPSAVQGLYESVYNFLSDQSRNEHRKFSAEELKVIFSKVRLPKDDKKESIKRLHSLLTIKESAATVPKNLEARRRLEFF

TNSLFMKMPDCPPVAAMKSFSVYTPYYEEIVMYSGKELCEDNEDGISTLFYLQKIFPDEWKNFLEKIGLIEATFDTQF

KSGALADENLLKLRLWASYRGQTLARTVRGMMYYRRAIVLQSLLEARGKGRWSEQECNERAKAYADLKFTYVITCQ

KFGQQKVEEKGKAKAQDILYLMKENEALRIAYIDQVESDGVKGGKEFYSKLVKNDGGALKDQLIFSIKLPGNPILGEG

KPENQNHAVIFTRGEALQTIDMNQDNYFEEALKMRNLLQEFEGNYGLRSPTILGVREHVFTGSVSSLAWFMSNQET

SFVTLGQRVLARPLKVRMHYGHPDVFDRLFHLTRGGFSKASKVINISEDIYAGFNSTLRQGNVTHHEYIQVGKGRDV

GLNQIAMFEAKVSSGNGEQSLSRDVYRLGQLLDFFRMLSFYTTSVGYYVCTMLTVLVVYVFLYGKAYLALSGVGQSL

QDMALLTNNQALQSALNTQFLLQIGVFTAVPMVMGFILEQGVLKAIVSFITMQLQLASVFFTFSLGTRTHYFGRTILHG

GAKYRSTGRGFVVTHVKFAENYRLYSRSHFVKALEIAMLLIVYLAYGYETTTISYILLSFSSWFLAISWLFAPYIFNPSG

FEWQKTVDDFDDWMGWLLYRGGVGVKTDDSWEAWWEDEQGHIRHNRGRILETILSLRFFFFQYGVIYQLKVLNNN

TSLAVYGISWLILVGIILIFKVFAYRSKSSVVLRLIQGLLFIAIIGSLVASIAVTSLTLGDVFAALLALVPTGWGILCIGIAWKG

RIKALGLWSSFRAIARFYDAAMGMVIFLPVAFLSWFPFVSTFQTRLLFNQAFSRGLEISLILAGNRPNAQS

>Cerri7-Ceratopteris richardii 1917

MSTSSRHVQRPIRNWERLCKAVLQRQQLHGNSSLGRLESTPALGGSVPSSLGHQSANIDAILQVANEIQSENPNVS

RILCEHAYSLSQDLDPNSEGRGVLQFKTGLMSVIKQKLAKKDGEGIDRSQDVVHIEKFYKDYRKRHNIRDMQEEQQ

GSSPSGRRLNELERSLEMKKAYAVLGVLKGVVDSLPPDAITAEFRRILEADAAKTEQFKAYNILPLGAPGIADGNRDT

SEILAAMNSLDGQVVLDVYVKTLPNDFEYPHYRRVDVFDLLEYIFGFQRDNASNQREHFVLLLASAQSHLYSYYKNG

NQGPGEEVLHQLDENAINKVCEKVLDNYIKWCDYLQKPPFYRFHGDKQTKLLLASLYLLVWGEAANVRFLPECLCYI

FHRMADELLTILNSSRIKRADSCQVDHGSSFLNRVILPIYMAVEKEASANRGHGGKKVPHSKWRNYDDFNEYFWSS

ACFELQWPLDLDSPFFSRSKPVVQGGRRSPAEKKMGKSSFVEHRSFVHIYHSFHRLWIFLVLAFQGLAVIAFSSSLIS

MTTLKGLLSLAPTFLVMKFIESILDILLMVGAYRNSPGNAVARIFIRFFWFGALSVSLTFLYVLVLNENSRSFSESTLLTF

YTAIVVLYIVLELLLALFLRVPTFRDWTQKSSRWGVVRFLKWMHQEQYYVGRGLYEKPTHYLQYVAFWVLVMGCKF

PFTYFLQIKPLVTPTRTISGYNDLTYTWHDFISQGNHNALTIAAMWAPVIMIYLLDIQIWYTILSSVVGGLIGTNDRLGEI

RSLESMRSQFHEFPQEFGKKLIAGAQMNFEMVKYNAARFASYWNQIITALREEDYISNREKDLLVMPPNVKSEFAK

WPLFLLVSKVFLAAKLADEHHDNQRELSEKIKRDKYMLNAVEEVYQSFFTILSNMFEGVESQWVSSLRNKIENSIVED

RLLTEFRVKNIQSVLTKVAALLAVLGKEETPEQRKEAVKAMQDLYDGVTHDLMNSSQGQDLYHGALLQRMAEGRLF

SNLRWPAISDDRDQVRRLHMLLTFNESAASVPKNLEARRRLLFFSNSLFMNMPKAVPVREMRSFSVFTPYYKEDVL
YTNDQLQKQNKDGISTLFYLQKIFKDEWSNFLERVGIDEPTLFAKVKLKDPVALELRLWASYRGQTLARTVRGMMYY
RRALLLQGFLESQEGFHQSGADRDSHHIPSFSSSGYLNSSAARAQADLKFTYVVSCQVYGQQKQSEKEKHVAADIL
YLMQRNDALRVAYIDTVEVLRGQKTHKEYFSKLVKADLSGKDQEIYKIKLPGNPLLGEGKPENQNHAIIFTRGEAVQTI
DMNQDNYFEEALKMRNLLEEFVVKDHGLRLPTILGVREHVFTGSVSSLAWFMSNQEGSFVTLGQRVLARPLKVRM
HYGHPDVFDRVFHITRGGISKASQIINISEDIYAGFNSTLRQGNITHHEYIQVGKGRDVGLNQIAMFEGKVAAGNGEQ
ALSRDVYRLGQLFDFFRMLSFYVTSVGFYVCTMMTVLTVYAFLYGKAYLALSGVGAQLESRAQITSNAALQSALETQ
FLFQIGIFTAIPMIMGFILEQGPLKAVVNFVTMQLQLASVFFTFSLGTRTHYFGRTLLHGGAKYRATGRGFVVEHIKFA
EIYRLFARSHFVKGLEIVILLLIYLVYGFGDSTLGYILLSFSSWFLAISWLYAPFIFNPSGFEWQKTVEDFGDWTNWLLY
KGGVGVKGEESWETWWDEEQSHIQTLRGKILETLLSLRFFFFQYGVVYRLHAADSSTSLRVYGVSWLVLIAIVLLFKI
FTFSQKTSVNFQMFLRLFQGTVFVLLLVALALLIVLTALSFGDIFASLLALIPTGWAILSIAITWKPVVKRLGLWKSIRTIA
RFYDAAMGMVVFFPIALLSWFPFVSTFQNRLLFNQAFSRGLEISQILSGKPADA

>Cerri8-Ceratopteris richardii 1917

MSTSSRHEQRPIRNWERLGKALLRRQQLHGNSSVGRAESMPALGGFVPSSLGHQSANIDAILQVANEIQSENPNVS
RILCEHAYSLSQDLDPNSEGRGVLQFKTGLMSVIKQKLAKKDGEGIDRSQDIVHIEKFYKDYRKRHNIGDLQEEQQG
SSLSGRRLNELERSLEMKKAYAVLGVLKGVVDSLPSDVITADFRRILEADAAKTEQFKAYNILPLGAPGIADGNRDTP
EILAAVNSLDGQVVLDVYVKTLPNDFEYPHYRRVDVFDLLEYIFGFQRDNASNQREHFVLLLASAQSHLYSYYKNGN
QGPGEEVLHQLDENAINKVCEKVLDNYIKWCDYLQKPPFYHFNGDKQTKLLLTSLYLLIWGEAANVRFLPECLCYIFH
RMADELLSTLNSSMIRRAESCKADHGSSFLNRVILPIYTSLEKEASANRGHGAKKVPHSKWRNYDDFNEYFWSSTC
FELQWPLDLDSLFFSKRKPVAQVGKKSPAERKMGKSSFVEHRTFLHIYHSFHRLWIFLVLAFQGLAVIAFSGSLISITT
LKGLLSLGPTFLVMKFIESILDILLMVGAYQNSPGNAVARIFIRFFWFGALSGSLTFLYVLVLKENSTSFSDSTLLTFYTAI
IILYIVLELLLALFLRVPTFRDWTQKSSRWGVVRFLKWMHQEQYYVGRGLYEKPTHYLQYVAFWLLVMGCKFPFSYF
LQIKPLVAPTQRISGYNDLTYTWHDFVSQGNHNALTIVALWAPVVMIYLLDIQIWYTILSSIVGGLIGTNDRLGEIRSLES
MRSQFHEFPEEFGKKLIGGAQMNFEMVKYNAARFASYWNQIITALREEDYISNREKDLLVMPPNVKSEFAKWPLFLL
VSKVFLAAKLADEHHDNQRELSEKIKRDKYMLNAVEEVYQSFFTILSNMFEGVESQWVSSLRNKIENSIVEDRLLTEF
RVKNIQSVLTKVAALLAVLGKEETPEQRKEAVKAMQDLYDGVTHDLMNSSQGQDLYHGALLQRMAEGRLFSNLRW
PAISDDRDQVRRLHMLLTFNESAASVPKNLEARRRLLFFSNSLFMNMPKAVPVREMRSFSVFTPYYKEDVLYTNDQL
QKMNKDGISTLFYLQKIFKDEWTNFLERVGCDEPTLFEKVKRKDPEALELRLWASYRGQTLARTVRGMMYYRRALL

LQSFLEGQQGFHQAGGDRDSQHIPSFSSSGYLNSIAARAQADLKFTYVVSCQVYGQQKQNEKEKHVAADILYLMQ

RNEALRVAYIDTVEVLRDQKTHKEYYSKLVKADLSGKDQEIYNIKLPGNPLLGEGKPENQNHAIIFTRGEAVQTIDMN

QDNYFEEALKIRNLLEEFVVKDHGLRLPTILGVREHVFTGSVSSLAWFMSNQEGSFVTLGQRVLARPLKVRMHYGH

PDVFDRVFHITRGGISKASQIINISEDIYAGFNSTLRQGNITHHEYVQVGKGRDVGLNQIAMFEGKVAAGNGEQVLSR

DVYRLGQLFDFFRMLSFYVTSVGFYVCTMMTVLTVYAFLYGKAYLALSGIGAQLESRAQITSNAALQSALETQFLFQI

GIFTAIPMIMGFILEQGPLKAVVNFVTMQLQLASIFFTFSLGTRTHYFGRTLLHGGAKYRATGRGFVVEHIKFAEIYRLF

ARSHFVKGLEIVILLLIYMVYGYEDSTVGYILMSFSSWFLAISWLYAPFIFNPSGFEWQKTVEDFGDWTNWLLYKGGV

GVKGEESWETWWDEEQSHMQTLRGKFLETLLSLRFLFFQYGVVYRLHAADSSTSLRVYGVSWVVLIAIVLLFKIFTF

SQKTSVNFQMFLRLFQGTVFVLLLAALALLIVLTALSFGDIFASLLALIPTGWAILSIAITWKPVVKRLWLWKSIRTIARFY

DAAMGMVVFFPIAILSWFPFVSTFQNRLLFNQAFSRGLEISQILSGKPADA

>Cerri9-Ceratopteris richardii 1780

MRPSMGRTVSEDEEVYNILPIHDMLADNDALRFPEVRAAMKALEPMVDLPMPPTFVLQGREMKRWRRQRRDIMD

WLEIFFGFQKDNVKNQREHLVLLLANYQMRLQPPPDPLDTLDHKVVHHLRRRLLKNYTKWCSFLREPSSLFLSEVN

GDWRQLTYCSLYLLIWGEAANLRFMPECLCYIFHHMAKELNRILGSPQTDKDNLPYSFGQCGFLDKVVKPIYEAVRG

EAQAAMEGRAPHSSWRNYDDMNEYFWTKRCFRQLGWPLDMQSNYLMTPHQQKNQEQNGGSKHLQQKVGKTG

FVEQRSFLNIYRSFDHLWTGLILMFQAMMILAFNKNGIPWDVLYDRDVQGSVLTIFVTWPGLRLLQATLDVVTQYRLV

SSETKLVGLRMVLKILVASIWTTIFSYLLARAWKQRTIDRKWSPEANLKLIQLLETAAVFILPEILAIVLFILPWIRNFVERS

EWKIFHLITWWFQTRLFVARGLRENVFDNIRYTTFWLMVLCTKFSFSYFMQVRPLVEPTRALLRLDMINYEWHEFFT

NHNRFVVIVIWTPVILVYLMDIQIWYAVFQSLVGAMVGLTNHIGEIRTVQQLKLRFPFFASAITFNLAPKEGLLGEDILPH

SRYVNLVKDMVRRVKLRYGLGKDYRKFESNTLEARKFAYIWNTVIHFFRQEDLISDHESQLLQFPGSSWNVSVVQW

PSVLLSNEVLAAVSMTQVWQGNDDQRLWTKICKNEYRRCAVVECYDSIKYLLKRILNEGSEEYRIVDDLFNQIEREIE

EGTFCENFKVSALAEVHSRILGLVTVLSNKAPRKEVVVALQNLYDAVVRDFLQFHTKDSVSSVRGHASSTERIELLFV

NAVQVPHYEKEKAFYHRLRRFSTTLSPKGAMNDVPKNLDARRRIAFFSNSIFMNMPHAPTVERMLSFSVLTPYYKE

DVLYSIQQLNSPNEDGVTVLFYLQSIYPDEWRNFLERMKLQDVQHPEENLPEEKLESSEEFKMQLRLWASYRGQTL

GRTVRGMMYYHHALETLDYLDTASEFDLKQGQDYIRSESLNSCQSFDYSSTQKASSNLNSMSAVYRVSQEKATALL

KFTYVVACQMYGSHKSKNDPRANDILFLMRNNPAMRVAYIDEVCRPGSGKKFYSVLVKYDTVRRQEVEIYRIQLPGE

VKLGEGKPENQNQALIFTRGDAVQTIDMNQDNYFEEALKIRNLLQEFTMYYGLRRPQILGVREHVFTGSVSSVAWF

MSAQESSFVTLGQRVLAYPLRIRMHYGHPDVFDRIWFLTRGGISKASRVINISEDIFAGYNCALRGGTVTHHEYIQVG

KGRDLGLNQIALFEAKVSSGNGEQMLSRDVFRLGHRLDFFRMLSFYYTTVGFYISSLITILTIYAFLWGRVYLALSGFE
QFIVNSLHNSALMASLNQQFLVQLGFFSALPMIVESSLERGFSTALWEFLTMQLEMCSVFFTFSLGTKGHHFGKTLL
HGGAKYRATGRGFVVRHEKFTENYRLYARSHFVKGVELMILLIVYEAYGGLTKTTHVYLLLTFSSWFLAISWILAPFLF
NPFCLQTVNEFEDFQNWLWCRRGLLAKGNQSWEVWWNEEQDYLRTTGVWGKILEVILSCRFFLIQYGVVYRLHIA
AKNKSIFVYLFSWIFVIAAIFLCFLISRAGERFSAKNHLLYRTIQLLVALGMVLGLIVLLDLTEFHFVDLFLSFLAFIPSGW
GLLCISLVFKPSLVQTRMWPVVVTIARLYEFALGVTVMIPLAILSWVPGFQEMQTRMLFNQAFNRGLHISKILKRPNQP
V

>Cerri10-Ceratopteris richardii 1769
MAYKEDFEDVYKYNILQLNEGSIGSDDGLQFPAVRAATEALKRVKDLRRPPAEALLVRENTDIFDWLGAFFGFQADN
VRNQRENVVLLLANYQMSLQPPPEPMSRLDRSAVQRLRKKLLKNYTMWCSYFHKKPNLWLPKKAHNIDDHRELLY
SCLYLLIWGEAANLRFMPECLSFIFHNMAMELNSIIEFQMDAETGQYTPASYGRNGFLIRVVQPIYTTVKGEAEASNG
GKSPHSAWRNYDDMNEYFWTKRCFKQLSWPLKQSSSYLVAPNKNRRRNSRPIGKTGFVEQRSFWNIFRSFDHLWI
SLILMLQAMMILAFNGDGLPWHVLRDRDVIAALLTVFITWAGLRCMQAVLDAGMQYSLISSETKMIGIRIFLKILVAAG
WTTAFSVLLSRAWQQRMKDRHWSPEAESLLKEFLEAAALFILPETLAIVLFLVPWVRNFLEKSEFRVLHALTWWFQT
RLFVARGLREGVIDSLKYATFWLAILSVKFSFSYFLQVRPLVAPTRELLNLNIVEFQWHEFFRNHNRFVVVVLWAPVV
LIYAMDLQIWYSIASSLVGAAIGLFSHIGEIRNMEQLKLRFPFFASAVAFNLSPEVEKYMKQPSPHYRLVALFRDMVRR
VKLRYGLEKAYTTFEKSSPESLKFAKLWNEILFHLRQEDLVSDYEVELLQVPQFSWNIDVMQWPSVLLSNSVLVALGF
ARDWRSVDCQKLWEKISKNEYRKCAVLECYYSFKHLMSRVLKDDSAESSIVEHLFMEIENEIHLKTFCQSYKVNLIPE
VHAHIVSVVEAILKRPGLKDIQKVVDTLQNLYDVVVRDFPVQKRQYESLRGAGLVAESNSLLFVDAVKLPDPDDELFF
HVLKRLHITLSTKEALDDVPKNIDAKRRISFFSNSLFMNMPHAPSLERMRSFSVLTPYYSEDVIYTKDQLRIPNEDGVT
ILYYLQSVYPDEWNNFLERMGLKESDHPEEDLWNNKDRLKQLRLWASYRGQTLSRTVRGMMYYQHALDALAYYDA
DFEPDFQVSSHSRQLESLSYSFRIAGKPFFQTGAYSGGAKPQMKADRKVKRSKAIARLKFTYVVACQIYGTQKGKKD
AKADDIYFLMQTNPAVRVAYVDEVPTDHGVQYFSVLVKYDTYLDKEIEIYRIQLPGPLKLGEGKPENQNHAIIFTRGDA
IQTIDMNQDNYFEEALKMRNLLEEFDKHYGIRRPQILGVREHVFTGSVSSLAWFMSAQETSFVTLGQRVLARPLKVR
MHYGHPDVFDRLWFLTRGGISKASRVINISEDIFAGFNCTLRGGNVTHHEYIQVGKGRDVGLNQIALFEAKVSSGNG
EQMLSRDVYRLGHHLDFFRMLSFYHTTVGFYIANMLIVLTVYAFLWGRVYLALSGIEDVVKNSIVNTALSASLNQQFIF
QMGLFTALPMIIENTLELGFSGAVWDFITMQLELCSVFYAFSLGTKCHYFGRTLLHGGAKYRATGRGFVVRHESFAE
NYRLYARSHFVKGVELIVLLIIYEAYGGTASSTVVYILLTFSSWFLALSWLIAPFLFNPTGFDWLQTVYDLEDFQSWLW

YKGGVLTKAEQSWEIWWNEENDHLRTTDFWGRVLEIVLNIRFFLIHYGMVYRLHIADNNKSVLVYFVSWIYILCAMAL
YMILAYAGEKYAAKKHLYFRAIQAFVGLLVALVIVLLLELTDFQLVDLLLSFLAFLPTGWGFISICIVLKPFIVNTIAWPVIV
SMARLYELAIAGIVIVPLAILSWIPGFQAMQTRILFNQAFSRGLQLSRILTAKKPTRG

>Cerri11-Ceratopteris richardii 1773
MDGHISSRGRENEGSVDDEPYNILPLHDMLADHDALRFPEVRAAHAALQAVGDLSRPQGWRHGMDLLDWLGVLF
GFQAGNVKNQREHLVLLLANDQMRIHPPPDPVDRMDARVVRKLRKKVLKNYVNWCSYLRKKSNLWLKNQVNEQR
DLLYISLYLLIWGESANLRFMPECLCFIFHNMANELNRVLEHYIDDTGRPAQPAYTGSNAFLEKVVTPLYDIVRAEALA
CHEGKAPHSAWRNYDDMNEYFWSKRCFSQLSWPLNRSCSFMVDPSADRGKFFGQRRVGKTGFVEQRSFWNIYR
SFHHLWTGYILLLQAMVILAFNSDSTPWKQLTERSVQAKLLTIFITWAGLRILQAVLDLVMQFRIISVGNILTGLRMVLKI
VVASGWTVVFVVLYIRMWNQRNSVGSWSGRARTAFLQFLEAAAVFIIPEVLALTSFVLPWIRILVEQSEWKFFHILTW
WFQARWFVARGLREGVWDNFKYTSFWFLVLLTKIAFSYFLQIKPLVDPTRELLDLKNVDYAWHEFWSGSNRFSVLVL
WAPVVLIYFMDTQIWYTVFSSLIGALVGLFAHLGEIRNLPQLKLRFPFFASAVFFNLMPEDSYITARPWGNVSKSIKDV
WHRVKLRYGFGTIYRKLEPGSLEAGKFAYLWNSIMENFREEDIISDKELELLEIPAPAWNISVVQWPSVLLSNELQLAL
RQAQAWTGKDDKRLWRKVCRSEYRRCAVIECFESTKHVLMRIVKLKTREYEIIDNILSDINSSLASYRFLENYAVREL
PEVHARVLALVTILEKKPVDEDIPALVQALQNLFEVVVRDLQLEKEDSMSARVTTGLASELLFLDAIELPHHDDEAFFR
QLRRFKTTLSTREAMNNIPRNLEARRRIAFFSNSLFMKMPHAPPVEKMLAFSVLTPYYSEDVMYSREKLLAQNEDGI
SILYYLNKIYPDEWENFMERMRREEGIKDIKDLWSLEKALQLRLWASYRGQTLARTVRGMTYYHKALQMLSYLDGVS
EVDMRQGIELMTASVRGGRTNSIASAEINSNGSQRLSLGSSGRSNTGELFKKSQQQATASMKFTYVVTCQIYGAQK
AKKEVQAEDILYLLQKHEALRVAYVDEVATGNGKKYYSVLVKYDDIMEKEVEIYRVQLPGPLKLGEGKPENQNHAIIFT
RGDAVQTIDMNQDNYFEEALKARNLLQEFTRKYGIRRPQILGVREHIFTGSVSSLAWFMSAQETSFVTLGQRVLANP
LKIRMHYGHPDVFDRLWFITRGGISKASRVINISEDIFAGFNCTLRGGNVTHHEYIQVGKGRDVGLNQTALFEAKVAS
GNGEQILSRDVYRLGHHLDFFRMLSFYYTTVGFFISNMMVVLTVYAFLWGRAYLALSGLENAASGTLASGALTASINQ
QFIVQLGIFTALPMIVENTIEHGFASSVWDFLTMQLQLSSVFFTFSLGTRAHFFGRTVLHGGAKYRATGRGFVVVHEK
FAHIYRLFSRSHFVKAIELILLLIVYMSFSSVGKSTTSYILITFSSWFLALSWILAPFLFNPSGFDWLKTVEDFEDFLDWI
WYRGGISVKSDQSWEVWWNEEQEHLRSTGFWGKVLEIVLSLRFFVFQYGVVYHMHIAADSTSILVYLLSWICVAGS
LFIYIVLMLAANRYSMNNHIYFRAIQALVIFLMVLLIVVLVEFTSFQILDVLLSILAFVPTGWGFLSIALVFRHFLERLKLWP
LVISVARMYELGFGVIVFTPVAVLSWLPGFQSMQTRILFNEAFSRGLHISRILVGKRPNPGF

>Cerri12-Ceratopteris richardii 1807

MASNRRFNGHAHPPGIRAQTDEEETAEETFNIVPLHDMLMDEDIVRFPEVRAARAALQTLGGLSQPAGWEEGMDLL

DWLSLLFGFQEGNVKNQREHLVMLLANKQMRLQPPPDPLDRLDDTVVEDIRNKLLRNYVEWCSFLRKANNLWLQR

GSHDQQRKELLYIGLHLLIWGEAANLRFMPECLCFLFHNMAGEMNKILEHKLQGGDASQPFMPHYTGDNAFLLKVIT

PLYRLVRAEAEASHHGSKPHSSWRNYDDLNEFFWSDECFRQLSWPMNPSCSFMYCPPSGRKAQRRSRVGKTGF

VEQRSFWNLFRSFDHLWAGLILMLQAMIILAFHDHEKAPWTLLSDRDVQARALSIFISWAGIRLLQSVLDIIMQFALISA

ETILIGVRMVFKIVVASIWTLLFALLYRKAWDRRGVEGGWFNEADAAFRQLVIAAALFILPELFSIILLLLPWLGNPIERS

EHRVSRALTWWFYSPCFVGRGLREGLWDNIKSALYWGIVLVTKFSFSYFLQIRPLVVPTRELLALRGVDYSWYQFW

GSGSNRVAVFVLWAPVVLVYLMDLQIWYTILSAFVGYLIGLLQHIGEIRNLDQVKMRFQFFASAVSLNLTPEEPLFRVR

ELGSWLSKFIRRTRLRYGFSTDYKKYASGSYEAEKFAFLWNTIIKTFREEDIVSDKEEQLLEVPSPAWNISVTQWPCM

LLANELSIALQQIKNWQGDDDRRLWRKIAKSEYRRCAVIECFESSRHVMQRILREGSAELRRIEKIFSRIQSSLNAGSF

LKDYKVSSLIRVHEWILELVMMLLMVEKSKHDEGFHARVVEILQSLSEVVDRDLRSESQQTGEESTSRGNLSRMKEL

PFTAAIVLPDFRDTAFMRQLRRFYTILSTKDTMRDIPKNCEARRRIAFFCNSLFMNMPSAPPVERMLGFSVLTPYYEE

EVLYSKANLKAKTEDGSSVLYYLQTIFPDDWTNFIERMIREKGLTNVQDLWDLDGGIDLRLWASYRGQTLARTVRGM

MYYHKAMKVLSYLDSASPIELQEGRELLASMNLEGNTASPGSSSRDHILNRTRSGVSGVYKEAQQNAIANMKFTYV

VTCQLYGEHKAIDSKPEKKRLADDILFLMRNNKALRIAYVDRVKAGGADDFYSVLVKYDSLLEREVEIYRIKLPGPMKL

GEGKPENQNHALIFTRGDALQTIDMNQDNYFEEALKARNLLQEFTVRHGLRKPQILGVREHVFTGTVSSVASFMSG

QETSFVTMGQRVLANPLKVRMHYGHPDVFDRLWFLQRGGISKASSKINVSEDIYAGFNCTLRGGNVTHHEYIQAGK

GRDLGLSQIAVFESKVASGNGEQVLSRDVYRLGHRLDFFRMLSFYYTTVGFFISNLMVVLTVYAFLWGRVYLALSGL

ESSVLTQSAFANTALTASLNQQFLVQLGILTALPMILQNSLEQGFYSSLWDFIIMQLQLASVFFTFSLGTRAHYFGRTVL

HGGAGYRATGRGFVLHHEKFCDTYILFSRSHFVKGVELIVLLVIYQAFGAVGAVSTTAYVLITISTWFLALSWVLGPFL

FNPLGFDQLKLSQDIDDFWNWIWDKRECAEDRDWEKYRANPQRSWKVWWSKEYEHLNHTGLWGMIFEIVLNLRF

LFLQYGIVYHLNIAGGQKSIFVYIFSWLYMAVITGTYVIVLIAARKLSVKKHVYYRLVQATIAVVVVVVLIVLATRTSFQIID

TILSVFAFLPTGWALLSIVVVFRRFIEHTPVWEIVVSVARLYELFLGILVFIPLLVLSWIPGFQDMQNRILFSTAYISGINIT

RLFEEKKPRNQPESNKTV

>Cerri13-Ceratopteris richardii 1770

MGPQPAGSIVCGGSSKPRQCAELLGLDGRGRRTEDTFNILPLHNILVDDEIVRFPEVRAVLTALQNVEGLSEPPECA

HGKEFKDLLDWLALFFGFQTHNVKNQREHLVMLLANQQMRLQPPPDPLDHLDSTVVEELRKKLLNNYVEWCSFLR

KANNLWLQRGSHEQQQRELLYVGLYLLIWGEAANLRFMPECLCFLFHNMAGELNKILEPLRRTTPGRMHFTCRSSI
HCTNLSAQRLKPHTMDPALIPPGGIYDDLNEFFWSDQCFTQLSWPMNPSCFFMYCPPSSVNPDRRVGVGKTGFVE
QRSFWNLFRSFDHLWIALIIILQTMIILAFHNHEKAPWTLLSERDVQAQTMSIFISWGGMRLLHSLLDMIMQFALISAET
ILIGVRMLFKIFFASIWTMVFAILYTRAWHQRGVEGDWINEADAAFRRLVIAVVVFILPEMFSIILLVLPWIGNLIEGSEHR
VSRALTWWFYTPSYVGRALREGLWDNIKSALFWIIVLAAKFCFSYFLQIRPLVVPTRELLALRDVEYSWYEFWGSGS
NRVAVFVLWAPVVLVYFMDLQIWYTILSALVGYVIGLLQHIGEIRNFDQVKMRFQFFASAASLNLRPEESLFKSDENW
GSWLRKSIRRITLRHGFSADYKEYASGCNEVERFALLWNTIIKTLREEDIISDKEVRLLEVPPPAWNISVIQILREGSEE
SQLIERIFSHIQNSLNVGSFLKDYKVKSLRRVRDLIMELVKVLKNRKSKYDKRFNKVENIFRSLCEVVGLHLMRSDSQ
QIGEESTHRRMTELPFTKAIVLPDPHDAAFRRQLLRFYTILKTERTMHEIPRSREARRRIVFFCNSLFMNMPNAPPVD
RMLGFSVLTPYFEEEVLYSKDYLEQKTDDGISVLFYLRTIFRDDWSNFKRRMKREKGLEKVRIKNLWNIDGGIDLRLW
ASYRGQTLARTVRGMMYYHKALKMLAYLDSASSIELQEGRELLAAVNLERNHVVHRGLYNEAQQNAVANIKFTYVVT
CQLYGEYNVATNPEKKRLAEDIQYLMRNNNALRIAYVDKEVKPDGSEVFSSVLVKYDALLGSDVVIYRIQLPGPIKLG
EGKPENQNHALIFTRGDALQTIDMNQDNYFEEALKARNLLQEFTVRHGLRKPQILGVREHVFTGTVSSVASFMSGQ
ETSFVTLGQRVLANPLKVRMHYGHPDVFDRLWFLQRGGISKASSKINVSEDIYAGFNCTLRGGNVTHHEYIQAGKG
RDLGLSQIAVFESKVASGNGEQVLSRDVYRLAHRLDFFRMLSFYYTTVGFFISNLMVVITVYAFLWGRVYLALSGLEN
SVVSQSAFANTALTASLNQQFLVQLGILTALPMILENSLEHGCQSALWDFIIMQLQLASVFFTFSLGTRAHYFGRTILHT
GAGYRATGRGFVLRHEKFCQTYILFSRSHFVKGLELIVLLVIYQVFGAAGTVSTTAYLLITISTWFLAFSWILGPFLFNA
LGFDQLKLSQDFDEFWDWIWDRRELQRHKDAKNKKAEDEDENKKAEDEDDEETEDEDDDEDEDEDDDTETNPER
SWKAWWSKEYEHLNHTSFWGMIFEIVLNLRFLFLQYGIVYHLNIVGGHKSILVYVVSWVYMAVLAAIYMILLIAARKFS
LKQHLYYRLVQAIIVVLMLIVLIVLATKTSFQFFDTVLSVFAFLPTGWALLSIALVFRRFIERTPFWEIVISVARLYELFLGIL
VFIPLLVLSWIPGFQGMQSRILFSTAYVNKINIARLLQAKQPKKQSGYNRLHKI

>Cerri14-Ceratopteris richardii 1867
MASSQKHFNILPLTREEVDLWKEEANKASKELWKEANKASKELEEIMEANKVLNELSEFEEIKEAQKALKDAGKRLG
EIRMSNSDKQKDGDVFDMLRELFKFQEDNVRNQREHFLLLLANRIMRKSINCGLEEALKEAARHLNVKLLENYTQW
CSFLGRRANLQNLRRKLERQSEALVNLDLIYCSLFLLIWGEAANLRFMPECLSYIFHVLAQSLNTALDCKASPQATTV
HIDVWFPQESFLTEIIEPIYSAVRAEALGSKEGTAPHYSWRNYDDMNEFFWSKDCFTLSKECFAPVRDECKCDYLTN
LPERKERPANKDVTPGSDSEPSTSAALERTDTGKRRFKELVRSESVRVGKKIGFKVGFVEQRSFWNAFHSFHRLWI
AQILTLQVLLTLAFSNGNGKPWNQVTDPDIQFNFLYVFVTWAALRAVQALLTIIMQFTLISRNRKLLALRMVVKLAISFA

WIAIFSLLLARIQEERVKASETRRLVFIFLWSAGIFVIPVVFDVLIFLLPRLANKMELIVLLGSNKNIRRIFEWCFTDGNYV
GRGMHQSLVQSLQYAIFWLLIFAAKISFSYFFQILPMVGPTRQLKNLTSIQFKWFEPFPGNRFLLVCLWAPVILIYLMDT
QIWFSILAALTGAMVGLFSRIGEIRNIDQFRLRFQDMACALETNLFPKEPDDAGKKREGRWMMIRHGPGCLSRKRKP
SPSIKFAYLWNYLVKCFRKEDLVSNEEVELLSMDIDMEDLKRQPPMPSSNQVMEDLKTPRMLLSNQVILACQYAQHY
GEKVEKMYRGWREESPQKMEDECKRETQRLWEKVNSDKNMSAVVRTALVSLESLLVKDIWEEKQEPNEKKQLTE
MIKKIKRSFSNDKFLEEYDARELHQILFLVNNLLKVLVISHPESHVQGRQSEEQGQAHEQREEIMRALLELIKRLQWN
FPSVRSAELETSAELETDYEALIRAIRLPEKGSLRKLTMLKHLLEEKTTSLKLPRNSEARRRLAFFSNSLFMKMPRAP
SVKQMLPFSILVPYNNEDVMFTTKQVLERNEDGIAIQFYLRQIFPDEFKNMEERLDELTREIKEDEKIPIRIEQNRRWA
SYRGQTLARTLRGMVYYQNALEMLACLDDMPDNSREKFTAAPSEKHKALARSKFTLVVACQNYQRQQKETDPEQH
GKAVEMDKLLKENPFMRIAYVISKLEDNRKKSYYSVLAKYDQNCQKIVKIYEIKLPGDFMLGEGKPENQNQAIIFTRG
EALQAIDMNQENYFEEALKVRNLLEEFSPYKGKPPRVLGFREHIFTGSVSSLASFMSSQETSFVTLIQRVLAKPLRIR
MHYGHPDMFDRLWFLTRGGIGKASKAINLSEDIYAGFNCTLRRGHVIHREYMQVGKGRDVGFNQITRFEAKISGGN
AEQLISRDVYRLGKRLDFFRMSSFYYTTVGFYFSSLLVVLAGYVFLWGRVYIALSGVEDAIAAQSSFKNSALETVLNQ
QFVVQIGVFSSLPMLLENSLELGFTYATGNFLIMQMQLCSVFYTFSLGTKAYYFGTTLLHGGAIYKASGRSFVVKREQ
FITIFQQHSRSHFIKGIELIILLITHEIYSKISRNSTVYITMTIAYWFLAFTWLLAPFIFNPSGFDWLQTVYDYENFQEWLW
RRGGASSTAEESWEVWWNEQQEYIHHTSIWGKMAEVVVSIRFFLIHYGTVYRLKIAAHNKSILVYLVSWTFIVVSIVV
YIVVARAKENYGKRKYSYYRTIQCCVILFVCFVIGLLVGLTEFQFADLFISILGFLPTGWGILCMAAVAKPQLERYNRVW
GVVVDVARLYEMAIGIIVLVPVGILSWFPGFQHMQTRILFNQAFSRGLQISRLFTGKKVTKRTFLRDLASRRKIA

>Ginbi1-Ginkgo biloba 1937
MAGRSADPSTRRLSRTHTTGNLADSSTFDSEVVPSSLSSIAPILRVANEIESQCPRVAYLCRFYAFEKAHKLDPTSSG
RGVRQFKTALLQRLERDAEPTLALRKRKSDAREMQSFYQLYYEKYVKALDAAADQADRAELAKAYQTAAVLFEVLK
AVNQTEAAEVAPEIMEAGKDVEKKTEMYVPYNILPLDPAGAHQAIMLLPEIKAAVTALRNIRGLPWPANRQKSGTDQD
LLDWLQAMFGFQKDNVANQREHLILLLANVHIRQIPKPEPMSKLDERALNEVMKKLFKNYKKWCKYLGRKSSLWLP
TIQQEVQQRKILYMGLYLLIWGEAANLRFMPECLCYIYHHMAYELYGMLAGNVSPMTGENIKPAYGGDEESFLRKVV
TPIYAVIAKEAQRSRNITAKHSQWRNYDDLNEFFWSVDCFRLGWPMRADADFFGHPPEENHHHKKKGLRTDRKRW
LGKTNFVEIRSFLHLFRSFDRMWAFYILALQAMIITAWNGSGSPIAIFDGPVFKKILSIFITAAILRFLQAILDLVLSWKAR
QSMKFPQILRYVLKVVVAAAWVIILPVCYAHTWEHPTGLNKIVKSWVGHKWKSPSLYISAVVIYLAPNVLGAALFLFPM
IRRFFESSNWRIVNILMWWAQPRLYVGRGMHESQFSLFKYTMFWILLLITKLAFSYYIEIRPLVKPTQAIMSMSIGTFE

WHEFFPQAKKNIGVVIALWSPIVLVYFMDTQIWYAIFSTIFGGIYGAFRHLGEIRTLGMLRSRFESLPGAFNARLVPPN

VEKHEKRGLRTLLSRKFEQVEPGKRIEAAKFAQLWNQVITSFRAEDLINDREMDLLLVPYSSDPSLPRIQWPPFLLAS

KIPIALDMAKDFRGKDAELGKRIKADGYMRFAVEECYESFKHVLKLVVVGEQEKRVINDIIQEVEMNITNGTLLDAFKM

SELPLLYRKFEELLELLLKNDNDLSSREKVVLLLQDMLEVVTRDMIEDDSRILDLSHGSHSRPDDVTTLGPNRRPQLF

ATTGPDPAVRYPFPANDAWMEQIKRLHLLLTVKESAMDVPVNLEARRRIAFFTNSLFMDMPDAPKVRNMLSFSVLTP

YYKEEVLFSEKELQDENEDGVSIVFYLQKIYPDEWKNFLERVKCQTEQEVLDNDDNVQELRHWASYRGQTLTRTVR

GMMYYRKALELLSFLDMAKEEEILEGYKVVESVEDEHKKSQRSLSAQLKAVADLKFAYVVSCQQYGTQKRSSDPRA

TDILSLMVTHPSLRVAYIDEVEEREKDKTQKVYYSVLVKAVNQLDQEIYRIKLPGPAILGEGKPENQNHAIIFTRGEGLQ

TIDMNQDNYLEEAFKMRNLLEEFLQPHGVRPPTILGLREHIFTGSVSSLAWFMSNQETSFVTIGQRLLANPLKVRFH

YGHPDVFDRLFHLTRGGLSKASKVINLSEDIFAGFNSTLRQGNVTHHEYMQVGKGRDVGLNQISLFEAKIANGNGE

QTLSRDIYRLGHRFDFFRMMSCYFTTVGFYFSTLITVLTVYIFLYGRLYLVLSGLEKEMVKQASIQHNTPLQAALASQS

FVQLGMLMALPMVMEIGLERGFRTAASDFVVMQLQLAPVFFTFSLGTKIHYYGRTLLHGGAEYRATGRGFVVFHAK

FAENYRFYSRSHFVKGLELMMLLVVYNVFGHTYRTTVAYLLITFSMWFMVGTWLFAPFLFNPSGFEWQKIVDDWTD

WNKWINNRGGIGVPQDKSWESWWDKEQEHLNCSGLRGRICEIVLSLRFFLYQYGLVYHLKIAGKNKSVLVYGLSWL

VIVVVLLVLKTVSMGRRRFSADYQLMFRLLKGLLFIGFISVLIILYLVCNMTLTDLFACILAFMPTGWALLQIAQACRPLV

LRTGFWDSVRALARGYEFIMGLLLFAPIAVLAWFPFVSEFQTRLLFNQAFSRGLQISRILAGRKKDWSSTSKE

>Ginbi2-Ginkgo biloba 1940

MAENNQGPSIGQAPSRRLSRTHTTGNLAETAFDSEVVPSSLATVAPILRVANEIERVSPRVAYLCRFYAFEKAHKLDP

TSSGRGVRQFKTALLQRLERDAEPTLEARKKKSDAREMQSFYQQYYEKYVKALDAFADQTDRAQLAKAYQTAAVLF

EVLKAVNQTEAAEVAPEIMEAGKDVEKKTELYVPYNILPLDPASAHQAIMQFPEIKASVVALRNVRGLPWPENHQKT

GADLDLLDWLQAVFGFQKDNVANQREHLILLLANVHIRQLPKPEPMSKLDDRALNEVMKKLFKNYKKWCKYLGRKS

SLWLPSIQQEVQQRKILYMGLYLLIWGEASNLRFMPECLCYVYHHMAYELYGILAGNVSSVTGENIKPAYGGDEESFL

RKVVTPIYRIIAQEAQRNRSGTARHSQWRNYDDLNEFFWSVDCFRLGWPMRADADFFYVPPPHVRHPPQKETSTR

QGQRWMGKTNFVEIRSFWHLFRSFDRMWTFFILALQAMIIIAWNSSGSPTAIFEGGVFKKVLSIFITAAILKFLQATLDL

ILSWKARRSMTFYSILRYVLKVIVAAAWVIILPACYVHTWEKPTGLLKTIKNWVGQWKSSSLYVSAVVIYLVPNAVGAII

FLFPMIRRFIENSNWRVLTILTWWAQPRLYVGRGMHESIWSLSKYTIFWIMLLISKLAFSYYIEIKPLVKPTKVIMGMSV

GNNYEWHELFPQARNNIGVVISVWAPIVLVYFMDTQIWYAIFSTLFGGIYGAFRHLGEIRTLGMLRSRFQSLPGAFNA

CLVPPAVEKSEKKGLKTFLSRKFEQVEPGKRKEAAKFAQLWNQVISSFRSEDLINNREKDLLLVPYSSDSTIRIVQWP

PFLLASKIPIALDMAKDFIGKDVDLKKRIKADEYMRCAVEECYESFKHVLASVVVGEQEKSVINQIIKEVESSIESEHLL

ENFKMSELPLLYSKFVQLVELLLKNDEELTSREKVVLLLQDMLEVVTRDMMSDGSRILDSSHGSQARPEDVNMLGG

KPQLFASAGSQPAVRYPPPNTDSWVEQIKRLHLLLTVKESAMDVPSNLEARRRIAFFTNSLFMDMPQAPKVRNMLS

FSVLTPYYKEEVLYSLKELQLENEDGVSIVFYLQKIYPDEWKNFLERIECGTEEEVLRDADKVQELRHWASYRGQTLT

RTVRGMMYYRKALELQSFLDMAKDEEILQGYRALELAQEEHKKSQRSLWAQLQAVADMKFTYVVSCQQYGTQKRS

GDARAADILNLMVTHPSLRVAYIDEVEEREKEKTQKVYYSVLVKAVNQLDQEIYRIKLPGPANLGEGKPENQNHAIIFT

RGEALQTIDMNQDNYLEEAFKMRNLLQEFLEKHGVRYPTILGLREHIFTGSVSSLAWFMSNQETSFVTIGQRLLANP

LKVRFHYGHPDVFDRLFHITRGGISKASKVINLSEDIFAGFNSTLRQGNVTHHEYMQVGKGRDVGLNQISLFEAKVA

NGNGEQTLSRDIYRLGHRFDFFRMMSCYFTTVGFYFSTLVTVLTVYIFLYGRLYLVLSGLEKQLLHKAAVQHNASLEA

ALASQSFVQLGLLMALPMVMEIGLERGFRTALSDFVVMQLQLASVFFTFSLGTKTHYYGRTLLHGGAKYRATGRGF

VVFHAKFADNYRFYSRSHFVKGLELMMLLIVYNVYGQSYRNTVAYLLITFSMWFMVGTWLFAPFLFNPSGFEWQKIV

DDWTDWNKWINNRGGIGVPQEKSWESWWDEEQEHLKYSGLRGRILEILLSLRFFLYQYGLVYHLNIAHENRSVLVY

GLSWVVIAVVLLVLKTVSMGRRRFSADFQLMFRLLKGLLFICFVSVLIILFVVCGLTVADLFACVLAFMPTGWALLQIG

QACRPLVYRIGFWDSVRALARGYEYVMGLLLFTPVAILAWFPFVSEFQTRLLFNQAFSRGLQISRILAGRKKDWSST

SKE

>Ginbi3-Ginkgo biloba 1933

MASSGLGVSRRAVSRTQTATGGSFSSEVFDTEVVPSTLASIAPILRVANEIESERPRVAYLCRFYAFEKAHRLDASSS

GRGVRQFKTALLQRLERDNAPTLAARVKKSDAREIESFYRQYYEHYVRALDRAEQADRAQLAKAYQTAGVLFEVLC

AVNKSEKVEEVAPEIIAAARDVKEKTEIYAPYNILPLDAAGASQAIMQMEEIKAAVASLRNTRGLNWPPLFEQQRQNS

GDLDVLDWLRAMFGFQRDNVTNQREHLILLLANVHIRLVPKPEPLNKLDERAVDAVMSKLFKNYKNWCKFLGRKHS

LWLPKVQQEVQQRKLLYMGLYLLIWGEAANIRFMPECLCYIYHNMAYELHGMLAGNVSVVTGENIKPAYGGDEESFL

RKVVTPLYHIVEEEAKKNKNGTAPHSAWCNYDDLNEYFWSVDCFRLGWPMRDDSEFFRTPPDTGSLTHSQQPSEK

NAIRGTGKTNFVEIRSFWHVFRSFDRMWTFYILALQAMIIVAWNGSGSPMDIFQIDLFKQVLSIFITAAILRLIQGVLDIV

MNFPGYRSMKFVTMLRHFLKLLIAVAWVIILPTCYVHAWEKPSGIIRTIKIWLGQTGKMPSLYITAVILYLLPNILAALLFV

FPMLRRWIESSDWHIIMFLLWWSQPRIYIGRGMHESQLTLFKYTLFWVLLLICKLTFSYYVQIKPLVQPTKDIMNVTDV

RYLWHELFPNVPGNMGAVISVWAPVILIYFMDTQIWYAIFSTLSGGVSGAFRRLGEIRTLGMLRSRFQSLPGAFNAYL

VPSERLPRKGFSLARRFEEVSPSRRTDAAKFAQLWNEVISSFHEEDLISNREMDLLLVPYSSDPSLKLIQWPPFLLAS

KIPIALDMAAQFRAKDSDLWKRICAGEYMKCAVLECYESFKHVLNILVAGENEKRIIEIIIKEVEVNISKNTLLANFRMSA

LPIIHRKFVALVEILKTGEPSMRDTVVLLLQDMLEVVTRDMMVNEIRELLDSGHGRQESSHGRYDFVHAPQTNRQLF
AGIEPKPAVVFPPPATAPWIEQIKRLHLLLTVKESAMDVPVNLEARRRIAFFTNSLFMDMPRAPKVRKMLSFSVLTPYY
SEETVYSKSDLELENEDGVSIVFYLQKIFPDEWNNFMERLNCKKESEVWASEENVLHLRHWASQRGQTLSRTVRG
MMYYRRALKLQAFLDMATENEILEGYKAVALPTEEEKKSQRSLWAQLEALADMKFTYVATCQNYGLQKQCGDRRAT
DILNLMVNHPSLRVAYIDEVEEREGEKVKKVYYSVLVKAVNNLDQEIYRIKLPGPAKIGEGKPENQNHAIIFTRGEALQ
TIDMNQDNYLEEAFKMRNLLEEFHEDHGVRPPTILGVREHIFTGSVSSLAWFMSNQETSFVTIGQRVLASPLKVRFH
YGHPDVFDRIFHITRGGISKASRVINLSEDIFAGFNSTLRRGNVTHHEYIQVGKGRDVGLNQISLFEAKVACGNGEQA
LSRDIYRLGHRFDTFRMLSCYFTTVGFYFSTLLVVITVYVFLYGRLYLALSGLEKSLVNQADIRRNFPLAAALASQSLV
QIGLLMALPMVMEIGLERGFRSALSDFVIMQLQLAAVFFTFSLGTKSHYFGRTILHGGAKYRATGRGFVVRHEKFAE
NYRLYSRSHFTKGLELMMLLFVYNIYGTSAKDFLSYLLITLSMWFLVATWLFAPFLFNPSGFEWQKIVEDWEDWTKW
INNRGGIGVPANKSWESWWDEEQEHLQYTGYCGRILEVLLSLRFFLYQYGLVYHLNIANGSKNIIVYALSWLVIFAVML
VLKVVSIGRKKFSADFQLMFRLLKVFLFVGSIATLVTLFSVLHLTVGDLFASIVAFMPTGWALLQIAQASRPAVVTIGFW
DSVRALARGYEYVMGLVIFSPVAVLAWFPFVSEFQTRLLFNQAFSRGLQISRILAGGKKQN

>Ginbi4-Ginkgo biloba 1915

MARVETNWERLVRATLQRQQLRSGGRGPGRVSTGLVSSVPSSLGKTSIDAILHAADEIQAEDPNVARILCEHAYSLA
QNLDPNSEGRGVLQFKTGLMSVIKQKLAKRDGGGIDRSQDIARIQEFYKSYREKHRVDELQEEEMKLRESGPFSGD
LGELERRTVKMKRVYATLKVLGEVVEALTRDVAPEDADKLIPEELKRVMESDAAMTEELVAYNIVPLDAPAVTNAIVSF
PEVKAAVSALSYSGDLPKLPDDYSMPQLRNLDILDLLHYVFGFQKDNVSNQREHIVLLLANAQARLGILEEAEPKVDE
AAIQVVFVKCLDNYTKWCNYLCIHPVWNNLEALRKEKMLILVSLYFLIWGEAANVRFLPECICYIFHHMARELEEILRQ
SLAQPAKSCISDDGVSFLRQVISPLYETIAAEAASNDDGRKPHSSWRNYDDFNEYFWSLECFELGWPWRMDTAFFV
KPRKRVKAMQFSRGNERTGKSCFVEHRTFLHLYHSFHRLWIFLIMMFQGLTIIAFNDGKINLGTLKEVLSLGPTFFIMK
FIQSILDIMMMYGAYATTRSLAISRIFLRFLWFGIISGIFSYFYVKGLQERSQSNSNSFFFRTYVFILGAYAAAQLFVSFL
MRIPACRTLTNRCDQWSLVRFIKWMHQERYYVGRGLYERASDYLRYALFWLVVFGCKFSFAYFLQIKPLVSPTRTIVT
LKGLQYSWHDFFSKHNYNALTIASLWSPVLAIYVLDLYVWYTVLSAIVGALRGAKDRLGEIRSIETVHKRFEQFPEAFV
KNLQATRTKRRGNEASFSQVSIEASKSYAAKFSPFWNEIIKSLREEDYITNREMELLLTPINTGSLPLVQWPLFLLASK
VFLAMDLAVEGKDSQMELWDRISRDEYMKYAVVECYYSVGNVLKAIMDEEGRLWVERIFGDASESLQKESILVDFQL
NKLPLVLSRITALTGLLIHDETPDLAKGAVKAVQDLYDVVTHDIQSYDLREHIDDRRRIAKARNEGRLFSKLEWPKEPE
LREQIKRLHLLLTIKDSAANIPKNLEARRRLEFFTNSLFMKIPPARPVREMLSFSVFTPYYSETVLYSMAELKQENEDGI

STLFYLQKIFPDEWKNFLDRIGRDEGTLDSELEENSNDLLELRLWASYRGQTLARTVRGMMYYRKALVLQSYMERP
VFGDMEDGLTKDDIVNTHGYELSREARAQADLKFTYVVTCQIYGKQKQERKPEATDIALLMQRNEALRVAFIDVIETL
KDGKVYKEFYSKLVKADVHGKDKEIYSVKLPGDPKLGEGKPENQNHAIIFTRGDAVQTIDMNQDNYLEEALKMRNLL
EEFDVDHGLRPPTILGVREHVFTGSVSSLASFMSNQETSFVTLGQRVLANPLKVRMHYGHPDVFDRIFHITRGGISK
ASRVINISEDIYAGFNSTLRQGNVTHHEYIQVGKGRDVGLNQIALFEGKVAGGNGEQVLSRDVYRLGQLFDFFRMLS
FYFTTVGFYVCTMVTVLTVYIFLYGKAYLALSGMGGIIMHQADISGNTALEAALNTQFLFQIGIFTAIPMIMGFILEQGFL
KAIVTFVTMQLQLCSVFFTFSLGTRTHYFGRTILHGGAKYRPTGRGFVVRHIKFAENYRLYSRSHFVKALEVTMLLLV
YLAYGYDENGAITYILLTISSWFLAVSWLFAPYIFNPSGFEWQKTVQDFDDWTNWLLYRGGVGVKGEESWEAWWD
EEQAHIRSLRGRIFETILSLRFFVFQYGIVYKLHATGTDTSLTVYGLSWIVLAGLIVIFKVFTFSQKVSVNFQLLLRFIQG
ATFISVVTGLVVVVATTGLSVADVFASTLAFIPTGWGILCLAVAWKPIVKRIGMWKSIRSLARLYDAGMGMIVFIPIAICS
WLPFISTFQTRLLFNQAFSRGLEISLILAGNRPNTQL

>Ginbi5-Ginkgo biloba  1912
MARVETNWERLVRATLQRQQLRSGGRGPGRVSTGLVSSVPSSLGKTSIDAILHAADEIQAEDPNVARILCEHAYSLA
QNLDPNSEGRGVLQFKTGLMSVIKQKLAKKDGAPIDRSQDIARLREFYKDYRERHRVDELQEEEQKWRESGAFSG
DLQELERRTVKMKRVYATLKVLGEVVEALTMDATPEEADELIPEELKKVMKSDAAMNEEITTYNIVPLDAPAMTNAIGL
FPEVRAAVSALRYSRDLPQLPDEFQAPQLRSLDIFDLLHYVFGFQKDNVSNQREHIVLMIANAQARFGGLGDSEPRA
DEAPIHRVFLKSLDNYFKWCKYLRKRPVWNSLEALNKEKKLILVSLYFLIWGEAANVRFLPECICYIFHNMARDLDEIL
HQRLAQPAKCCIVENGVSFHQQIILPVYEIIAAEAGNNDNGKAPHSAWRNYDDFNEYFWSPNCFELGWPWNKDASF
FSKPKKRVKAIQAPHRTGKSCFVEHRTYLHLYHSFHRLWIFLVMMFQGLTIIGFNDGKINLGTVKEVLSLGPTYAIMKFI
ESVLDILMMYGAYATARGLAIARIFLRFLWFGVTSGFITYLYVKVLQERGKPNSNSSYFRIYALVLGLYAAAQLFVGLLL
RIPSYRALTNKCDQWSLVQFVKWMYQERYFVGRGLYERTGDYFRYVLFWLVVFGCKFSFAYFLQIKPLIRPTRTIVKL
KGLQYSWHDFVSKHNSNALTIISLWAPVLAIYVLDLQIWYTLLSAIVGGLIGARGRLGEIRSIDMVHKRFESFPEAFVK
NLQASNPIRLPSVATYAQAPEDTSKSYAAKFSPFWNEIIKSLREEDYITNREMELLLIPSNIGSLNLVQWPLFLLSSKIFL
AIDLAVECKDTQMDLWERISRDEYMGYAVVECYYNVEKILKSLLDSEGRLWVERLFRDANESVLDRSVLVNFQMNKL
PLVVSRITALTGLLGHDETPDLAKGVVKAMQDLYDVVTHDFLSVNLREQYDTWGILTKARNEGRLFSRIEWPSDLEIK
EQIKRLHLLLTIKDYAANIPKNLEARRRLEFFTNSLFMKMAPAKPVREMLSFSVFTPYYSETVLYSMTELRKENEDGISI
LFYLQKIFPDEWRNFLERIGRDETTLDSELQDNSNDLLELRLWASYRGQTLARTVRGMMYYRKALMLQSHMEKPLF
GDMEDGLSGVDITNTRGYELSREARAQADLKFTYVVTCQIYGQQKQKRAQEAADIALLMQRNEALRVAFIDIVESLK

DGKVFKEFYSKLVKADTDGKDQEIYSIKLPGDPKLGEGKPENQNHAIIFTRGDAIQTIDMNQDNYFEEALKMRNLLEE
FNSDHGLRPPTILGVREHVFTGSVSSLAWFMSNQETSFVTLGQRVLANPLKVRMHYGHPDVFDRIFHISRGGISKAS
RVINISEDIYAGFNSTLRQGNVTHHEYIQVGKGRDVGLNQIALFEGKVAGGNGEQVLSRDVYRLGQLFDFFRMLSFY
FTTVGFYVCTMMTVLTVYIFLYGKAYLALSGVDVAIVREAKILGNTALEAALNTQFLFQIGVFTAVPMIMGFILEQGLLK
AIVSFVTMQLQLCSVFFTFSLGTRTHYFGRTLLHGGAKYRATGRGFVVRHIKFAENYRLYSRSHFVKAFEVVLLLIVYI
AYGYANGGAVSYILLTISSWFLAISWLFAPYIFNPSGFEWQKTVQDFDDWTTWLLYKGGVGVKAEDSWEAWWDEE
QVHIRTLRGRIFETVLSLRFFVFQYGIVYKLHATGKDTAIVVYGLSWVVLAGFILIFKVFTFNQKVSVNFQLVLRFIQGA
TFIAVVTGLVIVVALTRLTVTDLFASILAFVPTGWAILCIAITWKPLVRSLGLWKSVSAIARLYDAGMGMLVFIPIAVLSWF
PFISTFQTRLLFNQAFSRGLEISLILAGNRPNTQL

>Ginbi6-Ginkgo biloba 1794
MSLRHRNSAQDSVSTATYSSEDEVYNILPVKNMLADHPALQYTEVRAAATALKSVRELRKPPSVQWNADMDLLDWL
GAFFGFQKDNVRNQREHLILLLANRQMQLQPPPIPIEQLDVRIVRTLRKTILKNYSSWCAFVGRKSSVLVSGRKRGVI
DERRELLYTSLYLLIWGEAANLRFLPECLCYIFHHMAMELNRILEDYLDESTGQPALPAYIGENAFLNKVVTPLYLTLKA
ETEASRGGKAPHSAWRNYDDINEYFWSKRCFAQLGWPLKLNSNFFVLSDSQPKQEGGLQRFKGMLHKQKVGKTG
FVEQRSFWNIFRSFDRLWIMYILFLQAAIIVAWNGSGIPWKELKHRDIQARVLSVFITWAGLRVLQSLLDAGMQYSLVS
RETPLLGIRMVLKSVVATVWTVVFAVLYSRMWDQRNHDLRWSGEANRRLVNYLEAALVFITPELLALLLFILPWVRNF
MEKTNWRLFHVLTWWFQTRTFVGRGLREGLFDNISYTLFWVALLAAKFSFSYFVQIKPMVSPTKATLKINVTTYKWH
EFFKNTNRFAVGIMWAPVILVYFMDTQIWYSIFSSVVGALVGLFSHLGEIRNIQQLRLRFQFFASAIQFNLMPEEPLFK
PKGTLRVKLRDAIRRLQLRYGFGAPYKKLESGQVEAGRFALIWNEIVSTFREEDIVSDLEVELLEVPPSSWNIRVIRW
PCLLLCNELLLALSQAKELQSTDKRLWRKICKNEYRRCAVIETYDSLKHLLQRIIREDSEEHSIIDSFFRDIDKSLQMEK
FTEKYRMSALPEIHTKLISLVKVLLTKPSEKDQQKVVDTLQNLYDIVIRDFLIEKQSTEQLREDGLISQRTGNKLLFEDA
VELPDVEDAAFYRQLRRLHTILTSRDSMHNVPKNLEARRRISFFSNSLFMNMPHAPQVEKMWAFSVLTPYYDEDVM
YNKEQLRTENEDGVSIIFYLKKIFPDEWSNFEERMCQKGWKGREEELWSNNNIRELRLWASYRGQTLTRTVRGMM
YYYKALKMLAFLDSASEMDIREGFKELVATASGSREDANGLNSQSFPSGRSLSSESSGVGMLFKDHEHATALMKFT
YVVACQIYGAQKAKKDPRADDILYLMKNNEALRIAYVDEVHAERDIVEYYSVLVKYDQDLQREVEIYRVRLPGPLKLG
EGKPENQNHALIFTRGDAVQTIDMNQDNYFEEALKMRNLLQEFTKYYGIRRPAILGVREHVFTGSVSSLAWFMSAQ
ETSFVTLGQRVLANPLKIRMHYGHPDVFDRLWFLSRGGISKASRVINISEDIFAGFNCTLRGGNVTHHEYIQVGKGRD
VGLNQISMFEAKVASGNGEQVLSRDVYRLGHRLDFFRMLSFYYSTIGFFFNTMLVVLTVYAFLWGRVYLTLSGLEAAI

LGTADTTNNKALGAALNQQFIIQLGLFTALPMIIENSLEHGFLSAVWDFITMQLQLASVFYTFSMGTRTHFFGRTILHG
GAKYRATGRGFVVQHKKFAENYRLYARSHFVKAIELGILLIVFASYSELSTDTFVYILLTISSWFLVLTWIMAPFLFNPS
GFDWLKTVYDFDDFMNWIWYEGGILTKADQSWEVWWNEEQDHLRTTGLWGKVLEILLDIRFFFFQYGIVYQLGISG
KSTSILVYLLSWIYVVVALAIYLVISYARNKYSAKQHIYYRAIQALVISITILVVVVLLEFTGFKFIDLVTSLLAFIPTGWGLIS
IAQVLRPFLQHTLVWETVVAVARLYEIAFGIIVMIPMAVLSWLPGFQSMQTRILFNEAFSRGLQISRIISGKKSNPGY

>Ginbi7-Ginkgo biloba 1791
MSLRHRNSAQDSVSTATYSSEDEVYNILPVKNMLADHPALQYTEVRAAATALKSVRELRKPPSVQWNADMDLLDWL
GAFFGFQKDNVRNQREHLILLLANRQMQLQPPPIPIEQLDVRIVRTLRKTILKNYSSWCAFVGRKSSVLVSGRKRGVI
DERRELLYTSLYLLIWGEAANLRFLPECLCYIFHHMALELNSILEDYLDKNTGQLALPAYSGENAFLRKVVMPLYLTVK
AETEASRGGKAPHSAWRNYDDINEYFWSKRCFEQLGWPLKLDGKFFLLPELPQMQHNEDFQRFRDMLQKQKVGK
TGFVEQRSFWNIFRSFDRLWIMYILFMQAAIIVACQGSGIPWVELRHRDTQVQALSVFITWAGLRVLQSLLDAGMQY
SRVSKETPLIGIRMVLKSIVALVWIIVFAVLYSRIWAQRNHDFRWSGEANRRLLDYLKAALVFITPELLALLLFVLPWVR
NFVEKTNYRIFHVLTWWFQTRMFVGRGLREGIFDNILYTLFWVGLLLAKFSFSYFFQIKPMISPTRAILKIKIIPYQWHE
LFRHTNRFAVGLLWAPVILVYFMDTQIWYSIFSSLVGALVGLFSHLGEIRNIRQLRLRFEFFANAIQFNLMPEELLFKIED
TLKVRVRDAIRRLQLRYGLGTSYKKIESSQVEVGRFSIIWNEIVGTFREEDIVSDLEVELLEVPPTSWNVRVIQWPCLL
LCNELLLALSQASEFEGTDKALWIKVCKNEYRRCAVIETYDSVKRLLQRIIKKDSEEHSIVMSLFDEIDASLKVEKFTEK
YRMSELPEIHSKVIALVSALLTKPSEKDQQKIVHTLQNLYEIVLRDFFKEKRSIGQLREDGPLSQRTSGKLLFEDALKLS
DVQDALFYRQLRRLHTVLTTRDSMHQIPKNLEARRRLAFFSNSLFMNMPRALQVDKMMAFSVLTPYYDEDVMYNK
EQLRTENEDGVSIIFYLQKIYPDEWSNFMERMQVDDPEELWIDKVKELRLWASYRGQTLTRTVRGMMYYHKALKML
AFLDSASEMDIKKGFQELVATALRPMEGANGCQNYTPGQSLRSESSRVDMLFKGSEDASALMKFTYVVTCQIYGTQ
KAKKDPRATDILYLMKNNKALRIAYVDEVGAGSEVVEYYSVLVKYDRHLEREVEIYRVRLPGPLKLGEGKPENQNHA
LIFTRGDALQTIDMNQDGYFEEALKVRNLLQEFTRYYGLRKPTILGVRENVFTGSVSSLAWFMSAQETSFVTLGQRV
LANPLKIRMHYGHPDVFDRLWFLTRGGISKASRVINISEDIFAGFNCTLRGGNVTHHEYIQVGKGRDVGLNQISMFEA
KVASGNGEQVLSRDVYRLGHRLDFFRMLSFYYSTIGFYFNTMLVVLTVYAFLWGRVYMTLSGVEEAIINVANTTNNK
ALGAALNQQFLIQLGLFTTLPMVVENSLEHGFLSAVWDSFTMHLQLASIFYTFSMGTRTHFFGRTILHGGAKYRATG
RGFVVQHKKFAENYRLYSRSHFVKAIELAILLIVYASYSVLSTNTFVYIILTLSSWFLVFSWIMAPFIFNPSGFDWLKTVY
DFYDFMNWIWYKGGILTKADQCWEVWWYEEQDHLRTTGLWGKALEILLDLRFFILQYGIVYHLGIANKSKSILVYLLS
WIYVVVALAIYLVITYARDKYAAKEHIYFRAIQFSVIGFTLLVIVVLVECTGFKIIDLVTSILALMPTGWGLISIAQVLRPVLQ

PTLVWETVVAVARIYDITFGIIVMIPMAILSWLPGFQSMQTRILFNEAFSRGLQILRILAGKNLDLGRQSR

>Gnemo1-Gnetum montanum 1961

MENSGNGQGPGQASGQAQPPMRRLSRTHTVGSITDSSAIDSEVVPSSLAFVMPILRVANEVESRNPRVAYLLRFHA

FEKAHKADPQSVGRGVRQFKTALLQRLERDQDTSLKARRHKSDAREITSFYNQYYQEYVKSLDSVADKANRAELTK

AFQTASVLFEVVKAVNQTESQELSPELMQAGRYVEEKKGIFKVYNILPLDPGGEHLAIMQYPEIQAAVIALRNTRGLP

WPNSEKQGDMLDWLQMMFGFQKDNVGNQREHLILLLANVHIRHQKQRTQQQQQKQKTQQQQGHQLDADAIKEV

MKKLFKNYKDWCKHLNRKSSLWLPVIEQEVQQRQLLYMGLYLLIWGEAANLRFMPECLCYIYHHMAYELYNVLGGT

VSATTGEQKSPAYGGEEEAFLRKVVTPIYNVIAKESERSKNGTAKHSQWRNYDDLNEYFWSKDCFKLGWPMHADD

DFFFVPENNRNWLQRHSRSRHSRWPGKTKFVEIRSYWHIFRSFDRMWSFYILCLQAMIILAWNGNHQLSGIFDDGIL

KKLLSIFITAAVLKFMQAILDIVFTWKARKSLKYYDILRYVLKLIVAAAWVVILPVCYAHTWKNPTGIVKTIKSWMGHRW

QSPSLYISAVVLYLAPNVLGAIMFLLPMVRRSVESANWRVFKFLLWWSQLPVYVGRGMQESSFDLFKYTLFWACLLI

TKLLFSYYVEIKPLIGPTRAIIKMHIHHFEWHEFFPGAKYNVGALIALWSPIILVYFMDTQIWYAIFSTIVGGIYGAFRRLG

EIRTLGMLRSRFRSLPLAFNDHLVPSDSDKQENRQNGTLMRALSKKYDQETHDLDTMIKFSQFWNEVISSFREEDLI

SDWERDLLLVPYTSDKDFAQIQWPPFLLASKIPIALDMAKSFNGRDEELWQRITKVDYMADAVKECYLLLKMVLFGV

VKGEPEKRVLGEIFDKVDKSIEKSDFTKDFNLRELPQLYKIFVELLKVLMENSENKKIVIRDRSNPNSNASSVVLLLQD

MLEVVTQDIVEIEALSNILDPSNSSSYSKQDKAKQQLFASPGAIKYPVQDKSVRWMEQIKRLNLLLTVTESAMDVPTN

LEARRRITFFTNSLFMDMPKAPKVRNMLSFSVLTPYYKEKVIFSQNELQLENEDGVSILFYLQKIYPDEWKNFLQRVQ

CKSEEEVLSDPANAEELRNWASYRGQTLTKTVRGMMYYRKALELQAFLDMAKVEDIREGYKSQMSTCDEKHERSL

SAQLHAVADLKFSYVVSCQQYGIQKRSADSRAQDILKIMINYPSLRVAYIDEIEEAHKDKPIKTYFSVLVKAGQNNLDE

EIYRIKLPGPANIGEGKPENQNHAIVFTRGEGLQTIDMNQDNYLEEAFKMRNLLEEFRERHGVHPPTILGLREHIFTG

SVSSLAWFMSNQETSFVTIGQRLLANPLKVRFHYGHPDVFDRLFHVTRGGISKASKGINLSEDIFAGFNSTLRKGNV

THHEYIQVGKGRDVGLNQISLFEAKIANGNGEQTLSRDVYRLGHRFDFFRMLSCYFTTVGFYVSSLITVLTVYIFLYG

RLYLVLSGIEKVMVKKASAQHNVSLQVALASQSFVQIGLLMALPMVMEIGLERGFRNALSEFVIMQLQLAPVFFTFSL

GTKAHYYGRNLLHGGAQYRATGRGFVVFHEKFADNYRFYSRSHFVKGLELTMLLVIYKIFGQSYENTVAYVLITFSM

WFMVGTWLFAPFLFNPSGFEWQKIVDDWADWNRWINNRGGIGVPVLKSWESWWEKEQEHLKHTGFSGRLFEIILS

LRFLIYQYGLVYHLHITKHKKSILVYGLSWLVIVVVLVFLKVVSMGRRRFNANIQLVFRLVKGILFLGFVAVLVVLYILLGM

KFGDLFICILAFMPTGWALLQISQACRGVVERLGIWDSVRALARAYEYIMGLLLFAPVAILAWFPFVSEFQTRLLFNQA

FSRGLQISRILAGRKKDWSSSSRE

>Gnemo2-Gnetum montanum 1955

MESTSKEPGAGTSSGGEGARPRINRRLSRTQTVGNLITDGAIDSEVVPSSLLAIAPILRVANEIENEHPRVAYLCRFHA
FEKAHKLDPSSSGRGVRQFKTALLQRLEKDNDKTMNLRKKRGDFREMESFYRQYLNNYVKAREEASDHADRQQL
AKTYNTANILFEVLMGVNQTEASAEISPEIIQAGKDIEKKTEMFSNCNILPLDPAGAEQPIMQFPEIKAAVTALRNIRGLP
WPEDQQKPKEDLDLLDWLKAIFGFQKGNVNNQREHLILLLANVHIRKVTSLSKLDDSALNEIMKKLFKNYKQWCKYL
KLKSSLWLPTNHIEQEVQQRKMLYVGLYLLIWGEAANLRFMPECLCYIFHHMAYELYGILAGNVNSVTGENMRPAYG
GEEESFLKHVVTPIYEVIRKEVERNRSGTAKHSQWRNYDDLNEFFWARDCFKLKWPMDRNHKFFYVPEDEHYPKK
GHTELSSKVHNGARKNLWMGKQNFVETRSFWHLFRSFDRMWTFFILALQVMIIIASHGSGSPSEISHGSVFKEVLSI
FITAAILKFLQAILDVFFSWKARHSMKRLQIMRYIMKVIVAAVWVIVLPVCYARSWENPTGLVKTVESWVGGGQRMPS
LYIIVVMIYLIPNAIAMLLFLFPMLRRWIESSDWSICNAITWWQQPRLYVGRGMHESIFSLIKYTMFWALLLICKLTFSYY
VEIKPLVKPTKVIMQMHIVNKYEWHELFPQAKYNIGVVIAIWAPTILVYFMDTQIWYAVFYSIFGGIYGAFRHLGEIRTLE
MLRSRFLSLHGAFNERLIPPSEGKVPTNGLQALFLRKYDKPKPEKRIEAAKFAQLWNQIITSFREEDLITNNEMLLMIV
PYSSDSELNIIQWPPFLLASKIPVALDMVKDFKGTYSDLENRIKADHYMHCAVEECYQSFKRVLRRLIFGEHEKKVIEE
IFLFVDGNIDNRTFLNSFKLSGLQLLYDRFTQLLNLLLQNDASSTNNIIILLQDMLEIFSKDIARDPYSVVDSTHGHVSQ
EEMDPLGNAGKRQLFASAGSKPAISYPLNSNDEAWMEQLNRLNLLLTVKQSVMDVPTNLEAQRRVTFFTNSLFMN
MPNAPKVRNMLSFSVLTPYYDEDVLYSQSELWDENEDGVSILFYLQKIYPDEWKNFIERIECENEEEILGNPELVAFL
RLWASYRGQTLARTVRGMMYYRKALELQSYLDMANVDDLLKGYKALEQAEDEKASGHSLWTQAQAVSDMKFTYV
VSCQKYGIQKRANDKRANDILGLMLKYPSLRVAYIDEIEDTEKDNKQKSYHSVLVRGGNSLDQEIYRIKLPGPPVLGE
GKPENQNHAIIFTRGEALQTIDMNQDNYLEEAFKMRNLLEEFLTRHGVRYPTILGLREHIFTGSVSSLAWFMSNQETS
FVTIGQRILASPLKVRFHYGHPDIFDRLFHLTRGGISKASKVINLSEDIFAGFNSTLRKGNITHHEYMQVGKGRDVGLN
QIALFESKVASGNGEQTLSRDIYRLGHRFDFFRMLSCYFTTVGFYFSSLITVLTVYSFLYGRVYLVMSGLEKAMFHEA
ALQHNTSLEASLASQSFVQLGLLMVLPMVMEIGLERGFRSALTDFIIMQLQLASVFFTFSLGTKTHYYGRTLLHGGAK
YKGSGRGFVVFHAKFVDNYRFYSRSHFVKGLELMMLLIIYNVYGETYRNAIGYILITFSMWFMVGTWLFAPFLFNPS
GFEWQKIVDDWADWGKWINNSGGIGVPQEKSWESWWHDEQEHLNYSGFLGRTIEILLSLRFFLYQYGLVYHLHISG
QHKSFLVYLLSWLVIAVVVLVSKAASIGREKVKWHQLLVRVMKALFFTSFVSILIVLLVVFNLTVGDMFACVLAFMPTG
WALLQIAQVSKRLVDRLGLWDSVRELARCYEYVMGLLLFAPIAVLAWFPFVSEFQTRLLFNQAFSRGLQISRILAGKR
KDRSSANAR

>Gnemo3-Gnetum montanum 1998

MASTSASSSAPGDAAGGRRLQRTQTIADIAAEATKDSELVPTSLTSIAPILRVANDIEEENPRVAYLCRFHAFDKAHRS

DPTSSGRGVRQFKTALLQRLERDQEKTKKERRHKSDAREIQSFYKRYYDTYVRALDAAATQTADRAELAKRYQTAAI

LFEVLKAVNQAESGGELTAEIIETGQDVVKKTEIYVPYNILPLDPQISQYPQHQIAQEASMQFPEIQAAIAALRNTRGLR

LPEAHQSSKSDWDLLDWLQLTFGFQKANAANQREHLILLLANVHIRDSSKMKSRRNSATAKRDNMLDDEALEFVME

KLFKNYKNWCKYLKRNCNIQNPNIDLERRQRKILYMGLYLLIWGEAANLRFMPECLCYIFHNMAYEMLGLMDRNTTK

VTGEPIKPAYGDEEEAFLNKVVTPIYEVIAKEVERNKGGKAKHSEWRNYDDLNEFFWSEECFTLKWPMITDHEFFNV

PGVAESNSSKVSNLAKSKAPKRWMGKTNFVEMRSFWHLFRSFDRMWTFFILSLQAMIIIAWQAPGSPSELFTGPVF

KQVLSVFITASLLKLLQAILDIIFSWKARHTMRFREVLRLILKLVVAVAWVVILPFCYVHTWDHPTGLIRTVEHYFGDWE

NNSLYIIAVVFYLIPNIISVILFFLPSLKRQVENSHYCIFHMLTWWQQPKLYVGRGMHENTTCLIKYTTFWILLLTSKLAF

SYFVEIKPLVRPTKKVMKLSIHNKYEWHELFPQAKYNFGVVISLWAPVILVYFMDTQIWYAVFSTIFGGISGAFRHLGEI

RTLGMLRSRFQSLPAAVNASLVPPKRGHASGLKSFWKTFEKAKPGRREEAAKFAQLWNQVVDSFREEDLINDGEK

NLLRAPYSSDPDLKVVQWPPFLLASKIPIAVDMVKDFKSKDDKDLLSKIGHDIYMISAVKESYGSFKHVLNSIVIGEKE

KMIIEKILLEVDSYINRSLLLEKLKMKELPKLYAKFVDLVDILLKSIQDQNSKKNLEKNLKIRNEQKLNMDAEQKSRIENE

EKTRHKMIILLLDMLEVFIRDMVEDGNRFIDPIIGQESAHGALTVDQLQKLQLFASVGDEHHVLYPLEQPGEKNEAWK

EQIVRLHLLLTVKESAMDVPANLEARRRMAFFANSLFMDMPPAPKVRTMMSFSVMTPYYSEPVLYSLDELQLLNED

GVSILFYLQKISPDEWKNFLQRIDCRTEEEVLSSEENKQHLRLWASYRGQTLARTVRGMMYYRKALELQAFLDLADI

RDIMEGYRVIKLQEEEDRHQRSLWAQLQAISDIKFTYVVSCQIYGAQKKRRDPCAADILYLMNVYPSLRVAYIEEGET

MVKEKPCKVYYSVLVKGVDQRDQEIYRIKLPGRANLGEGKPENQNHAIIFTRGEALQTIDMNQDNYLEESFKMRNLL

QEFLTRHGVNYPTILGVREHIFTGGVSSLAWFMSNQETSFVTIGQRVLASPLRVRFHYGHPDVFDRIFHLTRGGISKA

SKGVNLSEDIFAGFNSTLRGGNVTHHEYMQVGKGRDVGLNQITMFEAKVANGNGEQTLCRDLYRLGHRFDFFRML

SCYFTTIGFYFSTLITVLTVYVFLYGRLYLALSGLEKAIRDAASLQHDSGLEAALVSQSFVQLGILMALPMVMEIGLERG

FRTALSDFVIMQLQLASVFFTFSLGTKTHYYGRTLLHGGAKYRATGRGFVVYHAKFADNYRFYSRSHFVKGLELTLLL

VVYKVYGASFNNTLGYMLVTFSMWFMVVTWLFAPFLFNPSGFEWQKIVDDWTDWNKWINSRGGIGVSPEKSWES

WWEEEQEHLKHSGLAGRLWEIVLSIRFFLYQYGMVYHLNIAHERRSFLVYGLSWVVIGVILLVLKTVSMGRRRFSAD

FQLIFRLLKGLLFISFVSVLTILFVICNLTVSDLFACVLAFMPTGWAFLQIALACRPLVQHTGFWDSVRGLARGYEFVM

GLVLFTPTAVLAWFPFVSEFQTRLLFNQGFSRGLQISRILAGGKRDWSTKNAD

>Gnemo4-Gnetum montanum 1911

MATSTSLEKLPRSSTVTRGEATEIVPPSLATLSSILRVANHVELESPRVAYLCRLYCLEKAQRLDPASRGRGVRQFKT

GLLQRLERENEPTLSLRVERSDAREVESFYREYYENYVKALDKDDEADRALLSKSYQTAGVLFEVLCAVNKCEKVE

EVAPEIIAAARDVQEKEEIYTPFNILPLDAAGSSQAIMQMEEVKVAVASLRNIKGLHWPHMFQQNPQKTVELDLLDWL

QAMFGFQKDNVRNQREHLILLLANGQARLTPRPETGNQLDEYALDVVMKNLFKNYKSWCKFLGRGHSLSIPEGRQ

AAQQGKLLYMALYLLIWGEASNIRFMPECLCYIFHNMAYELHGLLAGNVSVVTGENIQPAYGGDEESFLKKVITPLYS

VIEKEAKKNKNGSAPHSAWCNYDDLNEYFWSVDCFRLGWPMRDDNDFFCTASDLGPAIHLTRPYTRVKAKGTAKR

NFVEERTFWHIFRSFDRMWTFYILALQAMIIVAWSGSGSPMDIFETKVSQNVSSIFITAALLRLIQSFLNFFMNLKAFQ

SMSFSDCLRYILKVMISTAWVLILVACYVHAFEESASIVKIIKSWIGKTWDMPSLYISAVSLYLIPNILAAFCFLFPLSRRS

VEKSDWLVTKFLLWWSQARIYTGRGMHESQCTLSKYTLFWVLLIICKLAFSYYIQIKPLVLPTKALLKIRNVEYQWHEA

FSNVHWNVGAIISIWAPVIMIYFMDTQIWYSIFSTLIGGLLGASRRLGEIRTLGMLRSRFHSLPGAFNANLIPSERRLQR

GFSLARRFEEVSFSQRIEAAKFAQLWNGVISSFREEDLISNREMDLLLVPYSADPNLKLIQWPPFLLASKISIALDTAVQ

FKGKDSDLWRRISSDHYMKCSVIECYELSKHVLRTLVNGVNERRIIEILVSEVESSISRNTLLEDFRMSALPTVYSKFV

ELVEILKIGDPSRHDNVVLLLQDMLEVVTKDMMVDETGELLELVQGKLESNKQLFAGTEPSPAILFPPPSTASWIEQV

KRLHLLLTMKEYAMDVPMNLEARRRIAFFTNSLFMDMPCAPKIREMLSFSVLTPYYSEETLYSKSDLELENEDGISIM

FYLQKIFPDEWTNFMERMDCKTEAQVWGSAENVYHVCHWASQRGQTLSRTVRGMMYYRRALKLQAFLDMATEN

EILQGYKTVAFPSEDDRISQKSQWALLDAMADMKFTYVVTCQNYGTQKQNGDRRALDILDMMIRNPSLRVAYIDEVE

YRGNDEVKTVYFSVLVKAVNGVDQEIYRIKLPGPPKIGEGKPENQNHAIIFTRGEALQTIDMNQDNYLEEAFKMRNLL

QEFHEDHGVSSPTILGLREHIFTGSVSSLAWFMSNQETSFVTIGQRLLANPLKVRFHYGHPDVFDRIFHITRGGISKA

SRVINLSEDIFAGFNSTLRGGNVTHHEYIQVGKGRDVGLNQISLFEAKVACGNGEQALSREIYRLGHRFDMFRMLSF

YFTTVGFYVSCMLVVLTVYVFLYGRLYLAISGLEESLLIQADIRNDISLEGVLTSQSLVQIGLLMALPMLMEIGLERGFR

SALGDFIMMQLQLASVFFTFSLGTKTHYFGRTILHGGARYRATGRGFVVRHEKFAENYRLYSRSHFTKALELMMLLL

VYNIYSITRARTIPYVMSNGFTWFMIVSWLFAPFLFNPSGFEWQKIVEDWEDWCRWMNNKGGVGVPANKSWESW

WKEEQQHLRHTGFCGLVLELVLSVRFFLYQYGIVYHLHIAKENKSIIVYGLSWLVIAVALLILKVLSIGRKKFSADFQLM

FRIVKLLLFLGCIATIAVLFCAVGLTMGDLFASTLAFLPTGWGLLQIAQAWRPVCVMFCCWDSVRALARAYDYMMGITI

FTPIAILAWFPFASEFQTRLHFNQAFSRGLQISRILSGGKKRV

>Gnemo5-Gnetum montanum 1880

MTNAERNWKRLVEASLQSQWLLSETSDLAFGLPSSLQSSNINEILKVADQIQEDGLTVARILCEYAYSLAQQLDPNSE

GRGVLQFKTALMAITKQKLLMKDKGELDRSQDLVKLQEFYLRFREQRKDEATSSESSKGQRFEKNQEHVWKFYTSL

KVFGDAIMSIAREMDSENLEKLIPDKLRRMINSDATVMGLSTYNIVPLGVLAKTHPISLLPEVRATISALLYSEDKLILPT

KHHFPQIKKPDILDILQHIFGFQDDNVKNQREHIILLLSNAQFRLSKPEGEDPKLDEEAIRVVFENALSNYINWCSYLGI

PHWDNVAALPSWKKLLLLSLYFLIWGEAANVRFLPECLCYIFHRMSQELEEVLRKKVAEPAQSCRGDDGGSFLKQVI

IPIYDVIAAEARYCVKWKPPHSAWRNYDDFNEVFWSKECFKLGWPWNKDAPFFTKPEITKKPSLVSNHTQKTNFVE

HRTFFHLYHSFHRLWIFLILMFQVLTIIAFNDEVISQNTIKEVLSIGPTYMFMKLLKSILDIMMLCGAYKATYNVLILKVLIR

CILFGILAGATTFSYVIGLQGKTSFAMYLSIIGAYVAFQILSFILQVPACQMITDNFDQSSVVQIIKWMQKDIYYVGRGMY

ESTTGYISYACFWLSIFICKFLFSYELEIKPLVKSTQIIVGLKQVQYAWHDLVSKNNHNALTIVSIWAPVVSIYLLDLYIWY

TVFSAVVGVAVGAKQKLGEIKTMRSLRQQFDRFPVAFRDLQTSRHIPNRRNVPQTQVSEDENKTYVAEFSPFWNEII

KHLREEDYITNREKDLLYVPSNKGNFNLIQWPLFLLVNKIFLAIKLTAKRRNREASQQELWRKISEDKYMKYTVMELYY

LIKLIMDTILKDEGKLWADRVFEDITEALRKGSFVEDFDLHGLENVQNAIIALTVALMAEEKQDKQKTAVKAMRDLHDIV

THDLCSSYDKRHHSDTWLKIAKAKKTGTLFSKLELPEDPELKTQIKRLYLLLTSKGSAADTPKNSEARRRLEFFTNSL

FMNMPPTKPVQEMLSFSVLTPYYQEAVMFTVDELENENKDGISILFYLQTVFKDEWENFMERIGLNKNAYISKLKEN

PNHLLELRLWASYRGQTLARTVRGMMYYRKALMLQCYLECQMNHEEKQGYESSQFAKAWADLKFTYVISCQKYGI

QKRSNKQEEKAKATDIALLMQRNEALRVAFIDVVETFKNGQVYKEYYSALVKADAKGKEKEIYRIKLPGDPILGEGKP

ENQNHAIVFTRGDAIQTIDMNQDNYFEEALKMRNLLEEFNRNHGLRLPTILGVREHIFTGSVSSLALFMCNQETSFVT

LGQRILASPLKVRMHYGHPDVFNRIFHITRGGISKASRIINISEDIYAGFNSTLRQGNVTHHEYIQVGKGRDVGLNQIS

MFEGKVAGGNGEQVLSRDVYRLGQNFDFFRMMSFYFTTVGFYVCTLMTVLTVYVFLYGRAYLALSGVGESILKSSR

DIQDNKALNAALSTQFLFQIGVFTALPMIMGFILEEGLLKAIVSFLTMQFQLCSVFFTFSLGTRTQYFGRVILHGGAKY

QETGRGFVVQHIKFKENYRLYSRSHFVKAFEIMILLIIDANYSYHKQGTFAYILLTISSWFLVISWLFAPFIFNPSGFELTK

ITTDFRNWMKWLNNRGGIGMNAKKSWETWWNEEYAHIRTLKGRIFETILCLRYFFFQYGIVYKLHVTNDDTRLSVYG

LSWLVILGVIILFKLYTLSEKTTANFQLPARLLQGSLTVGVLTAVVILIITTKMTVTDVLASILAFVPTGWGILSIAIAWKPVT

KKLGLWKSVRSVAWVYDIVIGAVVLAPIATLSCIPFIATTQTRLMYNQAFSRGLEVSLILRGDKLHD

>Gnemo6-Gnetum montanum 1912

MTTTVRAEENWKRLVQAALRASRGPGHGAVSGGLAATVPSSLNAANIDAILNAADEIQAEDANVARILCEHAYSMAQ

NLDPNSEGRGVLQFKTGLMSLIKQKLSKREGVLDRSQDLARIQEFYKHYREKHKVDELQEEELRMQESGTYSGDY

KELEKRARNMKRVFATLKVLGDVVEAMTSDVAPDQAEALIPEQLKRVIKSDAEKTEGLYDFNIVPLDAPAMANPISLF

PEVKAAISALSSIGGLPSLPVDYEVPQTRKPDILDLLQYTFGFQKDNVKNQREHVVLLLSNAQARLRIPEEIDPKLDEA

AVHMVFQKSLENYVKWCSYLNIPQRHSFESIQKEKKLMLVSLYFLIWGEAANVRFLPECICYLFYQMSRELDEIMEQ

QVPQPAKSCIGEDGVSFLQQVILPLYESLAAESAVCDNGKEPHSAWRNYDDFNEYFWSRNCFELGWPWNKESSPF

LFKPSKKGSKVMKISRRRHGMGKTCFVEHRTFFHLYHSFHRLWIFLAMMFQALTIIGFNDSKISWRTIRELLSVGPTF

MIMKFIESILDIVMMYGAYAATRSVALSRIFLRFLWFGALSGFISYLYVKGLQDYSARNSSSFIFRVYVVVLGIYAVSQLF

FSFLMRIPACHMLTERCDKWSLVRFLKWMHQEHYYVGRGLYERPFDYLRYVFFWIIIFGCKFAFAYFLEIRPLIAPTRT

ILRLKALRYSWHDLVSRNNHNALTIASLWAPVFAVYILDFYIWYTVLSAIVGALIGAKERLGEIQSIAALHKRFATFPEAF

VKSLQVAKMKGDGAIVPHGQVNDDLQKRNYVSRFSPFWNEIIKCLREEDYITNREKELLCIPSNSGRLTFVQWPLFL

LVSKIFLAQELAAENDSQSSIWEKVSKDEYMKYAVSECYSSVENILKEILDEEGKLWVNRVFDDIRASIEKVTLPQDFQ

LGKISLVLSRIIGLTGLLVREETADTVKGAVQALQDLYDVVTHDILSYNIREHYDTWRNLAKARIEGRLFSKIEWPKDSE

LKQQIKRLHLMLTVKESAANIPKNLEARRRLEFFTNSLFMKMPSSRLVREMLSFSVFTPYYSEIVMYSMNELNRENE

DGISTLFYLQKIFPDEWKNFLERIGRDDLTRESELQENPTDLLELRLWASYRGQTLARTVRGMMYYRKALVLQSYIER

VQNNDIENGLARSDIQSIEDYELSRDARAQADLKFTYVITCQIYGKQKQDKKPEAADIALLMQRNEALRVAFIDVVETL

KDGKVHKEFYSKLVKADVHGKDKEIYSIKLPGDPKLGEGKPENQNHAIIFTRGNAVQTIDMNQDNYFEEALKMRNLL

EEFDCDHGLRPATILGVREHVFTGSVSSLASFMSNQESSFVTMGQRVLASLKVRMHYGHPDVFDRIFHITRGGISKA

SRIINISEDIYAGFNSTLRQGNITHHEYIQVGKGRDVGLNQIAMFEGKVAGGNGEQVLSRDVYRLGQLFDFFRMLSFY

HTTVGYYFCTMLTVLTVYVFLYGKAYLALSGVGGQIRDRVKNNASLDAALNTQFLFQIGVFTAVPMIMGFILEQGLLKA

IVSFVTMQLQLCTVFFTFSLGTRAHYFGRTILHGGAKYRATGRGFVVQHIKFAENYRLYSRSHFVKALEVAMLLLVYT

VYGYIDSGVLSYILVTFSSWFLVISWLFAPYIFNPSGFEWQKTVKDFEDWTNWLLYRGGIGVKGEESWEAWWDEEQ

AHIRSIRGRIFETILSLRFFMFQYGIVYKLNATGRNTSLTVYGFSWLVLLGLILIFKVFTFSQKVSVNFQLLLRFVQGATFI

SVIIGLGAVVLFTDLTIGDLFASILAFIPSGWAILSIAIAWKPIIKRLGLWKSVRSLARLYDAGMGMVVFIPIAICSWLPFVS

TFQTRLMYNQAFSRGLEISLILTGNKQNFDDV

>Gnemo7-Gnetum montanum 1910

MSRVEKNWERLVRATLRRQQLRAGGRAAVGRAGLIDHVPSSLGKGSNIDAILHAADEIQAEDRVVARVLCEYAYTLA

QELDPNSEGRGVLQFKTGLMSIIKQKLAKREGAVIDRSQDFARLWEFYEAYRARHKVDELQQEEQQWRESGAFSG

QVKDFERRSAKMRRVIATLNVLADCIGHLTRDISAEESGIVIQDELRRKMQMDKAMSEEISTFNIVPLDAPSFTNAIRHI

SEVKAAISAVSLSRELPQLPDDFPKPQKRSLDVFDLLHYIFGFQKDNVRNQREHVILMIANSQSRLGVPEGPGLSIDE

AAIQGVFGKALRNYIEWCKYLRKRPAWERRLEPLNKEKRLLLIALYFLIWGEAANIRFLPECLCYIFHNMVIELAEDMS

KEEIAQRAKSCTTEDNASFLENIITPLYQVIAHEASNNDNGKAPHSAWRNYDDFNEYFWSASCFELHWPWNRDTKF

FSKPPKKQGTCIQRAGKVSFVEHRTFLHLYHSFHRLWIFLVMMFQGLTIIGFNDGKINLGTVKDLFSLGPTYVFMKFIE

SVLDIMMLYGAYSTTRGLAIARVFVRFVLFGAVSVLITYIFVKIQEETGDLNLDSFYVKIYLLIMGVYSALYFVFSLLLRF

PSVRSVTAKWDRLFCIQMIKWMFEEHYFVGRGLYERASDYCRYVLYWVVILACKFCFTYFLQIKPLVQPTREIVKLKN

LRYSWHDFFSKNNHNALTIASLWAPVFSIYLLDLYVWYTILCAIIGGLIGARDRLGEIKSIEMVHKRFESFPEAFATNLLS

GESNRSQADRAAQVSDRNKIYAAKFSPFWNDIIKSLREEDYITNREMELLLIPPNTGNINIVQWPLFLLSSKIFLAMDL

ALECKDTQKELWERISRDEYMAYAVLECYHSVENIMKSLLENEGKQWLEKLFHDINESIEVGSVLVSFQMNKLHLLVS

RLTALTGLLIHDHTSEQEKGAVKAMQELYDVVIHDILSTELSDRYETSGIFARARSQGRLFSRIRWPRDTEMKEQVKR

LHMLLKRKDFAANIPKNLEARRRLEFFTNSLFMNMPSAKPVREMIPFSVFTPYYSETVMYSVPELRKENEDGISILFY

LQKIFPDEWSNFLERIGRDDSTMDSDLLENPSDTLELRLWASYRGQTLARTVRGMMYYRKALMLQSYLERSSYGD

MEAAYSTASVADAAGYELSLEAKALADLKFTYVVTCQIYGQQRQQKKAEAADISLLMQRNEALRVAYIDSVDESKDG

KSCKHFYSKLVKADAEGNDQEIFSIKLPGDPKLGEGKPENQNHAIIFTRGDAIQTIDMNQDNYFEEALKMRNLLEEFK

GNHGLRPPTILGIREHVFTGSVSSLAWFMSNQETTFVTLGQRVLAYPLKVRMHYGHPDVFDRIFHITRGGISKASRVI

NISEDIYAGFNSTLRQGNITHHEYIQVGKGRDVGLNQIALFEGKVAGGNGEQVLSRDIYRLGQLFDFFRMLSFYFTTV

GFYVSTMMTVLTVYIFLYGKTYLALSGVDRAIARVADTSSNTSLESVLNTQFLIQIGVFTAVPMVMGFILEEGLLKAIVS

FVQMQLQLCSVFFTFSLGTRTHYFGRTILHGGAKYRATGRGFVVRHINFADNYRLYSRSHFVKGFEVVLLLTVYVAY

GYVRGGSTTFILITVSSWFLAISWLFAPYIFNPSGFEWQKTVQDFDDWTSWLLYKGGVGVKAEDSWETWWDEEQA

HIRTVRGKLLETILSLRFFVFQYGIVYKLHATRSYTNLTVYGVSWLVLVGFVMIFKVFTISRKVSVNFQLMVRFIQGVVF

IAVIAALALVVALTSLSVTDLFASILAFVPTGWAVLSLAITWKSCVKKLGLWKSVSSIARLYDAGMGMLLFVPIAFLSWFP

FVSTFQTRLLFNQAFSRGLEISLILAGNRPNVEA

>Gnemo8-Gnetum montanum 1794

MSTSQRSSAPPRIPVDVSSSHQYETYNIIPLDNMLADHPALRFPEVRAAVTALKTTGDLRPPPFVRWSPSYDLLDWL

GLFFGFQKDNVKNQREHLVLLLANSQMRLQPPPDIIDKLEPGIVKKLRKKILGNYETWCSFLGRKSNVRASQRRRSE

SDKRRELLYISLFLLIWGESANLRFIPECLCYIFHHMAMELNKILDGYVDGSTGQYCLPSHRGENAFLIHVVTPLYETV

QAEADASGGGKAPHSAWRNYDDINEYFWSNRCFTHLGWPMRRDSNFFVQPKKEETTMQKLRGLMQKQKVGKTG

FVEQRTFWNLFHSFDRLWILLILFLQAAILVAWEDRGAPWNVLKRKDAQVKMLSLFITWAGLRLWQALLDAGMEFNL

VSRETPLQGFRMLLKILTAAVWSILFTVLYVRTWNQRNHDFRWSPEADRRLKNCLWAAAIFILPECLALVLFIVPWIRN

FVEKSNFKIFHLLTWWFQSRSYVGRGLREGLVDNLYYSLFWVLLLAVKFAFSYQFQIKPMVEPTKEILKVKHIKYRWH

EFFKKTNHLAVGLIWAPVILIYFMDTQIWYSIMSSIIGALVGLFDHLGEIRNIQQLRLRFQMFASAIQFNLMPQEPLFRQ

SDSLNVRIKDAWKRFRLRYGLGTAFKKMESNHAEAGRFALIWNEIIKTFREEDIVNNREVELLEMPLNSWNIRVIQWP

CVLLCNALLLSLSQARTLVEYSDRRLWRKISKYEYRRCAVIETYDSVKHLLLKIVREDSEERTVIRAGFEDIESAIKMEK
LTEKFKMSALPAVHSKVIALVEAVKQKPSSKELQKVVSALRSLYEIFIRDFTREKRDVEQLRRDGLVSPTGGTKLQFG
DAVDLPDADDEGFYRQLRRLHTVLTSRDSMNNVPKNLEARRRIAFFSNSLFMNMPHAPQVEKMRTFSVLTPYYDED
VMYSKEQLRTENEDGVSIIFYLQKIFNDEWENFRERMAQKGIVKESELWGTHIREPRLWASYRGQTLARTVRGMMY
YHNALKLLAFLDSASEMEVREGFRELVATVSGQRAEAIDLNSSSFRSARSVGSVGSGAGFSYQDHELATAMLKFTY
VVACQIYGTQRSKKDPRAEDILYLMKNNEALRVAYVDQVNVGRDNAGKDVWEYYSVLVKYDPDLRREVEIYRVKLP
GPLKLGEGKPENQNHAMIFTRGDAVQTIDMNQDNYFEEALKMRNLLQEFTKYYGIRRPTILGVREHVFTGSVSSLT
WFMSAQEMSFVTLGQRVLATPLKVRMHYGHPDVFDRLWFISRGGISKASGVINISEDIFAGFNCTLRGGNVTHHEYI
QVGKGRDVGLNEISMFEAKVASGNGEQVLSRDVYRLGHRLDFFRMLSFFYTTVGFYFNTMMIVLTVYAFLWGRVYL
ALSGVEGSINSSSTNNKALIAILNQQFIGQLGLLTALPMIIENALERGFLTAIRDFLTMQLQLASVFYTFSMGTRTHYFG
RTILRGGAKYRATGRGFVVEHKKFAENYRLYARSHFVKAIELGILLIVYASYNASSSSTVYILLTIISWFLVFSWIMAPFL
FNPSGFDRLKTVYDYDDFMNWIWYRGGIFTKSEHSWEAWWNEENDHLRSSGLWAIVVEILLDLRFFFFQYGIVYQL
GIANGSTSILVYLFSWIYVFAAFAIYMVVSSAHDKYAAKQHIYYRAIQSLVISVVILAMVILFQFAHLKITDLITSLLAFIPTG
WGLISIVQVFRPFLQHRTVVWHSVVSVARLYEIMFGMIIMMPMAILSWLPGFRSMQTRVLFNEAFSRGLQISRILAGK
KANPNI

>Gnemo9-Gnetum montanum 1741

MSTSQRSSAPPRIPVDASSSHQYETYNIIPLDNMLGDHPALRFPEVRAAVTALKTTGDLRAPPFVRWSPSYDLLDWL
GLFFGFQKDNVKNQREHLVLLLANSQMRLQPPPDIIDKLEPGIVRKLRKKILRNYETWCSFLGRKSNVWISQRRSSE
SDERRQLLYISLFLLIWGESANLRFMPECLCYIFHHMAMELNKILEGYVDESTGQHCLPSHRGENAFLIHVVTPLYET
VQAEADASGGGKAPHSAWRNYDDINEYFWSNRCFTHLGWPMRRDSNFFVQPKKQETTMQKLRGLMQRQKVGKT
GFVEQRTFWNLFHSFDRLWILLILFLQAAILVAWEDRGAPWNVLKRKDAQVKMLSLFITWAALRLWQALLDAGMQFN
LVSRETPLQGFRMLLKILTAAVWSILFTVLYVRTWNQRNHDFRWSPEADRRLKNCLWAAAIFILPECLALVLFIVPWIR
NFVEKSNFKIFHLLTWWFQSRSYVGRGLREGLVDNLYYSLFWVLLLAVKFAFSYQFQIKPMVEPTKEILKVKHIKYRW
HEFFKKTNHLAVGLIWAPVILIYFMDTQIWYSIMSSIIGALVGLFDHLGEIRNIQQLRLRFQMFASAIQFNLMPEEPLFR
QSDSLNVRIKDAWKRFRLRYGLGTAFKKMESNHAEAGRFALIWNEIIKTFREEDIVNNREVELLEMPLNSWNIRVIQW
PCVLVCNELLLSLSQARTLVEYSDRRLWRKISKYEYRRCAIIETYDSVKHLLLRIVREDSEERTVILAVFEDIESAIEMEK
LTEKFKMSALPAVHSKVIALVEAIKQKPSSKELQKVVNALQSLYEIFIRDFTREKRDVEQLRRDGLVSPSGGTKLLFGD
AVDLPDADDEGFYRQLRRLHTVLTSRDSMNNVPKNLEARRRIAFFSNSLFMNMPHAPQRHCIDIYCRASGAKINWN

KQQAIRSSFSPRPPWTYHFDINFSSTVRGMMYYHKALKLLAFLDSASEMEVREGFRELVATVSGQRAEAIDLNSSSF
RSARSVGSVGSGDPHAEDILYLMKNNEALRVAYVDQVNVGRDNAGVDVWEYYSVLVKYDPDLRREVEIYRVKLPG
PLKLGEGKPENQNHAMIFTRGDAVQTIDMNQDNYFEEALKMRNLLQEFTKYYGIRKPTILGVREHVFTGSVSSLAWF
MSAQEMSFVTLGQRVLATPLKVRMHYGHPDVFDRLWFISRGGISKASRVINISEDIFAGFNCTLRGGNVTHHEYIQV
GKGRDVGFNQISMFEAKVASGNGEQVLSRDVYRLGHRLDFFRMLSFFYTTVGFYFNTMMIVLTVYAFLWGRVYLAL
SGVEESINSSSTNNKALSAILNQQFIVQLGLFTALPMIIENSLERGFLTAIWDFLTMQLQLASVFYTFSMGTRTHYFGR
TILHGGAKYRATGRGFVVQHKKFAENYRLYARSHFVKAIELGILLIVYAAYNASSSSTVVYILLTISSWFLVFSWIMAPFL
FNPSGFDWLKTVYDYDDFMNWIWYRGGIFTKSEHSWEAWWNEENGHLRSSGLWAIVVEILLDLRFFFFQYGIVYQL
GIANGSTSILVYLLSWIYVFAAFAIYMVVSSAHDKYAAKQHIYYRAIQSLVISVFILAMVILFQFAHLKITDLITSLLAFIPTG
WGLISIVQVFKPFLQHRTVVWHSVVSVARLYEIMFGMIIMMPMAILSWLPGFQSMQTRILFNEAFSRGLQISRILAGKK
ANPNI

>Gnemo10-Gnetum montanum 1790
MGDQTVYIEEDEVFNILPVNNVLADHPAMQVTAVRAAIETLKFVEKLDMPPYAVLGEMVDILDWLGAYFGFQKDNVK
NQREHLVLLLANRQMQLQLPRNIEPDIMNQLGPTMVQTLRKKLLRNYTDWCSYLGQKSCMRVRKSILTRVENDLLHI
SLYLLIWGEAANLRFMPECLCYIFHQMGTELHKILERVINPDTGTVDIPVSMGIDGFLKHIVTPIYRTIKAEAEASRGGK
APHSAWRNYDDLNEFFWSRKCFQLLGWPFKLGSKFFVTAKRPQKIQGWDFQRFRRALQRQKVRKKGFVEQRSF
WNIFRSFDRLWIMHILFLQASIVVACQGSGNPFVELRYRDTQAQVLSIFITWAGIRFLQSLLDAGTQYSLASKQNPFIA
TRMVLKSCVALAWTVVFAVFYYLMWKQRNHDFRWSAEANRRLVIYLKICLVFILPELLALLLLILPWVRNFVEKRNWRI
FYVLTWWFQSRLFVGRGLRESLLYSILYSSFWVILLLSKFSFSYYFQIKPMISPSRTIAETEDISYQWHALFKHSNYISL
VLMWAPVTVIYFMDIQIWYSIYSALVGSLIGLLSHIGEIRDMRQFRLRFQFFASAIQFNLMSVESLFRINMSFRAKFRD
GVRRLQLRYGFGGYKKFESSESESGRFAIIWNEIVKTFREEDIVSDAEVELLEIPLPSGNVRVIHWPCILLCNELLLAL
NQAQALQGNDRTLWSKISKNEYRRCAVIEIYDSFKYLLQHIVMDGTQEHAIISKLFEEIDSSLKSGKFTEIYKTTELPGI
HSQFCSLVGALLIQPKERAQQRVIHILQNLYDIVVRDFPKERRSIEQLRKDRVASTDGTDKLLFEDSLYLPDEDDAEED
YLFFRRLRRLNTILTTRDPMLHIPRNLEARRRIAFFSNSLFMNMPRAVPVETMTAFSVLTPYYDEDVMYSREQLRTEN
EDGVSILFYLRNVFPDEWQNFMERMYRQGMKDSSELWTSKLLHLRLWASYRGQTLARTVRGMMYYDKALKLQAF
LDRASEVDLKDSLQQLALKVSSSLRSFDSQSQELFPPQQQQHHELLNREISSGIAIDNKEDAYALMKFTYVVTCQIYG
AQKARKDHRAKDILFLMKNHEALRIAYVDEVSTAGGETNFCSVLVKYDSKLQREVEIYRLRLPGPVKVGEGKPENQN
HAIIFTRGDALQAIDMNQENYFEETLKMRNLLQEFSTDYGLHKPSILGIREHVFTGSVSSLAWFMSAQEMCFVTIGQR

VLANPLKIRMHYGHPDVFDRLWFLGRGGMSKASKVINISEDIFAGYNCTLRGGNVTHHEYIQVGKGRDVGLNQISMF

EAKVASGNGEQVLSRDVYRLGHRLDFFRMLSLYYSTVGFFFSTMLVVVAVFVFLWGRVYMALSGVEKSLKHKSDVT

NNRALASVLNQQFIVQLGLLTALPLIIEMALERGFLGAVWDFLTMQLQLAAVFYAFSMGTRTHFFGRTVLHGGAKYRA

TGRGFVVQHKSFAENYRLYARSHFVKAIELGVLLIVYASYTAVNTKLFVYILMSLSSWFLVFSWAMAPFIFNPLGFDWL

KAVDDFGDFITWIWNKNTIYAKADNSWEVWWYEEQEHLQSTGIWGKALEIIVDFRFFILQYGIVYQLGIANNSKSILVY

LISWVYVVAALAIYLIMTYAEEKYAANKHIYYRGVQFMVMLFVLIVMTVLLTSTDFKILDIVKSVLAVVPTGWGLISIALVL

RPLLEPTIVWQMVLVLARFYDMIFGLIIMAPVALLSWIPGFQLLQTRILFNEAFSRGLRISQILVVKKRNVD

>CS1-A.thaliana 1950

MAQRREPDPPPPQRRILRTQTVGSLGEAMLDSEVVPSSLVEIAPILRVANEVEASNPRVAYLCRFYAFEK

AHRLDPTSSGRGVRQFKTALLQRLERENETTLAGRQKSDAREMQSFYQHYYKKYIQALLNAADKADRAQL

TKAYQTAAVLFEVLKAVNQTEDVEVADEILETHNKVEEKTQIYVPYNILPLDPDSQNQAIMRLPEIQAAV

AALRNTRGLPWTAGHKKKLDEDILDWLQSMFGFQKDNVLNQREHLILLLANVHIRQFPKPDQQPKLDDRA

LTIVMKKLFRNYKKWCKYLGRKSSLWLPTIQQEVQQRKLLYMGLYLLIWGEAANLRFMPECLCYIYHHMA

FELYGMLAGSVSPMTGEHVKPAYGGEDEAFLQKVVTPIYQTISKEAKRSRGGKSKHSVWRNYDDLNEYFW

SIRCFRLGWPMRADADFFCQTAEELRLERSEIKSNSGDRWMGKVNFVEIRSFWHIFRSFDRLWSFYILCL

QAMIVIAWNGSGELSAIFQGDVFLKVLSVFITAAILKLAQAVLDIALSWKARHSMSLYVKLRYVMKVGAA

AVWVVVMAVTYAYSWKNASGFSQTIKNWFGGHSHNSPSLFIVAILIYLSPNMLSALLFLFPFIRRYLERS

DYKIMMLMMWWSQPRLYIGRGMHESALSLFKYTMFWIVLLISKLAFSYYAEIKPLVGPTKDIMRIHISVY

SWHEFFPHAKNNLGVVIALWSPVILVYFMDTQIWYAIVSTLVGGLNGAFRRLGEIRTLGMLRSRFQSIPG

AFNDCLVPQDNSDDTKKKRFRATFSRKFDQLPSSKDKEAARFAQMWNKIISSFREEDLISDREMELLLVP

YWSDPDLDLIRWPPFLLASKIPIALDMAKDSNGKDRELKKRLAVDSYMTCAVRECYASFKNLINYLVVGE

REGQVINDIFSKIDEHIEKETLITELNLSALPDLYGQFVRLIEYLLENREEDKDQIVIVLLNMLELVTRD

IMEEEVPSLLETAHNGSYVKYDVMTPLHQQRKYFSQLRFPVYSQTEAWKEKIKRLHLLLTVKESAMDVPS

NLEARRRLTFFSNSLFMDMPPAPKIRNMLSFSVLTPYFSEDVLFSIFGLEQQNEDGVSILFYLQKIFPDE

WTNFLERVKCGNEEELRAREDLEEELRLWASYRGQTLTKTVRGMMYYRKALELQAFLDMAKDEELLKGYK

ALELTSEEASKSGGSLWAQCQALADMKFTFVVSCQQYSIHKRSGDQRAKDILRLMTTYPSIRVAYIDEVE

QTHKESYKGTEEKIYYSALVKAAPQTKPMDSSESVQTLDQLIYRIKLPGPAILGEGKPENQNHAIIFTRG

EGLQTIDMNQDNYMEEAFKMRNLLQEFLEKHGGVRCPTILGLREHIFTGSVSSLAWFMSNQENSFVTIGQ

RVLASPLKVRFHYGHPDIFDRLFHLTRGGICKASKVINLSEDIFAGFNSTLREGNVTHHEYIQVGKGRDV

GLNQISMFEAKIANGNGEQTLSRDLYRLGHRFDFFRMLSCYFTTIGFYFSTMLTVLTVYVFLYGRLYLVL

SGLEEGLSSQRAFRNNKPLEAALASQSFVQIGFLMALPMMMEIGLERGFHNALIEFVLMQLQLASVFFTF

QLGTKTHYYGRTLFHGGAEYRGTGRGFVVFHAKFAENYRFYSRSHFVKGIELMILLLVYQIFGQSYRGVV

TYILITVSIWFMVVTWLFAPFLFNPSGFEWQKIVDDWTDWNKWIYNRGGIGVPPEKSWESWWEKELEHLR

HSGVRGITLEIFLALRFFIFQYGLVYHLSTFKGKNQSFWVYGASWFVILFILLIVKGLGVGRRRFSTNFQ

LLFRIIKGLVFLTFVAILITFLALPLITIKDLFICMLAFMPTGWGMLLIAQACKPLIQQLGIWSSVRTLA

RGYEIVMGLLLFTPVAFLAWFPFVSEFQTRMLFNQAFSRGLQISRILGGQRKDRSSKNKE

>CS2-A.thaliana 1950

MAQRKGPDPPPPQRRILRTQTAGNLGEAMLDSEVVPSSLVEIAPILRVANEVEASNPRVAYLCRFYAFEK

AHRLDPTSSGRGVRQFKTALLQRLERENETTLAGRQKSDAREMQSFYQHYYKKYIQALQNAADKADRAQL

TKAYQTAAVLFEVLKAVNQTEDVEVADEILEAHTKVEEKSQIYVPYNILPLDPDSQNQAIMRFPEIQATV

SALRNTRGLPWPAGHKKKLDEDMLDWLQTMFGFQKDNVSNQREHLILLLANVHIRQFPRPEQQPRLDDRA

LTIVMKKLFKNYKKWCKYLGRKSSLWLPTIQQEVQQRKLLYMGLYLLIWGEAANLRFLPECLCYIYHHMA

FELYGMLAGSVSPMTGEHVKPAYGGEDEAFLQKVVTPIYKTIAKEAKRSRGGKSKHSEWRNYDDLNEYFW

SIRCFRLGWPMRADADFFCQTAEELRLDRSENKPKTGDRWMGKVNFVEIRSFWHIFRSFDRMWSFYILSL

QAMIIIAWNGSGKLSGIFQGDVFLKVLSIFITAAILKLAQAVLDIALSWKSRHSMSFHVKLRFIFKAVAA

AIWVVLMPLTYAYSWKTPSGFAETIKNWFGGHQNSSPSFFIIVILIYLSPNMLSTLLFAFPFIRRYLERS

DYKIVMLMMWWSQPRLYIGRGMHESALSLFKYTMFWVVLLISKLAFSFYAEIKPLVKPTKDIMRVHISVY

RWHEFFPHAKSNMGVVIALWSPVILVYFMDTQIWYAIVSTLVGGLNGAFRRLGEIRTLGMLRSRFQSLPE

AFNACLVPNEKSETPKKKGIMATFTRKFDQVPSSKDKEAARFAQMWNKIISSFREEDLISDREMELLLVP

YWADRDLDLIRWPPFLLASKIPIALDMAKDSNGKDRELTKRLSVDSYMTCAVRECYASFKNLINFLVVGE

REGQVINEIFSRIDEHIEKETLIKDLNLSALPDLYGQFVRLIEYLMENREEDKDQIVIVLLNMLEVVTRD

IMDEEVPSMLESTHNGTYVKYDVMTPLHQQRKYFSQLRFPVYSQTEAWKEKIKRLHLLLTVKESAMDVPS

NLEARRRLTFFSNSLFMEMPDAPKIRNMLSFSVLTPYYSEDVLFSIFGLEKQNEDGVSILFYLQKIFPDE

WTNFLERVKCGSEEELRAREELEEELRLWASYRGQTLTKTVRGMMYYRKALELQAFLDMAKDEELMKGYK

ALELTSEDASKSGTSLWAQCQALADMKFTFVVSCQQYSVQKRSGDQRAKDILRLMTTYPSLRVAYIDEVE

QTHKESYKGADEKIYYSALVKAAPQTKSMDSSESVQTLDQVIYRIKLPGPAILGEGKPENQNHSIIFTRG

EGLQTIDMNQDNYMEEAFKMRNLLQEFLVKHGGVRTPTILGLREHIFTGSVSSLAWFMSNQENSFVTIGQ
RVLASPLKVRFHYGHPDVFDRLFHLTRGGVCKASKVINLSEDIFAGFNSTLREGNVTHHEYIQVGKGRDV
GLNQISMFEAKIANGNGEQTLSRDLYRLGHRFDFFRMLSCYFTTIGFYFSTMLTVLTVYVFLYGRLYLVL
SGLEEGLSNQKAFRSNMPLQAALASQSFVQIGFLMALPMMMEIGLERGFHNALIDFVLMQLQLASVFFTF
QLGTKTHYYGRTLFHGGAEYRGTGRGFVVFHAKFAENYRFYSRSHFVKGIELMILLLVYQIFGHAYRGVV
TYILITVSIWFMVVTWLFAPFLFNPSGFEWQKIVDDWTDWNKWIYNRGGIGVPPEKSWESWWEKEIGHLR
HSGKRGIILEIVLALRFFIFQYGLVYQLSTFKQENQSLWIYGASWFVILFILLIVKGLGVGRQRFSTNFQ
LLFRIIKGFVFLTFLGLLITFLALRFLTPKDIFLCMLAFMPTGWGMLLIAQACKPLIQRLGFWSSVRTLA
RGYEILMGLLLFTPVAFLAWFPFVSEFQTRMLFNQAFSRGLQISRILGGQRKDRSSKNKE

>CS3-A.thaliana 1955
MSATRGGPDQGPSQPQQRRIIRTQTAGNLGESFDSEVVPSSLVEIAPILRVANEVESSNPRVAYLCRFYA
FEKAHRLDPTSSGRGVRQFKTALLQRLEREHDPTLMGRVKKSDAREMQSFYQHYYKKYIQALHNAADKAD
RAQLTKAYQTANVLFEVLKAVNLTQSIEVDREILEAQDKVAEKTQLYVPYNILPLDPDSANQAIMRYPEI
QAAVLALRNTRGLPWPEGHKKKKDEDMLDWLQEMFGFQKDNVANQREHLILLLANVHIRQFPKPDQQPKL
DDQALTEVMKKLFKNYKKWCKYLGRKSSLWLPTIQQEMQQRKLLYMALYLLIWGEAANLRFMPECLCYIY
HHMAFELYGMLAGNVSPMTGENVKPAYGGEEDAFLRKVVTPIYEVIQMEAQRSKKGKSKHSQWRNYDDLN
EYFWSVDCFRLGWPMRADADFFCLPVAVPNTEKDGDNSKPIVARDRWVGKVNFVEIRSFWHVFRSFDRMW
SFYILCLQAMIIMAWDGGQPSSVFGADVFKKVLSVFITAAIMKLGQAVLDVILNFKAHQSMTLHVKLRYI
LKVFSAAAWVIILPVTYAYSWKDPPAFARTIKSWFGSAMHSPSLFIIAVVSYLSPNMLAGVMFLFPLLRR
FLERSNYRIVMLMMWWSQPRLYVGRGMHESAFSLFKYTMFWVLLIATKLAFSYYIEIRPLVAPTQAIMKA
RVTNFQWHEFFPRAKNNIGVVIALWAPIILVYFMDSQIWYAIFSTLFGGIYGAFRRLGEIRTLGMLRSRF
ESLPGAFNDRLIPDGKNQQKKKGIRATLSHNFTEDKVPVNKEKEAARFAQLWNTIISSFREEDLISDREM
DLLLVPYWADRDLDLIQWPPFLLASKIPIALDMAKDSNGKDRELKKRIESDTYMKCAVRECYASFKNIIK
FVVQGNREKEVIEIIFAEVDKHIDTGDLIQEYKMSALPSLYDHFVKLIKYLLDNKEEDRDHVVILFQDML
EVVTRDIMMEDYNISSLVDSSHGGTWHGGMIPLEQQYQLFASSGAIRFPIEPVTEAWKEKIKRIYLLLTT
KESAMDVPSNLEARRRISFFSNSLFMDMPMAPKVRNMLSFSVLTPYYTEEVLFSLRDLETPNEDGVSILF
YLQKIFPDEWNNFLERVKCLSEEELKESDELEEELRLWASYRGQTLTRTVRGMMYYRKALELQAFLDMAM
HEDLMEGYKAVELNSENNSRGERSLWAQCQAVADMKFTYVVSCQQYGIHKRSGDPRAQDILRLMTRYPSL

RVAYIDEVEEPVKDKSKKGNQKVYYSVLVKVPKSTDHSTLAQNLDQVIYRIRLPGPAILGEGKPENQNHA

IIFSRGEGLQTIDMNQDNYMEEALKMRNLLQEFLTKHDGVRHPSILGLREHIFTGSVSSLAWFMSNQETS

FVTIGQRLLANPLRVRFHYGHPDVFDRLFHLTRGGVSKASKVINLSEDIFAGFNSTLREGNVTHHEYIQV

GKGRDVGLNQISMFEAKIANGNGEQTLSRDIYRLGHRFDFFRMMSCYFTTVGFYFSTLITVLTVYIFLYG

RLYLVLSGLEQGLSTQKGIRDNTPLQIALASQSFVQIGFLMALPMLMEIGLERGFRTALSEFVLMQLQLA

PVFFTFSLGTKTHYYGRTLLHGGAKYRSTGRGFVVFHAKFADNYRLYSRSHFVKGLEMMLLLVVYQIFGS

AYRGVLAYLLITISMWFMVGTWLFAPFLFNPSGFEWQKIVDDWTDWNKWINNIGGIGVPAEKSWESWWEE

EQEHLRYSGKRGIVVEILLALRFFIYQYGLVYHLTITEKTKNFLVYGVSWLVIFLILFVMKTVSVGRRRF

SASFQLMFRLIKGLIFMTFIAIIVILITLAHMTIQDIIVCILAFMPTGWGMLLIAQACKPVVHRAGFWGS

VRTLARGYEIVMGLLLFTPVAFLAWFPFVSEFQTRMLFNQAFSRGLQISRILGGHRKDRSSRNKE

>CS4-A.thaliana 1871

MNQPNRGQILQTVFSHFFPVASPDSELVPSSLHEDITPILRVAKDVEDTNPRSLFLQDLDIKSVDDSINI

LSGHSHALDKANELDPTSSGRDVRQFKNTILQWLEKNNESTLKARQKSSDAHEMQSFYQQYGDEGINDLL

NAGAGSSSSQRTKIYQTAVVLYDVLDAVHRKANIKVAAKILESHAEVEAKNKIYVPYNILPLDPDSKNHA

MMRDPKIVAVLKAIRYTSDLTWQIGHKINDDEDVLDWLKTMFRFQKDNVSNQREHLILLLANVQMRQTQR

QPNLLDDRALDTVMEKLLGNYNKWCNHVGLESSLRFPKDKQQKVVQQRKLLYTGLYLLIWGEAANLRFMP

ECLCYIYHHMAFELFEMLESKGSKKKYKPKNPTYSGKDEDFLTKVVTPVYKTIAEEAKKSGEGKHSEWRN

YDDLNEYFWSKQYLDKLGWPMKANADFFCKTSQQLGLNKSEKKPDLGDGCVGKVNFVEIRTFWHLFRSFD

RMWSFYILSLQAMIIIAWNETSESGGAVFHKVLSVFITAAKLNLFQAFLDIALSWKARHSMSTHVRQRYI

FKAVAAAVWVLLMPLTYAYSHTSIFIVAILIYLSPNMLPEMLLLIPSIRRTLEKSDFRPVKLIMWWSQPE

LYIGRGMHESAWSIYKYMMFWIVLLTSKLAFSYYVEQIKPLMGPTKEIMSVPMPGYWLPEFFPHVKNNRG

VVITLWSPVILVYFMDTQIWYAIVSTLVGGLYGAFRHIGEIQTLGMLRSRFQSLPGAFNACLIPNENTKE

KGIKLAFSRKCHKIPNTNGKEAKQFSQMWNTIINSFREEDLISNRELELLLMSCWAYPDLDFIRWPIFLL

ASKIPIAVDIAKKRNGKHRELKNILAEDNCMSCAVRECYASIKKLLNTLVTGNSDLMLITTVFTIIDTHI

EKDTLLTELNLSVLPDLHGHFVKLTEYVLQNKDKDKIQIVNVLLKILEMVTKDILKEEIKRLHLLLTVKE

SAMDVPSNLEARRRLTFFSNSLFMEMPGAPKIQNMLSFSALTPYYSEDVLFSTFDLEKENDGVSILFYLQ

KIFPDEWKNFLERVKCGTEEELDAIDYLKEEIRLWASYRGQTLTKTVRGMMYYQKALELQAFFDLANERE

LMKGYKSAEASSSGSSLWAECQALADIKFTYVVACQQYSIHKRSGDQRAKDILTLMTTYPSLRVAYIDEV

EQTHIYSKGTSENFYYSALVKAAPQTYSTDSSDSGHMLDQVIYQIKLPGPPIIGEGKPENQNNAIIFTRG
EALQTIDMNQDYYIEEAFKMRNLLQEFLEKNGGVRYPTILGLREHIFTRSVSCLAWFMSNQEHSFVTIGQ
RVLANPLKVRFHYGHPDVFDRVFHLTRGGVSKASKVINLSEDIFAGFNSTLREGTVSHHEYIQVGKGRDV
GLNQISMFEAKIANGSGEQTLSRDLYRLGHQFDFFRMLSCYFTTVGFYFCSMLTVLTVYVFLYGRLYLVL
SGVEKELGNKPMMMEIILASQSFVQIVFLMAMPMIMEIGLERGFYDALFDFVLMQLQLASVFFTFQLGTK
FHYYCKTLLHGGAEYRGTGRGFVVFHAKFAENYRFYSRSHFVKATELGILLLVYHIFGPTYIGLFTISIW
FMVGTWLFAPFLFNPSGFEWHEIVEDWADWKKWIEYDNGGIGVPPEKSWESWWEKDIEHLQHSGKWGIVV
EIFFALRFFIFQYGLVYQLSAFKNKYSSLWVFGASWLLILILLLTVTVLDYARRRLGTEFQLLFRIIKVS
LFLAFMAIFITLMTCRLILPQDVFLCMLALIPTGWGLLLIAQSCKPLIQQPGIWSWVMTLAWVYDLVMGS
LLFIPIAFMAWFPFISEFQTRMLFNQAFSRGLHISRILSGQRKHRSSKNKD

>CS5-A.thaliana 1923
MAQSSTSHDSGPQGLMRRPSRSAATTVSIEVFDHEVVPASLGTIAPILRVAAEIEHERPRVAYLCRFYAF
EKAHRLDPSSGGRGVRQFKTLLFQRLERDNASSLASRVKKTDGREVESFYQQYYEHYVRALDQGDQADRA
QLGKAYQTAGVLFEVLMAVNKSEKVEAVAPEIIAAARDVQEKNEIYAPYNILPLDSAGASQSVMQLEEVK
AAVAALGNTRGLNWPSGFEQHRKKTGNLDLLDWLRAMFGFQRDNVRNQREHLVCLFADNHIRLTPKPEPL
NKLDDRAVDTVMSKLFKNYKNWCKFLGRKHSLRLPQAAQDIQQRKILYMGLYLLIWGEAANIRFMPECLC
YIFHNMAYELHGLLAGNVSIVTGENIKPSYGGDDEAFLRKVITPIYRVVQTEANKNANGKAAHSDWSNYD
DLNEYFWTPDCFSLGWPMRDDGDLFKSTRDTTQGKKGSFRKAGRTGKSNFTETRTFWHIYHSFDRLWTFY
LLALQAMIILAFERVELREILRKDVLYALSSIFITAAFLRFLQSVLDVILNFPGFHRWKFTDVLRNILKI
VVSLAWCVVLPLCYAQSVSFAPGKLKQWLSFLPQVKGVPPLYIMAVALYLLPNVLAAIMFIFPMLRRWIE
NSDWHIFRLLLWWSQPRIYVGRGMHESQIALIKYTIFWLLLFCCKFAFSYFLQVKLLVKPTNAIMSIRHV
KYKWHEFFPNAEHNYGAVVSLWLPVILVYFMDTQIWYAIFSTICGGVIGAFDRLGEIRTLGMLRSRFQSL
PGAFNTYLVPSDKTRRRGFSLSKRFAEVTAARRTEAAKFSQLWNEIISSFREEDLISDREMDLLLVPYTS
DPSLKLIQWPPFLLASKIPIALDMAAQFRTRDSDLWKRICADEYMKCAVIECYESFKHVLHTLVIGENEK
RIIGIIIKEVESNISKNSFLSNFRMAPLPALCSKFVELVGILKNADPAKRDTVVLLLQDMLEVVTRDMMQ
NENRELVELGHTNKESGRQLFAGTDAKPAILFPPVATAQWHEQISRLHLLLTVKESAMDVPTNLEAQRRI
AFFTNSLFMDMPRAPRVRNMLSFSVLTPYYSEETVYSKNDLEMENEDGVSVVYYLQKIFPDEWTNFLERL
DCKDETSVLESEENILQLRHWVSLRGQTLFRTVRGMMYYRRALKLQAFLDMANETEILAGYKAISEPTEE

DKKSQRSLYTQLEAVADLKFTYVATCQNYGNQKRSGDRRATDILNLMVNNPSLRVAYIDEVEEREGGKVQ

KVFYSVLIKAVDNLDQEIYRIKLPGPAKIGEGKPENQNHALIFTRGEALQAIDMNQDHYLEEALKMRNLL

EEFNEDHGVRAPTILGFREHIFTGSVSSLAWFMSNQETSFVTIGQRVLASPLKVRFHYGHPDVFDRIFHI

TRGGISKASRGINLSEDIFAGFNSTLRRGNVTHHEYIQVGKGRDVGLNQISLFEAKVACGNGEQTLSRDL

YRLGHRFDFFRMMSCYFTTVGFYISSMIVVLTVYAFLYGRLYLSLSGVEEAIVKFAAAKGDSSLKAAMAS

QSVVQLGLLMTLPMVMEIGLERGFRTALSDLIIMQLQLAPVFFTFSLGTKVHYYGRTILHGGSKYRATGR

GFVVKHEKFAENYRMYSRSHFVKGMELMVLLICYRIYGKAAEDSVGYALVMGSTWFLVGSWLFAPFFFNP

SGFEWQKIVDDWDDWNKWISSRGGIGVPANKSWESWWEEEQEHLLHSGFFGKFWEIFLSLRYFIYQYGIV

YQLNLTKESRMGKQHSIIVYGLSWLVIVAVMIVLKIVSMGRKKFSADFQLMFRLLKLFLFIGSVVIVGML

FHFLKLTVGDIMQSLLAFLPTGWALLQISQVARPLMKTVGMWGSVKALARGYEYIMGVVIFMPVTVLAWF

PFVSEFQTRLLFNQAFSRGLQIQRILAGGKKQK

>CS6-A.thaliana 1921

MEASSSGTAELPRSLSRRAPSRATTMMIDRPNEDASAMDSELVPSSLASIAPILRVANEIEKDNPRVAYL

CRFHAFEKAHRMDATSSGRGVRQFKTYLLHRLEKEEEETKPQLAKNDPREIQAYYQNFYEKYIKEGETSR

KPEEMARLYQIASVLYDVLKTVVPSPKVDYETRRYAEEVERKRDRYEHYNILPLYAVGTKPAIVELPEVK

AAFSAVRNVRNLPRRRIHLPSNTPNEMRKARTKLNDILEWLASEFGFQRGNVANQREHIILLLANADIRK

RNDEEYDELKPSTVTELMDKTFKSYYSWCKYLHSTSNLKFPDDCDKQQLQLIYISLYLLIWGEASNVRFM

PECICYIFHNMANDVYGILFSNVEAVSGETYETEEVIDEESFLRTVITPIYQVIRNEAKRNKGGTASHSQ

WRNYDDLNEYFWSKKCFKIGWPLDLKADFFLNSDEITPQDERLNQVTYGKSKPKTNFVEVRTFWNLFRDF

DRMWIFLVMAFQAMVIVGWHGSGSLGDIFDKDVFKTVLTIFITSAYLTLLQAALDIILNFNAWKNFKFSQ

ILRYLLKFAVAFMWAVLLPIAYSKSVQRPTGVVKFFSTWTGDWKDQSFYTYAVSFYVLPNILAALLFLVP

PFRRAMECSDMRPIKVIMWWAQPKLYVGRGMHEDMFSLFKYTTFWIMLLISKLAFNYYVEILPLITPTKM

IMNLHIGHYQWHEFFPHATNNIGVVIAIWAPIVLVYLMDTQIWYAIFSTLFGGIHGAFSHLGEIRTLGML

RSRFESIPIAFSRTLMPSEDAKRKHADDYVDQKNITNFSQVWNEFIYSMRSEDKISDRDRDLLLVPSSSG

DVSVIQWPPFLLASKIPIAVDMAKDFKGKEDAELFRKIKSDSYMYYAVIESYETLKKIIYALLEDEADRR

VMNQVFLEVDMSMQQQRFIYEFRMSGLPLLSDKLEKFLSILLSDYEDQGTYKSQLINVFQDVIEIITQDL

LVNGHEILERARVHSPDIKNEKKEQRFEKINIHLVRDRCWREKVIRLHLLLSVKESAINVPQNLEARRRI

TFFANSLFMNMPSAPRIRDMLSFSVLTPYYKEDVLYSEEDLNKENEDGISILFYLQKIYPDEWTNYLDRL

KDPKLPEKDKSEFLREWVSYRGQTLARTVRGMMYYRQALELQCYQEVAGEQAEFSVFRAMASNDENQKAF
LERARALADLKFTYVVSCQVYGNQKKSGDIHNRSCYTNILQLMLKYPSLRVAYVDEREETADAKSPKVFY
SVLLKGGDKFDEEIYRIKLPGPPAEIGEGKPENQNHAIIFTRGEALQTIDMNQDNYFEEAFKLRNVLEEF
NKERVGRRKPTILGLREHIFTGSVSSLAWFMSNQESSFVTIGQRILANPLRVRFHYGHPDIFDRIFHITR
GGVSKASKVINLSEDIFGGFNSTLRGGYVTHHEYIQVGKGRDVGLNPISIFEAKVANGNGEQTLSRDVYR
LGHRFDFYRMLSFYFTTIGFYFSSMLTVLTVYAFLYGRMYMVMSGLEKEILRLASPNQLEALEQALATQS
IFQLGFLMVLPMVMEIGLEHGFRSAIVDFFIMQLQLASVFFTFQLGTKSHYYGRTILHGGSKYRPTGRGF
VVFHAKFAENYRLYSRSHFVKGLELLLLLVVYQIYGHSYRSSNLYLYITVSMWFMVGSWLFAPFIFNPSG
FEWQKTVDDWTDWKRWLGDRGGIGIPVEKSWESWWNVEQEHLKHTSIRGRILEITLALRFFIYQYGIVYQ
LNISQRSKSFLVYGLSWVVLLTSLLVLKMVSMGRRRFGTDFQLMFRILKALLFLGFLSVMTILFVVFKLT
LTDLSASVLAFLPTGWAILLIGQVLRSPIKALGVWDSVKELGRAYENIMGLVIFAPIAVLSWFPIVSEFQ
ARLLFNQAFSRGLQISMILAGRKDKATSSHK

>CS7-A.thaliana 1958
MASTSSGGRGEDGRPPQMQPVRSMSRKMTRAGTMMIEHPNEDERPIDSELVPSSLASIAPILRVANDIDQ
DNARVAYLCRFHAFEKAHRMDPTSSGRGVRQFKTYLLHKLEEEEEITEHMLAKSDPREIQLYYQTFYENN
IQDGEGKKTPEEMAKLYQIATVLYDVLKTVVPQARIDDKTLRYAKEVERKKEQYEHYNILPLYALGAKTA
VMELPEIKAAILAVCNVDNLPRPRFHSASANLDEVDRERGRSFNDILEWLALVFGFQRGNVANQREHLIL
LLANIDVRKRDLENYVEIKPSTVRKLMEKYFKNYNSWCKYLRCDSYLRFPAGCDKQQLSLLYIGLYLLIW
GEASNVRFMPECLCYIFHNMANEVHGILFGNVYPVTGDTYEAGAPDEEAFLRNVITPIYQVLRKEVRRNK
NGKASHSKWRNYDDLNEYFWDKRCFRLKWPMNFKADFFIHTDEISQVPNQRHDQVSHGKRKPKTNFVEAR
TFWNLYRSFDRMWMFLVLSLQTMIIVAWHPSGSILAIFTEDVFRNVLTIFITSAFLNLLQATLDLVLSFG
AWKSLKFSQIMRYITKFLMAAMWAIMLPITYSKSVQNPTGLIKFFSSWVGSWLHRSLYDYAIALYVLPNI
LAAVFFLLPPLRRIMERSNMRIVTLIMWWAQPKLYIGRGMHEEMFALFKYTFFWVMLLLSKLAFSYYVEI
LPLVNPTKLIWDMHVVNYEWHEFFPNATHNIGVIIAIWGPIVLVYFMDTQIWYAIFSTLFGGIYGAFSHL
GEIRTLGMLRSRFKVVPSAFCSKLTPLPLGHAKRKHLDETVDEKDIARFSQMWNKFIHTMRDEDLISDRE
RDLLLVPSSSGDVTVVQWPPFLLASKIPIALDMAKDFKGKEDVDLFKKIKSEYYMHYAVVEAYETVRDII
YGLLQDESDKRIVREICYEVDISIQQHRFLSEFRMTGMPLLSDKLEKFLKILLSDYEEDDYKSQIINVLQ
DIIEIITQDVMVNGHEILERAHLQSGDIESDKKEQRFEKIDLSLTQNISWREKVVRLLLLLTVKESAINI

PQSLEARRRMTFFANSLFMNMPDAPRVRDMLSFSVLTPYYKEDVLYSEEELNKENEDGITILFYLQRIYP

EEWSNYCERVNDLKRNLSEKDKAEQLRQWVSYRGQTLSRTVRGMMYYRVALELQCFQEYTEENATNGGYL

PSESNEDDRKAFSDRARALADLKFTYVVSCQVYGNQKKSSESRDRSCYNNILQLMLKYPSLRVAYIDERE

ETVNGKSQKVFYSVLLKGCDKLDEEIYRIKLPGPPTEIGEGKPENQNHAIIFTRGEALQTIDMNQDNYFE

ECFKMRNVLQEFDEGRRGKRNPTILGLREHIFTGSVSSLAWFMSNQETSFVTIGQRVLANPLRVRFHYGH

PDIFDRIFHITRGGISKASKIINLSEDIFAGYNSTLRGGYVTHHEYIQAGKGRDVGMNQISFFEAKVANG

NGEQTLSRDVYRLGRRFDFYRMLSFYFTTVGFYFSSMITVLTVYVFLYGRLYLVLSGLEKNILQSASVHE

SNALEQALAAQSVFQLGFLMVLPMVMEIGLEKGFRTALGDFIIMQLQLASVFFTFQLGTKAHYFGRTILH

GGSKYRATGRGFVVFHAKFAENYRLYSRSHFVKGLELVILLVVYQVYGTSYRSSSTYMYITFSMWFLVTS

WLFAPFIFNPSGFEWQKTVDDWTDWKRWMGNRGGIGIVLDKSWESWWDIEQEHLKHTNLRGRVLEILLAL

RFLLYQYGIVYHLNIARRHTTFLVYGLSWAILLSVLLVLKMVSMGRRKFGTDFQVMFRILKALLFLGFLS

VMTVLFVVCGLTISDLFASILAFLPTGWAILLIGQALRSVFKGLGFWDSVKELGRAYEYIMGLVIFTPIA

VLSWFPFVSEFQTRLLFNQAFSRGLQISMILAGKKDKETPSTKYLGHTEESFGLEHDTNTFNHYYLWT

>CS8-A.thaliana 1976

MSHEIVPVDPIDVPSTSYSRPILGPREDSPERATEFTRSLTFREHVSSEPFDSERLPATLASEIQRFLRI

ANLVESEEPRIAYLCRFHAFEIAHHMDRNSTGRGVRQFKTSLLQRLELDEEFTVRRRKEKSDVRELKRVY

HAYKEYIIRHGAAFNLDNSQREKLINARRIASVLYEVLKTVTSGAGPQAIADRESIRAKSEFYVPYNILP

LDKGGVHQAIMHLPEIKAAVAIVRNTRGLPPPEEFQRHQPFLDLFEFLQYAFGFQNGNVANQREHLILLL

SNTIIRQPQKQSSAPKSGDEAVDALMKKFFKNYTNWCKFLGRKNNIRLPYVKQEALQYKTLYIGLYLLIW

GEASNLRFMPECLCYIFHHMAYELHGVLTGAVSMITGEKVAPAYGGGHESFLADVVTPIYMVVQKEAEKN

KNGTADHSMWRNYDDLNEFFWSLECFEIGWPMRPEHDFFCVESSETSKPGRWRGMLRFRKQTKKTDEEIE

DDEELGVLSEEQPKPTSRWLGKTNFVETRSFWQIFRSFDRMWSFFVLSLQALIIMACHDVGSPLQVFNAN

IFEDVMSIFITSAILKLIKGILDIIFKWKARNTMPINEKKKRLVKLGFAAMWTIILPVLYSHSRRKYICY

FTNYKTWLGEWCFSPYMVAVTIYLTGSAIELVLFFVPAISKYIETSNHGIFKTLSWWGQPRLYVGRGMQE

TQVSQFKYTFFWILVLLTKFAFSYAFEIKPLIEPTRLIMKVGVRNYEWHEIFPEVKSNAAAIVAVWAPIM

VVYFMDTQIWYSVYCTIFGGLYGVLHHLGEIRTLGMLRGRFHTLPSAFNASLIPHSTKDEKRRKQRGFFP

FNLGRGSDGQKNSMAKFVLVWNQVINSFRTEDLISNKELDLMTMPLSSEVLSGIIRWPIFLLANKFSTAL

SIAKDFVGKDEVLYRRIRKDEYMYYAVKECYESLKYILQILVVGDLEKKIISGIINEIEESIRQSSLLEE

FKMAELPALHDKCIELVQLLVEGSAEQLQVEKSEELHGKLVKALQDIFELVTNDMMVHGDRILDLLQSRE

GSGEDTGIFMRVIEPQLFESYGEWRCIHFPLPDSASLSEQIQRFLLLLTVKDSAMDIPENLDARRRLSFF

ATSLFMDMPDAPKVRNMMSFSVLTPHYQEDINYSTNELHSTKSSVSIIFYMQKIFPDEWKNFLERMGCDN

LDALKKEGKEEELRNWASFRGQTLSRTVRGMMYCREALKLQAFLDMADDEDILEGYKDVERSNRPLAAQL

DALADMKFTYVVSCQMFGAQKSSGDPHAQDILDLMIKYPSLRVAYVEEREEIVLDVPKKVYYSILVKAVN

GFDQEIYRVKLPGPPNIGEGKPENQNHAIVFTRGEALQTIDMNQDHYLEEAFKMRNLLQEFLRNRGRRPP

TILGLREHIFTGSVSSLAWFMSYQETSFVTIGQRLLANPLRVRFHYGHPDVFDRIFHITRGGISKSSRTI

NLSEDVFAGYNTTLRRGCITYNEYLQVGKGRDVGLNQISKFEAKVANGNSEQTISRDIYRLGQRFDFFRM

LSCYFTTIGFYFSSLISVIGIYIYLYGQLYLVLSGLQKTLILEAKVKNIKSLETALASQSFIQLGLLTGL

PMVMEIGLEKGFLIAFQDFILMQLQLAAFFFTFSLGTKTHYFGRTILHGGAKYRPTGRKVVVFHANFSEN

YRLYSRSHFIKGFELMILLVVYELFKHTSQSNMAYSFITFSVWFMSFTWLCAPFLFNPSGFTWEIIVGDW

RDWNRWIKEQGGIGIQQDKSWQSWWNDEQAHLRGSGVGARCLEIILSLRFFVYQYGLVYHLDITQSNTNI

IVYALSWVVILATFFTVKAVDLGRQLFSTRKHLVFRFFKVFVFVSILTIIITLANICHLSVKDLLVSCLA

FLPTGWGLILIAQAVRPKIEGTSLWEFTQVLARAYDYGMGVVLFAPMAILAWLPIISAFQTRFLFNEAFN

RRLQIQPILAGKKKNR

>CS9-A.thaliana 1890

MSRAESSWERLVNAALRRDRTGGVAGGNQSSIVGYVPSSLSNNRDIDAILRAADEIQDEDPNIARILCEH

GYSLAQNLDPNSEGRGVLQFKTGLMSVIKQKLAKREVGTIDRSQDILRLQEFYRLYREKNNVDTLKEEEK

QLRESGAFTDELERKTVKRKRVFATLKVLGSVLEQLAKEIPEELKHVIDSDAAMSEDTIAYNIIPLDAPV

TTNATTTFPEVQAAVAALKYFPGLPKLPPDFPIPATRTADMLDFLHYIFGFQKDSVSNQREHIVLLLANE

QSRLNIPEETEPKLDDAAVRKVFLKSLENYIKWCDYLCIQPAWSNLEAINGDKKLLFLSLYFLIWGEAAN

IRFLPECLCYIFHHMVREMDEILRQQVARPAESCMPVDSRGSDDGVSFLDHVIAPLYGVVSAEAFNNDNG

RAPHSAWRNYDDFNEYFWSLHSFELGWPWRTSSSFFQKPIPRKKLKTGRAKHRGKTSFVEHRTFLHLYHS

FHRLWIFLAMMFQALAIIAFNKDDLTSRKTLLQILSLGPTFVVMKFSESVLEVIMMYGAYSTTRRLAVSR

IFLRFIWFGLASVFISFLYVKSLKAPNSDSPIVQLYLIVIAIYGGVQFFFSILMRIPTCHNIANKCDRWP

VIRFFKWMRQERHYVGRGMYERTSDFIKYLLFWLVVLSAKFSFAYFLQIKPLVGPTRMIVKQNNIPYSWH

DFVSRKNYNALTVASLWAPVVAIYLLDIHIFYTIFSAFLGFLLGARDRLGEIRSLEAIHKLFEEFPGAFM

RALHVPLTNRTSDTSHQTVDKKNKVDAAHFAPFWNQIIKSLREEDYITDFEMELLLMPKNSGRLELVQWP

LFLLSSKILLAKEIAAESNSQEEILERIERDDYMKYAVEEVYHTLKLVLTETLEAEGRLWVERIYEDIQT

SLKERNIHHDFQLNKLSLVITRVTALLGILKENETPEHAKGAIKALQDLYDVMRLDILTFNMRGHYETWN

LLTQAWNEGRLFTKLKWPKDPELKALVKRLYSLFTIKDSAAHVPRNLEARRRLQFFTNSLFMDVPPPKSV

RKMLSFSVFTPYYSEVVLYSMAELTKRNEDGISILFYLQKIYPDEWKNFLARIGRDENALEGDLDNERDI

LELRFWASYRGQTLARTVRGMMYYRKALMLQSYLERKAGNDATDAEGFELSPEARAQADLKFTYVVTCQI

YGRQKEDQKPEAVDIALLMQRNEALRIAYIDVVDSPKEGKSHTEYYSKLVKADISGKDKEIYSIKLPGDP

KLGEGKPENQNHAIVFTRGNAIQTIDMNQDNYFEEALKMRNLLEEFDRDHGIRPPTILGVREHVFTGSVS

SLASFMSNQETSFVTLGQRVLAKPLKIRMHYGHPDVFDRVFHITRGGISKASRVINISEDIFAGFNTTLR

QGNVTHHEYIQVGKGRDVGLNQIALFEGKVAGGNGEQVLSRDVYRLGQLLDFFRMMSFFFTTVGFYLCTM

LTVLTVYIFLYGRAYLALSGVGATIRERAILLDDTALSAALNAQFLFQIGVFTAVPMVLGFILEQGFLQA

IVSFITMQFQLCTVFFTFSLGTRTHYFGRTILHGGARYQATGRGFVVKHIKFSENYRLYSRSHFVKAMEV

ILLLVVYLAYGNDEAGAVSYILLTVSSWFLAVSWLFAPYLFNPAGFEWQKVVEDFKEWTNWLFYRGGIGV

KGAESWEAWWEEELSHIRTLSGRIMETILSLRFFIFQYGIVYKLKLQGSDTSFAVYGWSWVAFAMIIVLF

KVFTFSQKISVNFQLLLRFIQGLSLLMALAGIIVAVVLTPLSVTDIFACVLAFIPTGWGILSIACAWKPV

LKRMGMWKSIRSLARLYDALMGMLIFLPVALCSWFPFVSTFQTRMMFNQAFSRGLEISLILAGDNPNSGL

>CS10-A.thaliana 1904

MARVYSNWDRLVRATLRREQLRNTGQGHERVSSGLAGAVPPSLGRATNIDAILQAADEIQSEDPSVARIL

CEQAYSMAQNLDPNSDGRGVLQFKTGLMSVIKQKLAKRDGASIDRDRDIERLWEFYKLYKRRHRVDDIQK

EEQKWRESGTTFSSNVGEILKMRKVFATLRALIEVLEVLSRDADPNGVGRSIRDELGRIKKADATLSAEL

TPYNIVPLEAQSMTNAIGVFPEVRGAVQAIRYTEHFPRLPVDFEISGQRDADMFDLLEYIFGFQRDNVRN

QREHLVLTLSNAQSQLSIPGQNDPKIDENAVNEVFLKVLDNYIKWCKYLRIRVVYNKLEAIDRDRKLFLV

SLYFLIWGEAANVRFLPECICYIFHNMAKELDAKLDHGEAVRADSCLTGTDTGSVSFLERIICPIYETIS

AETVRNNGGKAAHSEWRNYDDFNEYFWTPACFELSWPMKTESRFLSKPKGRKRTAKSSFVEHRTYLHLFR

SFIRLWIFMFIMFQSLTIIAFRNEHLNIETFKILLSAGPTYAIMNFIECLLDVVLMYGAYSMARGMAISR

LVIRFLWWGLGSAFVVYYYVKVLDERNKPNQNEFFFHLYILVLGCYAAVRLIFGLLVKLPACHALSEMSD

QSFFQFFKWIYQERYFVGRGLFENLSDYCRYVAFWLVVLASKFTFAYFLQIKPLVKPTNTIIHLPPFQYS

WHDIVSKSNDHALTIVSLWAPVLAIYLMDIHIWYTLLSAIIGGVMGAKARLGEIRTIEMVHKRFESFPEA

FAQNLVSPVVKRVPLGQHASQDGQDMNKAYAAMFSPFWNEIIKSLREEDYLSNREMDLLSIPSNTGSLRL

VQWPLFLLCSKILVAIDLAMECKETQEVLWRQICDDEYMAYAVQECYYSVEKILNSMVNDEGRRWVERIF

LEISNSIEQGSLAITLNLKKLQLVVSRFTALTGLLIRNETPDLAKGAAKAMFDFYEVVTHDLLSHDLREQ

LDTWNILARARNEGRLFSRIAWPRDPEIIEQVKRLHLLLTVKDAAANVPKNLEARRRLEFFTNSLFMDMP

QARPVAEMVPFSVFTPYYSETVLYSSSELRSENEDGISILFYLQKIFPDEWENFLERIGRSESTGDADLQ

ASSTDALELRFWVSYRGQTLARTVRGMMYYRRALMLQSFLERRGLGVDDASLTNMPRGFESSIEARAQAD

LKFTYVVSCQIYGQQKQQKKPEATDIGLLLQRYEALRVAFIHSEDVGNGDGGSGGKKEFYSKLVKADIHG

KDEEIYSIKLPGDPKLGEGKPENQNHAIVFTRGEAIQTIDMNQDNYLEEAIKMRNLLEEFHGKHGIRRPT

ILGVREHVFTGSVSSLAWFMSNQETSFVTLGQRVLAYPLKVRMHYGHPDVFDRIFHITRGGISKASRVIN

ISEDIYAGFNSTLRQGNITHHEYIQVGKGRDVGLNQIALFEGKVAGGNGEQVLSRDVYRIGQLFDFFRMM

SFYFTTVGFYVCTMMTVLTVYVFLYGRVYLAFSGADRAISRVAKLSGNTALDAALNAQFLVQIGIFTAVP

MVMGFILELGLLKAIFSFITMQFQLCSVFFTFSLGTRTHYFGRTILHGGAKYRATGRGFVVQHIKFADNY

RLYSRSHFVKAFEVALLLIIYIAYGYTDGGASSFVLLTISSWFLVISWLFAPYIFNPSGFEWQKTVEDFE

DWVSWLMYKGGVGVKGELSWESWWEEEQAHIQTLRGRILETILSLRFFMFQYGIVYKLDLTRKNTSLALY

GYSWVVVLVVIVFLFKLFWYSPRKSSNILLALRFLQGVASITFIALIVVAIAMTDLSIPDMFACVLGFIPT

GWALLSLAITWKQVLRVLGLWETVREFGRIYDAAMGMLIFSPIALLSWFPFISTFQSRLLFNQAFSRGLE

ISIILAGNRANVET

>CS11-A.thaliana 1768

MRRQRPSVATARDAPSLEVYNIIPIHDFLTEHPSLRYPEVRAAAAALRIVGDLPKPPFADFTPRMDLMDW

LGLLFGFQIDNVRNQRENLVLHLANSQMRLQPPPRHPDGLDPTVLRRFRKKLLRNYTNWCSFLGVRCHVT

SPIQSRHQTNAVLNLRRELLYVALYLLIWGESANLRFMPECLCYIFHHMAMELNKVLAGEFDDMTGMPYW

PSFSGDCAFLKSVVMPIYKTVKTEVESSNNGTKPHSAWRNYDDINEYFWSKRALKSLKWPLDYTSNFFDT

TPKSSRVGKTGFVEQRSFWNVYRSFDRLWILLLLYLQAAIIVATSDVKFPWQDRDVEVALLTVFISWAGL

RLLQSVLDASTQYSLVSRETYWLFIRLTLKFVVAVAWTVLFSVFYARIWSQKNKDGVWSRAANERVVTFL

KVVFVYVIPELLALVLFIVPCIRNWVEELNLGVVYFLTWWFYSKTFVGRGMREGLVDNVKYTLFWIIVLA

TKFIFSYFLQIRPLIAPTRALLNLKDATYNWHEFFGSTHRIAVGMLWLPVILVYLMDLQIWYSIYSSLVG

ATIGLFSHLGEIRNIDQLRLRFQFFSSAMQFNLKPEEHLLSPKATMLKKARDAIHRLKLRYGIGQPFNKI

ESSQVEATWFALIWNEIILTFREEDLISDREVELLELPPNCWNIRVIRWPCFLLCNELLLALSQANELCD

APDHWLWSKICSSEYRRCAVMEAFDSIKFVILKIVKNGTEEESILNRLFMEIDENVENEKITEVYKLTVL

LRIHEKLISLLERLMDPEKKVFRIVNILQALYELCAWEFPKTRRSTPQLRQLGLAPISLEADTELLFVNA

INLPPLDDVVFYRQIRRVHTILTSRDPMHNVPKNIEARERLAFFSNSLFMTMPQAPSVEKMMAFSVLTPY

YDEEVMYRQEMLRAENEDGISTLFYLQRIYEDEWVNFLERMRREGAENENDIWSKKVRDLRLWASYRGQT

LSRTVRGMMYYYSALKKLAFLDSASEMDIRMGTQIAPEARRSYYTNDGGDNTLQPTPSQEISRMASGITH

LLKGSEYGSAMMKFTYVVACQVYGQHKARGDHRAEEILFLMKNHDALRIAYVDEVDLGRGEVEYYSVLVK

FDQQLQREVEIYRIRLPGPLKLGEGKPENQNHALIFTRGDAIQTIDMNQDNHFEEALKMRNLLESFKTYY

GIRKPTILGVREKVFTGSVSSLAWFMSAQETSFVTLGQRVLANPLKVRMHYGHPDVFDRFWFVPRGGISK

ASRVINISEDIFAGFNCTLRGGNVTHHEYIQVGKGRDVGLNQISMFEAKVASGNGEQALSRDVYRLGHRL

DFFRMLSFFYTTVGYYFNTMLIVFTVYAFLWGRLYLALSGVEKIAKDRSSSNEALGAILNQQFIIQLGLF

TALPMILENSLERGFLPAVWDFITMQLQLASFFYTFSMGTRTHYFGRTILHGGAKYRATGRGFVVEHKKF

AENYRLYARTHFIKAIELAIILLVYAAYSPLAKSSFVYILMTISSWFLITSWIISPFLFNPSGFDWLKTV

NDFDDFIAWLWSRGGLFTKADQSWFTWWNEEQEHLKTTGVWGKLLEIILDLRFFFFQYSIVYHLRIAENR

TSIGVYLISWGCIIGIVAIYITTIYAQKRYSVKEHIKYRFIQFLVILLTVLVVVMMLQFTKLTVVDLLIS

LLAFVPTGWGLISIAQVLKPFLLSTVVWDTVISVARFYDLFFGLIVMAPVALLSWLPGFQNMQTRILFNE

AFSRGLQISIILAGKKST

>CS12-A.thaliana 1780

MSLRHRTVPPQTGRPLAAEAVGIEEEPYNIIPVNNLLADHPSLRFPEVRAAAAALKTVGDLRRPPYVQWR

SHYDLLDWLALFFGFQKDNVRNQREHMVLHLANAQMRLSPPPDNIDSLDSAVVRRFRRKLLANYSSWCSY

LGKKSNIWISDRNPDSRRELLYVGLYLLIWGEAANLRFMPECICYIFHNMASELNKILEDCLDENTGQPY

LPSLSGENAFLTGVVKPIYDTIQAEIDESKNGTVAHCKWRNYDDINEYFWTDRCFSKLKWPLDLGSNFFK

SRGKSVGKTGFVERRTFFYLYRSFDRLWVMLALFLQAAIIVAWEEKPDTSSVTRQLWNALKARDVQVRLL

TVFLTWSGMRLLQAVLDAASQYPLVSRETKRHFFRMLMKVIAAAVWIVAFTVLYTNIWKQKRQDRQWSNA

ATTKIYQFLYAVGAFLVPEILALALFIIPWMRNFLEETNWKIFFALTWWFQGKSFVGRGLREGLVDNIKY

STFWIFVLATKFTFSYFLQVKPMIKPSKLLWNLKDVDYEWHQFYGDSNRFSVALLWLPVVLIYLMDIQIW

YAIYSSIVGAVVGLFDHLGEIRDMGQLRLRFQFFASAIQFNLMPEEQLLNARGFGNKFKDGIHRLKLRYG

FGRPFKKLESNQVEANKFALIWNEIILAFREEDIVSDREVELLELPKNSWDVTVIRWPCFLLCNELLLAL

SQARELIDAPDKWLWHKICKNEYRRCAVVEAYDSIKHLLLSIIKVDTEEHSIITVFFQIINQSIQSEQFT

KTFRVDLLPKIYETLQKLVGLVNDEETDSGRVVNVLQSLYEIATRQFFIEKKTTEQLSNEGLTPRDPASK

LLFQNAIRLPDASNEDFYRQVRRLHTILTSRDSMHSVPVNLEARRRIAFFSNSLFMNMPHAPQVEKMMAF
SVLTPYYSEEVVYSKEQLRNETEDGISTLYYLQTIYADEWKNFKERMHREGIKTDSELWTTKLRDLRLWA
SYRGQTLARTVRGMMYYYRALKMLAFLDSASEMDIREGAQELGSVRNLQGELGGQSDGFVSENDRSSLSR
ASSSVSTLYKGHEYGTALMKFTYVVACQIYGSQKAKKEPQAEEILYLMKQNEALRIAYVDEVPAGRGETD
YYSVLVKYDHQLEKEVEIFRVKLPGPVKLGEGKPENQNHAMIFTRGDAVQTIDMNQDSYFEEALKMRNLL
QEYNHYHGIRKPTILGVREHIFTGSVSSLAWFMSAQETSFVTLGQRVLANPLKVRMHYGHPDVFDRFWFL
SRGGISKASRVINISEDIFAGFNCTLRGGNVTHHEYIQVGKGRDVGLNQISMFEAKVASGNGEQVLSRDV
YRLGHRLDFFRMLSFFYTTVGFFFNTMMVILTVYAFLWGRVYLALSGVEKSALADSTDTNAALGVILNQQ
FIIQLGLFTALPMIVEWSLEEGFLLAIWNFIRMQIQLSAVFYTFSMGTRAHYFGRTILHGGAKYRATGRG
FVVEHKGFTENYRLYARSHFVKAIELGLILIVYASHSPIAKDSLIYIAMTITSWFLVISWIMAPFVFNPS
GFDWLKTVYDFEDFMNWIWYQGRISTKSEQSWEKWWYEEQDHLRNTGKAGLFVEIILVLRFFFFQYGIVY
QLKIANGSTSLFVYLFSWIYIFAIFVLFLVIQYARDKYSAKAHIRYRLVQFLLIVLAILVIVALLEFTHF
SFIDIFTSLLAFIPTGWGILLIAQTQRKWLKNYTIFWNAVVSVARMYDILFGILIMVPVAFLSWMPGFQS
MQTRILFNEAFSRGLRIMQIVTGKKSKGDV

>Phypa1-P.patens 1934
MASGEGAETGSTHKPRRTSRASAVGGVTESFDSEVVPSSLAAIAPILRVANEIESSTPRVAYLCRYHAFEKAHRIDPK
SSGRGVRQFKTALLQRLERDNEPTLALRHRRSDAREIQSYYQQYYNDYVKALDGAEHSDRAQLAKAYQTASVLFEV
LKAVNRDKTEEPPPEIIAAAADVEQKKEIYVSYNVLPLDAAGASQAIMQLDEVRAAVESLRNVRGLPWQTEKESHPR
AGDLDCLDWLQDMFGFQKDNVANQREHLILMLANVHNRLLPRPEPMHKLDDRALNAVMNKLFKNYKSWCKFLGR
KHKLWLPRIHQEERQRKILYMGLYLLIWGEAANLRFMPECLCYIYHHMASELHGMLAGNVSMVTGDNMKPAYGGKA
ESFLTLVVTPIYDVISRETLKNKNGTAPHSAWRNYDDLNEYFWKVDCFCLGWPMRTDADFFVPTQRSSQRSEDSNG
KFFQSTSKSFFVEIRTFWHLFRSFDRLWAFYILGLQAMIVLAWNVGPNLQYAFNGTVIKQVLSIFITASILRLIQAFLDLF
MGYHAFSSIKLLGVLRLILKLLVSAAWVIVLTVCYVRTWKNPQGLVGVIQKWFGSGWESSYLYIAAVVVYLVPNIIGAC
FFMFPMIRRWIESSNWPIVRVLLWWSQPRLYIGRGMHESQFALIGYTFFWVLLIASKFAFSYFIQIEPLVAPTKAIMQQ
TNVSYTWHEFFPKARNNPGALLSLWAPVILVYFMDSQIWYAVYSTIFGGISGSFRRLGEIRTLGMLRSRFSSLPGAFN
ESLVPDEDNRARKGFSFSRDFEKVAPPTNRSKAARFSQLWNEVITSFREEDLISNRERDLMLVPYSSDPDLKLVQW
PPFLLASKVPIALQMAKQAAETGRAADLLRKIKNDEYMKCAVVECYESFKRVLKRLIVGEVEIRVIEGLLAVVDENVEK
ETLLDNFNLGDLPLLSVKFIELLELLEKDYAGQEAIDNARDLVVLKLQDMYEVVTRDMMSETMRESWDTSHGALAGG

QGRKSELFSSKGDEPAKVLFPPPRKEAWIEQIKRLHLLLTERESAMDVPENLEARRRIAFFTNSLFMNMPRAPKVRN

MLSFSVLTPYYKEDVVYSKENLMKENEDGISVLFYLQKIYPDEWNNFLQRLGLENSDDPEAQIFSSNDLEDKLREWA

SFRGQTLSRTVRGMMYYRRALELQAFLDMATDDELEDGYKILTDATPEQKKSQRSTWSQLQAIADMKFTYVAACQ

MYGDQKRQGHHSATEILKLMLNNPSLRVAYIDEVEERQNEKTSKVYYSVLVKAVNGLDQEIYRIKLPGTVRLGEGKP

ENQNHAVIFTRGEGLQTIDMNQDNYLEEAFKMRNLLQEFHEPHGVRPPTILGVREHIFTGSVSSLAWFMSNQETSF

VTIGQRVLASPLKVRFHYGHPDVFDRLFHITRGGMSKASRVINLSEDIFAGFNSILRRGNVTHHEYIQVGKGRDVGLN

QISLFEAKIACGNGEQALSRDIYRLGHRFDFFRMLSCYFTTVGYYFSTMIVVLTVYIFLYGRIYLALSGVDDSLVHTAN

NKALTAALASQSLVQLGLLMALPMVMEIGLERGFRTALSDFLTMQLQLASVFFTFSLGTKTHYFGRTILHGGAKYRAT

GRGFVVRHERFADNYRLYSRSHFTKAIELFLLLIVYTLYVTKSAKGAVTYILITVSMWFLVASWLFAPFLFNPSGFEWQ

KIVEDWDDWNKWMSNRGGIGVEGSKSWESWWDEEQEHLNYTGFFGRLVESILSFRFFLYQYGIVYHLNIARSSNN

LSISVYGLSWLVIVAVLAILKIVSMGRDKFSADFQLMFRLLKALVFIGSVSVIAILHVKNLTVGDLFASILAFIPTGWALIQI

AVACKPVVINLGFWKSVKSLARGYEYMMGILLFTPIAVLSWFPFVSEFQTRLLFNQAFSRGLQISRILAGRKKL

>Phypa2-P.patens 1986

MERPPRPPQRISRRVLGNWEKLVNKAVRTELLGRDRYNRDGRRVGSSYGQRGERTPQSLAQQADIDAVLQASDEI

GRHNSQVACILAEHAYRLTQQLDPRSEGRGVLQFKTGLQSIIKQRKAMQEGTHDRSQDIRILQEYYKRYRAENRIDQ

LEAQAQFSTDLVPDSHEQSFERLRKVYETARILDDVVNALLKEAEPEDVSRLQNSDEKRVMEEDAQKLKGFKPYNIL

PPQRFLAETVEAPGILNPFEHFPEVVGATKALRYTKNLPRFPSDFIVPQDHILDIFDFLHYAFGFQKDNVANQREHIILL

LASAQSRLCTLDGRDGDSEKWADKAITDVHDRILQNYVRWCHFLRREPQNKRAFTQQRRLCLTALYLLVWGEAANL

RFMPECLCYIFHHLADECFDLLERTYVERSKTVKQNEDGSIEFSFLEQIITPVYNIVAKEAKASQNGKVPHSHWRNYD

DFNEYFWQPSCFLELGWPWRTDSGFFRPPVMKDAKQVAAPTQNAAQPRRIKHKVGKVHFVEHRSGFHLYHSFHR

LWIFLVCMLQGLTIWAFCSEDGKLNLHVRTIKKIMSVGPTFVVMKFIQSIFDVVFMWGAFKSTRLTTVARMLLRLLWFA

SLSAAILFLYVKTLQEDARNDGSGSWFRIYYILVSSYAGANVLFIFILRIPWLQRQAAKHSNVYFFQFVKWLHQERYYV

GRSMYERTRNYVKYSLFWIFILACKFSFAMHFQIMPLVTPTRLIIGFDNIVYKWPDFVSDSNHNALSILSIWAPVLMIYF

LDTQVWYTVVSAILGGIEGARDKLGEIRTLEMLRKRFPNYPAAFVKHMLPPINRFSSASSIQEPEPSTPKAKKTNKRD

AIRFQPIWNRVIKSLREEDLINNREKTLLKMPPNLMYHTNGTPNKLIHWPLFLLANKVHIAVELAAQHKTQDILGLWSK

VREDEYMGHAVQETYETLEPLLHLVLNSEGRRWVSEIFNSLRKSLNNGGDERDSFKMNKLRDVLVKLRDLTEHLGN

EHSPERQNKASDALKKLYEVVMHDFASENCRRIFTESSEHQRALVEESLFSELNWPNKSGQKQARRLNNLLTVQKI

KDQEGKTKTLNTETVPHNLEARRRLQFFTNSLFMHMPQAPPIRKMFSFCVFTPYYEEDVMYDMEKLYKENEDGISIL

FYLQKIYPDEWQNFLERIGLIENIVFREVGNPNPEKHKELKLELRLWASYRGQTLARTVRGMMYYKEALVIQGQQEG

ASGGDLEEGIPPSLVEAQGSIQRSAWAQAELKFTYVVTCQIYGEQKRKGKVQAADILYLMQKHDSLRVAYIDVVESS

GKDKKPSYYSKLCKVDRSDPKGSGANQRDQEVYSIKLPGDVKLGEGKPENQNHAIIFTRGDCIQTIDMNQDNSMEE

AFKMRNLLEEFKQPHGLHLPTILGVREHVFTGSVSSLAWFMSMQESSFVTLGQRVLARPLKVRMHYGHPDVFDRV

FHITRGGISKASRVINLSEDIFAGFNTTLRLGNVTHHEYIQVGKGRDVGLNQIALFEAKVASGNGEQTLSRDVYRLGQ

LLDFPRMLSFFYTSVGFYVCTMMTVLTLYVFLYGKAYLALSGVDASLRRNSQILQNPALESALNTQFLFQIGIFTAVPMI

VNLILEQGILKAIISFCTMQLQLASVFFTFSLGTRTHYFGRTILHGGAKYRSTGRGFVVTHIHFAENYRLYSRSHFTKAL

EVIMLLIVYLAYGAQNRTSVTFILLTFSSWFLALSWLFAPYIFNPSGFEWQKTVEDFEDWTNWLFYKGGVAVKTDNS

WEAWWVDEHDHIRTPRGRFLEIILSLRFFLFQYGVVYSLSVTRGTNSILVYAYSWFVLLGIVVIFKVFLVSQKSSASFQ

LAVRLFQGLFFSCLLAGLIVAVVLSPLTIGDVFSVALALVPTGWGLLSIAIALRPLMEKMRFWKSVREIARFYDACMGM

FIFIPIALLSWFPFVSTFQTRLVFNQAFSRGLEISLILSGNRSNRKT

>Phypa3-P.patens 2020

METSERSFRSRRDRDGARVAPRRAVPRRVVRNWEQLVGAAVSSALGNEDPDYSGSEEGEDESRMTSRSSRRYE

RSESYIVPQSLAAQTDIAAVMEVAEKIQDEDDDVGRILFEYAYNLTQQMDPLNQGRGVLQFKSALKAVLARNRIKHQI

DRSQDVRLLTEYHRMYKEREDIESLDAEEKAAQEGFGLEENPESQARRAKKRREFYKISKILNNAADFLVAVEPQVS

SIVDPAKEALDADAKKMEEFKPYNILPLESIGVTNPFQSFPEVVAATRALYTSQWSHFPRFEPDHSKTVGRDVLDIFD

FLHFAFCFQKDNVSNQREHLILLLANAESRVGTLSKGTTLAYNAKLDENAVKQVFDRILANYVRWCNFLNEKPQTLLA

MNSEKRLFLAALYLLIWGEAANVRFLPECLCYIFHHMAKECFELLDRNNVERATKTIKVDEDNIDYLFLDQIITPIYNIVA

AEAKNSEHGKAPHASWRNYDDFNEYFWQSSCFDLHWPWRLESGFFTKPRKKGDGKPSSREDTMPLLEANNSRR

ERRVGKINFVEHRSSLHLYHSFHRLWVFLVCMLQVLAVWAFCSENGRLNLRLRTVKFMLSVGPTFAIMKLLKSILDFA

FMWGAIRNTRKPIVLRMFVRLVWLLGLSGGIVYLYVKTLQEEARDTPSTPWFRLYCIVLGSYAGAQVFFTFVLRLPFL

RKQVDRCSNVRLCQFLTWMKEERYYVGRGMYERTKDYVKYSFFWGVVLACKFAFTMHFQLMPMVEPTRLIIGFEN

ITYRWHSFVSQGNKNIFTLVSLWAPVVMIYVLDLQVWYTVASALVGGLGGARDKLGEIRSLEMLRKRFLDCPEAFAK

QMETNRSSLTPAREDLAADEKKAIQNKDDARRFLPIWNAVINCLREEDLLDNRECDMLEMPPNSNTYPNGKQDTAIC

WPLFLLANKVHIAVDLAAENKHDDQQDIWEKVTVDEYMKFAIQESFQTIEQLLLSMFANNINAQRWIIDIFGDVRGRVA

DMAFVGLYKLHKLREVVDIIRDLTYYLGQEENPAVRKKAITELNRVSKVVMNDLLGRESSDRLRNWVLYQKFIQEEQL

FSDLLWPNEGWQKRATRLHNILKVHKFKDEADGKQKTYNTESIPKNLEARRRLEFFTNSLFMHMPKARPVSEMFSF

CVFTPYYSEDVMYDLKKKGAKKDKLKKDDIKELDRENEDGITILFYLRKIYPDEFKNFLERLKVTEKEFERQVWNPTY

MKEETKLELRLWASYRGQTLARTVRGMMYYKKALELQSAQDKGCSSDLESGGSSSSFRRGSLQRSPKAQAELKF

VYLVSCQIYGDQKKTGKPQAADILYLMQQNESLRVAYVDEVTIESGAKETTYYSKLVKVDKMDKGKDQIIYSVKLPGP

FKLGEGKPENQNHAIIFSRGDAVQTIDMNQDNYLEEAFKVRNLLEEFDQIHGRNRPTILGVREHVFTGSVSSLAWFM

SMQESSFVTLGQRVLARPLKVRMHYGHPDIFDRVFHFTTGGVSKASAGINLSEDIFAGFNTTLRQGNVTHHEYIQVG

KGRDVGLNQIATFEAKVASGNGEQVLARDVYRLGQLLDFPRMLSFFFTSVGFYVTTMMTVLTLYVFLYGKAYLALSG

VDASLKANNDILGNSALQSVLASQFLFQIGMFTAVPMIVNLVLEQGLLKAIMSFCTMQLQLASVFFTFSLGTRTHYFG

RIVLHGGAKYRSTGRGFVVRHINFAENYRLFSRSHFTKAFEIVMLLVVYLAYGAQNRTSATYILLTFSSWFLALSWLYA

PYIFNPSGFEWQKTVDDFEDWTNWIMYKGGVGVTSDNSWEAWWAEEQAHLRTAGGKFWEFILCLRFFFFQYGVS

YQLDVIQGSTSILVYVYSWILLFVCVLIFKVFTISQKASSKRATLHLAVRLFQAALLLGLITGGILAIIFSPLSITDVFALALG

IVPTGWGLISIAILFQPLVQYIGVWDSVREIARMYDAFMGIIIFIPIALFSWFPFFSTFQTRLVFNQAFSRGLEISLILAGN

RANTST

>Phypa4-P.patens 2110

MERHQGGRERRERIERGRSERSERTERFGRSEISEESEGSSVSRRGSVRGSEGSSRFRMNVVRNWEELVRKALW

AYREESDEFDSGSDEGEEEDEEESVVRKSSGWRQKSQSYSVPQSLAQQTGIDAVMAFAEEVDKDHSNVARILFEY

AYNLTQQMDPMNQGRGVLQFKSALKAVLITNRIKANRPTQQTDPSQDVKILTEFYSMYKEAHDIDHLQEQDRAARE

GHIQDGTDEYQEWRAGKLRKFYEASKILNSAVKYYRRLSESDVSNVEVEPQVGSSIRDRPKTGPTAPISSDVKPTGS

YAAEAALDIDAKKIDQFKAYNILPLESTGVPNPFQSFAEVVAATKALYTTEWLQFPQFDRGYSKKVGRDVLDIFDFLHY

AFCFQKDNVSNQREHLVLLLANAETRADKPCNGAAPHNAKLGEKAIETVHDRILANYMRWCKFLNLNDHTKWASNP

QKKLCLTALYLLIWGEAANVRFLPECLCYIFHNMADDCFSLLEKDKPARSTVTLKIEDIKNSVTNTEYLFLEQIITPVYEI

VAAEAANSQHGKVPHGSWRNYDDFNEYFWQPSCFELGWPWKLEACFFTKRGDKKTINSRTELDPLLGSDSRKAP

PVGKIHFVEHRSSLHLYHTFHRLWVMLVCMLQILAVWAFCSENRKLNLHLRTIKKMMSVGPTFAIMKLFKSILDFVFM

WGAMKSTRKQIVSRMLIRLIWLICVSSALVFLYVKTLQEDARNHSSTPWFRLYSLVLGCYAGAQVFFAFLLRLPFLRK

QFDSCSNVRACQFIKWIQEERYYVGRGMYERTSDYLKYSLFWIVVLACKFAFTMHFQLLPMVEPTRIIVGFKNITYS

WHSFVSKGNHNVFTLVSFWAPVIMIYVLDVQVWYTVASALLGGLEGARDRLGEIRSLDTLRNRFLYFPQEFVKKMD

ATMGGKKAKSRSISSKDDARRFLPIWNAVIESLREEDLLSNTERLMLEMPPNSRTYPNGKEDTQMCWPLFLVANKV

QIAVDTAADEVKKSKERDFHLAPSFTEVSRGDYQIELWEKVSSDEFTKFAIEESFHTLEQLLLSLFRENDNPWLWLQR

LFGDVRAKVAAGGFVIQYNIEKLPLVVKKLADLTKHLAGEENEERRKASISLLDELARIVMNDMLNLNGNDIPSDFLRF

KKLIQEGRFFKNLIWPDEAWRKRADRLQNIFKIHTYFDKDRNKKTYDTHTVPKNLEARRRLEFFTNSLFMNMPDARP

VAKMFAFCVFTPYYSEEVMFDIKKRDGKKKYDKPKKDSDIKELDVKNEDGITILEYLKTIYPDEWKNFLQRLGLTEGT
FHSHVWPDSAKGQKSDTILKLRLWASYRGQTLARTVRGMMYYKKALELQAELERSSVSDPERGVPSSSVHNQRDL
LQRTPQAQADLKFVYLVSCQIYGDQKQKGLAQAKDILYLMQQNESLRVAYVDTVNGELGAKSKTTYYSKLVKVDKM
DKGKDQVIYSVKLPGPFKLGEGKPENQNHAIIFSRGDAVQTIDMNQDNYLEEAFKVRNLLEEFDKVHGRNPPTILGV
REHVFTGSVSSLAWFMSMQEASFVTLGQRVLARPLKVRMHYGHPDIFDRIFHFTTGGVSKASCGINLSEDIFAGFNT
TLRQGNVTHHEYIQVGKGRDVGLNQIAMFEAKVASGNGEQLLARDLYRLGQLLDFPRMLSFFFTSVGYYVTTMMTV
LTLYAFLYGKAYLALSGVDASLKSLNDILGNEALQSVLASQFLFQIGVFTAIPMIVNLVLEQGIRKAIMSFCTMQLQLAS
VFFTFSLGTRTHYFGRIVLHGGAKYLATGRGFVVRHIKFRDNYRLFSRSHFTKAFEIILLLVIYLAYGAQNRSSVTYILLT
FSSWFLALSWLFAPYVFNPSGFEWQKTVDDFGDWQKWILYKDGIGVNSETSWETWWLDEQSHLRTTAGKFWEIVF
SLRFFFFQYGVSYHLDVFQGSTSIMVYVYSWITLCGCVAIFTVFSSSTAIALKHSHRHFTVRLFQAALFVLLIGGVIVAI
ALSPLAVTDCLAVALAIVPTGWGIISIAVVFQPQLKGFKIWYSVKEIARLYDMCMGLIIFIPIAVLSWFPFFSLLQTRLVFN
QAFSRGLEISLLLAGNRANASV

>Phypa5-P.patens 1989
MDTPQRTPRRISKRVLMRWEALVYRARMAAALRGAGEHQALGGASNTTVPETLRSQRANINAILETADELGKQDHE
VARILCEHAYTLVQNLDPYSEGRGVLQFKTGLLSVIKQNRSRTAGEKIVRSLDAVKLQEFYKKYREKNHLDKLEAEAK
TSRESDSYDEDSATIEQRTELQRRVYLTARIINEAIDALTEDGQTEDLDPELKRIMEEDANKLREYKPYNILPLETPGVT
NAFSSFPEVIGAARALEYKVSSSELPDFPEDFDKPQERRVDVFDFLQYTFGFQEDNAANQREHLILLLSNSQSRLGV
LVDTEIKLDDGAISHVYLSMMENYERWCKFLGRESMAKRAYSMQLMIFLTALYLLIWGEAANLRFLPECLCYIFHHMA
DEMYDLLDKREVERSRTFIHGSSHSFLDKIVKPVHEILAAESKMCAAGNAPHSDWRNYDDFNEFFWSPSCFELSWP
WRLDAGFFRKPEKKIYTDADRCLRNRDHLEEPITLEQESLPHTGEPQEKKVGKTHFVEHRTGFHIYHSFHRLWIFLV
CMLQGLGIFAFCDRRLTLRNIKLIMSVGPTFILMRLIQSVMDVTLMIGAYRSTRKRNISRMLIRFVWFIVLSTVVVLLYVK
TIEEENSGSGADTWFRIFYWVLGTYAVIHMVIALLLRVPWFRMQAERCSNFYVLQFIKWVHQERYYVGHNMYERTR
DYFSYTLFWFIVGTCKFAFSYFLQIQPLVEPTRTIIGIRNVNYRWKDLISQSNHNALTLVALWAPVIMIYFLDTQVWYILV
SALIGGFAGARMHLGEIRNLDMLRSRFFSLPGAFVTTLVPTRSVSHSQMDMSVPISAPKDLTDNAKVDAIRFAPLWN
EVILSLREEDLINNREKEWLLMPDNKIRLGASGQQTLVQWPLFLLANKVYIGIDIVLENRNFFQNELWDRIKRDRYLEN
AVQEAFVSLQSVLLHLLNEDGRAWVDKIYEDIYNSLDTGNVLHFFDFKNLLSVLNRVTELTEILVAEPTKVEESEMQE
EQLKMQDRAVRALVGLYEVVMRDFLADSELREYYEQEEKLQSAKLDGSLFSDLNWPTGLFKDQVKRLHYILTIKESA
LNVPVNLEARRRLQFFSNSLFMSMPQPPPVRKMFSFSALTPYYNEDVMYSKAQLEDKNVDGITILYYLQTIVPDEWK

NFLERMIPGVDYNQLGLYTEANIDAIDIVQLRLWASYRGQTLARTVRGMMYYKKALLLQAQQEGASVAEDEEEGHDI
VTDELALVSLSTPRTPRGSLVRNARSQAELKFCHVVTAQNYGKQKNSLLTADKDRAADLLRLMQMYDSLRLAYIDEV
KKMVQGKEITEFYSKLVKTDLSGKEQEIYSIKLPGEVILGEEKSENQNHAIVFTRGEALQTVDMNQENYLEETLKIRNL
LEEFDSKKLGFRRPRILGVREHVFTGSVSSLAWFMSLQERSFVTLGQRVLANPLKVRMHYGHSDVFDRIFHITRGG
VSKASKQINLSTDIFAGFNSTLRQGNTTHHEYIQCGKGRDVGLNQIAAFEGKVAAGNGEQILSRDVFRLGQLFDFFR
MLSFFFTSVGYYFTTMLAVLTIYVFLYGKVYLALSGVDAALKANSLLDNTALLAALDTQFLLQIGVFTTVPMIVNFVLEQ
GVMRAVISFFTMQFQMSSLFFTFSLGTRTHYFGRTILHGGTKYKSTGRGFVVEHVPFAENYRTYARSHFVKGMEIIIL
LIVYVVYGAHDWTAASYILLTFSSWFLALSWLFAPFVFNPSGFEWQKTVKDFEDWTNWLFHKGGIGDEGKKSWEV
WWNEEQAHIHTFRGRLWEIILSSRFFLFQYGIVYALNAAGNNKTFWVYGYSWVVIVGVFLLFKIFTFSQKASANFQLI
VRLFQGIVFLAVVAGVSVAVVLTELTIGDLFACSLALIPTGWGLLSIAIALRPVFKWFGLWKSVRGIARFYDATMGMILFI
PIALLSWFPFVSTFQTRLVFNQAFSRGLEISVLLAGDNPNAAI

>Phypa6-P.patens 1981
MSTRSREPQRISKRILNRWETLVYRAKMEADRRAVLVPQAGAASNTTVPQSLHQQANISSILQAADELAKDNRDVG
RILCEYAYTLAQDLDPNSEGRGVLQFKTGLLSVIKQKRSKKGVERIDRSHDVSILQDFYRRYRERNHLDQLEDEDRR
FKQSDSYDEDSTTTEQRGEVIRKVYLTARILNEVIDALMKHDDRVENFNPELKRIMEEDAQKVKGFKAYNILPLETPG
VANVFHNFPEMVGAKRALEYNSSTSELPSFPEENFERPSDRALDIFDFLQYAFGFQTDNAANQREHLILLLSNSQSR
LGVLVDMEAKLDDGAINHVHLSMMSNYERWCKFIKKESMAMRAYSMQLRLFLTALYLLIWGEAANLRFLPECLCYIF
HHMADEMYDLLDEPVVKRSRTFIPGSSHSFLDKIIKPVYDIVAAEAKICAGGKAPHSAWRNYDDFNEFFWAPSCFEL
SWPWRLEAGFFKKPKQIIYSEADRYLQEQIPDEPEEPLMLSERREKKAWKTHFVEHRTGFHIYHSFHRLWIFLVCML
QGLGIVAFCDRRFTVRTLKLVMSVGPTFVLMKLLQSLMDVTLMIGAYRSTRAGNISRMLIRFLWFTVLSGIVVLLYVKTI
EEENSGTGRDTWFKAFYLVMGICGGLQFIFALLLRVPWFRMQAEKCSNFYVVQFIGWVHQERYYVGRNMYERTRD
YFTYTFFWFIVGTCKFAFSYFLQIQPMVGPTRTVISIKNFNYRWRDLISQSNYNALTLVAMWAPVVMIYFLDTQVWYIV
ISALVGGLDGARMHLGEIRSLDMLRSRFSSLPGAFVNNLFPSRSGAQSQMDVNVPLSSVKPGNPKVDAIRFAPLWN
EVISSLREEDLINNREKDWLMMPDNKITSTSLGQQTTLVQWPLFLLANKVYDALDIVHDNRQAFQDELWDKIKRDPY
LEFSVREAYESSQTVLWDLLNEDGRGWVRNIYQDIDNAIEASCLLSKFNFGELGNLLIRMAKLTNILVTETKVDDENG
KQEEESKLHYSAARALVDLYEDVMRDFVVDPGLRTIYEADTTLQNSKLNGVLFNKLNWPTGPAKERVRRLHYILSIK
DSALNVPVNLEARRRLQFFSNSLFMSMPQSPLVRKMISFSVFTPYFEEDVMYSKAQLENANVDGITILYYLQTIVPDE
WINFLERIFPNVEYNQLNTLSDADIIGDKILELRLWASYRGQTLARTVRGMMYYKRALLLQAQQEGASMTDEEEGHIV

QGNELATIGVETPRTPRGSLVRNARAQAELKFSYVVTAQLYGKLKNSVISAQQEKAADILYLMQKNDSLRIAYIHETKE
IVDGHLVTEYHSKLVKADPSGRDEEIYSIKLPGEVNLGEGKPENQNHAIVFTRGEALQTIDMNQEHYLEETLKMRNLL
EEFDSKKHGLRRPTILGVREHVFTGSVSSLAWFMSLQERSFVTLGQRVLAKPLKVRMHYGHPDVFDRIFHITRGGIS
KPSKQINLSEDIFAGFNSTLRRGNITHHEYIQCGKGRDVGLNQIAAFEGKVASGNGEQSISRDIYRLGQLFDFFRMCS
FFFTSVGFYFTTMLTVLTVYVFLYGKVYLALSGVDESLRANGLLENTALQSALNTQFLLQIGIFTAVPIIVNFILEQGILQA
VISFLTMQFQLSSVFFTFSLGTRTHYFGRTLLHGGAKYKSTGRGFVVEHIPFAENYRTYARSHFVKGMEITMLLIVYLV
YGAHDRNTASYILSTFSSWFLALSWLYAPFIFNPSGFEWQKTVKDFEDWTNWLFHKGGIGDEGKQSWMVWWDEE
QSHIQTPRGRFWEILLSLRFFIFQYGVVYALNVSGSNKSFWVYGYSWVVMLCVFVLFKIFTFSQKASANFQLIVRLFQ
GIVFLAVVTGVSVAVALTPLTVGDVFASLLALIPTGWGLLSIAVAMRPVIKWFGLWKSVRGIARLYDAAMGMILFMPIAF
LSWFPFVSTFQTRLVFNQAFSRGLEINILLAGNNPNAAI

>Phypa7-P.patens 1979
MSTRSREPNRISKRILHRWETLVYRAKMAAERKDLTMSHAGSSSNTTVPQSLLQQANVDSILQAADELALENPDVG
RILSEYAYTLVQDLDPNSEGRGVLQFKTGLLSVIKQRRSKKGAEKINRSQDINILQDFYRTYRERNHLDQLEDEDRRF
KQSYSYDEDSTTTEQRGKLIRKIYTTARILNQVIDALLKQNEKYEDFDPELKRIMEEDAQKEKGFKAYNILPLETPGVA
NVFHNFPEVVGAKRALEYNSSSATLPAFPEENFERPSDRPLDIFDFLQYVFGFQVDNAANQREHLILLLSNSQSRLG
VLVDTENKLDDGATNGVHISMMKNYEWWCRFLNKDSMAKRAYSTQLRLFLTALYLLIWGEAANLRFLPECLCYIFHH
MADEMYDLLDEDEVKRSRTFLSDSPHSFLDNIIKPVYDILAAEAKVSADGRNPHSAWRNYDDVNEFFWAPTCFELS
WPWRLDAGFFKKPEKVIYSEADRCEQIPEEPEEPQNLGEKREKKVGKTHFVEHRTGFHIYHSFHRLWILLVCMLQG
LGIFAFCDRRFTVRTVKFVMSVGPTFVLMKLLQSVMDVTLTIGAYRSTRARNISRMLMRFTWFTILSAVVVVLYVKTIE
EENGGSGTNTWFRAFYLVMGICGGLQLFFALILRVPWFRMQADKCSNFYVVQFVKWVHQERYYVGSKMYERTRD
YFTYTLFWFVVGTCKFAFSYFLQIHPMVEPTRTIIGIRNINYRWKDLVSQNNYNALTLVSLWAPIVMVYFLDTQVWYTII
AALVGGLVGARMHLGEIRSLDMLRSRFSSLPGAFVKNLVPSRGGCHSQMDVNVPLSAVKPGNPKVDAIRFAPLWNE
VVLSLREEDLINNRERDWLLMPDNMITLTALGQHTLVQWPLFLLANKVYIGLEIVHENRHGNQAELWDRIKHDTYLDY
AVREAYASSQSVLWDILNEDGRAWIRRIYQDIDDAIESSLLLKKFNFEDFGDVMEKILNLTEILVTEPKKDDEDGRHEE
ESKLHESAIGALVDLYEVVMRDFIMDSNLRANYESDTVLQASKQDGSLFSQLKWPTGQAKEQVRRLNYILAIKDSAL
NVPVNLEARRRLQFFSNSLFMSMPQPPPVRKMISFSVLTPYYEEDVMYSKKQLEDANEDGITILYYLQTIVPDEWTN
FLERMYPNVGYNQLKTFSEKAFSEEQFLELRLWASYRGQTLARTVRGMMYYKRALVLQAQQEGASMEEDEEGGH
NLEGNELTIVNVNTPRTPKGSLVRTARAQAELKFSYVVTAQNYGKHKSSSTPTQQEKAADILYLMHKNDSLRIAYIHE

AKKTIRGNLVSEYYSKLLKASPGGKDEEIYSIKLPGAVTLGEGKSENQNHAIVFTRGEALQTIDMNQEHYLEETLKMR
NLLEEFDSKDHGLRSPTILGVREHVFTGSVSSLAWFMSLQERSFVTLGQRVLAKSLKVRMHYGHPDVFDRIFHITRG
GISKSSKEINLSKDIFAGFNSTLRQGNITHHEYIQCGKGRDVGLNQIAAFEGRVASGNGEQTISRDIYRLGQLFDFFR
MCSFFFTSIGFYFTTMLTVLTIYVFLYGKIYLALSGVDEVLKQNNLLENTALQSALNTQFLLQIGIFTALPMIVNFILEQGV
LPAVISFLTMQFQLSSVFFAFSLGTRTHYFGRTLLHGGAKYKSTGRGFVVEHIPFAENYRTYARSHFVKGMEIIMLLIV
YVVYGAYNRSNASYILLTFSSWFLALSWLYAPFIFNPSGFEWQKTVIDFEDWTNWLFHKGGIGDEGKKSWEIWWDE
EQAHVQTFRGKFWEIIFSLRFFIFQYGIVYTLDAAGNDKSLWVYGYSWVVLLGIFLLFKIFTFSRKASANFQLIVRLLQ
GVVFLAAVAGVSVAVVLTRLTVGDVFASILALVPTGWGLLSIAIPLRPICKWFRIWGSVRGIARLYDAAMGIVLFMPIALL
SWLPFVSTFQTRLVFNQAFSRGLEINILLAGNNPNPAL

>Phypa8-P.patens 2008

MAMPPRLPHLVRKAVLAKWERLVGLAIEAEEERTLHESYGDGYGDTSVVPQILQQKKNIDDILQTARDVEQTYPQVA
RILFEYAYALSQNLDPRSESRGVLQFKTGLLSIIKQTRGEKTDRSQDVYIIEEFYKHLKRNLDQLEDEDWLRRQPQYIQ
RSPEEWTEMKRKIYVTCQILNEVLDFLIKENPEMQRHVEFDSDLKEDLEKTAKKVEDYKPYNILPFEAPGVVNPFENS
LEVMAAINTITLNLPDGYEFGADFTPPRTRNLDIFDFLQYGFGFQTDNVLNQREHLVLLLANSQSHLGSLGNRDSDA
SLKLDQSSIISVHSKLLENYERWCDFLRKEKYSNFRFQDSAVIPQPRLLFSALYLLIWGEASNVRFLPECICYIYHHMA
YEICAEGTDSLSKNGFRQKSIILRDSDSFLDAIIKPIHEIVAAEAKVCNHGKSPHSRWRNYDDFNEYFWAPFCFELGW
PWRLNSGFFVKPKQITNKKTSKFKSREAQDQVPLLLDRDQRSEPSQRRERKAGKSHFVEHRSGLHLYHSFHRLWIF
LVCMLQGLAIFAFCDAKLNSVSIKYILSVGPTFVAMKFLQSVLDVILMIGAYRSTRARTLSRIWLRLIWFASLSAAIIILFV
KTIQEQDSGSNSSTWFRLYCILLIIYGGSQLFVALLLNMPWLRRLTEKYFNFGPLSFLNWVHQERYYVGRGMYESTG
DYLSYILFWLLVLACKFSFSYFLQINTMVKPTRAIIDIKNIDYRWRDIFSKSHHNALTLVSLWAPVVMIYFLDLQIWYTVIS
ALVGGLNGARIGLGEIRSLHMLRTHFSSLPSAFTKRLQPNQPHQEFMAMRESADMRKPKLDARRFAPIWNEVIISLR
EEDLISNKERDLLVMPLNISTPLTTSSQPLTLIQWPLFLLANKVYVACDMAEVHKQANQDDLCEKIGKDPYMMFAVQE
AFYVLRIILEYLLMNDQGALWVTKVYEGLEQAMHVRQLRNKFNLRKSQLRKLLDKAAGLTTVVIRESKKIDDLVKEGA
LDDKMKKEYTESLRKELLDFYDVVMRDFIADSELRNDAEGNYELQTAKQSGRLFSDLALPTEESKALVERLHSILTFK
ESALNVPENLEARRRLEFFSNSLFMRMPNAPSVRKMLSFSVFTPYYSEDVIYSPQQLAKENDDGISMMYYLRTIVPD
EWNNFLERVYPKKEDREAKKALLKTIFPKEFKFKENEQPRKPEDLNEDVKLKLRLWASYRGQTLARTVRGMMYYKR
ALVLQSQQEGATVSEDLEQGRQYLTSAASQVPGVLNARAQAELKFLYVVSAQIYGEQNQGDKGAEGRQKAADISYL
MKTFDSLRISYIHKAKVKTEGKEVTEYYSKLMKADPSGNDQEIYSIKLPGEVILGEGKPENQNHAIIFTRGEALQTIDM

NQEHYLEETFKMRNLLEEFNESRRYGHRNPTILGVREHVFTGSVSSLAWFMSLQERSFVTLGQRVLANPLKVRMH

YGHPDVFDRIFHITRGGISKASKQINLSEDIFAGFNSTLRLGNVTHHEYIQCGKGRDVGLNQIAAFEGKVASGNGEQT

LSRDIYRLGQLFDFFRMLSFFFTTVGYYFTTMLTVLTVYVFLYGKVYLALSGVDQNLKDQGLSTNVALQSALDTQFLL

QIGVFTAVPMIMNFVLEEGILKAIISFLTMQLQLSSVFFTFSLGTRTHYFGRTILHGGAKYASTGRGFVVAHIPFAENYR

MYSRSHFVKALEIMLLLIVYLAYGASERTTLTYVLLTFSSWFLAISWLWAPYIFNPSGFEWQKTVADFDDWTNWLFHK

GGIGDEGKKSWEVWWLEEQAHIQTPRGRFWEIVLSLRFFLVQYGVIYALNVVGHDKGFRVYGFSWCVLVGIVLTFK

VFSMNQKSWANFQLFLRLFQMTVFLAIIGGVIVAVAMTALTIGDVFACALSLIPTGWGLISIAIAIRPVMKRLGLWKSIRA

IARLYEAFMGAIVFIPIAILSWFPFVSTFQTRLVFNQAFSRGLEISTLLAGNNPNSNM

>Phypa9-P.patens 1960

MAPHSHVPHLERRRVVGRWEELVSKAIDAKGKRPFLRLSRGEYEDTVVPQFLQEQNNKISDILQTAHDVENDYPIVA

RILFEYAYDLSQKMDPKSESRGVLQFKTGLLKAIKLRADGEKTDRSEAISMLQDFYQYLKGHIDRLEDENVSREQRK

KYNKTPEEWTELKRKVYITSQILNEVVDYLSPKTNQDVQFDSDLKEDLKKTAEKVNDFKAYNIIPFEAPGVVNPFQYS

PEITAAIKSIEFEPSGGHEFGVDFKPPKMRNLDIFDFFQYAFGFQADNVLNQREHLLLLVANAQSRVNNIVKAISNVEE

KLLGNYERWCKYVKRVNSTSRKPLDSSPRSMKLFWAALYLLIWGEAANVRFLPECLCYIFHHMAFETYELLNNPFN

QKSTILKDSETFLDAIIKPVYEVVAAEAKVCNHGKSPHSSWRNYDDFNEYFWAPSCFELSWPWRLHSGFFVKPMQV

SDKVKKFKFRKAQDQMPLLEQNHRSEGEHAMERKAGKSNFVEHRTGFHLYHSFHRLWIFLVCMLQGLAIFAFCDGK

LNNANIKYVLSVGPTYFIMKLLQSALDVILMIGAYRSTRYRTVARVWLSLIWFAGFSGIITILYVKTIQEQNSGSGLSTW

FRLYCIPLIFYGGSELFIWLFLNMPGLRILAASCSNFGPTRFLKWVHQEQYYVGRGMRESSSDYFSYLVFWAIVLACK

FSFSYFLQIKSMVGPTRIIIDLTDINYRWRDIVSKSNHNALTLASLWAPVVMIYFLDLQIWYTVISALVGGFDGARIGLGE

IRDLEMLRRRFFSLPSAFTTKLLPHESFQKNSRESVNNDESKVNAMKFAPIWNEVITCLREEDLISNKEKELLLMPNN

KVSRTPPLNDLLLIQWPLFLLSNKVFSAIDTVNAYKQSKNKELWDKIKDDRYMMYAVQEAYYSCKNILEYLLVKDQGV

LWVKSIFALVEAIKPDEHLNDIFRFNKLTKLLDKVANLTGVLINEAANEVFTVAAVREKLLDLYDMVTRDFVREETNVSV

DQQLFSEFSLPTHEFISQVRRLNSILTSKESASEVPVNEEARRRLEFFSNSLFMTMPKSPPVRKMFSFSVFTPYYSE

DVIYSIEKLTKPNDDGISIIYYLSTIVPDEWKNFLERQFPNDLEARRIFAKTVLPKKDYKKEKEKSRSLDDLVDEDKNQL

RLWASYRGQTLARTVRGMMYYKKALILQAEQESTYGSEDLEQGLHHSTPSSPSDSGVVTARAQAELKFLYVVSAQL

YGEQKQSTNPEDRQRATDIKWLMKEYDSLRISYIHKAKVTKRDKTKVYEYYSKLMKGLPDGNDQEIYSIKLPGEVILG

EGKPENQNHAIVFTRGEAIQTIDMNQEHYLEETFKMRNLLEEFEIQYGGRFPTILGVREHVFTGSVSSLAWFMSLQE

RSFVTLGQRVLAKPLKVRMHYGHPDVFDRIFHITRGGISKSSKQINLSEDIFAGFNSTLRLGNITHHEYIQCGKGRDV

GLNQIAAFEGKVASGNGEQTLSRDIYRLGHLFDFFRMMSFFFTTVGYYFTTMLTVLTVYVFLYGKVYLALSGVDAQL
KIKGLASNVALQSALDTQFLLQIGVFTAVPMIMNFILEEGLLRAITSFFTMQFQLSSVFFTFSLGTRTHYFGRTILHGGA
KYASTGRGFVIEHIKYAENYRNYSRTHFVKALEIMLLLIVYLIYGAPERTTFTYILLTFSSWFLAVAWLWAPYIFNPSGFE
WQKTVKDFENWTNWMFQQEGQDEKDDKCWEVWWKGQISHIRTLRGRFWEIALSLRFFMVQYGVAYSLNVAGHD
KSFRVYGFSWCVLVLIVVLFKVFSLSKKSLANFQLIVRILQLVVFCGVICGLIFTVAFTSLTIGDVFASVLSLIPTGWGLL
SIAIALKPVMKKLRLWKFVLAIARLYDVFIGAIVFIPIAFLSWFPFVSTFQTRLVFNQAFSRGLEISTLLAGGNPDVAGNQ
SQHSKTR

>Phypa10-P.patens 1781
MENPDMAYPVYNILPVDDPSADHAGMSFPEVKAAITALKKVDDLPMPPDVAWTPDMDMLSWLGSFFGFQADNVKN
QREHLVLLLSNGMMQLYHAGPTFETLEASIVRKTRKKVTENYVSWCKFIGRKHHLKLPDGKHTEHFDERRELIYICLY
LLIWGEAANLRFMPECLCFIYHHMLGELNRLLEFSGADDVLAVMPTYTGVNGFLNHVVVPLYTILKLEADSNNNGTAP
HSSWRNYDDLNEYFWTSRCFKQLQWPLQTKSSYLVEPRREKGYLSRGRKPQSEKVGKTGFVEQRSFWYIFRSFD
KLWIGYLLMLQASVVLLWHNGGAPWIELQKPDPFARFMSIFISWALLRFLQGLLDVGSQYSLVSKDTKLIGVRMVLKL
LVAATWAILFIIYYRRMWWQRNIDQYWTEIANQKLYEFLYIAAAFIVPEVLAILLFIVPWVRNFVETSTWKVFHLMTWW
FQSRGYVGRGLREGIMDNVRYTLFWACVLTSKFAFSYWLQIRPLIAPTKQILEATDVRYKWHEFFPDGNRAAVVALW
APVLMIYFMDTQIWYSIWSSGIGAFVGLLQHLGEIRNVEQLQLRFQIFPSAFQFSLMPVDDSVTRTVWAGAKDLLKRL
SLRYGWSSVYDKMEWGQIEGGRFAHVWNEIIKTFREEDLISDREVELMEIPQGAWRVSVFQWPSTLLANQILLALYS
IRYHRGDDKSVWNIICKNEYRKCAVVESYESMKHVIRKILKDDSDEFHIFIAIFEEIDFAIRKDRFTETFKLPELMEIHAR
VVELISFLLTRPAEKHKQKVVKDLQNLYEGLLHDFPLQPHIFLESIKARASYPQNNKGTELFMDAVELPDKGDEHFFK
NLKRLHTTLSTRDPLLYVPKGLEARRRISFFSNSLFMTMPRAPQVERMLAFSVLTPYYNEEVIFSKHQLKEENEDGV
TILFYLQRIFPEDWLNFLERMKKLELNESELWEKDDALELRLWASFRGQTLARTVRGMMYYKRALEVQTFLDSATED
ELLGIKELLERGSSTNSRGSMRSIGSMGSIGSELEVAELNRQRKLEQDLANAAMKFTYVVTCQIYGAQKKANDVRAA
DILRLMKTHTGLRIAYVDERSESYFDENIGEYVTRQLYYSVLVKYDPDLKQEVEIYRIRLPGPLKLGEGKPENQNHALI
FTRGDAVQTIDMNQEMYFEEAIKMRNLLQEFTVYHGTRKPTILGVREHVFTGSVSSLAWFMSAQETVFVTLSQRVL
ANPLKIRMHYGHPDVFDRLWFLTRGGISKASRTINISEDIFAGFNCTLRGGNVTHHEYIQAGKGRDVGLNQIAMFEAK
VASGNGEQILSRDVYRLGHHLDFFRMLSFYYTTVGFFVSNMMVVLTVYTFLWGRVYLALSGIEESLTSGSPALENSA
LTATLNQQLVVQLGLLTALPMVVEDALEHGFTTALWNMITMQLQLASIFFTFSMGTRCHYFGRTLLHGGAKYRATGR
GFVVKHEKFAENYRLYSRSHFVKGIELLLLLLCYLAYGVSSSSGTYILVNISSWFLALTWVMGPFVFNPSGFDWLKTV

EDFGDFMQWIWFKGDVFVKVEQSWEIWWEEEQAHLRTTGLWGKLLEIVLDLRFFIFQYGIVYHLGITGNNTSIFVYL

ASWSYMLFAALLHFILSNANEKLAANNHGLYRAIQALAIAIITALVVVLWVVTNFTFVDIIASFLAFLPTGWGIIQICLVLR

RPFLENSPLWSTIVAVARLYDLAMGIIVMAPVAVLSWLPGFQAMQTRILYNEAFSRGLQISRLLAGKRNRNID

>Phypa11-P.patens 1784

MADRVYNILPVDDPHAVDHAGMMFPEVKAAMRALQEVDRLPVPPDLRRWTPESDMLDWLGGFFGFQEDNVRNQ

REHLVLLLANGMMHLFPSPSMPLDTLETSVVKMIRKKVTGNYVKWCKFIGCKNNLIKLVERRGGPSEREPQWHDLM

YTCLFLLIWGEAANLRFMPECLCFIYDNMLQELNKAIDGFTDNVELQGEIPTYAGPNGFLNNIIVPIYEVVKAEADSNN

GGAAPHSSWRNYDDMNEYFWSSRCFEQLRWPFSLNPKMNEDIPYNQRVNYESCSYLIKPLRERGYLNRGTKDQD

HKVGKTGFVEQRSFWYIFRSFDRIWVAHILVLQASVVTLWHNGGPPWIELQKPDPLARFLSIFITWSLLRVLQGLLDI

GSQYSLVSKETVFTGIRMILKPLVAAVWAILFIIYYRRMWWQRNIDQYWSGYANDRLHEYLYIAAAFIVPEVLALVLFIL

PWLRNFVENSNWRIFHALTWWFQTRQFVGRGLREGVMDNLKYALFWLSVLASKCAFSYWLQIRPLIAPTKQILRTK

NITYKWHEFFPNGSRAAIVVLWAPVLLIYFMDVQIWYSIWSSGVGAFVGLLQHLGEIRNVHQLRLRFKIFPSAFEFNL

MPPKQLQHTTLWENAKDLVERFRLRYGWSAIHEKVEWGQREGVQFAHVWNLIVNTFRDEDLISDRELELLEIPSGA

WRLSVFLWPSALLANQILQVLTNEVQYFKGDDTKLWGIISKHEYRRCAVTECYESIKHILLRRLLKVDSQEHKIIESVFK

EIDASIAHDRFTTSFVLQKILIVHDRVVKLIAVLMTKPTGGNIRKVVDALQNLYEDVVEDFIRDSSVKEIIRGQHLSTATN

KDTELFMNAVTLPSDDDAPFFKHLSRIHTTLSTREPFLNVPKGLEARRRISFFSNSLFMTMPRAPQVDRMLAFSVLTP

YYTEEVIFSSKQLKEENEDGITILFYLQRIFPEDWLNFLERMKKKGLLELNLWDTDDAIELRLWASYRGQTLARTVRG

MMYYERALQVQAFLDTATDTEMQGIKELLDAGSSPNRRGSFRSEHAYEERENRKNKQLQNLAAAGMKFTYVVTCQ

IYGNQKKTNDYKAADILRLMKTYHTGLRIAYVDEIKEEKGNKYYSVLVKYDKVLKREVEIYRIQLPGPLKLGEGKPENQ

NHALIFTRGDGVQTIDMNQEMYFEEAMKMRNLLEEFNRFRGIRKPTILGVREHVFTGSVSSLAWFMSAQETVFVTL

NQRVYANPLKIRMHYGHPDVFDRLWFLGRGGISKASRSINISEDIFAGFNCTLRGGTVTHHEYIQAGKGRDVGLNQI

AMFEAKVASGNGEQMLSRDVYRLGHHLDFFRMFSFYYTTVGFFVNNLIIVLTVFVFLWGRVYLALSGIEKSLTTGSNA

LSNAALTATLNQQLVVQLGLLTALPMLVEYALEHGFTTALWNMITMQLQLASLFFTFEMGTRSHYFGRTLLHGGAKYR

ATGRTFVVKHEKFAEIYRLYSRSHFTKGIELLMLLFCYLAYGVVSSSATYMLVMISSWFLAFTWIMAPFIFNPSGFDWL

KTVEDFDEFLQWIWFKGDIFVKPEQSWEIWWEGEQTHLKTTGLWGKLLDIVLDLRLFLFQYGIVYHLQITGNSTSVF

VYLLSWSYMLAAILLHLVISNASDRYAANKHGRYRLIQTVTIAVVAAIVIVLATRTNFTFLDILASFLAFLPTGWGILQICL

VLRRPFLENSKVWGTITAVARLYDLGMGMIIMAPVAFLSWLPGFQAMQTRILYNEAFSRGLQISRLFVGKKNTHID

>Phypa12-P.patens 1803

MGDLVYNIVPVDDLSSAEGHPALKFPEVRGAIFALRSVGDLRKPPHSPWRRDMDILDWLGCWFGFQASNVKNQRE

HLVLLLANAQMRSSPDSSDKLDGKVVRRIRQKVTKNYQSWCRFVGRDSAMSLPPGKRVGDERQELIYTSLYLLIWG

EAANLRFMPECLCFIFHNMAHELTTMLDKRSNGENSKPFTCEPNGFLKKVVSPLYEVVKAESKVNGAHSKWRNYD

DINEYFWSDRCFTHLKWPLDEASNFLVKPQPGKPLTRQKVGKTGFVEQRSFFHIFRSFDRLWIGYILVLQACIITLWN

GQQRAPWVELQNRDSLARLLTIFITWSGLRLFLALLDLVMQFKLVSRETWKTGLRMLLKVVAAIIWVGVFSILYRSMW

SKRHQDHSWSNAANTLFNRYIYAMAAFILPEALALALFIIPFARNFVEKSRFKLFHLLTWWFQSRIYVARGLREGLLDN

FKYTLFWILVLVSKFLFSYFLQLKPLITPTKEILSITDIQYRWHQIFKGGNRVAVLAIWAPVILIYFMDTQIWYTVWSALVG

ALVGLMDHLGEIRDVHQLKMRFKMFPHAVQFHLIPASESLKQQFGWTAYFRNFYHRTRLRYGTGVSPQEEQVEVKR

FSHIWNEILKIFREEDLISNRELELLEIPAQVWNISVFQWPSTLLANEIHTALNIVKNMHAEDKAVWKKIIKSDYRRCAVI

ESYESIRHILKNRILRKNSSDQILVSTLFDDHIDRALNQKPMGQFTEAFSLSKLPGVHQRILTLVNSMLALKMLLSKHD

KARELETARDAESEKARDTEHEKAREKVVSSLQDLWNFVTTEFAKKNERDRINASFEDKHFGPKALANLFNNSVEIP

HHKDESFYKQLKRLQTSLVTKDTLLDVPHGLEARRRISFFANSLFMTMPRAPQVERMNAFSVLTPYYHEEVIYSLKD

LNTANEDGITTLFYLQRVFPDDWNNFKERFGGSKESDEKFVNRMSGKDDPKKDDAKKKDPEKKRDEVGLELCLWA

SYRGQTLARTVRGMMYYERALEFQAFLDAAEIRDLDELLGYKEMMDRASSSTSEGSSRRRQGETSEQRESINEQR

KSAELAIAAMKFTYVVAAQVYGAQKKSGSNAAKSIAYLLELYKGLRIAYVDEVDTPAGKQYFSVLVKYDRVAKLEMEV

FRVQLPGPLKLGEGKPENQNHALIFTRGDAVQTIDMNQEMYFEEALKMRNLLEEFDKRHGVRKPTILGVREHVFTG

SVSSLAWFMSAQETSFVTLGQRVLANPLKIRMHYGHPDVFNRLWFLSRGGISKASKTINISEDIFAGFNCTLRGGTVT

HHEYIQAGKGRDVGLNQIAMFEAKVASGNGEQVLSRDVYRLGHRLDFFRMLSFYYTTVGFFINNLLVVLTVYAFLWG

RVYLAVSGVEASLQNSKILSNTALLASLNQQLIVQLGILTALPMIVENALEHGFTKALWEFFTMQMQLASVFFTFSMGT

RAHYFGRTVLHGGATYRATGRGFVVKHERFGKIYRLYRTSHFVKAIELIALLIIYRAYGSSRSSTTYLLISLSSWFLSLT

WLVGPFIFNPSGFDWLKTLEDFEDFMGWLKYKGGFIVDSEQSWESWWMEEQSHFKTTGILGKVADIILNLRYFFFQ

YGIVYQLNITATSQSIFVYVISWSYVVVAALIHFVLAVAGSRYSNRKHGLYRAIQAALITVIVAIIVVLKVFTSFSLRDLFTS

LLAFVPTGWGIIQILTVIRFRGLEKSFVWPVVVNVARLYEFGIGLIVLVPVAVLSWLPGFQAMQTRVLFNEGFSRGLQIS

QLFATVQKVKKSD

>2006986 Polytrichum commune 1093

TDKQDAIRFLPIWNAVIKSLREEDLISNREKVLLKMPPSNGEPDNLIHWPLFLLANKVHIAVELAVEHKNSQTELWAKICS
DEYMGYAVLETFQTLEPLLFNRILEDVKDELDRSSFVVHFKVSKLRDVLEKLRELTEQLGHEFVEERNVKAKAAFYALYEVV

MNDFLSDTIRERYSNLTYLKHATTEGRLFSDLKWLDKAGQKQAKRLTNLMQVQKVKDSEGKTKTLNTETVPHNLEARRR
LQFFTNSLFMHMPSPPPIRKMFSFCIEKLYKENEDGISILFYLQKIYPDEWQNFLERIGIIESTLQREIEQKKSENHDRFVLEL
RLWASYRGQTLARTVRGMMYYKKALEIQGAQEGAFSSVPDLEEGIPISLVEHHGSMERSARAQAELKFTYVVTCQIYGD
QKKKGKVQAADILYLMQQYESLRIAFIDVVETGAKDKKATYYSKLVKVDKNDPTGKDQEIYSIKLPGKVRLGEGKPENQN
HAIIFTRGDCIQTIDMNQDNFMEEAFKVRNLLEEFDQGHHGLRAPTILGVREHVFTGSVSSLAWFMSLQESSFVTLGQR
VLARPLKVRMHYGHPDVFDRIFHISRGGISKASRVINLSEDIFAGFNSTLRLGNVTHHEYVQVGKGRDVGLNQIALFEAKV
ASGNGEQSLSRDVYRLGQLLDFPRMLSFFYTSVGFYFLTLYVFLYGKAYLALSGVDASLKANNDILQNAALQSVLNTQFLF
QIGIFTAVPMIVNLILEQGILKAIISFCTMQLQLASVFFTFSLGTRTHYFGRTILHGGAKYRSTGRGFVVRHIKFAENYRMYS
RSHFTKALEVIMLLIVYLAYGAQNRSSVTFILLTFSSWFLAISWLYAPYIFNPSGFEWQKTVEDFDDWTNWLFYKGGVGV
QVDNSWEAWWFDEQDHIRTPRGRFWEFVLCSRFFFFQYGVVYSLNVTRGSNSILVYAYSWFVLFALVIIFKAFTISQKAS
ASFQLAVRLFQGLLFVSLITGVIVAIAKSPGDVFAVALALIPTGWGVLSFAIAARPWMEKLKLWKPIREVARAYDACMGMI
IFIPIAFLSWFPFISTFQTRLVFNQAFSRGLEISLILAGNRPN------------------------------------------------------------------------
---------------------------------------------------------------------------------------------------------------------------------------------
---------------------------------------------------------------------------------------------------------------------------------------------
---------------------------------------------------------------------------------------------------------------------------------------------
---------------------------------------------------------------------------------------------------------------------------------------------
---------------------------------------------------------------------------------------------------------------------------------------------
---------------------------------------------------------------------------------------------------------------------------------------------
-----

>2011769 Polytrichum commune 1263

SQLALFGYTFFWVLLIISKFAFSYYIQIEPLVAPTRAIMDQHSVTYTWHEFFPHASNNPGALISLWAPVIMVYFMDSQIWY
AVYSTIFGGISGSFRRLGEIRTLGMLRSRFSALPGAFNENLVPQETKKRRRGFSFRRAFEKVTPRESRQKASRFSQLWNEVI
TSFRDKERDLMLVPYSSDPHLNLVQWPPFLLASKVPIALQMAEQAAETGRAADLMRKIKTDEFMKSAVMECYESFKRVL
TVLIVGEVEARVIKGIFEQVESNVEKDTLLDNFKLKELPVLSVKFIELLELLEKNHSDQEAESARDLVVLKLQDMYEVVTRDI
MSEGMRDTCELSNGGEAREAGPSELFSGKGDEPAVQFPPPRTEAWIEQIKRLHLLLTERESAMDVPENLEARRRIAFFTN
SLFMDMPRAPRVRNMLSFSVLTPYYKEDVVYSKANLMTENEDGISVLFYLQKIYPDEWSNFLERVGLPPLTDETEILNNE
DLEDKLREWASFRGQTLSRTVRGMMYYRRALELQAFLDMASEDELVDGYKIITEASAETKKSQRSTWSQLQAIADMKF
TYVAACQNYGEQKRQSHHNAAEILKLMLNNPSLRVAYIDEVEERPKERTVKVYYSVLVKAVDGLDQEIYRIKLPGPVRLGE
GKPENQNHAIIFTRGEGLQAIDMNQDNYLEEAFKMRNLLQEFHEPHGVRPPTILGVREHIFTGSVSSLAWFMSNQETSF
VTIGQRVLASPLKVRFHYGHPDVFDRLFHITRGGMSKASRVINLSEDIFAGFNSILRRGNVTHHEYIQVGKGRDVGLNQIS
LFEAKIANGNGEQSLSRDIYRLGHRFDFFRMLSCYFTTVGYYFSTMIVVLTVYVFLYGRMYLALSGVDNSLVHLANNKALT
AAVQLGLLMALPMVMEIGLERGFRTAMSDFLTMQLQLASVFFTFSLGTKTHYFGRTILHGGAKYRPTGRGFVVRHERFA
ENYRLYSRSHFTKGIELLLLLIVYTIYVSRSASGAVTYILITFSMWFLVASWLFAPFLFNPSGFEWQKIVEDWDDWNKWISN
RGGIGVEGAKSWESWWDEEQEHLSFTGFRGRLIECILAMRFFLYQYGIIYHLNIVQTSNNLSITVYGLSWLVILAVIAVLKIV
SMGRDKFSADFQLMFRLLKACLFVGSISVIAVLHVRQFTVGDLFASILAFIPTGWALLQIAQACKPVVVKLGFWDSVKSFA
RGYEFMMGLVLFTPVAIFSWFPFVSEFQTRLLFNQAFSRGLQISRILAGRKKL--------------------------------------------------
---------------------------------------------------------------------------------------------------------------------------------------------
---------------------------------------------------------------------------------------------------------------------------------------------
---------------------------------------------------------------------------------------------------------------------------------------------
---------------------------------------------------------------------------------------------------------------------------------------------
---------------------------------------------------------------------------------------------------------------------------------------
