# Supplementary material for: Callose in leptoid cell walls of the moss Polytrichum and the evolution of callose synthase across bryophytes
Source: Front Plant Sci. 2024 Feb 7;15:1357324. doi: 10.3389/fpls.2024.1357324 (PMC10879339; doi:10.3389/fpls.2024.1357324)
Supplement: Supplementary file 2 [file DataSheet_2.pdf]

Supplementary Table S3: List of accession number and species used in the construction of bryophyte CalS and homologous CalS proteins tree.

Abbreviation	Species	Accession/Identification number	Length (aa)
**Sequences obtained from Phytozome database**			
Arath1	Arabidopsis thaliana	NP001322482.1	1950
Arath2	Arabidopsis thaliana	Q9SL03.3	1950
Arath3	Arabidopsis thaliana	Q9LXT9.3	1955
Arath4	Arabidopsis thaliana	Q9LTG5.2	1871
Arath5	Arabidopsis thaliana	Q3B724.1	1923
Arath6	Arabidopsis thaliana	Q9LYS6.2	1921
Arath7	Arabidopsis thaliana	Q9SHJ3.3	1958
Arath8	Arabidopsis thaliana	Q9LUD7.2	1976
Arath9	Arabidopsis thaliana	Q9SFU6.2	1890
Arath10	Arabidopsis thaliana	Q9SJM0.5	1904
Arath11	Arabidopsis thaliana	Q9S9U0.1	1768
Arath12	Arabidopsis thaliana	Q9ZT82.1	1780
Cerri1	Ceratopteris richardii	Ceric.01G012200.1	1972
Cerri2	Ceratopteris richardii	Ceric.32G064600.1	1947
Cerri3	Ceratopteris richardii	Ceric.1Z138200.1	1905
Cerri4	Ceratopteris richardii	Ceric.07G084800.1	1751
Cerri5	Ceratopteris richardii	Ceric.03G007700.1	1683
Cerri6	Ceratopteris richardii	Ceric.10G079800.3	1862
Cerri7	Ceratopteris richardii	Ceric.20G078000.1	1917
Cerri8	Ceratopteris richardii	Ceric.03G005500.1	1917
Cerri9	Ceratopteris richardii	Ceric.01G025800.1	1780
Cerri10	Ceratopteris richardii	Ceric.12G070800.1	1769
Cerri11	Ceratopteris richardii	Ceric.09G062400.1	1773
Cerr12	Ceratopteris richardii	Ceric.12G002500.1	1807
Cerri13	Ceratopteris richardii	Ceric.12G004600.1	1770
Cerri14	Ceratopteris richardii	Ceric.33G042600.1	1867
Ginbi1	Ginkgo biloba	Gb_06219	1937
Ginbi2	Ginkgo biloba	Gb_32712	1940
Ginbi3	Ginkgo biloba	Gb_08787	1933
Ginbi4	Ginkgo biloba	Gb_01752	1915
Ginbi5	Ginkgo biloba	Gb_22029	1912
Ginbi6	Ginkgo biloba	Gb_37962	1794
Ginbi7	Ginkgo biloba	Gb_29725	1791
Gnemo1	Gnetum montanum	scaffold631917.fgen	1961
Gnemo2	Gnetum montanum	scaffold69713.fgen	1955
Gnemo3	Gnetum montanum	scaffold25191	1998
Gnemo4	Gnetum montanum	scaffold201255.fgen	1911
Gnemo5	Gnetum montanum	scaffold1005685.fgen	1880
Gnemo6	Gnetum montanum	scaffold364231.fgen	1912

Gnemo7	Gnetum montanum	scaffold651053.fgen	1910
Gnemo8	Gnetum montanum	scaffold732327.fgen	1794
Gnemo9	Gnetum montanum	scaffold945599.fgen	1741
Gnemo10	Gnetum montanum	scaffold751913.fgen	1790
Marpo2	Marchantia polymorpha	Mapoly0183s0015.1	2015
Marpo4	Marchantia polymorpha	Mapoly0148s0007.1	1930
Marpo6	Marchantia polymorpha	Mapoly0031s0080.1	1782
Phypa1	Physcomitrium patens	Pp3c9_4140V3.1	1934
Phypa2	Physcomitrium patens	Pp3c25_6500V3.1	1986
Phypa3	Physcomitrium patens	Pp3c10_19330V3.1	2110
Phypa4	Physcomitrium patens	Pp3c4_1790V3.1	2020
Phypa5	Physcomitrium patens	Pp3c10_16400V3.1	2008
Phypa6	Physcomitrium patens	Pp3c4_17856V3.1	1960
Phypa7	Physcomitrium patens	Pp3c10_20860V3.1	1981
Phypa8	Physcomitrium patens	Pp3c20_20533V3.1	1979
Phypa9	Physcomitrium patens	Pp3c24_1600V3.1	1989
Phypa10	Physcomitrium patens	Pp3c23_14230V3.1	1784
Phypa11	Physcomitrium patens	Pp3c4_15060V3.1	1781
Phypa12	Physcomitrium patens	Pp3c15_22960V3.1	1803
Selbr1	Selaginella bryopteris	GEMU01097770.1	1933
Selbr2	Selaginella bryopteris	GEMU01098803.1	1930
Selbr3	Selaginella bryopteris	GEMU01009363.1	1907
Selbr4	Selaginella bryopteris	GEMU01003186.1	1921
Selbr5	Selaginella bryopteris	GEMU01096940.1	1742
Sphfa1	Sphagnum fallax	Sphfalx0127s0051.1	1934
Sphfa2	Sphagnum fallax	Sphfalx0025s0046.1	1938
Sphfa3	Sphagnum fallax	Sphfalx0297s0001.1	1974
Sphfa4	Sphagnum fallax	Sphfalx0070s0043.1	1963
Sphfa5	Sphagnum fallax	Sphfalx0069s0020.1	1963
Sphfa6	Sphagnum fallax	Sphfalx0004s0275.1	1976
Sphfa7	Sphagnum fallax	Sphfalx0020s0148.1	1759
Sphfa8	Sphagnum fallax	Sphfalx0158s0006.1	1756
Sphfa9	Sphagnum fallax	Sphfalx0003s0232.1	1151
**Sequences obtained from 1000 kp database**			
Andru1	Andreaea rupestris	WOGB 2015637	1628
Anoro1	Anomodon rostratus	VBMM 2004118	2022
Anoro2	Anomodon rostratus	VBMM 2004436	1593
Anoro3	Anomodon rostratus	VBMM 2008307	1979
Antag1	Anthoceros agrestis	BSNI 2012143	1932
Antag2	Anthoceros agrestis	TWUW 2052598	1937
Antag3	Anthoceros agrestis	BSNI 2053016	1788
Antag4	Anthoceros agrestis	TWUW 2052586	1788
Atran1	Atrichum angustatum	ZTHV 2009163	1934
Bryar1	Bryum argenteum	JMXW 2005869	1898
Buxap1	Buxbaumia aphylla	HRWG 2005493	1909

Conco1	Conocephalum conicum	ILBQ 2007264	1863
Conco2	Conocephalum conicum	ILBQ 2007227	1778
Dipfo1	Diphyscium foliosum	AWOI 2012069	1971
Dipfo2	Diphyscium foliosum	AWOI 2009411	1956
Dipfo3	Diphyscium foliosum	AWOI 2014174	1983
Dipfo4	Diphyscium foliosum	AWOI 2073991	1978
Fonan1	Fontinalis antipyretica	DHWX 2000693	1731
Fru1	Frullania	TGKW 2015918	1757
Hypsu1	Hypnum subimponens	LNSF 2070349	1634
Leidu1	Leiosporoceros dussii B	ANON 2008762	1935
Leidu2	Leiosporoceros dussii B	ANON 2036504	1786
Leidu3	Leiosporoceros dussii B	ANON 2036486	1756
Lejsp1	Lejeuneaceae sp	CHJJ 2004303	1917
Lejsp2	Lejeuneaceae sp	CHJJ 2004304	1510
Lejsp3	Lejeuneaceae sp	CHJJ 2005114	1409
Lejsp4	Lejeuneaceae sp	CHJJ 2005115	1763
Leual1	Leucobryum albidum	VMXJ 2005189	1956
Leual2	Leucobryum albidum	VMXJ 2005192	1724
Leual3	Leucobryum albidum	VMXJ 2019568	1985
Leual4	Leucobryum albidum	VMXJ 2011519	1725
Leual5	Leucobryum albidum	VMXJ 2015443	2005
Leual6	Leucobryum albidum	VMXJ 2014275	1772
Leual7	Leucobryum albidum	VMXJ 20132845	1788
Leusc1	Leucodon sciuroides	ZACW 2006709	1932
Leusc2	Leucodon sciuroides	ZACW 2006713	1931
Leusc3	Leucodon sciuroides	ZACW 2006711	1931
Leusc4	Leucodon sciuroides	ZACW 2017067	1982
Leusc5	Leucodon sciuroides	ZACW 2012855	1580
Leusc6	Leucodon sciuroides	ZACW 2012977	1779
Marpa1	Marchantia paleacea	HMHL 2009140	1909
Marpa2	Marchantia paleacea	IHWO 2011602	1909
Marpa3	Marchantia paleacea	HMHL 2009140	1914
Marpa4	Marchantia paleacea	HMHL 2011621	1779
Marpa5	Marchantia paleacea	IHWO 2068567	1779
Marpo1	Marchantia polymorpha	JPYU 2005872	1693
Marpo3	Marchantia polymorpha	JPYU 2009972	1595
Marpo5	Marchantia polymorpha	JPYU 2009036	1701
Megto1	Megaceros tosanus	UCRN 2054246	1933
Megto2	Megaceros tosanus	UCRN 2012030	1811
Megto3	Megaceros tosanus	UCRN 2011996	1776
Metcra1	Metzgeria crassipillis	NRWZ 2101168	1923
Necdo1	Neckera douglasii	TMAJ 2013573	1972
Necdo2	Neckera douglasii	TMAJ 2025004	1978
Necdo3	Neckera douglasii	TMAJ 2025003	1991
Necdo4	Neckera douglasii	TMAJ 2025002	2004

Necdo5	Neckera douglasii	TMAJ 2010647	2044
Necdo6	Neckera douglasii	TMAJ 2017341	1976
Necdo7	Neckera douglasii	TMAJ 2020682	1455
Necdo8	Neckera douglasii	TMAJ 2020683	1772
Notae1	Nothoceros aenigmaticus	DXOU 238415	1589
Odopr1	Odontoschisma prostratum	YBQN 2130644	1931
Ortly1	Orthotrichum lyellii	CMEQ 201156	1996
Ortly2	Orthotrichum lyellii	CMEQ 2014675	1949
Ortly3	Orthotrichum lyellii	CMEQ 2012235	1973
Ortly4	Orthotrichum lyellii	CMEQ 2006259	1566
Ortly5	Orthotrichum lyellii	CMEQ 2007829*	1758
Parha1	Paraphymatoceros hallii	FAJB 2008240	1922
Parha2	Paraphymatoceros hallii	FAJB 2010681	1632
Parha3	Paraphymatoceros hallii	FAJB 2012507	1767
Phaca1	Phaeoceros carolinianus	RXRQ 2024670	1980
Phaca2	Phaeoceros carolinianus	WEEQ 2014103	1819
Phaca3	Phaeoceros carolinianus	WCZB 2120236	1928
Phaca4	Phaeoceros carolinianus	RXRQ 2016252	1937
Phaca5	Phaeoceros carolinianus	WCZB 2120204	1781
Phaca6	Phaeoceros carolinianus	WEEQ 2091206	1608
Phaca7	Phaeoceros carolinianus	ZFRE 2118264	1545
Phaca8	Phaeoceros carolinianus	WCZB 2120211	1768
Phaca9	Phaeoceros carolinianus	RXRQ 2025306	1768
Phaca10	Phaeoceros carolinianus	WEEQ 2091224	1609
Phaco1	Phaeomegaceros coriaceus	AKXB 2007915	1933
Phaco2	Phaeomegaceros coriaceus	AKXB 2068347	1608
Phaco3	Phaeomegaceros coriaceus	AKXB 2011770	1772
Pelcf1	Pellia cf. Epiphylla	PIUF 2003440	1917
Pelcf2	Pellia cf. Epiphylla	PIUF 2014265	1929
Pelcf3	Pellia cf. Epiphylla	PIUF 2011212	1770
Pelne1	Pellia neesiana	JHFI 2114436	1918
Pelne2	Pellia neesiana	JHFI 2114429	1770
Polco1	Polytrichum commune	SZYG 2011769	1263
Polco2	Polytrichum commune	SZYG 2006986	1093
Porna1	Porella navicularis	KRUQ 2019279	1919
Porna2	Porella navicularis	KRUQ 2017364	1927
Porna3	Porella navicularis	KRUQ 2016796	1773
Porpi1	Porella pinnata	UUHD 2150804	1930
Porpi2	Porella pinnata	UUHD2011853	1788
Pseel1	Pseudotaxiphyllum elegans	QKQO 2008279	1986

Pseel2	Pseudotaxiphyllum elegans	QKQO 2005131	1758
Pseel3	Pseudotaxiphyllum elegans	QKQO 2010537	1779
Racva1	Racomitrium varium	RDOO 2020438	1934
Racva2	Racomitrium varium	RDOO 2019568	1999
Racva3	Racomitrium varium	RDOO 2020191	2005
Racva4	Racomitrium varium	RDOO 2016948	1987
Racva5	Racomitrium varium	RDOO 2006785	1759
Racva6	Racomitrium varium	RDOO 2006786	1772
Racva7	Racomitrium varium	RDOO 2006787	1780
Radli1	Radula lindenbergia	BNCU 2087280	1917
Radli2	Radula lindenbergia	BNCU 2018895	1941
Radli3	Radula lindenbergia	BNCU 2011463	1764
Rhylo1	Rhytidiadelphus loreus	WSPM 2000747	1886
Rhyse1	Rhynchostegium serrulatum	JADL 2005634	1982
Rhyse2	Rhynchostegium serrulatum	JADL 2005118	1776
Rhyse3	Rhynchostegium serrulatum	JADL 2005119	1836
Ricbe1	Riccia berychiana	WJLO 2001956	1977
Ricbe2	Riccia berychiana	WJLO 2001546	1782
Scane1	Scapania nemorosa	IRBN 2168378	1923
Scane2	Scapania nemorosa	IRBN 2168395	1776
Schsp1	Schistochila sp.	LGOW 2105553	1915
Schsp2	Schistochila sp.	LGOW 2021351	1773
Schfa1	Schwetschkeopsis fabronia	IGUH 2003399	1932
Schfa2	Schwetschkeopsis fabronia	IGUH 2003402	1997
Schfa3	Schwetschkeopsis fabronia	IGUH 2003398	1961
Schfa4	Schwetschkeopsis fabronia	IGUH 2021788	1964
Schfa5	Schwetschkeopsis fabronia	IGUH 2021779	1982
Schfa6	Schwetschkeopsis fabronia	IGUH 2021788	1979
Schfa7	Schwetschkeopsis fabronia	IGUH 2019275	1994
Schfa8	Schwetschkeopsis fabronia	IGUH 2021864	1971
Schfa9	Schwetschkeopsis fabronia	IGUH 2016263	1841

Schf10	Schwetschkeopsis fabronia	IGUH 2016264	1692
Schfa11	Schwetschkeopsis fabronia	IGUH 2013096	1779
Sphpa1	Sphagnum pallustre	RCBT 2000511	1769
Sphpa2	Sphagnum pallustre	RCBT 2000510	1758
Sphpa3	Sphagnum pallustre	RCBT 2000508	1759
Sphre1	Sphagnum recurvatum	UHLI 2006658	1706
Takle1	Takakia lepidozioides	SKQD 2006862	1568
Takle2	Takakia lepidozioides	SKQD 2009602	1762
Timau1	Timmia austriaca	ZQRI 2012342	1985
Timau2	Timmia austriaca	ZQRI 2068262	1667
