# Supplementary figures and images for: Callose in leptoid cell walls of the moss Polytrichum and the evolution of callose synthase across bryophytes
Source: Front Plant Sci. 2024 Feb 7;15:1357324. doi: 10.3389/fpls.2024.1357324 (PMC10879339; doi:10.3389/fpls.2024.1357324)

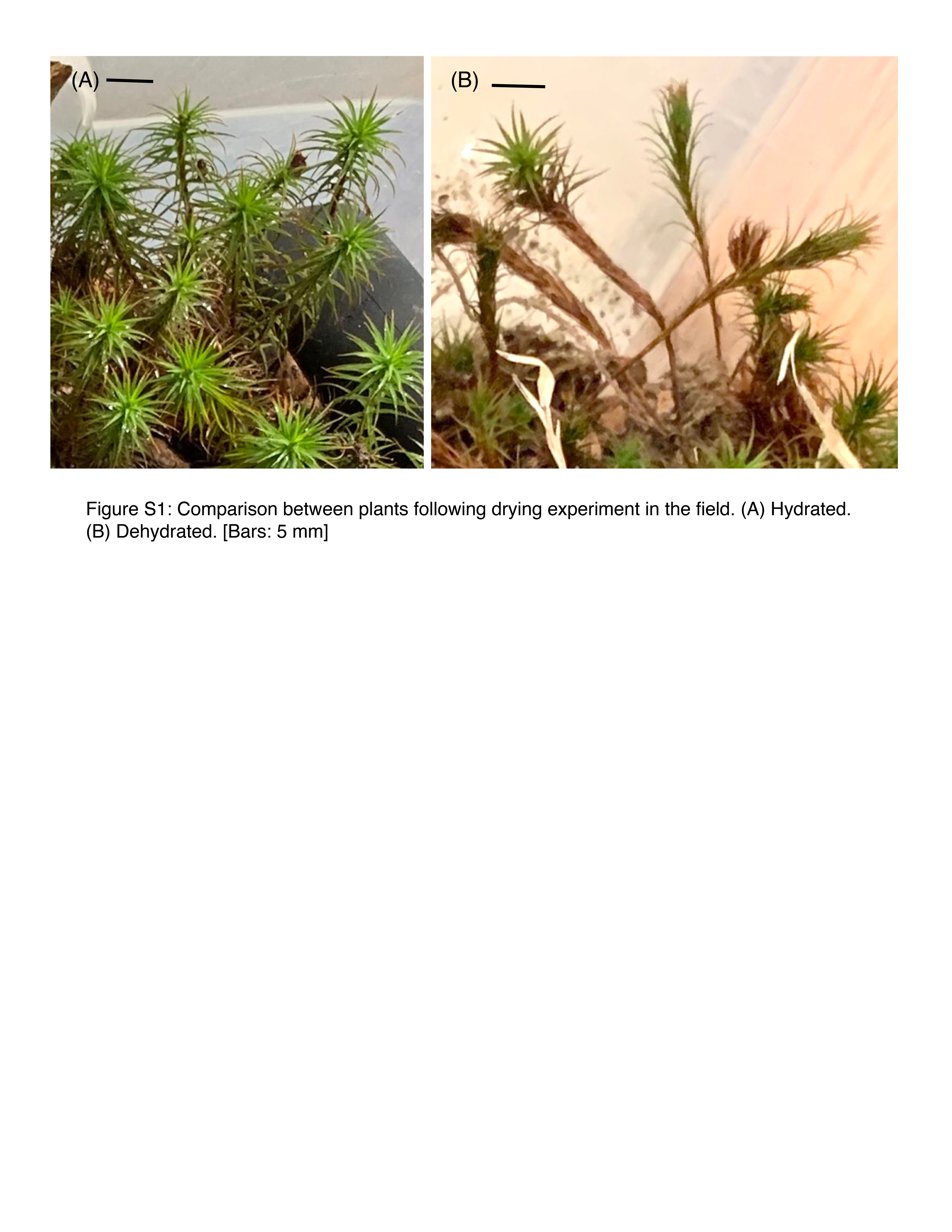

Supplement: Supplementary file 3 [file Image_1.jpeg]
